# Photocatalytic Late-Stage Functionalization of Sulfonamides via Sulfonyl Radical Intermediates

**DOI:** 10.1021/acscatal.2c01442

**Published:** 2022-05-06

**Authors:** Michael J. Tilby, Damien F. Dewez, Loïc R. E. Pantaine, Adrian Hall, Carolina Martínez-Lamenca, Michael C. Willis

**Affiliations:** † Department of Chemistry, Chemistry Research Laboratory, University of Oxford, Mansfield Road, Oxford OX1 3TA, U.K.; ‡ UCB Biopharma SPRL, 1420 Braine-l’Alleud, 1070 Brussels, Belgium; § Neuroscience Medicinal Chemistry, Janssen Research and Development, 2340 Beerse, Belgium

**Keywords:** sulfonamides, sulfones, photocatalysis, radicals, late-stage

## Abstract

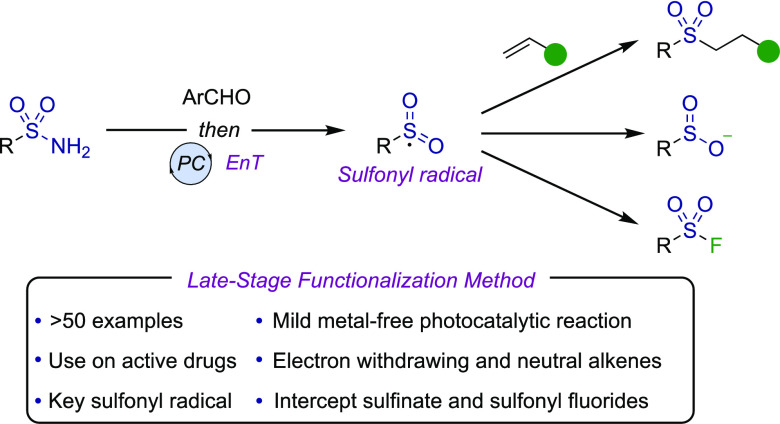

A plethora of drug molecules and agrochemicals contain the sulfonamide functional group. However, sulfonamides are seldom viewed as synthetically useful functional groups. To confront this limitation, a late-stage functionalization strategy is described, which allows sulfonamides to be converted to pivotal sulfonyl radical intermediates. This methodology exploits a metal-free photocatalytic approach to access radical chemistry, which is harnessed by combining pharmaceutically relevant sulfonamides with an assortment of alkene fragments. Additionally, the sulfinate anion can be readily obtained, further broadening the options for sulfonamide functionalization. Mechanistic studies suggest that energy-transfer catalysis (EnT) is in operation.

## 1 Introduction

Sulfur functional groups have a long history as pharmaceuticals, with some of the first antibiotics containing primary sulfonamides.^1^ For example, it is nearly a century since the discovery of the seminal drug Prontosil. The ongoing role of sulfonamides as valuable pharmacophores is evident from considering the top-selling 200 small molecule pharmaceuticals of 2020,^2^ where almost 10% contain a sulfonamide. The continued popularity of incorporating sulfonamide motifs in blockbuster drugs can be attributed to several features, including their high hydrolytic stability,^3^ their ability to interact with amino acids and metal ions in a biological setting,^4^ and the favorable physiochemical properties they often confer. These positive attributes mean that sulfonamides are now prominent in company compound collections.^5^ With the importance of aza-sulfur pharmacophores continually growing,^6^ a diverse selection of methods has been established for their synthesis,^7^ with approaches to sulfonamides being most common.^8^ Furthermore, endeavors to demonstrate the synthetic utility of sulfonamides in hydroaminations,^9^ as directing groups in C–H activation,^10^ and as precursors for C–N coupling reactions, have been reported.^11^ However, it is intriguing to note that in many medicinal agents, construction of the sulfonamide unit is often performed early in the synthetic route and that their latent reactivity is seldom exploited. This has resulted in sulfonamides being viewed as terminal functional groups.

**Figure 1:**
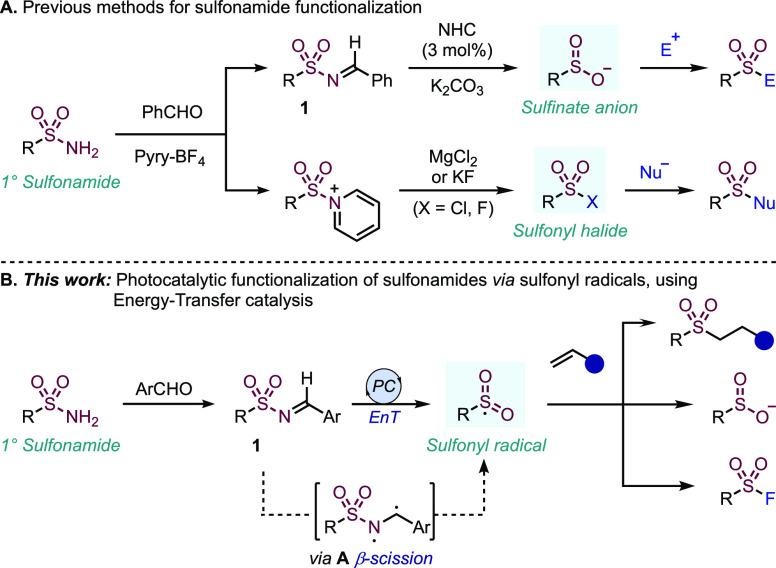
Late-stage functionalization of sulfonamides via nucleophilic,^5,12^ electrophilic,^13^ and neutral reactive intermediates.

Recent innovations from Fier and Maloney,^5,12^ and Cornella,^13^ have exploited sulfonamides in the late-stage generation of synthetically useful sulfonyl intermediates, reinvigorating the view of sulfonamides as useful synthetic functional groups (Figure 1A). In particular, Fier and Maloney have demonstrated that complex drug scaffolds featuring a primary sulfonamide can be activated by conversion to *N*-sulfonylimines (**1**), which under NHC-catalysis generate nucleophilic sulfinate anions.^5^ These intermediates can then be combined with a broad range of electrophilic reagents. In an alternative strategy developed by the Cornella laboratory, primary sulfonamides are converted to sulfonyl chlorides^13^ or fluorides, by way of pyridinium intermediates;^14^ functionalization with a selection of nucleophiles, including complex amines, was then possible. Extensions of these influential reports include the use of alternative activators,^15^ as well as applications to radiolabeling.^16^

Herein, we present a complementary approach to these elegant strategies; rather than generating formally nucleophilic or electrophilic sulfonyl-derivatives, we access neutral sulfonyl radical intermediates, and in doing so unlock underexplored reactivity (Figure 1B).^17^ Using mild photocatalytic conditions, readily formed *N*-sulfonylimines (**1**) function as sulfonyl radical precursors.^18^ We exploit these versatile reactive intermediates in the hydrosulfonylation of alkenes,^19^ thus diversifying the late-stage functionalization opportunities for sulfonamides and accessing new regions of chemical space. We also show that sulfonyl radicals can be used to intercept sulfinate anions and sulfonyl fluorides, providing the opportunity to use the prior reaction pathways for additional late-stage functionalization.

## 2 Results and Discussion

Although a variety of sulfonyl radical precursors are known, such as sulfonyl azides, sulfonyl chlorides, and sulfinate salts,^18^ these species do not enjoy the privileges of sulfonamides; in particular, they are generally reactive, which can affect long-term storage. In addition, they do not feature in medicinal agents and are therefore not amenable to late-stage functionalization. The primary consideration of our reaction design was the plausibility of generating a sulfonyl radical directly from sulfonamides. A challenge associated with the direct single electron reduction of sulfonamides is their low redox potentials (*ca*. −2.3 V).^20^ The photochemical methods that do achieve these direct reductions^21^ present a quandary, resulting from the precarious nature of sulfonamide fragmentation, which potentially produces the sulfinate anion and not the required radical. Hence, harnessing sulfonyl radicals via this approach requires specific substituents,^21g^ thus limiting applicability as a late-stage functionalization tool.^22^ To address this constraint, our design focused on using an aldehyde activator to form aldimine **1**,^23^ facilitating a controlled fragmentation to the key sulfonyl radical intermediate. Although *N*-sulfonylimines can be viewed as inherently electron-deficient in nature, we speculated that single electron reduction could again result in significant quantities of the sulfinate anion. Therefore, to circumvent this possibility, we considered an unconventional strategy of exciting the C=N bond to a biradical state **A**, which we proposed would readily undergo β-scission (Figure 1B).^24^ Currently, the photolysis of *N*-alkylimines^25^ requires direct UV irradiation. However, we were inspired by related oxime chemistry,^26^ in which recent breakthroughs have used accessible visible-light triplet energy-transfer (EnT) catalysis to achieve oxime fragmentation^27^ and cyclization.^28^ Given that *N*-sufonylimines have similar photophysical properties,^29^ we speculated that this would be an attractive strategy to access sulfonyl radicals. Despite the challenge of developing a novel EnT process with *N*-sufonylimines, we reasoned that a substantial benefit would be that the steps following sulfonyl radical generation would be independent of initiation.^30^

Based on the previously outlined principles, we selected *p*-tolyl *N*-sulfonylimine (**1a**) and methyl vinyl ketone for initial evaluation (Table 1). The super silane reagent (TMS_3_Si-H) was expected to act as an efficient hydrogen atom donor (HAD) in the hydrosulfonylation as a result of the polarity match with an electrophilic radical intermediate.^19b^ Using the 5CzBN photocatalyst, with a low catalytic loading of 0.5 mol %, the desired adduct **2a** was obtained in 84% yield. Although the 5CzBN catalyst has seldom been applied as a photocatalyst to develop novel reactivity,^31^ the characterization of its photophysical properties implies that it has favorable redox potentials and excited triplet state energy (*E*_ox_* = −1.42 V, *E*_T_ = 2.83 eV).^32^ The role of the *para*-methoxy component of the imine was established by examining the unsubstituted system (**1b**) as a reference, which in comparison, delivered a slightly lower yield of product (73%). To probe the possibility of a single electron reduction mechanism, we introduced imines bearing electron-withdrawing substituents (**1c**, **1d**), which caused diminished yields (55% and <5%, respectively). Steric effects also had a negative impact with substrate **1e**, giving a 40% yield. The use of alternative electron-donating substituents (**1f**, **1g**) only led to inferior yields. Whilst these results imply a delicate balance for the nature of the imine component, we were content with the use of the simple *para*-methoxy substrate, being derived from a commercial, readily available aldehyde. Further control reactions established that a photocatalytic procedure was in operation as the use of catalyst and light were essential for reactivity (entries 1 and 2). The reaction could not be initiated thermally at 80 °C (entry 3). Additionally, product formation was significantly reduced without the presence of a suitable HAD (entry 4). The solvent tolerance of the reaction was also explored (entries 5–9), and several common solvents, including acetonitrile, dichloromethane, and ethyl acetate, were capable of supporting yields >60%. Using tetrahydrofuran (THF), a 54% yield was observed, which was further lowered in the polar protic solvent MeOH to 44%. Taken together, these results indicate a useful solvent-independence. Finally, it is notable that a base is not needed to achieve high yields.

**Table 1:**
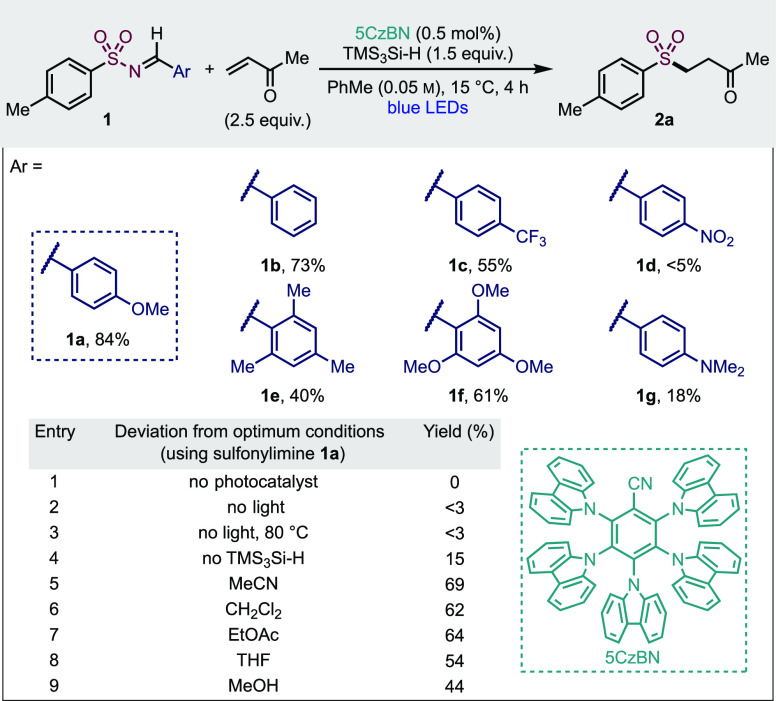
Optimization of Reaction Conditions for the Formation of Sulfone 2a^a^

a Reactions conducted on 0.1 mmol scale. Yields of **2a** were calculated from ^1^H NMR spectroscopy analysis of crude reaction mixtures using 1,3,5-trimethoxybenzene as the internal standard. 5CzBn = 2,3,4,5,6-penta(9*H*-carbazol-9-yl)benzonitrile.

Having an optimized system in hand, we next explored the variety of sulfonamides, in the form of *N*-sulfonylimines, that could be employed. These were used in combination with methyl vinyl ketone and provide an assessment of the functional group tolerance of the reaction (Table 2). Simple aryl sulfones (**2a–c**) were isolated in excellent yields. Sulfone **2a** was also obtained in 91% yield when the reaction was performed on 10 times the initial scale, whilst retaining a 0.5 mol % catalyst loading. The sterically congested mesityl substrate gave a reduced yield of 31% (**2d**). A selection of electron-poor arenes was well-tolerated, with fluoro-, trifluoromethyl-, and cyano-substituents delivering high isolated yields (**2e–g**). Synthetically useful ketone, ester, and bromo substituents were readily tolerated (**2h–l**), albeit with the *meta*- and *ortho*-bromo examples delivering lower yields (**2k**, **l**). Electron-rich aryl sulfones (**2m**, **n**) were also obtained in useful yields. The photocatalyzed conditions are suitable for the preparation of several alkyl sulfones (**2o–s**). As before, increasing steric congestion around sulfur led to a decreased yield for *tert*-butyl example **2r**. The cyclopropyl-derived substrate **2s** was effective, and no radical ring-opening was observed.

**Table 2:**
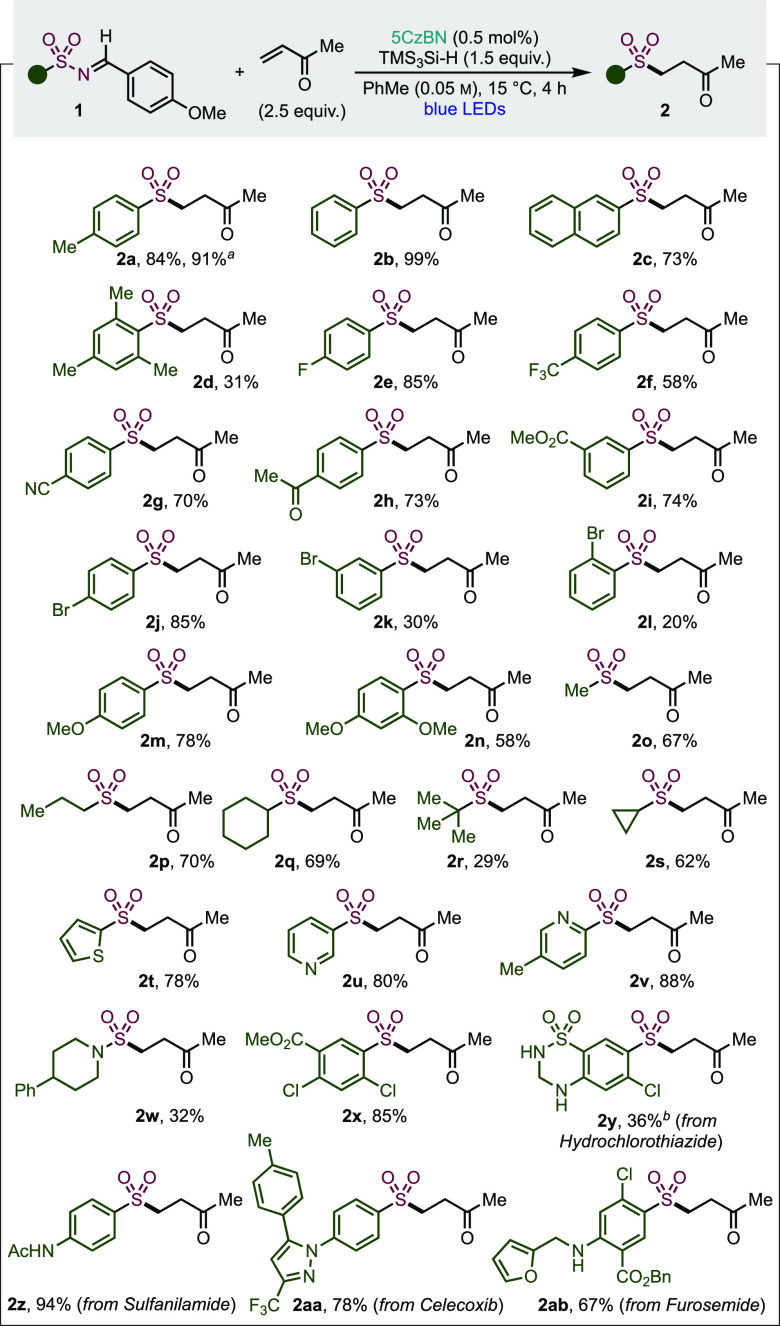
Scope of Sulfonyl Imines in the Photocatalytic Late-Stage Functionalization of Sulfonamides

a At 1.0 mmol scale.

b Reaction conducted in acetonitrile (0.05 M).

To further highlight the “late-stage” abilities of our method, medicinally relevant heterocycles and drug fragments were also functionalized, starting with the thiophene (**2t**), 3-pyridine (**2u**), and 2-pyridine (**2v**) heteroaromatics, all in excellent yields. The 2-pyridine substrate showed no SO_2_ extrusion and delivered the expected β-ketone; analogous motifs have successfully been used in palladium-catalyzed cross-coupling reactions.^33^ Sulfamides are a further important aza-sulfur pharmacophore^34^ and have been incorporated into a variety of pharmaceuticals. Using a sulfamide substrate under our standard reaction conditions delivered sulfonamide **2w** in 32% yield, thus establishing applicability to nonsulfonamide substrates. The sulfonamide of the densely functionalized arene **2x** is a common building block for chlorosulfonamide diuretics.^35^ The diuretic drug hydrochlorothiazide delivered complex sulfone **2y** in 36% with acetonitrile as the solvent because of solubility issues using toluene. Sulfonyl imines derived from the antibacterial sulfanilamide, COX2 inhibitor Celecoxib, and the diuretic medicine Furosemide, all provided the corresponding modified drugs (**2z**, **aa**, **ab**) in 94, 78, and 67% yields, respectively.

We next examined the scope of electron-deficient alkenes using the imine derived from Celecoxib **3** as the sulfonamide component (Table 3). An initial examination of the functional group tolerance demonstrated that a large set of unsubstituted acrylic derivatives could be used (**2aa**, **2ac**–**aj**), including nitriles, esters, secondary and tertiary amides, as well as a free carboxylic acid. We highlight examples **2ag**, for which complete selectivity for the acrylic alkene over the nonactivated alkene was achieved, and sulfone **2ah**, bearing an unaltered terminal alkyne, paving the way for further derivatization via CuAAC or Huisgen cycloaddition “click” reactions.^36^ Substituted acrylates were also competent reaction partners (**2ak–an**), where substrate **2al** exemplifies the use of a primary amide in excellent yield. We were also able to link together two bioactive molecules, Celecoxib and a complex estrone, providing sulfone **2ao** in 84% yield. The Celecoxib imine was also successfully reacted with amino acid derivatives (**2an**, **ap**). Finally, we evaluated the addition onto alkenes bearing small rings common in drug fragments; heterocyclic acrylamides (**2as**, **at**), as well as vinyl-pyridines (**2aq**, **ar**) and an electron-deficient styrene (**2au**) could all be incorporated, albeit with mixed efficiency.

**Table 3:**
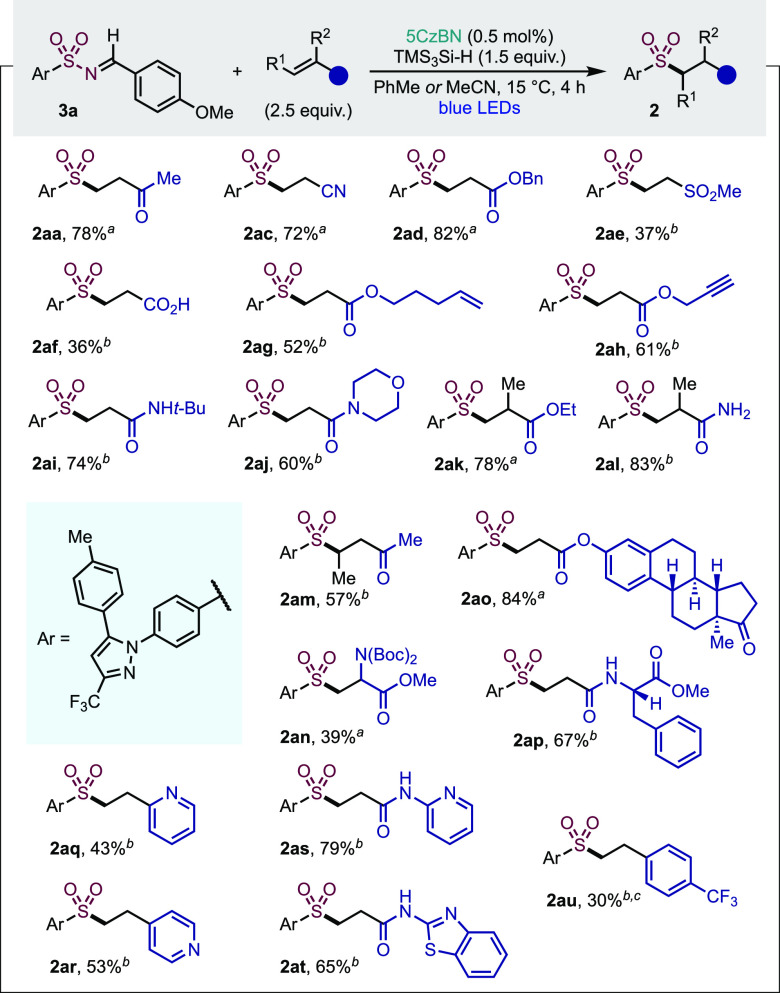
Scope of Electron-Poor Alkenes in the Photocatalytic Late-Stage Functionalization of Sulfonamides

a Reaction conducted in toluene (0.05 M).

b Reaction conducted in acetonitrile (0.05 M).

c Reaction conducted for 12 h.

A considerable challenge associated with many Giese reactions is addition to nonactivated alkenes.^37^ However, as we generate a putative electrophilic sulfonyl radical,^38^ we speculated that the addition to such alkenes should be possible.^19a,19b,39^ Realization of this reaction pathway would significantly expand the versatility of our late-stage functionalization method, and as such, we evaluated the use of neutral alkenes (Table 4). Under our previously optimized conditions, 3-butenylbenzene proved to be a poor reaction partner, delivering only traces of sulfone **4a**. This preliminary result most likely arises from a poor polarity-match between the HAD (i.e., TMS_3_Si-H) and the nucleophilic *C*-based radical obtained after the addition of the sulfonyl radical onto the nonactivated alkene.^19b^ We postulated that a solution would be to simply change the HAD for an appropriate polarity match, as this should operate orthogonally to the photocatalyst. A short optimization (see the Supporting Information for details) identified *p*-fluorothiophenol as a competent electrophilic H-atom donor, providing sulfone **4a** in synthetically useful yields, irrespective of which reaction partner was used in excess. Under these modified conditions, Celecoxib could be formally functionalized with a range of nonactivated alkenes to deliver the corresponding sulfones in moderate to good yields (**4a**–**h**). Functional groups such as a thioether (**4b**), an ether (**4c**), a protected amine (**4e**), a free alcohol (**4g**), and an alkyl bromide (**4h**) were well-tolerated. The hydrosulfonylation of sterically demanding (**4d**) and of 1,1- or 1,2-disubstituted alkenes (**4f**, **4g**) was also possible, with sulfone **4g** being obtained as a 5:1 separable mixture of regioisomers.

**Table 4:**
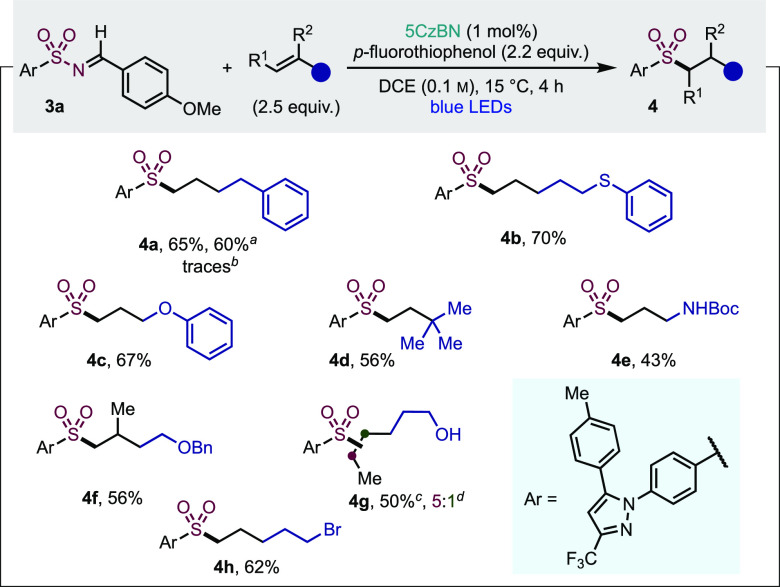
Scope of the Neutral Alkenes in the Photocatalytic Late-Stage Functionalization of Sulfonamides

a Using 1.65 equiv of sulfonyl imine **3a** and 1.0 equiv of alkene.

b Under previously optimized conditions.

c Combined yield.

d Determined by ^1^H NMR spectroscopy analysis of the crude reaction mixture. DCE = 1,2-dichloroethane.

**Figure 2:**
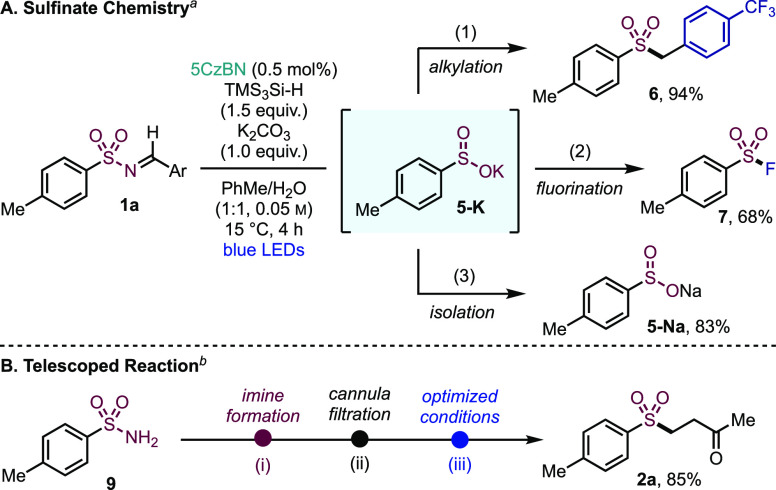
Photocatalyzed sulfinate salt formation and telescoped reaction from a primary sulfonamide. ^*a*^Reaction conditions: (1) *p*-CF_3_-benzyl bromide (1.5 equiv), TBAB (20 mol %), 100 °C, 24 h; (2) NFSI (1.5 equiv), K_2_CO_3_ (1.1 equiv), solvent switch to THF/H_2_O (10:1, 0.2 M), rt., 12 h; (3) acidify using 2 M aq. H_3_PO_4_, extract (Na_2_CO_3_). ^*b*^Reaction conditions: step (i) sulfonamide **9** (1.0 equiv), *p*-anisaldehyde (1.0 equiv), Amberlyst 15 (5 mg/mmol), PhMe (0.1 M), Dean-Stark, 12 h; step (iii) MVK (2.5 equiv), 5CzBN (0.5 mol %), TMS_3_Si-H (1.5 equiv), PhMe (0.05 M), blue LEDs, 15 °C, 4 h.

Inspired by the recent breakthroughs from Fier and Maloney,^5,12^ and Perry,^15^ we wanted to expand the utility of our photocatalyzed process to access sulfinate reactivity using our activation mode. We reasoned that this should be possible by combining the sufonyl radical with an appropriate HAD, and then effecting deprotonation. In the event, the formation of a sulfinate salt (**5-K**) was achieved by direct trapping of the sulfonyl radical, generated from sulfonylimine **1a**, with TMS_3_Si-H under basic biphasic conditions (Figure 2A). The potassium sulfinate thus generated could then be smoothly alkylated to benzylic sulfone **6**. Given the utility of sulfonyl fluorides as stable sulfur(VI)-electrophiles,^40^ as well as their role as chemical probes^41^ and covalent inhibitors,^42^ a route toward these was also developed. Here, we used *N*-fluorobenzenesulfonamide (NFSI), exemplified by compound **7** obtained in 68% yield. The sodium sulfinate salt **5-Na** could also be isolated in 83% yield, following simple acidification and extraction using aq. Na_2_CO_3_, paving the way to a plethora of derivatization reactions.^43^ Finally, the photocatalyzed reaction could also be telescoped with imine formation, therefore providing a direct route from sulfonamide **9** to sulfone **2a** without the isolation of an imine intermediate (Figure 2B). Imine formation was achieved using acidic resin catalysis, and cannula filtration onto the photocatalytic system then allowed radical generation and functionalization, delivering sulfone **2a** in 85% yield on 0.4 mmol scale.

**Figure 3:**
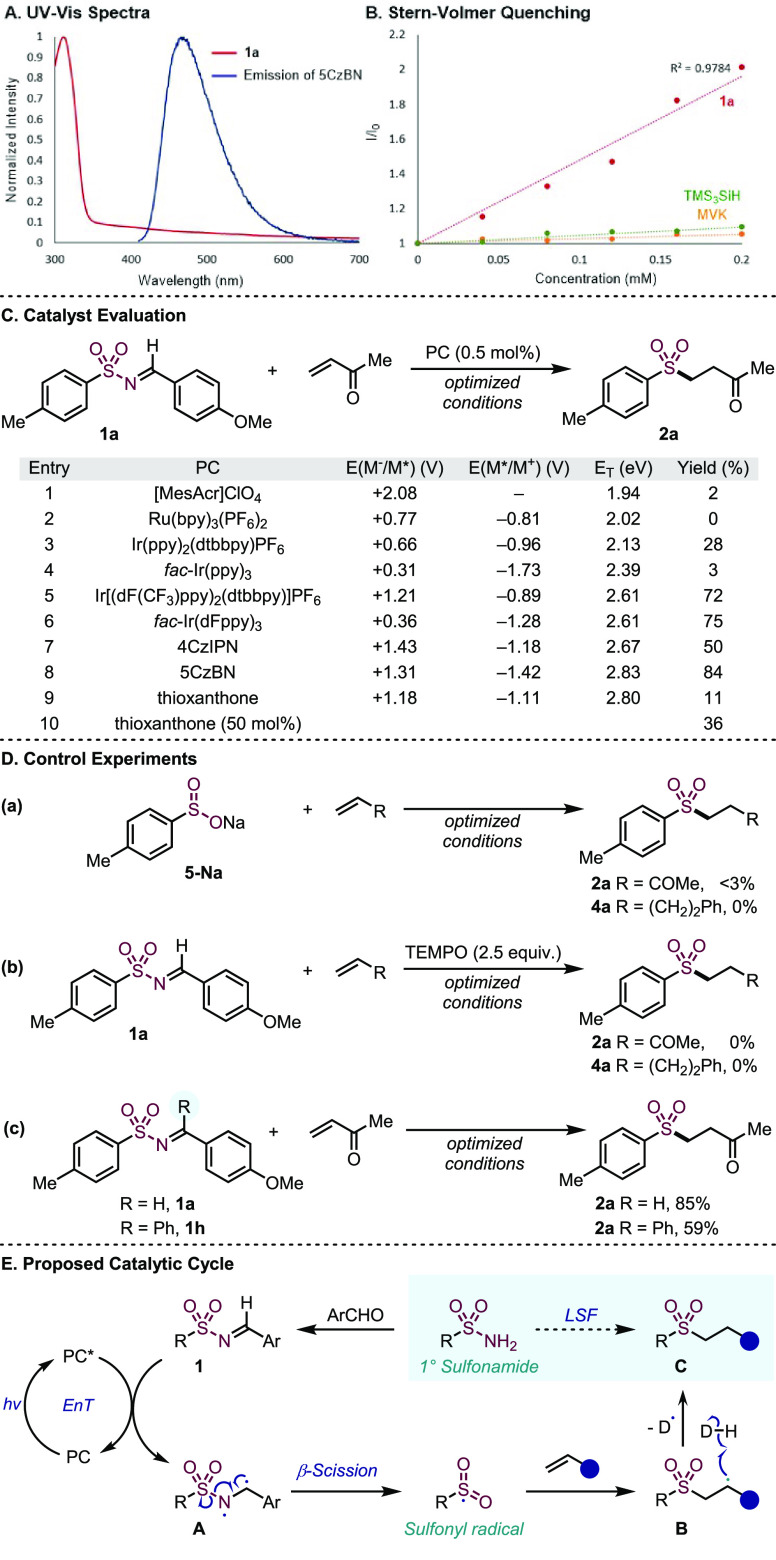
Mechanistic investigations. (A) Absorption spectra of **2a** in toluene and emission spectra of 5CzBN in toluene using excitation at 400 nm. (B) Stern–Volmer quenching of 5CzBN in toluene using excitation at 400 nm. (C) Further screening of photocatalysts using a 0.1 mmol scale with MVK (2.5 equiv), TMS_3_Si-H (1.5 equiv), PhMe (0.05 M), blue LEDs, 15 °C, 4 h. (D) Control reactions using a 0.1 mmol scale with MVK (2.5 equiv), 5CzBN (0.5 mol %), TMS_3_Si-H (1.5 equiv), PhMe (0.05 M), blue LEDs, 15 °C, 4 h, or 3-butenylbenzene (2.5 equiv), 5CzBN (1 mol %), *p*-fluorothiophenol (2.2 equiv), DCE (0.1 M), blue LEDs, 15 °C, 4 h. With all potentials and triplet energies measured in MeCN. (E) Proposed catalytic cycle.

From the previous experimental observations, we reasoned that the success of the developed method was grounded in the application of visible-light EnT catalysis; accordingly, several experiments were conducted on the optimized system to probe the mechanism (Figure 3). First, in order to determine if light could be directly exciting the *N*-sulfonylimine (**1a**), we measured the absorption spectra in toluene (Figure 3A). From this, λ_max_ = 341 nm was obtained, which is well below the wavelength of the visible light used (450 nm). The absorption band was then compared to the emission spectra of the 5CzBN catalyst, indicating no significant overlap, and hence, a Förster resonance energy transfer is unlikely to be in operation. To ensure that the reaction mechanism was initiated by the interaction of the photocatalyst and the imine, a series of Stern–Volmer quenching experiments was conducted (Figure 3B). The results indicated that substrate **2a** efficiently quenches the excited state of 5CzBN (*K*_SV_ = 4.811 mM^–1^). From these findings, we proposed that the reaction is initiated via a Dexter triplet-triplet energy transfer. To provide support for this mechanism, the computed solvated triplet energy of **2a** (see the Supporting Information) was determined to be 2.64 eV, which is close to that of the catalyst. However, the reduction potential of **2a**, as measured by cyclic voltammetry (see the Supporting Information), was found to be −1.44 V (vs SCE in MeCN). Therefore, to investigate the possibility of a photoredox pathway, various catalysts were tested in the reaction (Figure 3C). These results show a trend that catalysts with higher triplet energies produced higher yields of the product, with catalysts with *E*_T_ > 2.6 eV providing yields ≥50%. Notable results include the catalyst Ir[dF(CF_3_)ppy)_2_(dtbbpy)]PF_6_, which is weakly reducing in the excited state, but gave a high yield (entry 5), as did *fac*-Ir(dFppy)_3_, a catalyst with an identical triplet energy (Entry 6). In comparison, the catalyst *fac*-Ir(ppy)_3_, which theoretically has an excited state able to reduce imine **2a**, gave a negligible yield. When thioxanthone, a catalyst commonly used in energy-transfer catalysis,^44^ was employed, an 11% yield of adduct **2a** was achieved; using a 50 mol % loading of thioxanthone increased the yield to 36%. These low yields are attributed to the poor interaction of thioxanthone and 450 nm light; however, given the low cost of thioxanthone and the known EnT reactivity, these results are encouraging for further development. Next, to confirm that sulfinate formation was not responsible for the product, sodium sulfinate was used with the optimized reaction conditions (Figure 3D). Furthermore, the presence of radical intermediates was inferred from the addition of TEMPO to the reactions, resulting in no product formation. Finally, although elimination to form a nitrile-derived byproduct is unlikely due to no base being present in the reaction, this was further probed by the use of ketimine substrate **1h**. Using the optimized reaction conditions, ketimine **1h** was a competent reaction component, providing the expected addition product **2a** in 59% yield (reaction c). Overall, these mechanistic experiments allow a tentative catalytic cycle to be proposed (Figure 3E). The cycle is initiated by the irradiation of the photocatalyst resulting in an excited triplet state, which is subsequently quenched by *N*-sulfonylimine (**2a**) in a triplet-triplet energy transfer. This step provides the biradical **A**, which can undergo a controlled β-scission to the key sulfonyl radical intermediate. In the majority of reactions presented here, this sulfonyl radical undergoes addition to an alkene, generating intermediate **B**, followed by a hydrogen atom transfer (HAT) to the product **C**. The efficiency of the final step is reliant on a polarity match rather than a redox quenching cycle. We have not been able to establish the fate of the iminyl and tristrimethylsilyl radicals that would be formed from our proposed mechanism; however, both of these reactive species are off-cycle and susceptible to multiple decomposition pathways.^45^

## 3 Conclusions

In conclusions, within this report, we have detailed a new strategy for the photoinitiated late-stage functionalization of sulfonamides via key sulfonyl radical intermediates, allowing access to an underexplored category of radical reactions. This principle has predominantly been demonstrated with the synthesis of complex sulfones via a hydrosulfonylation process. A diverse array of pharmaceutically relevant molecules was subjected to the reaction conditions. A display of the reaction’s capabilities can be inferred from the rapid construction of 28 derivatives of the COX-2 inhibitor, Celecoxib, including those coupled with complex alkene building blocks. A key component of the transformation’s success is ascribed to the reaction design, based on using an organo-photocatalyst at low catalyst loading to enable EnT catalysis. Although EnT catalysis on *N*-sulfonylimines has previously not been reported, utilizing its key concepts has allowed a process, which is applicable to both electron-deficient and electron-neutral alkenes, with a broad functional group tolerance. This method delivers a medicinal chemistry-relevant synthetic process and also constitutes a novel reactivity mode for imines, exemplified here in a platform for sulfonyl radical generation.

## Supporting Information Available

Experimental procedures, spectral characterization, and additional data (PDF)
