# Supplementary material for: Photocatalytic Late-Stage Functionalization of Sulfonamides via Sulfonyl Radical Intermediates
Source: ACS Catal. 2022 May 6;12(10):6060–7. doi: 10.1021/acscatal.2c01442 (PMC9127806; doi:10.1021/acscatal.2c01442)
Supplement: Supplementary file 1 — cs2c01442_si_001.pdf [file cs2c01442_si_001.pdf]

Supporting Information for:

Photocatalytic Late-Stage Functionalization of Sulfonamides via Sulfonyl Radical Intermediates

Michael J. Tilby,[a] Damien F. Dewez,[a] Loïc R. E. Pantaine,[a] Adrian Hall,[b] Carolina Martínez-Lamenca,[c] Michael C. Willis*,[a]

[a] Department of Chemistry, Chemistry Research Laboratory, University of Oxford, Mansfield Road, Oxford OX1 3TA, United Kingdom. *Email:* michael.willis@chem.ox.ac.uk
[b] UCB Biopharma SPRL, 1420 Braine-l'Alleud, Belgium
[c] Neuroscience Medicinal Chemistry, Janssen Research and Development, 2340 Beerse, Belgium

Table of Contents

1. General Information S3

2. Optimisation of the Addition to Neutral Alkenes S5

2.1. HAD Screen S5

2.2. Solvent Screen S6

2.3. Fine-Tuning of the Conditions and Control Reactions S7

3. Experimental Procedures S8

3.1. Synthesis of Photocatalyst S8

3.2. Synthesis of Alkenes S8

3.3. Synthesis of Primary Sulfonamides S8

3.4. Synthesis of *N*-sulfonylimines S16

3.5. Photochemical Functionalisation with Activated Alkenes S34

3.6. Photochemical Functionalisation with Neutral Alkenes S60

3.7. Photochemical Functionalisation *via* Sulfinate Salts S66

3.8. Telescoped Reaction from Sulfonamide S68

4. Mechanistic Experiments S70

4.1. UV-Vis Experiments S70

4.2. Stern-Volmer Quenching S71

4.3. Computational Experiments S72

4.4. Cyclic Voltammetry S73

4.5. Control Reactions S75

5. References S77

6. HPLC Data S81

7. NMR Spectra S82

8. Computational Coordinates S232

1. General Information

Reactions were conducted under an inert atmosphere of nitrogen, with anhydrous solvents, unless otherwise stated. Anhydrous toluene, acetonitrile, THF and CH_2Cl_2 were obtained by passing through alumina columns using an Innovative Technology Inc. PS-400-7 solvent purification system, these solvents were then degassed with nitrogen prior to use, all other solvents were purchased as anhydrous from a commercial source. All glassware was oven dried (> 80 °C) for at least 16 h prior to use and allowed to cool to room temperature under a positive pressure of nitrogen. All reagents were purchased from Sigma-Aldrich Chemical Co. Ltd., Alfa Aesar, Acros Organic Ltd., Fluorochem Ltd., Strem Chemicals Inc. or Insight Biotechnology Ltd. and were used as supplied unless stated otherwise.

^{1}H NMR spectra were recorded on a Bruker AVIII400 (400 MHz), AVII500 (500 MHz) or NEO600 (600 MHz) spectrometer, ^{13}C NMR were recorded on a Bruker AVIII400 (101 MHz), AVII500 (126 MHz), AVIIIHD500 (126 MHz) or NEO600 (600 MHz) spectrometer and ^{19}F NMR were recorded on a Bruker AVIII400 (377 MHz) or NEO600 (565 MHz) spectrometer. Chemical shifts (δ_H, δ_C and δ_F) are quoted in ppm and relative to tetramethylsilane (δ_H = 0 ppm) and referenced to the residual solvent peaks. Coupling constants (*J*) are quoted in Hz and rounded to the nearest 0.5 Hz, with the following abbreviations used: s - singlet, br. - broad, app. - apparent, d - doublet, t - triplet, q - quartet, p - pentet, m - multiplet, dd - doublet of doublets, etc. Spectra assignments were determined with the use of chemical shifts, coupling constants, two-dimensional (COSY, HSQC and HMBC) NMR spectroscopy and by comparison to spectra of other compounds.

Low resolution mass spectra were recorded on a Waters LCT Premier mass spectrometer. High resolution mass spectra were recorded by the mass spectrometry service at the S4 Chemistry Research Laboratory, University of Oxford, using a Bruker Daltonic μTOF spectrometer (ESI). *m/z* values are reported in Daltons (Da) and high-resolution values are calculated to four decimal places from the molecular formula, with all found values in a tolerance of 5 ppm.

Infrared spectra (IR) were recorded using a Bruker Tensor 27 Fourier Transform spectrometer using a diamond ATR module. Absorption maximum ν_{max} are given in wavenumbers (cm^{-1}).

Melting points (mp) are recorded in degrees Celsius (°C) using a Leica Galen III hot-stage microscope apparatus, with the crystallisation solvent reported in parenthesis.

Thin layer chromatography (TLC) was performed on Merck aluminium silica gel 60 F254 precoated plates with visualisation under a UV lamp (λ_{max} = 254 or 365 nm) or by staining with potassium permanganate solution. The retention factors (R_f) are reported with the solvent system used in parenthesis. Reactions were followed by TLC when practical.

For reaction monitoring HPLC samples were analysed using an Agilent Technologies 1200 series HPLC Hypersol ODS, 5 μm column (100 ×4.0 mm). Data was processed using Chem Station for LC systems Rev.B.04.01.SP1.

Flash column chromatography (FCC) as carried out on Merck silica gel 60 (230-400 mesh) and the solvent system used reported in parenthesis. Petrol refers to the fraction of light petroleum ether boiling in the range of 40-60 °C.

The enantiomeric ratio (*er*) was determined by chiral stationary phase HPLC in a Dionex P680 chromatogram with a Dionex UVD170U detector (λ_{max} = 225 nm) using a flow rate of 0.8 mL min^{-1} with a Daicel Chiralpack IC column. The eluent used and retention times (τ_{major} and τ_{minor}) are given in parentheses.

Compound names are generated by CambridgeSoft ChemBioDraw Ultra 12.0.

Reaction temperature was maintained at a constant 15 °C using a glass tempering beaker connected to a flow of water, the vials were submerged in water and irradiated with two EvoluChem™ 450 nm LED spotlights (Figure S1.).

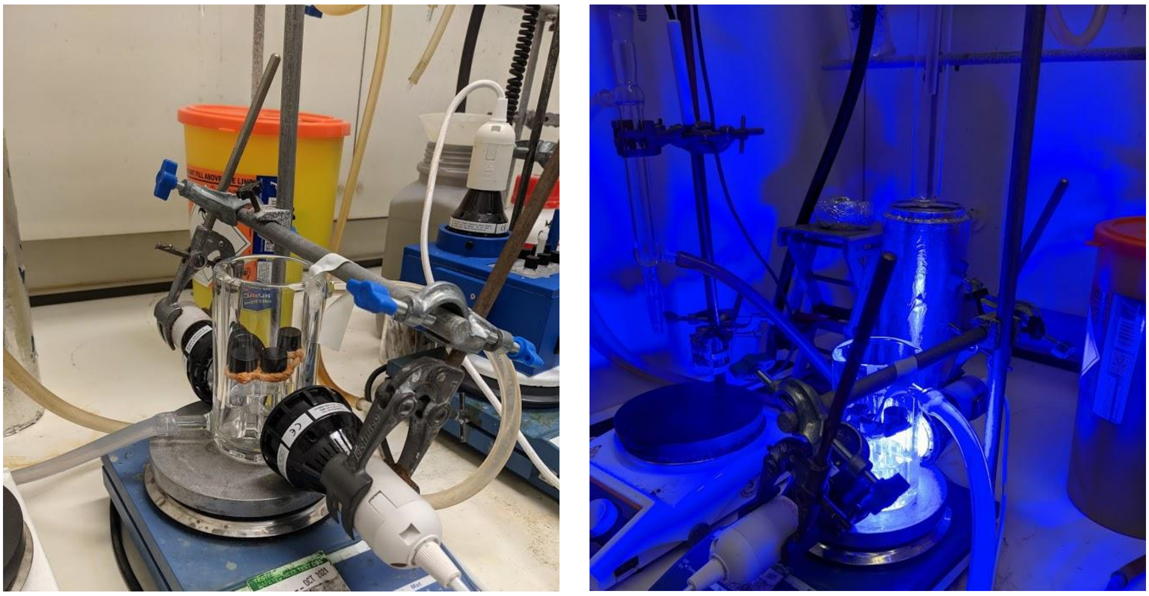

Figure S1. Photochemistry reaction set up for the scope, using a EvoluChem™ 450 nm LED spotlights and a tempering beaker. The image on the left shows the lights off, and on the right lights on, both with the vials submerged in water.

2. Optimisation of the Addition to Neutral Alkenes

2.1. HAD Screen

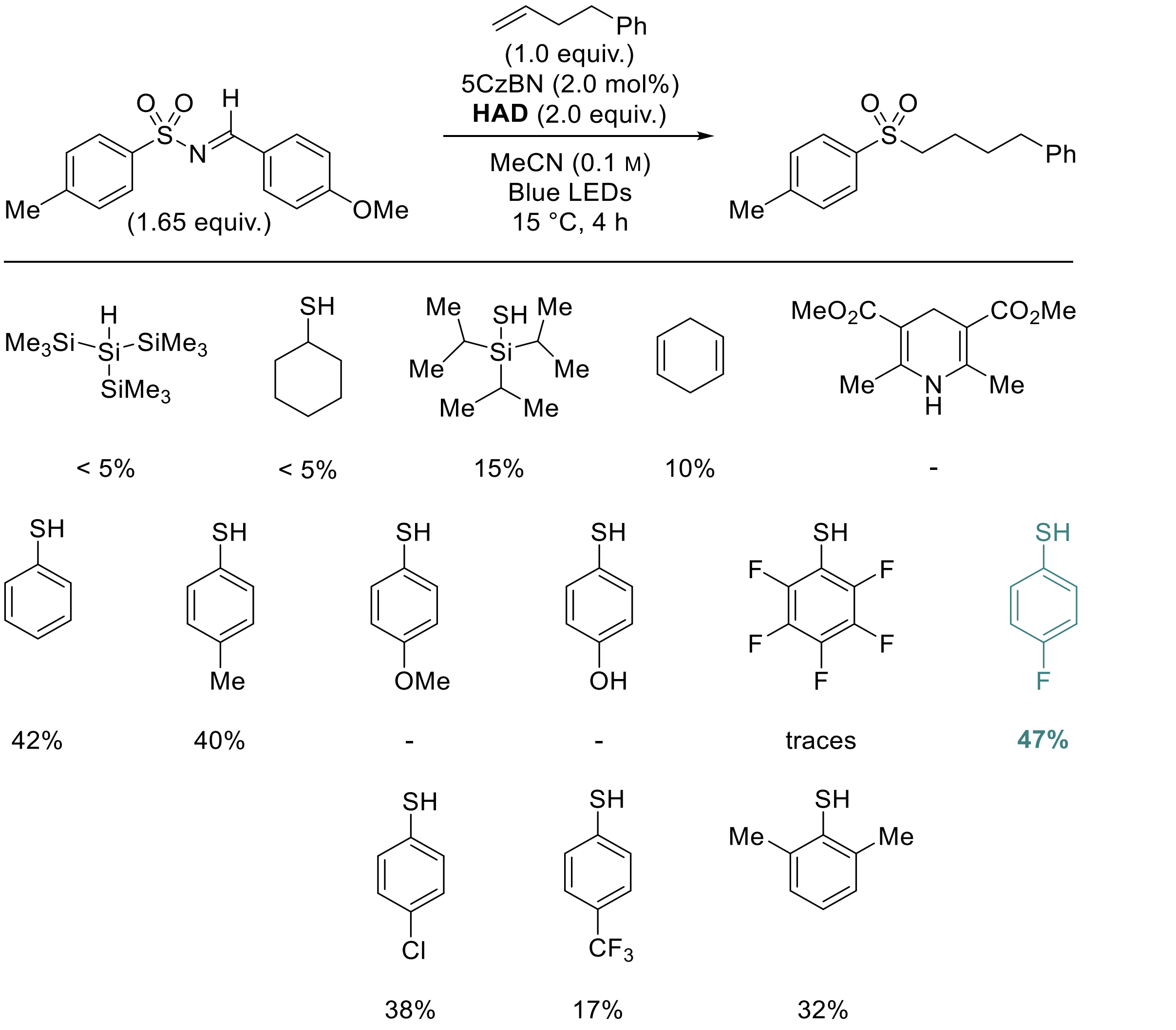

Yields determined by analysis of crude ^{1}H NMR spectrum using 1.0 equiv. of 1,3,5-trimethoxybenzene as internal standard.

A rapid H-atom donor screen showed that electrophilic HAD thiophenols are superior for this transformation. The use of aliphatic thiols, as well as tris(trimethylsilyl)silane and other common HADs such as Hantzsch ester, delivered low yields of the desired product. Among thiophenols, the electronic properties of the aromatic ring appears important, with *p*-fluorothiophenol being optimal (47% yield). Sterically hindered 2,6-dimethylthiophenol has also been tested in our conditions, but did not gave any significant improvement.

2.2. Solvent Screen

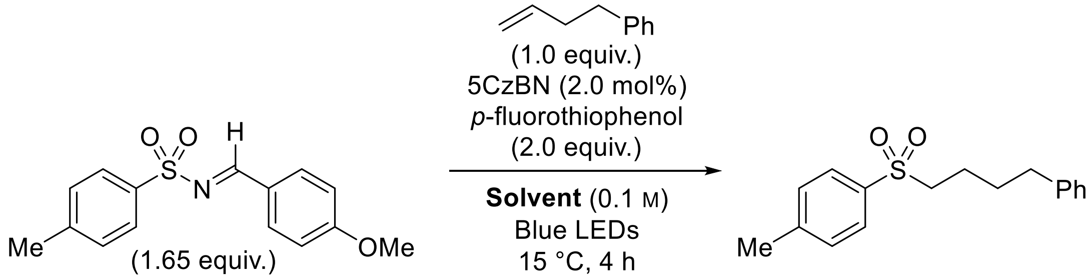

Entry	Solvent	^{1}H NMR Yield	Entry	Solvent	^{1}H NMR Yield
1	MeCN	47%	7	MeOH	0%
2	DMSO	10%	8	EtOAc	41%
3	PhMe	41%	9	$CHCl_3$	48%
4	Acetone	39%	**10**	**DCE**	**60%**
5	DCM	48%	11	DMF	0%
6	THF	42%			

Yields determined by analysis of crude ^{1}H NMR spectrum using 1.0 equiv. of 1,3,5-trimethoxybenzene as internal standard.

A solvent screen revealed again a good solvent tolerance for this transformation. Only very polar aprotic solvents DMSO (entry 2) and DMF (entry 11), as well as protic solvent MeOH (entry 7), are not compatible with the reaction conditions. Giving a substantial increase in yield (60%, entry 10), DCE was selected as optimum solvent.

2.3. Fine-Tuning of the Conditions and Control Reactions

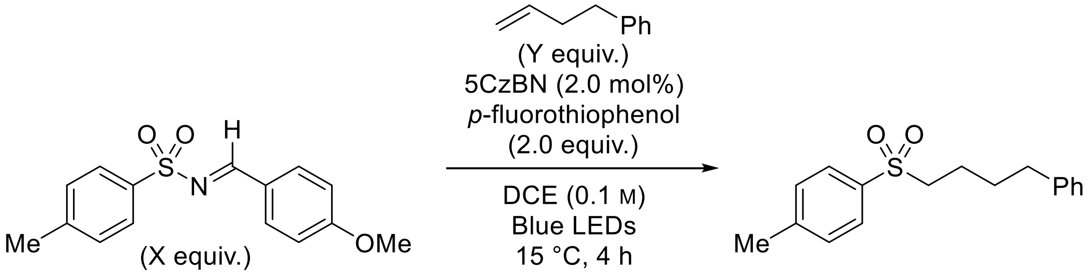

Entry	Modification to the Above Conditions	Stoichiometry (X : Y)	^{1}H NMR Yield
1	None	1.65 : 1.0	60%
2	DCE (0.05 M)	1.65 : 1.0	51%
3	DCE (0.25 M)	1.65 : 1.0	43%
**4**	**5CzBN (1.0 mol%), HAD (2.2 equiv.)**	**1.65 : 1.0**	**68%**
**5**	**5CzBN (1.0 mol%), HAD (2.2 equiv.)**	**1.0 : 2.5**	**72%**
6	No photocatalyst	1.0 : 2.5	0%
7	No light	1.0 : 2.5	0%
8	No light, 80 °C	1.0 : 2.5	0%
9	*p*-TolSO_2Na instead of sulfonyl imine	1.0 : 2.5	0%
10	+ TEMPO (2.5 equiv.)	1.0 : 2.5	0%

Yields determined by analysis of crude ^{1}H NMR spectrum using 1.0 equiv. of 1,3,5-trimethoxybenzene as internal standard.

Finally, fine-tuning of the reaction conditions showed that using 1.0 mol% of the photocatalyst 5CzBN along with 2.2 equiv. of *p*-fluorothiophenol was optimal, delivering 68% yield of the desired product (entry 4). Pleasingly, the stoichiometry could also be reversed (entry 5), giving an even better yield of 72% (entry 5). Control reactions were then performed, and removing the photocatalyst (entry 6) or the irradiation source (entry 7), completely shut down the reaction. Thermal activation also appeared inefficient (entry 8).

3. Experimental Procedures

3.1. Synthesis of Photocatalyst

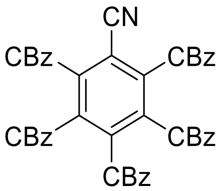

Photocatalyst 5CzBN was synthesised according to the procedure from Speckmeier *et al.*[1] Whilst all other catalysed were obtained from a commercial source.

3.2. Synthesis of Alkenes

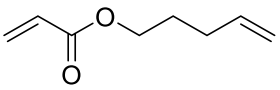

8a

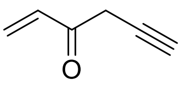

8b

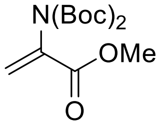

8c

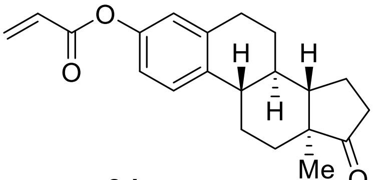

8d

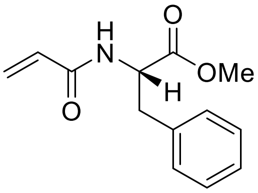

8e

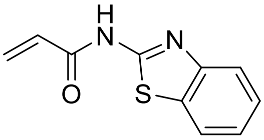

8f

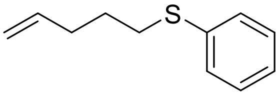

8g

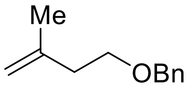

8h

Several alkenes were prepared by literature reported methods including pent-4-en-1-yl acrylate (**8a**),[2] prop-2-yn-1-yl acrylate (**8b**),[3] 2-(bis[*tert*-butoxycarbonyl]amino)acrylate (**8c**),[4] an estrone-derived acrylate (**8d**),[5] a phenylalanine-derived acrylamide (**8e**),[6] *N*-(benzo[*d*]thiazol-2-yl)acrylamide (**8f**),[4] pent-4-en-1-yl phenyl sulfide (**8g**),[7] and benzyl-protected 3-methylbut-3-en-1-ol (**8h**).[8]

3.3. Synthesis of Primary Sulfonamides

General Procedure A:

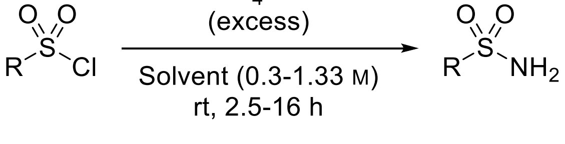

Modified procedure from Mallinger *et al.*[9] Under an atmosphere of air sulfonyl chloride was added to a round bottom flask then dissolved in an appropriate solvent (0.30-1.33 M). To this solution was added ammonium hydroxide in excess by slow dropwise addition. The reaction mixture was stirred at room temperature until

completion as determined by TLC and then the solvent was removed under reduced pressure. The reaction mixture was diluted with EtOAc, and water was added. The two layers were separated, and the aqueous layer was extracted with EtOAc × 3. The combined organic layers were dried over Na_2SO_4, filtered, and the solvent removed under reduced pressure. The crude product was purified by washing with *n*-hexane and Et_2O.

General Procedure B:

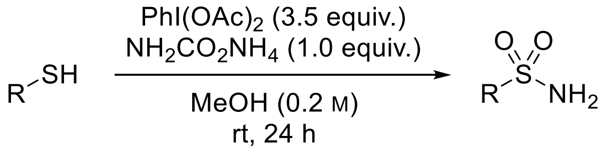

Modified procedure from Tota *et al.*[10] Under an atmosphere of air, thiol (1.0 equiv.) was added to a round bottom flask and dissolved in MeOH (0.2 M). To this solution was added ammonium carbamate (1.0 equiv.) and [(diacetoxy)iodo]benzene (3.5 equiv.) (2.5 mL). The reaction mixture was stirred at room temperature for 24 h and then the solvent was removed under reduced pressure. The crude material was directly purified by flash column chromatography.

Naphthalene-2-sulfonamide (9c)

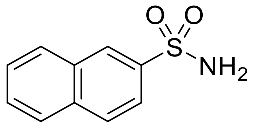

Prepared according to general procedure A, using naphthalene-2-sulfonyl chloride (907 mg, 4.0 mmol), THF (13.0 mL) and ammonium hydroxide (8.7 mL), with stirring for 2.5 h, affording the title compound (647 mg, 78%) as a pale pink solid. **mp** (*n*-hexane/Et_2O) 207 – 209 °C; ^{1}H NMR (400 MHz, **acetone-*d*$_6$**) δ_H 8.47 (d, *J* = 2.0 Hz, 1H, Ar*H*), 8.14 – 8.06 (m, 2H, Ar*H*), 8.06 – 7.98 (m, 1H, Ar*H*), 7.95 (dd, *J* = 8.5, 2.0 Hz, 1H, Ar*H*), 7.73 – 7.61 (m, 2H, Ar*H*), 6.66 (br. s, 2H, SO_2NH_2); ^{13}C NMR (101 MHz, **acetone-*d*$_6$**) δ_C 142.2, 135.5, 133.2, 130.0 (2C), 129.3, 128.8, 128.3, 127.3, 123.2; LRMS (ESI, *m/z*) $[M-H]^-$ 206.0, $[M+Na]^+$ 230.0. The spectroscopic data is in agreement with literature.[11]

2,4,6-Trimethylbenzenesulfonamide (9d)

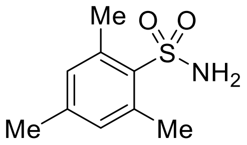

Prepared according to general procedure A, using 2,4,6-trimethylbenzenesulfonyl chloride (875 mg, 4.0 mmol), THF (13.0 mL) and ammonium hydroxide (8.7 mL), with stirring for 2.5 h, affording the title compound (717 mg, 90%) as a colourless solid. **mp** (*n*-hexane/Et_2O) 129 – 131 °C; **^{1}H NMR** (400 MHz, acetone-d_6) δ_H 7.00 (s, 1H, Ar*H*), 6.43 (br. s, 2H, SO_2NH_2), 2.62 (s, 6H, ArCH_3), 2.28 (s, 3H, ArCH_3); **^{13}C NMR** (101 MHz, acetone-d_6) δ_C 142.1, 139.1, 138.7, 132.5, 23.1, 20.9; **LRMS** (ESI, *m/z*) $[M-H]^-$ 198.1, $[M+Na]^+$ 222.0. The spectroscopic data is in agreement with literature.[12]

4-(Trifluoromethyl)benzenesulfonamide (9f)

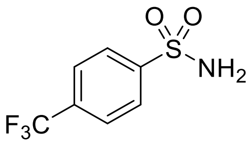

Prepared according to general procedure A, using 4-(trifluoromethyl)benzenesulfonyl chloride (978 mg, 4.0 mmol), THF (13.0 mL) and ammonium hydroxide (8.7 mL), with stirring for 2.5 h, affording the title compound (720 mg, 80%) as a colourless solid. **mp** (*n*-hexane/Et_2O) 162 – 164 °C; **^{1}H NMR** (400 MHz, acetone-d_6) δ_H 8.14 (d, *J* = 8.0 Hz, 2H, Ar*H*), 7.95 (d, *J* = 8.0 Hz, 2H, Ar*H*), 6.87 (br. s, 2H, SO_2NH_2); **^{13}C NMR** (101 MHz, acetone-d_6) δ_C 148.8, 133.7 (q, *J* = 32.5 Hz), 127.8, 127.0 (q, *J* = 4.0 Hz), 124.6 (q, *J* = 272.0 Hz); **^{19}F NMR** (377 MHz, acetone-d_6) δ_F -58.31; **LRMS** (ESI, *m/z*) $[M-H]^-$ 224.0. The spectroscopic data is in agreement with literature.[12]

4-Acetylbenzenesulfonamide (9h)

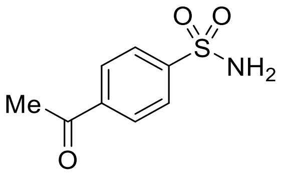

Prepared according to general procedure A, using 4-acetylbenzenesulfonyl chloride (875 mg, 4.0 mmol), THF (13.0 mL) and ammonium hydroxide (8.7 mL), with stirring for 2.5 h, affording the title compound (494 mg, 62%) as a pale-yellow solid. **mp** (*n*-hexane/Et_2O) 166 – 168 °C; **^{1}H NMR** (400 MHz, acetone-d_6) δ_H 8.14 (d, *J* = 8.5 Hz, 2H, Ar*H*), 8.02 (d, *J* = 8.5 Hz, 2H, Ar*H*), 6.83 (br. s, 2H, SO_2NH_2), 2.64 (s, 3H, COCH_3); **^{13}C NMR**

(101 MHz, acetone-d_6) δ_C 197.3, 148.5, 140.4, 129.5, 127.0, 26.9; **LRMS** (ESI, *m/z*) $[M-H]^-$ 198.0, $[M+Na]^+$ 222.0. The spectroscopic data is in agreement with literature.[12]

Methyl 3-sulfamoylbenzoate (9i)

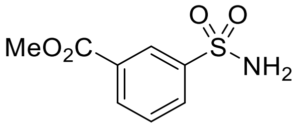

Prepared according to general procedure A, using methyl 3-(chlorosulfonyl)benzoate (409 mg, 1.74 mmol), THF (6.0 mL) and ammonium hydroxide (3.8 mL), with stirring for 2.5 h, affording the title compound (232 mg, 62%) as a colourless solid. **mp** (*n*-hexane/Et_2O) 119 – 121 °C; ^{1}**H NMR** (400 MHz, acetone-d_6) δ_H 8.49 (app. td, *J* = 2.0, 0.5 Hz, 1H, Ar*H*), 8.20 (ddd, *J* = 8.0, 1.5, 1.0 Hz, 1H, Ar*H*), 8.14 (ddd, *J* = 8.0, 2.0, 1.0 Hz, 1H, Ar*H*), 7.73 (app. td, *J* = 8.0, 0.5 Hz, 1H, Ar*H*), 6.77 (br. s, 2H, SO_2NH_2), 3.94 (s, 3H, CO_2CH_3); ^{13}**C NMR** (101 MHz, acetone-d_6) δ_C 166.1, 145.7, 133.3, 131.9, 131.1, 130.4, 127.7, 52.8; **LRMS** (ESI, *m/z*) $[M-H]^-$ 214.0, $[M+Na]^+$ 238.0. The spectroscopic data is in agreement with literature.[12]

3-Bromobenzenesulfonamide (9k)

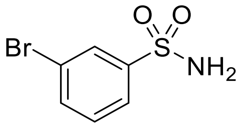

Prepared according to general procedure B, using 3-bromobenzenethiol (0.41 mL, 4.0 mmol), ammonium carbamate (384 mg, 4.0 mmol) and [(diacetoxy)iodo]benzene (4.51 g, 14.0 mmol). Purification by flash column chromatography (*n*-hexane:EtOAc, 50:50 to 10:90), afforded the title compound (547 mg, 58%) as a colourless solid. **mp** (*n*-hexane/EtOAc) 140 – 142 °C; ^{1}**H NMR** (400 MHz, MeOD-d_4) δ_H 8.06 (t, *J* = 2.0 Hz, 1H, Ar*H*), 7.88 (ddd, *J* = 8.0, 2.0, 1.0 Hz, 1H, Ar*H*), 7.76 (ddd, *J* = 8.0, 2.0, 1.0 Hz, 1H, Ar*H*), 7.48 (t, *J* = 8.0 Hz, 1H, Ar*H*), (note: SO_2NH_2 peak not observed); ^{13}**C NMR** (101 MHz, MeOD-d_4) δ_C 147.0, 136.1, 131.9, 130.0, 125.9, 123.5; **LRMS** (ESI, *m/z*) $[^{79}BrM-H]^-$ 233.9, $[^{81}BrM-H]^-$ 235.9; **HRMS** (ESI, *m/z*) calculated for $(C_6H_5{}^{79}BrNO_2S)^-$ 233.9230 $[^{79}BrM-H]^-$, found 233.9224, calculated for $(C_6H_5{}^{81}BrNO_2S)^-$ 235.9209 $[^{81}BrM-H]^-$, found 235.9201; **IR** (thin film, ν_{max}/cm^{-1}) 3330, 3243, 1571, 1463, 1418, 1326, 1297, 1162, 1069, 995, 879, 848, 786, 675, 656; $\mathbf{R_f}$ 0.65 (*n*-hexane:EtOAc, 50:50).

2-Bromobenzenesulfonamide (9l)

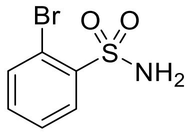

Prepared according to general procedure A, using 2-bromobenzenesulfonyl chloride (1.02 g, 4.0 mmol), THF (13.0 mL) and ammonium hydroxide (8.7 mL), with stirring for 2.5 h, affording the title compound (708 mg, 75%) as a colourless solid. **mp** (*n*-hexane/EtOAc) 184 – 186 °C; **^{1}H NMR** (400 MHz, acetone-d_6) δ_H 8.12 (dd, *J* = 7.5, 2.0 Hz, 1H, Ar*H*), 7.83 (dd, *J* = 7.5, 1.5 Hz, 1H, Ar*H*), 7.57 (app. td, *J* = 7.5, 1.5 Hz, 1H, Ar*H*), 7.52 (app. td, *J* = 7.5, 2.0 Hz, 1H, Ar*H*), 6.73 (br. s, 2H, SO_2NH_2); **^{13}C NMR** (101 MHz, acetone-d_6) δ_C 143.7, 135.9, 134.2, 130.5, 128.8, 120.2; **LRMS** (ESI, *m/z*) [^{79}BrM-H]$^-$ 233.9, [^{81}BrM-H]$^-$ 235.9. The spectroscopic data is in agreement with literature.[10]

2,4-Dimethoxybenzenesulfonamide (9n)

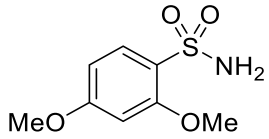

Prepared according to general procedure A, using 2,4-dimethoxybenzenesulfonyl chloride (947 mg, 4.0 mmol), THF (13.0 mL) and ammonium hydroxide (8.7 mL), with stirring for 2.5 h, affording the title compound (337 mg, 39%) as a pink solid. **mp** (*n*-hexane/Et_2O) 148 – 150 °C; **^{1}H NMR** (400 MHz, acetone-d_6) δ_H 7.72 (d, *J* = 8.5 Hz, 1H, Ar*H*), 6.71 (d, *J* = 2.5 Hz, 1H, Ar*H*), 6.60 (dd, *J* = 8.5, 2.5 Hz, 1H, Ar*H*), 6.14 (br. s, 2H, SO_2NH_2), 3.97 (s, 3H, OCH_3), 3.88 (s, 3H, OCH_3); **^{13}C NMR** (101 MHz, acetone-d_6) δ_C 165.2, 158.7, 130.3, 125.1, 105.2, 99.9, 56.6, 56.1; **LRMS** (ESI, *m/z*) [M+Na]$^+$ 240.0; **HRMS** (ESI, *m/z*) calculated for $(C_8H_{12}NO_4S)^+$ 218.0482 [M+H]$^+$, found 218.0484; **IR** (thin film, ν_{max}/cm^{-1}) 3382, 3271, 1601, 1574, 1490, 1464, 1434, 1413, 1299, 1212, 1163, 1079, 1018, 920, 895, 866, 834, 808, 720; **R_f** 0.13 (*n*-hexane:EtOAc, 50:50).

Propane-1-sulfonamide (9p)

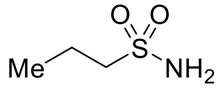

Prepared according to general procedure A, using propanesulfonyl chloride (1.01 g, 10.0 mmol), MeOH (7.5 mL) and ammonium hydroxide (37.0 mL), with stirring for 16 h, affording the title compound (160 mg, 13%) as a colourless solid. **mp** (*n*-hexane/EtOAc) 48 – 50 °C; **^{1}H NMR** (400 MHz, DMSO-d_6) δ_H 6.72 (br. s, 2H, SO_2NH_2), 2.98 – 2.88 (m, 2H, $CH_3CH_2CH_2S$), 1.80 – 1.61 (m, 2H, $CH_3CH_2CH_2$), 0.97 (t, *J* = 7.5 Hz, 3H,

CH_3CH_2); **^{13}C NMR** (101 MHz, DMSO-d_6) δ_C 56.2, 17.2, 12.8; **LRMS** (ESI, *m/z*) [M-H]$^-$ 122.1; **HRMS** (ESI, *m/z*) calculated for $(C_3H_8NO_2S)^-$ 122.0281 [M-H]$^-$, found 122.0275; **IR** (thin film, ν_{max}/cm^{-1}) 3340, 3254, 2965, 1664, 1540, 1460, 1308, 1288, 1135, 1039, 907, 885, 785, 692; **R$_f$** 0.27 (*n*-hexane:EtOAc, 50:50).

Cyclohexanesulfonamide (9q)

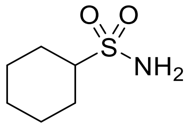

Prepared according to general procedure B, using cyclohexanethiol (0.61 mL, 5.0 mmol), ammonium carbamate (480 mg, 5.0 mmol) and [(diacetoxy)iodo]benzene (5.64 g, 17.5 mmol). Purification by flash column chromatography (*n*-hexane:EtOAc, 50:50 to 10:90), afforded the title compound (654 mg, 80%) as a white waxy solid. **mp** (*n*-hexane/EtOAc) 82 – 84 °C; **^{1}H NMR** (400 MHz, $CDCl_3$) δ_H 4.72 (br. s, 2H, SO_2NH_2), 2.91 (tt, *J* = 12.0, 3.5 Hz, 1H, Cy*H*), 2.23 (app. dq, *J* = 12.0, 1.5 Hz, 2H, Cy*H*), 1.90 (app. dq, *J* = 13.0, 3.5 Hz, 2H, Cy*H*), 1.72 (app. dq, *J* = 12.0, 1.5 Hz, 1H, Cy*H*), 1.49 (app. qd, *J* = 12.0, 3.5 Hz, 2H, Cy*H*), 1.30 (app. qt, *J* = 12.0, 3.0 Hz, 1H, Cy*H*), 1.19 (app. qt, *J* = 13.0, 3.0 Hz, 1H, Cy*H*); **^{13}C NMR** (101 MHz, $CDCl_3$) δ_C 62.9, 26.7, 25.2 (2C); **HRMS** (ESI, *m/z*) calculated for $(C_6H_{12}NO_2S)^-$ 162.0594 [M-H]$^-$, found 162.0585. The spectroscopic data is in agreement with literature.[10]

Cyclopropanesulfonamide (9s)

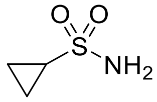

Prepared according to general procedure A, using cyclopropylsulfonyl chloride (1.01 mL, 10.0 mmol), MeOH (7.5 mL) and ammonium hydroxide (37.0 mL), with stirring for 16 h, affording the title compound (516 mg, 43%) as a colourless solid. **mp** (*n*-hexane/Et_2O) 96 – 98 °C; **^{1}H NMR** (400 MHz, acetone-d_6) δ_H 6.09 (br. s, 2H, SO_2NH_2), 2.57 (tt, *J* = 8.0, 5.0 Hz, 1H, $(CH_2)_2CH$), 1.06 – 0.87 (m, 4H, $(CH_2)_2CH$); **^{13}C NMR** (101 MHz, acetone-d_6) δ_C 32.8, 5.6; **LRMS** (ESI, *m/z*) [M-H]$^-$ 120.0. The spectroscopic data is in agreement with literature.[13]

Pyridine-3-sulfonamide (9u)

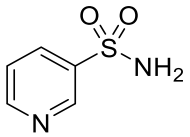

Prepared according to general procedure A, using pyridine-3-sulfonyl chloride (763 mg, 4.0 mmol), THF (13.0 mL) and ammonium hydroxide (8.7 mL), with stirring for 2.5 h, affording the title compound (410 mg, 65%) as a colourless solid. **mp** (*n*-hexane/Et_2O) 94 – 96 °C; **^{1}H NMR** (400 MHz, acetone-d_6) δ_H 9.05 (dd, *J* = 2.5, 1.0 Hz, 1H, Py*H*), 8.78 (dd, *J* = 5.0, 1.5 Hz, 1H, Py*H*), 8.24 (ddd, *J* = 8.0, 2.5, 1.5 Hz, 1H, Py*H*), 7.59 (ddd, *J* = 8.0, 5.0, 1.0 Hz, 1H, Py*H*), 6.91 (br. s, 2H, SO_2NH_2); **^{13}C NMR** (101 MHz, acetone-d_6) δ_C 153.3, 147.7, 141.1, 134.5, 124.5; **LRMS** (ESI, *m/z*) $[M-H]^-$ 157.0, $[M+H]^+$ 159.0. The spectroscopic data is in agreement with literature.[14]

4-Phenylpiperidine-1-sulfonamide (9w)

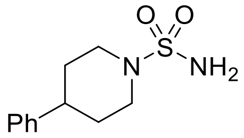

Prepared according to the modified procedure from Jennings *et al.*[15] Under an atmosphere of air, 4-phenylpiperidine amine (645 mg, 4.0 mmol, 1.0 equiv.) and sulfamide (422 mg, 4.4 mmol, 1.1 equiv.) were added to a round bottom flask equipped with a reflux condenser and dissolved in 1,4-dioxane (30 mL). The resulting suspension was refluxed at 100 °C for 2 h and then cooled to room temperature, and the solvent removed under reduced pressure. Purification by flash column chromatography (Petrol:EtOAc, 20:80) afforded the title compound (359 mg, 75%) as a colourless solid. **mp** (*n*-hexane/Et_2O) 160 – 162 °C; **^{1}H NMR** (400 MHz, acetone-d_6) δ_H 7.36 – 7.25 (m, 4H, Ph*H*), 7.24 – 7.16 (m, 1H, Ph*H*), 3.74 (app. ddt, *J* = 11.5, 4.0, 2.0 Hz, 2H, $NCH_AH_BCH_2$) 2.75 (app. td, *J* = 12.0, 2.0 Hz, 2H, $NCH_AH_BCH_2$), 2.64 (tt, *J* = 12.0, 4.0 Hz, 1H, $PhCH(CH_2)_2$), 1.96 – 1.86 (m, 2H, $NCH_2CH_AH_B$), 1.78 (app. qd, *J* = 13.0, 4.0 Hz, 2H, $NCH_2CH_AH_B$), (note: SO_2NH_2 peak not observed); **^{13}C NMR** (101 MHz, acetone-d_6) δ_C 146.4, 129.1, 127.4, 126.9, 47.5, 42.4, 33.1; **LRMS** (ESI, *m/z*) $[M-H]^-$ 239.0; **HRMS** (ESI, *m/z*) calculated for $(C_{11}H_{15}N_2O_2S)^-$ 239.0860 $[M-H]^-$, found 239.0859; **IR** (thin film, ν_{max}/cm^{-1}) 3370, 3274, 2160, 2029, 1543, 1468, 1358, 1248, 1166, 1094, 934, 883, 765, 722, 701, 641; **R_f** 0.47 (*n*-hexane:EtOAc, 50:50).

Methyl 2,4-dichloro-5-sulfamoylbenzoate (9x)

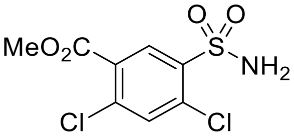

Prepared according to the modified procedure from Matulis *et al.*[16] Under an atmosphere of air 2,4-dichloro-5-sulfamoylbenzoic acid (1.35 g, 5.0 mmol) were added to a round bottom flask equipped with a reflux condenser and dissolved in MeOH (50.0 mL). The solution was refluxed at 65 °C with concentrated H_2SO_4 (0.5 mL) for 16 h. The reaction mixture was cooled to room temperature and then concentrated under reduced pressure. Purification by recrystallisation from MeOH, affording the title compound (1.01 g, 71%) as a colourless solid. **mp** (MeOH) 186 – 188 °C; **^{1}H NMR** (400 MHz, DMSO-d_6) δ_H 8.39 (s, 1H, Ar*H*), 8.01 (s, 1H, Ar*H*), 7.85 (br. s, 2H, SO_2NH_2), 3.90 (s, 3H, CO_2CH_3); **^{13}C NMR** (101 MHz, DMSO-d_6) δ_C 163.7, 140.1, 136.3, 134.6, 133.6, 131.3, 128.4, 53.1; **LRMS** (ESI, *m/z*) $[M\text{-}H]^-$ 281.9; **HRMS** (ESI, *m/z*) calculated for $(C_8H_6{}^{35}Cl_2NO_4S)^-$ 281.9400 $[M\text{-}H]^-$, found 281.9399; **IR** (thin film, ν_{max}/cm^{-1}) 3350, 3247, 1721, 1562, 1430, 1358, 1293, 1257, 1171, 1074, 975, 920, 779, 688, 610; **R_f** 0.38 (*n*-hexane:EtOAc, 50:50).

***N*-(4-sulfamoylphenyl)acetamide (9z)**

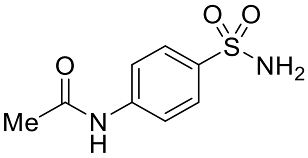

Prepared according to general procedure A, using 4-acetamidobenzenesulfonyl chloride (1.87 mg, 8.0 mmol), THF (26.0 mL) and ammonium hydroxide (17.4 mL), with stirring for 2.5 h, affording the title compound (536 mg, 31%) as a colourless solid. **mp** (*n*-hexane/Et_2O) 206 – 208 °C; **^{1}H NMR** (400 MHz, DMSO-d_6) δ_H 10.26 (br. s, 1H, CON*H*), 7.75 (d, *J* = 9.5 Hz, 2H, Ar*H*), 7.72 (d, *J* = 9.5 Hz, 1H, Ar*H*), 7.22 (br. s, 2H, SO_2NH_2), 2.08 (s, 3H, $NHCOCH_3$); **^{13}C NMR** (101 MHz, DMSO-d_6) δ_C 168.9, 142.2, 138.1, 126.7, 118.4, 24.1; **LRMS** (ESI, *m/z*) $[M\text{-}H]^-$ 213.0, $[M+H]^+$ 215.0, $[M+Na]^+$ 237.0. The spectroscopic data is in agreement with literature.[17]

Benzyl 4-chloro-2-((furan-2-ylmethyl)amino)-5-sulfamoylbenzoate (9ab)

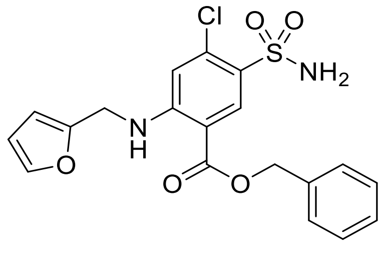

Prepared according to the modified procedure from Pérez-Palau *et al.*[18] Under air atmosphere, Furosemide (2.00 g, 6.00 mmol, 1.00 equiv.) was added to a round bottom flask equipped with a reflux condenser and dissolved in DMF (40 mL). To this solution was added benzyl chloride (0.65 mL, 5.72 mmol, 0.95 equiv.) and Et_3N (0.88 mL, 6.32 mmol, 1.05 equiv.). The mixture was stirred at 80 °C for 12 h and allowed to cool to room temperature. The mixture was diluted with CH_2Cl_2 (80 mL) and washed with H_2O (80 mL), sat. aq. NH_4Cl (80 mL), then dried over Na_2SO_4, filtered, and concentrated under reduced pressure. Purification by flash column chromatography (*n*-hexane:EtOAc, 20:90 to 40:60) afforded the title compound (2.08 g, 86%) as an off-white solid. **mp** (*n*-hexane/EtOAc) 90 – 92 °C; **^{1}H NMR** (400 MHz, DMSO-d_6) δ_H 8.55 – 8.50 (m, 2H, Ar*H*, CH_2N*H*Ar), 7.62 (d, *J* = 2.0 Hz, 1H, HetAr*H*), 7.47 – 7.33 (m, 7H, SO_2NH_2, Ph*H*), 7.14 (s, 1H, Ar*H*), 6.43 (dd, *J* = 3.5, 2.0 Hz, 1H, HetAr*H*), 6.40 (d, *J* = 3.5 Hz, 1H, HetAr*H*), 5.37 (s, 2H, CO_2CH_2Ph), 4.62 (d, *J* = 6.0 Hz, 2H, HetArCH_2NH); **^{13}C NMR** (101 MHz, DMSO-d_6) δ_C 166.3, 152.3, 151.2, 142.8, 136.9, 135.9, 133.0, 128.6, 128.3, 128.1, 127.2, 114.0, 110.6, 107.8, 107.2, 66.3, 39.3; **LRMS** (ESI, *m/z*) $[M+Na]^+$ 443.0. The spectroscopic data is in agreement with literature.[18]

3.4. Synthesis of *N*-sulfonylimines

General Procedure C:

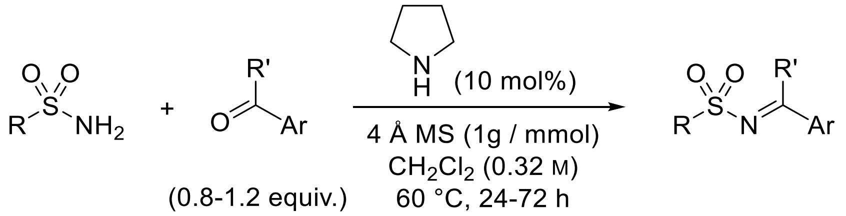

Modified procedure from Morales *et al.*[19] The amino compound (1.0 equiv.) and 4 Å molecular sieves (1g / mmol) were added to an appropriate reaction vessel and CH_2Cl_2 (0.32 M) was added. The corresponding aldehyde (0.8-1.2 equiv.) and pyrrolidine (10 mol%) were added and the resulting suspension was refluxed at 60 °C, either with a reflux condenser equipped or in a sealed vial. The reaction was stirred for 24-72 h and cooled to room temperature, then filtered through a short pad of Celite®, whilst washing with CH_2Cl_2. The solvent was removed from the filtrate under reduced pressure and the crude product was purified by crystallisation in Et_2O and washing with *n*-hexane.

N-(4-Methoxybenzylidene)-4-methylbenzenesulfonamide (1a)

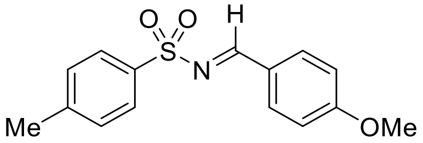

Prepared according to general procedure C, using 4-methylbenzenesulfonamide (5.14 g, 30.0 mmol, 1.0 equiv.), 4-methoxybenzaldehyde (3.65 mL, 36.0 mmol, 1.2 equiv.) and pyrrolidine (0.25 mL, 3.0 mmol, 10 mol%) with stirring for 24 h, affording the title compound (6.85 g, 79%) as a colourless solid. **mp** (*n*-hexane/Et_2O) 116 – 118 °C; **^{1}H NMR** (400 MHz, $CDCl_3$) δ_H 8.93 (s, 1H, N*H*C), 7.91 – 7.82 (m, 4H, *p*-OMeAr*H*, *p*-MeAr*H*), 7.32 (d, *J* = 8.0 Hz, 2H, *p*-MeAr*H*), 6.95 (d, *J* = 9.0 Hz, 2H, *p*-OMeAr*H*), 3.87 (s, 3H, OCH_3), 2.41 (s, 3H, $ArCH_3$); **^{13}C NMR** (101 MHz, $CDCl_3$) δ_C 169.3, 165.4, 144.4, 135.8, 133.8, 129.8, 128.0, 125.3, 114.8, 55.8, 21.7; **LRMS** (ESI, *m/z*) $[M+H]^+$ 290.0, $[M+Na]^+$ 312.0. The spectroscopic data is in agreement with literature.[20]

N-((4-Methoxyphenyl)methylene-d)-4-methylbenzenesulfonamide (1a-d_1)

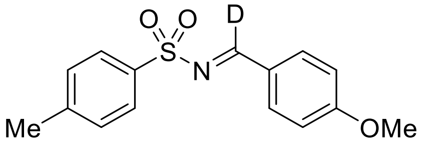

Prepared according to general procedure C, using 4-methylbenzenesulfonamide (171 mg, 1.0 mmol, 1.0 equiv.), *p*-anisaldehyde-α-d_1[21] (0.15 mL, 1.2 mmol, 1.2 equiv.) and pyrrolidine (9 μL, 0.1 mmol, 10 mol%) with stirring for 24 h, affording the title compound (191 mg, 66%) as a yellow solid. **mp** (*n*-hexane) 111 – 113 °C; **^{1}H NMR** (400 MHz, $CDCl_3$) δ_H 7.91 – 7.82 (m, 4H, *p*-OMeAr*H*, *p*-MeAr*H*), 7.33 (d, *J* = 8.0 Hz, 2H, *p*-MeAr*H*), 6.96 (d, *J* = 9.0 Hz, 2H, *p*-OMeAr*H*), 3.88 (s, 3H, OCH_3), 2.42 (s, 3H, $ArCH_3$); **^{13}C NMR** (101 MHz, $CDCl_3$) δ_C 169.1 (m), 165.4, 144.4, 135.8, 133.8, 129.9, 128.0, 125.3, 114.8, 55.8, 21.8; **LRMS** (ESI, *m/z*) $[M+H]^+$ 291.0. The spectroscopic data is in agreement with literature.[22]

N-Benzylidene-4-methylbenzenesulfonamide (1b)

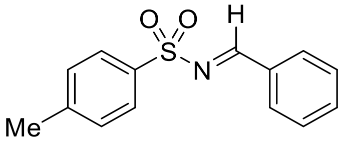

Prepared according to general procedure C, using 4-methylbenzenesulfonamide (1.71 g, 10.0 mmol, 1.0 equiv.), benzaldehyde (1.22 mL, 12.0 mmol, 1.2 equiv.) and pyrrolidine (83 μL, 1.0 mmol, 10 mol%) with stirring for 24 h, affording the title compound (1.74 g, 67%) as a colourless solid. **mp** (*n*-hexane/Et_2O) 99 – 101 °C; **^{1}H NMR** (400 MHz, $CDCl_3$) δ_H 9.03 (s, 1H, N*H*C), 7.97 – 7.83 (m, 4H, *p*-MeAr*H*, Ph*H*), 7.65 – 7.55

(m, 1H, PhH), 7.52 – 7.43 (m, 2H, PhH), 7.34 (d, J = 8.0 Hz, 2H, p-MeArH), 2.43 (s, 3H, ArCH_3); **^{13}C NMR** (101 MHz, $CDCl_3$) δ_C 170.2, 144.7, 135.2, 135.0, 132.5, 131.4, 129.9, 129.2, 128.2, 21.7; **LRMS** (ESI, *m/z*) $[M+H]^+$ 260.0, $[M+Na]^+$ 282.0. The spectroscopic data is in agreement with literature.[20]

4-Methyl-*N*-(4-(trifluoromethyl)benzylidene)benzenesulfonamide (1c)

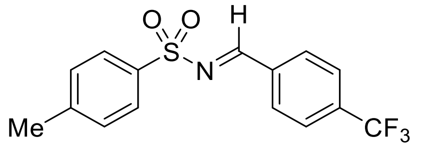

Prepared according to general procedure C, using 4-methylbenzenesulfonamide (171 mg, 1.0 mmol, 1.0 equiv.), 4-(trifluoromethyl)benzaldehyde (0.16 mL, 1.2 mmol, 1.2 equiv.) and pyrrolidine (9 µL, 0.1 mmol, 10 mol%) with stirring for 24 h, affording the title compound (232 mg, 71%) as a colourless solid. **mp** (*n*-hexane/Et_2O) 146 – 148 °C; **^{1}H NMR** (400 MHz, $CDCl_3$) δ_H 9.07 (s, 1H, NHC), 8.03 (d, J = 8.0 Hz, 2H, p-CF_3ArH), 7.89 (d, J = 8.0 Hz, 2H, p-MeArH), 7.72 (d, J = 8.0 Hz, 2H, p-CF_3ArH), 7.35 (d, J = 8.0 Hz, 2H, p-MeArH), 2.42 (s, 3H, ArCH_3); **^{13}C NMR** (101 MHz, $CDCl_3$) δ_C 168.5, 145.1, 135.4, 135.7 (q, J = 33.0 Hz), 134.5, 131.4, 130.0, 128.3, 126.1 (q, J = 4.0 Hz), 123.4 (q, J = 273.0 Hz), 21.7; **^{19}F NMR** (377 MHz, $CDCl_3$) δ_F -63.28; **LRMS** (ESI, *m/z*) $[M+Na]^+$ 350.0. The spectroscopic data is in agreement with literature.[20]

4-Methyl-*N*-(4-nitrobenzylidene)benzenesulfonamide (1d)

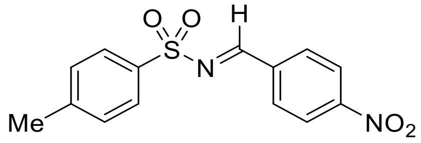

Prepared according to general procedure C, using 4-methylbenzenesulfonamide (188 mg, 1.1 mmol, 1.1 equiv.), 4-nitrobenzaldehyde (94 mg, 1.0 mmol, 1.0 equiv.) and pyrrolidine (9 µL, 0.1 mmol, 10 mol%) with stirring for 24 h, affording the title compound (193 mg, 63%) as a colourless solid. **mp** (*n*-hexane/Et_2O) 185 – 187 °C; **^{1}H NMR** (400 MHz, $CDCl_3$) δ_H 9.10 (s, 1H, NHC), 8.32 (d, J = 9.0 Hz, 2H, p-NO_2ArH), 8.11 (d, J = 9.0 Hz, 2H, p-NO_2ArH), 7.90 (d, J = 8.0 Hz, 2H, p-MeArH), 7.38 (d, J = 8.0 Hz, 2H, p-MeArH), 2.46 (s, 3H, ArCH_3); **^{13}C NMR** (101 MHz, $CDCl_3$) δ_C 167.5, 151.3, 145.5, 137.6, 134.3, 132.0, 130.2, 128.5, 124.3, 21.9; **LRMS** (ESI, *m/z*) $[M+Na]^+$ 327.0. The spectroscopic data is in agreement with literature.[20]

4-Methyl-*N*-(2,4,6-trimethylbenzylidene)benzenesulfonamide (1e)

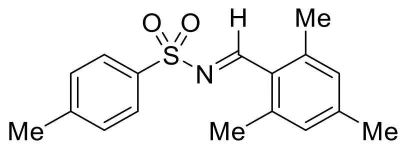

Prepared according to general procedure C, using 4-methylbenzenesulfonamide (171 mg, 1.0 mmol, 1.0 equiv.), 2,4,6-trimethylbenzaldehyde (0.18 mL, 1.2 mmol, 1.2 equiv.) and pyrrolidine (9 μL, 0.1 mmol, 10 mol%) with stirring for 24 h, affording the title compound (216 mg, 72%) as a colourless solid. **mp** (*n*-hexane/Et_2O) 84 – 86 °C; **^{1}H NMR** (400 MHz, $CDCl_3$) δ_H 9.50 (s, 1H, N*H*C), 7.88 (d, *J* = 8.5 Hz, 2H, *p*-MeAr*H*), 7.33 (d, *J* = 8.5 Hz, 2H, *p*-MeAr*H*), 6.92 (s, 2H, Ar*H*), 2.53 (s, 6H, ArC*H*$_3$), 2.44 (s, 3H, CC*H*$_3$), 2.31 (s, 3H, ArC*H*$_3$); **^{13}C NMR** (101 MHz, $CDCl_3$) δ_C 169.2, 144.7, 144.3, 143.1, 130.8, 129.8, 128.0, 126.6, 126.3, 21.9, 21.8, 21.7; **LRMS** (ESI, *m/z*) $[M+H]^+$ 302.0, $[M+Na]^+$ 324.0; **HRMS** (ESI, *m/z*) calculated for $(C_{17}H_{20}NO_2S)^+$ 302.1209 $[M+H]^+$, found 302.1209; **IR** ($CHCl_3$, ν_{max}/cm^{-1}) 1589, 1555, 1304, 1088, 1035, 831, 815, 772, 724, 705, 635; **R$_f$** 0.75 (*n*-hexane:EtOAc, 60:40).

4-methyl-*N*-(2,4,6-trimethoxybenzylidene)benzenesulfonamide (1f)

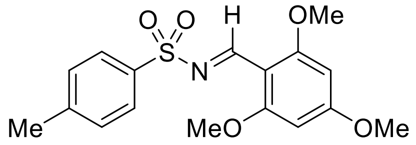

Prepared according to general procedure C, using 4-methylbenzenesulfonamide (171 mg, 1.0 mmol, 1.0 equiv.), 2,4,6-trimethoxybenzaldehyde (235 mg, 1.2 mmol, 1.2 equiv.) and pyrrolidine (9 μL, 0.1 mmol, 0.1 equiv.) with stirring for 24 h, affording the title compound (246 mg, 71%) as an off-white solid. **mp** (*n*-hexane/Et_2O) 146 – 148 °C; **^{1}H NMR** (400 MHz, $CDCl_3$) δ_H 9.43 (s, 1H, N*H*C), 7.86 (d, *J* = 8.0 Hz, 2H, 2 × *p*-MeAr*H*), 7.28 (d, *J* = 8.0 Hz, 2H, 2 × *p*-MeAr*H*), 6.04 (s, 2H, 2 × Ar*H*), 3.86 (s, 3H, OC*H*$_3$), 3.85 (s, 6H, OC*H*$_3$), 2.40 (s, 3H, ArC*H*$_3$); **^{13}C NMR** (101 MHz, $CDCl_3$) δ_H 167.6, 164.5 (2C), 143.4, 137.2, 129.6, 127.8, 104.5, 90.6, 56.2, 55.8, 21.7; **LRMS** (ESI, *m/z*) $[M+H]^+$ 350.0 & $[M+Na]^+$ 372.0. The spectroscopic data is in agreement with literature.[23]

N-(4-(Dimethylamino)benzylidene)-4-methylbenzenesulfonamide (1g)

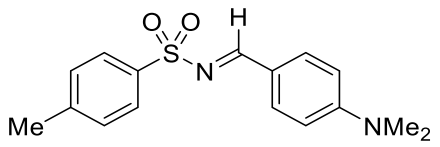

Prepared according to general procedure C, using 4-methylbenzenesulfonamide (171 mg, 1.0 mmol, 1.0equiv.), 4-(dimethylamino)benzaldehyde (0.13 mL, 1.2 mmol, 1.2 equiv.) and pyrrolidine (9 µL, 0.1 mmol, 10 mol%) with stirring for 24 h, affording the title compound (232 mg, 77%) as a bright yellow solid. **mp** (*n*-hexane/Et_2O) 165 – 167 °C; **^{1}H NMR** (400 MHz, $CDCl_3$) δ_H 8.80 (s, 1H, N*H*C), 7.85 (d, *J* = 8.0 Hz, 2H, *p*-MeAr*H*), 7.76 (d, *J* = 9.0 Hz, 2H, *p*-NMe_2Ar*H*), 7.29 (d, *J* = 8.0 Hz, 2H, *p*-MeAr*H*), 6.64 (d, *J* = 9.0 Hz, 2H, *p*-NMe_2Ar*H*), 3.08 (s, 6H, N(CH_3)$_2$), 2.40 (s, 3H, ArCH_3); **^{13}C NMR** (101 MHz, $CDCl_3$) δ_C 169.1, 155.0, 143.6, 137.0, 134.1, 129.7, 127.7, 119.9, 111.4, 40.2, 21.7; **LRMS** (ESI, *m/z*) $[M+H]^+$ 303.0, $[M+Na]^+$ 325.0. The spectroscopic data is in agreement with literature.[24]

N-((4-Methoxyphenyl)(phenyl)methylene)-4-methylbenzenesulfonamide (1h)

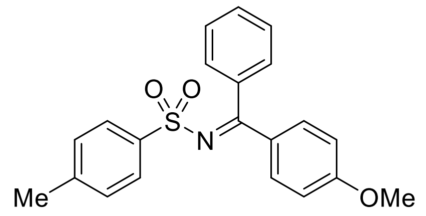

Prepared according to a modified procedure from Ram et al.[25] 4-methylbenzenesulfonamide (428 mg, 2.5 mmol, 1.0 equiv.) and 4-methoxybenzophenone (531 mg, 2.5 mmol, 1.0 equiv.) were added to an appropriate reaction vessel and dissolved in 1,2-dichloroethane (10 mL). $TiCl_4$ (143 µL, 1.3 mmol, 60 mol%) was added with stirring, followed by dropwise addition of triethylamine (700 µL, 5.0 mmol, 2.0 equiv.). The reaction mixture was heated at reflux for 3 – 5 h. After completion of the reaction as indicated by TLC, the mixture was cooled to room temperature and then quenched with 1 M aq. HCl. The layers were separated, and the organic layer was washed by saturated aq. $NaHCO_3$ and water, dried over anhydrous $MgSO_4$, and concentrated under reduced pressure. Purification by flash column chromatography (Petrol:EtOAc, 70:30), afforded the title compound (793 mg, 87%) as a brown oil. **^{1}H NMR** (600 MHz, $CDCl_3$) δ_H 7.81 (d, *J* = 8.0 Hz, 2H, ArH), 7.60 (d, *J* = 8.5 Hz, 2H, ArH), 7.54 – 7.50 (m, 1H, ArH), 7.50 – 7.39 (m, 4H, ArH), 7.27 (app. d, *J* = 8.0 Hz, 2H, ArH), 6.88 (br. s, 2H, ArH), 3.85 (s, 3H, ArOCH_3), 2.43 (s, 3H, ArCH_3); **^{13}C NMR** (151 MHz, $CDCl_3$) δ_C 178.2 (br.), 164.2 (br.), 143.2, 139.0, 136.2 (br.), 133.1 (br.), 129.4, 128.1 (br.), 127.4, 113.8 (br.), 55.7, 21.7, (note: 3 aromatic carbon peaks not observed); LRMS (ESI, *m/z*) $[M+H]^+$ 366.1, $[M+Na]^+$ 388.1;

HRMS (ESI, *m/z*) calculated for $(C_{21}H_{20}NO_3S)^+$ 366.1158 $[M+H]^+$, found 366.1157. The spectroscopic data is in agreement with literature.[25]

N-(4-Methoxybenzylidene)benzenesulfonamide (1ab)

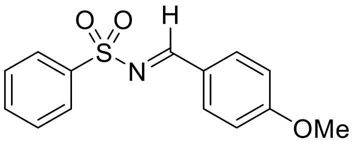

Prepared according to general procedure C, using benzenesulfonamide (157 mg, 1.0 mmol, 1.0 equiv.), 4-methoxybenzaldehyde (0.15 mL, 1.2 mmol, 1.2 equiv.) and pyrrolidine (9 µL, 0.1 mmol, 10 mol%) with stirring for 24 h, affording the title compound (142 mg, 52%) as a colourless solid. **mp** (*n*-hexane/Et_2O) 102 – 104 °C; **^{1}H NMR** (400 MHz, $CDCl_3$) δ_H 8.97 (s, 1H, N*H*C), 8.03 – 7.96 (m, 2H, Ph*H*), 7.89 (d, *J* = 9.0 Hz, 2H, *p*-OMeAr*H*), 7.65 – 7.57 (m, 1H, Ph*H*), 7.57 – 7.49 (m, 2H, Ph*H*), 6.97 (d, *J* = 9.0 Hz, 2H, *p*-OMeAr*H*), 3.88 (s, 3H, OCH_3); **^{13}C NMR** (101 MHz, $CDCl_3$) δ_C 169.8, 165.5, 139.0, 134.0, 133.4, 129.2, 128.0, 125.3, 114.9, 55.8; **LRMS** (ESI, *m/z*) $[M+H]^+$ 276.0, $[M+Na]^+$ 298.0. The spectroscopic data is in agreement with literature.[26]

N-(4-methoxybenzylidene)naphthalene-2-sulfonamide (1ac)

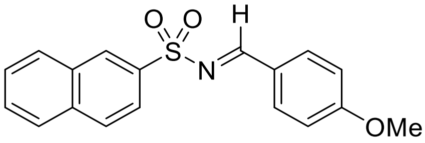

Prepared according to general procedure C, using naphthalene-2-sulfonamide (**9c**) (207 mg, 1.0 mmol, 1.0 equiv.), 4-methoxybenzaldehyde (0.15 mL, 1.2 mmol, 1.2 equiv.) and pyrrolidine (9 µL, 0.1 mmol, 10 mol%) with stirring for 24 h, affording the title compound (255 mg, 78%) as a pale-yellow solid. **mp** (*n*-hexane/Et_2O) 98 – 100 °C; **^{1}H NMR** (400 MHz, $CDCl_3$) δ_H 9.02 (s, 1H, N*H*C), 8.59 (s, 1H, Ar*H*), 8.02 – 7.93 (m, 3H, Ar*H*), 7.93 – 7.87 (m, 3H, *p*-OMeAr*H*, Ar*H*), 7.69 – 7.56 (m, 2H, Ar*H*), 6.96 (d, *J* = 9.0 Hz, 2H, *p*-OMeAr*H*), 3.88 (s, 3H, OCH_3); **^{13}C NMR** (101 MHz, $CDCl_3$) δ_C 169.7, 165.5, 135.8, 135.3, 134.0, 132.3, 129.5, 129.5 (2C), 129.2, 128.1, 127.7, 125.4, 123.1, 114.9, 55.8; **LRMS** (ESI, *m/z*) $[M+H]^+$ 326.0, $[M+Na]^+$ 348.0. The spectroscopic data is in agreement with literature.[27]

N-(4-methoxybenzylidene)-2,4,6-trimethylbenzenesulfonamide (1ad)

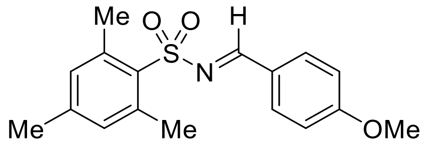

Prepared according to general procedure C, using 2,4,6-trimethylbenzenesulfonamide (**9d**) (200 mg, 1.0 mmol, 1.0 equiv.), 4-methoxybenzaldehyde (0.15 mL, 1.2 mmol, 1.2 equiv.) and pyrrolidine (9 µL, 0.1 mmol, 10 mol%) with stirring for 24 h, affording the title compound (245 mg, 76%) as a colourless solid. **mp** (*n*-hexane/Et_2O) 112 – 114 °C; **^{1}H NMR** (400 MHz, $CDCl_3$) δ_H 8.94 (s, 1H, N*H*C), 7.87 (d, *J* = 9.0 Hz, 2H, *p*-OMeAr*H*), 7.00 – 6.93 (m, 4H, *p*-OMeAr*H*, Ar*H*), 3.88 (s, 3H, OC*H*$_3$), 2.70 (s, 6H, ArC*H*$_3$), 2.30 (s, 3H, ArC*H*$_3$); **^{13}C NMR** (101 MHz, $CDCl_3$) δ_C 168.1, 165.2, 143.1, 140.2, 133.7, 132.8, 132.0, 125.6, 114.8, 55.8, 23.2, 21.2; **LRMS** (ESI, *m/z*) $[M+H]^+$ 318.0, $[M+Na]^+$ 340.0; **HRMS** (ESI, *m/z*) calculated for $(C_{17}H_{20}NO_3S)^+$ 318.1158 $[M+H]^+$, found 318.1156; **IR** ($CHCl_3$, ν_{max}/cm^{-1}) 1588, 1561, 1512, 1425, 1314, 1260, 1150, 1056, 1025, 836, 800, 761, 666; **R$_f$** 0.69 (*n*-hexane:EtOAc, 60:40).

4-Fluoro-N-(4-methoxybenzylidene)benzenesulfonamide (1ae)

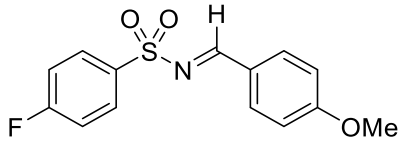

Prepared according to general procedure C, using 4-fluorobenzenesulfonamide (1.75 g, 10.0 mmol, 1.0 equiv.), 4-methoxybenzaldehyde (1.5 mL, 12.0 mmol, 1.2 equiv.) and pyrrolidine (82 µL, 1.0 mmol, 10 mol%) with stirring for 24 h, affording the title compound (2.05 g, 70%) as a colourless solid. **mp** (*n*-hexane/Et_2O) 108 – 110 °C; **^{1}H NMR** (400 MHz, $CDCl_3$) δ_H 8.96 (s, 1H, N*H*C), 8.06 – 7.96 (m, 2H, *p*-FAr*H*), 7.89 (d, *J* = 9.0 Hz, 2H, *p*-OMeAr*H*), 7.25 – 7.16 (m, 2H, *p*-FAr*H*), 6.98 (d, *J* = 9.0 Hz, 2H, *p*-OMeAr*H*), 3.89 (s, 3H, OC*H*$_3$); **^{13}C NMR** (101 MHz, $CDCl_3$) δ_C 169.8, 165.7 (d, *J* = 255.5 Hz), 165.7, 135.1 (d, *J* = 2.5 Hz), 134.0, 130.8 (d, *J* = 9.5 Hz), 125.2, 116.5 (d, *J* = 22.7 Hz), 114.9, 55.9; **^{19}F NMR** (377 MHz, $CDCl_3$) δ_F -104.24; **LRMS** (ESI, *m/z*) $[M+Na]^+$ 316.0; **HRMS** (ESI, *m/z*) calculated for $(C_{14}H_{13}FNO_3S)^+$ 294.0595 $[M+H]^+$, found 294.0593; **IR** ($CHCl_3$, ν_{max}/cm^{-1}) 1587, 1557, 1512, 1494, 1426, 1367, 1291, 1261, 1151, 1086, 1023, 834 802, 764, 675; **R$_f$** 0.30 (*n*-hexane:EtOAc, 60:40).

N-(4-methoxybenzylidene)-4-(trifluoromethyl)benzenesulfonamide (1af)

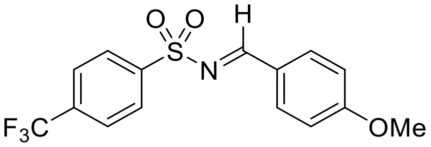

Prepared according to general procedure C, using 4-(trifluoromethyl)-benzenesulfonamide (**9f**) (225 mg, 1.0 mmol, 1.0 equiv.), 4-methoxybenzaldehyde (0.15 mL, 1.2 mmol, 1.2 equiv.) and pyrrolidine (9 µL, 0.1 mmol, 10 mol%) with stirring for 24 h, affording the title compound (148 mg, 43%) as a colourless solid. **mp** (*n*-hexane/Et_2O) 119 – 121 °C; **^{1}H NMR** (400 MHz, $CDCl_3$) δ_H 9.00 (s, 1H, N*H*C), 8.13 (d, *J* = 8.5 Hz, 2H, *p*-CF_3Ar*H*), 7.91 (d, *J* = 9.0 Hz, 2H, *p*-OMeAr*H*), 7.80 (d, *J* = 8.5 Hz, 2H, *p*-CF_3Ar*H*), 6.99 (d, *J* = 9.0 Hz, 2H, *p*-OMeAr*H*), 3.90 (s, 3H, OC*H*$_3$); **^{13}C NMR** (101 MHz, $CDCl_3$) δ_C 170.8, 166.0, 142.7, 135.0 (d, *J* = 33.0 Hz), 134.3, 128.5, 126.3 (q, *J* = 4.0 Hz), 125.1, 123.3 (d, *J* = 273.0 Hz), 115.0, 55.9; **^{19}F NMR** (377 MHz, $CDCl_3$) δ_F -63.18; **LRMS** (ESI, *m/z*) $[M+H]^+$ 344.0, $[M+Na]^+$ 336.0; **HRMS** (ESI, *m/z*) calculated for $(C_{15}H_{13}F_3NO_3S)^+$ 344.0563 $[M+H]^+$, found 344.0558; **IR** ($CHCl_3$, ν_{max}/cm^{-1}) 1589, 1557, 1513, 1426, 1405, 1321, 1264, 1158, 1108, 1062, 1020, 837, 809, 764, 712, 622; **R$_f$** 0.42 (*n*-hexane:EtOAc, 60:40).

4-Cyano-N-(4-methoxybenzylidene)benzenesulfonamide (1ag)

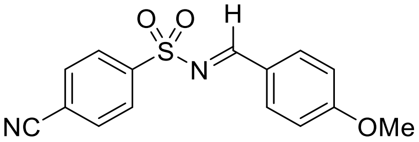

Prepared according to general procedure C, using 4-cyanobenzenesulfonamide (182 mg, 1.0 mmol, 1.0 equiv.), 4-methoxybenzaldehyde (0.15 mL, 1.2 mmol, 1.2 equiv.) and pyrrolidine (9 µL, 0.1 mmol, 10 mol%) with stirring for 24 h, affording the title compound (163 mg, 54%) as a colourless solid. **mp** (*n*-hexane/Et_2O) 142 – 144 °C; **^{1}H NMR** (400 MHz, $CDCl_3$) δ_H 9.00 (s, 1H, N*H*C), 8.11 (d, *J* = 8.5 Hz, 2H, *p*-CNAr*H*), 7.91 (d, *J* = 9.0 Hz, 2H, *p*-OMeAr*H*), 7.83 (d, *J* = 8.5 Hz, 2H, *p*-CNAr*H*), 6.99 (d, *J* = 9.0 Hz, 2H, *p*-OMeAr*H*), 3.90 (s, 3H, OC*H*$_3$); **^{13}C NMR** (101 MHz, $CDCl_3$) δ_C 171.2, 166.1, 143.5, 134.4, 133.0, 128.6, 125.0, 117.4, 117.1, 115.1, 55.9; **LRMS** (ESI, *m/z*) $[M+H]^+$ 301.0, $[M+Na]^+$ 323.0; **HRMS** (ESI, *m/z*) calculated for $(C_{15}H_{13}N_2O_3S)^+$ 301.0641 $[M+H]^+$, found 301.0643; **IR** ($CHCl_3$, ν_{max}/cm^{-1}) 1587, 1556, 1513, 1462, 1321, 1264, 1154, 1087, 1021, 837, 813, 763, 650; **R$_f$** 0.20 (*n*-hexane:EtOAc, 60:40).

4-Acetyl-*N*-(4-methoxybenzylidene)benzenesulfonamide (1ah)

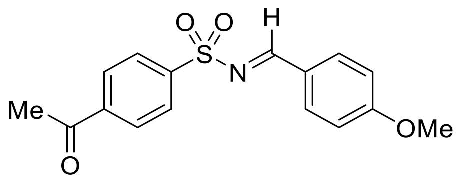

Prepared according to general procedure C, using 4-acetylbenzenesulfonamide (**9h**) (239 mg, 1.2 mmol, 1.0 equiv.), 4-methoxybenzaldehyde (0.12 mL, 1.0 mmol, 1.0 equiv.) and pyrrolidine (9 μL, 0.1 mmol, 10 mol%) with stirring for 48 h. Purification by flash column chromatography (*n*-hexane:EtOAc, 100:0 to 20:80), afforded the title compound (86 mg, 27%) as a yellow solid. **mp** (*n*-hexane/EtOAc) 109 – 111 °C; **^{1}H NMR** (400 MHz, $CDCl_3$) δ_H 9.00 (s, 1H, N*H*C), 8.09 (app. s, 4H, Ar*H*), 7.90 (d, *J* = 9.0 Hz, 2H, *p*-OMeAr*H*), 6.98 (d, *J* = 9.0 Hz, 2H, *p*-OMeAr*H*), 3.89 (s, 3H, OC*H*$_3$), 2.65 (s, 3H, COC*H*$_3$); **^{13}C NMR** (101 MHz, $CDCl_3$) δ_C 196.9, 170.6, 165.9, 143.0, 140.6, 134.2, 129.0, 128.3, 125.2, 115.0, 55.9, 27.0; **LRMS** (ESI, *m/z*) $[M+H]^+$ 318.0, $[M+Na]^+$ 340.0; **HRMS** (ESI, *m/z*) calculated for $(C_{16}H_{16}NO_4S)^+$ 318.0795 $[M+H]^+$, found 318.0796; **IR** ($CHCl_3$, ν_{max}/cm^{-1}) 1689, 1588, 1557, 1513, 1361, 1319, 1260, 1155, 1090, 1022, 808, 778, 756, 647; **R$_f$** 0.16 (*n*-hexane:EtOAc, 60:40).

Methyl 3-(*N*-(4-methoxybenzylidene)sulfamoyl)benzoate (1ai)

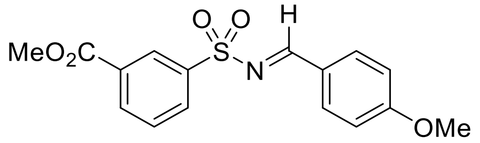

Prepared according to general procedure C, using methyl 3-sulfamoylbenzoate (**9i**) (124 mg, 0.6 mmol, 1.2 equiv.), 4-methoxybenzaldehyde (0.60 mL, 0.5 mmol, 1.0 equiv.) and pyrrolidine (5 μL, 0.05 mmol, 10 mol%) with stirring for 24 h, affording the title compound (40 mg, 24%) as a colourless solid. **mp** (*n*-hexane/Et_2O) 108 – 110 °C; **^{1}H NMR** (400 MHz, $CDCl_3$) δ_H 9.00 (s, 1H, N*H*C), 8.64 (app. td, *J* = 2.0, 0.5 Hz, 1H, Ar*H*), 8.27 (app. dt, *J* = 8.0, 1.5 Hz, 1H, Ar*H*), 8.19 (ddd, *J* = 8.0, 2.0, 1.0 Hz, 1H, Ar*H*), 7.90 (d, *J* = 9.0 Hz, 2H, *p*-OMeAr*H*), 7.63 (app. td, *J* = 8.0, 0.5 Hz, 1H, Ar*H*), 6.98 (d, *J* = 9.0 Hz, 2H, *p*-OMeAr*H*), 3.95 (s, 3H, CO_2C*H*$_3$), 3.89 (s, 3H, OC*H*$_3$); **^{13}C NMR** (101 MHz, $CDCl_3$) δ_C 170.4, 165.8, 165.6, 139.8, 134.3, 134.1, 132.0, 131.5, 129.5, 129.1, 125.2, 114.9, 55.9, 52.7; **LRMS** (ESI, *m/z*) $[M+H]^+$ 334.0, $[M+Na]^+$ 356.0; **HRMS** (ESI, *m/z*) calculated for $(C_{16}H_{16}NO_5S)^+$ 334.0744 $[M+H]^+$, found 334.0743; **IR** ($CHCl_3$, ν_{max}/cm^{-1}) 1727, 1589, 1561, 1513, 1426, 1305, 1266, 1156, 1123, 1023, 839, 807, 773, 753, 611; **R$_f$** 0.43 (*n*-hexane:EtOAc, 60:40).

4-Bromo-*N*-(4-methoxybenzylidene)benzenesulfonamide (1aj)

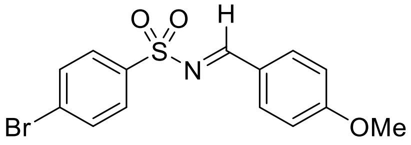

Prepared according to general procedure C, using 4-bromobenzenesulfonamide (236 mg, 1.0 mmol, 1.0 equiv.), 4-methoxybenzaldehyde (0.15 mL, 1.2 mmol, 1.2 equiv.) and pyrrolidine (9 μL, 0.05 mmol, 10 mol%) with stirring for 24 h, affording the title compound (233 mg, 66%) as a colourless solid. **mp** (*n*-hexane/Et_2O) 115 – 117 °C; **^{1}H NMR** (400 MHz, $CDCl_3$) δ_H 8.96 (s, 1H, N*H*C), 7.89 (d, *J* = 9.0 Hz, 2H, *p*-OMeAr*H*), 7.86 (d, *J* = 8.5 Hz, 2H, Ar*H*), 7.67 (d, *J* = 8.5 Hz, 2H, Ar*H*), 6.98 (d, *J* = 9.0 Hz, 2H, *p*-OMeAr*H*), 3.89 (s, 3H, OC*H*$_3$); **^{13}C NMR** (101 MHz, $CDCl_3$) δ_C 170.1, 165.8, 138.1, 134.1, 132.5, 129.5, 128.6, 125.2, 114.9, 55.9; **LRMS** (ESI, *m/z*) [^{79}BrM+Na]$^+$ 376.0, [^{81}BrM+Na]$^+$ 378.0. The spectroscopic data is in agreement with literature.[28]

3-Bromo-*N*-(4-methoxybenzylidene)benzenesulfonamide (1ak)

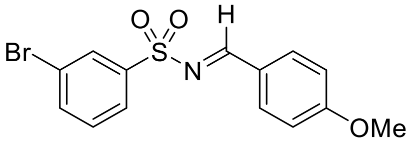

Prepared according to general procedure C, using 3-bromobenzenesulfonamide (**9k**) (236 mg, 1.0 mmol, 1.0 equiv.), 4-methoxybenzaldehyde (0.15 mL, 1.2 mmol, 1.2 equiv.) and pyrrolidine (9 μL, 0.05 mmol, 10 mol%) with stirring for 24 h, affording the title compound (55 mg, 16%) as a colourless solid. **mp** (*n*-hexane/Et_2O) 79 – 81 °C; **^{1}H NMR** (400 MHz, $CDCl_3$) δ_H 8.97 (s, 1H, N*H*C), 8.13 (app. t, *J* = 2.0 Hz, 1H, Ar*H*), 7.97 – 7.85 (m, 3H, Ar*H*, *p*-OMeAr*H*), 7.73 (ddd, *J* = 8.0, 2.0, 1.0 Hz, 1H, Ar*H*), 7.41 (app. t, *J* = 8.0 Hz, 1H), 6.99 (d, *J* = 9.0 Hz, 2H, *p*-OMeAr*H*), 3.90 (s, 3H, OC*H*$_3$); **^{13}C NMR** (101 MHz, $CDCl_3$) δ_C 170.5, 165.8, 140.9, 136.4, 134.2, 130.9, 130.7, 126.5, 125.2, 123.1, 115.0, 55.9; **LRMS** (ESI, *m/z*) [^{79}BrM+Na]$^+$ 375.9, [^{81}BrM+Na]$^+$ 377.9; **HRMS** (ESI, *m/z*) calculated for $(C_{14}H_{13}{}^{79}BrNO_3S)^+$ 353.9794 [^{79}BrM+H]$^+$, found 353.9792, calculated for $(C_{14}H_{13}{}^{81}BrNO_3S)^+$ 355.9773 [^{81}BrM+H]$^+$, found 355.9771; **IR** ($CHCl_3$, ν_{max}/cm^{-1}) 1589, 1557, 1513, 1461, 1426, 1320, 1264, 1156, 1099, 1023, 873, 837, 782, 753, 678, 658, 611; **R_f** 0.62 (*n*-hexane:EtOAc, 60:40).

2-Bromo-*N*-(4-methoxybenzylidene)benzenesulfonamide (1al)

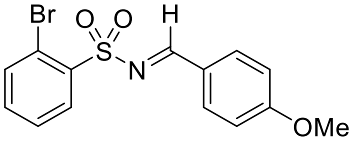

Prepared according to general procedure C, using 2-bromobenzenesulfonamide (**9l**) (236 mg, 1.0 mmol, 1.0 equiv.), 4-methoxybenzaldehyde (0.15 mL, 1.2 mmol, 1.2 equiv.) and pyrrolidine (9 µL, 0.05 mmol, 10 mol%) with stirring for 24 h, affording the title compound (113 mg, 32%) as a colourless solid. **mp** (*n*-hexane/Et_2O) 102 – 104 °C; **^{1}H NMR** (400 MHz, $CDCl_3$) δ_H 9.13 (s, 1H, N*H*C), 8.33 (dd, *J* = 8.0, 2.0 Hz, 1H, Ar*H*), 7.93 (d, *J* = 9.0 Hz, 2H, *p*-OMeAr*H*), 7.70 (dd, *J* = 8.0, 1.5 Hz, 1H, Ar*H*), 7.52 (app. td, *J* = 7.5, 1.5 Hz, 1H, Ar*H*), 7.43 (app. td, *J* = 7.5, 2.0 Hz, 1H, Ar*H*), 6.99 (d, *J* = 9.0 Hz, 2H, *p*-OMeAr*H*), 3.89 (s, 3H, OC*H*$_3$); **^{13}C NMR** (101 MHz, $CDCl_3$) δ_C 172.6, 165.8, 137.9, 135.4, 134.4, 134.2, 131.6, 128.0, 125.2, 121.2, 115.0, 55.9; **LRMS** (ESI, *m/z*) [^{79}BrM+H]$^+$ 354.0, [^{81}BrM+H]$^+$ 356.0, [^{79}BrM+Na]$^+$ 376.0, [^{81}BrM+Na]$^+$ 378.0; **HRMS** (ESI, *m/z*) calculated for $(C_{14}H_{13}{}^{79}BrNO_3S)^+$ 353.9794 [^{79}BrM+H]$^+$, found 353.9792, calculated for $(C_{14}H_{13}{}^{81}BrNO_3S)^+$ 355.9773 [^{81}BrM+H]$^+$, found 355.9771; **IR** ($CHCl_3$, ν_{max}/cm^{-1}) 1589, 1558, 1513, 1426, 1319, 1264, 1157, 1125, 1101, 1026, 837, 809, 770, 734, 607; **R$_f$** 0.60 (*n*-hexane:EtOAc, 60:40).

4-Methoxy-*N*-(4-methoxybenzylidene)benzenesulfonamide (1am)

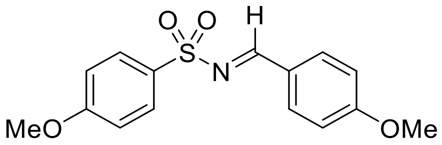

Prepared according to general procedure C, using 4-methoxybenzenesulfonamide (187 mg, 1.0 mmol, 1.0 equiv.), 4-methoxybenzaldehyde (0.15 mL, 1.2 mmol, 1.2 equiv.) and pyrrolidine (9 µL, 0.1 mmol, 10 mol%) with stirring for 24 h, affording the title compound (171 mg, 56%) as a colourless solid. **mp** (*n*-hexane/Et_2O) 83 – 85 °C; **^{1}H NMR** (400 MHz, $CDCl_3$) δ_H 8.91 (s, 1H, N*H*C), 7.92 (d, *J* = 9.0 Hz, 2H, *p*-OMeAr*H*), 7.87 (d, *J* = 9.0 Hz, 2H, *p*-OMeAr*H*), 6.99 (d, *J* = 9.0 Hz, 2H, *p*-OMeAr*H*), 6.96 (d, *J* = 9.0 Hz, 2H, *p*-OMeAr*H*), 3.87 (s, 3H, OC*H*$_3$), 3.86 (s, 3H, OC*H*$_3$); **^{13}C NMR** (101 MHz, $CDCl_3$) δ_C 168.8, 165.3, 163.6, 133.8, 130.3, 130.2, 125.4, 114.8, 114.5, 55.8 (2C); **LRMS** (ESI, *m/z*) [M+H]$^+$ 306.0; **HRMS** (ESI, *m/z*) calculated for $(C_{15}H_{16}NO_4S)^+$ 306.0795 [M+H]$^+$, found 306.0791; **IR** ($CHCl_3$, ν_{max}/cm^{-1}) 1592, 1561, 1513, 1499, 1318, 1259, 1150, 1090, 1023, 835, 810, 762, 674; **R$_f$** 0.25 (*n*-hexane:EtOAc, 60:40).

2,4-Dimethoxy-*N*-(4-methoxybenzylidene)benzenesulfonamide (1an)

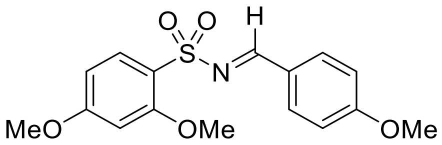

Prepared according to general procedure C, using 2,4-dimethoxybenzenesulfonamide (**9n**) (217 mg, 1.0 mmol, 1.0 equiv.), 4-methoxybenzaldehyde (0.15 mL, 1.2 mmol, 1.2 equiv.) and pyrrolidine (9 µL, 0.1 mmol, 10 mol%) with stirring for 24 h, affording the title compound (207 mg, 62%) as a colourless solid. **mp** (*n*-hexane/Et_2O) 136 – 138 °C; **^{1}H NMR** (400 MHz, $CDCl_3$) δ_H 8.99 (s, 1H, N*H*C), 8.01 (d, *J* = 9.0 Hz, 1H, Ar*H*), 7.89 (d, *J* = 9.0 Hz, 2H, *p*-OMeAr*H*), 6.97 (d, *J* = 9.0 Hz, 2H, *p*-OMeAr*H*), 6.58 (dd, *J* = 9.0, 2.5 Hz, 1H, Ar*H*), 6.43 (d, *J* = 2.5 Hz, 1H, Ar*H*), 3.87 (s, 3H, OCH_3), 3.84 (s, 3H, OCH_3), 3.77 (s, 3H, OCH_3); **^{13}C NMR** (101 MHz, $CDCl_3$) δ_C 171.3, 165.7, 165.2, 158.7, 133.7, 132.5, 125.6, 118.2, 114.8, 104.9, 99.5, 56.2, 55.9, 55.8; **LRMS** (ESI, *m/z*) $[M+H]^+$ 336.0, $[M+Na]^+$ 358.0; **HRMS** (ESI, *m/z*) calculated for $(C_{16}H_{18}NO_5S)^+$ 336.0900 $[M+H]^+$, found 336.0896; **IR** ($CHCl_3$, ν_{max}/cm^{-1}) 1590, 1561, 1513, 1464, 1424, 1306, 1259, 1214, 1159, 1139, 1074, 1023, 873, 836, 806, 764, 676, 611; **R_f** 0.38 (*n*-hexane:EtOAc, 40:60).

***N*-(4-Methoxybenzylidene)methanesulfonamide (1ao)**

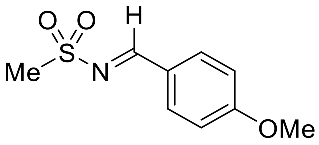

Prepared according to general procedure C, using methanesulfonamide (95 mg, 1.0 mmol, 1.0 equiv.), 4-methoxybenzaldehyde (0.15 mL, 1.2 mmol, 1.2 equiv.) and pyrrolidine (9 µL, 0.05 mmol, 10 mol%) with stirring for 24 h, affording the title compound (67 mg, 31%) as a colourless solid. **mp** (*n*-hexane/Et_2O) 78 – 80 °C; **^{1}H NMR** (400 MHz, $CDCl_3$) δ_H 8.93 (s, 1H, N*H*C), 7.92 (d, *J* = 9.0 Hz, 2H, *p*-OMeAr*H*), 7.01 (d, *J* = 9.0 Hz, 2H, *p*-OMeAr*H*), 3.91 (s, 3H, OCH_3), 3.11 (s, 3H, SO_2CH_3); **^{13}C NMR** (101 MHz, $CDCl_3$) δ_C 170.6, 165.6, 133.9, 125.1, 114.9, 55.9, 40.6; **LRMS** (ESI, *m/z*) $[M+H]^+$ 214.0, $[M+Na]^+$ 236.0; **HRMS** (ESI, *m/z*) calculated for $(C_9H_{12}NO_3S)^+$ 214.0532 $[M+H]^+$, found 214.0533; **IR** ($CHCl_3$, ν_{max}/cm^{-1}) 1593, 1566, 1514, 1426, 1306, 1261, 1139, 1022, 965, 810, 783, 741; **R_f** 0.16 (*n*-hexane:EtOAc, 60:40).

***N*-(4-Methoxybenzylidene)propane-1-sulfonamide (1ap)**

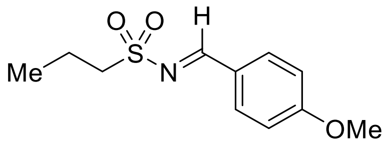

Prepared according to general procedure C, using propane-1-sulfonamide (**9p**) (123 mg, 1.0 mmol, 1.0 equiv.), 4-methoxybenzaldehyde (0.15 mL, 1.2 mmol, 1.0 equiv.) and pyrrolidine (9 μL, 0.1 mmol, 10 mol%) with stirring for 48 h. Purification by flash column chromatography (*n*-hexane:EtOAc, 100:0 to 20:80), afforded the title compound (167 mg, 69%) as a pale-yellow oil. **^{1}H NMR** (400 MHz, $CDCl_3$) δ_H 8.87 (s, 1H, N*H*C), 7.87 (d, *J* = 9.0 Hz, 2H, *p*-OMeAr*H*), 6.97 (d, *J* = 9.0 Hz, 2H, *p*-OMeAr*H*), 3.86 (s, 3H, OC*H*$_3$), 3.19 – 3.08 (m, 2H, SO_2CH_2), 1.94 – 1.77 (m, 2H, $CH_2CH_2CH_3$), 1.04 (t, *J* = 7.5 Hz, 3H, $(CH_2)_2CH_3$); **^{13}C NMR** (101 MHz, $CDCl_3$) δ_C 170.9, 165.4, 133.7, 125.1, 114.8, 55.7, 54.3, 16.9, 13.0; **LRMS** (ESI, *m/z*) $[M+H]^+$ 242.2, $[M+Na]^+$ 264.0; **HRMS** (ESI, *m/z*) calculated for $(C_{11}H_{16}NO_3S)^+$ 242.0845 $[M+H]^+$, found 242.0845; **IR** ($CHCl_3$, ν_{max}/cm^{-1}) 1591, 1564, 1513, 1426, 1313, 1260, 1169, 1135, 1023, 766; **R$_f$** 0.32 (*n*-hexane:EtOAc, 60:40).

***N*-(4-Methoxybenzylidene)cyclohexanesulfonamide (1aq)**

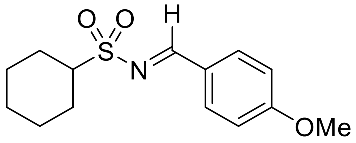

Prepared according to general procedure C, using cyclohexanesulfonamide (**9q**) (392 mg, 2.4 mmol, 1.0 equiv.), 4-methoxybenzaldehyde (0.38 mL, 2.9 mmol, 1.2 equiv.) and pyrrolidine (18 μL, 0.2 mmol, 10 mol%) with stirring for 48 h, affording the title compound (211 mg, 31%) as a colourless solid. **mp** (*n*-hexane/Et_2O) 88 – 90 °C; **^{1}H NMR** (400 MHz, $CDCl_3$) δ_H 8.93 (s, 1H, N*H*C), 7.92 (d, *J* = 9.0 Hz, 2H, *p*-OMeAr*H*), 7.00 (d, *J* = 9.0 Hz, 2H, *p*-OMeAr*H*), 3.91 (s, 3H, OC*H*$_3$), 3.06 (tt, *J* = 12.0, 3.5 Hz, 1H, Cy*H*), 2.23 (ddd, *J* = 12.0, 4.0, 2.0 Hz, 2H, Cy*H*), 1.90 (app. dq, *J* = 13.0, 3.0 Hz, 2H, Cy*H*), 1.76 – 1.66 (m, 1H, Cy*H*), 1.56 (app. qd, *J* = 12.0, 3.5 Hz, 2H, Cy*H*), 1.36 – 1.13 (m, 3H, Cy*H*); **^{13}C NMR** (101 MHz, $CDCl_3$) δ_C 171.5, 165.4, 133.7, 125.4, 114.7, 60.9, 55.8, 26.8, 26.1, 25.3; **LRMS** (ESI, *m/z*) $[M+H]^+$ 304.0; **HRMS** (ESI, *m/z*) calculated for $(C_{14}H_{20}NO_3S)^+$ 282.1158 $[M+H]^+$, found 282.1157; **IR** ($CHCl_3$, ν_{max}/cm^{-1}) 2936, 2857, 1591, 1564, 1513, 1453, 1425, 1307, 1261, 1169, 1138, 1023, 827, 802, 776, 750, 622; **R$_f$** 0.68 (*n*-hexane:EtOAc, 60:40).

***N*-(4-Methoxybenzylidene)-2-methylpropane-2-sulfonamide (1ar)**

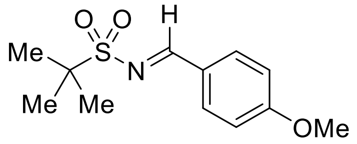

Prepared according to general procedure C, using *tert*-butylsulfonamide (137 mg, 1.0 mmol, 1.0 equiv.), 4-methoxybenzaldehyde (0.15 mL, 1.2 mmol, 1.2 equiv.) and pyrrolidine (9 µL, 0.1 mmol, 10 mol%) with stirring for 24 h, affording the title compound (145 mg, 57%) as a colourless solid. **mp** (*n*-hexane/Et_2O) 74 – 76 °C; **^{1}H NMR** (400 MHz, $CDCl_3$) δ_H 8.88 (s, 1H, N*H*C), 7.88 (d, *J* = 9.0 Hz, 2H, *p*-OMeAr*H*), 6.96 (d, *J* = 9.0 Hz, 2H, *p*-OMeAr*H*), 3.85 (s, 3H, OC*H*$_3$), 1.43 (s, 9H, C(C*H*$_3$)$_3$); **^{13}C NMR** (101 MHz, $CDCl_3$) δ_C 171.8, 165.3, 133.5, 125.3, 114.8, 58.2, 55.7, 24.1; **LRMS** (ESI, *m/z*) $[M+Na]^+$ 278.0. The spectroscopic data is in agreement with literature.[29]

***N*-(4-Methoxybenzylidene)cyclopropanesulfonamide (1as)**

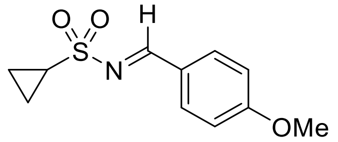

Prepared according to general procedure C, using cyclopropanesulfonamide (**9s**) (217 mg, 2.0 mmol, 1.0 equiv.), 4-methoxybenzaldehyde (0.30 mL, 2.4 mmol, 1.2 equiv.) and pyrrolidine (18 µL, 0.2 mmol, 10 mol%) with stirring for 24 h, affording the title compound (199 mg, 41%) as a colourless solid. **mp** (*n*-hexane/Et_2O) 79 – 81 °C; **^{1}H NMR** (400 MHz, $CDCl_3$) δ_H 8.85 (s, 1H, N*H*C), 7.91 (d, *J* = 9.0 Hz, 2H, *p*-OMeAr*H*), 6.99 (d, *J* = 9.0 Hz, 2H, *p*-OMeAr*H*), 3.89 (s, 3H, OC*H*$_3$), 2.57 (tt, *J* = 8.0, 4.5 Hz, 1H, $(CH_2)_2$C*H*), 1.36 – 1.23 (m, 2H, $(CH_AH_B)_2$CH), 1.15 – 0.99 (m, 2H, $(CH_AH_B)_2$CH); **^{13}C NMR** (101 MHz, $CDCl_3$) δ_C 170.1, 165.4, 133.7, 125.2, 114.8, 55.8, 29.7, 6.0; **LRMS** (ESI, *m/z*) $[M+H]^+$ 240.0, $[M+Na]^+$ 262.0; **HRMS** (ESI, *m/z*) calculated for $(C_{11}H_{14}NO_3S)^+$ 240.0689 $[M+H]^+$, found 240.0688; **IR** ($CHCl_3$, ν_{max}/cm^{-1}) 1589, 1561, 1513, 1425, 1319, 1298, 1260, 1169, 1137, 1023, 887, 806, 768, 705; **R_f** 0.26 (*n*-hexane:EtOAc, 60:40).

***N*-(4-Methoxybenzylidene)thiophene-2-sulfonamide (1at)**

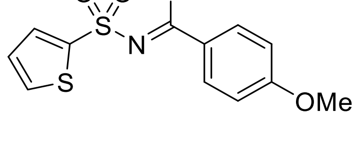

Prepared according to general procedure C, using thiophene-2-sulfonamide (163 mg, 1.0 mmol, 1.2 equiv.), 4-methoxybenzaldehyde (0.15 mL, 1.0 mmol, 1.0 equiv.) and pyrrolidine (9 µL, 0.1 mmol, 10 mol%) with

stirring for 24 h, affording the title compound (171 mg, 61%) as a colourless solid. **mp** (*n*-hexane/Et_2O) 94 – 96 °C; **^{1}H NMR** (400 MHz, $CDCl_3$) δ_H 8.91 (s, 1H, N*H*C), 7.90 (d, *J* = 9.0 Hz, 2H, *p*-OMeAr*H*), 7.76 (dd, *J* = 4.0, 1.5 Hz, 1H, HetAr*H*), 7.67 (dd, *J* = 5.0, 1.5 Hz, 1H, HetAr*H*), 7.12 (dd, *J* = 5.0, 4.0 Hz, 1H, HetAr*H*), 6.98 (d, *J* = 9.0 Hz, 2H, *p*-OMeAr*H*), 3.89 (s, 3H, OC*H*$_3$); **^{13}C NMR** (101 MHz, $CDCl_3$) δ_C 169.5, 165.7, 139.4, 134.1, 133.7, 133.6, 127.7, 125.1, 114.9, 55.9; **LRMS** (ESI, *m/z*) $[M+H]^+$ 282.0, $[M+Na]^+$ 304.0. The spectroscopic data is in agreement with literature.[30]

***N*-(4-Methoxybenzylidene)pyridine-3-sulfonamide (1au)**

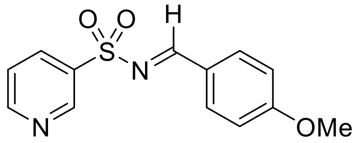

Prepared according to general procedure C, using pyridine-3-sulfonamide (**9u**) (285 mg, 1.8 mmol, 1.2 equiv.), 4-methoxybenzaldehyde (0.18 mL, 1.5 mmol, 1.0 equiv.) and pyrrolidine (14 μL, 0.15 mmol, 10 mol%) with stirring for 48 h, affording the title compound (211 mg, 51%) as a colourless solid. **mp** (*n*-hexane/Et_2O) 140 – 142 °C; **^{1}H NMR** (400 MHz, $CDCl_3$) δ_H 9.20 (d, *J* = 2.0 Hz, 1H, Py*H*), 9.01 (s, 1H, N*H*C), 8.83 (dd, *J* = 4.5, 2.0 Hz, 1H, Py*H*), 8.28 (app. dt, *J* = 8.0, 2.0 Hz, 1H, Py*H*), 7.91 (d, *J* = 9.0 Hz, 2H, *p*-OMeAr*H*), 7.48 (dd, *J* = 8.0, 4.5 Hz, 1H, Py*H*), 6.99 (d, *J* = 9.0 Hz, 2H, *p*-OMeAr*H*), 3.90 (s, 3H, OC*H*$_3$); **^{13}C NMR** (101 MHz, $CDCl_3$) δ_C 170.8, 166.0, 153.8, 148.8, 136.0, 135.6, 134.3, 125.0, 123.8, 115.0, 55.9; **LRMS** (ESI, *m/z*) $[M+H]^+$ 277.0, $[M+Na]^+$ 299.0; **HRMS** (ESI, *m/z*) calculated for $(C_{13}H_{13}N_2O_3S)^+$ 277.0641 $[M+H]^+$, found 277.0639; **IR** ($CHCl_3$, ν_{max}/cm^{-1}) 1589, 1558, 1513, 1426, 1317, 1264, 1160, 1103, 1021, 874, 838, 805, 738, 701, 625, 609; **R_f** 0.34 (*n*-hexane:EtOAc, 40:60).

***N*-(4-Methoxybenzylidene)-5-methylpyridine-2-sulfonamide (1av)**

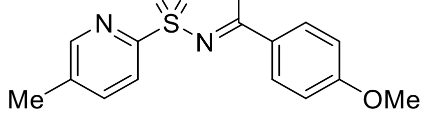

Prepared according to general procedure C, using 5-methylpyridine-2-sulfonamide (172 mg, 1.0 mmol, 1.0 equiv.), 4-methoxybenzaldehyde (0.15 mL, 1.2 mmol, 1.2 equiv.) and pyrrolidine (9 μL, 0.1 mmol, 10 mol%) with stirring for 24 h, affording the title compound (202 mg, 69%) as a colourless solid. **mp** (*n*-hexane/Et_2O) 134 – 136 °C; **^{1}H NMR** (400 MHz, $CDCl_3$) δ_H 9.13 (s, 1H, N*H*C), 8.52 (app. dt, *J* = 2.0, 1.0 Hz, 1H, HetAr*H*), 8.11 (dd, *J* = 8.0, 1.0 Hz, 1H, HetAr*H*), 7.92 (d, *J* = 9.0 Hz, 2H, *p*-OMeAr*H*), 7.73 (ddd, *J* = 8.0, 2.0, 1.0 Hz, 1H, HetAr*H*), 6.97 (d, *J* = 9.0 Hz, 2H, *p*-OMeAr*H*), 3.89 (s, 3H, OC*H*$_3$), 2.43 (app. t, *J* = 1.0 Hz, 3H, HetArC*H*$_3$); **^{13}C NMR** (101 MHz, $CDCl_3$) δ 172.8, 165.7, 153.7, 151.0, 138.2, 137.9, 134.3,

125.4, 123.1, 114.9, 55.9, 18.7; **LRMS** (ESI, *m/z*) $[M+H]^+$ 291.1, $[M+Na]^+$ 313.1; **HRMS** (ESI, *m/z*) calculated for $(C_{14}H_{15}N_2O_3S)^+$ 291.0798 $[M+H]^+$, found 291.0797; **IR** ($CHCl_3$, ν_{max}/cm^{-1}) 1590, 1557, 1513, 1426, 1318, 1264, 1163, 1100, 1025, 831, 802, 765, 671; $\mathbf{R_f}$ 0.19 (*n*-hexane:EtOAc, 60:40).

N-(4-methoxybenzylidene)-4-phenylpiperidine-1-sulfonamide (1aw)

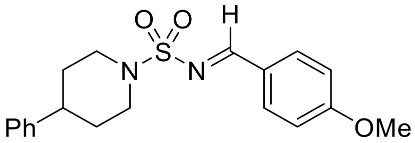

Prepared according to general procedure C, using 4-phenylpiperidine-1-sulfonamide (**9w**) (120 mg, 0.50 mmol, 1.0 equiv.), 4-methoxybenzaldehyde (0.08 mL, 0.65 mmol, 1.2 equiv.) and pyrrolidine (5 µL, 0.05 mmol, 10 mol%) with stirring for 24 h, affording the title compound (136 mg, 76%) as a colourless solid. **mp** (*n*-hexane/Et_2O) 130 – 132 °C; ^{1}**H NMR** (400 MHz, $CDCl_3$) δ_H 8.84 (s, 1H, N*H*C), 7.92 (d, *J* = 9.0 Hz, 2H, *p*-OMeAr*H*), 7.34 – 7.27 (m, 2H, Ph*H*), 7.25 – 7.15 (m, 3H, Ph*H*), 7.02 (d, *J* = 9.0 Hz, 2H, *p*-OMeAr*H*), 4.02 – 3.92 (m, 2H, $NCH_AH_BCH_2$), 3.91 (s, 3H, OCH_3), 2.91 – 2.80 (m, 2H, $NCH_AH_BCH_2$), 2.58 (tt, *J* = 10.5, 5.5 Hz, 1H, $PhCH(CH_2)_2$), 1.96 – 1.80 (m, 4H, NCH_2CH_2); ^{13}**C NMR** (101 MHz, $CDCl_3$) δ_C 169.6, 165.1, 145.2, 133.4, 128.7, 126.9, 126.7, 125.5, 114.9, 55.8, 47.4, 42.1, 32.6; **LRMS** (ESI, *m/z*) $[M+Na]^+$ 381.2; **HRMS** (ESI, *m/z*) calculated for $(C_{19}H_{23}N_2O_3S)^+$ 359.1424 $[M+H]^+$, found 359.1420; **IR** ($CHCl_3$, ν_{max}/cm^{-1}) 1595, 1566, 1513, 1350, 1262, 1155, 1114, 1055, 1023, 937, 873, 806, 766, 701; $\mathbf{R_f}$ 0.33 (*n*-hexane:EtOAc, 60:40).

Methyl 2,4-dichloro-5-(*N*-(4-methoxybenzylidene)sulfamoyl)benzoate (1ax)

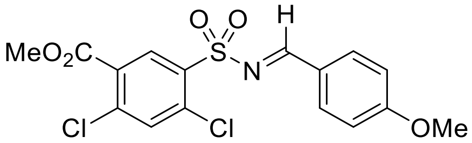

Prepared according to general procedure C, using 2,4-dichloro-5-sulfamoylphenyl acetate (**9x**) (682 mg, 2.4 mmol, 1.0 equiv.), 4-methoxybenzaldehyde (0.38 mL, 2.9 mmol, 1.2 equiv.) and pyrrolidine (18 µL, 0.2 mmol, 10 mol%) with stirring for 24 h, affording the title compound (648 mg, 67%) as a colourless solid. **mp** (*n*-hexane/Et_2O) 148 – 150 °C; ^{1}**H NMR** (400 MHz, $CDCl_3$) δ_H 9.08 (s, 1H, N*H*C), 8.75 (s, 1H, Ar*H*), 7.92 (d, *J* = 9.0 Hz, 2H, *p*-OMeAr*H*), 7.61 (s, 1H, Ar*H*), 6.99 (d, *J* = 8.9 Hz, 2H, *p*-OMeAr*H*), 3.96 (s, 3H, CO_2CH_3), 3.90 (s, 3H, OCH_3); ^{13}**C NMR** (101 MHz, $CDCl_3$) δ_C 173.0, 166.2, 163.8, 139.9, 136.6, 135.2, 134.5, 134.1, 128.9, 125.0, 115.1, 114.4, 55.9, 53.1; **LRMS** (ESI, *m/z*) $[M+H]^+$ 402.0, 404.0; **HRMS** (ESI, *m/z*) calculated for $(C_{16}H_{14}{}^{35}Cl_2NO_5S)^+$ 401.9964 $[M+H]^+$, found 401.9960; **IR** ($CHCl_3$, ν_{max}/cm^{-1}) 1736, 1584,

1554, 1512, 1427, 1290, 1255, 1156, 1075, 1022, 893, 838, 808, 765, 686, 654, 621; **R$_f$** 0.60 (*n*-hexane:EtOAc, 60:40).

6-Chloro-*N*-(4-methoxybenzylidene)-3,4-dihydro-2*H*-benzo[*e*][1,2,4]thiadiazine-7-sulfonamide 1,1-dioxide (1ay)

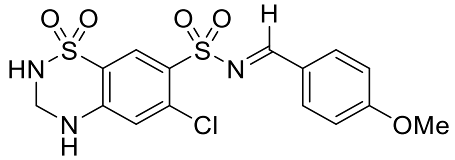

Under an atmosphere of air, hydrochlorothiazide (595 mg, 2.0 mmol, 1.0 equiv.) was added to a round bottom flask equipped with a reflux condenser and MeOH (3 mL) was added. 4-Methoxybenzaldehyde (0.48 mL, 4.0 mmol, 2.0 equiv.) and conc. HCl (2 µL, 20 mol%) were added and the resulting suspension was refluxed at 65 °C for 18 h, then the reaction was cooled to room temperature and the solvent removed under reduced pressure. Purification by washing with MeOH (10 mL) then CH_2Cl_2 (10 mL) afforded the title compound (125 mg, 30%) as a colourless solid. **mp** (*n*-hexane/Et_2O) 192 – 194 °C; **^{1}H NMR** (400 MHz, DMSO-d_6) δ_H 9.10 (s, 1H, N*H*C), 8.32 (br. t, *J* = 3.0 Hz, 1H, SO_2N*H*), 8.11 (s, 1H, Ar*H*), 8.05 (m, 3H, ArN*H*, *p*-OMeAr*H*), 7.12 (d, *J* = 8.5 Hz, 2H, *p*-OMeAr*H*), 7.00 (s, 1H, Ar*H*), 4.77 (s, 2H, $(NH)_2CH_2$), 3.88 (s, 3H, OCH_3); **^{13}C NMR** (101 MHz, DMSO-d_6) δ_C 172.0, 165.3, 148.0, 135.1, 134.1, 127.5, 124.7, 121.2, 119.0, 117.4, 115.0, 55.9, 54.4; **LRMS** (ESI, *m/z*) $[M+H]^+$ 416.1, $[M+Na]^+$ 438.1; **HRMS** (ESI, *m/z*) calculated for $(C_{15}H_{15}{}^{35}ClN_3O_5S_2)^+$ 416.0136 $[M+H]^+$, found 416.0136; **IR** (thin film, ν_{max}/cm^{-1}) 3411, 3304, 1597, 1585, 1546, 1509, 1456, 1427, 1368, 1335, 1309, 1293, 1267, 1165, 1151, 1109, 1055, 1022, 915, 867, 837, 806, 770, 729, 710, 686, 608; **R$_f$** 0.24 (MeOH:EtOAc, 10:90).

N-(4-(*N*-(4-methoxybenzylidene)sulfamoyl)phenyl)acetamide (1az)

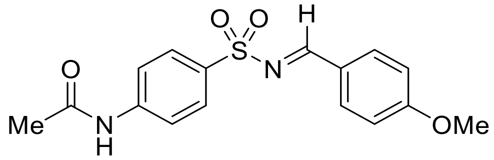

Prepared according to general procedure C, using *N*-(4-sulfamoylphenyl)acetamide (**9z**) (214 mg, 1.0 mmol, 1.0 equiv.), 4-methoxybenzaldehyde (0.15 mL, 1.2 mmol, 1.2 equiv.) and pyrrolidine (9 µL, 0.1 mmol, 10 mol%) with stirring for 24 h, affording the title compound (115 mg, 36%) as an off-white solid. **mp** (*n*-hexane/Et_2O) 166 – 168 °C; **^{1}H NMR** (400 MHz, DMSO-d_6) δ_H 10.40 (br. s, 1H, CON*H*Ar), 9.00 (s, 1H, N*H*C), 7.99 (d, *J* = 9.0 Hz, 2H, *p*-OMeAr*H*), 7.86 (d, *J* = 9.5 Hz, 2H, Ar*H*), 7.82 (d, *J* = 9.5 Hz, 2H, Ar*H*), 7.10 (d, *J* = 9.0 Hz, 2H, *p*-OMeAr*H*), 3.86 (s, 3H, OCH_3), 2.09 (s, 3H, COCH_3); **^{13}C NMR** (101 MHz, DMSO-d_6) δ_C 169.9, 169.1, 165.0, 143.9, 133.7, 131.6, 128.7, 124.9, 118.8, 114.9, 55.8, 24.2; **LRMS** (ESI, *m/z*) $[M+H]^+$

333.0, [M+Na]$^+$ 355.0; **HRMS** (ESI, *m/z*) calculated for $(C_{16}H_{17}N_2O_4S)^+$ 333.0904 [M+H]$^+$, found 333.0900; **IR** ($CHCl_3$, ν_{max}/cm^{-1}) 3340, 1680. 1590, 1531, 1319, 1262, 1152, 1023, 836, 805, 767, 639, 624; **R$_f$** 0.16 (*n*-hexane:EtOAc, 40:60).

N-(4-Methoxybenzylidene)-4-(5-(*p*-tolyl)-3-(trifluoromethyl)-1*H*-pyrazol-1-yl)benzene-sulfonamide (3a)

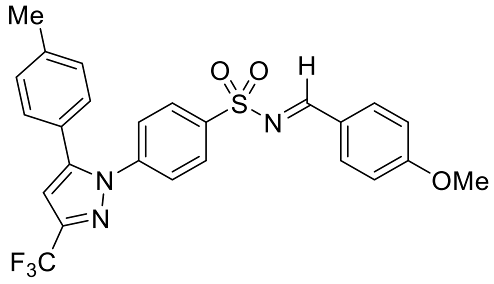

Prepared according to general procedure C, using celecoxib (5.00 g, 13.1 mmol, 1.0 equiv.), 4-methoxybenzaldehyde (1.91 mL, 15.7 mmol, 1.2 equiv.) and pyrrolidine (0.11 mL, 1.3 mmol, 10 mol%) with stirring for 24 h, affording the title compound (4.73 g, 72%) as a colourless solid. **mp** (*n*-hexane/Et_2O) 114 – 116 °C; **^{1}H NMR** (400 MHz, $CDCl_3$) δ_H 8.94 (s, 1H, N*H*C), 7.97 (d, *J* = 9.0 Hz, 2H, Ar*H*), 7.87 (d, *J* = 9.0 Hz, 2H, *p*-OMeAr*H*), 7.48 (d, *J* = 9.0 Hz, 2H, Ar*H*), 7.16 (d, *J* = 8.0 Hz, 2H, *p*-Tol*H*), 7.11 (d, *J* = 8.0 Hz, 2H, *p*-Tol*H*), 6.97 (d, *J* = 9.0 Hz, 2H, *p*-OMeAr*H*), 6.72 (s, 1H, HetAr*H*), 3.87 (s, 3H, OC*H*$_3$), 2.36 (s, 3H, ArC*H*$_3$); **^{13}C NMR** (101 MHz, $CDCl_3$) δ_C 170.2, 165.7, 145.3, 144.1 (q, *J* = 38.5 Hz), 143.0, 139.8, 138.3, 134.0, 129.8, 128.9, 128.8, 125.7, 125.4, 125.0, 121.1 (q, *J* = 269.5 Hz) 114.9, 106.4 (q, *J* = 2.0 Hz), 55.8, 21.3; **^{19}F NMR** (377 MHz, $CDCl_3$) δ_F -62.38; **LRMS** (ESI, *m/z*) [M+H]$^+$ 500.0; **HRMS** (ESI, *m/z*) calculated for $(C_{25}H_{21}F_3N_3O_3S)^+$ 500.1250 [M+H]$^+$, found 500.1247; **IR** ($CHCl_3$, ν_{max}/cm^{-1}) 1593, 1561, 1513, 1472, 1426, 1372, 1322, 1267, 1237, 1157, 1091, 975, 838, 808, 774, 638; **R$_f$** 0.25 (*n*-hexane:EtOAc, 60:40).

Benzyl 4-chloro-2-((furan-2-ylmethyl)amino)-5-(*N*-(4-methoxybenzylidene)sulfamoyl)-benzoate (3b)

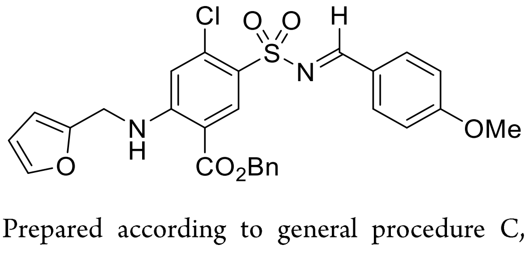

Prepared according to general procedure C, using sulfonamide (**9ab**) (421 mg, 1.0 mmol, 1.0 equiv.), 4-methoxybenzaldehyde (0.15 mL, 1.2 mmol, 1.2 equiv.) and pyrrolidine (9 µL, 0.1 mmol, 10 mol%) with stirring for 24 h, affording the title compound (373 mg, 69%) as a colourless solid. **mp** (*n*-hexane/Et_2O) 121 – 123 °C; **^{1}H NMR** (400 MHz, $CDCl_3$) δ_H 9.03 (s, 1H, N*H*C), 8.85 (s, 1H, Ar*H*), 8.72 (t, *J* = 5.5 Hz, 1H,

CH_2NHAr), 7.91 (d, *J* = 9.0 Hz, 2H, *p*-OMeAr*H*), 7.47 – 7.30 (m, 6H, Ph*H*, HetAr*H*), 6.98 (d, *J* = 9.0 Hz, 2H, *p*-OMeAr*H*), 6.83 (s, 1H, Ar*H*), 6.33 (dd, *J* = 3.5, 2.0 Hz, 1H, HetAr*H*), 6.26 (dd, *J* = 3.5, 1.0 Hz, 1H, Het*Ar*H), 5.34 (s, 2H, CO_2CH_2Ph), 4.42 (d, *J* = 5.5 Hz, 2H, $HetArCH_2Ar$), 3.89 (s, 3H, OCH_3); **^{13}C NMR** (101 MHz, $CDCl_3$) δ_C 171.2, 167.0, 165.5, 153.7, 150.2, 142.7, 138.8, 136.1, 135.6, 133.9, 128.8, 128.5, 128.3, 125.3, 121.7, 114.8, 113.7, 110.6, 108.8, 107.9, 66.9, 55.8, 40.3; **LRMS** (ESI, *m/z*) $[M+H]^+$ 539.2; **HRMS** (ESI, *m/z*) calculated for $(C_{27}H_{24}{}^{35}ClN_2O_6S)^+$ 539.1038 $[M+H]^+$, found 539.1036; **IR** ($CHCl_3$, ν_{max}/cm^{-1}) 3327, 1686, 1590, 1562, 1512, 1426, 1310, 1263, 1228, 1158, 1124, 873, 834, 806, 763; **R_f** 0.45 (*n*-hexane:EtOAc, 60:40).

3.5. Photochemical Functionalisation with Activated Alkenes

General Procedure D:

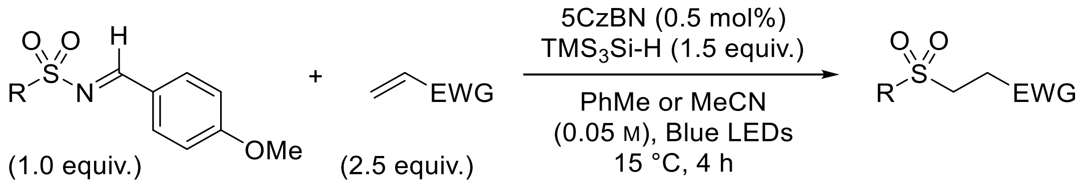

N-sulfonylimine (0.2 mmol, 1.0 equiv.) and 5CzBN (0.9 mg, 0.5 mol%) were added to an 8.0 mL screw capped vial equipped with a rubber septum. The vial was evacuated and back filled with nitrogen three times, followed by the addition of tris(trimethylsilyl)silane (93 µL, 0.3 mmol, 1.5 equiv.), the appropriate alkene (0.5 mmol, 2.5 equiv.) and toluene or acetonitrile (4 mL, 0.05 M). The vial was then placed in a glass tempering beaker connected to a flow of water, the vial was subsequently submerged in water and irradiated with two EvoluChem™ 450 nm LED spotlights. After 4 h the reaction was diluted with $CHCl_3$ (4 mL), and the solvent removed under reduced pressure, followed by purification by flash column chromatography.

4-Tosylbutan-2-one (2a)

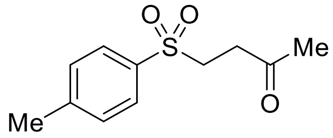

Prepared according to general procedure D, using *N*-sulfonylimine **1a** (58 mg, 0.2 mmol, 1.0 equiv.) in toluene (4 mL). Purification by flash column chromatography (*n*-hexane:EtOAc, 80:20 to 60:40), afforded the title compound (38 mg, 84%) as a brown solid. **mp** (*n*-hexane/EtOAc) 63 – 65 °C; **^{1}H NMR** (400 MHz, $CDCl_3$) δ_H 7.74 (d, *J* = 8.0 Hz, 2H, *p*-MeAr*H*), 7.33 (d, *J* = 8.0 Hz, 2H, *p*-MeAr*H*), 3.32 (t, *J* = 7.5 Hz, 2H, $SO_2CH_2CH_2$), 2.87 (t, *J* = 7.5 Hz, 2H, CH_2CH_2CO), 2.41 (s, 3H, $ArCH_3$), 2.13 (s, 3H $COCH_3$); **^{13}C NMR** (101 MHz, $CDCl_3$) δ_C 203.8, 145.0, 136.0, 130.0, 128.0, 50.6, 36.0, 29.9, 21.6; **LRMS** (ESI, *m/z*) $[M+Na]^+$ 249.0. The spectroscopic data is in agreement with literature.[31]

The reaction scale up was performed using a modified general procedure D, using *N*-sulfonylimine **1a** (289 mg, 1.0 mmol, 1.0 equiv.), 5CzBN (4.6 mg, 0.5 mol%), tris(trimethylsilyl)silane (0.42 mL, 1.5 mmol, 1.5 equiv.), methyl vinyl ketone (0.21 mL, 2.5 mmol, 2.5 equiv.) and toluene (20 mL), using a round bottom flask in place of a vial. Purification by flash column chromatography (*n*-hexane:EtOAc, 80:20 to 60:40), afforded the title compound (206 mg, 91%) as a brown solid.

4-(Phenylsulfonyl)butan-2-one (2b)

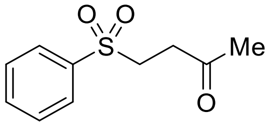

Prepared according to general procedure D, using *N*-sulfonylimine **1ab** (55 mg, 0.2 mmol, 1.0 equiv.) in tolune (4 mL). Purification by flash column chromatography (*n*-hexane:EtOAc, 80:20 to 60:40), afforded the title compound (43 mg, 100%) as an orange solid. **mp** (*n*-hexane/EtOAc) 68 – 70 °C; **^{1}H NMR** (400 MHz, $CDCl_3$) δ_H 7.93 – 7.87 (m, 2H, Ph*H*), 7.70 – 7.63 (m, 1H, Ph*H*), 7.61 – 7.53 (m, 2H, Ph*H*), 3.37 (t, *J* = 7.5 Hz, 2H, $SO_2CH_2CH_2$), 2.92 (t, *J* = 7.5 Hz, 2H, CH_2CH_2CO), 2.17 (s, 3H, $COCH_3$); **^{13}C NMR** (101 MHz, $CDCl_3$) δ_C 203.8, 139.1, 134.1, 129.5, 128.1, 50.7, 36.0, 30.0; **LRMS** (ESI, *m/z*) $[M+Na]^+$ 255.1. The spectroscopic data is in agreement with literature.[32]

4-(Naphthalen-2-ylsulfonyl)butan-2-one (2c)

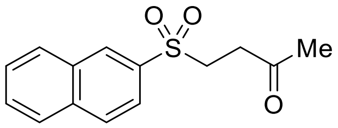

Prepared according to general procedure D, using *N*-sulfonylimine **1ac** (65 mg, 0.2 mmol, 1.0 equiv.) in toluene (4 mL). Purification by flash column chromatography (*n*-hexane:EtOAc, 80:20 to 60:40), afforded the title compound (38 mg, 73%) as an off-white solid. **mp** (*n*-hexane /EtOAc) 114 – 116 °C; **^{1}H NMR** (400 MHz, $CDCl_3$) δ_H 8.48 (d, *J* = 2.0 Hz, 1H, Ar*H*), 8.05 – 7.97 (m, 2H, Ar*H*), 7.94 (dd, *J* = 8.0, 1.5 Hz, 1H, Ar*H*), 7.86 (dd, *J* = 8.5, 2.0 Hz, 1H, Ar*H*), 7.69 (ddd, *J* = 8.0, 7.0, 1.5 Hz, 1H, Ar*H*), 7.64 (ddd, *J* = 8.0, 7.0, 1.5 Hz, 1H, Ar*H*), 3.46 (t, *J* = 7.5 Hz, 2H, $SO_2CH_2CH_2$), 2.95 (t, *J* = 7.5 Hz, 2H, CH_2CH_2CO), 2.16 (s, 3H, $COCH_3$); **^{13}C NMR** (101 MHz, $CDCl_3$) δ_C 203.8, 135.9, 135.5, 132.3, 130.0 (2C), 129.9, 129.6, 128.2, 128.0, 122.6, 50.7, 36.1, 30.0; **LRMS** (ESI, *m/z*) $[M+Na]^+$ 285.1; **HRMS** (ESI, *m/z*) calculated for $(C_{14}H_{15}O_3S)^+$ 263.0736 $[M+H]^+$, found 263.0738; **IR** ($CHCl_3$, ν_{max}/cm^{-1}) 1719, 1347, 1309, 1245, 1149, 1125, 1074, 820, 752, 638; **R_f** 0.22 (*n*-hexane:EtOAc, 60:40).

4-(Mesitylsulfonyl)butan-2-one (2d)

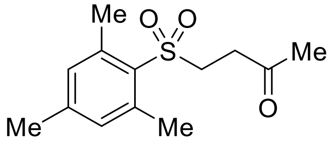

Prepared according to general procedure D, using *N*-sulfonylimine **1ad** (64 mg, 0.2 mmol, 1.0 equiv.) in toluene (4 mL). Purification by flash column chromatography (*n*-hexane:EtOAc, 80:20 to 60:40), afforded the title compound (16 mg, 31%) as an orange solid. **mp** (*n*-hexane/EtOAc) 53 – 55 °C; **^{1}H NMR** (400 MHz, $CDCl_3$) δ_H 6.97 (s, 2H, Ar*H*), 3.36 (t, *J* = 7.5 Hz, 2H, $SO_2CH_2CH_2$), 2.97 (t, *J* = 7.5, Hz, 2H, CH_2CH_2CO), 2.64 (s, 6H, ArCH_3), 2.31 (s, 3H, ArCH_3), 2.20 (s, 3H, $COCH_3$); **^{13}C NMR** (101 MHz, $CDCl_3$) δ_C 204.3, 143.7, 140.2, 133.0, 132.5, 50.4, 35.2, 30.1, 23.0, 21.1; **LRMS** (ESI, *m/z*) $[M+Na]^+$ 277.1; **HRMS** (ESI, *m/z*) calculated for $(C_{13}H_{18}NaO_3S)^+$ 277.0869 $[M+Na]^+$, found 277.0869; **IR** ($CHCl_3$, ν_{max}/cm^{-1}) 1720, 1603, 1452, 1416, 1309, 1246, 1146, 838, 771, 657; **R_f** 0.37 (*n*-hexane:EtOAc, 60:40).

4-((4-Fluorophenyl)sulfonyl)butan-2-one (2e)

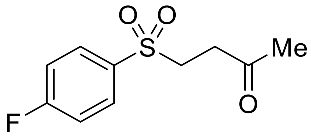

Prepared according to general procedure D, using *N*-sulfonylimine **1ae** (59 mg, 0.2 mmol, 1.0 equiv.) in toluene (4 mL). Purification by flash column chromatography (*n*-hexane:EtOAc, 80:20 to 60:40), afforded the title compound (39 mg, 85%) as an off-white solid. **mp** (*n*-hexane/EtOAc) 76 – 78 °C; **^{1}H NMR** (400 MHz, $CDCl_3$) δ_H 7.97 – 7.86 (m, 2H, *p*-FAr*H*), 7.29 – 7.19 (m, 2H, *p*-FAr*H*), 3.36 (t, *J* = 7.5 Hz, 2H, $SO_2CH_2CH_2$), 2.93 (t, *J* = 7.5 Hz, 2H, CH_2CH_2CO), 2.18 (s, 3H, $COCH_3$); **^{13}C NMR** (101 MHz, $CDCl_3$) δ_C 203.7, 166.1 (d, *J* = 257.0 Hz), 135.2 (d, *J* = 3.5 Hz), 131.0 (d, *J* = 9.5 Hz), 116.9 (d, *J* = 22.5 Hz), 50.8, 35.9, 30.0; **^{19}F NMR** (377 MHz, $CDCl_3$) δ_F -102.98; **LRMS** (ESI, *m/z*) $[M+Na]^+$ 253.0; **HRMS** (ESI, *m/z*) calculated for $(C_{10}H_{11}FNaO_3S)^+$ 253.0305 $[M+Na]^+$, found 253.0306; **IR** ($CHCl_3$, ν_{max}/cm^{-1}) 1720, 1591, 1494, 1407, 1317, 1291, 1236, 1148, 1087, 842, 772; **R_f** 0.19 (*n*-hexane:EtOAc, 60:40).

4-((4-(Trifluoromethyl)phenyl)sulfonyl)butan-2-one (2f)

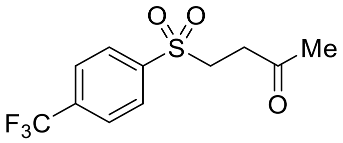

Prepared according to general procedure D, using *N*-sulfonylimine **1af** (69 mg, 0.2 mmol, 1.0 equiv.) in toluene (4 mL). Purification by flash column chromatography (*n*-hexane:EtOAc, 80:20 to 60:40), afforded the title compound (33 mg, 58%) as an off-white solid. **mp** (*n*-hexane/EtOAc) 115 – 117 °C; **^{1}H NMR** (400 MHz, $CDCl_3$) δ_H 8.05 (d, *J* = 8.0 Hz, 2H, *p*-CF_3Ar*H*), 7.85 (d, *J* = 8.0 Hz, 2H, *p*-CF_3Ar*H*), 3.41 (t, *J* = 7.5 Hz, 2H, $SO_2CH_2CH_2$), 2.95 (t, *J* = 7.5 Hz, 2H, CH_2CH_2CO), 2.20 (s, 3H, $COCH_3$); **^{13}C NMR** (101 MHz, $CDCl_3$) δ_C 203.4, 142.7, 135.8 (q, *J* = 33.5 Hz), 128.8, 126.7 (q, *J* = 3.5 Hz), 123.2 (q, *J* = 273.5 Hz), 50.6, 35.7, 30.0; **LRMS** (ESI, *m/z*) [M+Na]$^+$ 303.1; **HRMS** (ESI, *m/z*) calculated for $(C_{11}H_{11}F_3NaO_3S)^+$ 303.0273 [M+Na]$^+$, found 303.0275; **IR** ($CHCl_3$, ν_{max}/cm^{-1}) 1714, 1408, 1328, 1269, 1246, 1149, 1127, 1107, 1091, 1065, 1021, 842, 790, 765, 712, 620; **R$_f$** 0.46 (*n*-hexane:EtOAc, 60:40).

4-((3-Oxobutyl)sulfonyl)benzonitrile (2g)

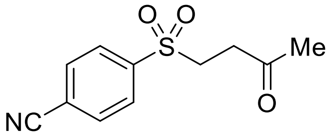

Prepared according to general procedure D, using *N*-sulfonylimine **1ag** (60 mg, 0.2 mmol, 1.0 equiv.) in toluene (4 mL). Purification by flash column chromatography (*n*-hexane:EtOAc, 80:20 to 50:50), afforded the title compound (33 mg, 70%) as an off-white solid. **mp** (*n*-hexane/EtOAc) 118 – 120 °C; **^{1}H NMR** (400 MHz, $CDCl_3$) δ_H 8.03 (d, *J* = 8.5 Hz, 2H, *p*-CNAr*H*), 7.88 (d, *J* = 8.5 Hz, 2H, *p*-CNAr*H*), 3.40 (t, *J* = 7.5 Hz, 2H, $SO_2CH_2CH_2$), 2.95 (t, *J* = 7.5 Hz, 2H, CH_2CH_2CO), 2.19 (s, 3H, $COCH_3$); **^{13}C NMR** (101 MHz, $CDCl_3$) δ_C 203.3, 143.4, 133.3, 128.9, 117.9, 117.1, 50.6, 35.6, 30.0; **LRMS** (ESI, *m/z*) [M+Na]$^+$ 260.0; **HRMS** (ESI, *m/z*) calculated for $(C_{11}H_{11}NNaO_3S)^+$ 260.0352 [M+Na]$^+$, found 260.0353; **IR** ($CHCl_3$, ν_{max}/cm^{-1}) 1709, 1409, 1365, 1326, 1269, 1245, 1168, 1145, 1085, 1022, 841, 802, 761, 642; **R$_f$** 0.13 (*n*-hexane:EtOAc, 60:40).

4-((4-Acetylphenyl)sulfonyl)butan-2-one (2h)

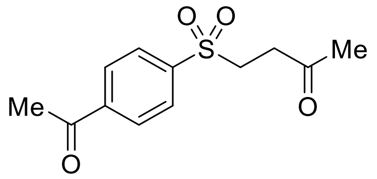

Prepared according to general procedure D, using *N*-sulfonylimine **1ah** (64 mg, 0.2 mmol, 1.0 equiv.) in toluene (4 mL). Purification by flash column chromatography (*n*-hexane:EtOAc, 80:20 to 50:50), afforded the title compound (37 mg, 73%) as a colourless solid. **mp** (*n*-hexane/EtOAc) 110 – 112 °C; **^{1}H NMR** (400 MHz, $CDCl_3$) δ_H 8.12 (d, *J* = 8.5 Hz, 2H, Ar*H*), 8.01 (d, *J* = 8.5 Hz, 2H, Ar*H*), 3.40 (t, *J* = 7.5 Hz, 2H, $SO_2CH_2CH_2$), 2.94 (t, *J* = 7.5 Hz, 2H, CH_2CH_2CO), 2.66 (s, 3H, $COCH_3$), 2.19 (s, 3H, $COCH_3$); **^{13}C NMR** (101 MHz, $CDCl_3$) δ_C 203.5, 196.7, 142.9, 141.2, 129.3, 128.5, 50.6, 35.8, 30.0, 27.1; **LRMS** (ESI, *m/z*) $[M+Na]^+$ 277.1; **HRMS** (ESI, *m/z*) calculated for $(C_{12}H_{14}NaO_4S)^+$ 277.0505 $[M+Na]^+$, found 277.0506; **IR** ($CHCl_3$, ν_{max}/cm^{-1}) 1719, 1690, 1398, 1361, 1317, 1297, 1262, 1152, 1014, 732, 641; **R_f** 0.14 (*n*-hexane:EtOAc, 60:40).

Methyl 3-((3-oxobutyl)sulfonyl)benzoate (2i)

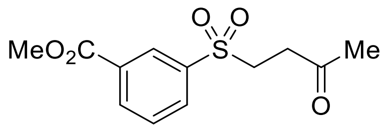

Prepared according to general procedure D, using *N*-sulfonylimine **1ai** (67 mg, 0.2 mmol, 1.0 equiv.) in toluene (4 mL). Purification by flash column chromatography (*n*-hexane:EtOAc, 80:20 to 50:50), afforded the title compound (40 mg, 74%) as an off-white solid. **mp** (*n*-hexane/EtOAc) 65 – 67 °C; **^{1}H NMR** (400 MHz, $CDCl_3$) δ_H 8.56 (app. td, *J* = 1.5, 0.5 Hz, 1 Hz, Ar*H*), 8.33 (app. dt, *J* = 8.0, 1.5 Hz, 1H, Ar*H*), 8.09 (ddd, *J* = 8.0, 2.0, 1.5 Hz, 1H, Ar*H*), 7.68 (app. td, *J* = 8.0, 0.5 Hz, 1H, Ar*H*), 3.97 (s, 3H, CO_2CH_3), 3.41 (t, *J* = 7.5 Hz, 2H, $SO_2CH_2CH_2$), 2.95 (t, *J* = 7.5 Hz, 2H, CH_2CH_2CO), 2.19 (s, 3H, $COCH_3$); **^{13}C NMR** (101 MHz, $CDCl_3$) δ_C 203.6, 165.3, 139.9, 135.0, 132.1, 131.9, 129.9, 129.3, 52.8, 50.7, 35.8, 30.0; **LRMS** (ESI, *m/z*) $[M+Na]^+$ 293.1; **HRMS** (ESI, *m/z*) calculated for $(C_{12}H_{14}NaO_5S)^+$ 293.0454 $[M+Na]^+$, found 293.0454; **IR** ($CHCl_3$, ν_{max}/cm^{-1}) 1723, 1439, 1304, 1270, 1151, 1122, 752; **R_f** 0.18 (*n*-hexane:EtOAc, 60:40).

4-((4-Bromophenyl)sulfonyl)butan-2-one (2j)

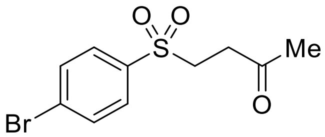

Prepared according to general procedure D, using *N*-sulfonylimine **1aj** (71 mg, 0.2 mmol, 1.0 equiv.) in toluene (4 mL). Purification by flash column chromatography (*n*-hexane:EtOAc, 80:20 to 60:40), afforded the title compound (50 mg, 85%) as an off-white solid. **mp** (*n*-hexane/EtOAc) 99 – 101 °C; **^{1}H NMR** (400 MHz, $CDCl_3$) δ_H 7.76 (d, *J* = 9.0 Hz, 2H, *p*-BrAr*H*), 7.72 (d, *J* = 9.0 Hz, 2H, *p*-BrAr*H*), 3.37 (t, *J* = 7.5 Hz, 2H, $SO_2CH_2CH_2$), 2.93 (t, *J* = 7.5 Hz, 2H, CH_2CH_2CO), 2.18 (s, 3H, $COCH_3$); **^{13}C NMR** (101 MHz, $CDCl_3$) δ_C 203.6, 138.2, 132.9, 129.7, 128.1, 50.7, 35.9, 30.0; **LRMS** (ESI, *m/z*) [^{79}BrM+Na]$^+$ 313.0, [^{81}BrM+Na]$^+$ 315.0; **HRMS** (ESI, *m/z*) calculated for $(C_{10}H_{11}{}^{79}BrNaO_3S)^+$ 312.9504 [^{79}BrM+Na]$^+$, found 312.9506, calculated for $(C_{10}H_{11}{}^{81}BrNaO_3S)^+$ 312.9483 [^{81}BrM+Na]$^+$, found 312.9484; **IR** ($CHCl_3$, ν_{max}/cm^{-1}) 1719, 1575, 1390, 1363, 1315, 1275, 1244, 1150, 1086, 1067, 1010, 836, 774; **R$_f$** 0.24 (*n*-hexane:EtOAc, 60:40).

4-((3-Bromophenyl)sulfonyl)butan-2-one (2k)

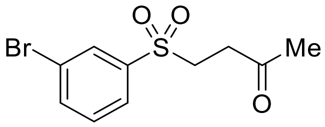

Prepared according to general procedure D, using *N*-sulfonylimine **1ak** (71 mg, 0.2 mmol, 1.0 equiv.) in toluene (4 mL). Purification by flash column chromatography (*n*-hexane:EtOAc, 80:20 to 60:40), afforded the title compound (18 mg, 30%) as a colourless solid. **mp** (*n*-hexane/EtOAc) 51 – 53 °C; **^{1}H NMR** (400 MHz, $CDCl_3$) δ_H 8.05 (app. t, *J* = 2.0 Hz, 1H, Ar*H*), 7.84 (ddd, *J* = 8.0, 2.0, 1.0 Hz, 1H, Ar*H*), 7.80 (ddd, *J* = 8.0, 2.0, 1.0 Hz, 1H, Ar*H*), 7.48 (app. t, *J* = 8.0 Hz, 1H, Ar*H*), 3.39 (t, *J* = 7.5 Hz, 2H, $SO_2CH_2CH_2$), 2.95 (t, *J* = 7.5 Hz, 2H, CH_2CH_2CO), 2.20 (s, 3H, $COCH_3$); **^{13}C NMR** (101 MHz, $CDCl_3$) δ_C 203.5, 141.1, 137.2, 131.1, 131.1, 126.7, 123.6, 50.8, 35.8, 30.0; **LRMS** (ESI, *m/z*) [^{79}BrM+Na]$^+$ 313.0, [^{81}BrM+Na]$^+$ 315.0; **HRMS** (ESI, *m/z*) calculated for $(C_{10}H_{11}{}^{79}BrNaO_3S)^+$ 312.9504 [^{79}BrM+Na]$^+$, found 312.9506, calculated for $(C_{10}H_{11}{}^{81}BrNaO_3S)^+$ 314.9485 [^{81}BrM+Na]$^+$, found 314.9483; **IR** ($CHCl_3$, ν_{max}/cm^{-1}) 2928, 1721, 1462, 1413, 1364, 1317, 1295, 1242, 1152, 1099, 782; **R$_f$** 0.45 (*n*-hexane:EtOAc, 60:40).

4-((2-Bromophenyl)sulfonyl)butan-2-one (2l)

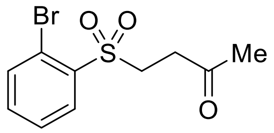

Prepared according to general procedure D, using *N*-sulfonylimine **1al** (71 mg, 0.2 mmol, 1.0 equiv.) in toluene (4 mL). Purification by flash column chromatography (*n*-hexane:EtOAc, 80:20 to 60:40), afforded the title compound (12 mg, 20%) as a colourless solid. **mp** (*n*-hexane/EtOAc) 68 – 70 °C; **^{1}H NMR** (400 MHz, $CDCl_3$) δ_H 7.97 – 7.88 (m, 2H, *o*-BrAr*H*), 7.72 – 7.63 (m, 1H, BrAr*H*), 7.63 – 7.52 (m, 2H, *o*-BrAr*H*), 3.38 (t, *J* = 7.5 Hz, 2H, $SO_2CH_2CH_2$), 2.94 (t, *J* = 7.5 Hz, 2H, CH_2CH_2CO), 2.18 (s, 3H, $COCH_3$); **^{13}C NMR** (101 MHz, $CDCl_3$) δ_C 203.8, 139.2, 134.1, 129.6 (2C), 128.1 (2C), 50.7, 36.0, 30.0; **LRMS** (ESI, *m/z*) $[^{79}BrM+K]^+$ 329.0, $[^{81}BrM+K]^+$ 331.0; **HRMS** (ESI, *m/z*) calculated for $(C_{10}H_{11}{}^{79}BrNaO_3S)^+$ 312.9504 $[^{79}BrM+Na]^+$, found 312.9505, calculated for $(C_{10}H_{11}{}^{81}BrNaO_3S)^+$ 314.9485 $[^{81}BrM+Na]^+$, found 314.9485; **IR** ($CHCl_3$, ν_{max}/cm^{-1}) 1720, 1447, 1416, 1364, 1307, 1150, 1087, 753, 690, 619; **R_f** 0.29 (*n*-hexane:EtOAc, 60:40).

4-((4-Methoxyphenyl)sulfonyl)butan-2-one (2m)

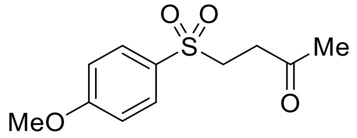

Prepared according to general procedure D, using *N*-sulfonylimine **1am** (61 mg, 0.2 mmol, 1.0 equiv.) in toluene (4 mL). Purification by flash column chromatography (*n*-hexane:EtOAc, 80:20 to 60:40), afforded the title compound (38 mg, 78%) as a yellow oil. **^{1}H NMR** (400 MHz, $CDCl_3$) δ_H 7.81 (d, *J* = 9.0 Hz, 2H, *p*-OMeAr*H*), 7.01 (d, *J* = 9.0 Hz, 2H, *p*-OMeAr*H*), 3.88 (s, 3H, OCH_3), 3.33 (t, *J* = 7.5 Hz, 2H, $SO_2CH_2CH_2$), 2.90 (t, *J* = 7.5 Hz, 2H, CH_2CH_2CO), 2.17 (s, 3H, $COCH_3$); **^{13}C NMR** (101 MHz, $CDCl_3$) δ_C 204.0, 164.0, 130.6, 130.3, 114.7, 55.8, 51.0, 36.2, 30.0; **LRMS** (ESI, *m/z*) $[M+Na]^+$ 265.1; **HRMS** (ESI, *m/z*) calculated for $(C_{11}H_{15}O_4S)^+$ 243.0686 $[M+H]^+$, found 243.0688; **IR** ($CHCl_3$, ν_{max}/cm^{-1}) 1719, 1596, 1499, 1317, 1298, 1262, 1144, 1089, 1023, 838, 805, 770, 733, 704, 667; **R_f** 0.13 (*n*-hexane:EtOAc, 60:40).

4-((2,4-Dimethoxyphenyl)sulfonyl)butan-2-one (2n)

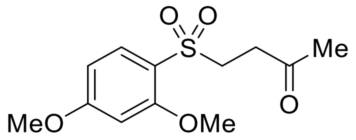

Prepared according to general procedure D, using *N*-sulfonylimine **1an** (67 mg, 0.2 mmol, 1.0 equiv.) in toluene (4 mL). Purification by flash column chromatography (*n*-hexane:EtOAc, 80:20 to 60:40), afforded the title compound (37 mg, 67%) as a yellow oil. **^{1}H NMR** (400 MHz, $CDCl_3$) δ_H 7.84 (d, *J* = 9.0 Hz, 1H, Ar*H*), 6.56 (dd, *J* = 9.0, 2.5 Hz, 1H, Ar*H*), 6.51 (d, *J* = 2.5 Hz, 1H, Ar*H*), 3.94 (s, 3H, OCH_3), 3.86 (s, 3H, OCH_3), 3.56 (t, *J* = 7.5 Hz, 2H, $SO_2CH_2CH_2$), 2.89 (t, *J* = 7.5 Hz, 2H, CH_2CH_2CO), 2.15 (s, 3H, $COCH_3$); **^{13}C NMR** (101 MHz, $CDCl_3$) δ_C 204.4, 165.9, 159.2, 132.4, 119.0, 104.9, 99.6, 56.4, 55.9, 49.0, 36.1, 30.1; **LRMS** (ESI, *m/z*) $[M+Na]^+$ 295.1; **HRMS** (ESI, *m/z*) calculated for $(C_{12}H_{16}NaO_5S)^+$ 295.0611 $[M+Na]^+$, found 295.0612; **IR** ($CHCl_3$, ν_{max}/cm^{-1}) 1719, 1597, 1576, 1468, 1439, 1416, 1364, 1302, 1214, 1165, 1126, 1073, 1022, 838, 773; **R$_f$** 0.38 (*n*-hexane:EtOAc, 60:40).

4-(Methylsulfonyl)butan-2-one (2o)

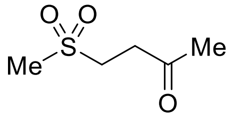

Prepared according to general procedure D, using *N*-sulfonylimine **1ao** (43 mg, 0.2 mmol, 1.0 equiv.) in toluene (4 mL). Purification by flash column chromatography (*n*-hexane:EtOAc, 80:20 to 60:40), afforded the title compound (20 mg, 67%) as a colourless solid. **mp** (*n*-hexane/EtOAc) 88 – 90 °C; **^{1}H NMR** (400 MHz, $CDCl_3$) δ_H 3.31 (td, *J* = 7.0, 0.5 Hz, 2H, $SO_2CH_2CH_2$), 3.02 (t, *J* = 7.0 Hz, 2H, CH_2CH_2CO), 2.93 (t, *J* = 0.5 Hz, 3H, SO_2CH_3), 2.24 (s, 3H, $COCH_3$); **^{13}C NMR** (101 MHz, $CDCl_3$) δ_C 204.1, 49.0, 41.8, 35.7, 30.0; **LRMS** (ESI, *m/z*) $[M+Na]^+$ 272.9; **HRMS** (ESI, *m/z*) calculated for $(C_5H_{10}NaO_3S)^+$ 173.0243 $[M+Na]^+$, found 173.0245; **IR** ($CHCl_3$, ν_{max}/cm^{-1}) 1716, 1291, 1261, 1120, 774; **R$_f$** 0.24 (*n*-hexane:EtOAc, 80:20).

4-(Propylsulfonyl)butan-2-one (2p)

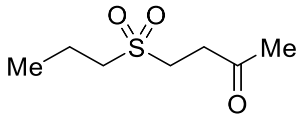

Prepared according to general procedure D, using *N*-sulfonylimine **1ap** (48 mg, 0.2 mmol, 1.0 equiv.) in toluene (4 mL). Purification by flash column chromatography (*n*-hexane:EtOAc, 80:20 to 60:40), afforded the title compound (25 mg, 69%) as an off-white solid. **mp** (*n*-hexane/EtOAc) 53 – 55 °C; **^{1}H NMR** (400 MHz, $CDCl_3$) δ 3.24 (t, *J* = 7.5 Hz, 2H, $SO_2CH_2CH_2CO$), 3.01 (t, *J* = 7.5 Hz, 2H, CH_2CH_2CO), 2.99 – 2.92 (m, 2H,

$SO_2CH_2CH_2CH_3$), 2.24 (s, 3H, $COCH_3$), 1.96 – 1.82 (m, 2H, $CH_2CH_2CH_3$), 1.09 (t, *J* = 7.5 Hz, 3H, CH_2CH_3); **^{13}C NMR** (101 MHz, $CDCl_3$) δ_C 204.3, 55.7, 47.0, 35.2, 30.0, 16.0, 13.3; **LRMS** (ESI, *m/z*) $[M+Na]^+$ 201.1. The spectroscopic data is in agreement with literature.[33]

4-(Cyclohexylsulfonyl)butan-2-one (2q)

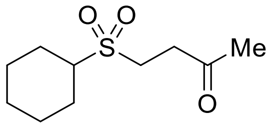

Prepared according to general procedure D, using *N*-sulfonylimine **1aq** (56 mg, 0.2 mmol, 1.0 equiv.) in toluene (4 mL). Purification by flash column chromatography (*n*-hexane:EtOAc, 80:20 to 60:40), afforded the title compound (31 mg, 70%) as a colourless solid. **mp** (*n*-hexane/EtOAc) 68 – 70 °C; **^{1}H NMR** (400 MHz, $CDCl_3$) δ_H 3.18 (t, *J* = 7.5 Hz, 2H, $SO_2CH_2CH_2$), 3.00 (t, *J* = 7.5 Hz, 2H, CH_2CH_2CO), 2.83 (tt, *J* = 12.5, 3.5 Hz, 1H, Cy*H*), 2.24 (s, 3H, $COCH_3$), 2.22 – 2.14 (m, 2H, Cy*H*), 1.99 – 1.87 (m, 2H, Cy*H*), 1.73 (dtt, *J* = 11.0, 3.5, 1.5 Hz, 1H, Cy*H*), 1.53 (app. qd, *J* = 12.5, 3.5 Hz, 2H, Cy*H*), 1.37 – 1.14 (m, 3H, Cy*H*); **^{13}C NMR** (101 MHz, $CDCl_3$) δ_C 204.5, 62.0, 43.6, 34.5, 30.1, 26.7, 25.3, 25.2 (2C); **LRMS** (ESI, *m/z*) $[M+Na]^+$ 241.1; **HRMS** (ESI, *m/z*) calculated for $(C_{10}H_{18}NaO_3S)^+$ 241.0869 $[M+Na]^+$, found 241.0870; **IR** ($CHCl_3$, ν_{max}/cm^{-1}) 2938, 2859, 1719, 1453, 1415, 1362, 1301, 1263, 1124, 612; **R_f** 0.26 (*n*-hexane:EtOAc, 60:40).

4-(*Tert*-butylsulfonyl)butan-2-one (2r)

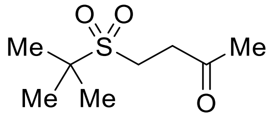

Prepared according to general procedure D, using *N*-sulfonylimine **1ar** (51 mg, 0.2 mmol, 1.0 equiv.) in toluene (4 mL). Purification by flash column chromatography (*n*-hexane:EtOAc, 80:20 to 60:40), afforded the title compound (11 mg, 29%) as an orange oil. **^{1}H NMR** (400 MHz, $CDCl_3$) δ_H 3.21 (t, *J* = 7.5 Hz, 2H, $SO_2CH_2CH_2$), 3.03 (t, *J* = 7.5 Hz, 2H, CH_2CH_2CO), 2.25 (s, 3H, $COCH_3$), 1.43 (s, 9H, $C(CH_3)_3$); **^{13}C NMR** (101 MHz, $CDCl_3$) δ_C 204.8, 59.2, 41.1, 40.2, 34.1, 23.5; **LRMS** (ESI, *m/z*) $[M+Na]^+$ 215.1; **HRMS** (ESI, *m/z*) calculated for $(C_8H_{17}O_3S)^+$ 193.0893 $[M+H]^+$, found 193.0896; **IR** ($CHCl_3$, ν_{max}/cm^{-1}) 1720, 1298, 1268, 1114, 756; **R_f** 0.14 (*n*-hexane:EtOAc, 60:40).

4-(Cyclopropylsulfonyl)butan-2-one (2s)

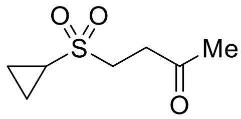

Prepared according to general procedure D, using *N*-sulfonylimine **1as** (48 mg, 0.2 mmol, 1.0 equiv.) in toluene (4 mL). Purification by flash column chromatography (*n*-hexane:EtOAc, 80:20 to 60:40), afforded the title compound (22 mg, 62%) as a brown oil. **^{1}H NMR** (400 MHz, $CDCl_3$) δ_H 3.34 (t, *J* = 7.5 Hz, 2H, $SO_2CH_2CH_2$), 3.02 (t, *J* = 7.5 Hz, 2H, CH_2CH_2CO), 2.40 (tt, *J* = 8.0, 4.5 Hz, 1H, $SO_2CH(CH_2)_2$), 2.24 (s, 3H, $COCH_3$), 1.31 – 1.15 (m, 2H, $CH(CH_AH_B)_2$), 1.12 – 0.98 (m, 2H, $CH(CH_AH_B)_2$); **^{13}C NMR** (101 MHz, $CDCl_3$) δ_C 204.2, 48.1, 35.5, 30.1, 30.0, 5.0; **LRMS** (ESI, *m/z*) $[M+Na]^+$ 199.1; **HRMS** (ESI, *m/z*) calculated for $(C_7H_{12}NaO_3S)^+$ 199.0399 $[M+Na]^+$, found 199.0402; **IR** ($CHCl_3$, ν_{max}/cm^{-1}) 1719, 1420, 1317, 1296, 1125, 1044, 889, 834; **R$_f$** 0.16 (*n*-hexane:EtOAc, 60:40).

4-(Thiophen-2-ylsulfonyl)butan-2-one (2t)

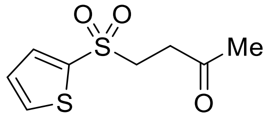

Prepared according to general procedure D, using *N*-sulfonylimine **1at** (56 mg, 0.2 mmol, 1.0 equiv.) in toluene (4 mL). Purification by flash column chromatography (*n*-hexane:EtOAc, 80:20 to 60:40), afforded the title compound (34 mg, 78%) as a brown oil. **^{1}H NMR** (400 MHz, $CDCl_3$) δ_H 7.73 (dd, *J* = 5.0, 1.5 Hz, 1H, HetAr*H*), 7.69 (dd, *J* = 4.0, 1.5 Hz, 1H, HetAr*H*), 7.17 (dd, *J* = 5.0, 4.0 Hz, 1H, HetAr*H*), 3.49 (t, *J* = 7.5 Hz, 2H, $SO_2CH_2CH_2$), 2.97 (t, *J* = 7.5 Hz, 2H, CH_2CH_2CO), 2.19 (s, 3H, $COCH_3$); **^{13}C NMR** (101 MHz, $CDCl_3$) δ_C 203.6, 140.0, 134.4, 134.4, 128.2, 52.2, 36.5, 30.0; **LRMS** (ESI, *m/z*) $[M+Na]^+$ 241.0; **HRMS** (ESI, *m/z*) calculated for $(C_8H_{10}NaO_3S_2)^+$ 240.9964 $[M+Na]^+$, found 240.9964; **IR** ($CHCl_3$, ν_{max}/cm^{-1}) 1719, 1507, 1403, 1344, 1314, 1228, 1133, 1091, 1069, 1017, 856, 772, 730, 666; **R$_f$** 0.19 (*n*-hexane:EtOAc, 60:40).

4-(Pyridin-3-ylsulfonyl)butan-2-one (2u)

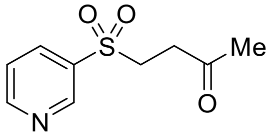

Prepared according to general procedure D, using *N*-sulfonylimine **1au** (55 mg, 0.2 mmol, 1.0 equiv.) in toluene (4 mL). Purification by flash column chromatography (*n*-hexane:EtOAc, 80:20 to 20:80), afforded the title compound (34 mg, 80%) as a brown solid. **mp** (*n*-hexane/EtOAc) 42 – 44 °C; **^{1}H NMR** (400 MHz,

$CDCl_3$) δ_H 9.11 (dd, *J* = 2.5, 1.0 Hz, 1H, Py*H*), 8.90 (dd, *J* = 5.0, 1.5 Hz, 1H, Py*H*), 8.19 (ddd, *J* = 8.0, 2.5, 1.5 Hz, 1H, Py*H*), 7.53 (ddd, *J* = 8.0, 5.0, 1.0 Hz, 1H, Py*H*), 3.43 (t, *J* = 7.5 Hz, 2H, $SO_2CH_2CH_2$), 2.97 (t, *J* = 7.5 Hz, 2H, CH_2CH_2CO), 2.19 (s, 3H, $COCH_3$); **^{13}C NMR** (101 MHz, $CDCl_3$) δ_C 203.4, 154.7, 149.2, 135.9, 135.7, 124.1, 51.1, 35.7, 30.0; **LRMS** (ESI, *m/z*) $[M+H]^+$ 214.1; **HRMS** (ESI, *m/z*) calculated for $(C_9H_{11}NNaO_3S)^+$ 236.0352 $[M+Na]^+$, found 236.0354; **IR** ($CHCl_3$, ν_{max}/cm^{-1}) 1719, 1575, 1417, 1313, 1121, 1103, 967, 839, 777, 703, 622; **R$_f$** 0.33 (EtOAc).

4-((5-Methylpyridin-2-yl)sulfonyl)butan-2-one (2v)

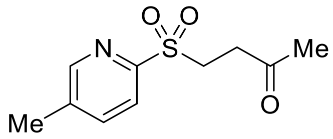

Prepared according to general procedure D, using *N*-sulfonylimine **1av** (58 mg, 0.2 mmol, 1.0 equiv.) in toluene (4 mL). Purification by flash column chromatography (*n*-hexane:EtOAc, 80:20 to 50:50), afforded the title compound (58 mg, 69%) as an off-white solid. **mp** (*n*-hexane/EtOAc) 130 – 132 °C; **^{1}H NMR** (400 MHz, $CDCl_3$) δ_H 8.54 (dt, *J* = 2.5, 1.0 Hz, 1H, HetAr*H*), 7.95 (dd, *J* = 8.0, 1.0 Hz, 1H, HetAr*H*), 7.74 (ddq, *J* = 8.0, 2.5, 1.0 Hz, 1H, HetAr*H*), 3.61 (t, *J* = 7.5 Hz, 2H, $SO_2CH_2CH_2$), 3.00 (t, *J* = 7.5 Hz, 2H, CH_2CH_2CO), 2.45 (d, *J* = 1.0 Hz, 3H, $ArCH_3$), 2.18 (s, 3H, $COCH_3$); **^{13}C NMR** (101 MHz, $CDCl_3$) δ_C 203.9, 154.6, 150.9, 138.5 (2C), 121.9, 47.1, 36.0, 30.0, 18.7; **LRMS** (ESI, *m/z*) $[M+H]^+$ 228.1, $[M+Na]^+$ 250.1; **HRMS** (ESI, *m/z*) calculated for $(C_{10}H_{13}NNaO_3S)^+$ 250.0508 $[M+Na]^+$, found 250.0508; **IR** ($CHCl_3$, ν_{max}/cm^{-1}) 1719, 1311, 1159, 1100, 1027, 902, 839, 770, 664; **R$_f$** 0.32 (*n*-hexane:EtOAc, 20:80).

4-((4-Phenylpiperidin-1-yl)sulfonyl)butan-2-one (2w)

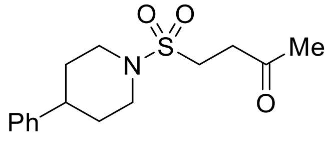

Prepared according to general procedure D, using *N*-sulfonylimine **1aw** (72 mg, 0.2 mmol, 1.0 equiv.) in toluene (4 mL). Purification by flash column chromatography (*n*-hexane:EtOAc, 80:20 to 50:50) and subsequent washing with Et_2O, afforded the title compound (19 mg, 32%) as a colourless solid. **mp** (*n*-hexane/EtOAc) 75 – 78 °C; **^{1}H NMR** (400 MHz, $CDCl_3$) δ_H 7.36 – 7.29 (m, 2H, Ph*H*), 7.25 – 7.18 (m, 3H, Ph*H*), 3.91 (app. dp, *J* = 12.0, 2.0 Hz, 2H, $NCH_AH_BCH_2$), 3.21 (t, *J* = 7.5 Hz, 2H, $SO_2CH_2CH_2$), 3.00 (t, *J* = 7.5 Hz, 2H, CH_2CH_2CO), 2.87 (app. td, *J* = 12.0, 2.5 Hz, 2H, $NCH_AH_BCH_2$), 2.62 (tt, *J* = 12.0, 3.5 Hz, 1H, $PhCH(CH_2)_2$), 2.25 (s, 3H, $COCH_3$), 1.99 – 1.89 (m, 2H, $NCH_2CH_AH_B$), 1.80 (app. qd, *J* = 13.0, 4.0 Hz, 2H,

$NCH_2CH_AH_B$); **^{13}C NMR** (101 MHz, $CDCl_3$) δ_C 204.7, 144.9, 128.8, 126.9, 126.8, 46.6, 43.4, 42.2, 36.8, 33.1, 30.1; **LRMS** (ESI, *m/z*) $[M+H]^+$ 296.2, $[M+Na]^+$ 318.2.; **HRMS** (ESI, *m/z*) calculated for $(C_{15}H_{21}NNaO_3S)^+$ 318.1134 $[M+Na]^+$, found 318.1136; **IR** ($CHCl_3$, ν_{max}/cm^{-1}) 1710, 1326, 1248, 1151, 1054, 939, 837, 760, 702, 640; **R$_f$** 0.19 (*n*-hexane:EtOAc, 60:40).

Methyl 2,4-dichloro-5-((3-oxobutyl)sulfonyl)benzoate (2x)

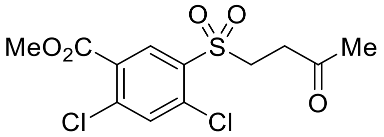

Prepared according to general procedure D, using *N*-sulfonylimine **1ax** (80 mg, 0.2 mmol, 1.0 equiv.) in toluene (4 mL). Purification by flash column chromatography (*n*-hexane:EtOAc, 80:20 to 50:50), afforded the title compound (41 mg, 61%) as a colourless solid. **mp** (*n*-hexane/EtOAc) 125 – 127 °C; **^{1}H NMR** (400 MHz, $CDCl_3$) δ_H 8.54 (s, 1H, Ar*H*), 7.69 (s, 1H, Ar*H*), 3.96 (s, 3H, CO_2CH_3), 3.66 (t, *J* = 7.5 Hz, 2H, $SO_2CH_2CH_2$), 2.95 (t, *J* = 7.5 Hz, 2H, CH_2CH_2CO), 2.19 (s, 3H, $COCH_3$); **^{13}C NMR** (101 MHz, $CDCl_3$) δ_C 203.3, 163.7, 140.8, 136.6, 135.5, 134.4, 129.2, 53.2, 49.1, 35.6, 30.0; **LRMS** (ESI, *m/z*) $[M+Na]^+$ 361.0; **HRMS** (ESI, *m/z*) calculated for $(C_{12}H_{12}{}^{35}Cl_2NaO_5S)^+$ 360.9675 $[M+Na]^+$, found 360.9676; **IR** ($CHCl_3$, ν_{max}/cm^{-1}) 1737, 1721, 1583, 1540, 1435, 1319, 1290, 1256, 1153, 1116, 1075, 971, 891, 839; **R$_f$** 0.57 (*n*-hexane:EtOAc, 60:40).

4-((6-Chloro-1,1-dioxido-3,4-dihydro-2*H*-benzo[*e*][1,2,4]thiadiazin-7-yl)sulfonyl)butan-2-one (2y)

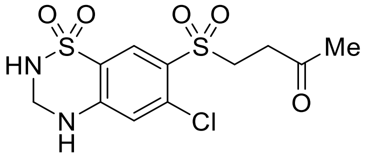

Prepared according to general procedure D, using *N*-sulfonylimine **1ay** (80 mg, 0.2 mmol, 1.0 equiv.) in acetonitrile (4 mL). Purification by flash column chromatography (*n*-hexane:EtOAc, 80:20 to 50:50), afforded the title compound (25 mg, 36%) as a colourless solid. **mp** (*n*-hexane/EtOAc) 117 – 119 °C; **^{1}H NMR** (400 MHz, MeOD-d_4) δ_H 8.12 (s, 1H, Ar*H*), 6.97 (s, 1H, Ar*H*), 4.80 (s, 2H, $(NH)_2CH_2$), 3.64 (t, *J* = 7.0 Hz, 2H, $SO_2CH_2CH_2$), 2.91 (t, *J* = 7.0 Hz, 2H, CH_2CH_2CO), 2.13 (s, 3H, $COCH_3$); **^{13}C NMR** (101 MHz, MeOD-d_4) δ_C 206.3, 149.8, 137.7, 130.2, 123.9, 121.0, 118.5, 56.0, 50.3, 36.8, 29.7; **LRMS** (ESI, *m/z*) $[M+Na]^+$ 375.0; **HRMS** (ESI, *m/z*) calculated for $(C_{11}H_{13}{}^{35}ClN_2NaO_5S_2)^+$ 374.9847 $[M+Na]^+$, found 374.9846; **IR** ($CHCl_3$, ν_{max}/cm^{-1}) 1596, 1552, 1514, 1457, 1412, 1367, 1322, 1238, 1156, 1113, 1055, 1026, 920, 856, 732, 675, 642; **R$_f$** 0.61 (EtOAc).

***N*-(4-((3-oxobutyl)sulfonyl)phenyl)acetamide (2z)**

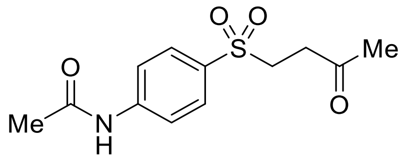

Prepared according to general procedure D, using *N*-sulfonylimine **1az** (67 mg, 0.2 mmol, 1.0 equiv.) in toluene (4 mL). Purification by flash column chromatography (*n*-hexane:EtOAc, 80:20 to 20:80), afforded the title compound (50 mg, 93%) as an off-white solid. **mp** (*n*-hexane/EtOAc) 129 – 131 °C; **^{1}H NMR** (400 MHz, $CDCl_3$) δ_H 8.12 (s, 1H, ArN*H*CO), 7.78 (d, *J* = 9.0 Hz, 2H, Ar*H*), 7.71 (d, *J* = 9.0 Hz, 2H, Ar*H*), 3.38 (d, *J* = 7.5 Hz, 2H, $SO_2CH_2CH_2$), 2.90 (t, *J* = 7.5 Hz, 2H, CH_2CH_2CO), 2.19 (s, 3H, CH_2COCH_3), 2.17 (s, 3H, $NHCOCH_3$); **^{13}C NMR** (101 MHz, $CDCl_3$) δ_C 204.0, 169.2, 143.5, 133.2, 129.3, 119.7, 50.8, 36.2, 30.0, 24.8; **LRMS** (ESI, *m/z*) $[M+Na]^+$ 292.1; **HRMS** (ESI, *m/z*) calculated for $(C_{12}H_{15}NNaO_4S)^+$ 292.0614 $[M+Na]^+$, found 292.0615; **IR** ($CHCl_3$, ν_{max}/cm^{-1}) 1701, 1591, 1531, 1402, 1370, 1319, 1263, 1137, 1089, 1034, 841, 753; **R$_f$** 0.53 (EtOAc).

4-((4-(5-(*p*-Tolyl)-3-(trifluoromethyl)-1*H*-pyrazol-1-yl)phenyl)sulfonyl)butan-2-one (2aa)

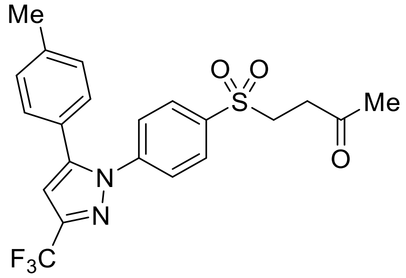

Prepared according to general procedure D, using *N*-sulfonylimine **3a** (100 mg, 0.20 mmol, 1.0 equiv.) and methyl vinyl ketone (42 µL, 0.50 mmol, 2.5 equiv.) in toluene (4 mL). Purification by silica gel column chromatography (*n*-pentane:EtOAc, 90:10 to 70:30), afforded the title compound (68 mg, 78%) as a yellow oil. **^{1}H NMR** (400 MHz, $CDCl_3$) δ_H 7.88 (d, *J* = 8.5 Hz, 2H, Ar*H*), 7.53 (d, *J* = 8.5 Hz, 2H, Ar*H*), 7.18 (d, *J* = 8.0 Hz, 2H, Ar*H*), 7.11 (d, *J* = 8.0 Hz, 2H, Ar*H*), 6.74 (s, 1H, Ar*H*), 3.37 (dd, *J* = 7.5 Hz, 2H, $SO_2CH_2CH_2CO$), 2.91 (dd, *J* = 7.5 Hz, 2H, $SO_2CH_2CH_2CO$), 2.38 (s, 3H, $ArCH_3$), 2.18 (s, 3H, $COCH_3$); **^{13}C NMR** (101 MHz, $CDCl_3$) δ_C 203.5, 145.5, 144.4 (q, *J* = 38.0 Hz), 143.8, 140.4, 138.3, 130.0, 129.2, 128.8, 125.8, 125.7, 121.1 (q, *J* = 269.5 Hz), 106.7 (q, *J* = 2.0 Hz), 50.6, 35.8, 30.0, 21.4; **^{19}F NMR** (377 MHz, $CDCl_3$) δ_F -62.53; **LRMS** (ESI, *m/z*) $[M+H]^+$ 437.0, $[M+Na]^+$ 459.0; **HRMS** (ESI, *m/z*) calculated for $(C_{21}H_{20}O_3N_2F_3S)^+$ 437.1141 $[M+H]^+$, found 437.1140; **IR** ($CDCl_3$, ν_{max}/cm^{-1}) 1721, 1450, 1408, 1236, 1133, 1095, 974, 910, 730; **R$_f$** 0.33 (petrol:EtOAc, 70:30).

Benzyl 4-chloro-2-((furan-2-ylmethyl)amino)-5-((3-oxobutyl)sulfonyl)benzoate (2ab)

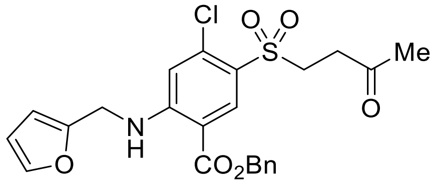

Prepared according to general procedure D, using *N*-sulfonylimine **3b** (108 mg, 0.2 mmol, 1.0 equiv.) in toluene (4 mL). Purification by flash column chromatography (*n*-hexane:EtOAc, 80:20 to 60:40), afforded the title compound (64 mg, 67%) as a colourless solid. **mp** (*n*-hexane/EtOAc) 97 – 99 °C; **^{1}H NMR** (400 MHz, $CDCl_3$) δ 8.75 (t, *J* = 5.5 Hz, 1H, CH_2NHAr), 8.63 (s, 1H, Ar*H*), 7.46 – 7.31 (m, 6H, Ph*H*, HetAr*H*), 6.89 (s, 1H, Ar*H*), 6.35 (dd, *J* = 3.5, 2.0 Hz, 1H, HetAr*H*), 6.28 (dd, *J* = 3.5, 1.0 Hz, 1H, HetAr*H*), 5.33 (s, 3H, CO_2CH_2Ph), 4.44 (d, *J* = 5.5 Hz, 2H, HetArCH_2NH), 3.59 (t, *J* = 7.5 Hz, 2H, $SO_2CH_2CH_2$), 2.93 (t, *J* = 7.5 Hz, 2H, CH_2CH_2CO), 2.17 (s, 3H $COCH_3$); **^{13}C NMR** (101 MHz, $CDCl_3$) δ_C 204.0, 166.9, 153.9, 150.0, 142.8, 138.8, 136.3, 135.6, 128.8, 128.6, 128.4, 122.1, 114.0, 110.7, 108.8, 108.1, 67.0, 49.5, 40.4, 35.9, 30.0; **LRMS** (ESI, *m/z*) $[M+Na]^+$ 498.1; **HRMS** (ESI, *m/z*) calculated for $(C_{23}H_{22}{}^{35}ClNNaO_6S)^+$ 498.0749 $[M+Na]^+$, found 498.0746; **IR** ($CHCl_3$, ν_{max}/cm^{-1}) 3334, 1719, 1689, 1593, 1570, 1313, 1279, 1258, 1229, 1151, 838, 749, 699; **R_f** 0.40 (*n*-hexane:EtOAc, 60:40).

3-((4-(5-(*p*-Tolyl)-3-(trifluoromethyl)-1*H*-pyrazol-1-yl)phenyl)sulfonyl)propanenitrile (2ac)

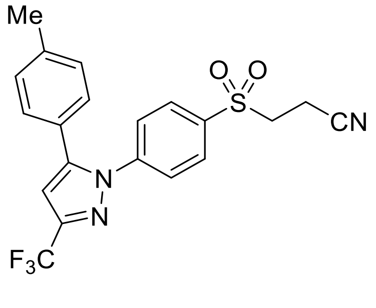

Prepared according to general procedure D, using *N*-sulfonylimine **3a** (100 mg, 0.20 mmol, 1.0 equiv.) and acrylonitrile (33 μL, 0.50 mmol, 2.5 equiv.) in toluene (4 mL). Purification by silica gel column chromatography (*n*-pentane:EtOAc, 90:10 to 70:30) afforded the title compound (60 mg, 72%) as a yellow solid. **mp** (CH_2Cl_2) 154 – 160 °C; **^{1}H NMR** (400 MHz, $CDCl_3$) δ_H 7.91 (d, *J* = 9.0 Hz, 2H, Ar*H*), 7.59 (d, *J* = 8.5 Hz, 2H, Ar*H*), 7.19 (d, *J* = 8.0 Hz, 2H, Ar*H*), 7.12 (d, *J* = 8.0 Hz, 2H, Ar*H*), 6.75 (s, 1H, Ar*H*), 3.40 (t, *J* = 7.5 Hz, 2H, $SO_2CH_2CH_2CN$), 2.83 (t, *J* = 7.5 Hz, 2H, $SO_2CH_2CH_2CN$), 2.39 (s, 3H, ArCH_3); **^{13}C NMR** (101 MHz, $CDCl_3$) δ_C 145.6, 144.6 (q, *J* = 40.5 Hz), 144.4, 140.2, 136.8, 130.0, 129.5, 128.9, 125.9, 125.7, 121.1 (q, *J* = 268.5 Hz), 117.1, 106.9 (q, *J* = 2.0 Hz), 51.3, 21.5, 12.1; **^{19}F NMR** (377 MHz, $CDCl_3$) δ_F -62.52; **LRMS**

(ESI, *m/z*) [M+H]$^+$ 420.0; **HRMS** (ESI, *m/z*) calculated for $(C_{20}H_{17}O_2N_3F_3S)^+$ 420.0988 [M+H]$^+$, found 420.0987; **IR** ($CDCl_3$, ν_{max}/cm^{-1}) 2235, 1380, 1161, 1137, 906, 729; **R$_f$** 0.21 (petrol:EtOAc, 70:30).

Benzyl 3-((4-(5-(*p*-tolyl)-3-(trifluoromethyl)-1*H*-pyrazol-1-yl)phenyl)sulfonyl)propanoate (2ad)

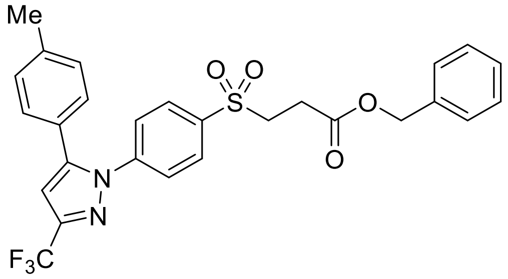

Prepared according to general procedure D, using *N*-sulfonylimine **3a** (100 mg, 0.20 mmol, 1.0 equiv.) and benzyl acrylate (77 μL, 0.50 mmol, 2.5 equiv.) in toluene (4 mL). Purification by silica gel column chromatography (*n*-pentane:EtOAc, 90:10 to 80:20) afforded the title compound (87 mg, 82%) as a yellow oil. **^{1}H NMR** (400 MHz, $CDCl_3$) δ_H 7.88 (d, *J* = 8.5 Hz, 2H, Ar*H*), 7.51 (d, *J* = 8.5 Hz, 2H, Ar*H*), 7.40 – 7.27 (m, 5H, Ar*H*), 7.19 (d, *J* = 8.0 Hz, 2H, Ar*H*), 7.11 (d, *J* = 8.5 Hz, 2H, Ar*H*), 6.76 (s, 1H, Ar*H*), 5.08 (s, 2H, OC*H*$_2$Ph), 3.45 (t, *J* = 7.5 Hz, 2H, $SO_2CH_2CH_2CO$), 2.80 (t, *J* = 7.5 Hz, 2H, $SO_2CH_2CH_2CO$), 2.39 (s, 3H, ArC*H*$_3$); **^{13}C NMR** (101 MHz, $CDCl_3$) δ_C 169.7, 145.5, 144.4 (q, *J* = 38.0 Hz), 143.8, 140.0, 137.8, 135.2, 129.9, 129.4, 128.8, 128.7, 128.6, 128.5, 125.7, 125.6, 121.1 (q, *J* = 269.5 Hz), 106.6 (q, *J* = 2.0 Hz), 67.4, 51.6, 27.9, 21.4; **^{19}F NMR** (377 MHz, $CDCl_3$) δ_F -62.45; **LRMS** (ESI, *m/z*) [M+Na]$^+$ 551.0; **HRMS** (ESI, *m/z*) calculated for $(C_{27}H_{24}O_4N_2F_3S)^+$ 529.1403 [M+H]$^+$, found 529.1403; **IR** ($CDCl_3$, ν_{max}/cm^{-1}) 1737, 1375, 1151, 1133, 973, 731; **R$_f$** 0.18 (petrol:EtOAc, 80:20).

1-(4-((2-(Methylsulfonyl)ethyl)sulfonyl)phenyl)-5-(*p*-tolyl)-3-(trifluoromethyl)-1*H*-pyrazole (2ae)

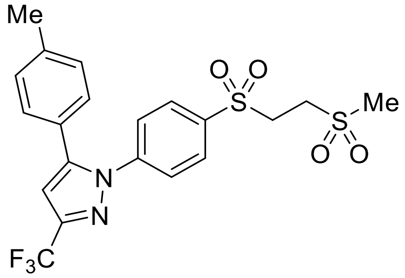

Prepared according to general procedure D, using *N*-sulfonylimine **3a** (100 mg, 0.20 mmol, 1.0 equiv.) and methyl vinyl sulfone (44 μL, 0.50 mmol, 2.5 equiv.) in acetonitrile (4 mL). Purification by silica gel column chromatography (*n*-pentane:EtOAc, 90:10 to 60:40) afforded the title compound (35 mg, 37%) as a white solid. **mp** (CH_2Cl_2) 190 – 192 °C; **^{1}H NMR** (400 MHz, $CDCl_3$) δ_H 7.91 (d, *J* = 8.5 Hz, 2H, Ar*H*), 7.58 (d,

J = 8.5 Hz, 2H, ArH), 7.21 (d, J = 8.0 Hz, 2H, ArH), 7.12 (d, J = 8.0 Hz, 2H, ArH), 6.76 (s, 1H, ArH), 3.60 – 3.52 (m, 2H, $SO_2CH_2CH_2SO_2$), 3.47 – 3.42 (m, 2H, $SO_2CH_2CH_2SO_2$), 3.02 (s, 3H, SO_2CH_3), 2.40 (s, 3H, ArCH_3); **^{13}C NMR** (126 MHz, $CDCl_3$) δ_C 145.6, 144.6 (q, J = 38.0 Hz), 144.3, 140.2, 137.3, 130.1, 129.3, 128.9, 125.9, 125.7, 121.1 (q, J = 270.0 Hz), 106.9 (q, J = 2.0 Hz), 48.9, 47.6, 41.8, 21.5; **^{19}F NMR** (377 MHz, $CDCl_3$) δ_F -62.55; **LRMS** (ESI, *m/z*) $[M+H]^+$ 473.0, $[M+Na]^+$ 495.0; **HRMS** (ESI, *m/z*) calculated for $(C_{20}H_{20}O_4N_2F_3S_2)^+$ 473.0811 $[M+H]^+$, found 473.0813; **IR** ($CDCl_3$, ν_{max}/cm^{-1}) 1597, 1499, 1378, 1303, 1295, 1236, 1156, 977, 789; **R$_f$** 0.32 (petrol:EtOAc, 50:50).

3-((4-(5-(*p*-Tolyl)-3-(trifluoromethyl)-1*H*-pyrazol-1-yl)phenyl)sulfonyl)propanoic acid (2af)

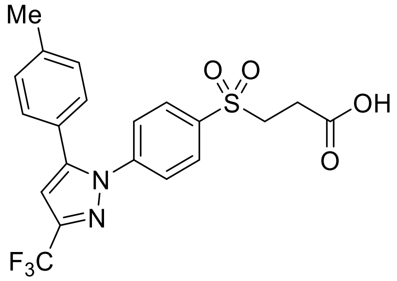

Prepared according to general procedure D, using *N*-sulfonylimine **3a** (100 mg, 0.20 mmol, 1.0 equiv.) and acrylic acid (35 mg, 0.50 mmol, 2.5 equiv.) in acetonitrile (4 mL). Purification by silica gel column chromatography (*n*-pentane:EtOAc + 1% AcOH, 90:10 to 50:50) afforded the title compound (32 mg, 36%) as an orange oil. **^{1}H NMR** (400 MHz, $CDCl_3$) δ_H 7.89 (d, J = 8.5 Hz, 2H, ArH), 7.53 (d, J = 8.5 Hz, 2H, ArH), 7.19 (d, J = 8.0 Hz, 2H, ArH), 7.10 (d, J = 8.0 Hz, 2H, ArH), 6.75 (s, 1H, ArH), 3.42 (t, J = 7.5 Hz, 2H, $SO_2CH_2CH_2CO_2H$), 2.79 (t, J = 7.5 Hz, 2H, $SO_2CH_2CH_2CO_2H$), 2.38 (s, 3H, ArCH_3); **^{13}C NMR** (151 MHz, $CDCl_3$) δ_C 174.6, 145.6, 144.5 (q, J = 38.5 Hz), 143.9, 140.2, 137.8, 130.0, 129.5, 128.9, 125.9, 125.7, 121.1 (q, J = 269.0 Hz), 106.7 (q, J = 2.0 Hz), 51.4, 27.6, 21.5; **^{19}F NMR** (377 MHz, $CDCl_3$) δ_F -62.50; **LRMS** (ESI, *m/z*) $[M+H]^+$ 439.0, $[M+Na]^+$ 461.0; **HRMS** (ESI, *m/z*) calculated for $(C_{20}H_{18}O_4N_2F_3S)^+$ 439.0934 $[M+H]^+$, found 439.0931; **IR** ($CDCl_3$, ν_{max}/cm^{-1}) 3503, 1719, 1498, 1237, 1156, 1136, 975; **R$_f$** 0.19 (petrol:EtOAc + 1% AcOH, 70:30).

Pent-4-en-1-yl 3-((4-(5-(*p*-tolyl)-3-(trifluoromethyl)-1*H*-pyrazol-1-yl)phenyl)sulfonyl)-propanoate (2ag)

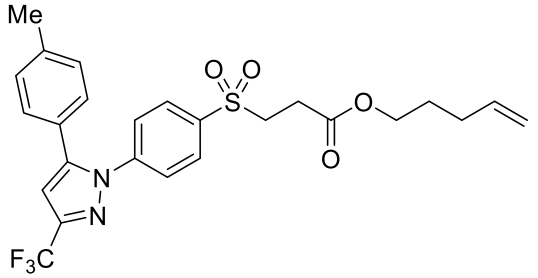

Prepared according to general procedure D, using *N*-sulfonylimine **3a** (100 mg, 0.20 mmol, 1.0 equiv.) and pent-4-en-1-yl acrylate (**8a**) (70 mg, 0.50 mmol, 2.5 equiv.) in acetonitrile (4 mL). Purification by silica gel column chromatography (*n*-pentane:EtOAc, 90:10 to 80:20) afforded the title compound (53 mg, 52%) as a colorless oil. **^{1}H NMR** (600 MHz, $CDCl_3$) δ_H 7.90 (d, *J* = 8.5 Hz, 2H, Ar*H*), 7.54 (d, *J* = 8.5 Hz, 2H, Ar*H*), 7.19 (d, *J* = 8.0 Hz, 2H, Ar*H*), 7.11 (d, *J* = 8.0 Hz, 2H, Ar*H*), 6.75 (s, 1H, Ar*H*), 5.77 (ddt, *J* = 17.0, 10.0, 6.5 Hz, 1H, $CH_2CH_2CH{=}CH_AH_B$), 5.02 (dd, *J* = 17.0, 1.5 Hz, 1H, $CH_2CH_2CH{=}CH_AH_B$), 4.98 (dd, *J* = 10.0, 1.5 Hz, 1H, $CH_2CH_2CH{=}CH_AH_B$), 4.07 (t, *J* = 6.5 Hz, 2H, $CO_2CH_2CH_2$), 3.43 (t, *J* = 7.5 Hz, 2H, $SO_2CH_2CH_2CO$), 2.74 (t, *J* = 7.5 Hz, 2H, $SO_2CH_2CH_2CO$), 2.39 (s, 3H, $ArCH_3$), 2.10 (dt, *J* = 7.0, 6.5 Hz, 2H, $CO_2CH_2CH_2CH_2CH{=}CH_2$), 1.71 (p, *J* = 7.0 Hz, 2H, $CO_2CH_2CH_2CH_2CH{=}CH_2$); **^{13}C NMR** (151 MHz, $CDCl_3$) δ_C 170.0, 145.5, 144.5 (q, *J* = 38.5 Hz), 143.9, 140.1, 137.9, 137.3, 130.0, 129.4, 128.9, 125.8 (2C), 121.1 (q, *J* = 269.0 Hz), 115.6, 106.7 (q, *J* = 2.0 Hz), 65.1, 51.7, 30.0, 27.8, 27.7, 21.5; **^{19}F NMR** (377 MHz, $CDCl_3$) δ_F -62.53; **LRMS** (ESI, *m/z*) $[M+H]^+$ 507.1, $[M+Na]^+$ 529.1; **HRMS** (ESI, *m/z*) calculated for $(C_{25}H_{26}O_4N_2F_3S)^+$ 507.1560 $[M+H]^+$, found 507.1561; **IR** ($CHCl_3$, ν_{max}/cm^{-1}) 2980, 2917, 1734, 1597, 1472, 1238, 1153, 1142, 908, 730; **R_f** 0.25 (petrol:EtOAc, 80:20).

Prop-2-yn-1-yl 3-((4-(5-(*p*-tolyl)-3-(trifluoromethyl)-1*H*-pyrazol-1-yl)phenyl)sulfonyl)-propanoate (2ah)

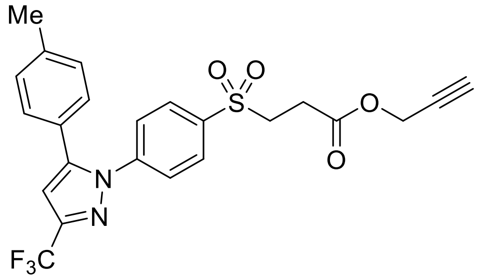

Prepared according to general procedure D, using sulfonylimine **3a** (100 mg, 0.20 mmol, 1.0 equiv.) and prop-2-yn-1-yl acrylate (**8b**) (55 mg, 0.50 mmol, 2.5 equiv.) in acetonitrile (4 mL). Purification by silica gel column chromatography (*n*-pentane:EtOAc, 95:5 to 80:20) afforded the title compound (58 mg, 61%) as a sticky white

foam. **^{1}H NMR** (600 MHz, $CDCl_3$) δ_H 7.90 (d, *J* = 8.5 Hz, 2H, Ar*H*), 7.54 (d, *J* = 8.5 Hz, 2H, Ar*H*), 7.19 (d, *J* = 8.0 Hz, 2H, Ar*H*), 7.11 (d, *J* = 8.0 Hz, 2H, Ar*H*), 6.75 (s, 1H, Ar*H*), 4.66 (d, *J* = 2.5 Hz, 2H, OCH_2CCH), 3.45 (t, *J* = 7.5 Hz, 2H, $SO_2CH_2CH_2CO$), 2.81 (t, *J* = 7.5 Hz, 2H, $SO_2CH_2CH_2CO$), 2.49 (t, *J* = 2.5 Hz, 2H, OCH_2CCH), 2.39 (s, 3H, $ArCH_3$); **^{13}C NMR** (151 MHz, $CDCl_3$) δ_C 169.2, 145.5, 144.5 (q, *J* = 38.5 Hz), 143.9, 140.1, 137.8, 130.0, 129.4, 128.9, 125.8, 125.7, 121.1 (q, *J* = 269.0 Hz), 106.7 (q, *J* = 2.0 Hz), 77.0, 75.7, 53.0, 51.5, 27.6, 21.5; **^{19}F NMR** (377 MHz, $CDCl_3$) δ_F -62.53; **LRMS** (ESI, *m/z*) $[M+H]^+$ 478.0; **HRMS** (ESI, *m/z*) calculated for $(C_{23}H_{20}O_4N_2F_3S)^+$ 477.1090 $[M+H]^+$, found 477.1089; **IR** ($CHCl_3$, ν_{max}/cm^{-1}) 2916, 2219, 1746, 1597, 1471, 1238, 1153, 1141, 908, 757, 733; **R_f** 0.43 (petrol:EtOAc, 80:20).

N-(*tert*-Butyl)-3-((4-(5-(*p*-tolyl)-3-(trifluoromethyl)-1*H*-pyrazol-1-yl)phenyl)sulfonyl)-propanamide (2ai)

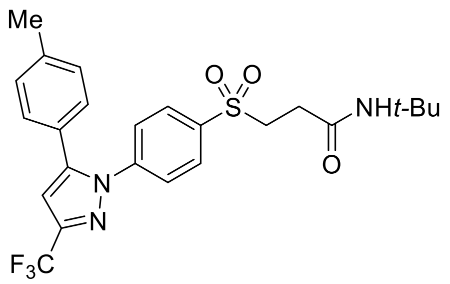

Prepared according to general procedure D, using *N*-sulfonylimine **3a** (100 mg, 0.20 mmol, 1.0 equiv.) and *N-tert*-butylacrylamide (64 mg, 0.50 mmol, 2.5 equiv.) in acetonitrile (4 mL). Purification by silica gel column chromatography (*n*-pentane:EtOAc, 90:10 to 70:30) afforded the title compound (73 mg, 74%) as a white solid. **mp** (CH_2Cl_2) 170 – 174 °C; **^{1}H NMR** (400 MHz, $CDCl_3$) δ_H 7.88 (d, *J* = 8.5 Hz, 2H, Ar*H*), 7.52 (d, *J* = 8.5 Hz, 2H, Ar*H*), 7.19 (d, *J* = 8.0 Hz, 2H, Ar*H*), 7.11 (d, *J* = 8.0 Hz, 2H, Ar*H*), 6.74 (s, 1H, Ar*H*), 5.46 (s, 1H, CON*H*), 3.50 – 3.37 (m, 2H, $SO_2CH_2CH_2CO$), 2.64 – 2.49 (m, 2H, $SO_2CH_2CH_2CO$), 2.38 (s, 3H, $ArCH_3$), 1.30 (s, 9H, $C(CH_3)_3$); **^{13}C NMR** (151 MHz, $CDCl_3$) δ_C 167.6, 145.5, 144.4 (q, *J* = 38.5 Hz), 143.7, 140.1, 138.4, 130.0, 129.2, 128.9, 125.7 (2C), 121.1 (q, *J* = 269.0 Hz), 106.7 (q, *J* = 2.0 Hz), 52.1, 51.9, 29.6, 28.8, 21.5; **^{19}F NMR** (377 MHz, $CDCl_3$) δ_F -62.51; **LRMS** (ESI, *m/z*) $[M+H]^+$ 494.2; **HRMS** (ESI, *m/z*) calculated for $(C_{24}H_{22}O_3N_3F_3S)^+$ 494.1720 $[M+H]^+$, found 494.1714; **IR** ($CHCl_3$, ν_{max}/cm^{-1}) 3377, 1663, 1454, 1237, 1152, 1137, 975; **R_f** 0.35 (petrol:EtOAc, 70:30).

1-Morpholino-3-((4-(5-(*p*-tolyl)-3-(trifluoromethyl)-1*H*-pyrazol-1-yl)phenyl)sulfonyl)-propan-1-one (2aj)

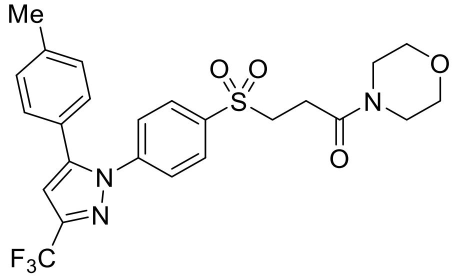

Prepared according to general procedure D, using *N*-sulfonylimine **3a** (100 mg, 0.20 mmol, 1.0 equiv.) and *N*-acryloylmorpholine (63 µL, 0.50 mmol, 2.5 equiv.) in acetonitrile (4 mL). Purification by silica gel column chromatography (*n*-pentane:EtOAc, 50:50) afforded the title compound (61 mg, 60%) as a yellow oil. **^{1}H NMR** (400 MHz, $CDCl_3$) δ_H 7.90 (d, *J* = 8.5 Hz, 2H, Ar*H*), 7.54 (d, *J* = 8.5 Hz, 2H, Ar*H*), 7.19 (d, *J* = 8.0 Hz, 2H, Ar*H*), 7.11 (d, *J* = 8.0 Hz, 2H, Ar*H*), 6.75 (s, 1H, Ar*H*), 3.70 – 3.63 (m, 4H), 3.59 – 3.56 (m, 2H), 3.53 – 3.41 (m, 4H), 2.85 – 2.77 (m, 2H), 2.39 (s, 3H, ArC*H*$_3$); **^{13}C NMR** (101 MHz, $CDCl_3$) δ_C 167.3, 145.5, 144.4 (q, *J* = 38.0 Hz), 143.8, 140.1, 138.4, 130.0, 129.2, 128.8, 125.8, 121.1 (q, *J* = 268.5 Hz), 106.7 (q, *J* = 2.0 Hz), 66.8, 66.5, 52.1, 45.9, 42.4, 25.8, 21.4; **^{19}F NMR** (377 MHz, $CDCl_3$) δ_F -62.51; **LRMS** (ESI, *m/z*) $[M+H]^+$ 508.2, $[M+Na]^+$ 530.2; **HRMS** (ESI, *m/z*) calculated for $(C_{24}H_{25}O_4N_3F_3S)^+$ 508.1512 $[M+H]^+$, found 508.1512; **IR** ($CHCl_3$, ν_{max}/cm^{-1}) 1647, 1472, 1273, 1153, 975, 767; **R$_f$** 0.33 (petrol:EtOAc, 30:70).

Ethyl 2-methyl-3-((4-(5-(*p*-tolyl)-3-(trifluoromethyl)-1*H*-pyrazol-1-yl)phenyl)sulfonyl)-propanoate (2ak)

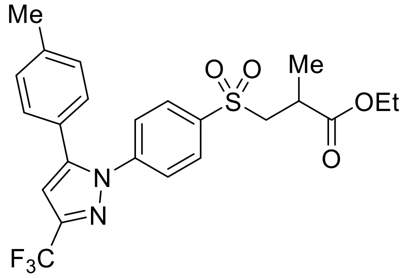

Prepared according to general procedure D, using *N*-sulfonylimine **3a** (100 mg, 0.20 mmol, 1.0 equiv.) and ethyl methacrylate (62 µL, 0.50 mmol, 2.5 equiv.) in toluene (4 mL). Purification by silica gel column chromatography (*n*-pentane:EtOAc, 90:10 to 85:15) afforded the title compound (73 mg, 76%) as a yellow oil. **^{1}H NMR** (400 MHz, $CDCl_3$) δ_H 7.89 (d, *J* = 8.5 Hz, 2H, Ar*H*), 7.52 (d, *J* = 8.5 Hz, 2H, Ar*H*), 7.18 (d, *J* = 8.0 Hz, 2H, Ar*H*), 7.10 (d, *J* = 8.0 Hz, 2H, Ar*H*), 6.74 (s, 1H, Ar*H*), 4.08 (q, *J* = 7.5 Hz, 2H, OC*H*$_2$CH$_3$), 3.70 (dd, *J* = 14.0, 7.5 Hz, 1H, $SO_2CH_AH_BCH$), 3.07 (dd, *J* = 14.0, 5.5 Hz, 1H, $SO_2CH_AH_BCH$), 3.02 – 2.93 (m, 1H, $SO_2CH_AH_BCH$), 2.38 (s, 3H, ArC*H*$_3$), 1.31 (d, *J* = 7.5 Hz, 3H, CHC*H*$_3$), 1.24 (t, *J* = 7.5 Hz, 3H, OCH$_2$C*H*$_3$);

^{13}**C NMR** (101 MHz, $CDCl_3$) δ_C 173.5, 145.5, 144.4 (q, *J* = 39.0 Hz), 143.7, 140.0, 138.6, 129.9, 129.4, 128.8, 125.7, 121.1 (q, *J* = 268.5 Hz), 106.6 (q, *J* = 2.0 Hz), 77.2, 61.5, 58.9, 34.9, 21.4, 18.0, 14.1; ^{19}**F NMR** (377 MHz, $CDCl_3$) δ_F -62.52; **LRMS** (ESI, *m/z*) $[M+Na]^+$ 503.0; **HRMS** (ESI, *m/z*) calculated for $(C_{23}H_{23}O_4N_2F_3SNa)^+$ 503.1223 $[M+Na]^+$, found 503.1221; **IR** ($CDCl_3$, ν_{max}/cm^{-1}) 1733, 1596, 1498, 1236, 1137, 975, 845, 809; $\mathbf{R_f}$ 0.23 (petrol:EtOAc, 80:20).

***N*-(*tert*-Butyl)-3-((4-(5-(*p*-tolyl)-3-(trifluoromethyl)-1*H*-pyrazol-1-yl)phenyl)sulfonyl)-propanamide (2al)**

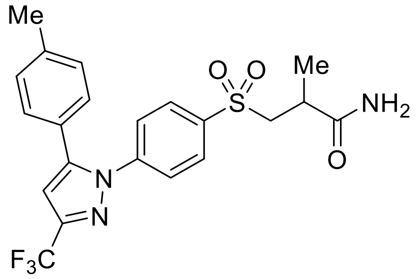

Prepared according to general procedure D, using *N*-sulfonylimine **3a** (100 mg, 0.20 mmol, 1.0 equiv.) and methacrylamide (43 mg, 0.50 mmol, 2.5 equiv.) in acetonitrile (4 mL). Purification by silica gel column chromatography (*n*-pentane:EtOAc, 70:30 to 30:70) afforded the title compound (75 mg, 83%) as a white solid. **mp** (CH_2Cl_2) 62 – 68 °C; ^{1}**H NMR** (400 MHz, $CDCl_3$) δ_H 7.88 (d, *J* = 8.5 Hz, 2H, Ar*H*), 7.51 (d, *J* = 8.5 Hz, 2H, Ar*H*), 7.17 (d, *J* = 8.0 Hz, 2H, Ar*H*), 7.10 (d, *J* = 8.0 Hz, 2H, Ar*H*), 6.74 (s, 1H, Ar*H*), 6.06 (s, 1H, N*H*), 5.94 (s, 1H, N*H*), 3.80 – 3.69 (m, 1H, C*H*(CH_3)CO), 3.06 – 2.96 (m, 2H, CH_AH_BCH(CH_3)CO), 2.37 (s, 3H, ArCH_3), 1.30 (d, *J* = 7.0 Hz, 3H, CHCH_3); ^{13}**C NMR** (101 MHz, $CDCl_3$) δ_C 175.6, 145.6, 144.4 (q, *J* = 38.0 Hz), 143.6, 140.1, 138.9, 129.9, 129.2, 128.9, 125.8, 125.7, 121.1 (q, *J* = 269.0 Hz), 106.6 (q, *J* = 2.0 Hz), 59.0, 35.1, 21.4, 19.0; ^{19}**F NMR** (377 MHz, $CDCl_3$) δ_F -62.73; **LRMS** (ESI, *m/z*) $[M+H]^+$ 452.2, $[M+Na]^+$ 474.1; **HRMS** (ESI, *m/z*) calculated for $(C_{21}H_{21}O_3N_3F_3S)^+$ 452.1250 $[M+H]^+$, found 452.1246; **IR** ($CHCl_3$, ν_{max}/cm^{-1}) 3451, 3359, 3198, 1675, 1472, 1237, 1141, 975; $\mathbf{R_f}$ 0.21 (petrol:EtOAc, 50:50).

4-((4-(5-(*p*-Tolyl)-3-(trifluoromethyl)-1*H*-pyrazol-1-yl)phenyl)sulfonyl)pentan-2-one (2am)

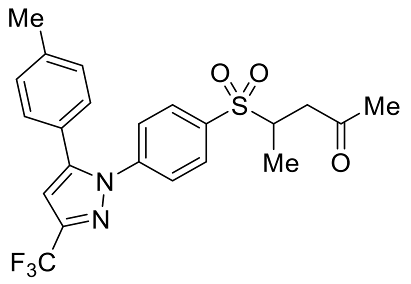

Prepared according to general procedure D, using *N*-sulfonylimine **3a** (100 mg, 0.20 mmol, 1.0 equiv.) and pent-3-en-2-one (49 μL, 0.50 mmol, 2.5 equiv.) in acetonitrile (4 mL). Purification by silica gel column chromatography (*n*-pentane:EtOAc, 95:5 to 80:20) afforded the title compound (51 mg, 57%) as an orange oil. **^{1}H NMR** (400 MHz, $CDCl_3$) δ_H 7.86 (d, *J* = 8.5 Hz, 2H, Ar*H*), 7.53 (d, *J* = 8.5 Hz, 2H, Ar*H*), 7.18 (d, *J* = 8.0 Hz, 2H, Ar*H*), 7.10 (d, *J* = 8.0 Hz, 2H, Ar*H*), 6.75 (s, 1H, Ar*H*), 3.71 – 3.61 (m, 1H, $SO_2CH(CH_3)CH_AH_B$), 3.18 (dd, *J* = 18.0, 3.5 Hz, 1H, $CH(CH_3)CH_AH_BCO$), 2.58 (dd, *J* = 18.0, 9.5 Hz, 1H, $CH(CH_3)CH_AH_BCO$), 2.38 (s, 3H, $ArCH_3$), 2.19 (s, 3H, $COCH_3$), 1.22 (d, *J* = 6.5 Hz, 3H, $SO_2CH(CH_3)CH_AH_B$); **^{13}C NMR** (101 MHz, $CDCl_3$) δ_C 203.8, 145.5, 144.5 (q, *J* = 39.0 Hz), 143.8, 140.1, 136.5, 130.1, 130.0, 128.9, 125.7, 125.7, 121.1 (q, *J* = 268.5 Hz), 106.6 (q, *J* = 2.0 Hz), 55.7, 42.4, 30.5, 21.5, 14.3; **^{19}F NMR** (377 MHz, $CDCl_3$) δ_F -62.51; **LRMS** (ESI, *m/z*) $[M+H]^+$ 451.0, $[M+Na]^+$ 473.0; **HRMS** (ESI, *m/z*) calculated for $(C_{22}H_{22}O_3N_2F_3S)^+$ 451.1298 $[M+H]^+$, found 451.1297; **IR** ($CDCl_3$, ν_{max}/cm^{-1}) 1721, 1596, 1472, 1236, 1135, 975, 845, 765; **R_f** 0.15 (petrol:EtOAc, 80:20).

Methyl *N,N*-bis(*tert*-butoxycarbonyl)-C3-((4-(5-(*p*-tolyl)-3-(trifluoromethyl)-1*H*-pyrazol-1-yl)phenyl)sulfonyl)alaninate (2an)

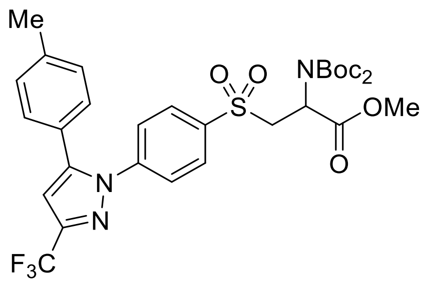

Prepared according to general procedure D, using *N*-sulfonylimine **3a** (100 mg, 0.20 mmol, 1.0 equiv.) and methyl 2-(bis[*tert*-butoxycarbonyl]amino)acrylate (**8c**) (151 mg, 0.50 mmol, 2.5 equiv.) in toluene (4 mL). Purification by silica gel column chromatography (*n*-pentane:EtOAc, 90:10 to 70:30) afforded the title compound (52 mg, 39%) as a yellow oil. **^{1}H NMR** (400 MHz, $CDCl_3$) δ_H 7.92 (d, *J* = 9.0 Hz, 2H, Ar*H*), 7.52 (d, *J* = 9.0 Hz, 2H, Ar*H*), 7.18 (d, *J* = 8.0 Hz, 2H, Ar*H*), 7.11 (d, *J* = 8.0 Hz, 2H, Ar*H*), 6.74 (s, 1H, Ar*H*), 5.48 (dd, *J* = 9.5, 3.0 Hz, 1H, $CH_AH_BCHNBoc_2$), 4.02 (dd, *J* = 15.5, 3.0 Hz, 1H, $CH_AH_BCHNBoc_2$), 3.82 (dd,

J = 15.5, 9.0 Hz, 1H, $CH_AH_BCHNBoc_2$), 3.70 (s, 3H, CO_2CH_3), 2.38 (s, 3H, $ArCH_3$), 1.49 (s, 18H, $COC(CH_3)_3$); **^{13}C NMR** (151 MHz, $CDCl_3$) δ_C 169.0, 151.8, 145.4, 144.4 (q, J = 38.0 Hz), 143.7, 140.0, 138.5, 130.0, 129.5, 128.8, 125.8, 125.7, 121.2 (q, J = 269.0 Hz), 106.6 (q, J = 2.0 Hz), 84.2, 56.3, 53.2, 28.3, 28.1, 21.5; **^{19}F NMR** (377 MHz, $CDCl_3$) δ_F -62.56; **LRMS** (ESI, *m/z*) $[M+Na]^+$ 690.2; **HRMS** (ESI, *m/z*) calculated for $(C_{31}H_{36}O_8N_3F_3SNa)^+$ 690.2067 $[M+Na]^+$, found 690.2064; **IR** ($CHCl_3$, ν_{max}/cm^{-1}) 1747, 1699, 1369, 1237, 1134; **R$_f$** 0.55 (Petrol:EtOAc, 70:30).

(8*R*,9*S*,13*S*,14*S*)-13-Methyl-17-oxo-7,8,9,11,12,13,14,15,16,17-decahydro-6*H*-cyclopenta[*a*]phenanthren-3-yl 3-((4-(5-(*p*-tolyl)-3-(trifluoromethyl)-1*H*-pyrazol-1-yl)phenyl)sulfonyl)propanoate (2ao)

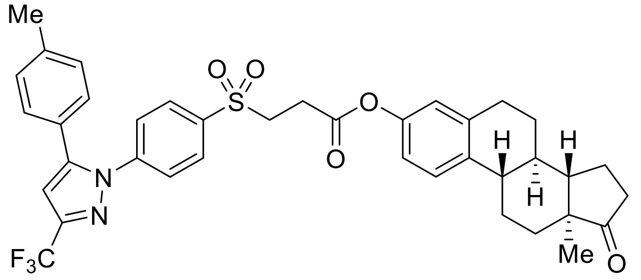

Prepared according to general procedure D, using *N*-sulfonylimine **3a** (50 mg, 0.10 mmol, 1.0 equiv.) and the estrone-derived acrylate (**8d**) (81 mg, 0.25 mmol, 2.5 equiv.) in toluene (4 mL). Purification by silica gel column chromatography (*n*-pentane:EtOAc, 90:10 to 60:40) afforded the title compound (58 mg, 84%) as a yellow oil. **^{1}H NMR** (600 MHz, $CDCl_3$) δ_H 7.84 (d, J = 8.5 Hz, 2H, Ar*H*), 7.56 (d, J = 8.5 Hz, 2H, Ar*H*), 7.26 (obs. d, J = 8.0 Hz, 2H, Ar*H*), 7.17 (d, J = 8.0 Hz, 2H, Ar*H*), 7.07 (d, J = 8.0 Hz, 2H, Ar*H*), 6.79 (dd, J = 8.5, 2.5 Hz, 1H, Ar*H*), 6.75 – 6.73 (m, 2H, Ar*H*), 3.54 (t, J = 7.5 Hz, 2H, $SO_2CH_2CH_2CO$), 2.99 (t, J = 7.5 Hz, 2H, $SO_2CH_2CH_2CO$), 2.86 (dd, J = 9.0, 4.5 Hz, 2H, CH_2CH_2CO), 2.50 (dd, J = 19.0, 8.5 Hz, 1H, $ArCH_AH_B$), 2.39 – 2.35 (m, 4H), 2.25 (td, J = 10.5, 4.5 Hz, 1H, ArC*H*), 2.13 (dd, J = 19.0, 9.0 Hz, 1H, $ArCH_AH_B$), 2.08 – 2.02 (m, 1H), 2.01 – 1.93 (m, 2H), 1.66 – 1.37 (m, 6H), 0.88 (s, 3H, CCH_3); **^{13}C NMR** (151 MHz, $CDCl_3$) δ_C 169.0, 148.3, 145.6, 144.5 (q, J = 38.5 Hz), 143.9, 140.1, 138.4, 138.1, 137.8, 130.0, 129.5, 128.9, 126.7, 125.8, 125.7 (obs. q, J = 13.0 Hz), 121.4, 121.1 (q, J = 269.0 Hz), 118.5, 106.7 (q, J = 1.5 Hz), 51.6, 50.6, 48.0, 44.2, 38.1, 36.0, 31.7, 29.5, 28.1, 26.4, 25.9, 21.7, 21.5, 13.9; **^{19}F NMR** (565 MHz, $CDCl_3$) δ_F -62.51; **LRMS** (ESI, *m/z*) $[M+H]^+$ 691.2, $[M+Na]^+$ 713.2; **HRMS** (ESI, *m/z*) calculated for $(C_{38}H_{38}O_5N_2F_3S)^+$ 691.2448 $[M+H]^+$, found 691.2447; **IR** ($CHCl_3$, ν_{max}/cm^{-1}) 2926, 2859, 1737, 1597, 1495, 1472, 1237, 1159, 1138, 975, 760; $[\alpha]_D^{25}$ +48 (c = 0.85, $CHCl_3$); **R$_f$** 0.27 (petrol:EtOAc, 70:30).

Methyl (3-((4-(5-(*p*-tolyl)-3-(trifluoromethyl)-1*H*-pyrazol-1-yl)phenyl)sulfonyl)-propan-oyl)-L-phenylalaninate (2ap)

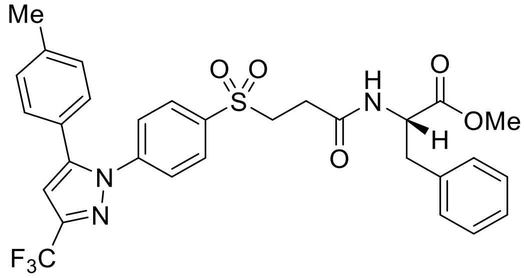

Prepared according to general procedure D, using *N*-sulfonylimine **3a** (100 mg, 0.20 mmol, 1.0 equiv.) and the phenylalanine-derived acrylamide (**8e**) (118 mg, 0.50 mmol, 2.5 equiv.) in acetonitrile (4 mL). Purification by silica gel column chromatography (*n*-pentane:EtOAc, 80:20 to 60:40) afforded the title compound (80 mg, 67%) as a sticky yellow oil. **^{1}H NMR** (600 MHz, $CDCl_3$) δ_H 7.86 (d, *J* = 8.5 Hz, 2H, Ar*H*), 7.51 (d, *J* = 8.5 Hz, 2H, Ar*H*), 7.30 – 7.22 (m, 3H, Ar*H*), 7.19 (d, *J* = 8.0 Hz, 2H, Ar*H*), 7.11 (d, *J* = 8.0 Hz, 2H, Ar*H*), 7.07 (d, *J* = 7.5 Hz, 2H, Ar*H*), 6.75 (s, 1H, Ar*H*), 6.03 (d, *J* = 8.0 Hz, 1H, CON*H*CH), 4.82 – 4.77 (m, 1H, NHC*H*), 3.73 (s, 3H, COOC*H*$_3$), 3.41 (t, *J* = 7.5 Hz, 2H, $SO_2CH_2CH_2CO$), 3.12 (dd, *J* = 14.0, 5.5 Hz, 1H, $CHCH_AH_BAr$), 3.06 (dd, *J* = 14.0, 6.0 Hz, 1H, $CHCH_AH_BAr$), 2.64 (t, *J* = 7.5 Hz, 2H, $SO_2CH_2CH_2CO$), 2.39 (s, 3H, ArC*H*$_3$); **^{13}C NMR** (151 MHz, $CDCl_3$) δ_C 171.8, 168.1, 145.5, 144.5 (q, *J* = 38.5 Hz), 143.8, 140.1, 138.2, 135.6, 130.0, 129.3 (2C), 128.9 (2C), 127.5, 125.8, 125.7, 121.2 (q, *J* = 269.0 Hz), 106.7 (q, *J* = 2.0 Hz), 53.5, 52.6, 51.8, 37.9, 28.9, 21.5; **^{19}F NMR** (565 MHz, $CDCl_3$) δ_F -62.52; **LRMS** (ESI, *m/z*) $[M+Na]^+$ 622.2; **HRMS** (ESI, *m/z*) calculated for $(C_{30}H_{29}O_5N_3F_3S)^+$ 600.1775 $[M+H]^+$, found 600.1772; **IR** ($CHCl_3$, ν_{max}/cm^{-1}) 3355, 3029, 2923, 2849, 1742, 1677, 1597, 1237, 1153, 1097; ***er*** was verified by HPLC using a Chiralpak IC column (*n*-hexane:*i*-PrOH, 70:30, 0.8 mL/min); τ_{major} = 58 min, τ_{minor} = 88 min (>99:1 *er*); $[\alpha]_D^{25}$ +21 (c = 2.25, $CHCl_3$); **R$_f$** 0.43 (petrol:EtOAc, 50:50).

2-(2-((4-(5-(*p*-Tolyl)-3-(trifluoromethyl)-1*H*-pyrazol-1-yl)phenyl)sulfonyl)ethyl)pyridine (2aq)

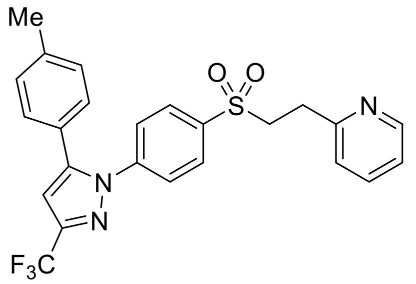

Prepared according to general procedure D, using *N*-sulfonylimine **3a** (50 mg, 0.10 mmol, 1.0 equiv.) and 2-vinylpyridine (27 μL, 0.25 mmol, 2.5 equiv.) in acetonitrile (2 mL). Purification by silica gel column chromatography (*n*-pentane:EtOAc, 90:10 to 50:50) afforded the title compound (20 mg, 43%) as a colorless

oil. **^{1}H NMR** (600 MHz, $CDCl_3$) δ_H 8.44 (d, *J* = 5.0 Hz, 1H, Py*H*), 7.89 (d, *J* = 8.5 Hz, 2H, Ar*H*), 7.59 (td, *J* = 7.5, 2.0 Hz, 1H, Py*H*), 7.50 (d, *J* = 8.5 Hz, 2H, Ar*H*), 7.18 (d, *J* = 8.0 Hz, 2H, Ar*H*), 7.15 – 7.08 (m, 4H, Ar*H*, Py*H*), 6.74 (s, 1H, Ar*H*), 3.67 – 3.62 (m, 2H, $SO_2CH_2CH_2Py$), 3.24 – 3.19 (m, 2H, $SO_2CH_2CH_2Py$), 2.38 (s, 3H, ArCH_3); **^{13}C NMR** (151 MHz, $CDCl_3$) δ_C 156.9, 149.6, 145.5, 144.4 (q, *J* = 38.5 Hz), 143.6, 140.0, 138.6, 136.9, 130.0, 129.4, 128.9, 125.7, 124.8, 123.4, 122.2, 121.2 (q, *J* = 269.0 Hz), 106.6 (q, *J* = 2.0 Hz), 55.2, 30.7, 21.5; **^{19}F NMR** (565 MHz, $CDCl_3$) δ_F -62.50; **LRMS** (ESI, *m/z*) $[M+H]^+$ 472.2; **HRMS** (ESI, *m/z*) calculated for $(C_{24}H_{21}O_2N_3F_3S)^+$ 472.1301 $[M+H]^+$, found 472.1302; **IR** ($CHCl_3$, ν_{max}/cm^{-1}) 3013, 2925, 1594, 1472, 1237, 1149, 1135, 1096, 975, 754; **R$_f$** 0.46 (petrol:EtOAc, 50:50).

4-(2-((4-(5-(*p*-Tolyl)-3-(trifluoromethyl)-1*H*-pyrazol-1-yl)phenyl)sulfonyl)ethyl)pyridine (2ar)

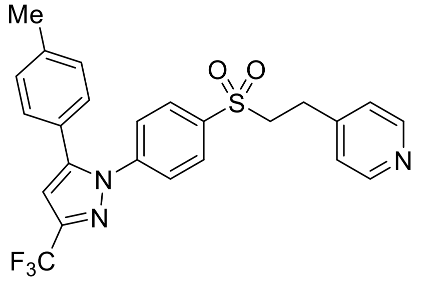

Prepared according to general procedure D, using *N*-sulfonylimine **3a** (100 mg, 0.20 mmol, 1.0 equiv.) and 4-vinylpyridine (53 μL, 0.50 mmol, 2.5 equiv.) in acetonitrile (4 mL). Purification by silica gel column chromatography (*n*-pentane:EtOAc, 50:50 to 40:60) afforded the title compound (50 mg, 53%) as a yellow oil. **^{1}H NMR** (400 MHz, $CDCl_3$) δ_H 8.52 (d, *J* = 5.5 Hz, 2H, Py*H*), 7.91 (d, *J* = 8.5 Hz, 2H, Ar*H*), 7.55 (d, *J* = 8.5 Hz, 2H, Ar*H*), 7.19 (d, *J* = 8.0 Hz, 2H, Ar*H*), 7.15 – 7.06 (m, 4H, Ar*H*, Py*H*), 6.75 (s, 1H, Ar*H*), 3.41 – 3.32 (m, 2H, $SO_2CH_2CH_2Py$), 3.10 – 3.00 (m, 2H, $SO_2CH_2CH_2Py$), 2.39 (s, 3H, ArCH_3); **^{13}C NMR** (126 MHz, $CDCl_3$) δ_C 150.1, 146.6, 145.5, 144.5 (q, *J* = 38.0 Hz), 143.9, 140.1, 138.0, 130.0, 129.3, 128.9, 125.9, 125.7, 123.8, 121.1 (q, *J* = 269.0 Hz), 106.7 (q, *J* = 2.0 Hz), 56.3, 28.2, 21.5; **^{19}F NMR** (377 MHz, $CDCl_3$) δ_F -62.53; **LRMS** (ESI, *m/z*) $[M+H]^+$ 472.0; **HRMS** (ESI, *m/z*) calculated for $(C_{24}H_{21}O_2N_3F_3S)^+$ 472.1301 $[M+H]^+$, found 472.1300; **IR** ($CHCl_3$, ν_{max}/cm^{-1}) 1598, 1450, 1236, 1151, 1133, 1096, 974, 807, 730; **R$_f$** 0.17 (petrol:EtOAc, 70:30).

N-(Pyridin-2-yl)-3-((4-(5-(p-tolyl)-3-(trifluoromethyl)-1H-pyrazol-1-yl)phenyl)sulfonyl)-propanamide (2as)

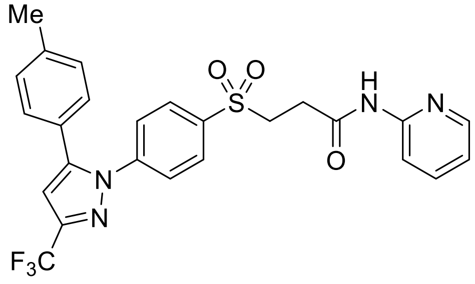

Prepared according to general procedure D, using *N*-sulfonylimine **3a** (75 mg, 0.15 mmol, 1.0 equiv.) and *N*-(pyridin-2-yl)acrylamide (56 mg, 0.38 mmol, 2.5 equiv.) in acetonitrile (3 mL). Purification by silica gel column chromatography (*n*-pentane:EtOAc, 80:20 to 50:50) afforded the title compound (20 mg, 79%) as a white solid. **mp** (*n*-pentane:EtOAc) 155 – 158 °C; **^{1}H NMR** (600 MHz, $CDCl_3$) δ_H 8.42 (s, 1H, CON*H*), 8.26 (d, *J* = 4.5 Hz, 1H, Py*H*), 8.02 (d, *J* = 7.0 Hz, 1H, Py*H*), 7.91 (d, *J* = 8.5 Hz, 2H, Ar*H*), 7.68 (td, *J* = 8.0, 2.0 Hz, 1H, Py*H*), 7.50 (d, *J* = 8.5 Hz, 2H, Ar*H*), 7.18 (d, *J* = 8.0 Hz, 2H, Ar*H*), 7.10 (d, *J* = 8.0 Hz, 2H, Ar*H*), 7.05 (dd, *J* = 7.5, 5.0 Hz, 1H, Py*H*), 6.74 (s, 1H, Ar*H*), 3.56 (t, *J* = 7.5 Hz, 2H, $SO_2CH_2CH_2CO$), 2.89 (t, *J* = 7.0 Hz, 2H, $SO_2CH_2CH_2CO$), 2.38 (s, 3H, ArCH_3); **^{13}C NMR** (151 MHz, $CDCl_3$) δ_C 167.1, 150.9, 148.0, 145.5, 144.5 (q, *J* = 38.5 Hz), 143.8, 140.1, 138.7, 138.1, 130.0, 129.4, 128.9, 125.8, 125.7, 121.2 (q, *J* = 269.0 Hz), 120.4, 114.3, 106.7 (q, *J* = 2.0 Hz), 51.6, 30.1, 21.5; **^{19}F NMR** (565 MHz, $CDCl_3$) δ_F -62.50; **LRMS** (ESI, *m/z*) $[M+H]^+$ 515.2; **HRMS** (ESI, *m/z*) calculated for $(C_{25}H_{22}O_3N_4F_3S)^+$ 515.1359 $[M+H]^+$, found 515.1362; **IR** ($CHCl_3$, ν_{max}/cm^{-1}) 2922, 1695, 1598, 1553, 1474, 1272, 1166, 1148, 1132, 977, 759; **R_f** 0.42 (petrol:EtOAc, 50:50).

N-(Benzo[d]thiazol-2-yl)-3-((4-(5-(p-tolyl)-3-(trifluoromethyl)-1H-pyrazol-1-yl)phenyl)-sulfonyl)propanamide (2at)

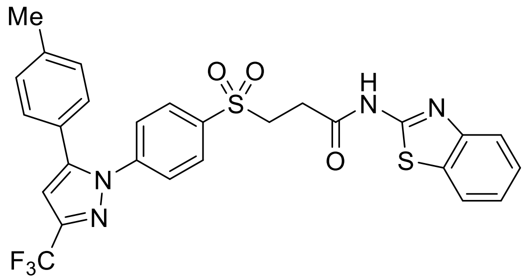

Prepared according to general procedure D, using *N*-sulfonylimine **3a** (100 mg, 0.20 mmol, 1.0 equiv.) and *N*-(benzo[*d*]thiazol-2-yl)acrylamide (**8f**) (102 mg, 0.50 mmol, 2.5 equiv.) in acetonitrile (4 mL). Purification by silica gel column chromatography (*n*-pentane:EtOAc, 90:10 to 70:30) afforded the title compound (74 mg, 65%) as a white solid. **mp** (*n*-pentane:EtOAc) 221 – 223 °C; **^{1}H NMR** (600 MHz, DMSO-d_6) δ_H 12.43 (s, 1H, CON*H*), 8.00 (d, *J* = 8.5 Hz, 2H, Ar*H*), 7.94 (d, *J* = 7.5 Hz, 1H, Ar*H*), 7.74 (d, *J* = 8.0 Hz, 1H, Ar*H*), 7.62 (d,

J = 8.5 Hz, 2H, Ar*H*), 7.43 (t, J = 7.5 Hz, 1H, Ar*H*), 7.29 (t, J = 7.5 Hz, 1H, Ar*H*), 7.22 – 7.17 (m, 5H, Ar*H*), 3.75 (t, J = 7.5 Hz, 2H, $SO_2CH_2CH_2CO$), 2.90 (t, J = 7.5 Hz, 2H, $SO_2CH_2CH_2CO$), 2.29 (s, 3H, $ArCH_3$); **^{13}C NMR** (151 MHz, DMSO-d_6) δ_C 168.6, 157.6, 148.5, 145.4, 142.9, 142.4 (q, J = 37.5 Hz), 139.2, 138.2, 131.4, 129.5, 129.2, 128.7, 126.2, 126.1, 125.2, 123.6, 121.6, 121.2 (q, J = 269.0 Hz), 120.6, 106.3 (q, J = 2.0 Hz), 50.1, 28.6, 20.8; **^{19}F NMR** (565 MHz, DMSO-d_6) δ_F -60.96; **LRMS** (ESI, *m/z*) $[M+H]^+$ 571.1, $[M+Na]^+$ 593.1; **HRMS** (ESI, *m/z*) calculated for $(C_{27}H_{22}O_3N_4F_3S_2)^+$ 571.1080 $[M+H]^+$, found 571.1078; **IR** ($CHCl_3$, ν_{max}/cm^{-1}) 2922, 1695, 1598, 1553, 1474, 1272, 1166, 1148, 1132, 977, 759; **R$_f$** 0.28 (petrol:EtOAc, 70:30).

5-(*p*-Tolyl)-3-(trifluoromethyl)-1-(4-((4-(trifluoromethyl)phenethyl)sulfonyl)phenyl)-1*H*-pyrazole (2au)

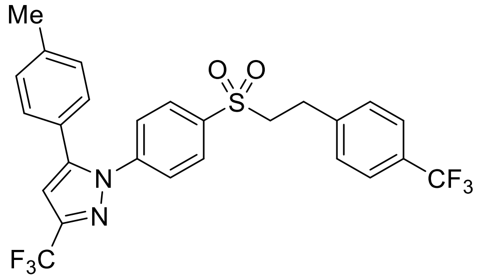

Prepared according to general procedure D, using *N*-sulfonylimine **3a** (100 mg, 0.20 mmol, 1.0 equiv.) and 4-trifluoromethylstyrene (74 μL, 0.50 mmol, 2.5 equiv.) in acetonitrile (4 mL) for 12 h. Purification by silica gel column chromatography (*n*-pentane:EtOAc, 95:5 to 80:20) afforded the title compound (32 mg, 30%) as a yellow oil. **^{1}H NMR** (600 MHz, $CDCl_3$) δ_H 7.91 (d, J = 8.5 Hz, 2H, Ar*H*), 7.54 (d, J = 8.5 Hz, 4H, Ar*H*), 7.24 (d, J = 8.0 Hz, 2H, Ar*H*), 7.18 (d, J = 8.0 Hz, 2H, Ar*H*), 7.11 (d, J = 8.0 Hz, 2H, Ar*H*), 6.75 (s, 1H, Ar*H*), 3.40 – 3.33 (m, 2H, $SO_2CH_2CH_2Ar$), 3.13 – 3.09 (m, 2H, $SO_2CH_2CH_2Ar$), 2.39 (s, 3H, $ArCH_3$); **^{13}C NMR** (151 MHz, $CDCl_3$) δ_C 145.5, 144.6 (q, J = 38.5 Hz), 143.9, 141.4, 140.1, 138.2, 130.0, 129.7 (q, J = 32.5 Hz), 129.4, 128.9, 128.8, 126.0 (q, J = 4.0 Hz), 125.9, 125.8, 124.1 (q, J = 272.0 Hz), 121.1 (q, J = 269.0 Hz), 106.7 (q, J = 2.0 Hz), 57.2, 28.7, 21.5; **^{19}F NMR** (565 MHz, $CDCl_3$) δ_F -62.54, -62.63; **LRMS** (ESI, *m/z*) $[M+H]^+$ 539.1, $[M+Na]^+$ 561.1; **HRMS** (ESI, *m/z*) calculated for $(C_{26}H_{21}O_2N_2F_6S)^+$ 539.1222 $[M+H]^+$, found 539.1222; **IR** ($CHCl_3$, ν_{max}/cm^{-1}) 3020, 2917, 2849, 1326, 1216, 753; **R$_f$** 0.40 (petrol:EtOAc, 80:20).

3.6. Photochemical Functionalisation with Neutral Alkenes

General Procedure E

(excess alkene):

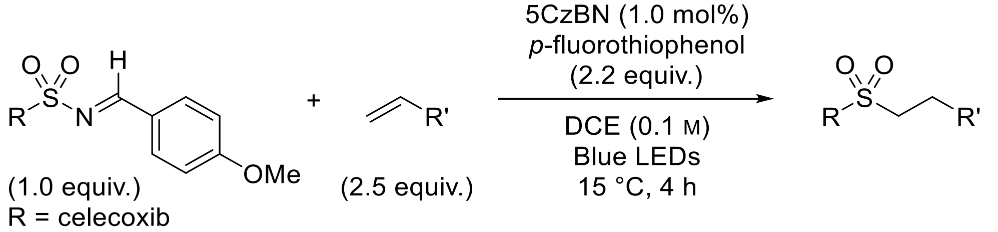

N-sulfonylimine (0.2 mmol, 1.0 equiv.) and 5CzBN (1.8 mg, 1.0 mol%) were added to an 8.0 mL screw capped vial equipped with a rubber septum. The vial was evacuated and back filled with nitrogen three times, followed by the addition of *p*-fluorothiophenol (47 µL, 0.44 mmol, 2.2 equiv.), the appropriate alkene (0.5 mmol, 2.5 equiv.) and 1,2-dichloroethane (2 mL, 0.1 M). The vial was then placed in a glass tempering beaker connected to a flow of water, the vial was subsequently submerged in water and irradiated with two EvoluChem™ 450 nm LED spotlights. After 4 h the reaction was diluted with $CHCl_3$ (4 mL), and the solvent removed under reduced pressure, followed by purification by flash column chromatography.

General Procedure F

(**excess** ***N***-sulfonylimine):

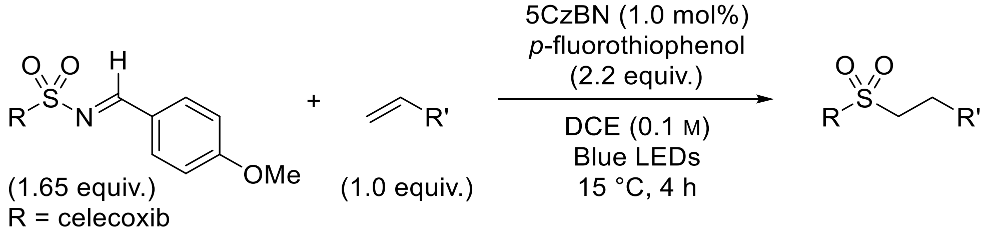

N-sulfonylimine (0.33 mmol, 1.65 equiv.) and 5CzBN (1.8 mg, 1.0 mol%) were added to an 8.0 mL screw capped vial equipped with a rubber septum. The vial was evacuated and back filled with nitrogen three times, followed by the addition of *p*-fluorothiophenol (47 µL, 0.44 mmol, 2.2 equiv.), the appropriate alkene (0.2 mmol, 1.0 equiv.) and 1,2-dichloroethane (2 mL, 0.1 M). The vial was then placed in a glass tempering beaker connected to a flow of water, the vial was subsequently submerged in water and irradiated with two EvoluChem™ 450 nm LED spotlights. After 4 h the reaction was diluted with $CHCl_3$ (4 mL), and the solvent removed under reduced pressure, followed by purification by flash column chromatography.

1-(4-((4-Phenylbutyl)sulfonyl)phenyl)-5-(*p*-tolyl)-3-(trifluoromethyl)-1*H*-pyrazole (4a)

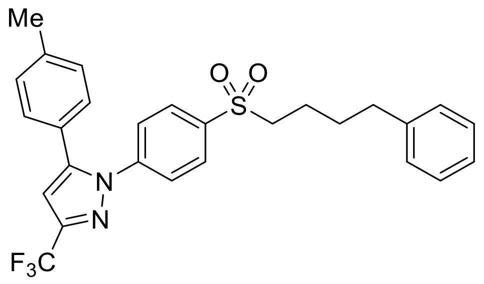

Prepared according to general procedure E, using *N*-sulfonylimine **3a** (100 mg, 0.20 mmol, 1.0 equiv.) and 4-phenyl-1-butene (75 μL, 0.50 mmol, 2.5 equiv.). Purification by silica gel column chromatography (*n*-pentane:EtOAc, 95:5 to 85:15) afforded the title compound (65 mg, 65%) as a yellow oil. **^{1}H NMR** (600 MHz, $CDCl_3$) δ_H 7.86 (d, *J* = 8.5 Hz, 2H, Ar*H*), 7.51 (d, *J* = 8.5 Hz, 2H, Ar*H*), 7.28 – 7.23 (m, 2H, Ar*H*), 7.19 – 7.15 (m, 3H, Ar*H*), 7.10 (app. d, *J* = 8.0 Hz, 4H, Ar*H*), 6.76 (s, 1H, Ar*H*), 3.11 – 3.06 (m, 2H, $SO_2CH_2(CH_2)_3Ph$), 2.59 (t, *J* = 7.0 Hz, 2H, $SO_2(CH_2)_3CH_2Ph$), 2.38 (s, 3H, $ArCH_3$), 1.77 – 1.66 (m, 4H, $SO_2CH_2(CH_2)_2CH_2Ph$); **^{13}C NMR** (151 MHz, $CDCl_3$) δ_C 145.4, 144.3 (q, *J* = 38.5 Hz), 143.4, 141.1, 139.9, 138.4, 129.8, 129.2, 128.7, 128.5, 128.3, 126.1, 125.7, 125.6, 121.0 (q, *J* = 269.0 Hz), 106.4 (q, *J* = 2.0 Hz), 56.1, 35.3, 30.0, 22.3, 21.3; **^{19}F NMR** (377 MHz, $CDCl_3$) δ_F -62.48; **LRMS** (ESI, *m/z*) $[M+H]^+$ 499.1, $[M+Na]^+$ 521.1; **HRMS** (ESI, *m/z*) calculated for $(C_{27}H_{26}O_2N_2F_3S)^+$ 499.1662 $[M+H]^+$, found 499.1661; **IR** ($CHCl_3$, ν_{max}/cm^{-1}) 3027, 2927, 1596, 1472, 1373, 1237, 1148, 1096, 975, 753, 700; **R_f** 0.34 (petrol:EtOAc, 80:20).

The reaction was also performed according to general procedure F, using *N*-sulfonylimine **3a** (165 mg, 0.33 mmol, 1.65 equiv.) and 4-phenyl-1-butene (30 μL, 0.20 mmol, 1.0 equiv.). Purification by silica gel column chromatography (*n*-pentane:EtOAc, 95:5 to 85:15) afforded the title compound (60 mg, 60%) as a yellow oil.

1-(4-((5-(Phenylthio)pentyl)sulfonyl)phenyl)-5-(*p*-tolyl)-3-(trifluoromethyl)-1*H*-pyrazole (4b)

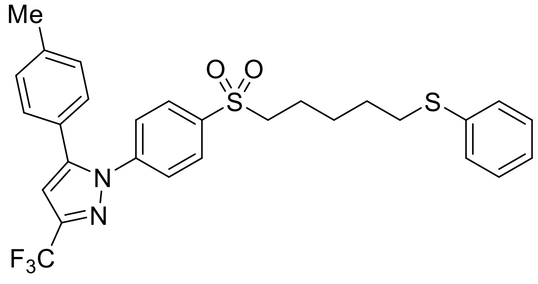

Prepared according to general procedure E, using *N*-sulfonylimine **3a** (100 mg, 0.20 mmol, 1.0 equiv.) and pent-4-en-1-yl phenyl sulfide (**8g**) (89 mg, 0.50 mmol, 2.5 equiv.). Purification by silica gel column chromatography (*n*-pentane:EtOAc, 95:5 to 85:15) afforded the title compound (76 mg, 70%) as a yellow oil. **^{1}H NMR** (600 MHz, $CDCl_3$) δ_H 7.87 (d, *J* = 8.5 Hz, 2H, Ar*H*), 7.52 (d, *J* = 8.5 Hz, 2H, Ar*H*), 7.31 – 7.25 (obs.

m, 4H, Ar*H*), 7.21 – 7.14 (m, 3H, Ar*H*), 7.10 (d, *J* = 8.0 Hz, 2H, Ar*H*), 6.75 (s, 1H, Ar*H*), 3.08 – 3.04 (m, 2H, $SO_2CH_2(CH_2)_4SPh$), 2.88 (t, *J* = 7.0 Hz, 2H, $SO_2(CH_2)_4CH_2SPh$), 2.38 (s, 3H, ArCH_3), 1.74 – 1.67 (m, 2H, $SO_2CH_2CH_2(CH_2)_3SPh$), 1.65 – 1.58 (m, 2H, $SO_2(CH_2)_3CH_2CH_2SPh$), 1.54 – 1.48 (m, 2H, $SO_2(CH_2)_2CH_2(CH_2)_2SPh$); **^{13}C NMR** (151 MHz, $CDCl_3$) δ_C 145.5, 144.5 (q, *J* = 38.5 Hz), 143.6, 140.1, 138.5, 136.4, 130.0, 129.4, 129.3, 129.1, 128.9, 126.2, 125.8 (2C), 121.2 (q, *J* = 269.0 Hz), 106.6 (q, *J* = 2.0 Hz), 56.3, 33.4, 28.6, 27.4, 22.5, 21.5; **^{19}F NMR** (565 MHz, $CDCl_3$) δ_F -62.50; **LRMS** (ESI, *m/z*) $[M+H]^+$ 545.1, $[M+Na]^+$ 567.1; **HRMS** (ESI, *m/z*) calculated for $(C_{28}H_{28}O_2N_2F_3S_2)^+$ 545.1539 $[M+H]^+$, found 545.1536; **IR** ($CHCl_3$, ν_{max}/cm^{-1}) 3060, 2926, 2864, 1595, 1496, 1237, 1147, 1096, 975, 742; **R$_f$** 0.36 (petrol:EtOAc, 80:20).

1-(4-((3-Phenoxypropyl)sulfonyl)phenyl)-5-(*p*-tolyl)-3-(trifluoromethyl)-1*H*-pyrazole (4c)

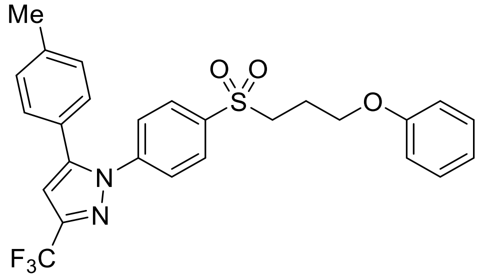

Prepared according to general procedure E, using *N*-sulfonylimine **3a** (100 mg, 0.20 mmol, 1.0 equiv.) and allyl phenyl ether (69 µL, 0.50 mmol, 2.5 equiv.). Purification by silica gel column chromatography (*n*-pentane:EtOAc, 95:5 to 85:15) afforded the title compound (67 mg, 67%) as a yellow solid. **mp** (*n*-pentane:EtOAc) 71 – 73 °C; **^{1}H NMR** (600 MHz, $CDCl_3$) δ_H 7.92 (d, *J* = 8.5 Hz, 2H, Ar*H*), 7.54 (d, *J* = 8.5 Hz, 2H, Ar*H*), 7.28 – 7.24 (m, 2H, Ar*H*), 7.18 (d, *J* = 8.0 Hz, 2H, Ar*H*), 7.11 (d, *J* = 8.0 Hz, 2H, Ar*H*), 6.95 (t, *J* = 7.5 Hz, 1H, Ar*H*), 6.83 (d, *J* = 7.5 Hz, 2H, Ar*H*), 6.75 (s, 1H, Ar*H*), 4.02 (t, *J* = 6.0 Hz, 2H, $SO_2(CH_2)_2CH_2O$), 3.35 – 3.30 (m, 2H, $SO_2CH_2(CH_2)_2O$), 2.38 (s, 3H, ArCH_3), 2.24 – 2.18 (m, 2H, $SO_2CH_2CH_2CH_2O$); **^{13}C NMR** (151 MHz, $CDCl_3$) δ_C 158.4, 145.5, 144.5 (q, *J* = 38.5 Hz), 143.7, 140.1, 138.5, 130.0, 129.7, 129.3, 128.9, 125.8 (2C), 121.4, 121.2 (q, *J* = 269.0 Hz), 114.6, 106.6 (q, *J* = 2.0 Hz), 65.4, 53.5, 23.2, 21.5; **^{19}F NMR** (377 MHz, $CDCl_3$) δ_F -62.49; **LRMS** (ESI, *m/z*) $[M+H]^+$ 501.1, $[M+Na]^+$ 523.1; **HRMS** (ESI, *m/z*) calculated for $(C_{26}H_{24}O_3N_2F_3S)^+$ 501.1454 $[M+H]^+$, found 501.1453; **IR** ($CHCl_3$, ν_{max}/cm^{-1}) 2925, 1598, 1498, 1472, 1237, 1143, 976, 756, 683; **R$_f$** 0.36 (petrol:EtOAc, 75:25).

1-(4-((3,3-Dimethylbutyl)sulfonyl)phenyl)-5-(*p*-tolyl)-3-(trifluoromethyl)-1*H*-pyrazole (4d)

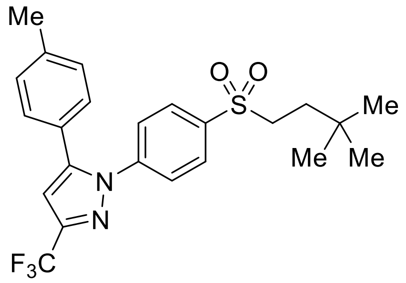

Prepared according to general procedure E, using *N*-sulfonylimine **3a** (100 mg, 0.20 mmol, 1.0 equiv.) and 3,3-dimethylbut-1-ene (65 μL, 0.50 mmol, 2.5 equiv.). Purification by silica gel column chromatography (*n*-pentane:EtOAc, 95:5 to 90:10) afforded the title compound (50 mg, 56%) as a yellow solid. **mp** (*n*-pentane:EtOAc) 79 – 81 °C; **^{1}H NMR** (600 MHz, $CDCl_3$) δ_H 7.89 (d, *J* = 8.5 Hz, 2H, Ar*H*), 7.53 (d, *J* = 8.5 Hz, 2H, Ar*H*), 7.17 (d, *J* = 8.0 Hz, 2H, Ar*H*), 7.10 (d, *J* = 8.0 Hz, 2H, Ar*H*), 6.75 (s, 1H, Ar*H*), 3.09 – 3.03 (m, 2H, $SO_2CH_2CH_2C(CH_3)_3$), 2.38 (s, 3H, ArCH_3), 1.57 – 1.52 (obs. m, 2H, $SO_2CH_2CH_2C(CH_3)_3$), 0.86 (s, 9H, $CH_2C(CH_3)_3$); **^{13}C NMR** (151 MHz, $CDCl_3$) δ_C 145.5, 144.5 (q, *J* = 38.5 Hz), 143.5, 140.1, 138.5, 130.0, 129.4, 128.9, 125.8 (2C), 121.2 (q, *J* = 269.5 Hz), 106.6 (q, *J* = 2.0 Hz), 53.2, 36.0, 30.2, 29.0, 21.5; **^{19}F NMR** (377 MHz, $CDCl_3$) δ_F -62.50; **LRMS** (ESI, *m/z*) $[M+H]^+$ 451.0, $[M+Na]^+$ 473.2; **HRMS** (ESI, *m/z*) calculated for $(C_{23}H_{26}O_2N_2F_3S)^+$ 451.1662 $[M+H]^+$, found 451.1660; **IR** ($CHCl_3$, ν_{max}/cm^{-1}) 2945, 2864, 1597, 1473, 1370, 1237, 1151, 976, 807; **R_f** 0.63 (petrol:EtOAc, 80:20).

***tert*-Butyl (3-((4-(5-(*p*-tolyl)-3-(trifluoromethyl)-1*H*-pyrazol-1-yl)phenyl)sulfonyl)propyl)-carbamate (4e)**

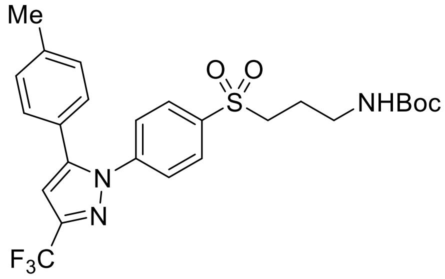

Prepared according to general procedure E, using *N*-sulfonylimine **3a** (100 mg, 0.20 mmol, 1.0 equiv.) and *N*-Boc-allylamine (84 μL, 0.50 mmol, 2.5 equiv.). Purification by silica gel column chromatography (*n*-pentane:EtOAc, 85:15 to 65:35) afforded the title compound (45 mg, 43%) as a colorless oil. **^{1}H NMR** (600 MHz, $CDCl_3$) δ_H 7.88 (d, *J* = 8.5 Hz, 2H, Ar*H*), 7.53 (d, *J* = 8.5 Hz, 2H, Ar*H*), 7.18 (d, *J* = 8.0 Hz, 2H, Ar*H*), 7.11 (d, *J* = 8.0 Hz, 2H, Ar*H*), 6.74 (s, 1H, Ar*H*), 4.67 (br. s, 1H, $CH_2NHC(CH_3)_3$), 3.23 (q, *J* = 6.5 Hz, 2H, $SO_2(CH_2)_2CH_2NH$), 3.14 (t, *J* = 7.5 Hz, 2H, $SO_2CH_2(CH_2)_2NH$), 2.38 (s, 3H, ArCH_3), 1.94 – 1.88 (m, 2H, $SO_2CH_2CH_2CH_2NH$), 1.41 (s, 9H, $NHC(CH_3)_3$); **^{13}C NMR** (151 MHz, $CDCl_3$) δ_C 156.1, 145.5, 144.4

(q, J = 39.0 Hz), 143.7, 140.1, 138.5, 130.0, 129.3, 128.9, 125.8 (2C), 121.1 (q, J = 269.0 Hz), 106.6 (q, J = 2.0 Hz), 79.9, 53.9, 38.9, 28.5, 23.8, 21.5; **^{19}F NMR** (377 MHz, $CDCl_3$) δ_F -62.51; **LRMS** (ESI, *m/z*) $[M+H]^+$ 524.1, $[M+Na]^+$ 546.1; **HRMS** (ESI, *m/z*) calculated for $(C_{25}H_{28}O_4N_3F_3NaS)^+$ 546.1645 $[M+Na]^+$, found 546.1642; **IR** ($CHCl_3$, ν_{max}/cm^{-1}) 3385, 2980, 2917, 1702, 1472, 1369, 1237, 1144, 975, 764; **R$_f$** 0.47 (petrol:EtOAc, 50:50).

1-(4-((4-(Benzyloxy)-2-methylbutyl)sulfonyl)phenyl)-5-(*p*-tolyl)-3-(trifluoromethyl)-1*H*-pyrazole (4f)

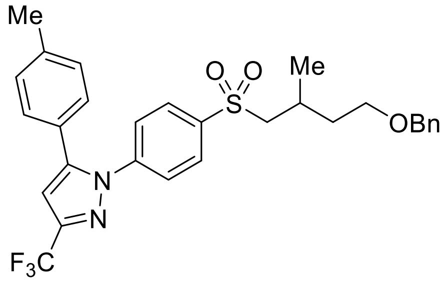

Prepared according to general procedure E, using *N*-sulfonylimine **3a** (100 mg, 0.20 mmol, 1.0 equiv.) and benzyl-protected 3-methylbut-3-en-1-ol (**8h**) (88 mg, 0.50 mmol, 2.5 equiv.). Purification by silica gel column chromatography (*n*-pentane:EtOAc, 95:5 to 85:15) afforded the title compound (61 mg, 56%) as a slightly yellow oil. **^{1}H NMR** (600 MHz, $CDCl_3$) δ_H 7.84 (d, J = 8.5 Hz, 2H, Ar*H*), 7.44 (d, J = 8.5 Hz, 2H, Ar*H*), 7.32 – 7.28 (m, 2H, Ar*H*), 7.28 – 7.24 (m, 3H, Ar*H*), 7.17 (d, J = 8.0 Hz, 2H, Ar*H*), 7.09 (d, J = 8.0 Hz, 2H, Ar*H*), 6.75 (s, 1H, Ar*H*), 4.43 (d, J = 12.0 Hz, 1H, OCH_AH_BPh), 4.41 (d, J = 12.0 Hz, 1H, OCH_AH_BPh), 3.52 – 3.43 (m, 2H, $CHCH_2CH_2O$), 3.26 (dd, J = 14.5, 4.5 Hz, 1H, $SO_2CH_AH_BCH$), 2.95 (dd, J = 14.5, 8.0 Hz, 1H, $SO_2CH_AH_BCH$), 2.38 (s, 3H, $ArCH_3$), 2.30 – 2.22 (m, 1H, $SO_2CH_2CH(CH_3)CH_2$), 1.78 (ddt, J = 11.5, 7.5, 5.5 Hz, 1H, $CHCH_AH_BCH_2O$), 1.60 – 1.53 (obs. m, 1H, $CHCH_AH_BCH_2O$), 1.09 (d, J = 6.5 Hz, 3H, $SO_2CH_2CH(CH_3)CH_2$); **^{13}C NMR** (151 MHz, $CDCl_3$) δ_C 145.5, 144.4 (q, J = 38.5 Hz), 143.4, 140.0, 139.5, 138.3, 129.9, 129.1, 128.9, 128.5, 127.8 (2C), 125.8, 125.7, 121.2 (q, J = 269.0 Hz), 106.5 (q, J = 2.0 Hz), 73.2, 67.8, 62.5, 36.3, 26.8, 21.5, 20.1; **^{19}F NMR** (377 MHz, $CDCl_3$) δ_F -62.47; **LRMS** (ESI, *m/z*) $[M+H]^+$ 543.1, $[M+Na]^+$ 565.1; **HRMS** (ESI, *m/z*) calculated for $(C_{29}H_{30}O_3N_2F_3S)^+$ 543.1924 $[M+H]^+$, found 543.1924; **IR** ($CHCl_3$, ν_{max}/cm^{-1}) 2917, 2850, 1596, 1472, 1373, 1237, 1138, 1097, 975, 741; **R$_f$** 0.50 (petrol:EtOAc, 75:25).

5-((4-(5-(*p*-Tolyl)-3-(trifluoromethyl)-1*H*-pyrazol-1-yl)phenyl)sulfonyl)hexan-1-ol (4g) & 4-((4-(5-(*p*-tolyl)-3-(trifluoromethyl)-1*H*-pyrazol-1-yl)phenyl)-sulfonyl)hexan-1-ol (4g')

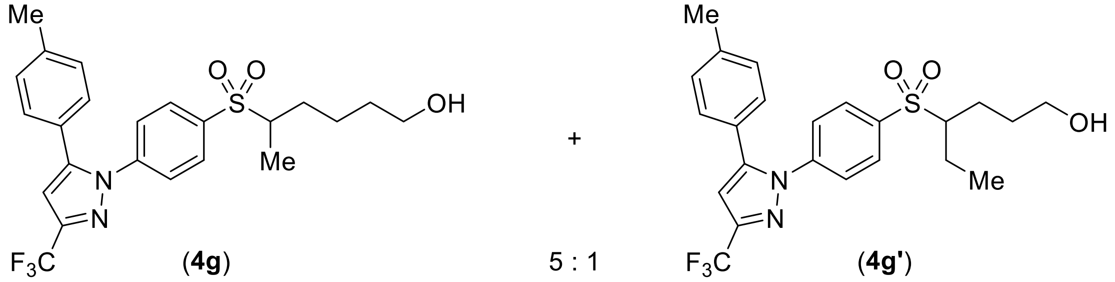

Prepared according to general procedure E, using *N*-sulfonylimine **3a** (100 mg, 0.20 mmol, 1.0 equiv.) and 4-hexen-1-ol (59 µL, 0.50 mmol, 2.5 equiv.). The regioisomers ratio was determined by analysis of ^{1}H NMR spectra of the crude reaction mixture and was found to be 5:1. Purification by silica gel column chromatography (*n*-pentane:EtOAc, 85:15 to 55:45) afforded regioisomer (**4g**) (39 mg, 42%) and regioisomer (**4g'**) (10 mg, 11%) both as yellow viscous oils.

Regioisomer (4g): **^{1}H NMR** (600 MHz, $CDCl_3$) δ_H 7.86 (d, *J* = 8.5 Hz, 2H, Ar*H*), 7.52 (d, *J* = 8.5 Hz, 2H, Ar*H*), 7.18 (d, *J* = 7.5 Hz, 2H, Ar*H*), 7.10 (d, *J* = 8.0 Hz, 2H, Ar*H*), 6.75 (s, 1H, Ar*H*), 3.66 – 3.60 (m, 2H, CH_2CH_2OH), 3.07 – 2.99 (m, 1H, $SO_2CH(CH_3)CH_2$), 2.38 (s, 3H, $ArCH_3$), 2.00 – 1.92 (m, 1H, $CH(CH_3)CH_AH_BCH_2$), 1.56 – 1.33 (m, 5H, $CH_AH_BCH_2CH_2$ CH_2OH), 1.26 (d, *J* = 7.0 Hz, 3H, $SO_2CH(CH_3)$); **^{13}C NMR** (151 MHz, $CDCl_3$) δ_C 145.6, 144.4 (q, *J* = 38.5 Hz), 143.5, 140.1, 136.8, 130.3, 130.0, 128.9, 125.7 (2C), 121.2 (q, *J* = 269.0 Hz), 106.6 (q, *J* = 2.0 Hz), 62.4, 60.4, 32.3, 29.1, 23.1, 21.5, 13.3; **^{19}F NMR** (377 MHz, $CDCl_3$) δ_F -62.49; **LRMS** (ESI, *m/z*) $[M+Na]^+$ 489.0; **HRMS** (ESI, *m/z*) calculated for $(C_{23}H_{26}O_3N_2F_3S)^+$ 467.1611 $[M+H]^+$, found 467.1606; **IR** ($CHCl_3$, ν_{max}/cm^{-1}) 3407, 2917, 2849, 1597, 1471, 1237, 1137, 976, 766, 668; **R_f** 0.47 (petrol:EtOAc, 40:60).

Regioisomer (4g'): **^{1}H NMR** (600 MHz, $CDCl_3$) δ_H 7.87 (d, *J* = 8.5 Hz, 2H, Ar*H*), 7.52 (d, *J* = 8.5 Hz, 2H, Ar*H*), 7.17 (d, *J* = 8.0 Hz, 2H, Ar*H*), 7.10 (d, *J* = 8.0 Hz, 2H, Ar*H*), 6.75 (s, 1H, Ar*H*), 3.64 (t, *J* = 6.0 Hz, 2H, CH_2CH_2OH), 2.96 – 2.90 (m, 1H, $SO_2CH(Et)CH_2$), 2.38 (s, 3H, $ArCH_3$), 1.98 – 1.90 (m, 1H, Alk*H*), 1.88 – 1.79 (m, 1H, Alk*H*), 1.79 – 1.70 (m, 2H, Alk*H*), 1.68 – 1.60 (m, 2H, $CHCH_2CH_2CH_2OH$), 0.99 (t, *J* = 7.5 Hz, 3H, $CHCH_2CH_3$); **^{13}C NMR** (151 MHz, $CDCl_3$) δ_C 145.6, 144.4 (q, *J* = 38.5 Hz), 143.5, 140.1, 137.6, 130.1, 130.0, 128.9, 125.8, 125.7, 121.2 (q, *J* = 269.0 Hz), 106.6 (q, *J* = 2.0 Hz), 65.8, 62.4, 29.9, 23.8, 21.5, 21.4, 11.2; **^{19}F NMR** (377 MHz, $CDCl_3$) δ_F -62.50; **LRMS** (ESI, *m/z*) $[M+Na]^+$ 489.2; **HRMS** (ESI, *m/z*) calculated for $(C_{23}H_{26}O_3N_2F_3S)^+$ 467.1611 $[M+H]^+$, found 467.1610; **IR** ($CHCl_3$, ν_{max}/cm^{-1}) 3380, 2916, 2849, 1597, 1237, 1140, 976, 763, 663; **R_f** 0.58 (petrol:EtOAc, 40:60).

1-(4-((5-Bromopentyl)sulfonyl)phenyl)-5-(*p*-tolyl)-3-(trifluoromethyl)-1*H*-pyrazole (4h)

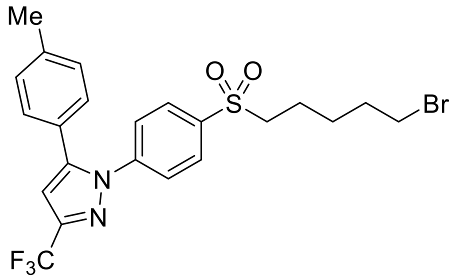

Prepared according to general procedure E, using *N*-sulfonylimine **3a** (100 mg, 0.20 mmol, 1.0 equiv.) and 5-bromo-1-pentene (59 µL, 0.50 mmol, 2.5 equiv.). Purification by silica gel column chromatography (*n*-pentane:EtOAc, 95:5 to 80:20) afforded the title compound (64 mg, 62%) as a sticky yellow oil. **^{1}H NMR** (600 MHz, $CDCl_3$) δ_H 7.89 (d, *J* = 8.5 Hz, 2H, Ar*H*), 7.53 (d, *J* = 8.5 Hz, 2H, Ar*H*), 7.18 (d, *J* = 8.0 Hz, 2H, Ar*H*), 7.11 (d, *J* = 8.0 Hz, 2H, Ar*H*), 6.75 (s, 1H, Ar*H*), 3.37 (t, *J* = 6.5 Hz, 2H, $(CH_2)_4C\mathit{H}_2Br$), 3.12 – 3.07 (m, 2H, $SO_2C\mathit{H}_2$), 2.39 (s, 3H, $ArC\mathit{H}_3$), 1.87 – 1.81 (m, 2H, $(CH_2)_3C\mathit{H}_2CH_2Br$), 1.77 – 1.71 (m, 2H, $CH_2C\mathit{H}_2(CH_2)_3Br$), 1.58 – 1.51 (obs. m, 2H, $C\mathit{H}_2(CH_2)_2Br$); **^{13}C NMR** (151 MHz, $CDCl_3$) δ_C 145.5, 144.5 (q, *J* = 38.5 Hz), 143.7, 140.1, 138.5, 130.0, 129.3, 128.9, 125.8 (2C), 121.2 (q, *J* = 269.0 Hz), 106.6 (q, *J* = 2.0 Hz), 56.2, 33.0, 32.1, 26.9, 22.1, 21.5; **^{19}F NMR** (377 MHz, $CDCl_3$) δ_F -62.51; **LRMS** (ESI, *m/z*) $[^{79}BrM+H]^+$ 515.0, $[^{81}BrM+H]^+$ 517.0; **HRMS** (ESI, *m/z*) calculated for $(C_{22}H_{23}O_2N_2{}^{79}BrF_3S)^+$ 515.0610 $[^{79}BrM+H]^+$, found 515.0610, calculated for $(C_{22}H_{23}O_2N_2{}^{81}BrF_3S)^+$ 517.0590 $[^{81}BrM+H]^+$, found 517.0590; **IR** ($CHCl_3$, ν_{max}/cm^{-1}) 2922, 2849, 1591, 1471, 1236, 1147, 976; **R_f** 0.43 (petrol:EtOAc, 80:20).

3.7. Photochemical Functionalisation *via* Sulfinate Salts

1-Methyl-4-((4-(trifluoromethyl)benzyl)sulfonyl)benzene (6)

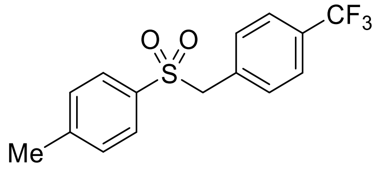

N-sulfonylimine **1a** (58 mg, 0.20 mmol, 1.0 equiv.), potassium carbonate (28 mg, 0.20 mmol, 1.0 equiv.) and 5CzBN (0.9 mg, 0.001 mmol, 0.5 mol%) were added to an 8.0 mL vial equipped with a rubber septum. The vial was evacuated and back filled with nitrogen three times, followed by the addition of tris(trimethylsilyl)silane (93 µL, 0.30 mmol, 1.5 equiv.), toluene (1.0 mL) and water (1.0 mL). The vial was placed in a glass tempering beaker connected to a flow of water, and was then subsequently submerged in water and irradiated with two EvoluChem™ 450 nm LED spotlights. After 4 h the reaction was removed from the reactor and 4-(trifluoromethyl)benzyl bromide (72 mg, 0.30 mmol, 1.5 equiv.) and TBAB (13 mg, 0.04 mmol, 20 mol%), were added. The reaction was sealed with a cap, then heated to 100 °C and stirred for 24 h. The reaction was

cooled to room temperature and diluted with EtOAc (2 mL), and the aqueous and organic phases were separated. The aqueous phase was further extracted with EtOAc (3 × 2 mL), and the organic extracts combined, dried with sodium sulfate, filtered, and the solvent removed under reduced pressure. Purification by flash column chromatography (*n*-hexane:EtOAc, 80:20), afforded the title compound (59 mg, 94%) as a yellow solid. **mp** (*n*-hexane:EtOAc) 213 – 215 °C; **^{1}H NMR** (400 MHz, DMSO-d_6) δ_H 7.69 (d, *J* = 8.0 Hz, 2H, Ar*H*), 7.61 (d, *J* = 8.5 Hz, 2H, Ar*H*), 7.41 (d, *J* = 8.0 Hz, 2H, Ar*H*), 7.39 (d, *J* = 8.5 Hz, 2H, Ar*H*), 4.80 (s, 2H, SO_2CH_2Ar), 2.40 (s, 3H, ArCH_3); **^{13}C NMR** (101 MHz, DMSO-d_6) δ_C 144.5, 135.4, 133.6 (d, *J* = 1.5 Hz), 131.8, 129.7, 128.8 (d, *J* = 32.0 Hz), 128.0, 125.1 (d, *J* = 4,0 Hz), 124.1 (d, *J* = 272.0 Hz), 60.1, 21.1; **^{19}F NMR** (377 MHz, DMSO-d_6) δ_F -61.07; **LRMS** (ESI, *m/z*) $[M+Na]^+$ 337.0. The analytical data is consistent with the literature.[34]

4-Methylbenzenesulfonyl fluoride (7)

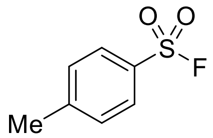

N-sulfonylimine **1a** (116 mg, 0.40 mmol, 1.0 equiv.), potassium carbonate (56 mg, 0.40 mmol, 1.0 equiv.) and 5CzBN (1.8 mg, 0.002 mmol, 0.5 mol%) were added to an 8.0 mL vial equipped with a rubber septum. The vial was evacuated and back filled with nitrogen three times, followed by the addition of tris(trimethylsilyl)silane (186 µL, 0.60 mmol, 1.5 equiv.), toluene (2.0 mL) and water (2.0 mL). The vial was placed in a glass tempering beaker connected to a flow of water, and was then subsequently submerged in water and irradiated with two EvoluChem™ 450 nm LED spotlights. After 4 h the reaction was removed from the reactor and more potassium carbonate (62 mg, 0.44 mmol, 1.1 equiv.) was added. The resulting mixture was concentrated under reduced pressure and the obtained crude dissolved in THF (2.0 mL). NFSI (190 mg, 0.60 mmol, 1.5 equiv.) and water (0.2 mL) were added, and the resulting mixture stirred at room temperature for 12 h. The reaction was then diluted with water (5 mL) and extracted with CH_2Cl_2 (3 × 5 mL). The combined organic extracts were dried with magnesium sulfate, filtered, and the solvent removed under reduced pressure. Purification by silica gel column chromatography (*n*-pentane:EtOAc, 90:10) afforded the title compound (47 mg, 68%) as a colorless oil. **^{1}H NMR** (400 MHz, $CDCl_3$) δ_H 7.90 (d, *J* = 8.5 Hz, 2H, Ar*H*), 7.42 (d, *J* = 8.0 Hz, 2H, Ar*H*), 2.49 (s, 3H, ArCH_3); **^{13}C NMR** (101 MHz, $CDCl_3$) δ_C 147.2, 130.4, 130.3 (d, *J* = 24.5 Hz), 128.6, 22.0; **^{19}F NMR** (377 MHz, $CDCl_3$) δ_F 66.25; **HRMS** (EI, *m/z*) calculated for $(C_7H_7O_2FS)^+$ 174.0145 $[M]^+$, found 174.0151; **R_f** 0.30 (petrol:EtOAc, 90:10). The analytical data is consistent with the literature.[35]

Sodium 4-methylbenzenesulfinate (5-Na)

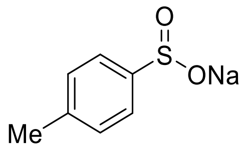

N-sulfonylimine **1a** (116 mg, 0.40 mmol, 1.0 equiv.), potassium carbonate (56 mg, 0.40 mmol, 1.0 equiv.) and 5CzBN (1.8 mg, 0.002 mmol, 0.5 mol%) were added to an 8.0 mL vial equipped with a rubber septum. The vial was evacuated and back filled with nitrogen three times, followed by the addition of tris(trimethylsilyl)silane (186 µL, 0.60 mmol, 1.5 equiv.), toluene (2.0 mL) and water (2.0 mL). The vial was placed in a glass tempering beaker connected to a flow of water, and was then subsequently submerged in water and irradiated with two EvoluChem™ 450 nm LED spotlights. After 4 h the reaction was removed from the reactor and 2 M aqueous H_3PO_4 (5 mL) and brine (12 mL) were added to the mixture. The aqueous layer was then extracted with Et_2O (2 × 10 mL). The combined organic phases were washed with further 2 M aqueous H_3PO_4 (5 mL) and brine (12 mL), and then extracted with a 10% aqueous Na_2CO_3 solution (3 × 10 mL). The aqueous layer was concentrated under reduced pressure by forming an azeotrope with toluene. EtOH (20 mL) was added, and the mixture refluxed for 10 mins. The mixture was then cooled to 0 °C, filtered through a P4 frit and the residue washed with cold EtOH. The filtrate was then concentrated under reduced pressure to afford the title compound (59 mg, 83%) as a white solid. **^{1}H NMR** (400 MHz, MeOD-*d*$_4$) δ_H 7.54 (d, *J* = 8.0 Hz, 2H, Ar*H*), 7.23 (d, *J* = 8.0 Hz, 2H, Ar*H*), 2.36 (s, 3H, ArC*H*$_3$); **^{13}C NMR** (101 MHz, MeOD-*d*$_4$) δ_C 154.3, 140.6, 130.0, 125.2, 21.3; **LRMS** (ESI, *m/z*) [M-Na]$^-$ 155.1. The analytical data is consistent with the literature.[36]

3.8. Telescoped Reaction from Sulfonamide

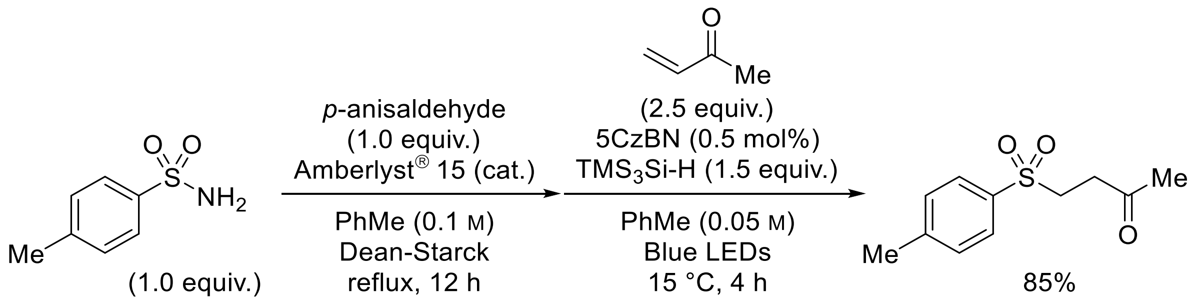

A 10 ml round bottom flask was charged with *p*-toluenesulfonamide (68.5 mg, 0.4 mmol, 1.0 equiv.), *p*-anisaldehyde (49 µL, 0.4 mmol, 1.0 equiv.) and amberlyst® 15 (20 mg). Toluene (4 mL, 0.1 M) was then added and the resulting mixture refluxed with a Dean-Starck until TLC shows complete conversion (around 12 h). The resulting *N*-sulfonylimine solution was then cannulated into a vial charged with a Teflon-coated stirrer bar, 5CzBN (2 mg, 0.5 mol%), methyl vinyl ketone (82 µL, 1.0 mmol, 2.5 equiv.) and tris(trimethylsilyl)silane (185 µL, 0.6 mmol, 1.5 equiv.). The round bottom flask was rinsed with an extra 4 mL

toluene, which was then cannulated to bring the vial solution to 0.05 M. The vial was then placed in a glass tempering beaker connected to a flow of water, the vial was subsequently submerged in water and irradiated with two EvoluChem™ 450 nm LED spotlights. After 4 h the reaction was diluted with $CHCl_3$ (8 mL), and the solvent removed under reduced pressure. Purification by flash column chromatography (*n*-hexane:EtOAc, 80:20 to 60:40), afforded the title compound (77 mg, 85%) as a beige solid. **mp** (*n*-hexane/EtOAc) 63 – 65 °C; **^{1}H NMR** (400 MHz, $CDCl_3$) δ_H 7.74 (d, *J* = 8.0 Hz, 2H, *p*-MeAr*H*), 7.33 (d, *J* = 8.0 Hz, 2H, *p*-MeAr*H*), 3.32 (t, *J* = 7.5 Hz, 2H, $SO_2CH_2CH_2$), 2.87 (t, *J* = 7.5 Hz, 2H, CH_2CH_2CO), 2.41 (s, 3H, ArCH_3), 2.13 (s, 3H COCH_3); **^{13}C NMR** (101 MHz, $CDCl_3$) δ_C 203.8, 145.0, 136.0, 130.0, 128.0, 50.6, 36.0, 29.9, 21.6; **LRMS** (ESI, *m/z*) $[M+Na]^+$ 249.0. The spectroscopic data is in agreement with literature.[31]

4. Mechanistic Experiments

4.1. UV-Vis Experiments

All spectroscopic measurements were conducted in nitrogen sparged HPLC grade toluene using quartz cuvettes (10 mm path length, Starna Scientific Ltd, UK). UV-vis absorption spectra were acquired on a Perkin Elmer Lambda 20 spectrometer at 298 K, with temperature control by a PTP-1 Peltier unit from Perkin Elmer. Fluorescence spectra were acquired at 298 K using an Edinburgh Instruments FS5 spectrofluorometer operating Fluoracle® software, and equipped with a xenon arc lamp (providing 230–1000 nm excitation range), a thermostatic sample holder (SC-20) and an R13456 PMT detector (200–950 nm spectral coverage, Hamamatsu).

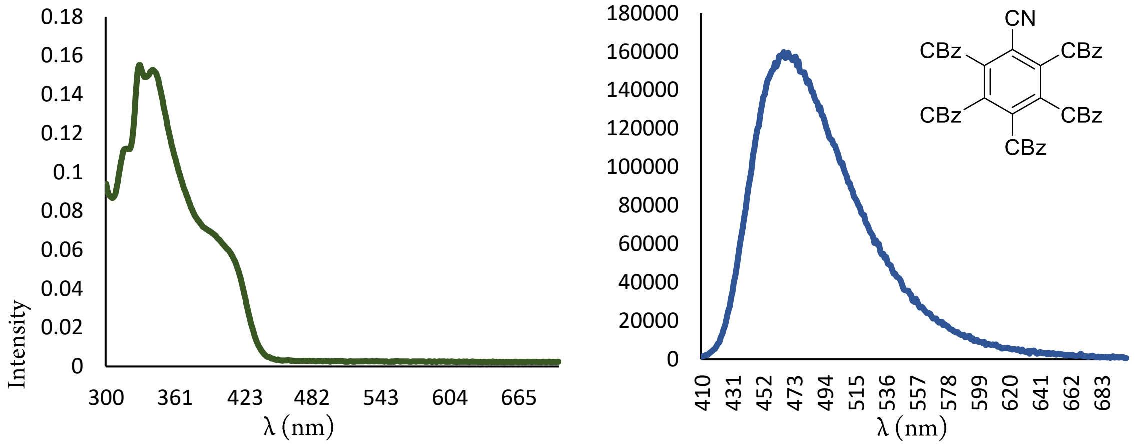

Graph S1. Absorption (left) and Emission (right) spectrum of 5CzBN in toluene, using excitation at 400 nm for the emission spectra.

The reported spectra are in agreement with previous literature reports.[37] The absorption spectrum indicates that the photocatalyst is likely excited from irradiation during the reaction.

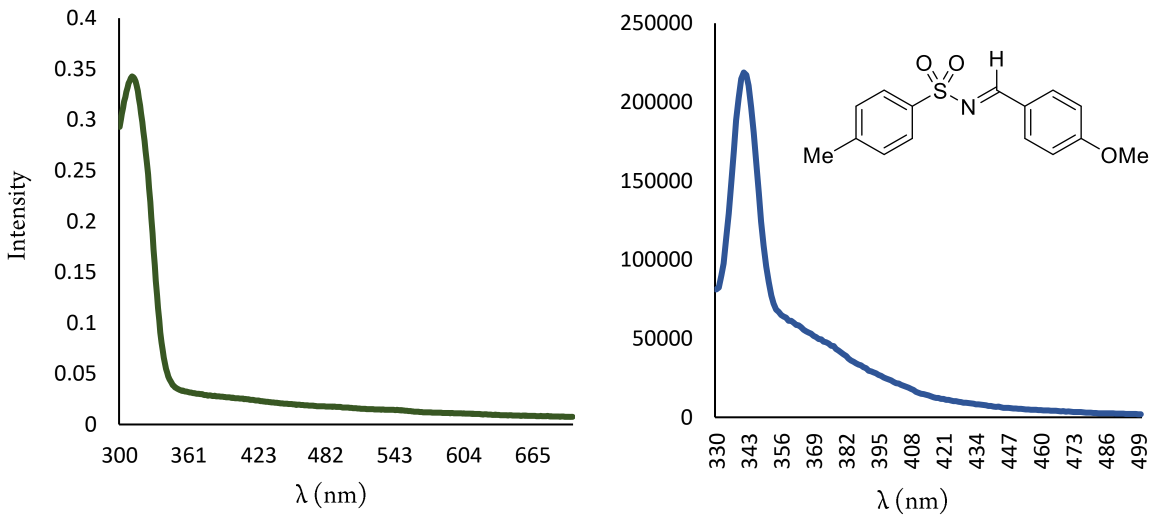

Graph S2. Absorption (left) and Emission (right) spectrum of **1a** in toluene, using excitation at 310 nm for the emission spectra.

The imine was shown to absorb at a low wavelength of *ca.* 310 nm and gave a weak fluorescence spectrum, indicating that it is unlikely that the imine directly interacts with the light. These results are similar to the previous photophysical characterisation of analogous imines.[24]

4.2. Stern-Volmer Quenching

Fluorescence quenching of 5CzBN was investigated by measuring the previous emission spectrum of 5CzBN in the presence of the reaction components at varying concentrations under an atmosphere of nitrogen. A solution of 5CzBN (0.01 mM) was treated with (0.04-0.80 mM) of either aldimine **1a**, methyl vinyl ketone or TMS_3SiH in toluene. The samples were irradiated at 400 nm and luminescence was measured over a range of 410 – 700 nm (at λ_{max} = 467 nm). I/I_0 in the range of 0.00-0.20 mM was plotted against the different concentrations of the quenchers.

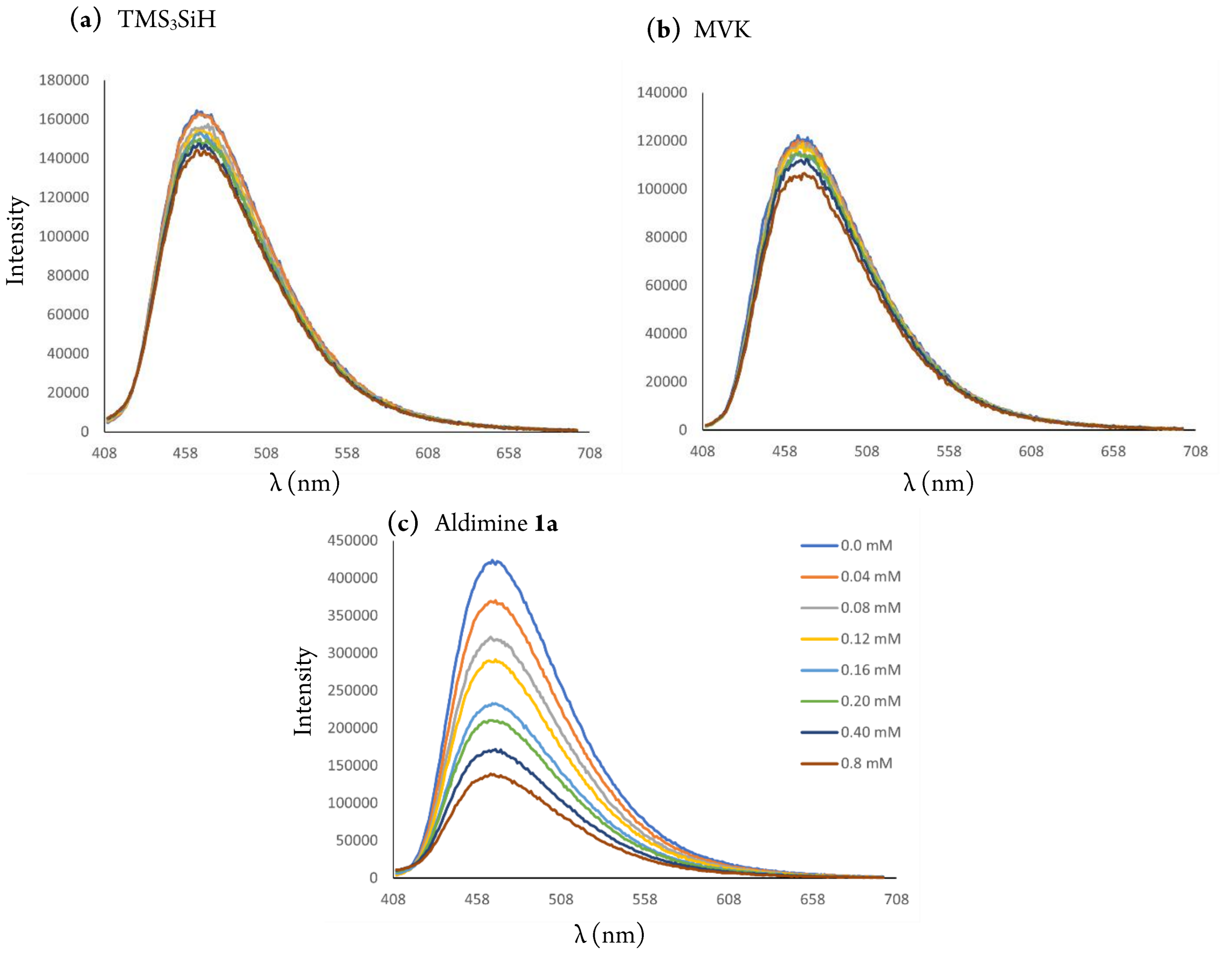

Figure S2. Fluorescence quenching of the catalyst 5CzBN in the presence of different components in the reaction at varying concentrations in toluene at room temperature. (**a**) In the presence of TMS_3SiH. (**b**) In the presence of methyl vinyl ketone. (**c**) In the presence of the model aldimine substrate.

Table S1. Fluorescence quenching of catalyst 5CzBN

c (mM)	**1a**	MVK	TMS_3SiH
0	1.00	1.00	1.00
0.04	1.15	1.03	1.01
0.08	1.33	1.02	1.06
0.12	1.47	1.03	1.06
0.16	1.82	1.05	1.07
0.2	2.02	1.05	1.09

The values for each concentration correspond to the normalised intensity (I/I_0) examined at λ_{max} = 467 nm.

The data indicates that the aldimine substrate is the most efficient quencher of the photocatalyst in the system.

4.3. Computational Experiments

All calculations were performed using the Gaussian 16 C.01 software package.[38] Geometry optimisations and frequency calculations were obtained at the (u)CAM-B3LYP/6-31G(d) level of theory, according to previous calculations.[24] When applicable implicit solvent effects were accounted for using the PCM at a standard sate. Vibrational entropies were obtained using a quasi-harmonic approximation, treating vibrational modes below 100 cm^{-1} as free rotors and as rigid rotors,[39] and implemented in Goodvibes.py.[40] Thermochemical data are reported in the standard state (298.15 K, 1 atm or 1 mol L^{-1}). Molecule visualisations were created using the CYLview software,[41] for the states of **1a** in gas phase (see below).

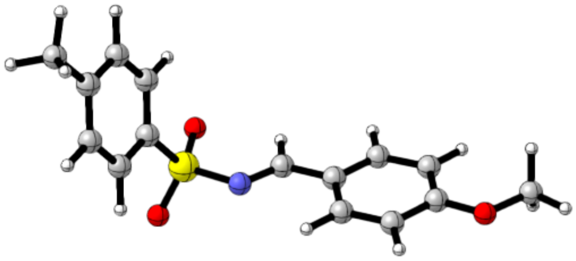

1a-S_0

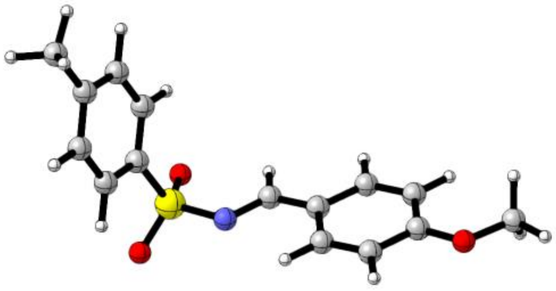

1a-T_1

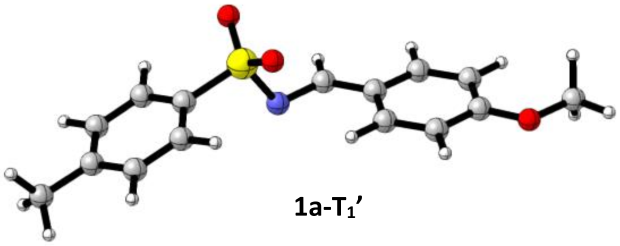

1a-T_1'

Table S2. Energies of calculated species in gas phase and various solvents.

	E	ZPE	H	T.S	T.qh-S	G(T)	qh-G(T)
**1a-S$_0$**	-1258.756030	0.277527	-1258.459008	0.069926	0.065288	-1258.528934	-1258.524297
**1a-T$_1$**	-1258.650469	0.273333	-1258.356900	0.073952	0.068331	-1258.430852	-1258.425230
**1a-T$_1$'**	-1258.658098	0.273189	-1258.364797	0.074192	0.068223	-1258.438989	-1258.433020
**1a-S$_0$**(PhMe)	-1258.764528	0.277324	-1258.467700	0.066609	0.062152	-1258.534309	-1258.529852
**1a-T$_1$**(PhMe)	-1258.660169	0.273462	-1258.366506	0.070355	0.065051	-1258.436861	-1258.431558
**1a-T$_1$'**(PhMe)	-1258.665091	0.273040	-1258.371980	0.069283	0.064306	-1258.441263	-1258.436286
**1a-S$_0$**(MeCN)	-1258.774251	0.277063	-1258.477648	0.066931	0.062330	-1258.544578	-1258.539978
**1a-T$_1$**(MeCN)	-1258.672019	0.273522	-1258.378347	0.069419	0.064587	-1258.447766	-1258.442935
**1a-T$_1$'**(MeCN)	-1258.673340	0.272844	-1258.380405	0.072386	0.067398	-1258.452790	-1258.447803

Calculations obtained with (u)CAM-B3LYP/6-31G(d), with energies reported in Hartree. E – electronic energy, ZPE – vibrational zero-point energy, H – enthalpy, S – Entropy, qh-S – quasi-harmonic corrected entropy, G – gibbs free energy, qh-G – quasi-harmonic corrected gibbs free energy. T is temperature at 298.15 K. The calculations are calculated in the gas phase unless solvent is specified in parenthesise.

1a-S$_0$ refers to the ground singlet state. Two triplet states were located during this study named **T$_1$** and **T$_1$'**, where **1a-T$_1$** contains a planar geometry around the C–N whilst **1a-T$_1$'** is twisted. The twisted triplet state was consistently lower in energy, in agreement with previous reports.[24] However when considering the feasibility of the triplet-triplet energy transfer process the estimation of E_T uses the higher energy triplet state, in order to minimise structural reorganisation during the process.

$$\mathbf{E_T \approx G(T_1) - G(S_0)}$$

Calc.	E_T (eV)
**1a**	2.70
**1a**(PhMe)	2.67
**1a**(MeCN)	2.64

Where the gibbs free energy uses the previous mentioned quasi-harmonic correction to the entropy term. The previous zero-zero vibrational state excitation energy ($E_{0,0}$) of 5CzBN was estimated to be 2.83 eV from the overlap of emission and absorption wavelengths in acetonitrile.[1] Alternatively triplet energies (E_T) from phosphorescence spectra of 5CzBN in toluene or acetonitrile at 77 K indicate an energy range of 2.72-2.78 eV, with charge transfer character.[42] Regardless, the catalyst appears to have at least an equal to or higher triplet energy than the substrate ($\Delta E_T \geq 0$, exergonic) in gas phase and solvent, predicting a favourable process.

4.4. Cyclic Voltammetry

Cyclic voltammetry measurements were made using an IKA ElectroSyn setup. The voltammetric cell consisted of a glassy carbon electrode, a Pt wire counter electrode, and Ag/$AgNO_3$ (0.01 M in acetonitrile) reference electrode. The measurements were carried out under a nitrogen atmosphere using a solution of sample with a

concentration of 1.0 mM in acetonitrile containing tetrabutylammonium perchlorate (*n*-$Bu_4N \cdot ClO_4$) as a supporting electrolyte (0.10 M). The scan rate was 100 mV s^{-1}. Voltammograms were calibrated with ferrocene (0.40 V vs SCE) as an internal reference. The reported potentials were taken at the half-height of the cathodic peaks ($E_{p/2}$) of the compounds since all redox processes were irreversible.

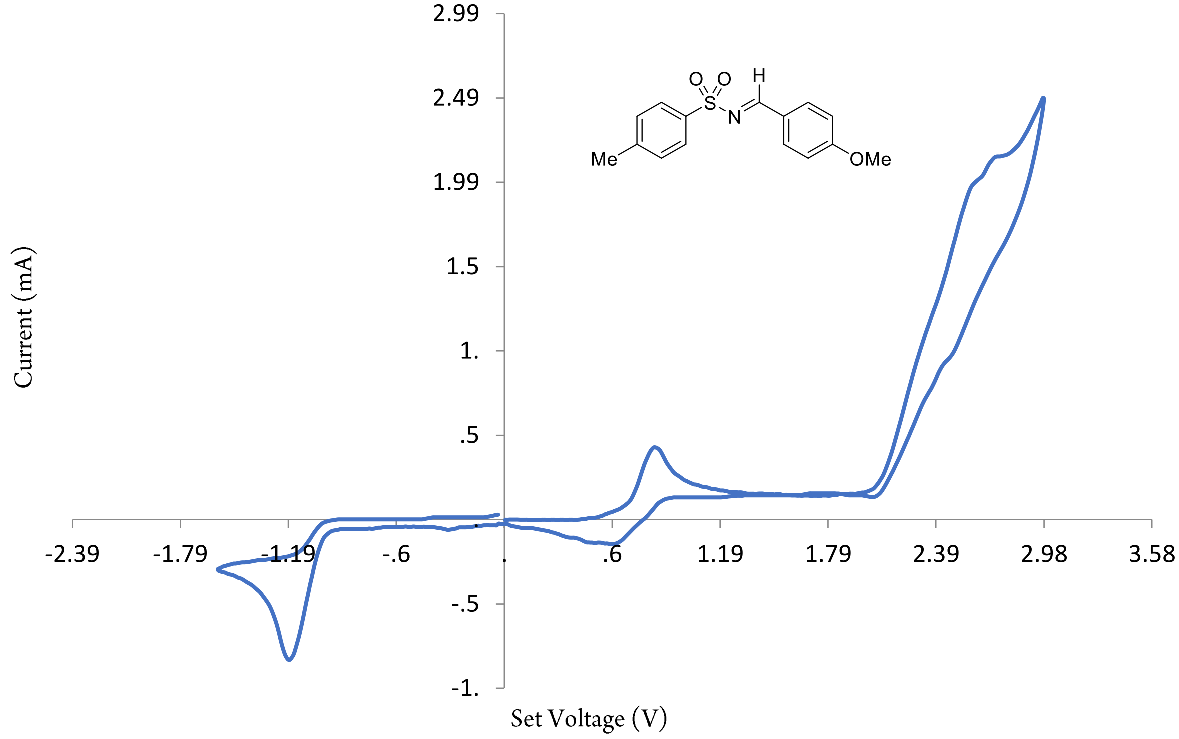

Graph S3. Cyclic voltammetry of **1a** scanning for both oxidation and reduction in the presence of ferrocene.

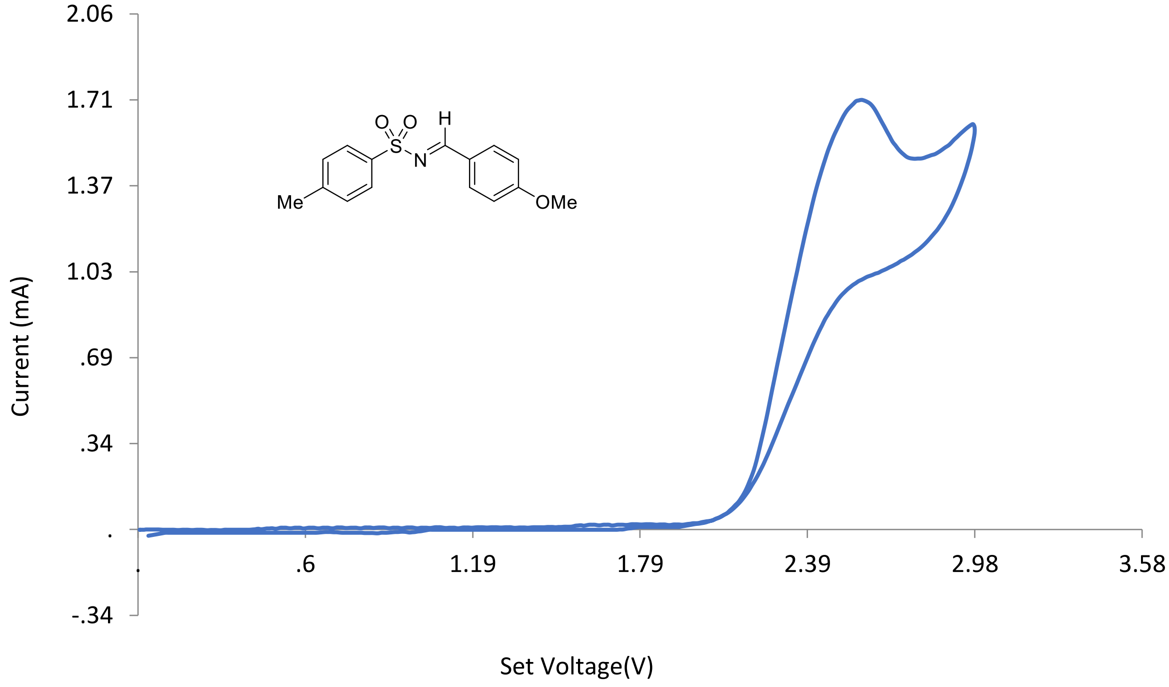

Graph S4. Cyclic voltammetry of **1a** scanning to obtain the oxidation potential.

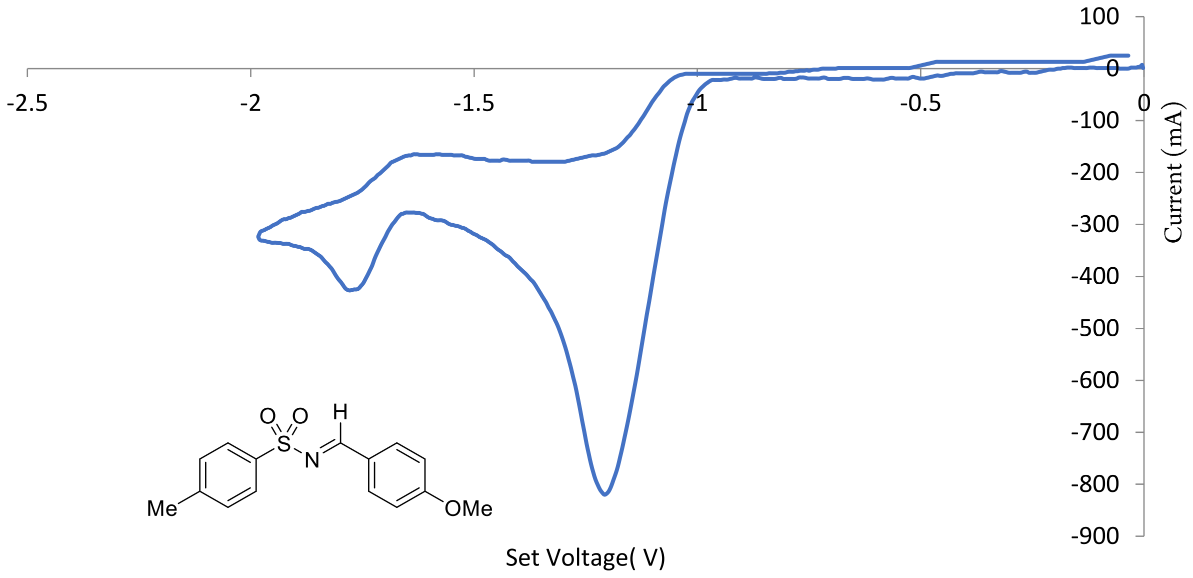

Graph S5. Cyclic voltammetry of **1a** scanning to obtain the reduction potential.

The redox potentials E(**1a**/**1a**$^{\bullet-}$) = −1.44 V and E(**1a**$^{\bullet+}$/**1a**) = +1.98 V (in MeCN vs. SCE) were obtained. The oxidation potential of the substrate is out of the range of the corresponding excited states of the catalyst. Therefore, oxidation of the substrate is an unlikely mechanism.

4.5. Control Reactions

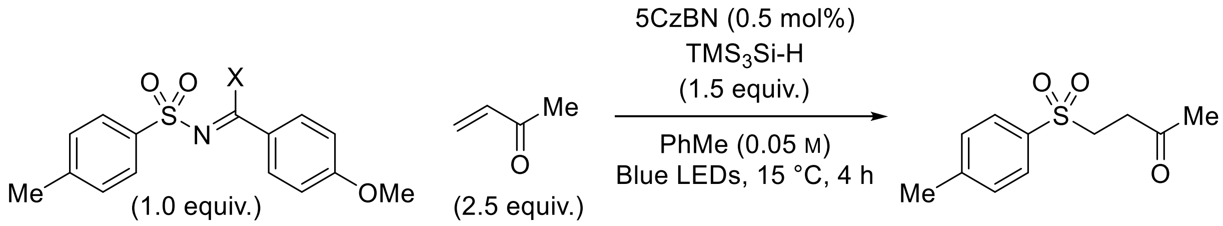

Entry	Modification to the Above Conditions	X	^{1}H NMR Yield
1	No photocatalyst	H	0%
2	No light	H	< 5%
3	No light, 80 °C	H	< 5%
4	No TMS_3Si-H	H	15%
5	No TMS_3Si-H, PhMe-d_8 instead of PhMe	H	22%[a]
6	No TMS_3Si-H	D	20%[a]
7	*p*-TolSO_2Na instead of sulfonyl imine	H	<3%
8	+TEMPO (2.5 equiv.)	H	0%

Yields determined by analysis of crude ^{1}H NMR spectrum using 1.0 equiv. of 1,3,5-trimethoxybenzene as internal standard. [a] No observed Deuterium incorporation.

Control reactions were performed on the optimized system for activated alkenes (see section 2.3 for the control reactions on the neutral alkenes system), and removing the photocatalyst (entry 1) completely shut down the reaction. Reactions in the dark, either at 15 (entry 2) or 80 °C (entry 3), also appeared inefficient, with observed yields being lower than 5%. Control reaction without HAD gave a significant drop in yield with only 15% of desired sulfone being observed (entry 4). Deuterium experiments were performed to rationalize this result (entries 5-6), but no Deuterium incorporation was observed.

5. References

(1) Speckmeier, E.; Fischer, T. G.; Zeitler, K., A Toolbox Approach To Construct Broadly Applicable Metal-Free Catalysts for Photoredox Chemistry: Deliberate Tuning of Redox Potentials and Importance of Halogens in Donor–Acceptor Cyanoarenes. *J. Am. Chem. Soc.* **2018,** *140*, 15353-15365.

(2) Qi, X.; Yu, F.; Chen, P.; Liu, G., Intermolecular Palladium-Catalyzed Oxidative Fluorocarbonylation of Unactivated Alkenes: Efficient Access to β-Fluorocarboxylic Esters. *Angew. Chem. Int. Ed.* **2017,** *56*, 12692-12696.

(3) Lippincott, D. J.; Linstadt, R. T. H.; Maser, M. R.; Gallou, F.; Lipshutz, B. H., Synthesis of Functionalized 1,3-Butadienes via Pd-Catalyzed Cross-Couplings of Substituted Allenic Esters in Water at Room Temperature. *Org. Lett.* **2018,** *20*, 4719-4722.

(4) M. T. Ferreira, P.; L. S. Maia, H.; S. Monteiro, L.; Sacramento, J., High yielding synthesis of dehydroamino acid and dehydropeptide derivatives. *J. Chem. Soc., Perkin Trans. 1* **1999**, 3697-3703.

(5) Hell, S. M.; Meyer, C. F.; Laudadio, G.; Misale, A.; Willis, M. C.; Noël, T.; Trabanco, A. A.; Gouverneur, V., Silyl Radical-Mediated Activation of Sulfamoyl Chlorides Enables Direct Access to Aliphatic Sulfonamides from Alkenes. *J. Am. Chem. Soc.* **2020,** *142*, 720-725.

(6) Gratais, A.; Pannecoucke, X.; Bouzbouz, S., Studies of the New Reactivity of Chiral Acrylamides and Unprotected Pyrroles: Diastereoselective and Carbonyl Compatible 1,4-Addition. *Synlett* **2014,** *25*, 1555-1560.

(7) Zhang, Y.; Lee, J. H.; Danishefsky, S. J., Antarafacial Mediation of Oxygen Delivery by a Phenylsulfinyl Group in the Epoxidation of Proximal Double Bonds: Intramolecular Trapping of an Early Pummerer Intermediate with Stereoelectronic Control. *J. Am. Chem. Soc.* **2011,** *133*, 752-755.

(8) Cleary, P. A.; Woerpel, K. A., Metal-Catalyzed Rearrangement of Homoallylic Ethers to Silylmethyl Allylic Silanes in the Presence of a Di-tert-butylsilylene Source. *Org. Lett.* **2005,** *7*, 5531-5533.

(9) Mallinger, A.; Schiemann, K.; Rink, C.; Stieber, F.; Calderini, M.; Crumpler, S.; Stubbs, M.; Adeniji-Popoola, O.; Poeschke, O.; Busch, M.; Czodrowski, P.; Musil, D.; Schwarz, D.; Ortiz-Ruiz, M.-J.; Schneider, R.; Thai, C.; Valenti, M.; de Haven Brandon, A.; Burke, R.; Workman, P.; Dale, T.; Wienke, D.; Clarke, P. A.; Esdar, C.; Raynaud, F. I.; Eccles, S. A.; Rohdich, F.; Blagg, J., Discovery of Potent, Selective, and Orally Bioavailable Small-Molecule Modulators of the Mediator Complex-Associated Kinases CDK8 and CDK19. *J. Med. Chem.* **2016,** *59*, 1078-1101.

(10) Tota, A.; St John-Campbell, S.; Briggs, E. L.; Estévez, G. O.; Afonso, M.; Degennaro, L.; Luisi, R.; Bull, J. A., Highly Chemoselective NH- and O-Transfer to Thiols Using Hypervalent Iodine Reagents: Synthesis of Sulfonimidates and Sulfonamides. *Org. Lett.* **2018,** *20*, 2599-2602.

(11) Hayashi, E.; Yamaguchi, Y.; Kita, Y.; Kamata, K.; Hara, M., One-pot aerobic oxidative sulfonamidation of aromatic thiols with ammonia by a dual-functional β-MnO2 nanocatalyst. *Chem. Commun.* **2020,** *56*, 2095-2098.

(12) Wang, M.; Fan, Q.; Jiang, X., Metal-free construction of primary sulfonamides through three diverse salts. *Green Chemistry* **2018,** *20*, 5469-5473.

(13) Davies, T. Q.; Tilby, M. J.; Skolc, D.; Hall, A.; Willis, M. C., Primary Sulfonamide Synthesis Using the Sulfinylamine Reagent N-Sulfinyl-O-(tert-butyl)hydroxylamine, t-BuONSO. *Org. Lett.* **2020,** *22*, 9495-9499.

(14) Galli, U.; Mesenzani, O.; Coppo, C.; Sorba, G.; Canonico, P. L.; Tron, G. C.; Genazzani, A. A., Identification of a sirtuin 3 inhibitor that displays selectivity over sirtuin 1 and 2. *Eur. J. Med. Chem.* **2012,** *55*, 58-66.

(15) McComsey, D. F.; Smith-Swintosky, V. L.; Parker, M. H.; Brenneman, D. E.; Malatynska, E.; White, H. S.; Klein, B. D.; Wilcox, K. S.; Milewski, M. E.; Herb, M.; Finley, M. F. A.; Liu, Y.; Lubin, M. L.; Qin, N.; Reitz, A. B.; Maryanoff, B. E., Novel, Broad-Spectrum Anticonvulsants Containing a Sulfamide Group: Pharmacological Properties of (S)-N-[(6-Chloro-2,3-dihydrobenzo[1,4]dioxin-2-yl)methyl]sulfamide (JNJ-26489112). *J. Med. Chem.* **2013,** *56*, 9019-9030.

(16) Matulis, D.; Čapkauskaite, E.; Zaksauskas, A.; Morkunaite, V. SELECTIVE INHIBITORS OF CARBONIC ANHYDRASE 2017.

(17) Wang, F.; Liu, H.; Fu, H.; Jiang, Y.; Zhao, Y., Highly Efficient Iron(II) Chloride/N-Bromosuccinimide-Mediated Synthesis of Imides and Acylsulfonamides. *Adv. Synth. Catal.* **2009,** *351*, 246-252.

(18) Pérez-Palau, M.; Cornella, J., Synthesis of Sulfonyl Fluorides from Sulfonamides. *Eur. J. Org. Chem.* **2020,** *2020*, 2497-2500.

(19) Morales, S.; Guijarro, F. G.; García Ruano, J. L.; Cid, M. B., A General Aminocatalytic Method for the Synthesis of Aldimines. *J. Am. Chem. Soc.* **2014,** *136*, 1082-1089.

(20) Stead, D., The diastereoselective synthesis of 2,3-diaryl-3-cyano-substituted pyrrolidines via the MgI2 mediated ring expansion of aryl cyclopropyl nitriles. *Tetrahedron Lett.* **2020,** *61*, 152325.

(21) Kolb, S.; Petzold, M.; Brandt, F.; Jones, P. G.; Jacob, C. R.; Werz, D. B., Electrocatalytic Activation of Donor–Acceptor Cyclopropanes and Cyclobutanes: An Alternative C(sp^3)–C(sp^3) Cleavage Mode. *Angew. Chem. Int. Ed.* **2021,** *60*, 15928-15934.

(22) Liu, H.; Zhang, Q.; Wang, L.; Tong, X., PPh_3-catalyzed [2 + 2 + 2] and [4 + 2] annulations: synthesis of highly substituted 1,2-dihydropyridines (DHPs). *Chem. Commun.* **2010,** *46*, 312-314.

(23) Reeves, J. T.; Visco, M. D.; Marsini, M. A.; Grinberg, N.; Busacca, C. A.; Mattson, A. E.; Senanayake, C. H., A General Method for Imine Formation Using $B(OCH_2CF_3)_3$. *Org. Lett.* **2015,** *17*, 2442-2445.

(24) Uraguchi, D.; Tsuchiya, Y.; Ohtani, T.; Enomoto, T.; Masaoka, S.; Yokogawa, D.; Ooi, T., Unveiling Latent Photoreactivity of Imines. *Angew. Chem. Int. Ed.* **2020,** *59*, 3665-3670.
(25) Ram, R. N.; Khan, A. A., A Simple Method for the Preparation of *N*-Sulfonylimines by Direct Condensation of *p*-Toluenesulfonamide with Diaryl Ketones. *Synth. Commun.* **2001,** *31*, 841-846.
(26) Kohler, M. C.; Yost, J. M.; Garnsey, M. R.; Coltart, D. M., Direct Carbon−Carbon Bond Formation via Soft Enolization: A Biomimetic Asymmetric Mannich Reaction of Phenylacetate Thioesters. *Org. Lett.* **2010,** *12*, 3376-3379.
(27) Sakamoto, R.; Inada, T.; Sakurai, S.; Maruoka, K., [2 + 2] Photocycloadditions between the Carbon−Nitrogen Double Bonds of Imines and Carbon−Carbon Double Bonds. *Org. Lett.* **2016,** *18*, 6252-6255.
(28) Rueping, M.; Tolstoluzhsky, N., Copper Catalyzed C−H Functionalization for Direct Mannich Reactions. *Org. Lett.* **2011,** *13*, 1095-1097.
(29) Lanter, J. C.; Chen, H.; Zhang, X.; Sui, Z., Asymmetric Aza-Mannich Reactions of Sulfinimines: Scope and Application to the Total Synthesis of a Bromopyrrole Alkaloid. *Org. Lett.* **2005,** *7*, 5905-5907.
(30) González, A. S.; Arrayás, R. G.; Carretero, J. C., Copper(I)-Fesulphos Lewis Acid Catalysts for Enantioselective Mannich-Type Reaction of N-Sulfonyl Imines. *Org. Lett.* **2006,** *8*, 2977-2980.
(31) Vellakkaran, M.; Andappan, M. M. S.; Nagaiah, K.; Nanubolu, J. B., Direct Synthesis of γ-Keto Sulfones from Allylic Alcohols: One-Pot Palladium(II)-Catalyzed Generation of Enones Followed by Water-Mediated 1,4-Addition of Organosulfinates. *Eur. J. Org. Chem.* **2016,** *2016*, 3575-3583.
(32) Marset, X.; Guillena, G.; Ramón, D. J., Deep Eutectic Solvents as Reaction Media for the Palladium-Catalysed C−S Bond Formation: Scope and Mechanistic Studies. *Chem. Eur. J.* **2017,** *23*, 10522-10526.
(33) Keim, W.; Herwig, J.; Pelzer, G., Synthesis of γ-Oxo Sulfones via Palladium- and Platinum-Catalyzed Hydrosulfination. *J. Org. Chem.* **1997,** *62*, 422-424.
(34) Zhu, H.; Zhang, Y.; Liu, Y.; Yang, L.; Xie, Z.; Jiang, G.; Le, Z.-G., A general and practical sulfonylation of benzylic ammonium salts with sulfonyl hydrazides for the synthesis of sulfones. *Tetrahedron Lett.* **2020,** *61*, 151975.
(35) Zhang, H.; Zhou, Y.; Tian, P.; Jiang, C., Copper-Catalyzed Amide Radical-Directed Cyanation of Unactivated Csp3−H Bonds. *Org. Lett.* **2019,** *21*, 1921-1925.
(36) Wang, J.-J.; Yu, W., Hydrosulfonylation of Unactivated Alkenes by Visible Light Photoredox Catalysis. *Org. Lett.* **2019,** *21*, 9236-9240.
(37) Hosokai, T.; Matsuzaki, H.; Nakanotani, H.; Tokumaru, K.; Tsutsui, T.; Furube, A.; Nasu, K.; Nomura, H.; Yahiro, M.; Adachi, C., Evidence and mechanism of efficient thermally activated delayed fluorescence promoted by delocalized excited states. *Sci. Adv.* **2017,** *3*, e1603282.

(38) Frisch, M. J.; Trucks, G. W.; Schlegel, H. B.; Scuseria, G. E.; Robb, M. A.; Cheeseman, J. R.; Scalmani, G.; Barone, V.; Petersson, G. A.; Nakatsuji, H.; Li, X.; Caricato, M.; Marenich, A. V.; Bloino, J.; Janesko, B. G.; Gomperts, R.; Mennucci, B.; Hratchian, H. P.; Ortiz, J. V.; Izmaylov, A. F.; Sonnenberg, J. L.; Williams; Ding, F.; Lipparini, F.; Egidi, F.; Goings, J.; Peng, B.; Petrone, A.; Henderson, T.; Ranasinghe, D.; Zakrzewski, V. G.; Gao, J.; Rega, N.; Zheng, G.; Liang, W.; Hada, M.; Ehara, M.; Toyota, K.; Fukuda, R.; Hasegawa, J.; Ishida, M.; Nakajima, T.; Honda, Y.; Kitao, O.; Nakai, H.; Vreven, T.; Throssell, K.; Montgomery Jr., J. A.; Peralta, J. E.; Ogliaro, F.; Bearpark, M. J.; Heyd, J. J.; Brothers, E. N.; Kudin, K. N.; Staroverov, V. N.; Keith, T. A.; Kobayashi, R.; Normand, J.; Raghavachari, K.; Rendell, A. P.; Burant, J. C.; Iyengar, S. S.; Tomasi, J.; Cossi, M.; Millam, J. M.; Klene, M.; Adamo, C.; Cammi, R.; Ochterski, J. W.; Martin, R. L.; Morokuma, K.; Farkas, O.; Foresman, J. B.; Fox, D. J. *Gaussian 16 Rev. C.01*, Wallingford, CT, 2016.

(39) Grimme, S., Supramolecular Binding Thermodynamics by Dispersion-Corrected Density Functional Theory. *Chem. Eur. J.* **2012,** *18*, 9955-9964.

(40) Luchini, G.; Alegre-Requena, J.; Funes-Ardoiz, I.; Paton, R., GoodVibes: automated thermochemistry for heterogeneous computational chemistry data *F1000Research* **2020,** *9*, 291.

(41) Y., L. C. *CYLview*, http://www.cylview.org., 2009.

(42) (a) Noda, H.; Nakanotani, H.; Adachi, C., Excited state engineering for efficient reverse intersystem crossing. *Sci. Adv.* **2018,** *4*, eaao6910; (b) Hosokai, T.; Noda, H.; Nakanotani, H.; Nawata, T.; Nakayama, Y.; Matsuzaki, H.; Adachi, C., Solvent-dependent investigation of carbazole benzonitrile derivatives: does the ^{3}LE−^{1}CT energy gap facilitate thermally activated delayed fluorescence? *J. Photon. Energy* **2018,** *8*, 32102.

6. HPLC Data

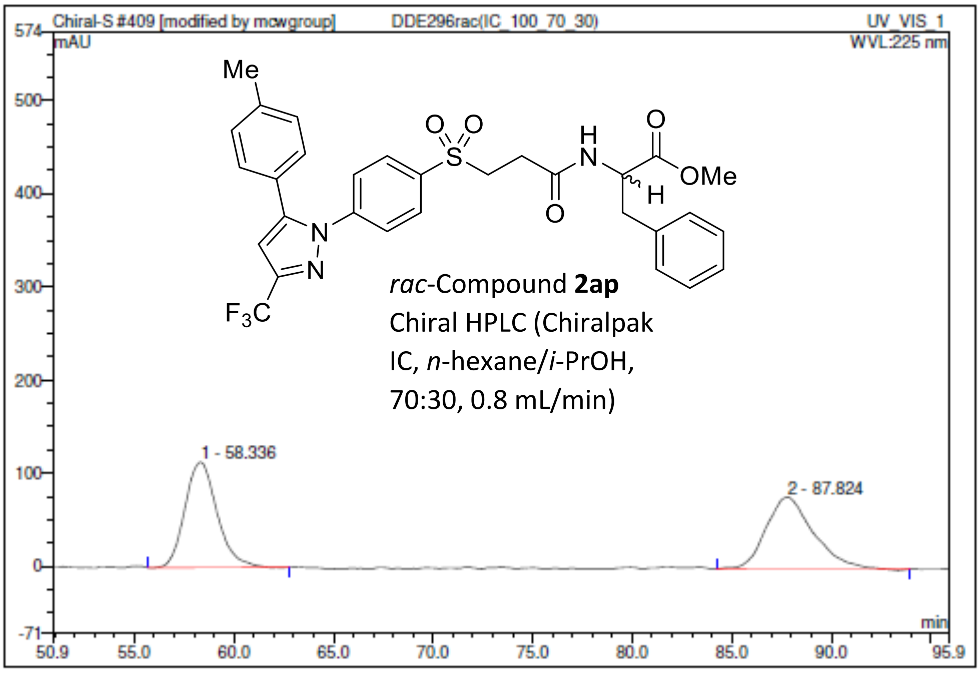

No.	Ret.Time min		Peak Name	Height mAU	Area mAU*min	Rel.Area %	Amount	Type
1	58.34	n.a.		113.072	211.301	50.00	n.a.	BMB*
2	87.82	n.a.		76.936	211.292	50.00	n.a.	BMB*
Total:				190.009	422.593	100.00	0.000	

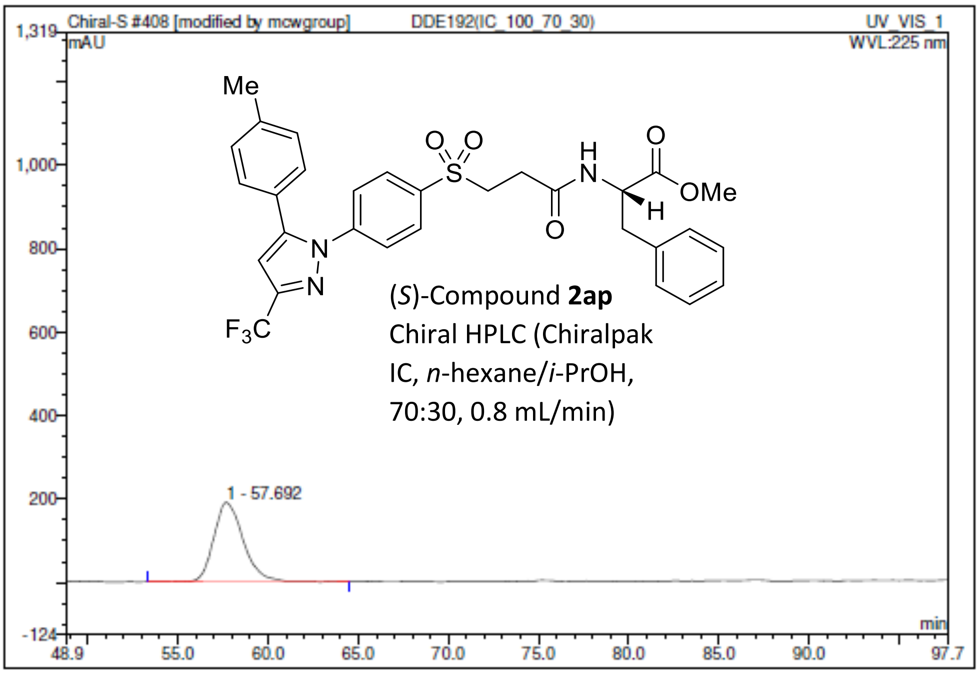

No.	Ret.Time min		Peak Name	Height mAU	Area mAU*min	Rel.Area %	Amount	Type
1	57.69	n.a.		189.286	354.801	100.00	n.a.	BMB*
Total:				189.286	354.801	100.00	0.000	

7. NMR Spectra

Compound **9c**
^{1}H NMR
(400 MHz, acetone-d_6)

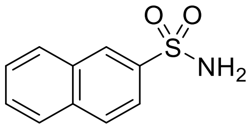

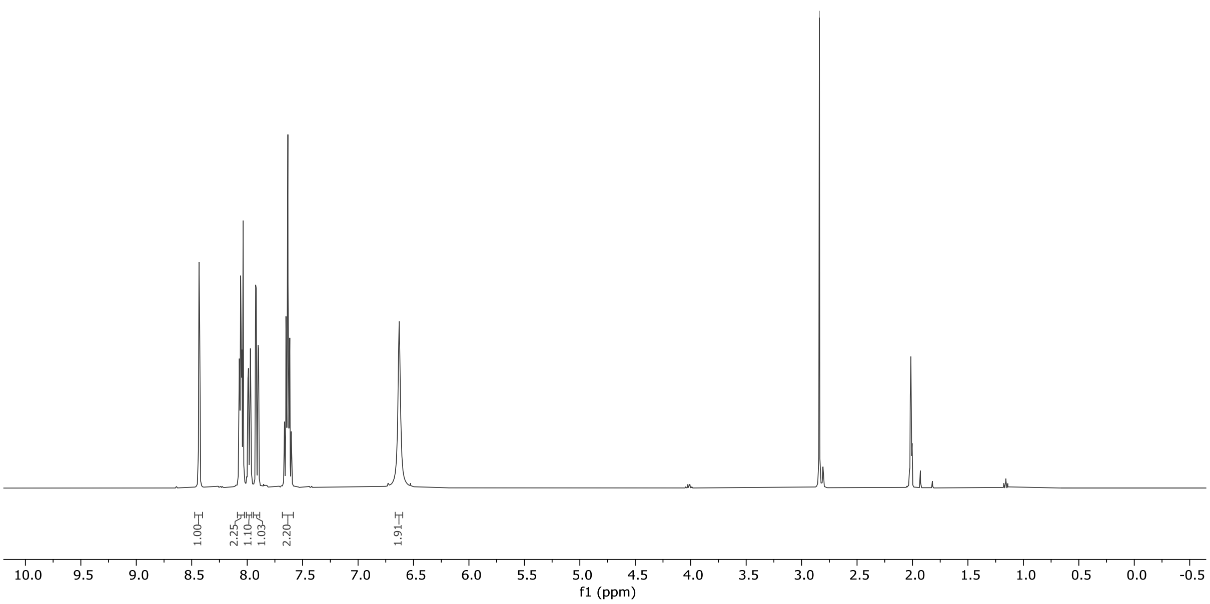

Compound **9c**
^{13}C NMR
(101 MHz, acetone-d_6)

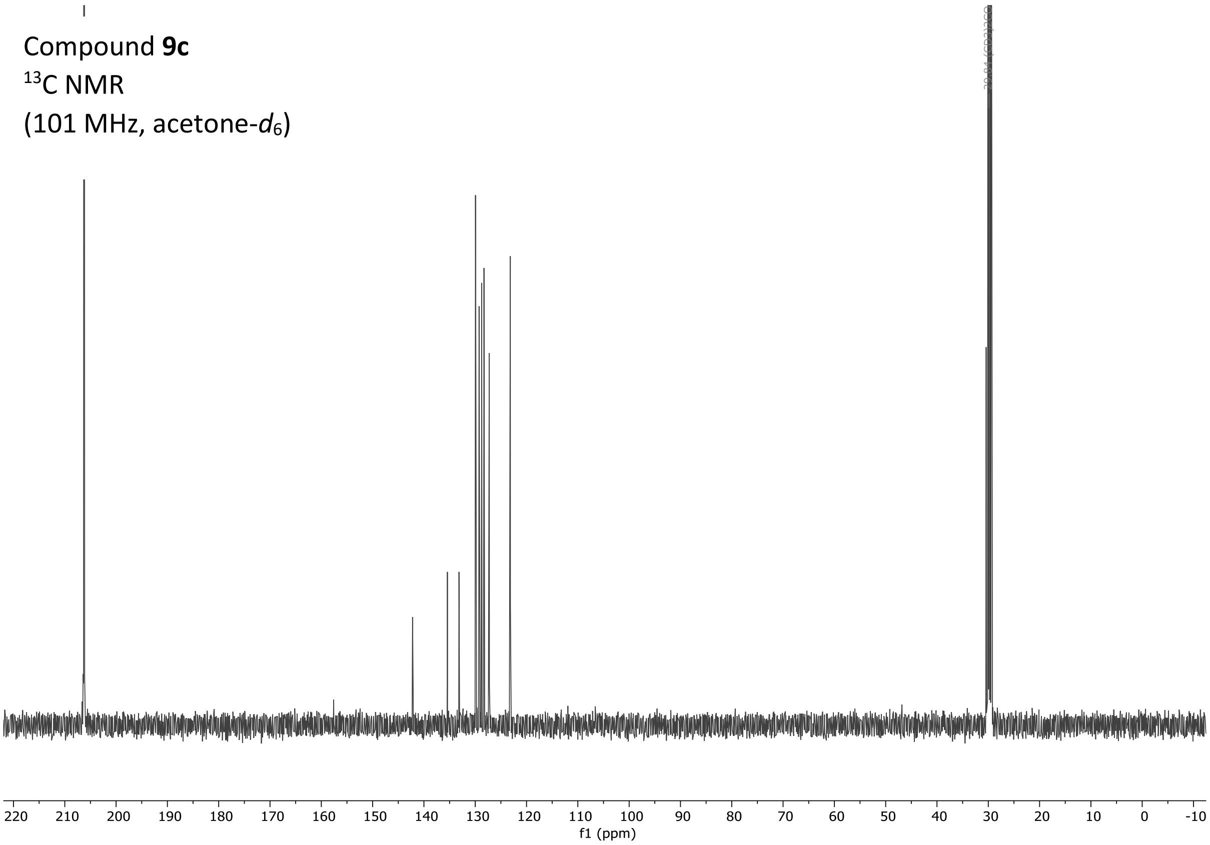

Compound **9d**
^{1}H NMR
(400 MHz, acetone-d_6)

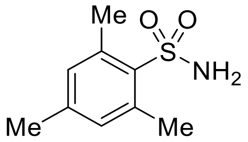

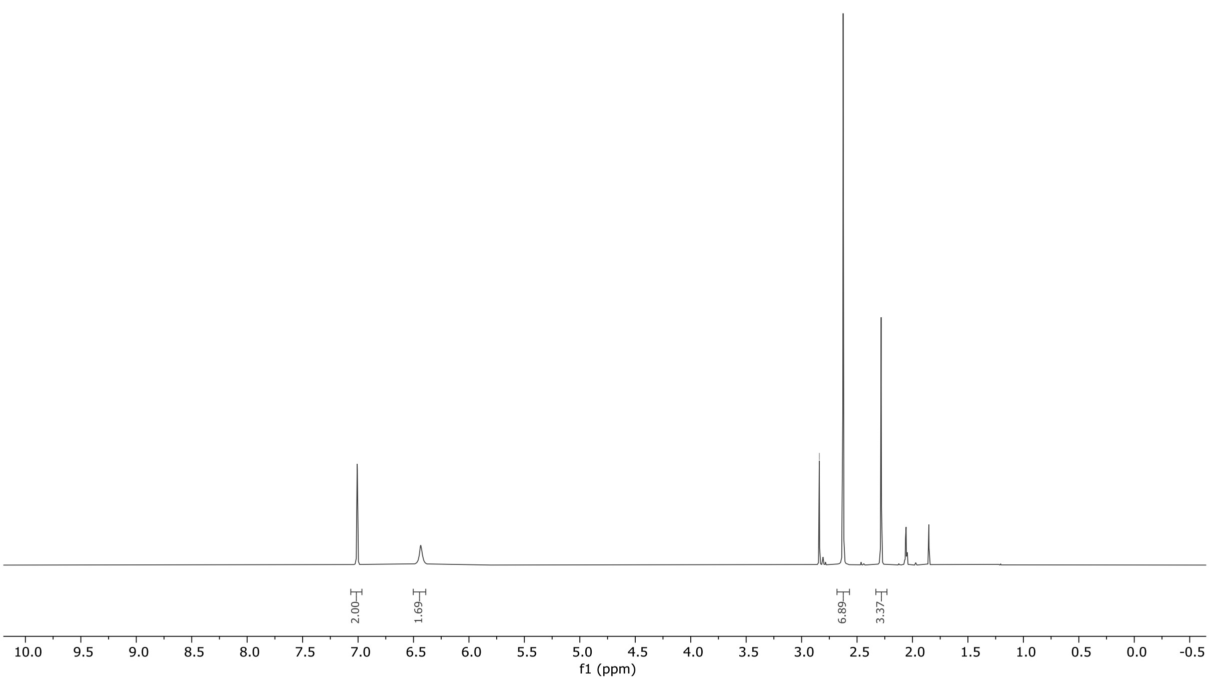

Compound **9d**
^{13}C NMR
(101 MHz, acetone-d_6)

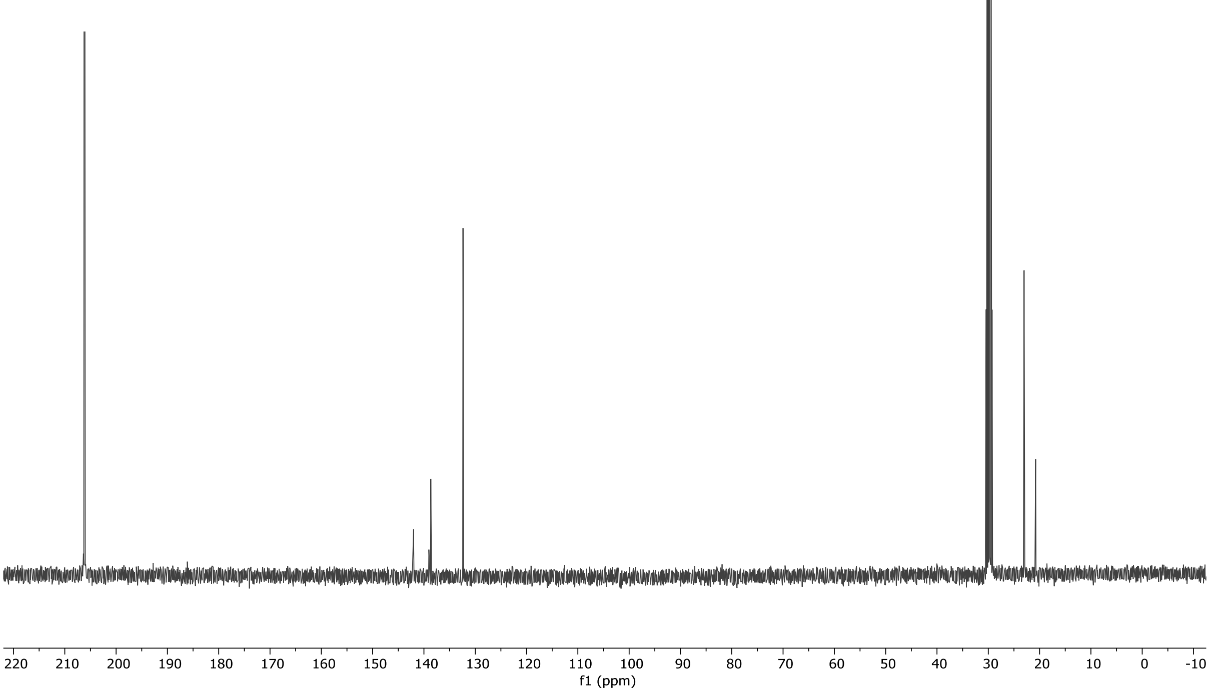

Compound **9f**
^{1}H NMR
(400 MHz, acetone-d_6)

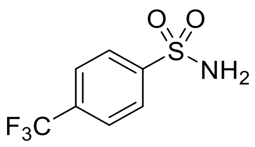

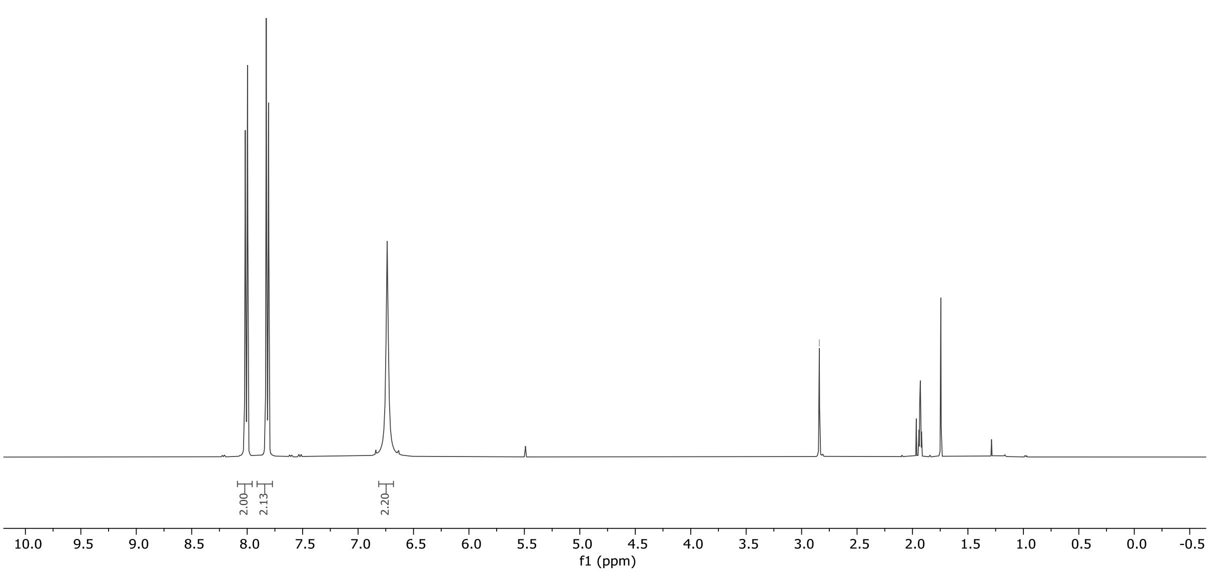

Compound **9f**
^{13}C NMR
(101 MHz, acetone-d_6)

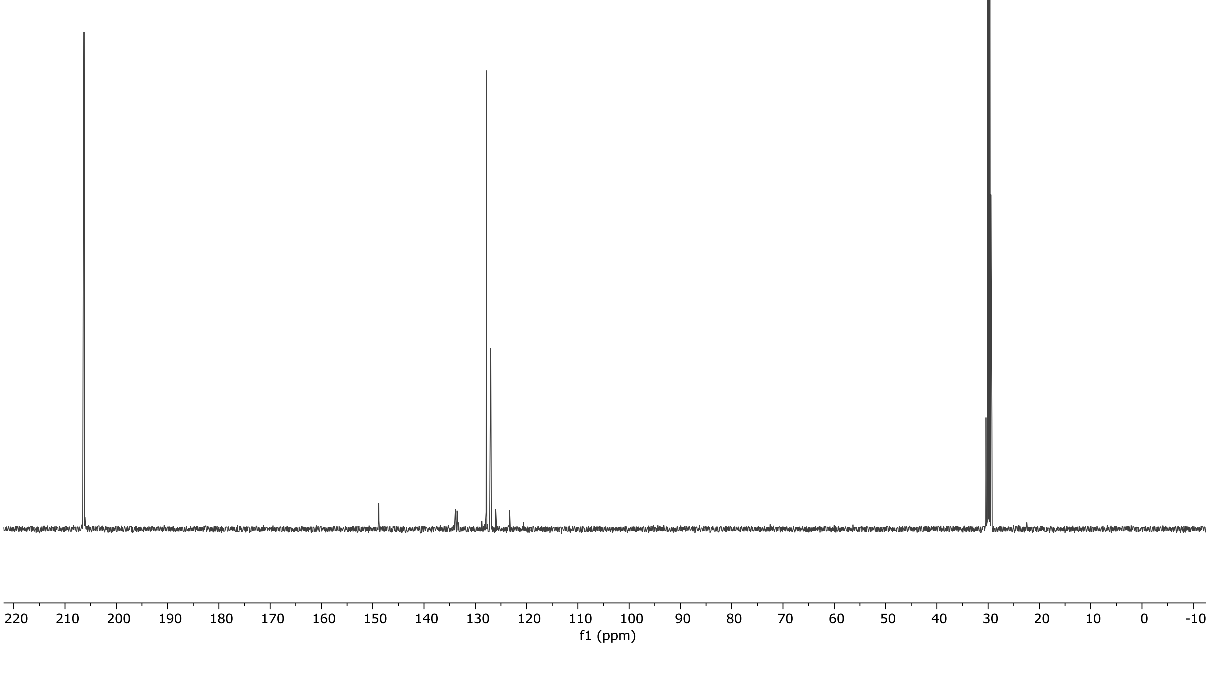

Compound **9f**
^{19}F NMR
(377 MHz, acetone-d_6)

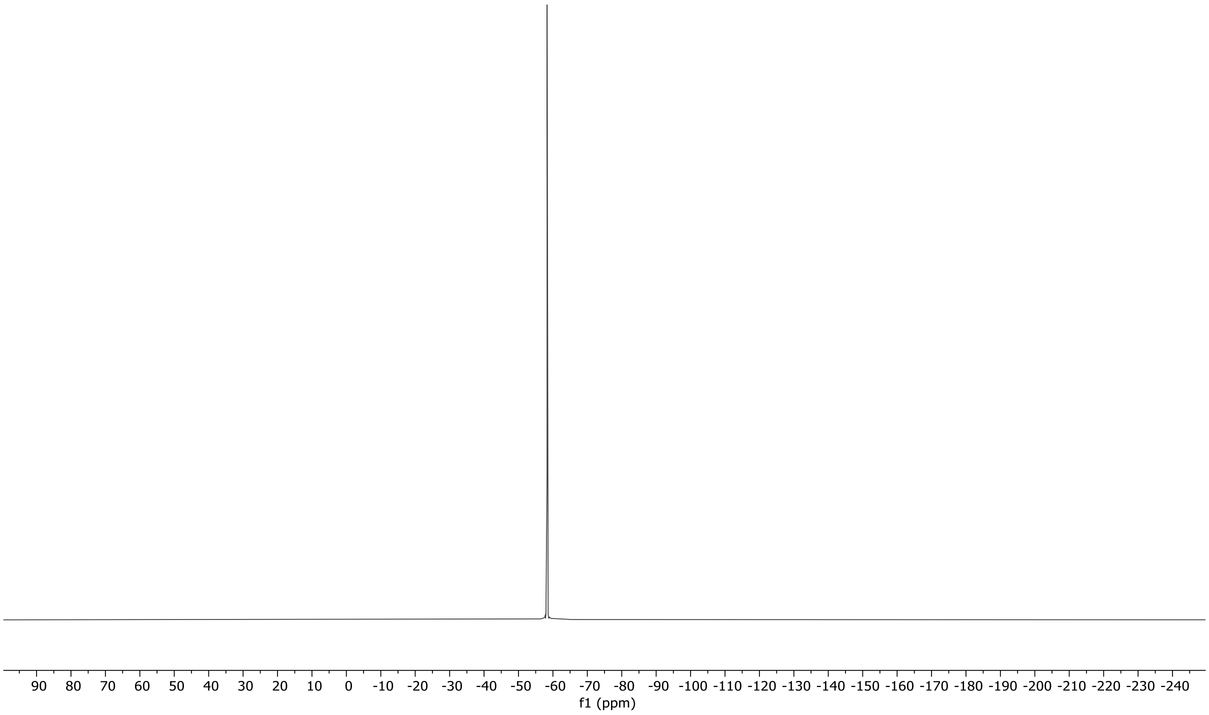

Compound **9h**
^{1}H NMR
(400 MHz, acetone-d_6)

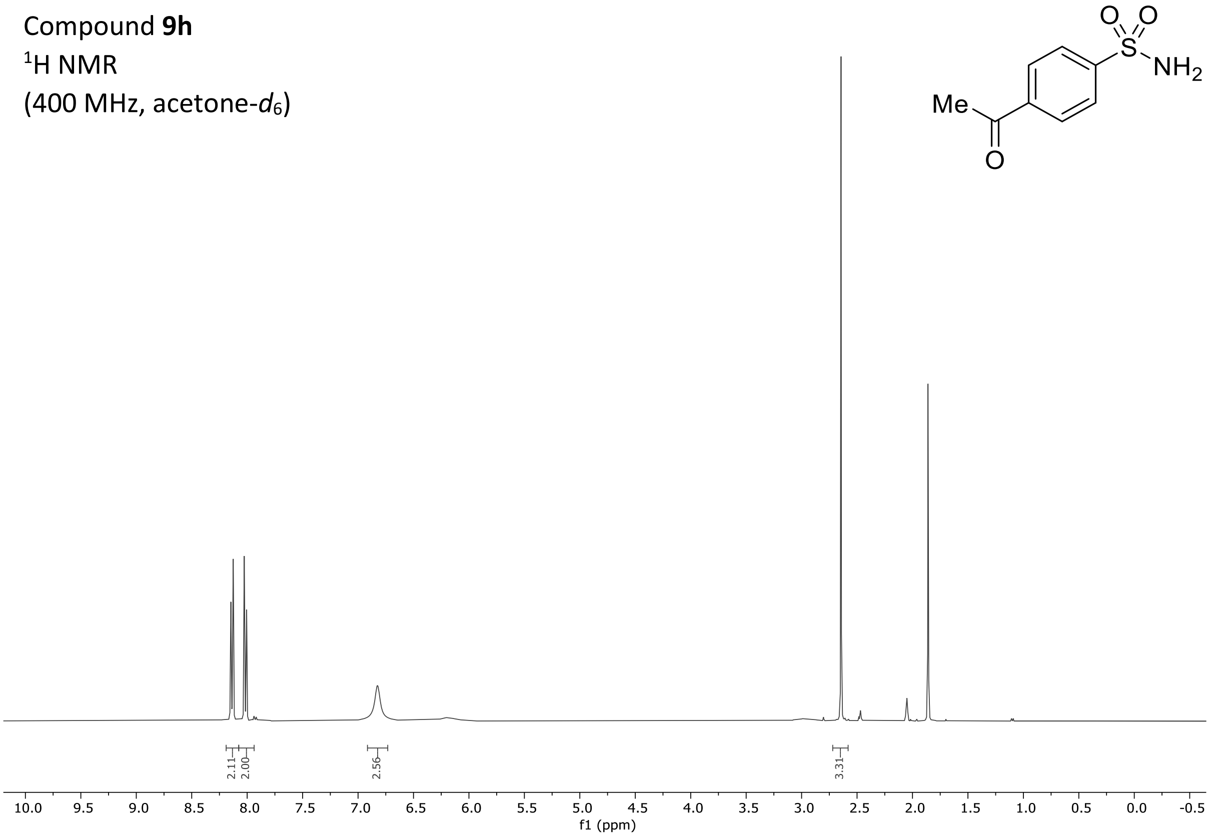

Compound **9h**
^{13}C NMR
(101 MHz, acetone-d_6)

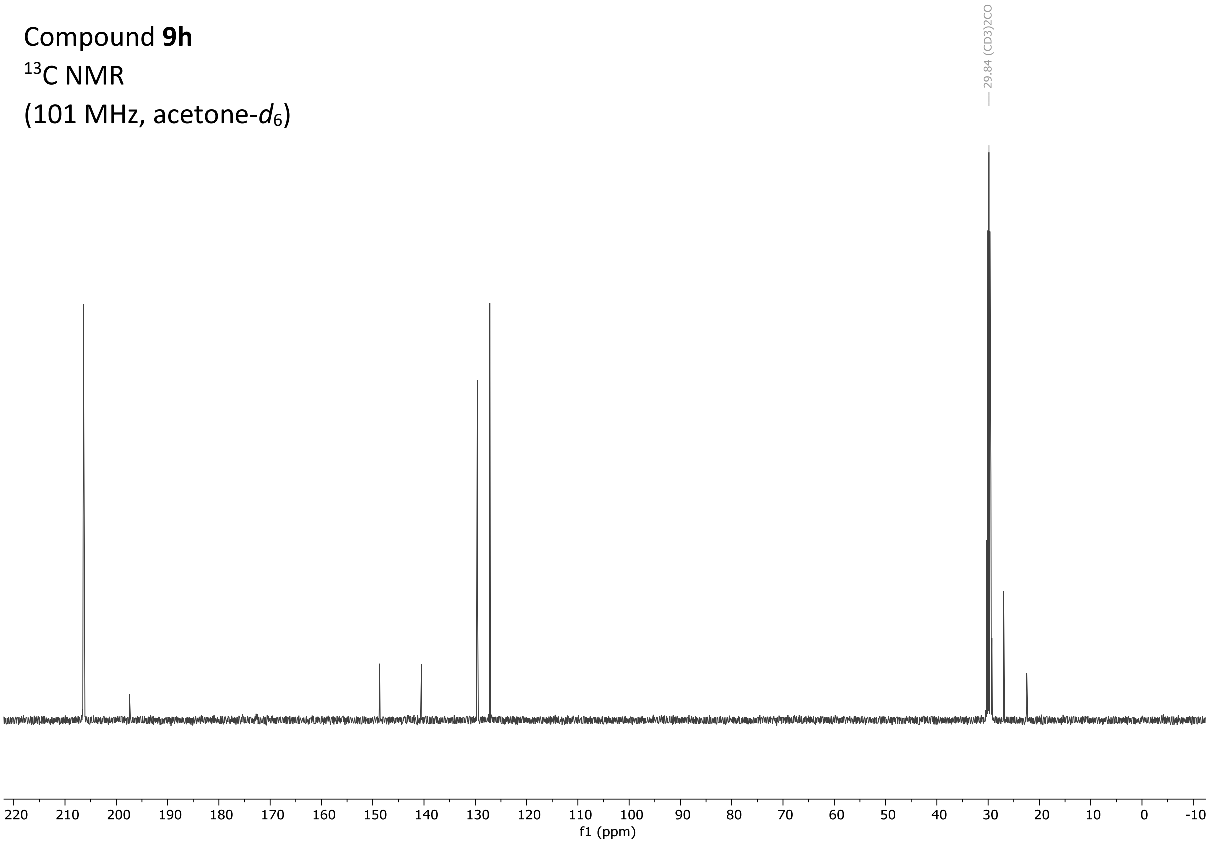

Compound **9i**
^{1}H NMR
(400 MHz, acetone-d_6)

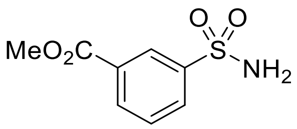

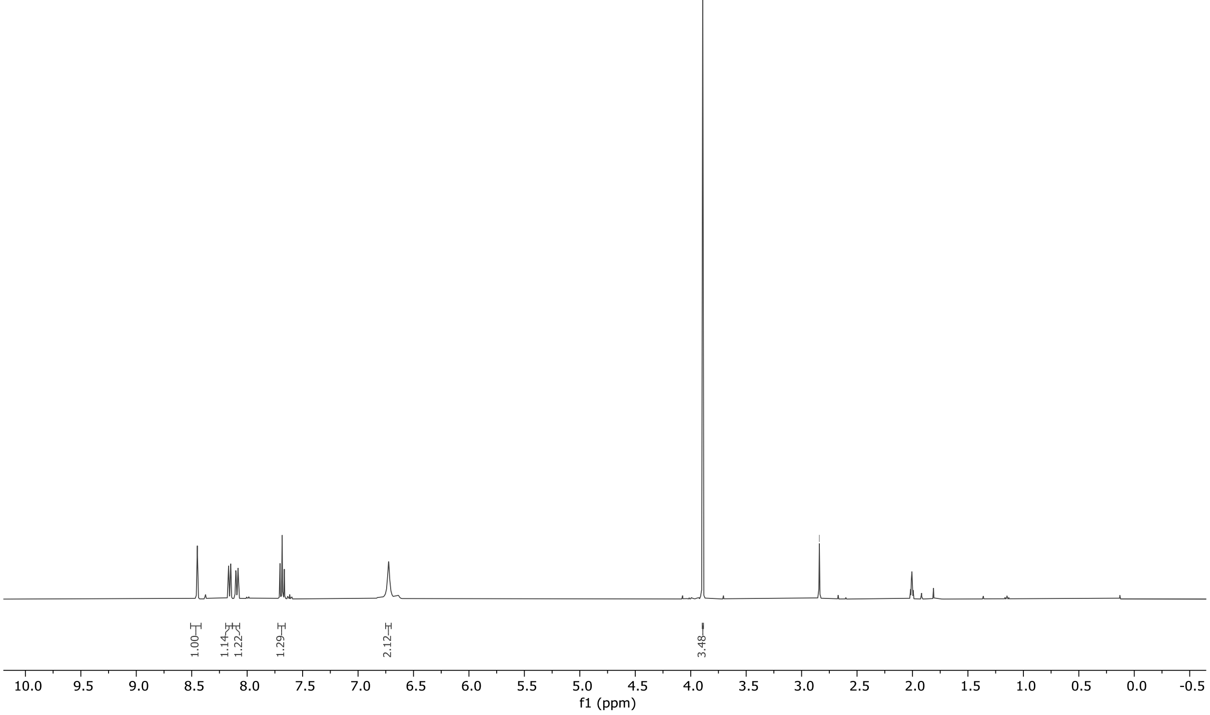

Compound **9i**
^{13}C NMR
(101 MHz, acetone-d_6)

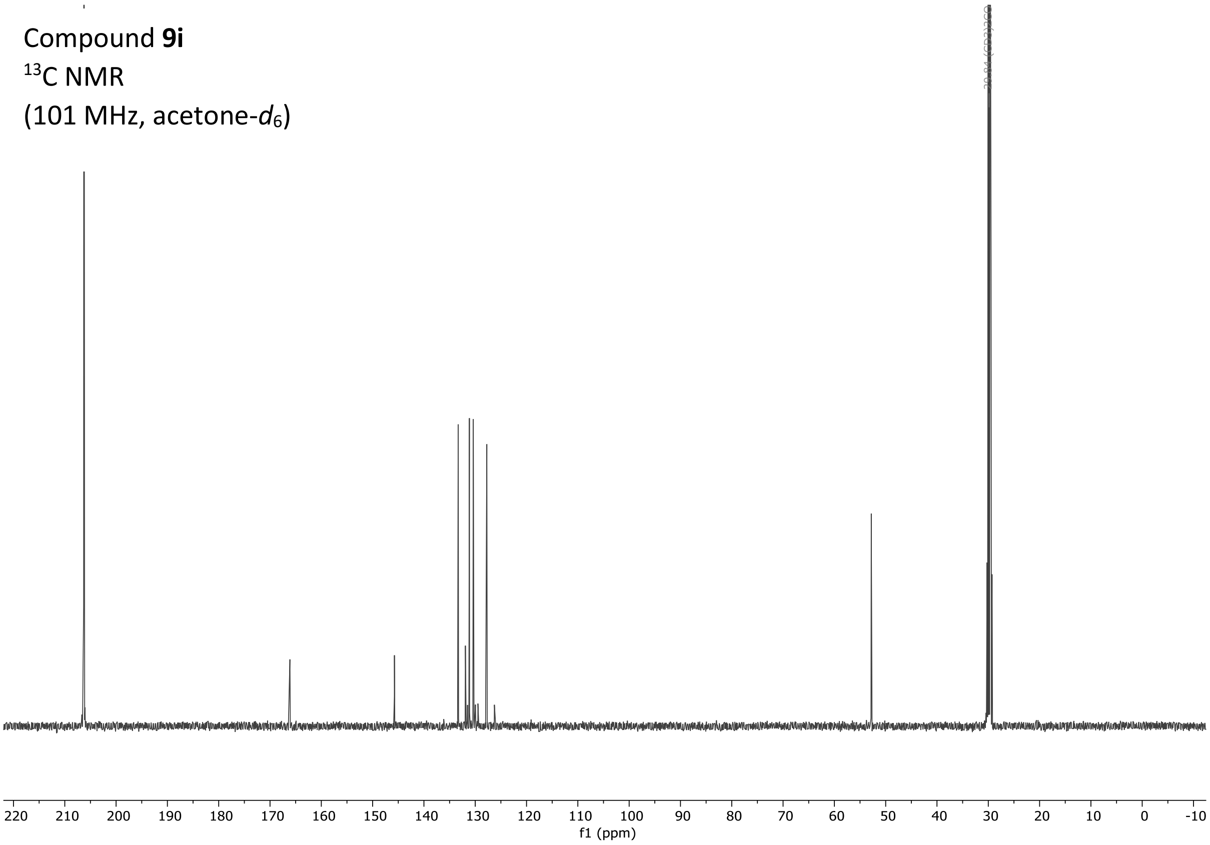

Compound **9k**
^{1}H NMR
(400 MHz, MeOD-d_4)

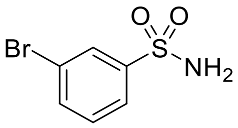

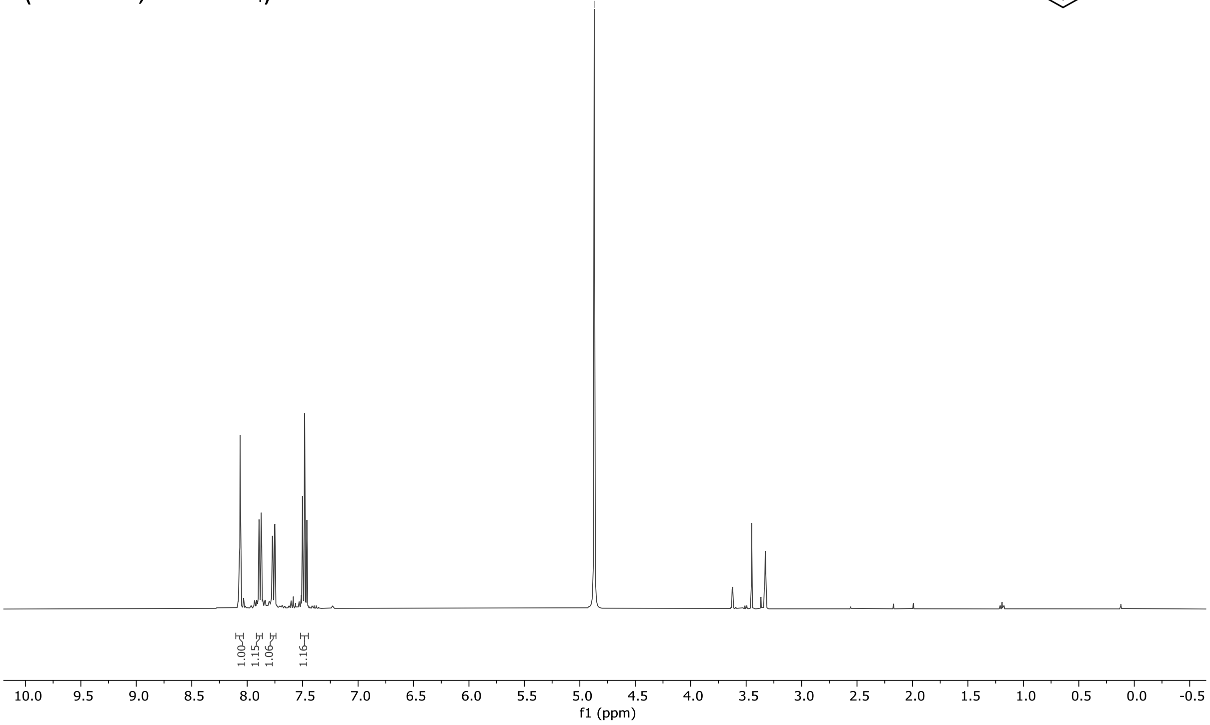

Compound **9k**
^{13}C NMR
(101 MHz, MeOD-d_4)

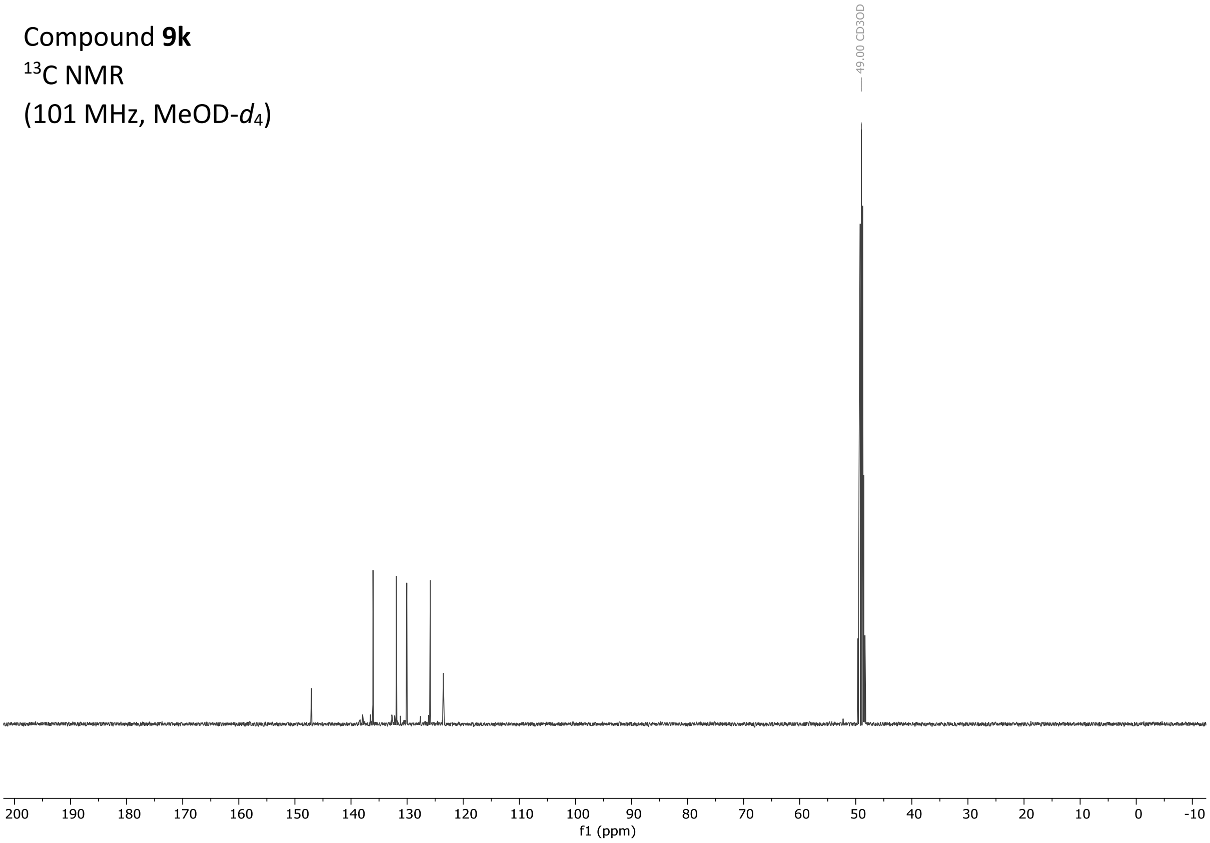

Compound **9l**
^{1}H NMR
(400 MHz, acetone-d_6)

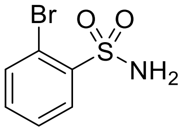

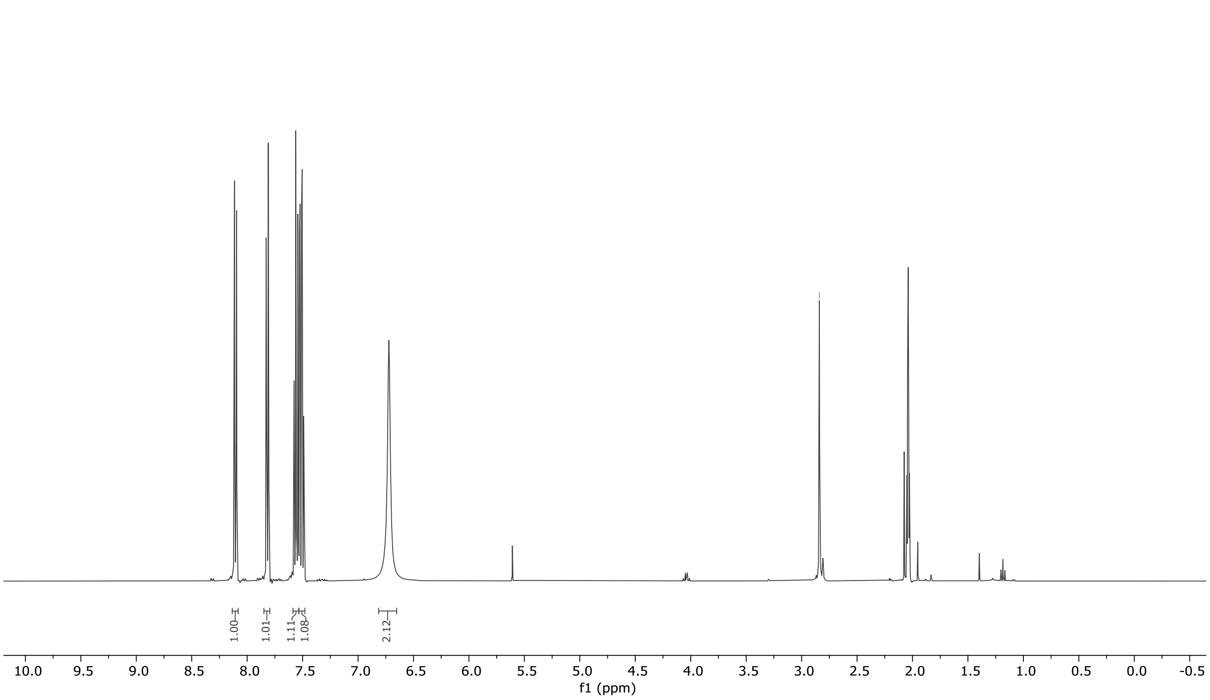

Compound **9l**
^{13}C NMR
(101 MHz, acetone-d_6)

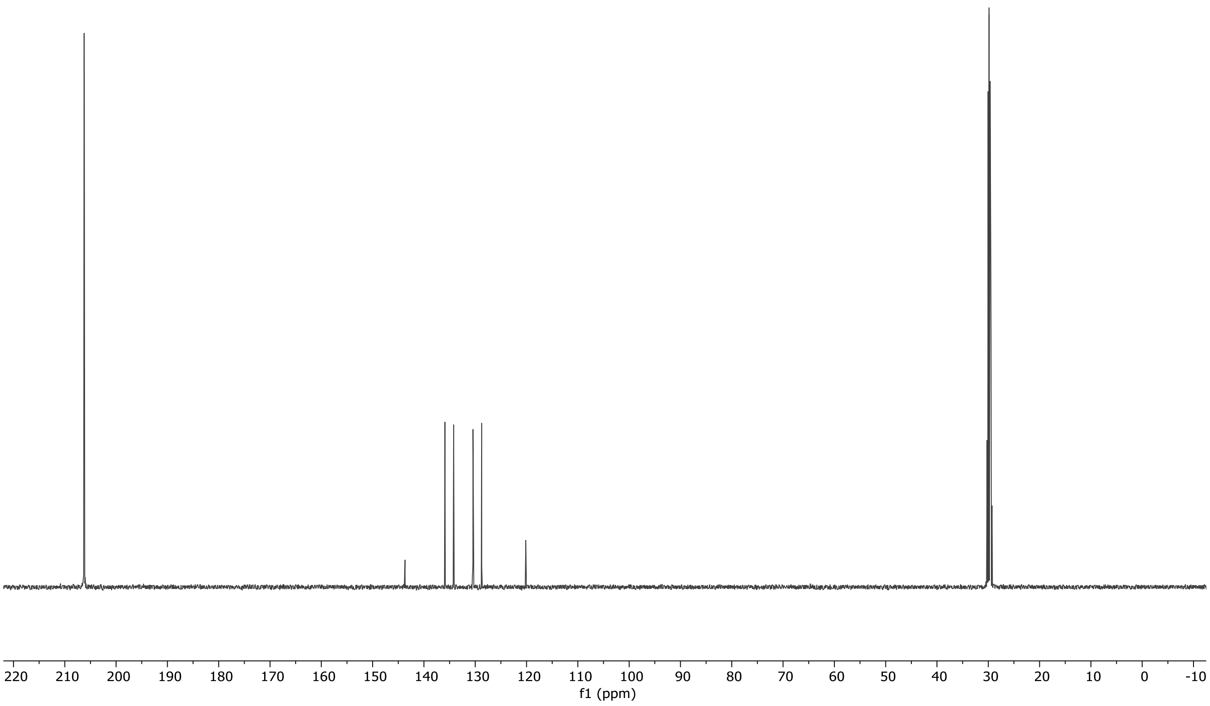

Compound **9n**
^{1}H NMR
(400 MHz, acetone-d_6)

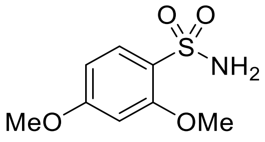

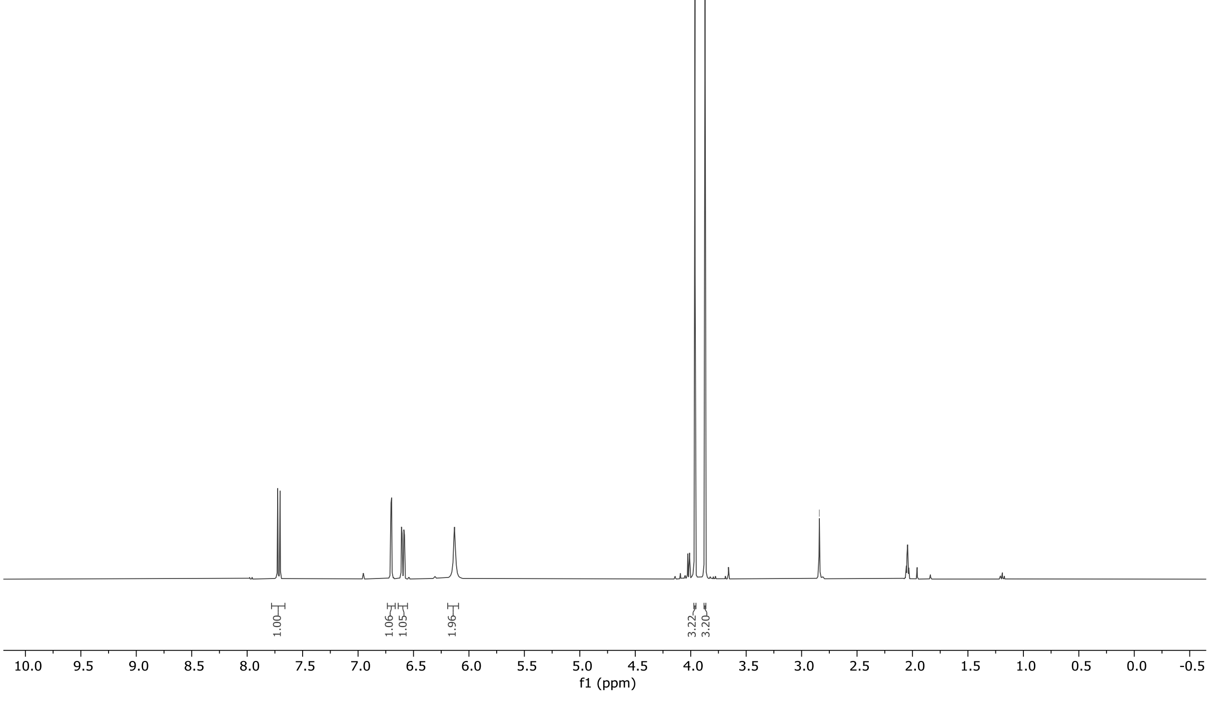

Compound **9n**
^{13}C NMR
(101 MHz, acetone-d_6)

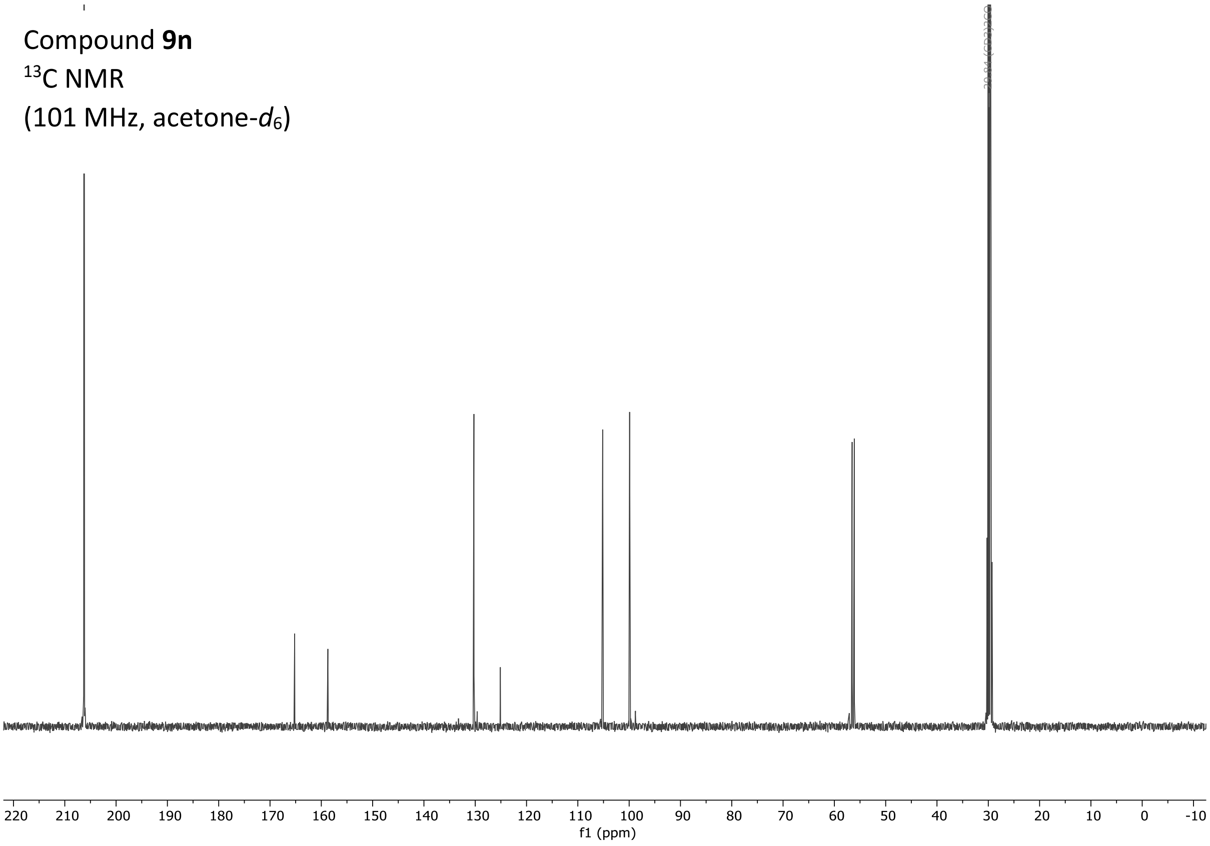

Compound **9p**
^{1}H NMR
(400 MHz, DMSO-d_6)

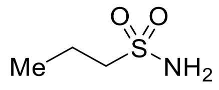

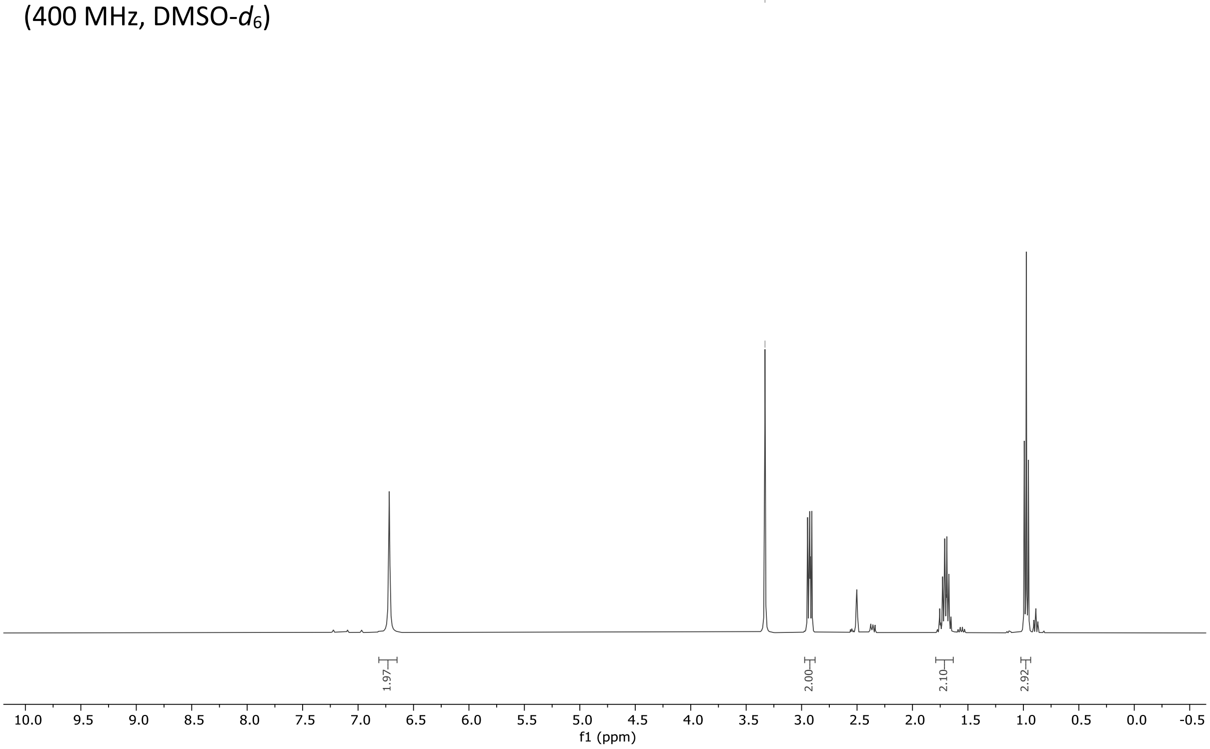

Compound **9p**
^{13}C NMR
(101 MHz, DMSO-d_6)

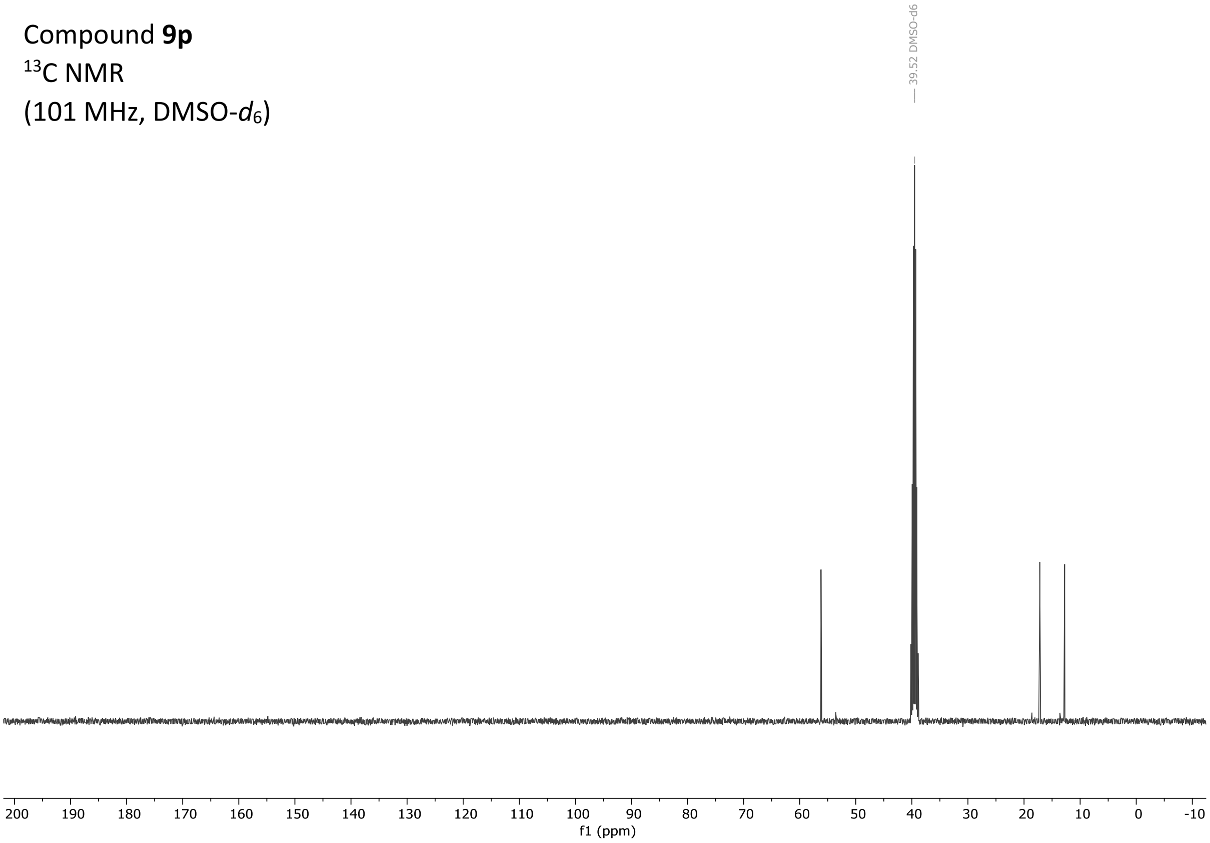

Compound **9q**
^{1}H NMR
(400 MHz, $CDCl_3$)

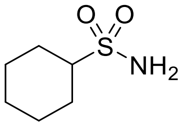

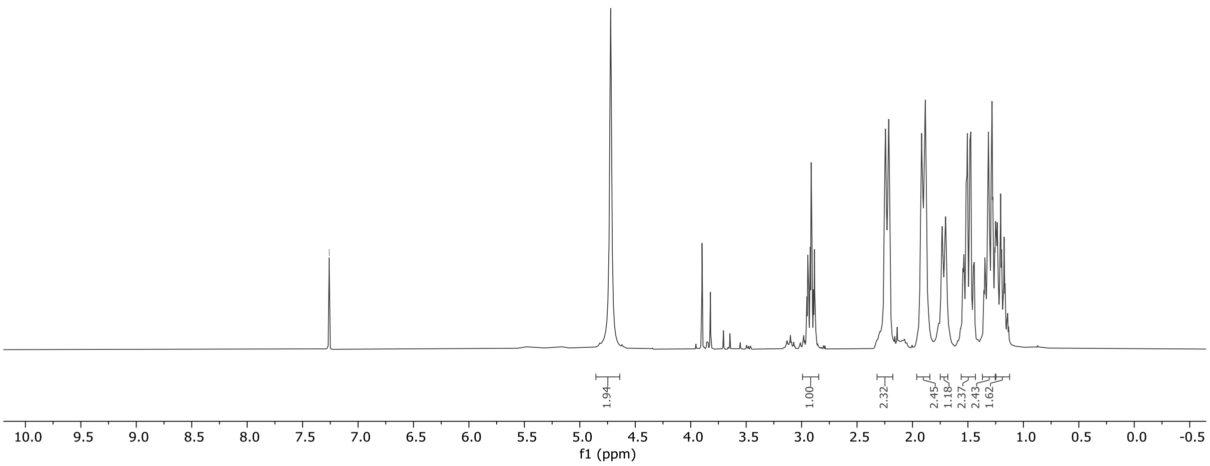

Compound **9q**
^{13}C NMR
(101 MHz, $CDCl_3$)

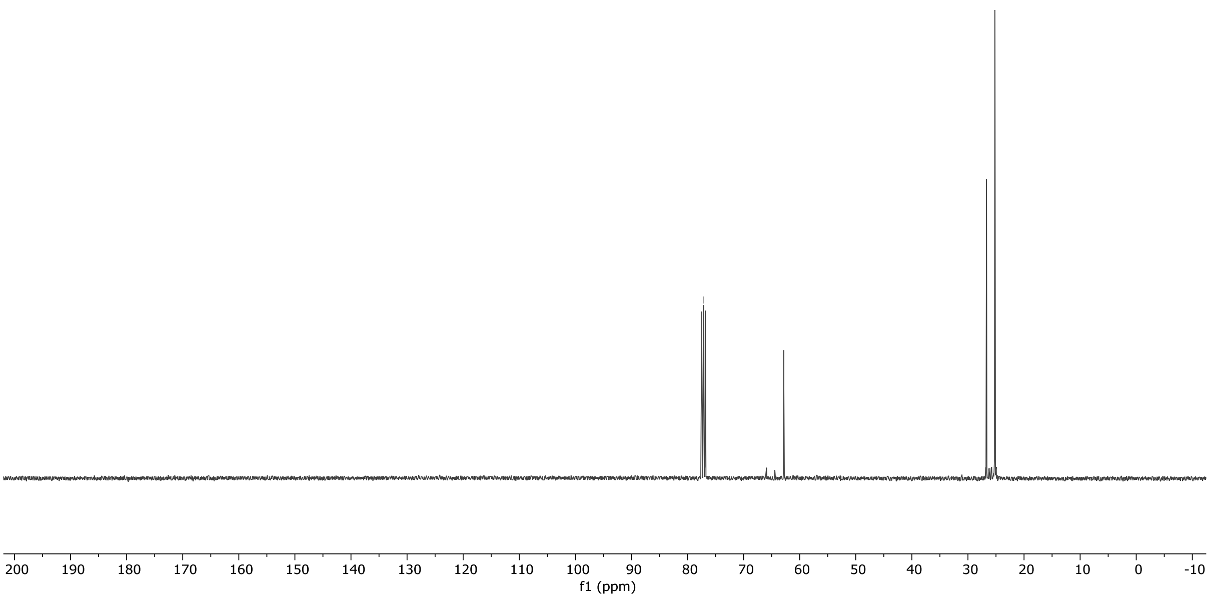

Compound **9s**
^{1}H NMR
(400 MHz, acetone-d_6)

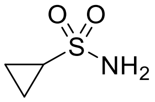

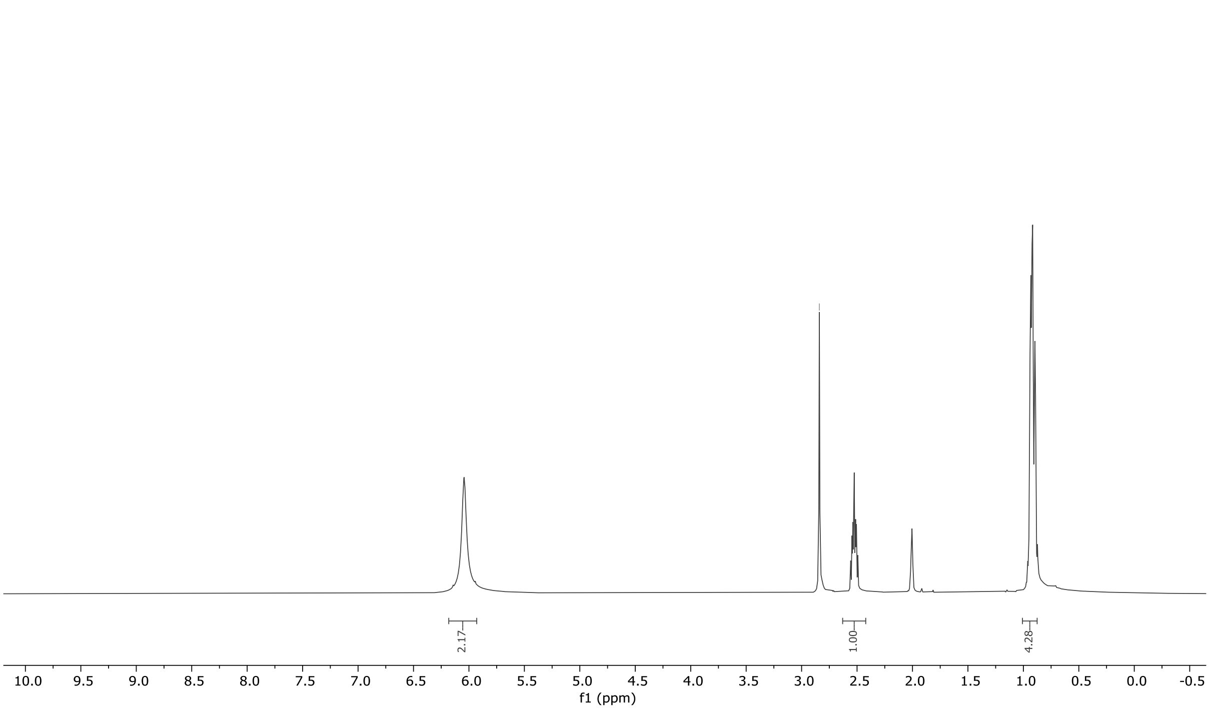

Compound **9s**
^{13}C NMR
(101 MHz, acetone-d_6)

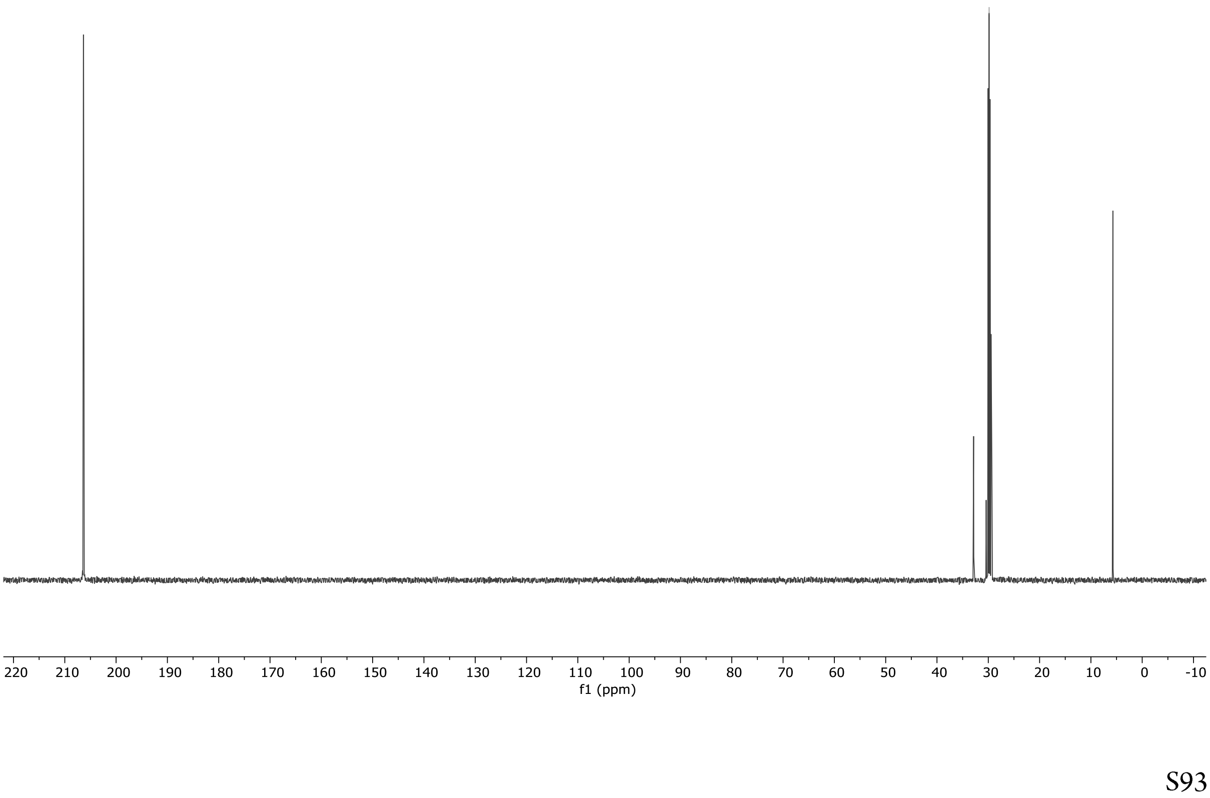

Compound **9u**
^{1}H NMR
(400 MHz, acetone-d_6)

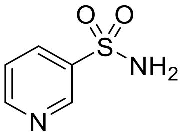

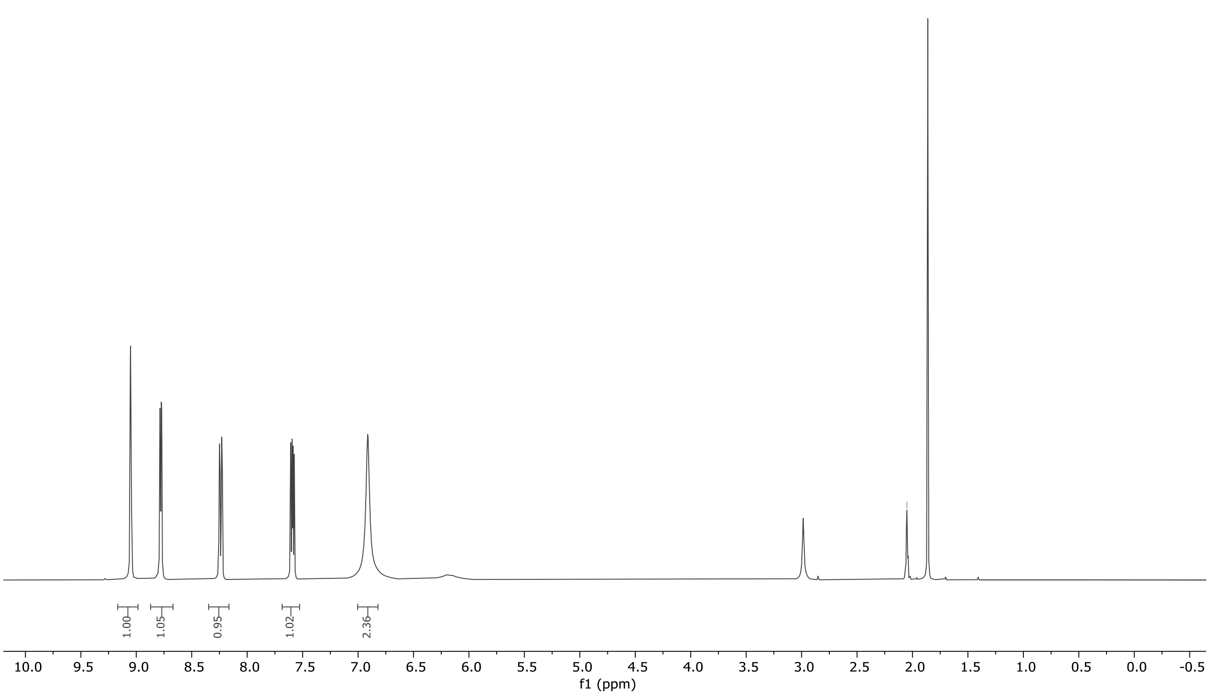

Compound **9u**
^{13}C NMR
(101 MHz, acetone-d_6)

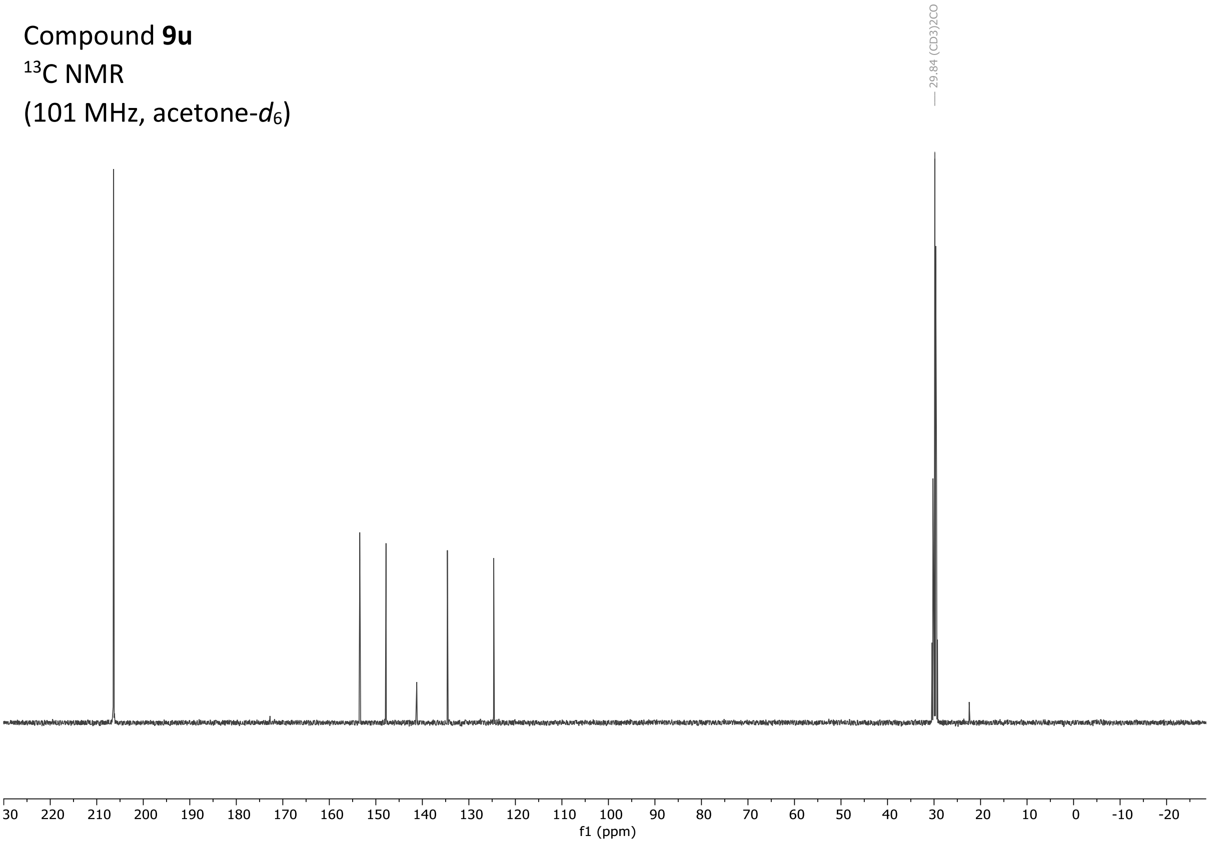

Compound **9w**
^{1}H NMR
(400 MHz, acetone-d_6)

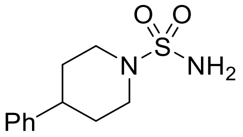

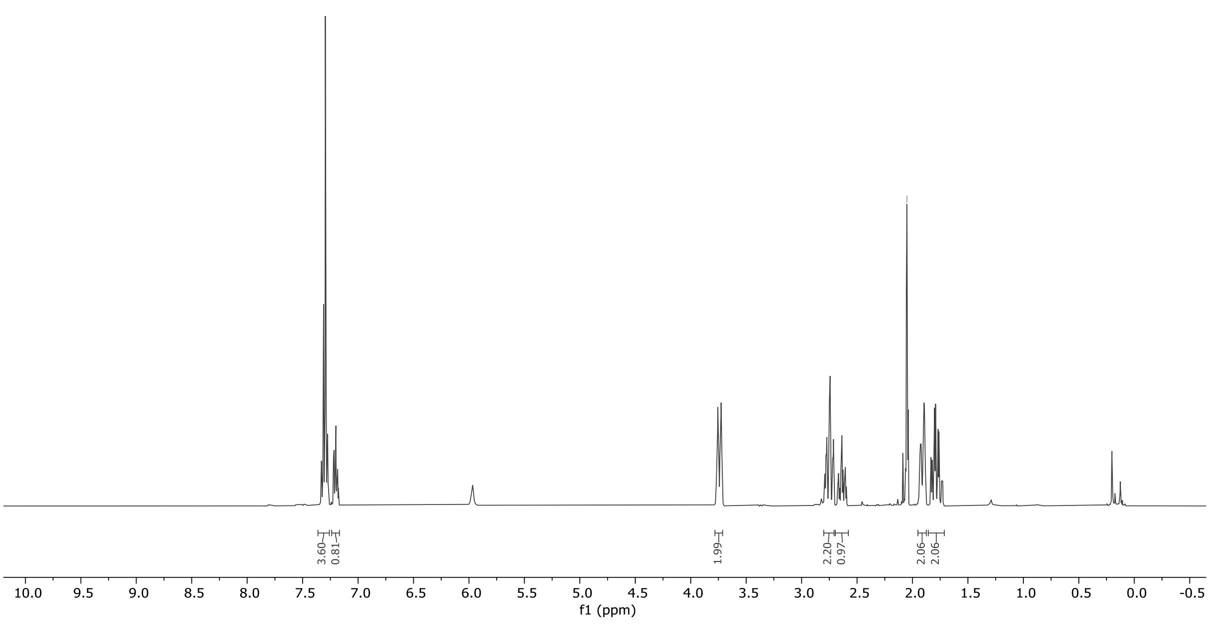

Compound **9w**
^{13}C NMR
(101 MHz, acetone-d_6)

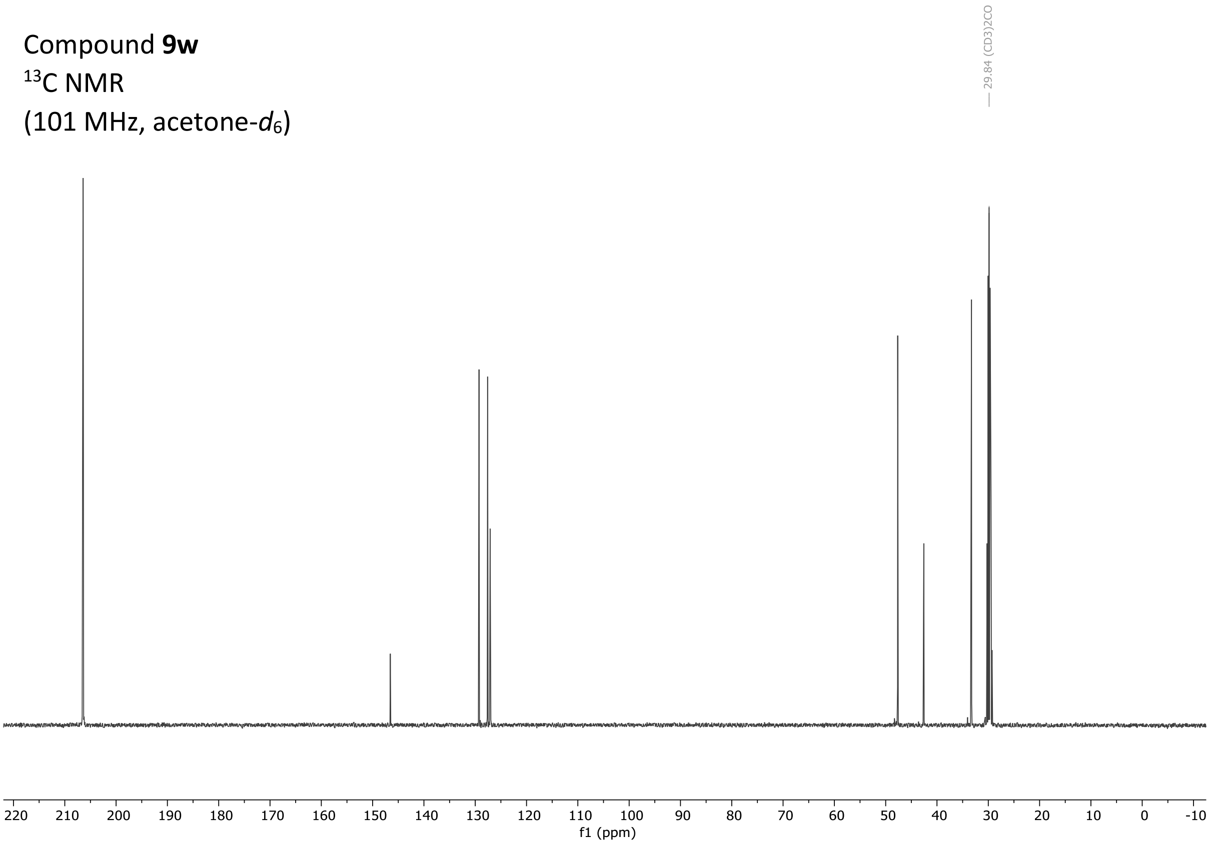

Compound **9x**
^{1}H NMR
(400 MHz, DMSO-d_6)

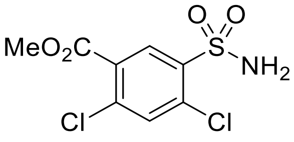

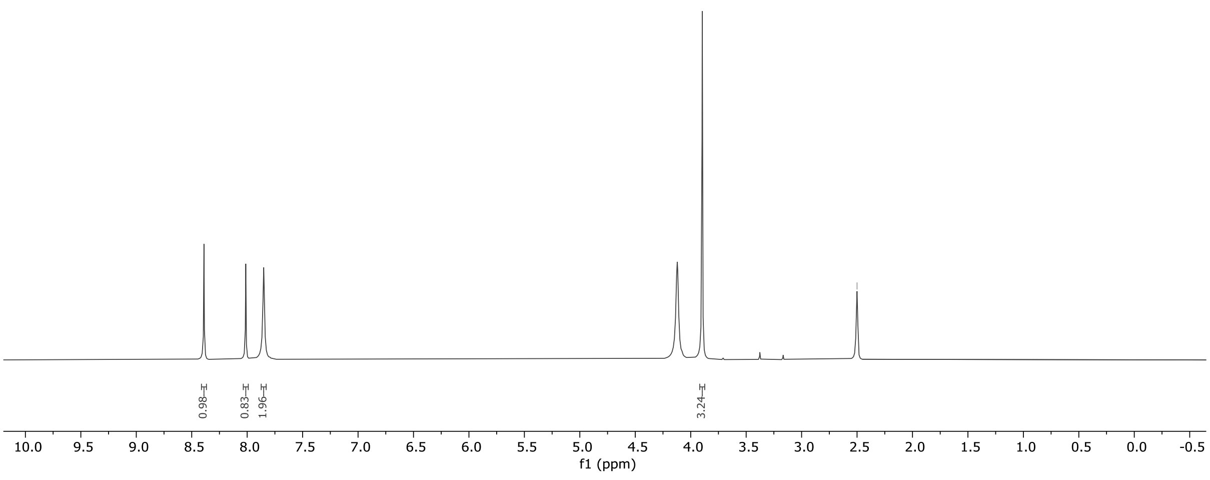

Compound **9x**
^{13}C NMR
(101 MHz, DMSO-d_6)

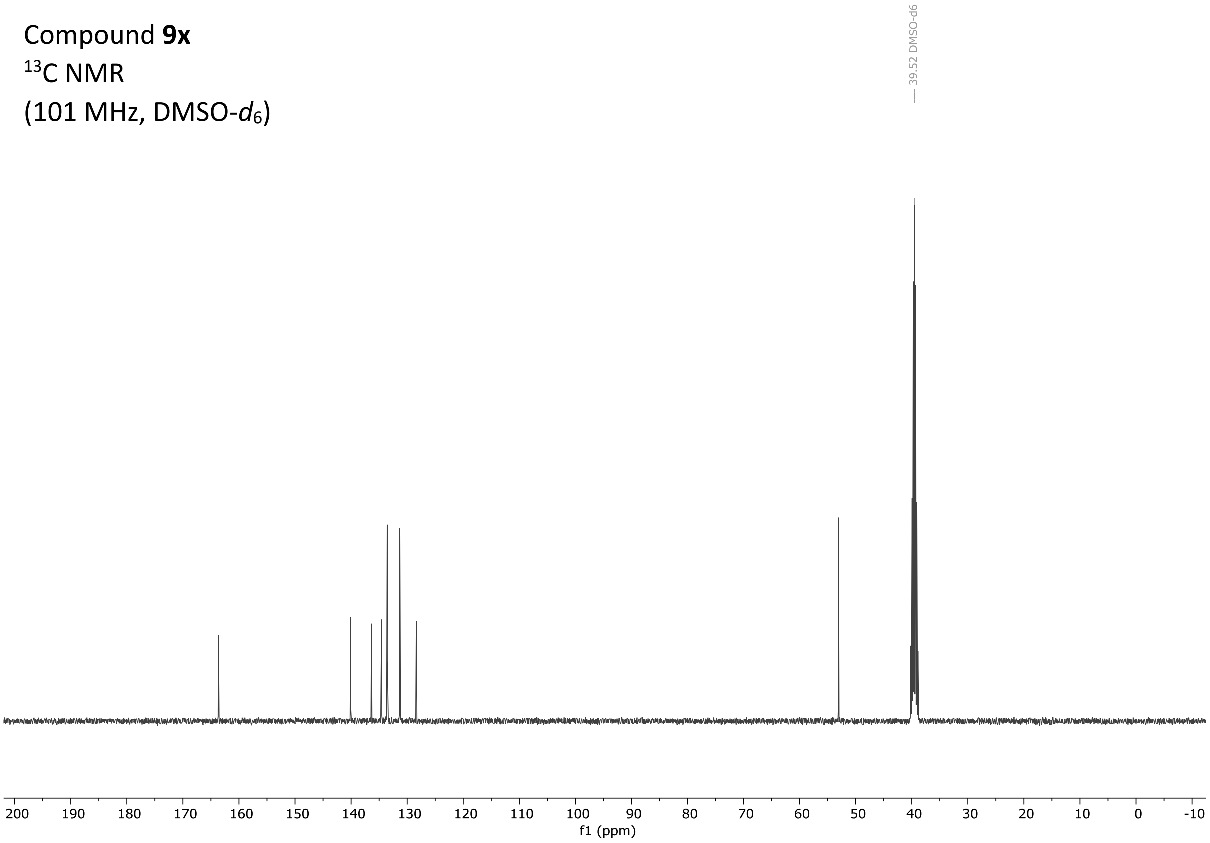

Compound **9z**
^{1}H NMR
(400 MHz, DMSO-d_6)

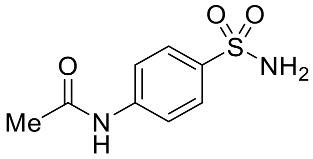

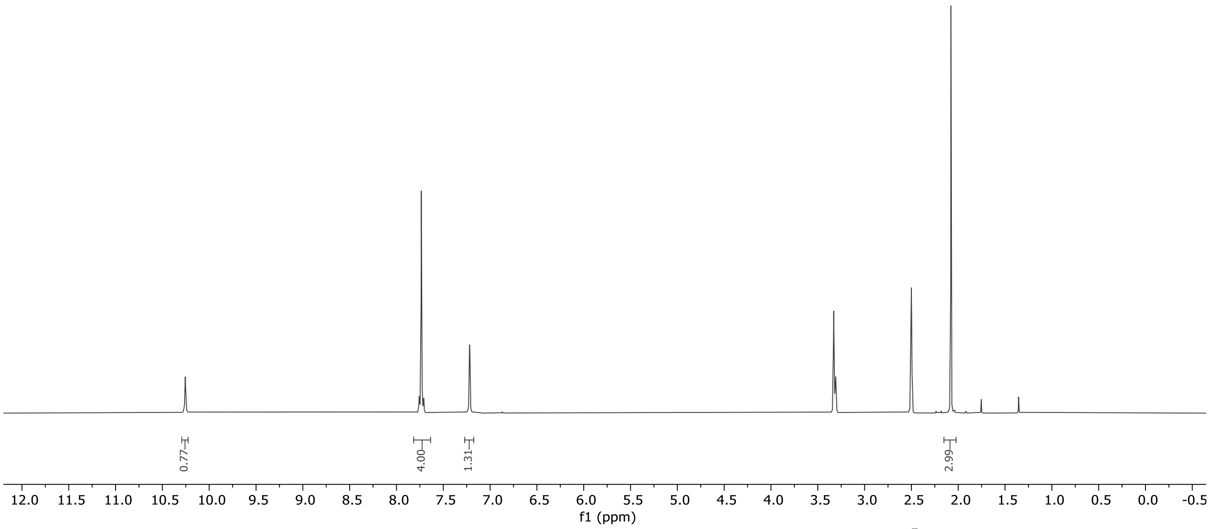

Compound **9z**
^{13}C NMR
(101 MHz, DMSO-d_6)

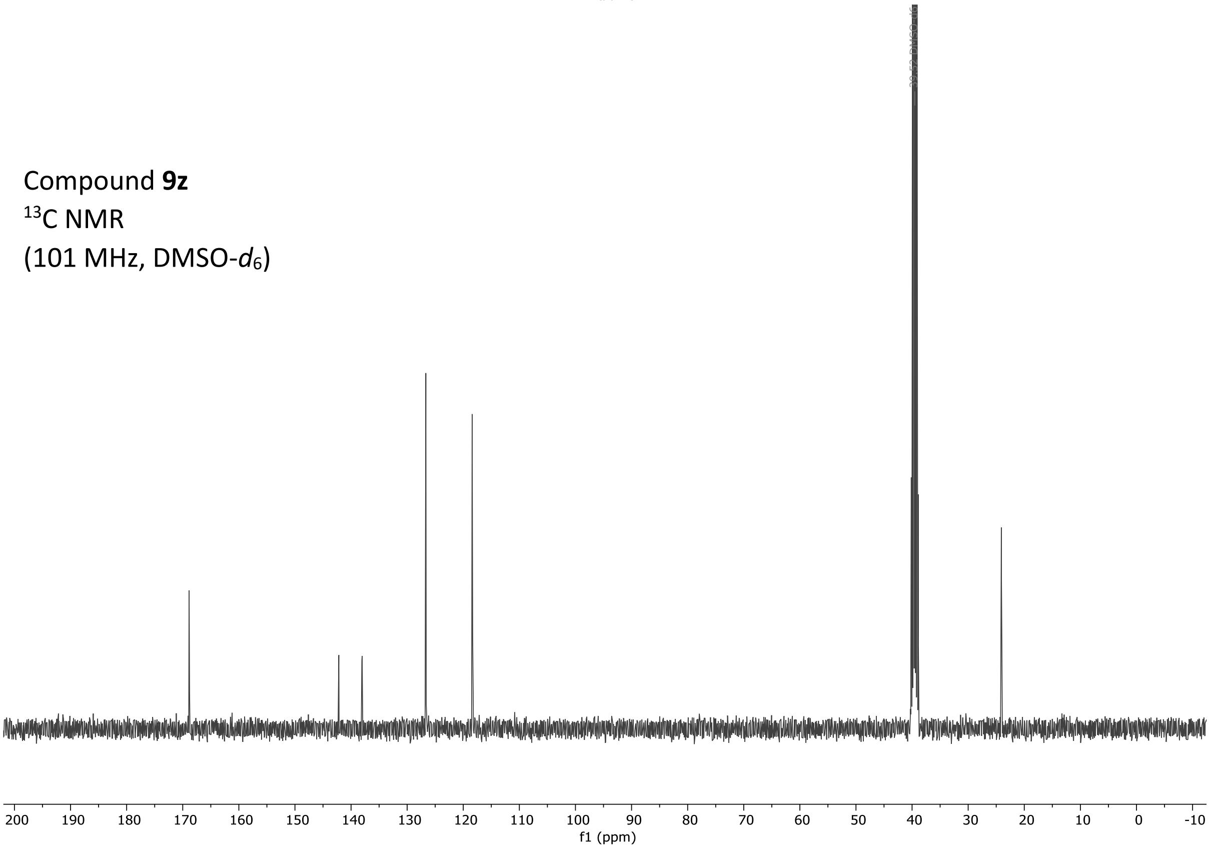

Compound **9ab**
^{1}H NMR
(400 MHz, DMSO-d_6)

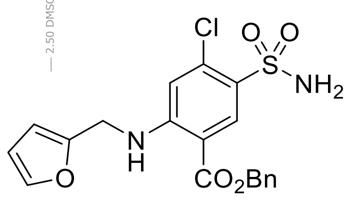

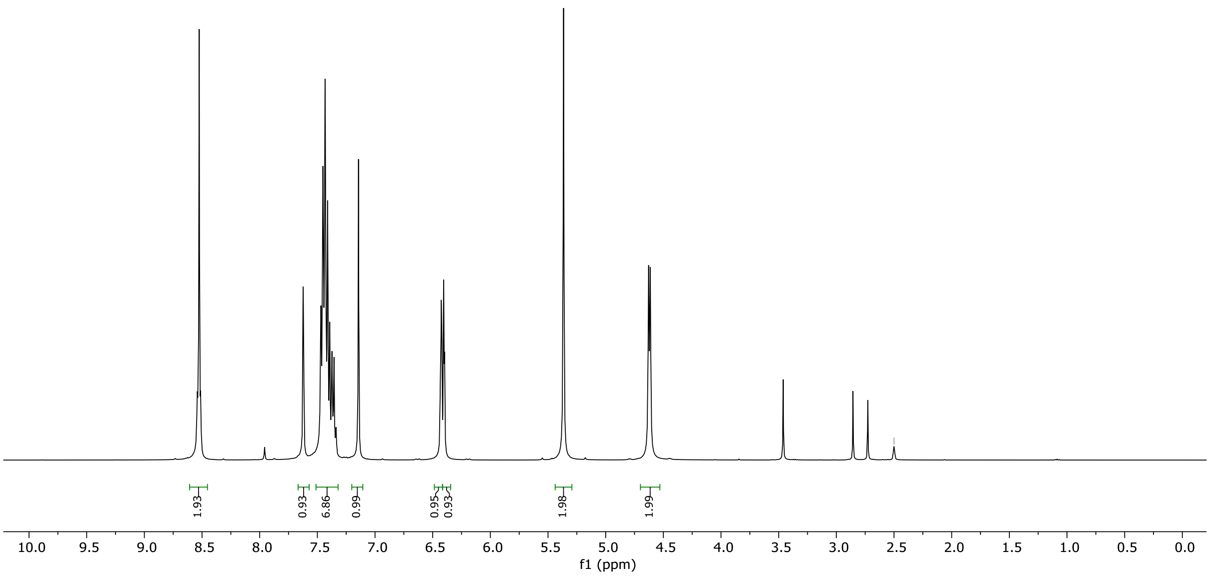

Compound **9ab**
^{13}C NMR
(101 MHz, DMSO-d_6)

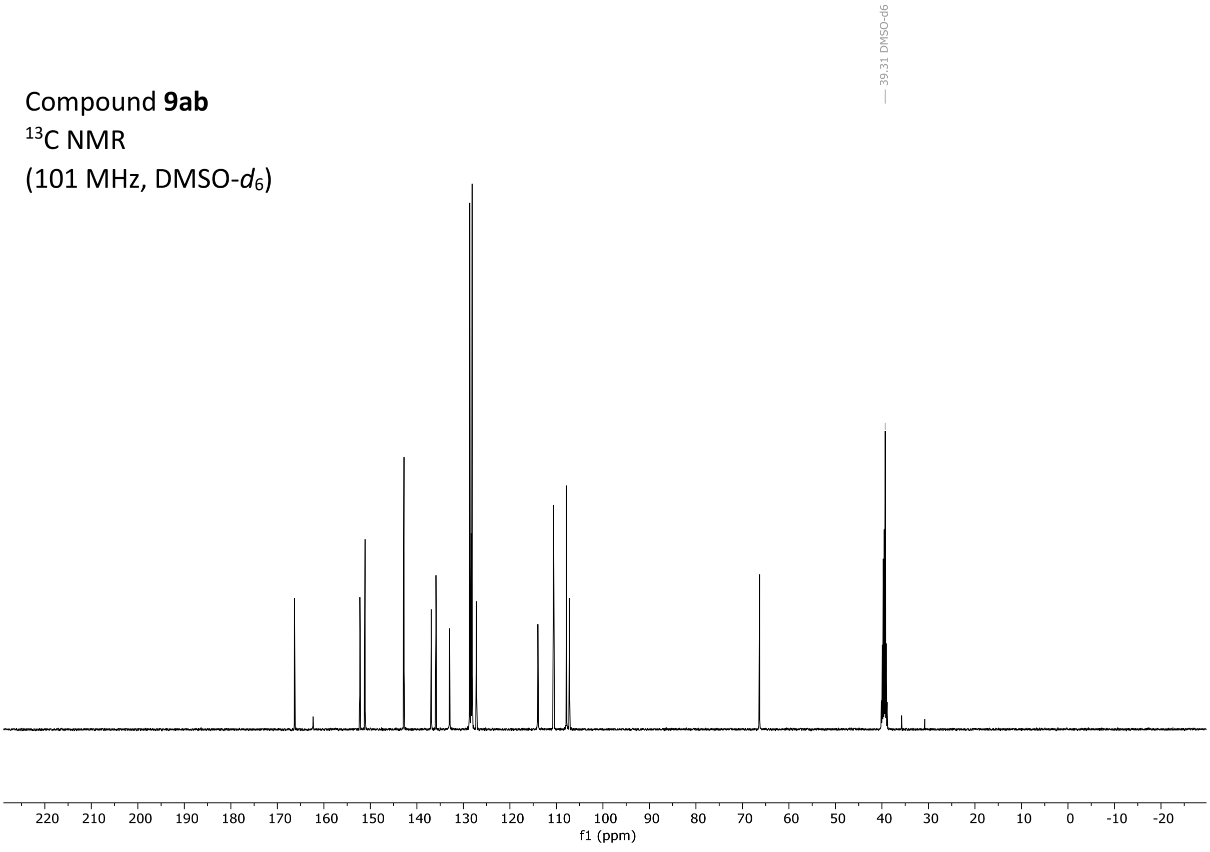

Compound **1a**
^{1}H NMR
(400 MHz, $CDCl_3$)

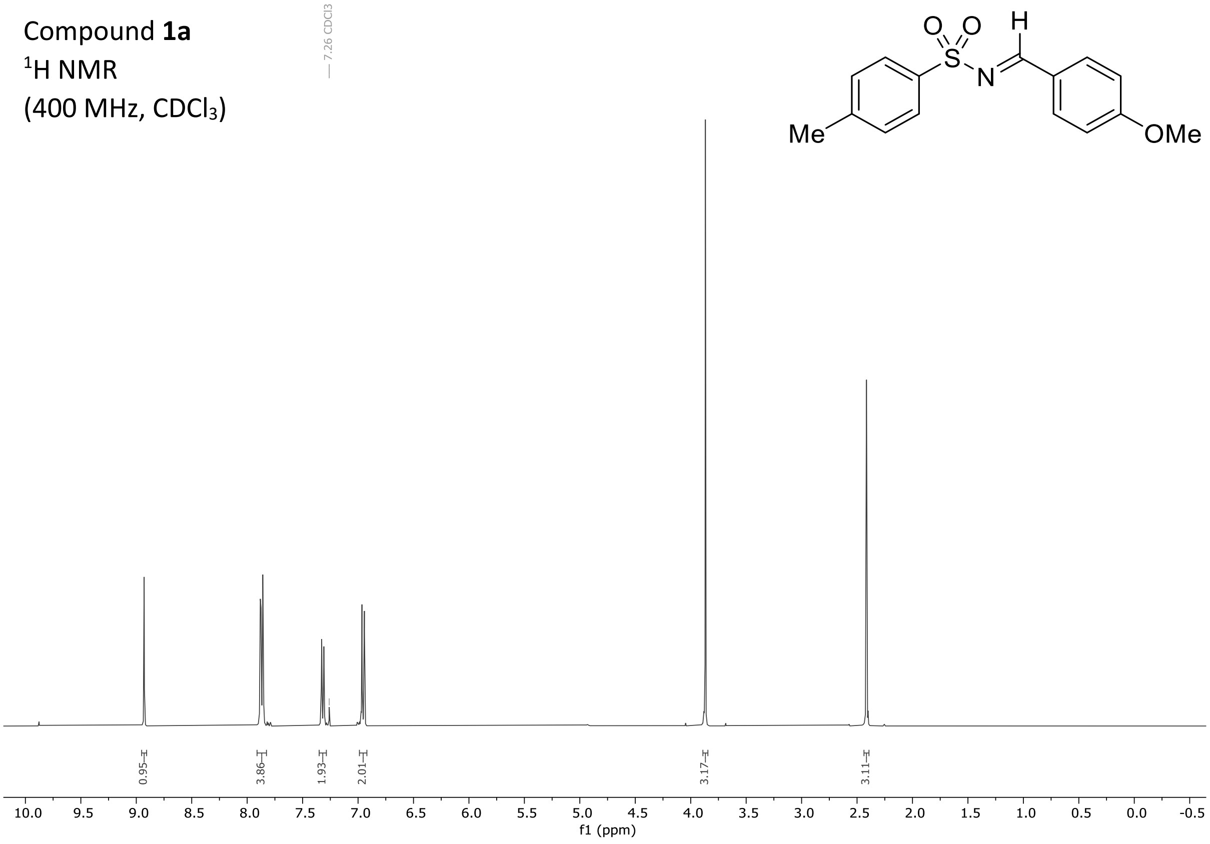

Compound **1a**
^{13}C NMR
(101 MHz, $CDCl_3$)

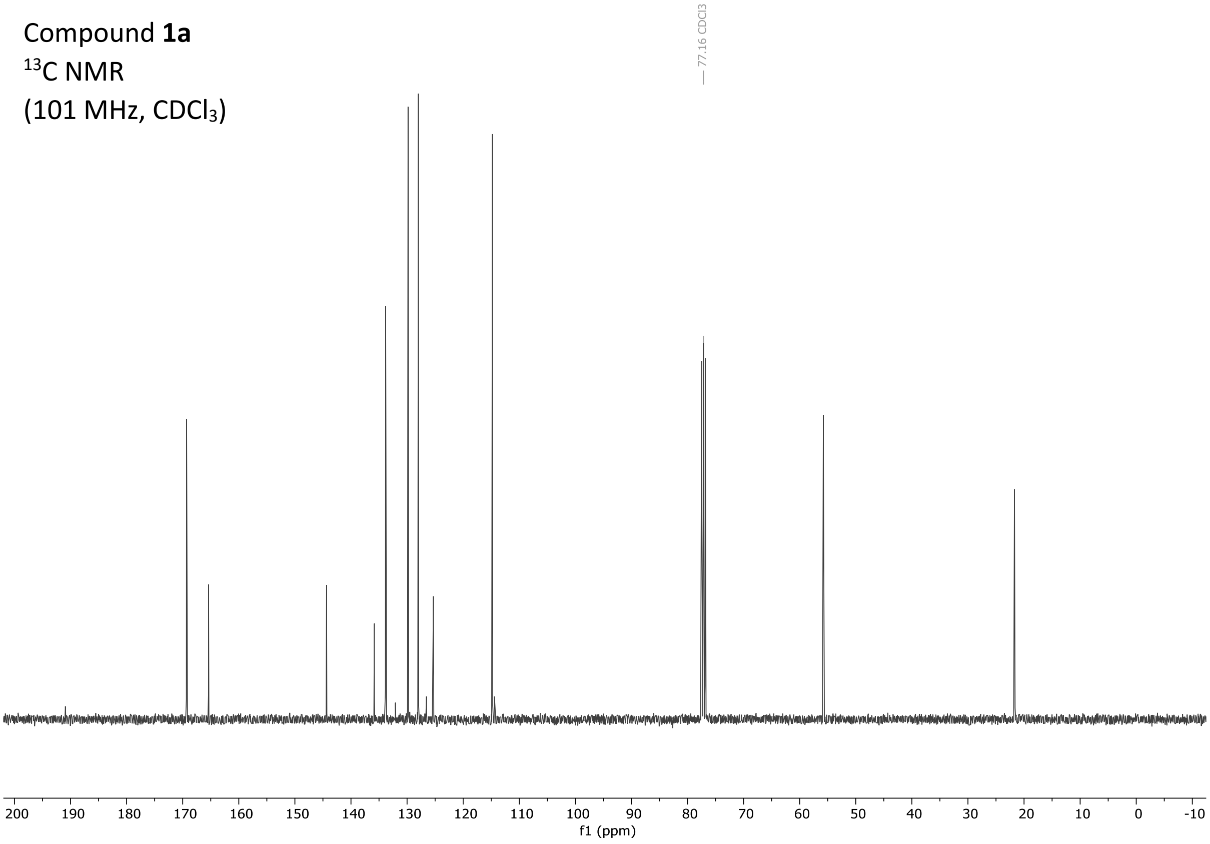

Compound **1a-*d*$_1$**
^{1}H NMR
(400 MHz, $CDCl_3$)

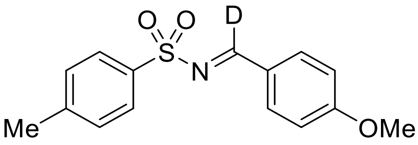

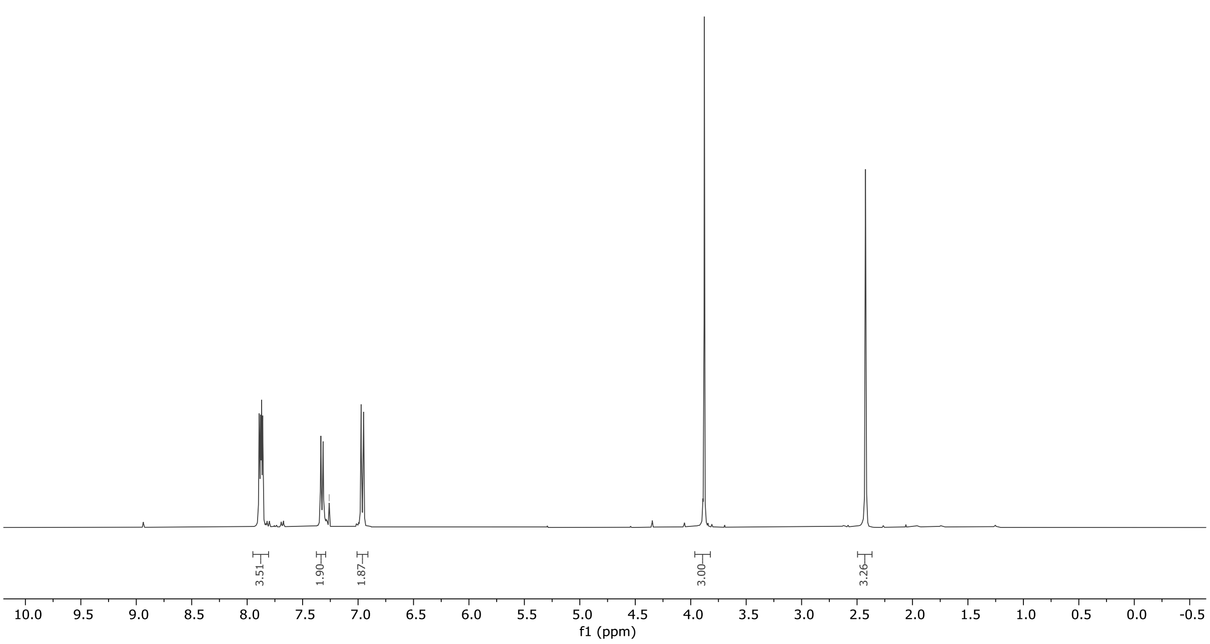

Compound **1a-*d*$_1$**
^{13}C NMR
(101 MHz, $CDCl_3$)

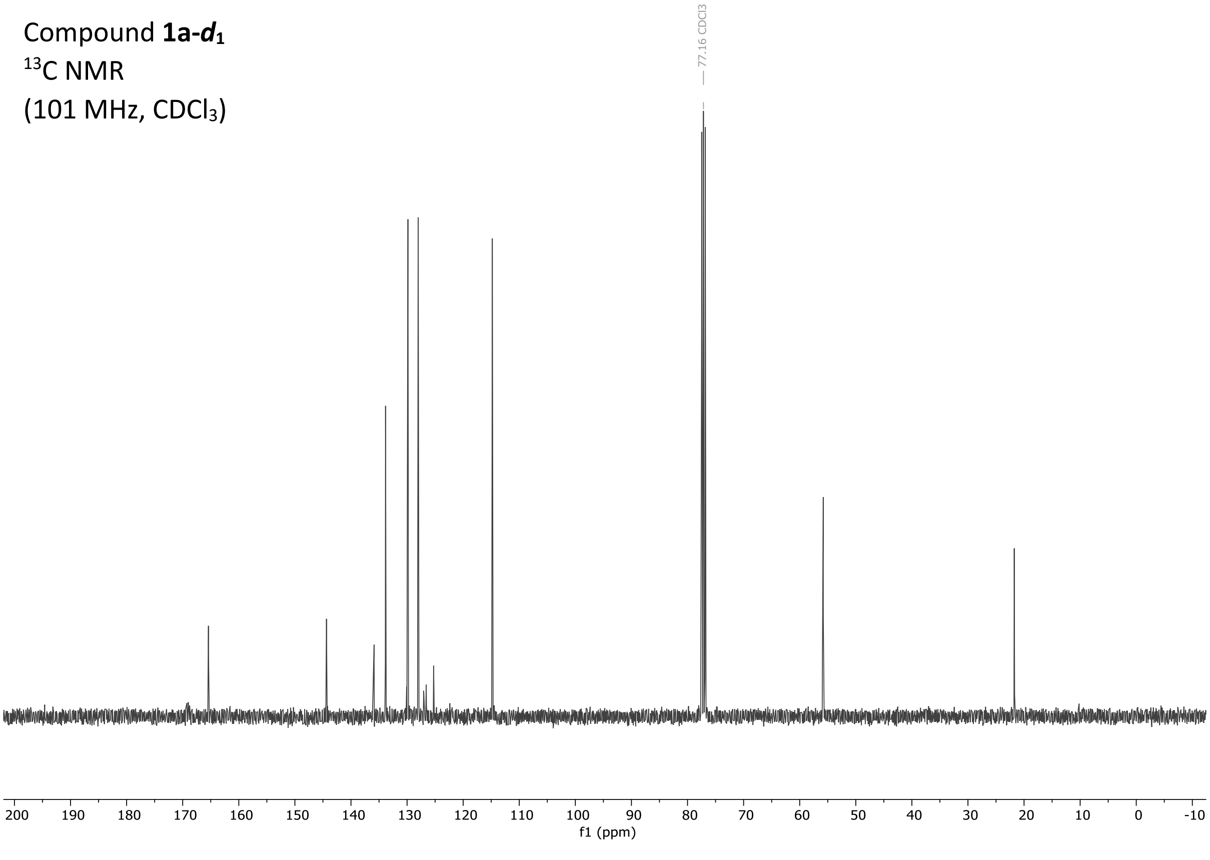

Compound **1b**
^{1}H NMR
(400 MHz, $CDCl_3$)

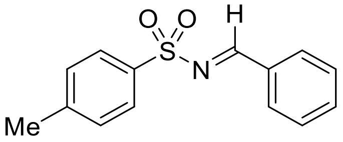

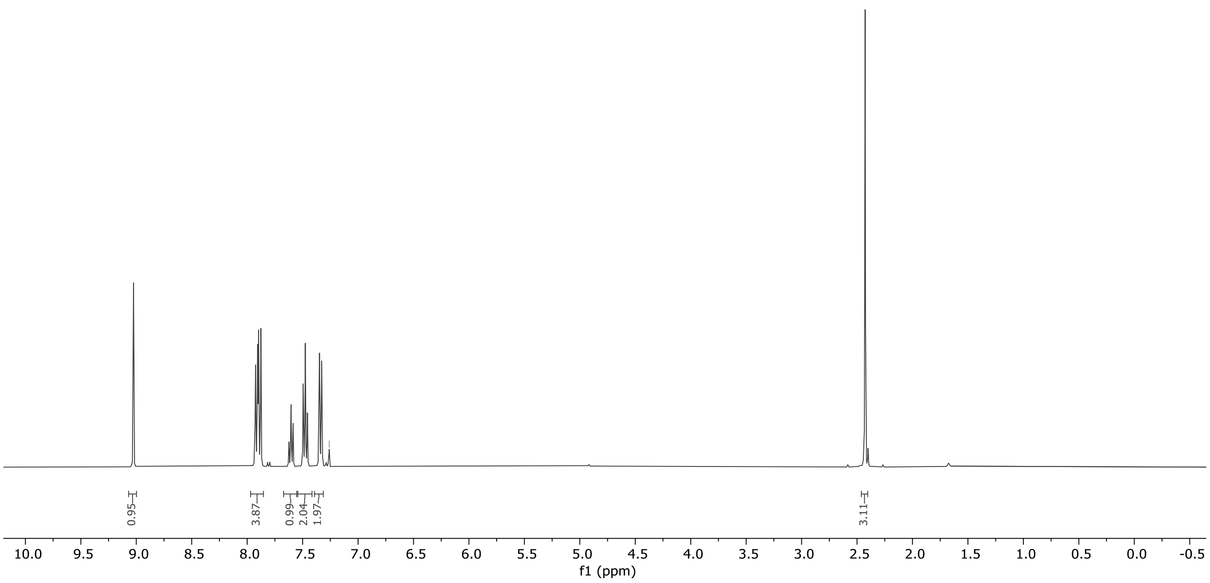

Compound **1b**
^{13}C NMR
(101 MHz, $CDCl_3$)

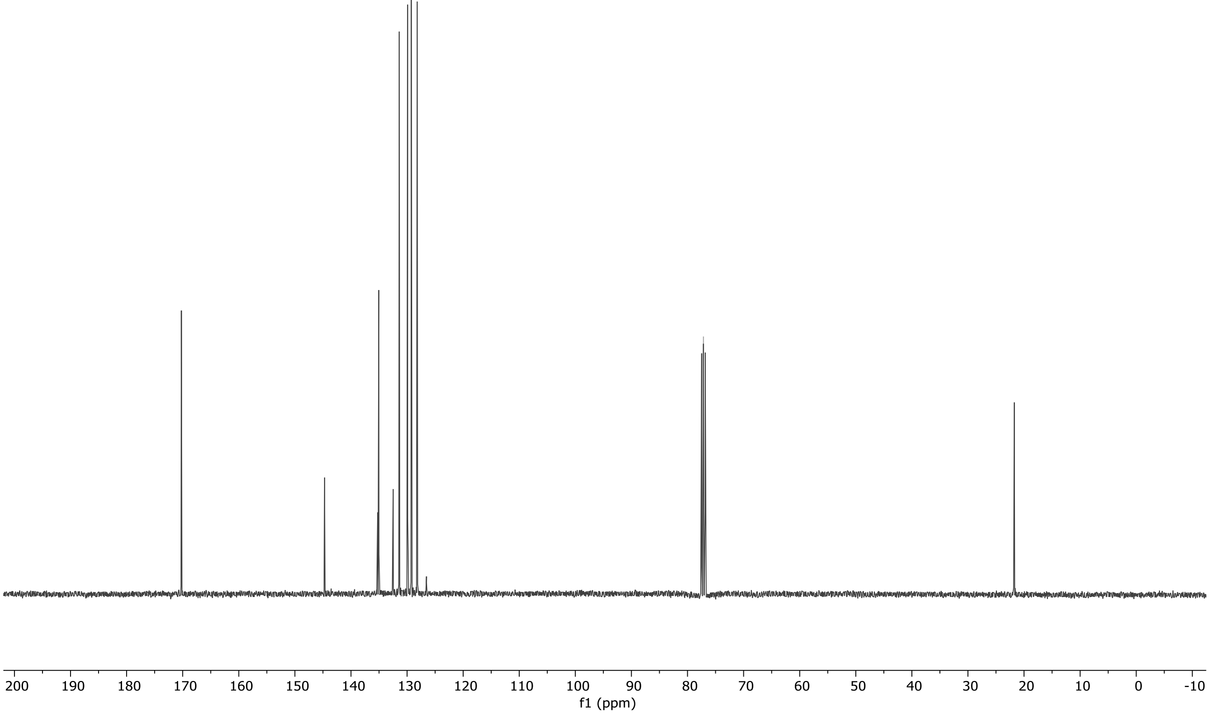

Compound **1c**
^{1}H NMR
(400 MHz, $CDCl_3$)

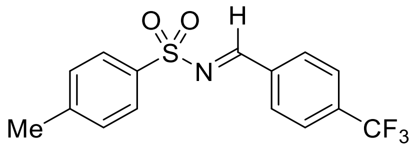

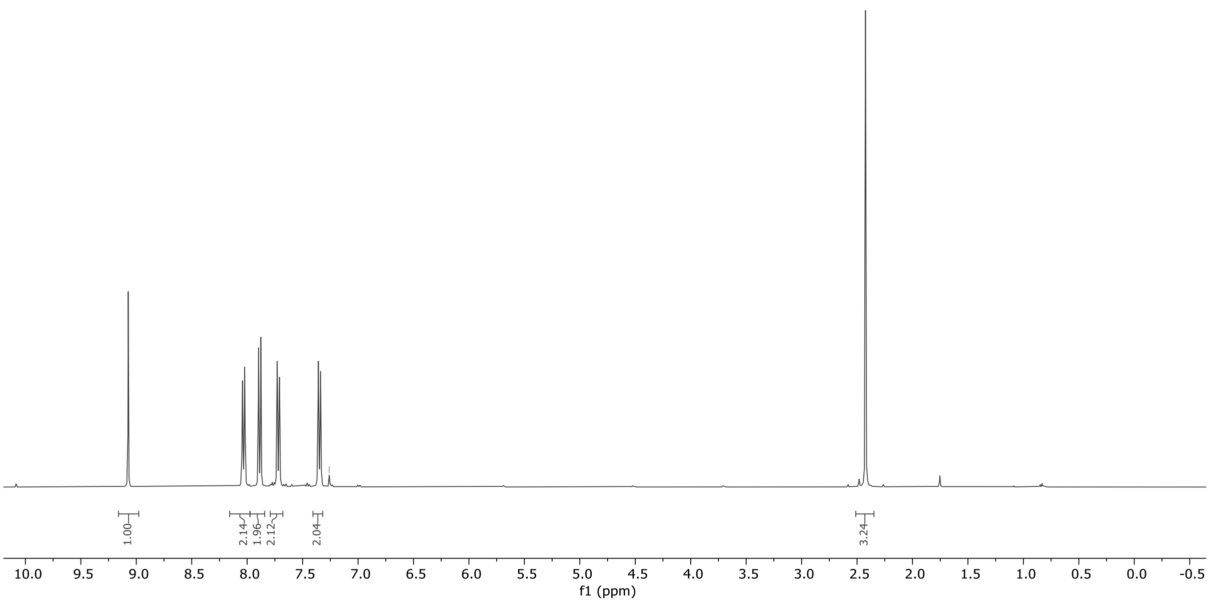

Compound **1c**
^{13}C NMR
(101 MHz, $CDCl_3$)

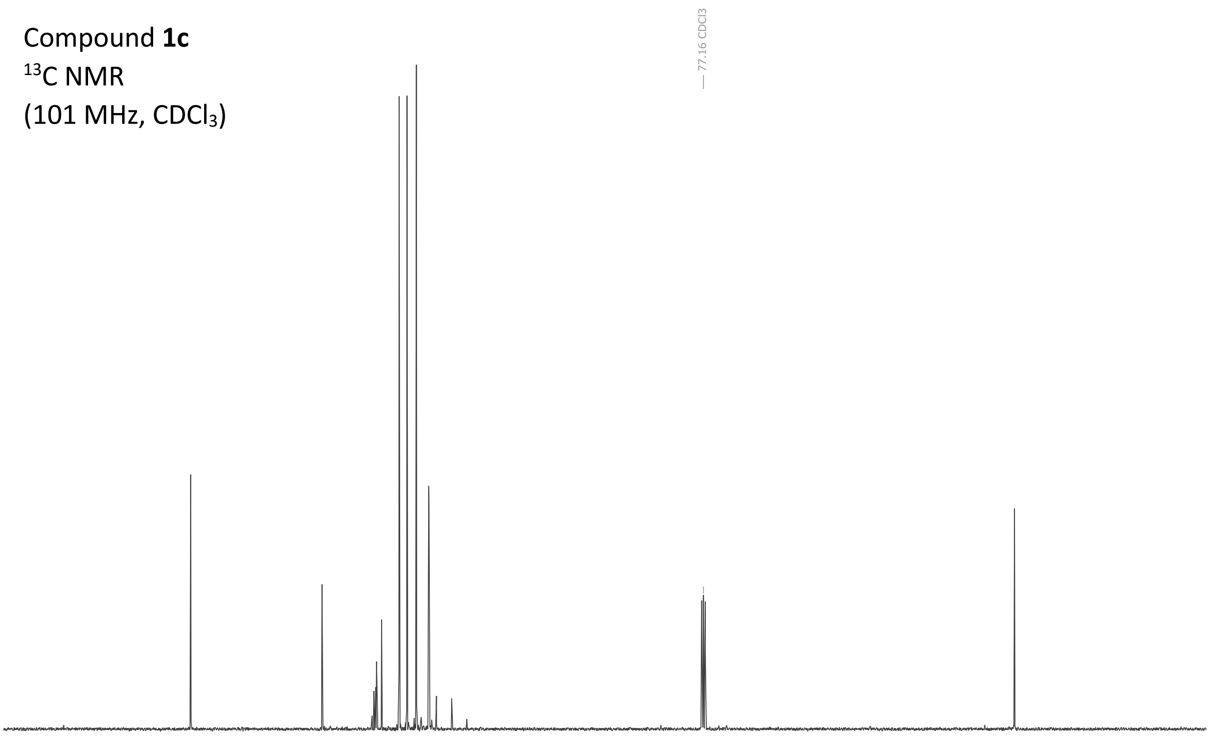

Compound **1c**
^{19}F NMR
(377 MHz, $CDCl_3$)

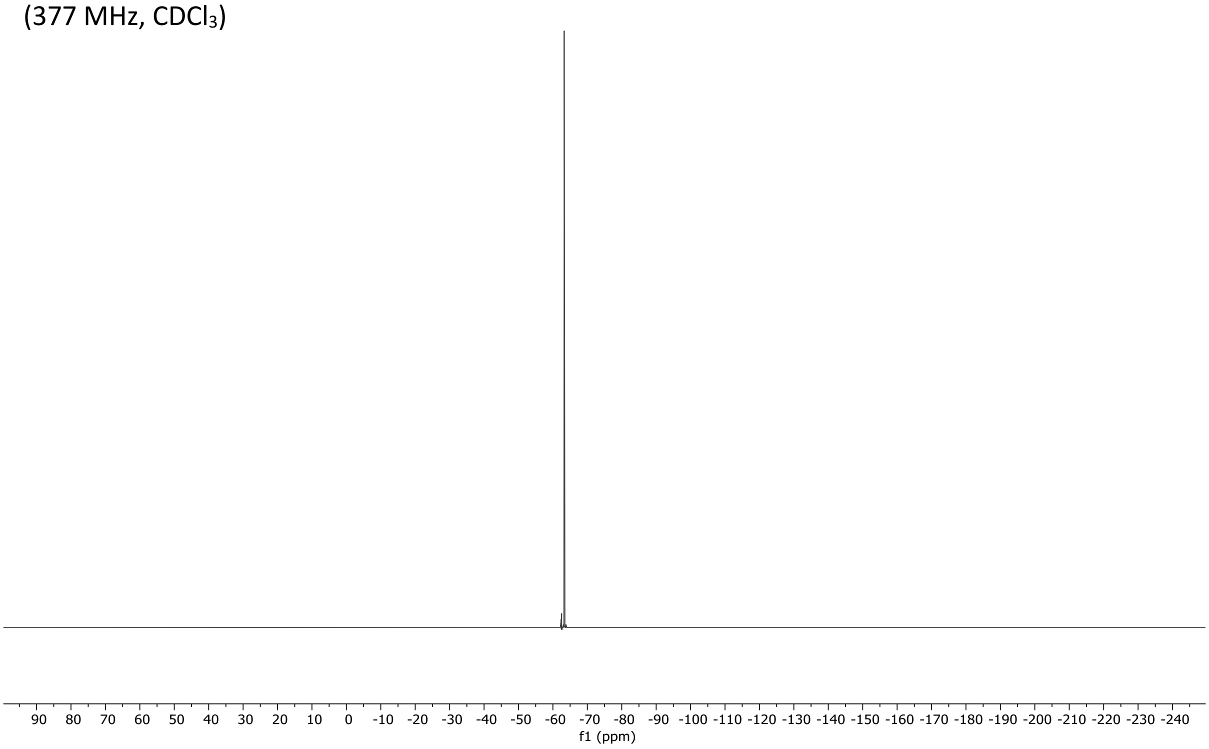

Compound **1d**
^{1}H NMR
(400 MHz, $CDCl_3$)

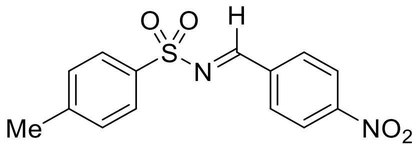

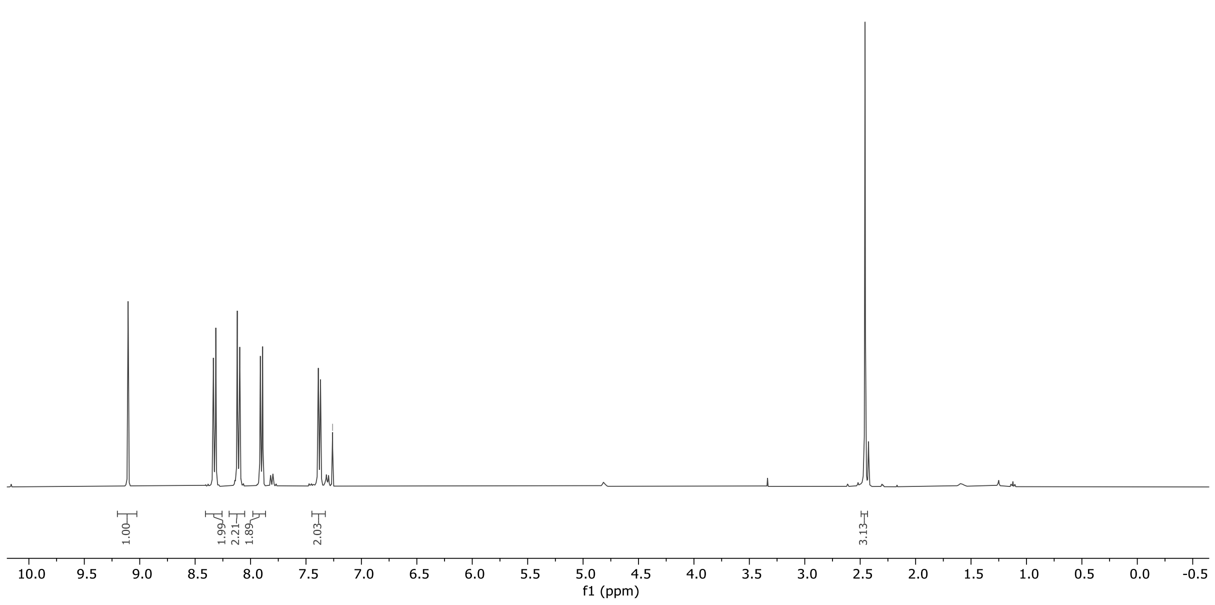

Compound **1d**
^{13}C NMR
(101 MHz, $CDCl_3$)

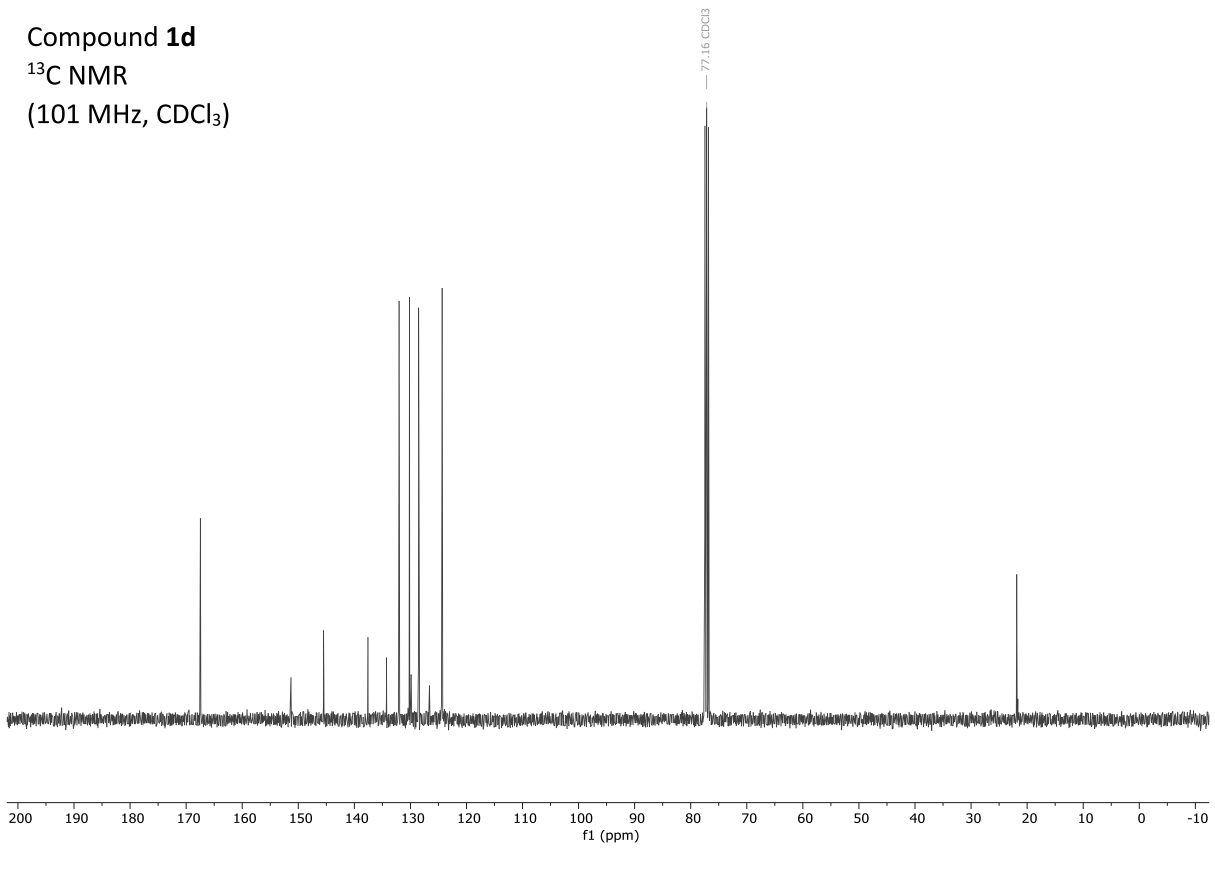

Compound **1e**
^{1}H NMR
(400 MHz, $CDCl_3$)

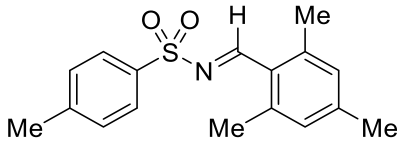

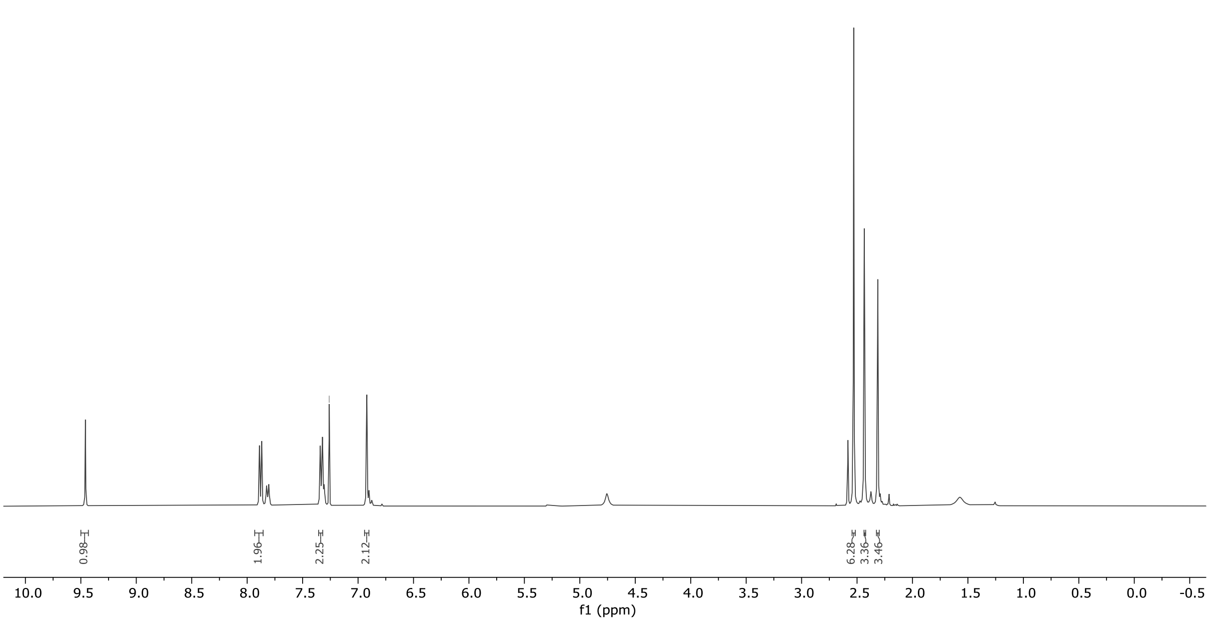

Compound **1e**
^{13}C NMR
(101 MHz, $CDCl_3$)

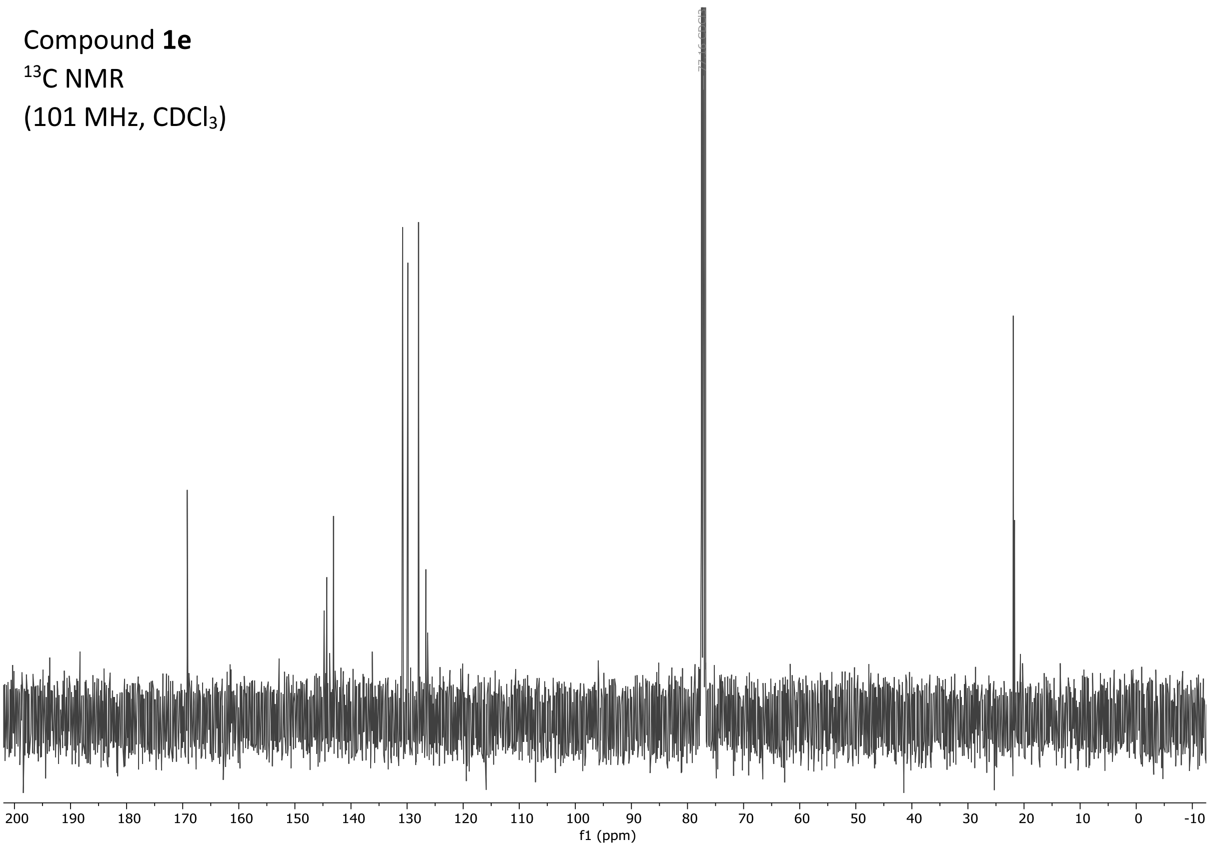

Compound **1f**
^{1}H NMR
(400 MHz, $CDCl_3$)

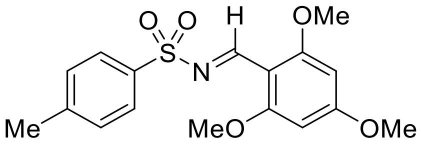

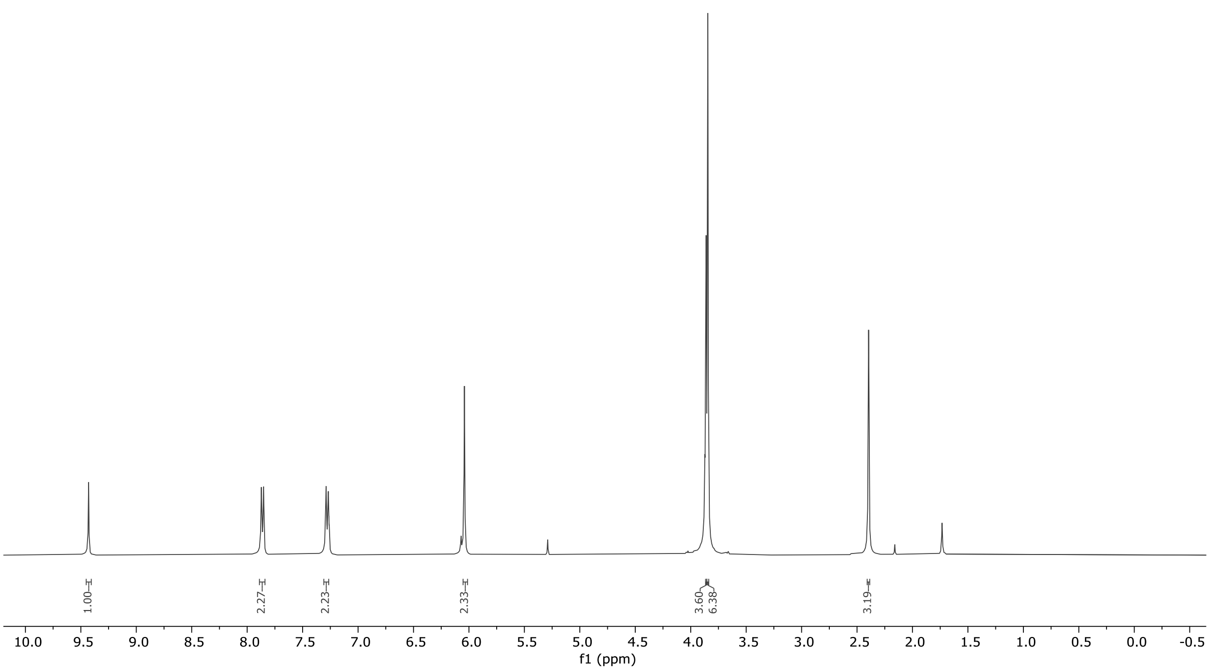

Compound **1f**
^{13}C NMR
(101 MHz, $CDCl_3$)

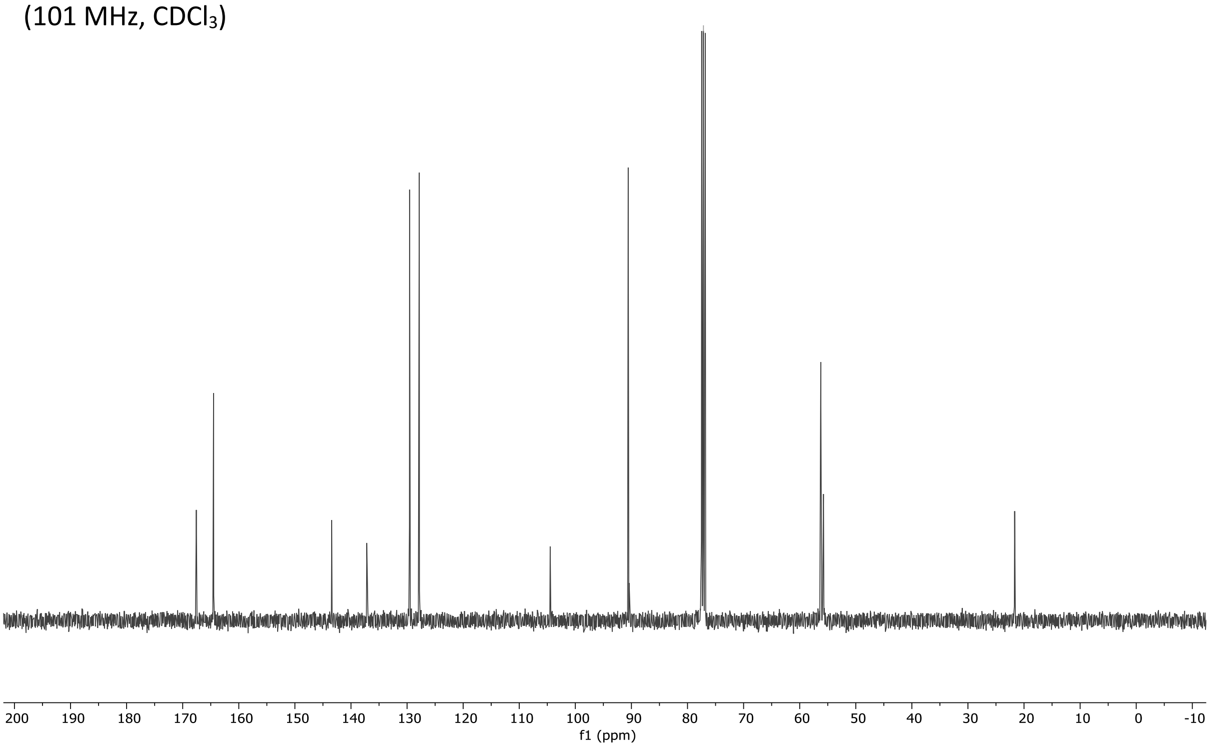

Compound **1g**
^{1}H NMR
(400 MHz, $CDCl_3$)

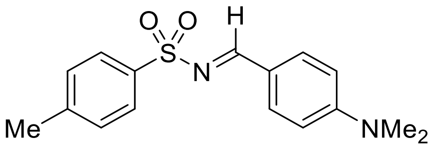

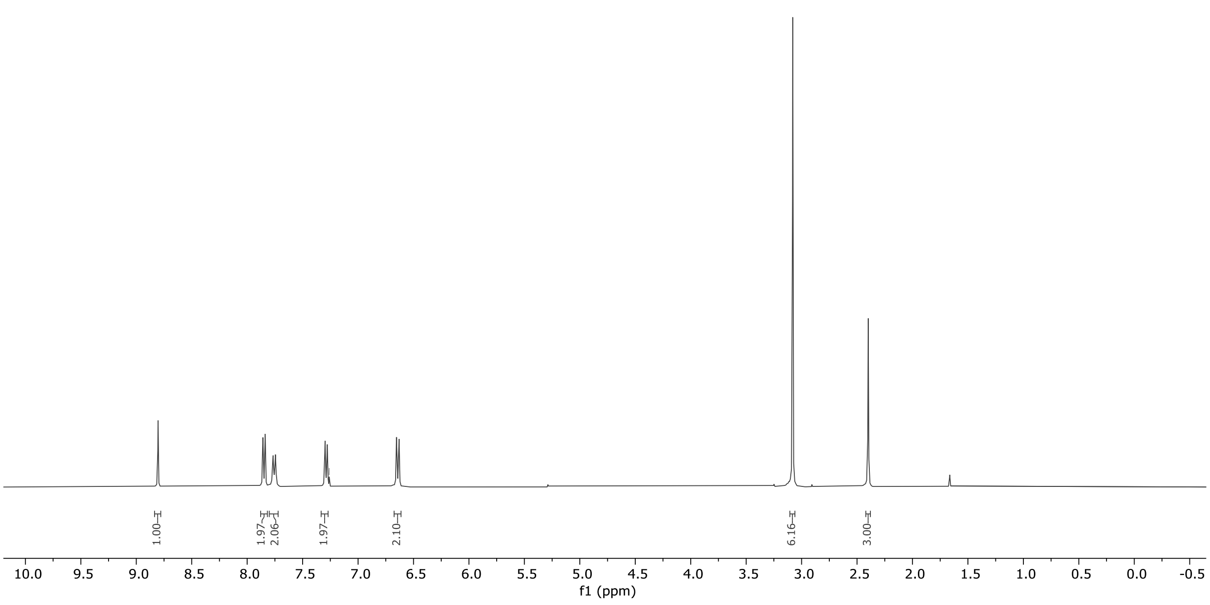

Compound **1g**
^{13}C NMR
(101 MHz, $CDCl_3$)

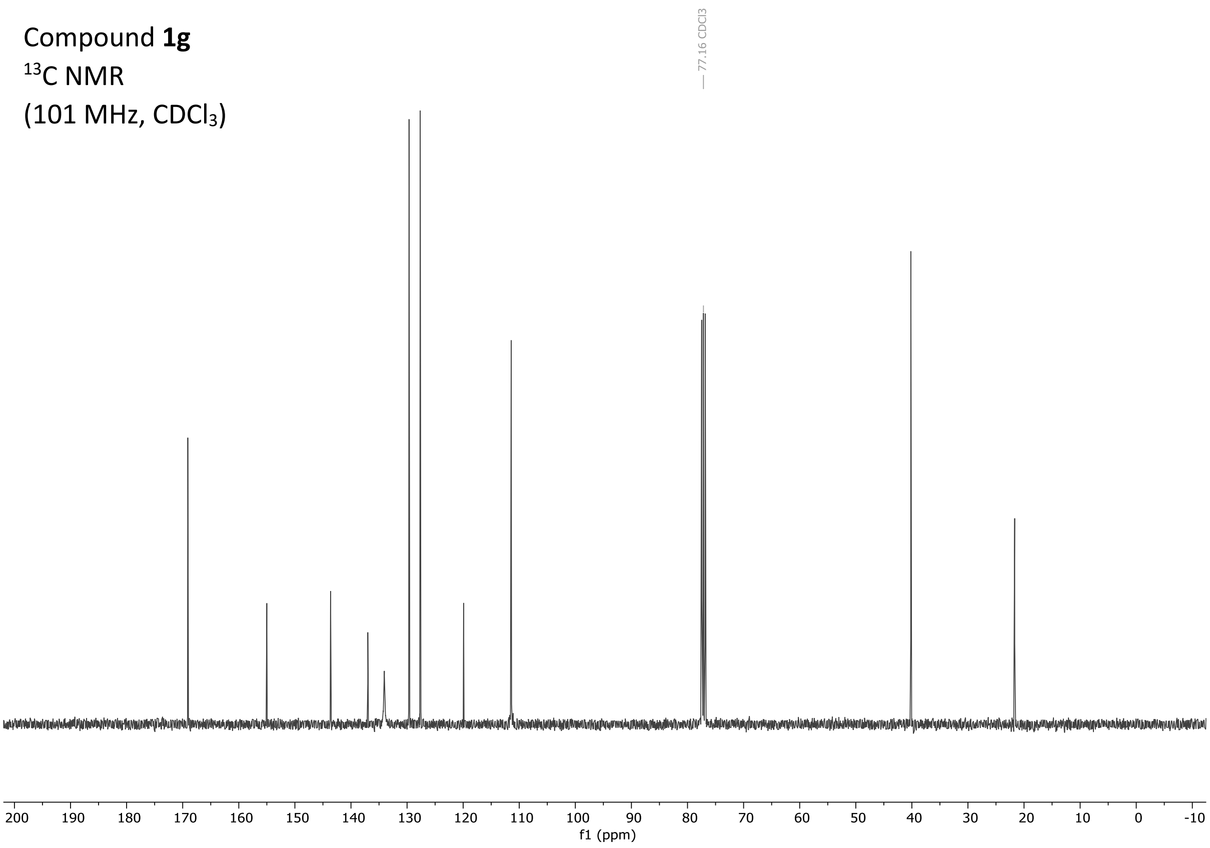

Compound **1h**
^{1}H NMR
(600 MHz, $CDCl_3$)

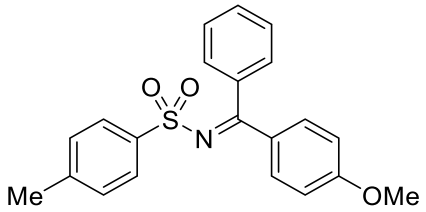

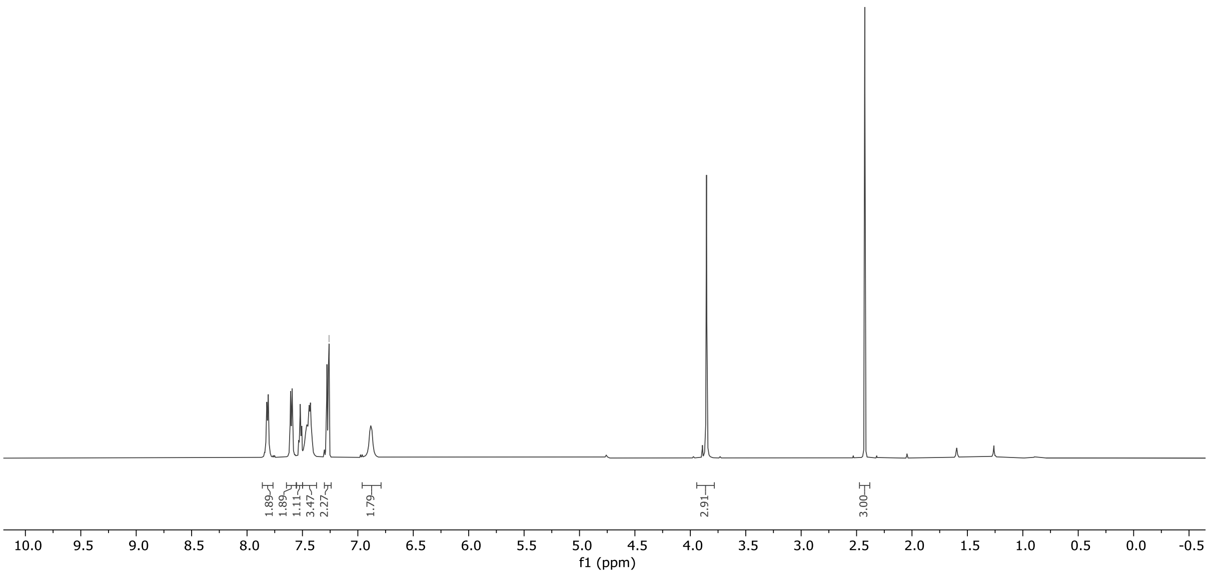

Compound **1h**
^{13}C NMR
(151 MHz, $CDCl_3$)

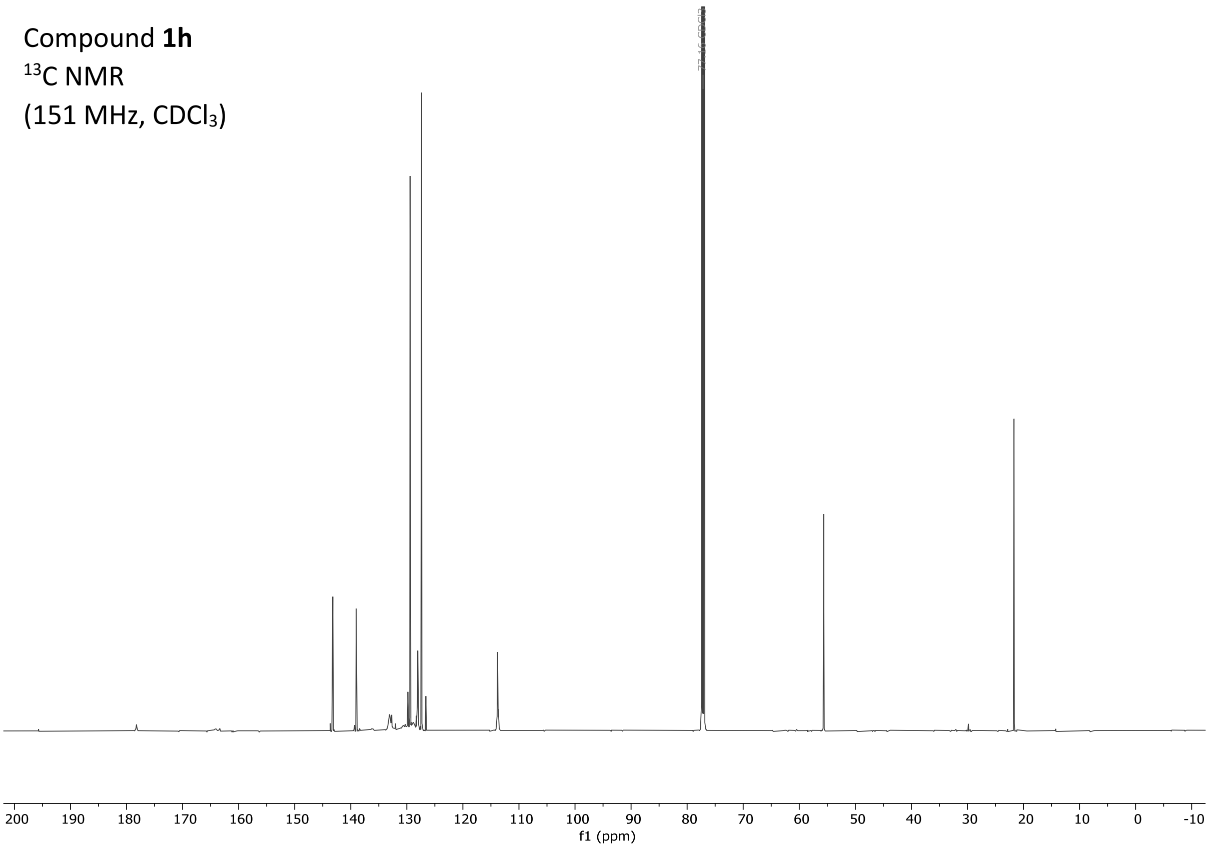

Compound **1ab**
^{1}H NMR
(400 MHz, $CDCl_3$)

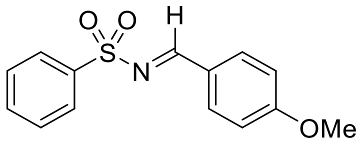

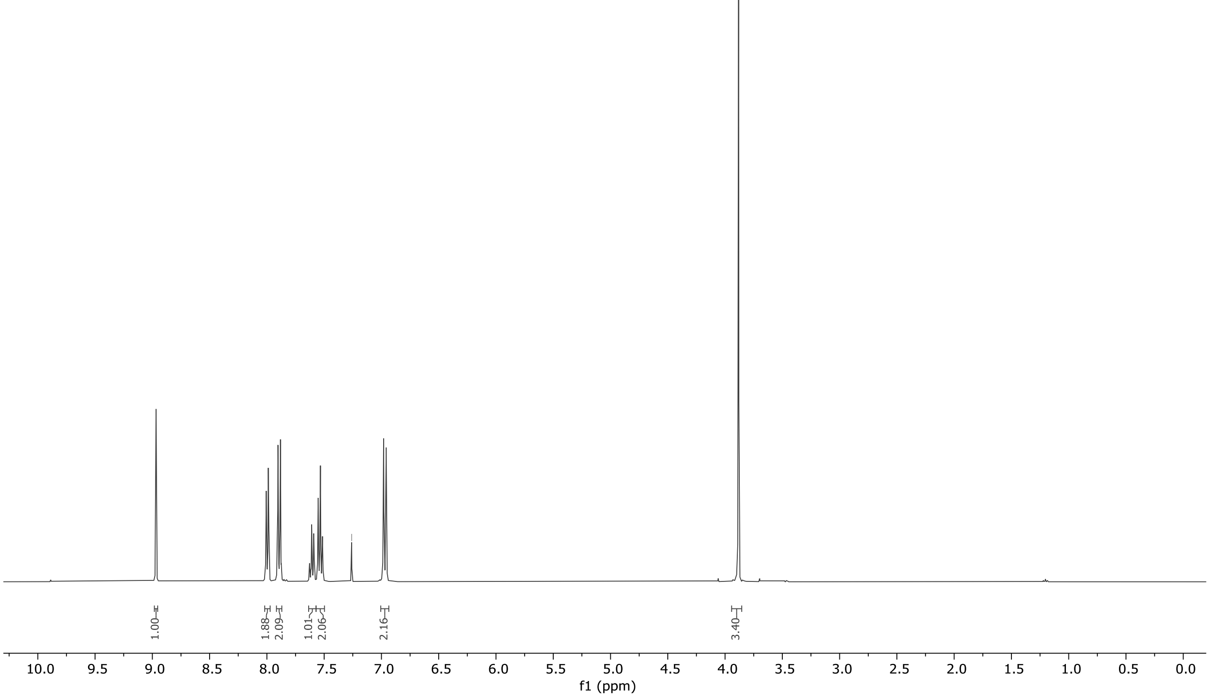

Compound **1ab**
^{13}C NMR
(101 MHz, $CDCl_3$)

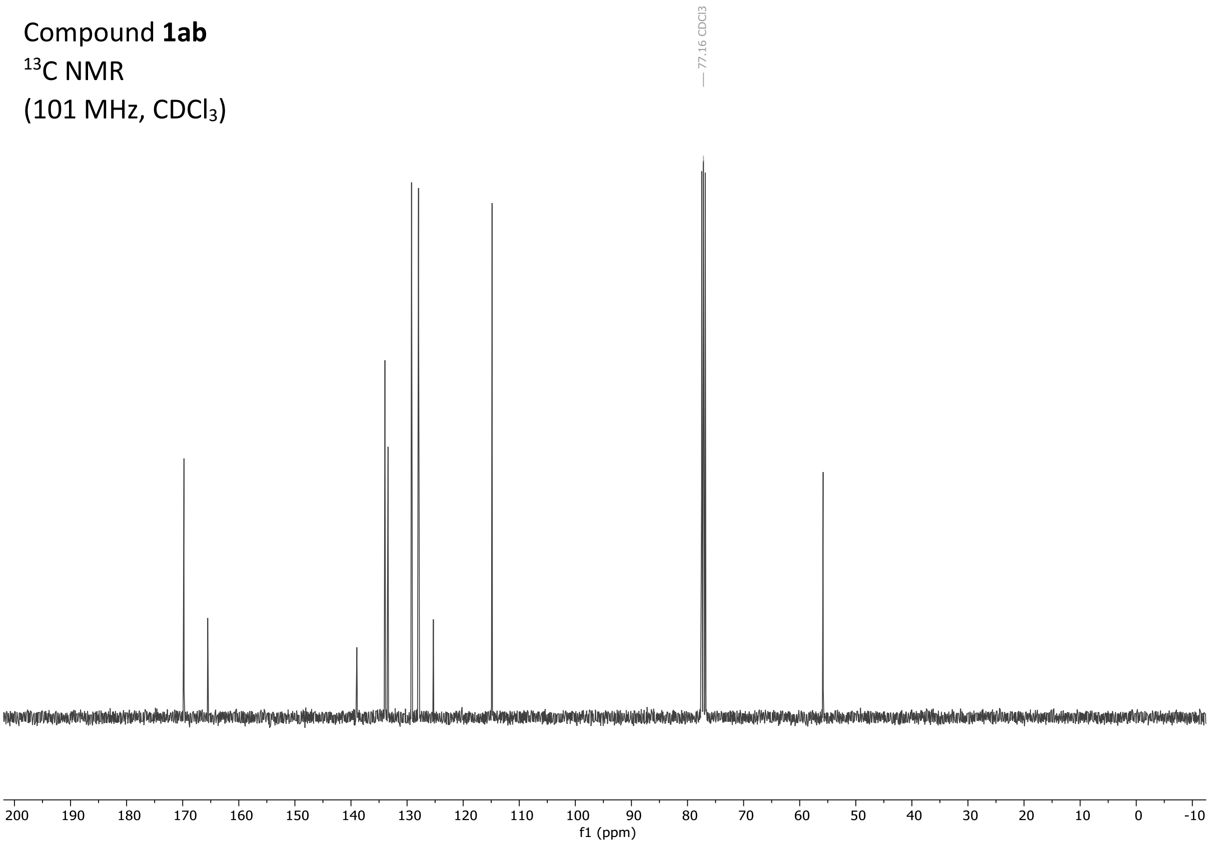

Compound **1ac**
^{1}H NMR
(400 MHz, $CDCl_3$)

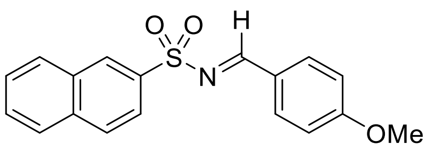

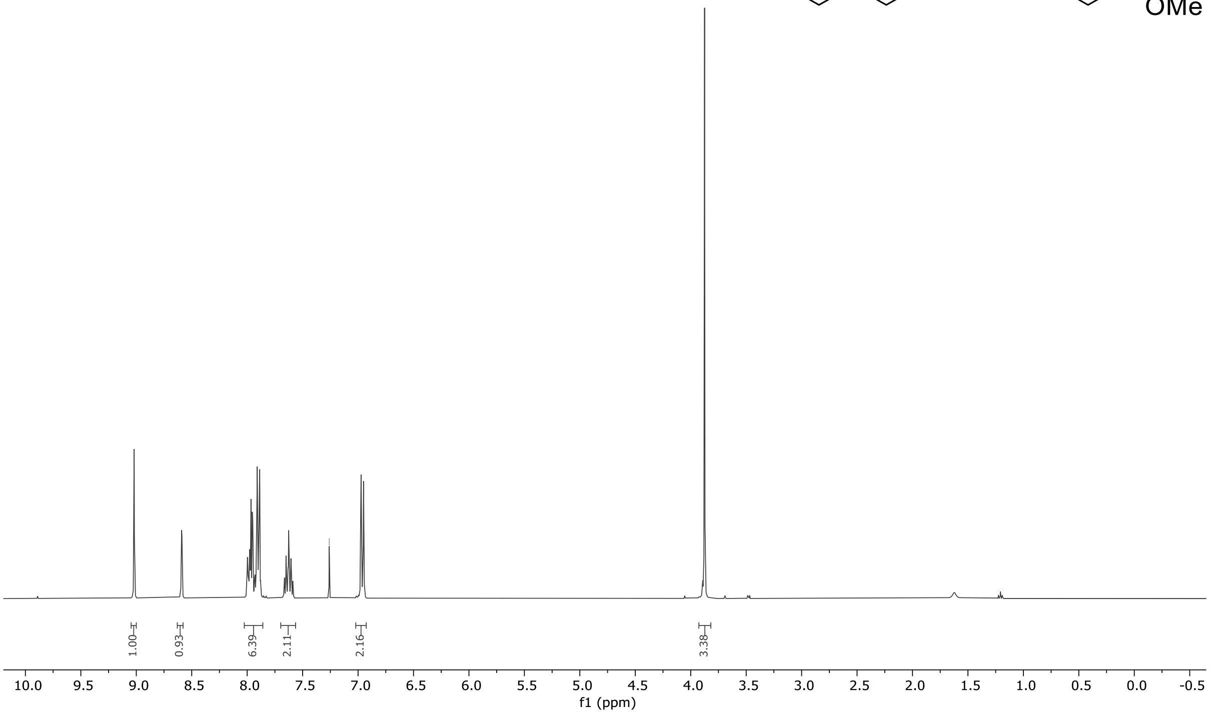

Compound **1ac**
^{13}C NMR
(101 MHz, $CDCl_3$)

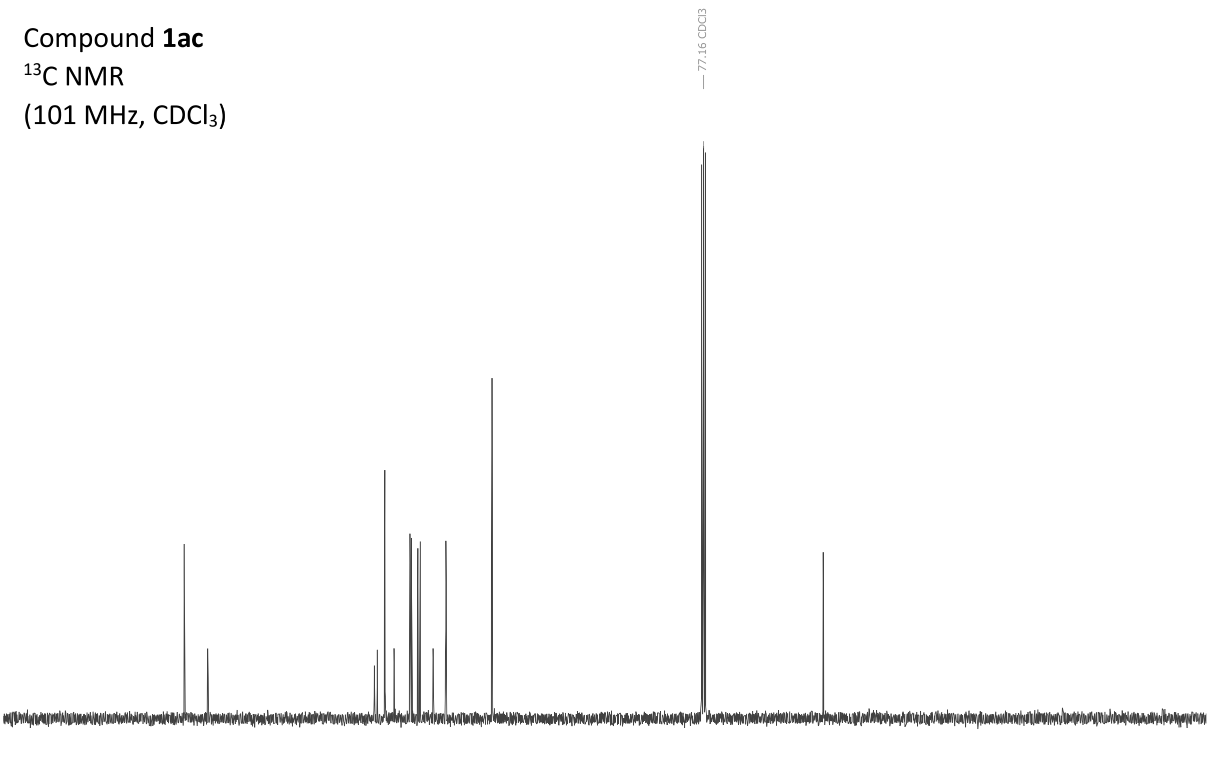

Compound **1ad**
^{1}H NMR
(400 MHz, $CDCl_3$)

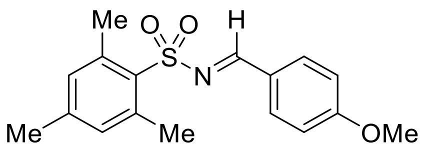

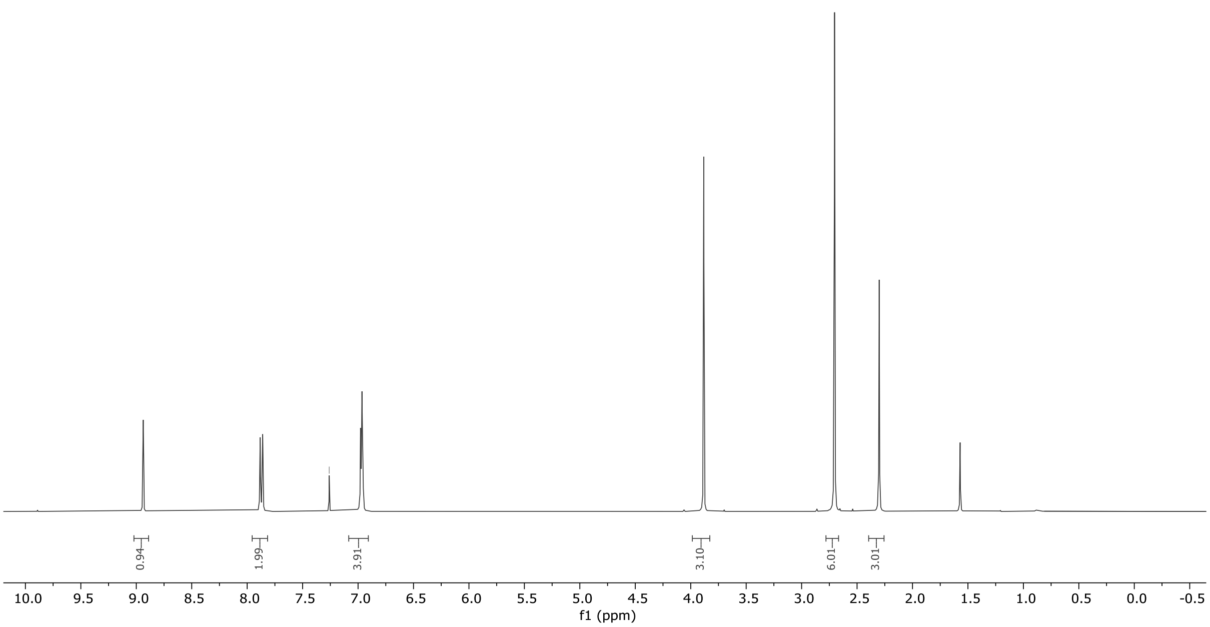

Compound **1ad**
^{13}C NMR
(101 MHz, $CDCl_3$)

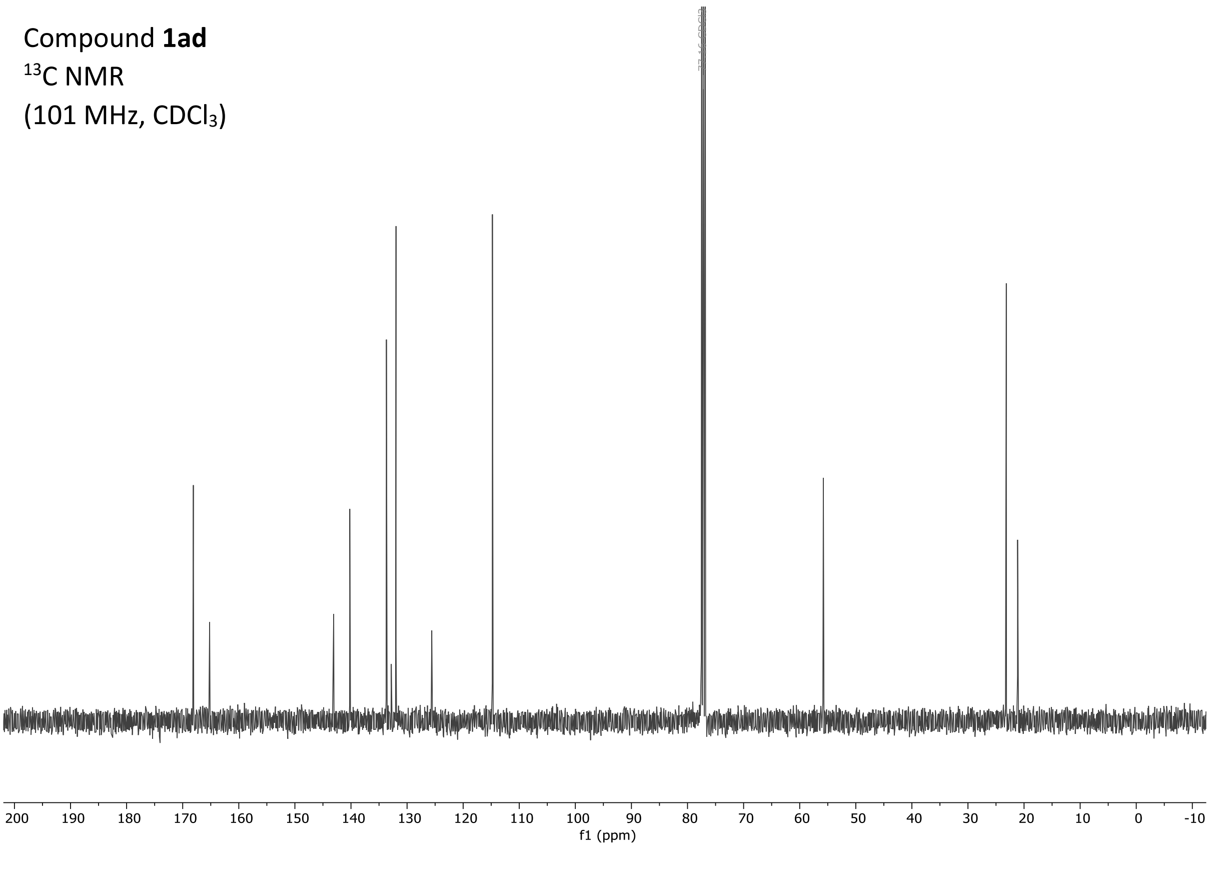

Compound **1ae**
^{1}H NMR
(400 MHz, $CDCl_3$)

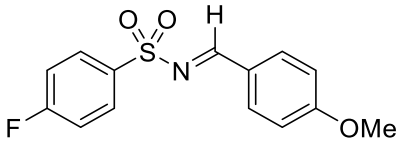

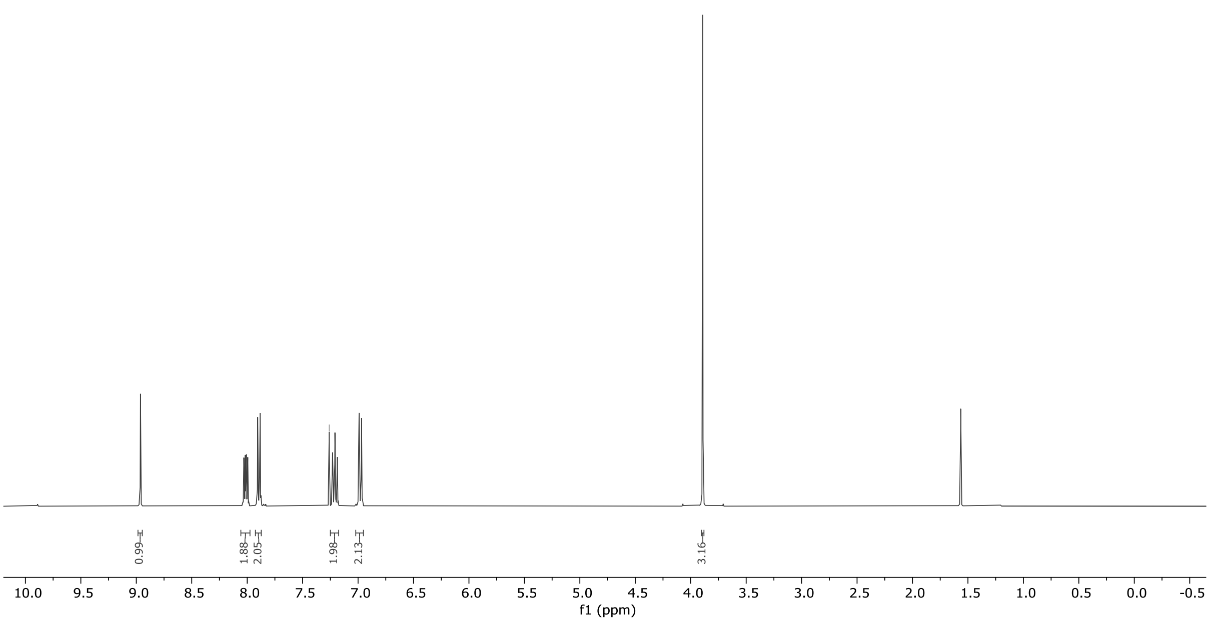

Compound **1ae**
^{13}C NMR
(101 MHz, $CDCl_3$)

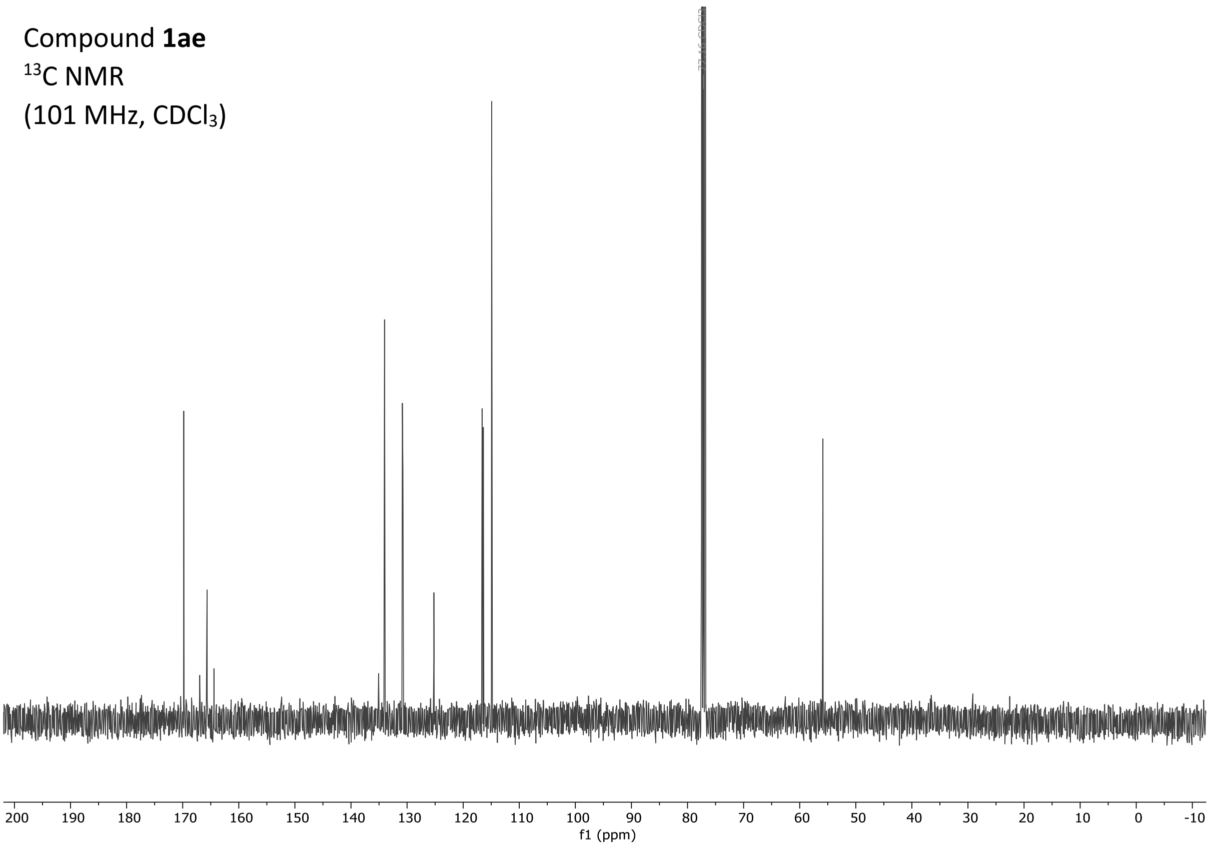

Compound **1ae**
^{19}F NMR
(377 MHz, $CDCl_3$)

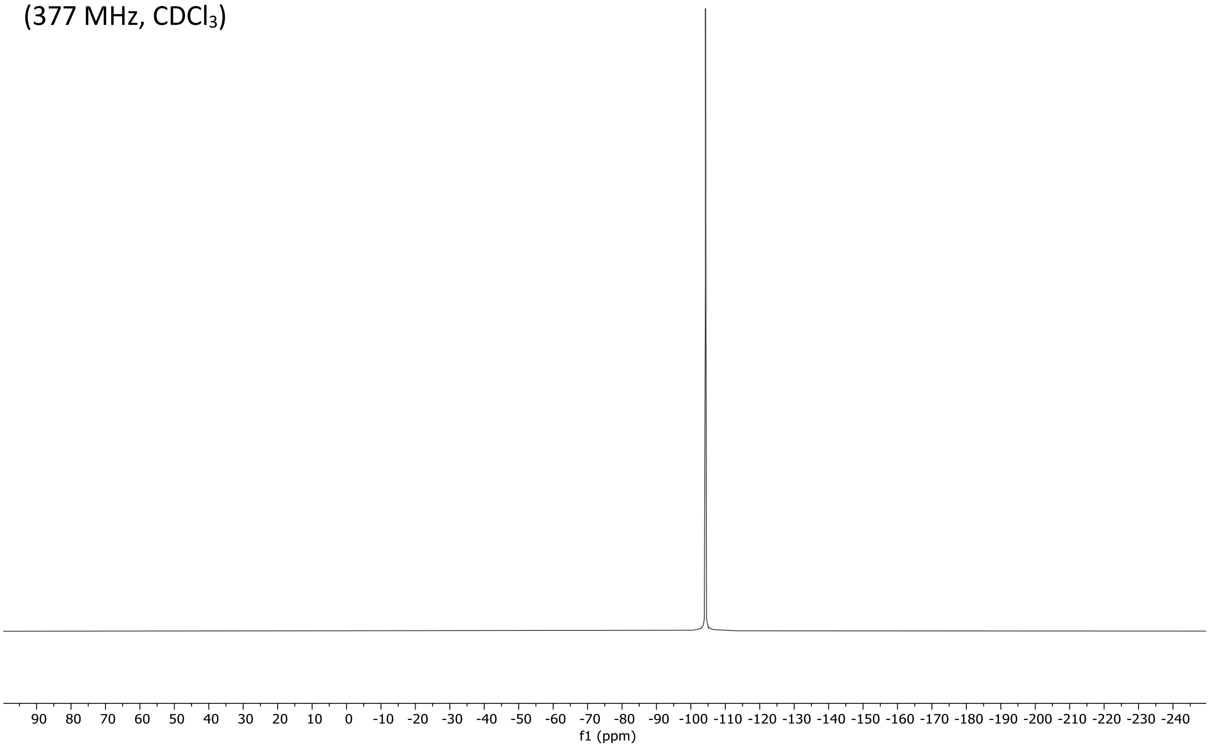

Compound **1af**
^{1}H NMR
(400 MHz, $CDCl_3$)

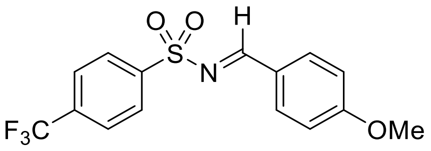

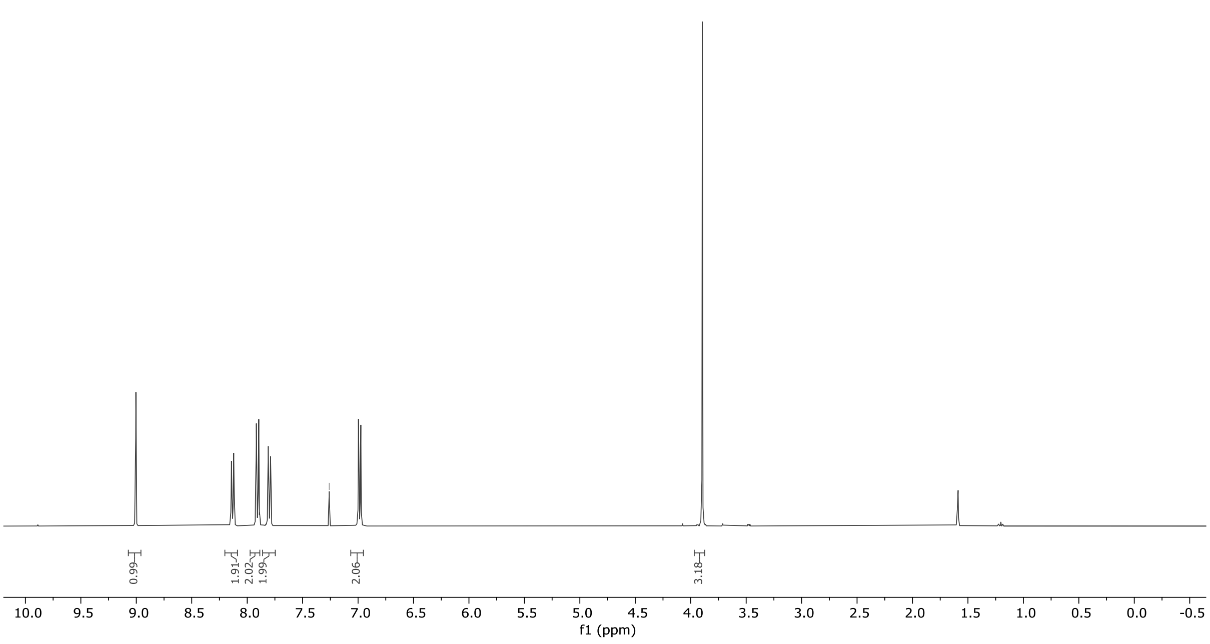

Compound **1af**
^{13}C NMR
(101 MHz, $CDCl_3$)

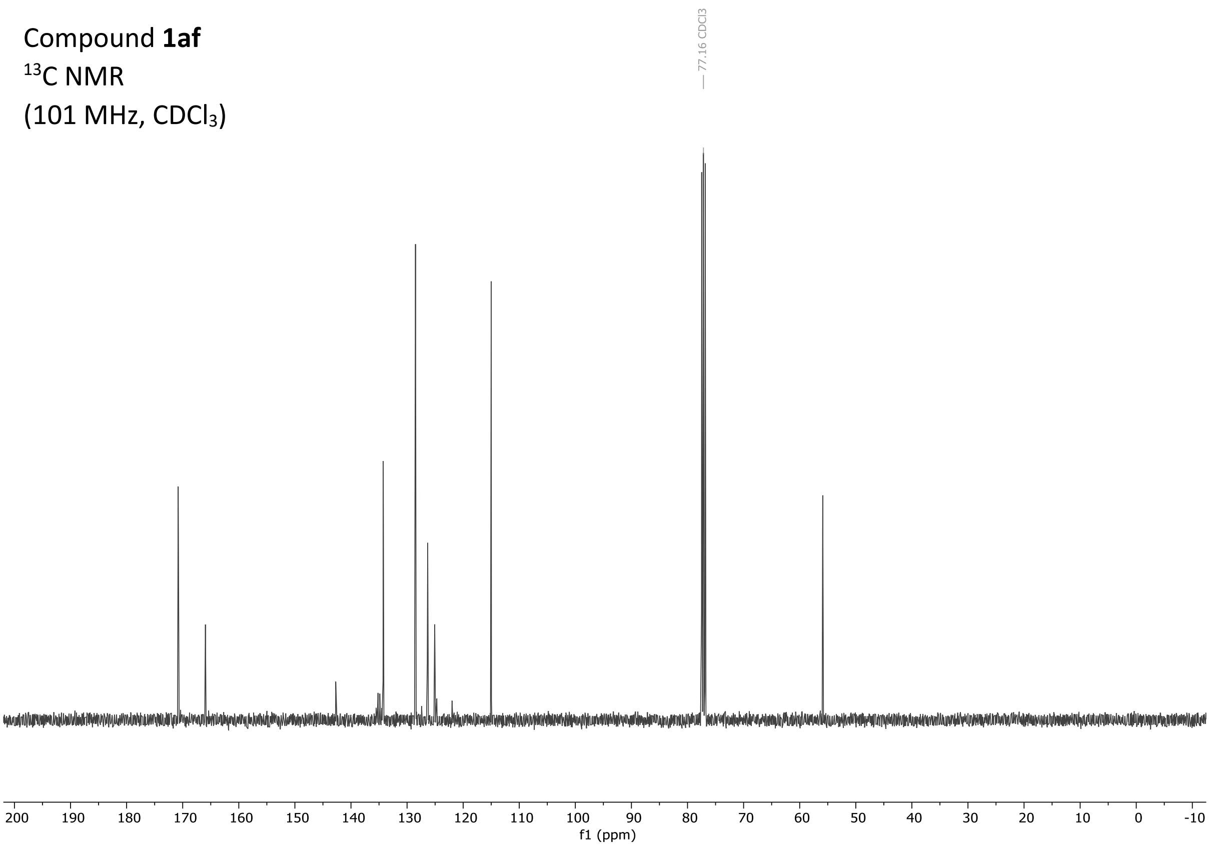

Compound **1af**
^{19}F NMR
(377 MHz, $CDCl_3$)

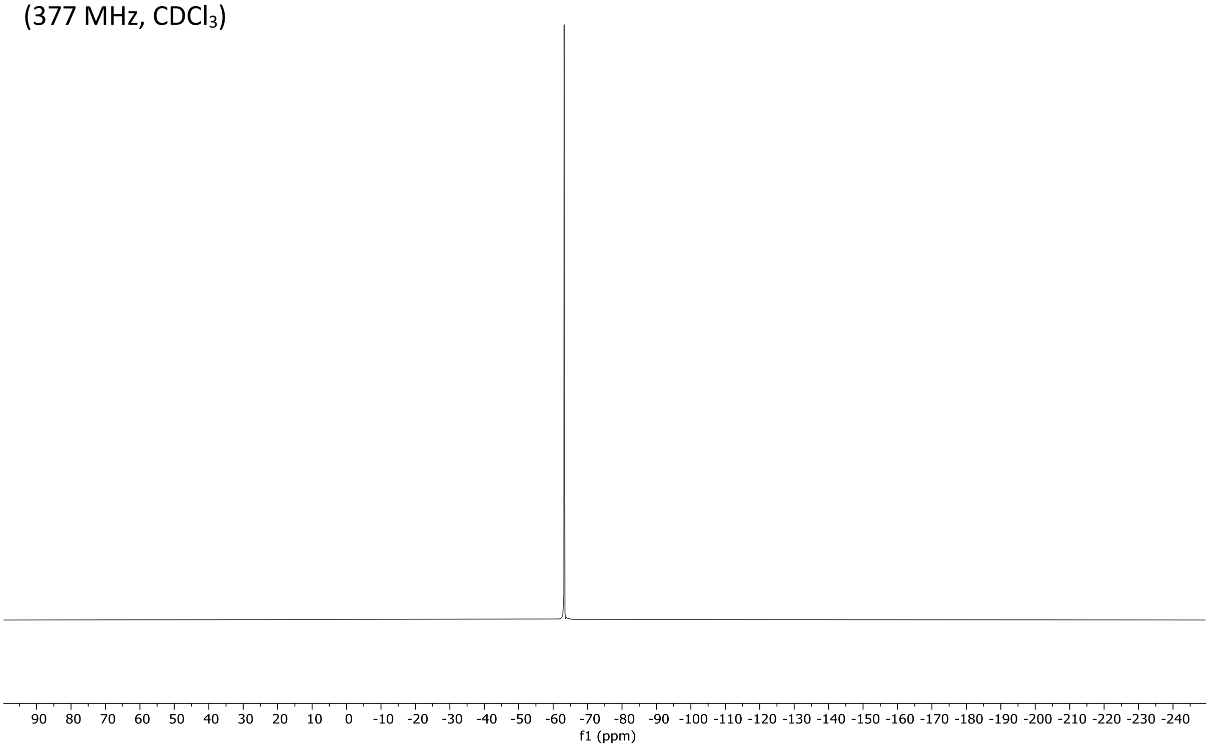

Compound **1ag**
^{1}H NMR
(400 MHz, $CDCl_3$)

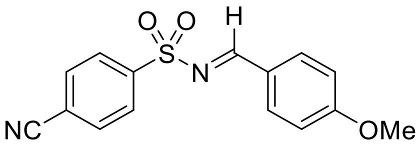

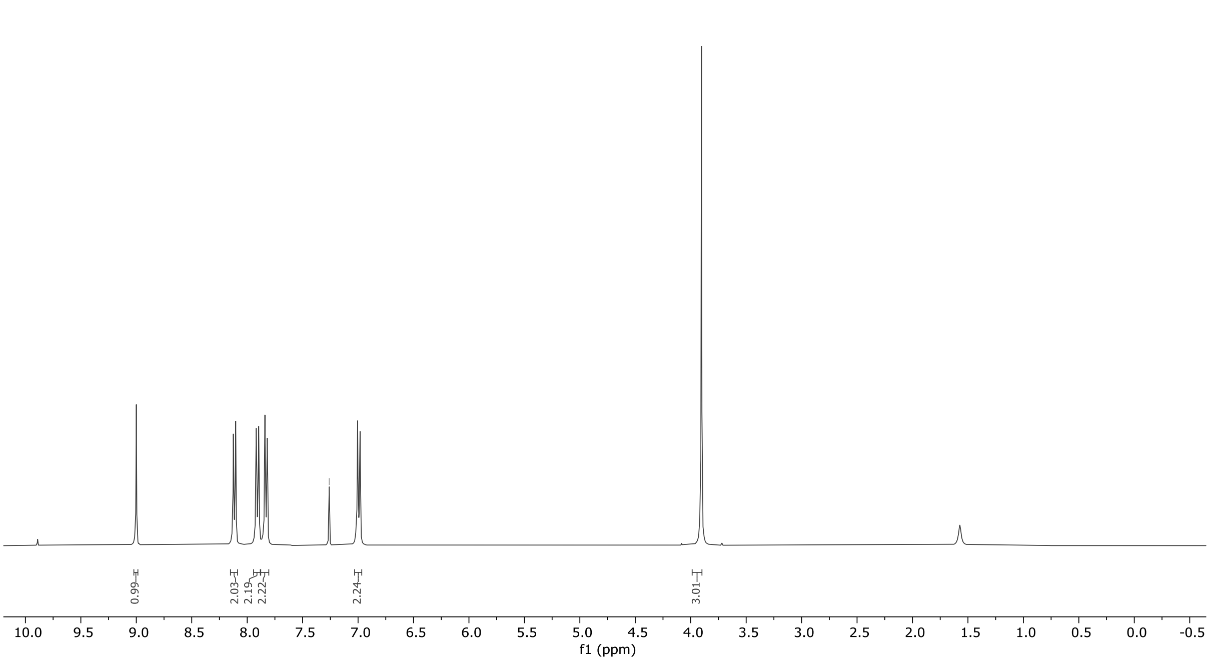

Compound **1ag**
^{13}C NMR
(101 MHz, $CDCl_3$)

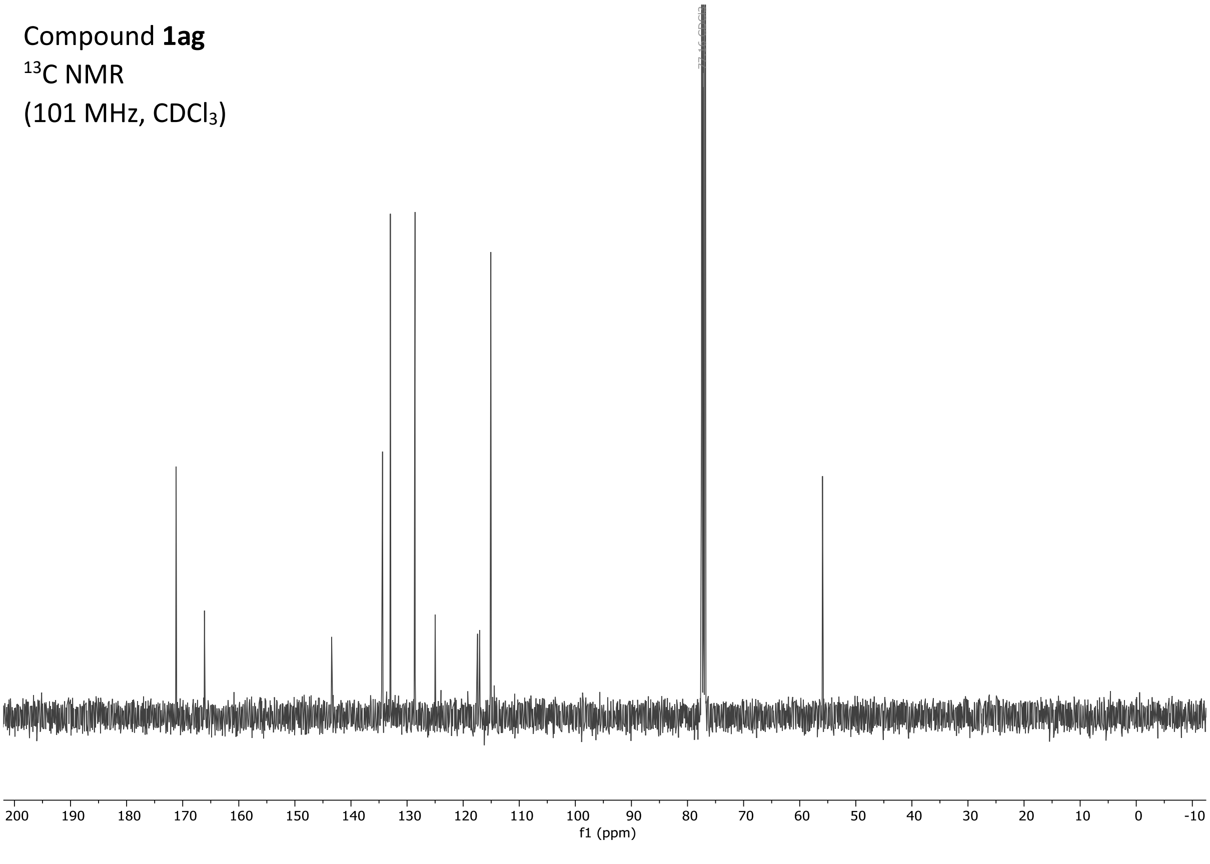

Compound **1ah**
^{1}H NMR
(400 MHz, $CDCl_3$)

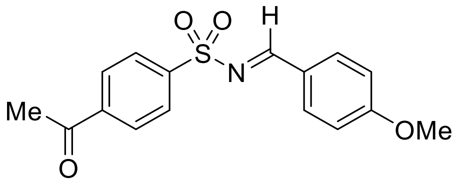

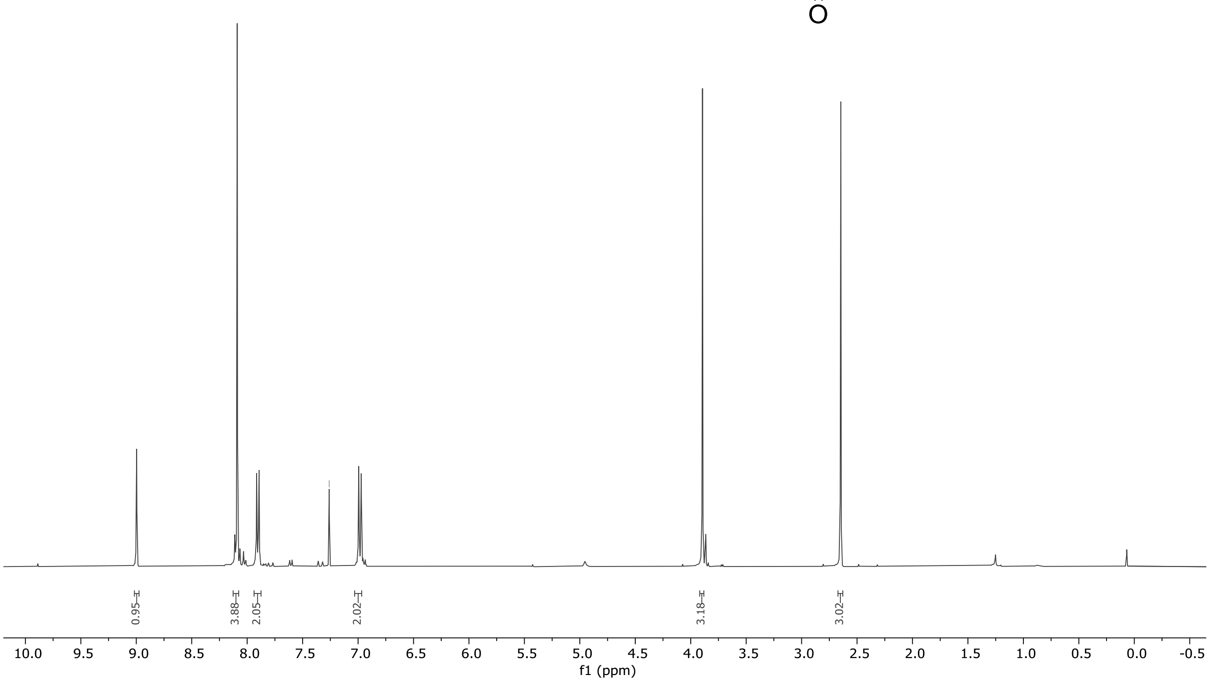

Compound **1ah**
^{13}C NMR
(101 MHz, $CDCl_3$)

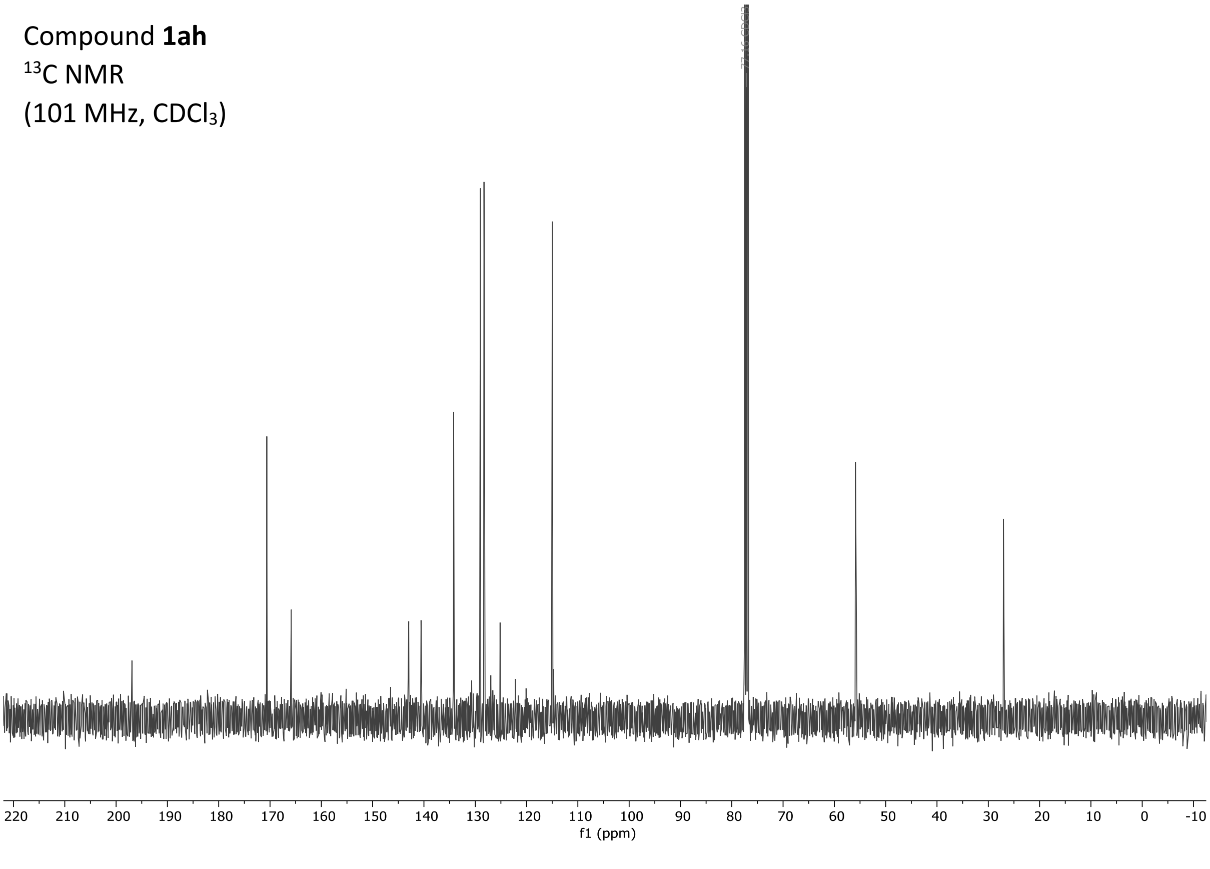

Compound **1ai**
^{1}H NMR
(400 MHz, $CDCl_3$)

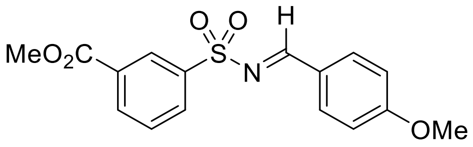

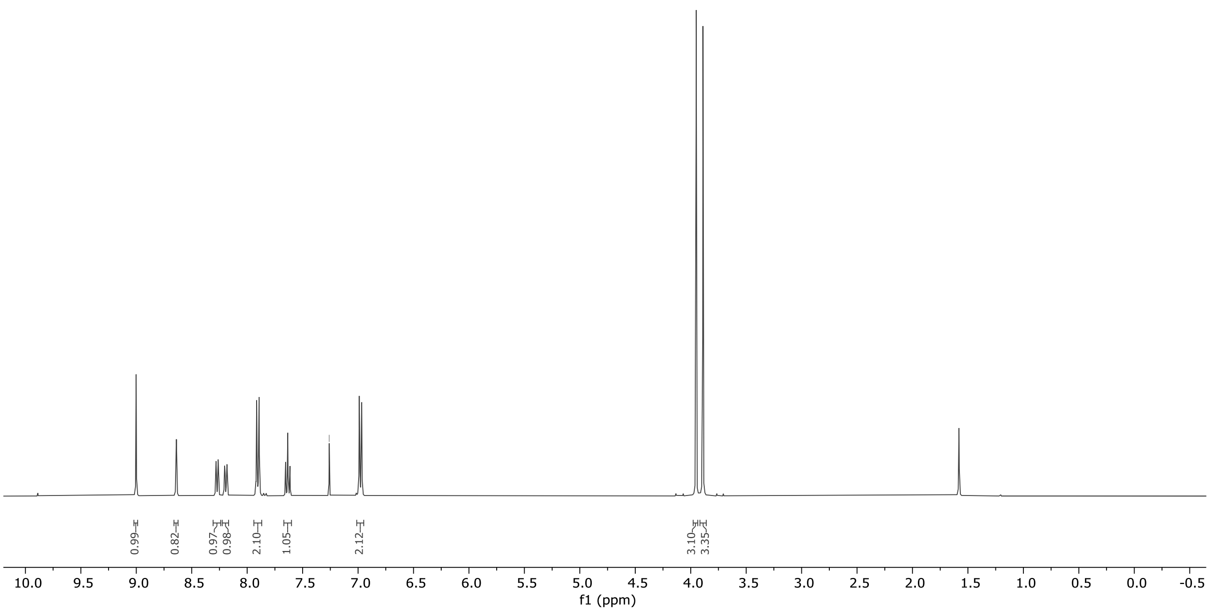

Compound **1ai**
^{13}C NMR
(101 MHz, $CDCl_3$)

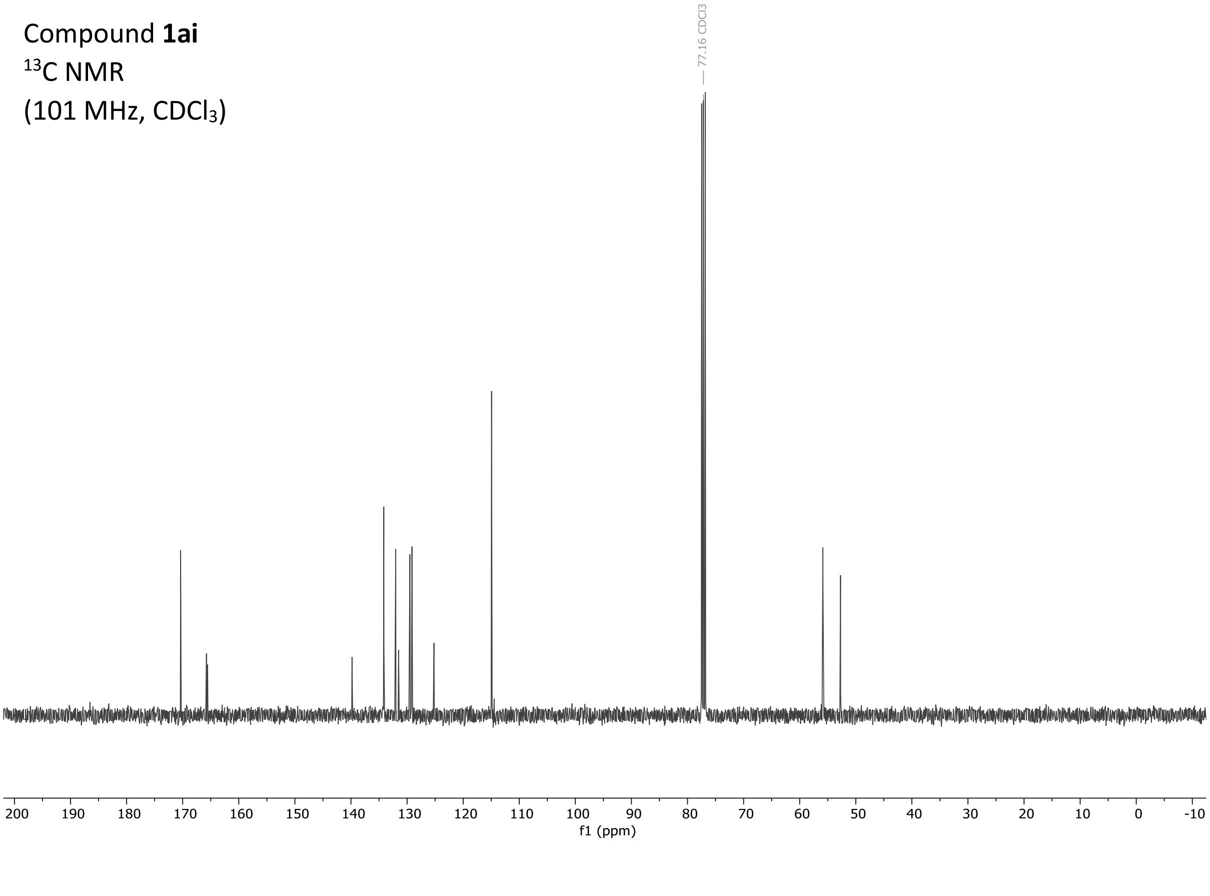

Compound **1aj**
^{1}H NMR
(400 MHz, $CDCl_3$)

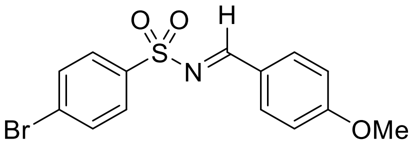

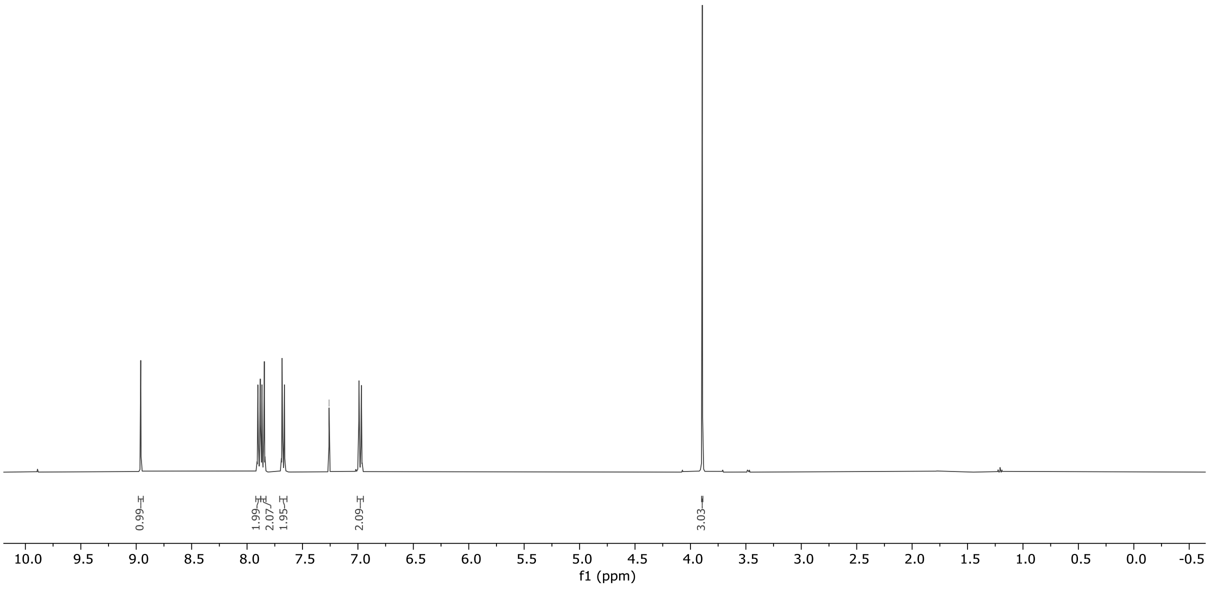

Compound **1aj**
^{13}C NMR
(101 MHz, $CDCl_3$)

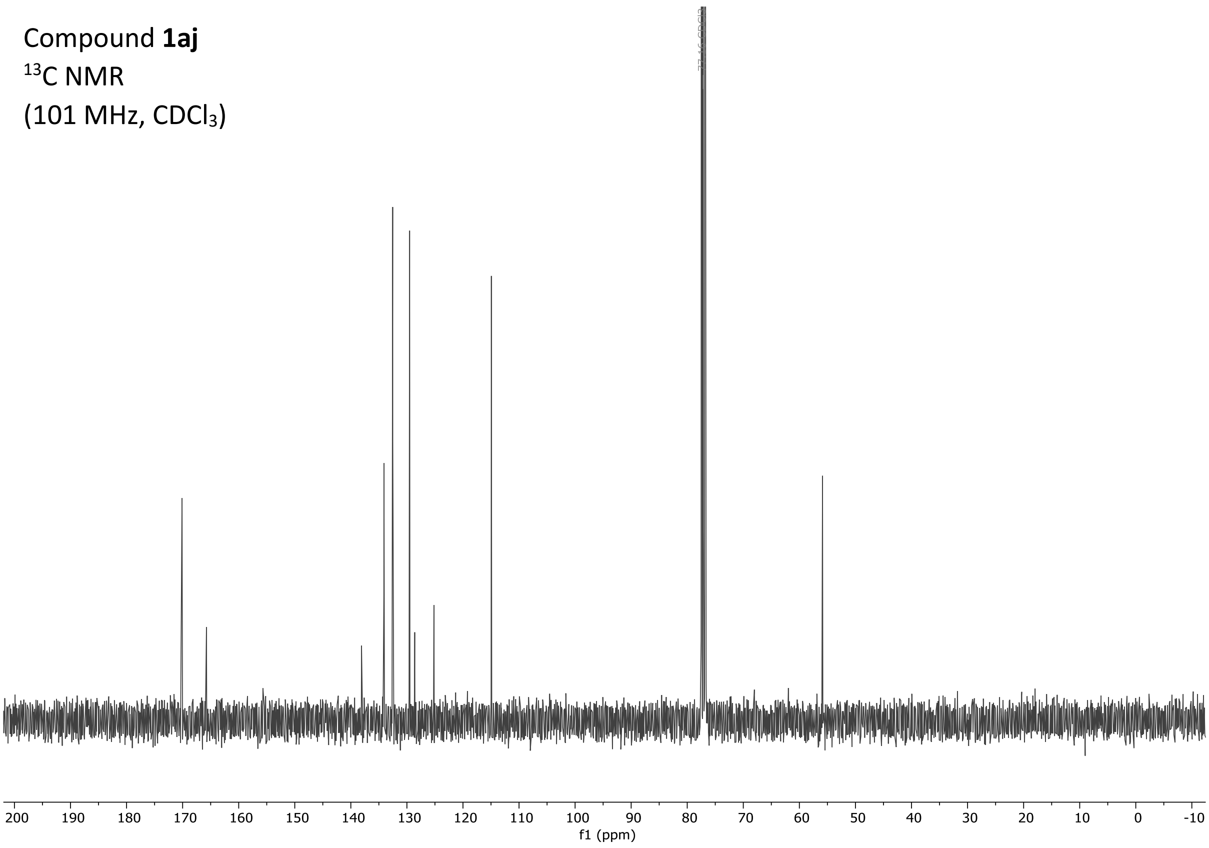

Compound **1ak**
^{1}H NMR
(400 MHz, $CDCl_3$)

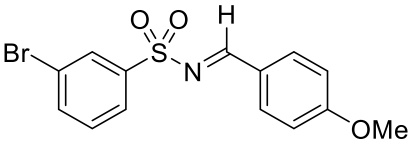

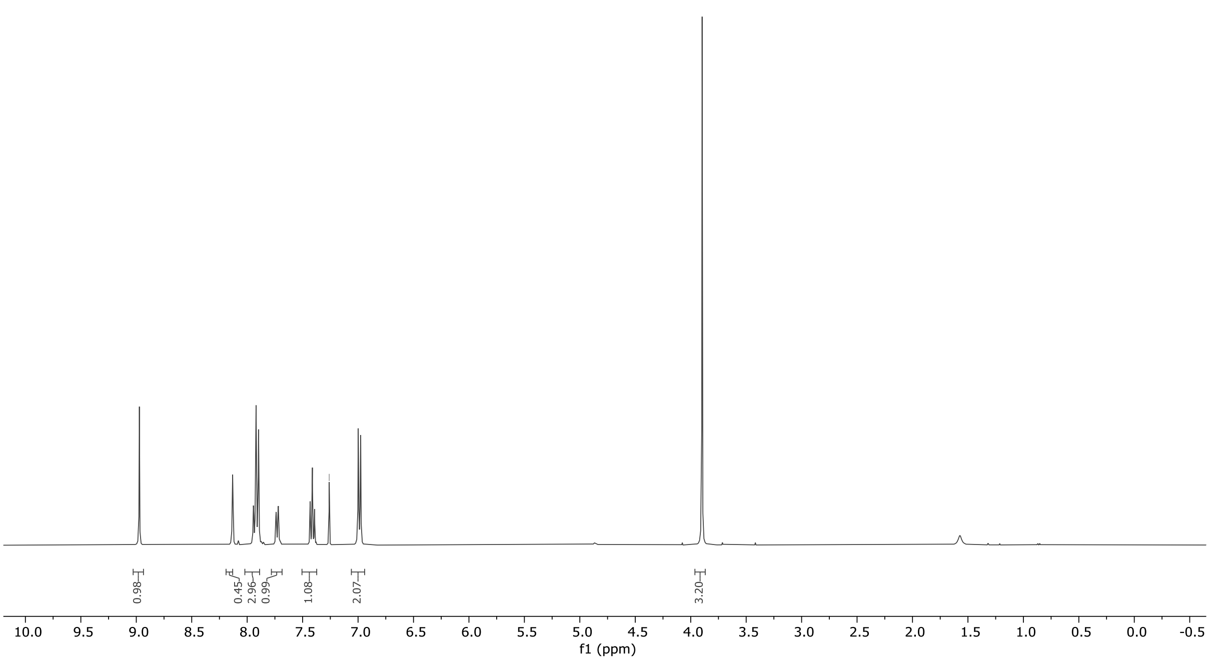

Compound **1ak**
^{13}C NMR
(101 MHz, $CDCl_3$)

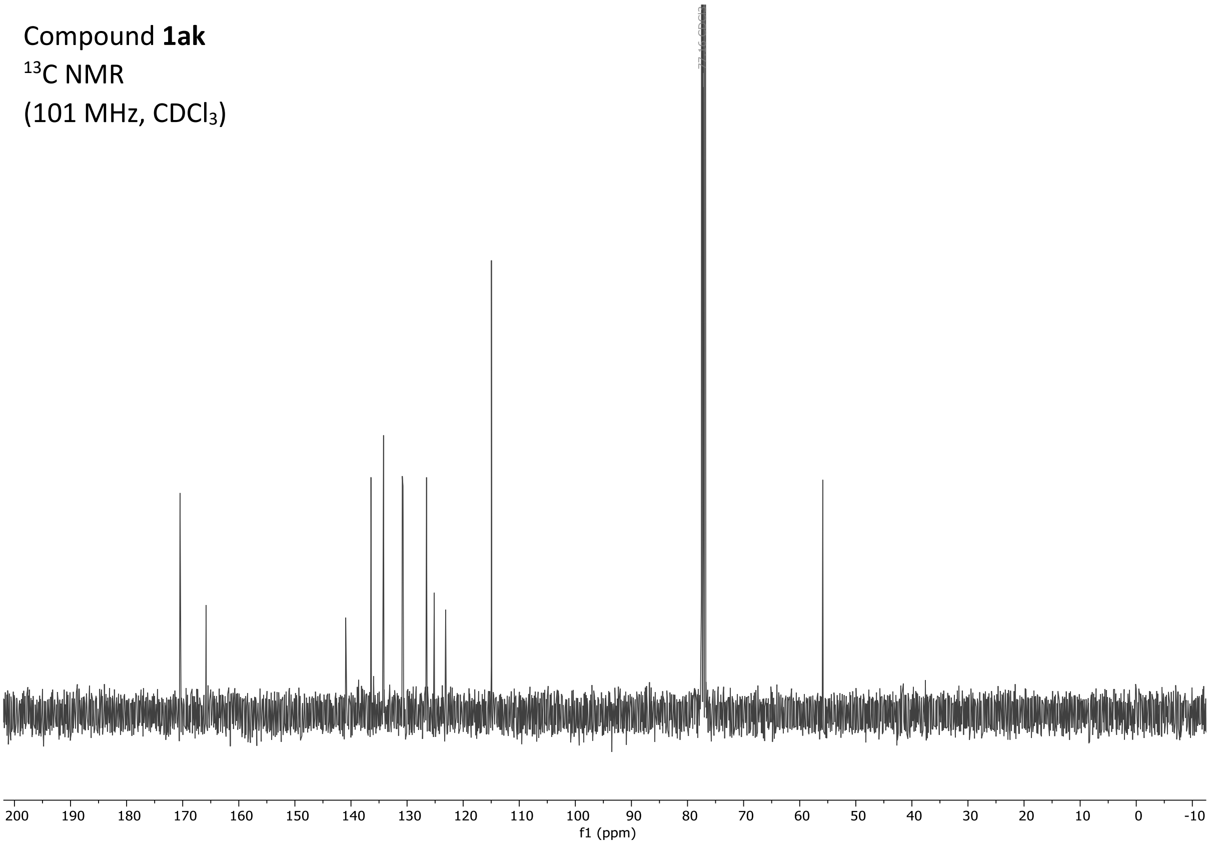

Compound **1al**
^{1}H NMR
(400 MHz, $CDCl_3$)

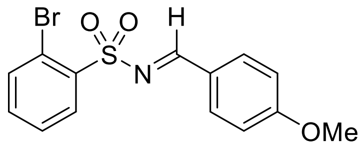

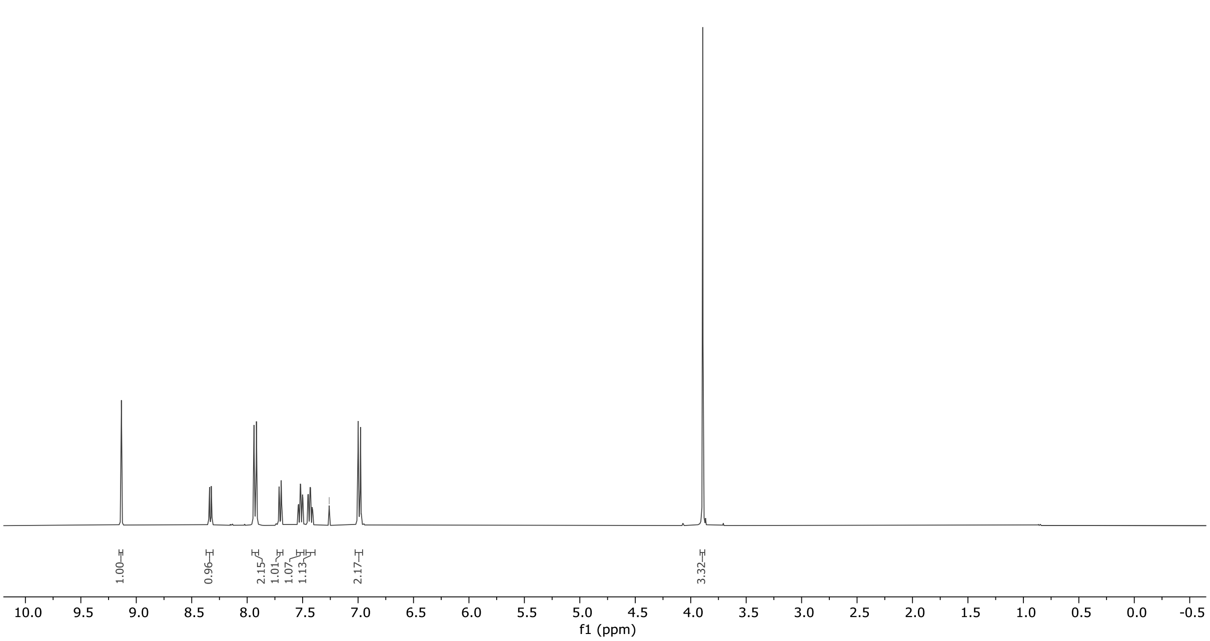

Compound **1al**
^{13}C NMR
(101 MHz, $CDCl_3$)

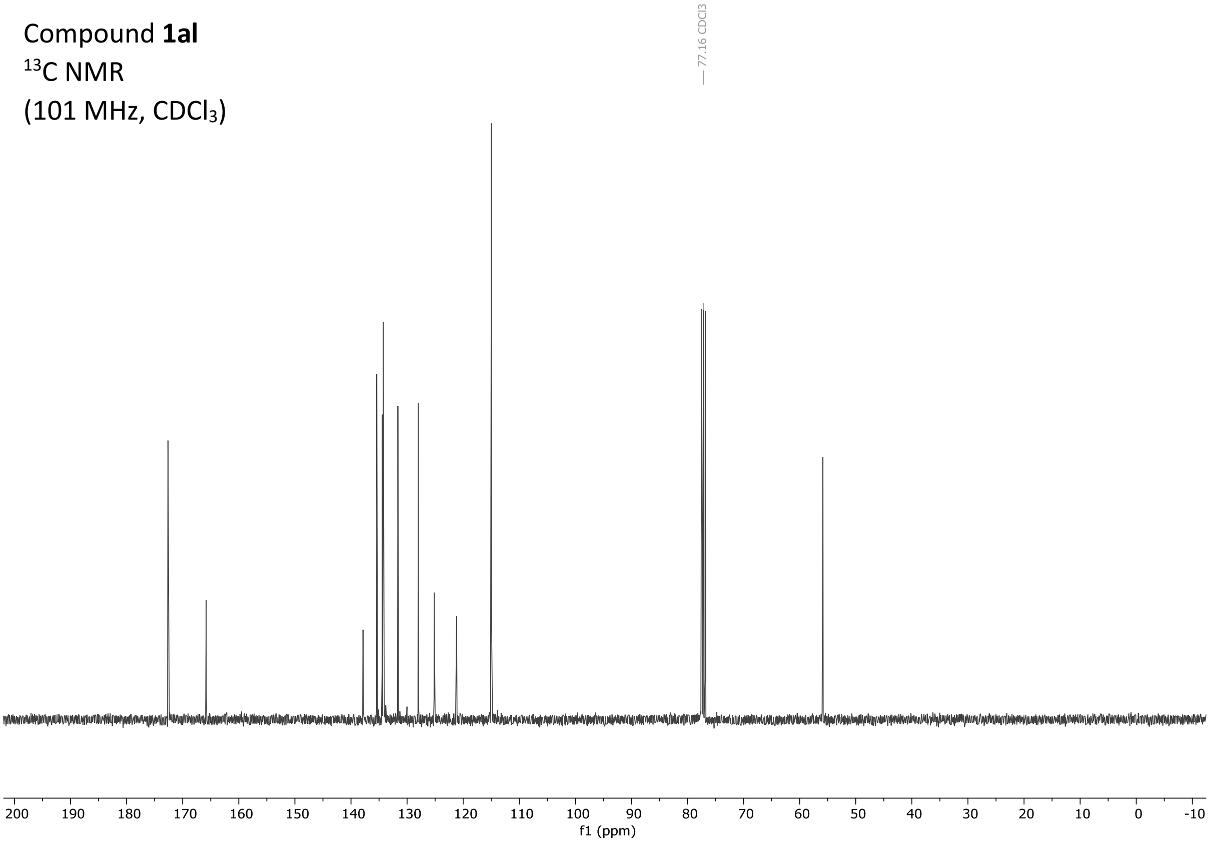

Compound **1am**
^{1}H NMR
(400 MHz, $CDCl_3$)

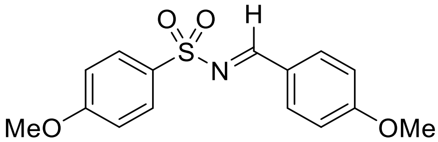

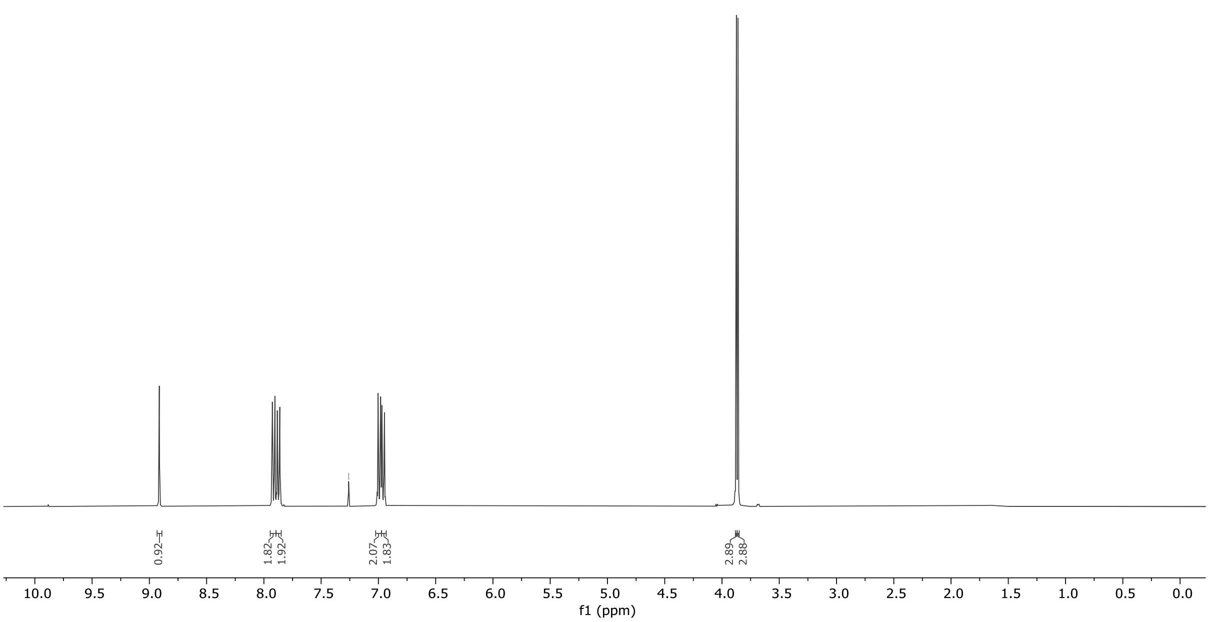

Compound **1am**
^{13}C NMR
(101 MHz, $CDCl_3$)

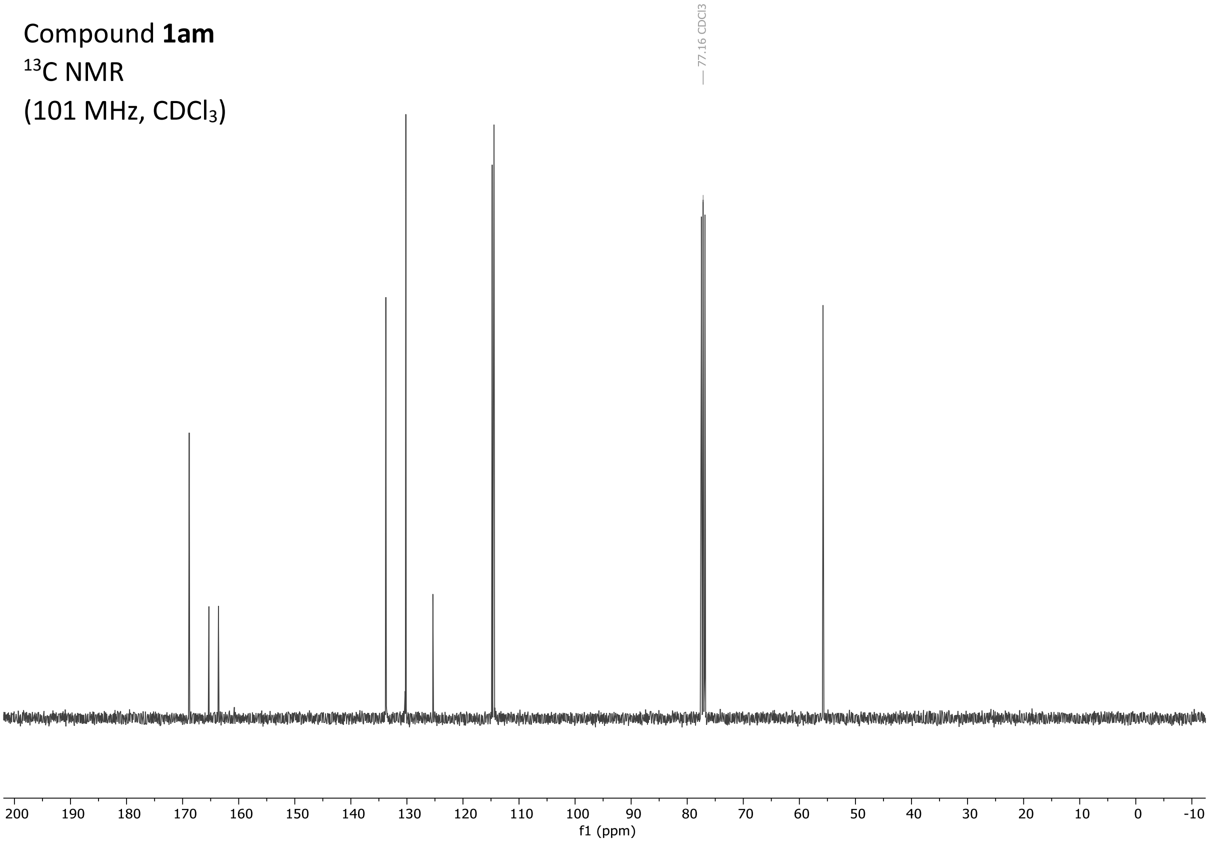

Compound **1an**
^{1}H NMR
(400 MHz, $CDCl_3$)

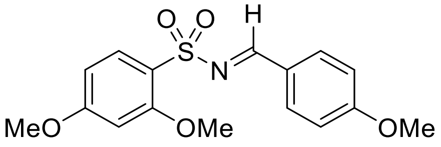

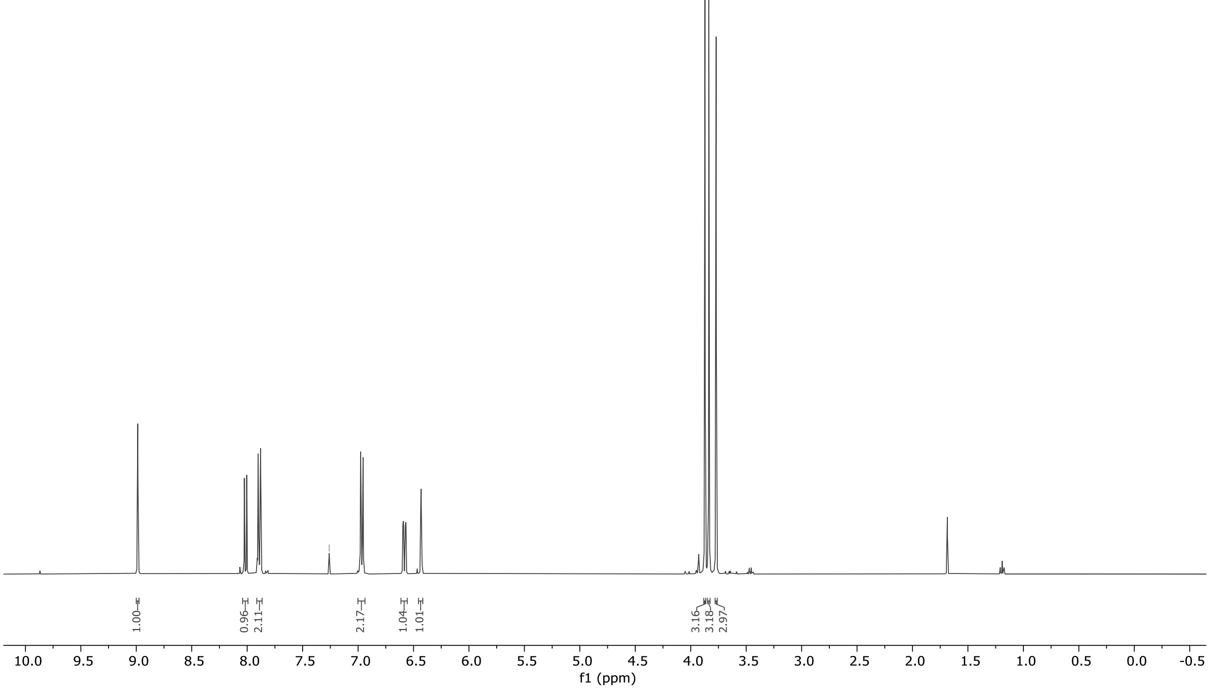

Compound **1an**
^{13}C NMR
(101 MHz, $CDCl_3$)

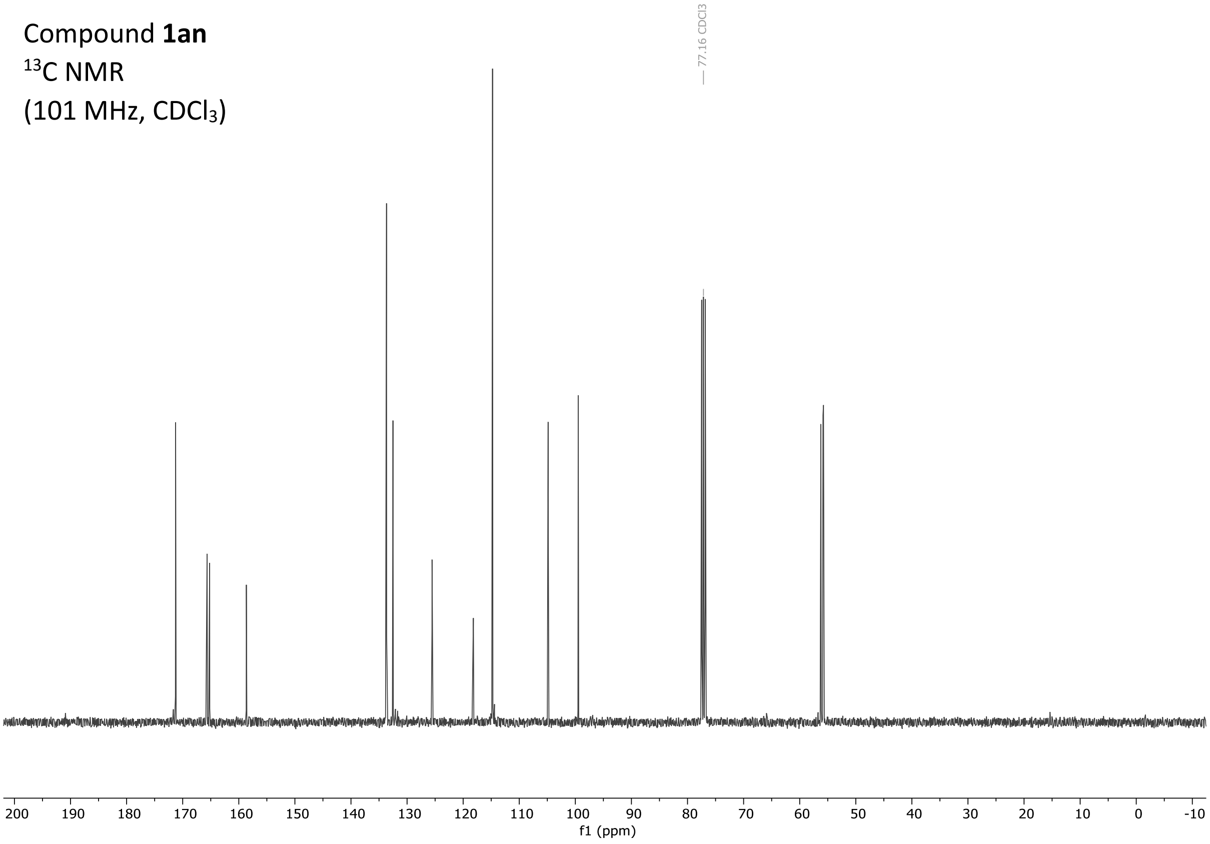

Compound **1ao**
^{1}H NMR
(400 MHz, $CDCl_3$)

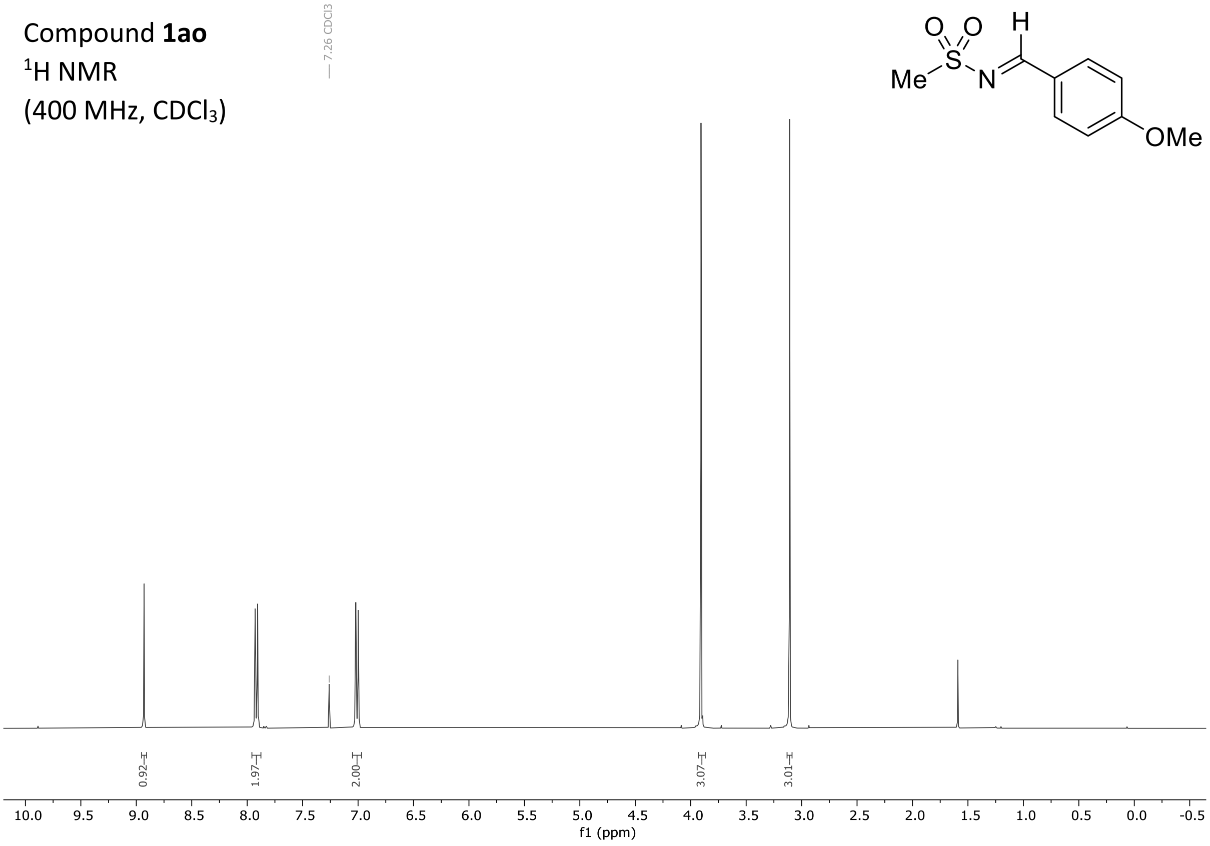

Compound **1ao**
^{13}C NMR
(101 MHz, $CDCl_3$)

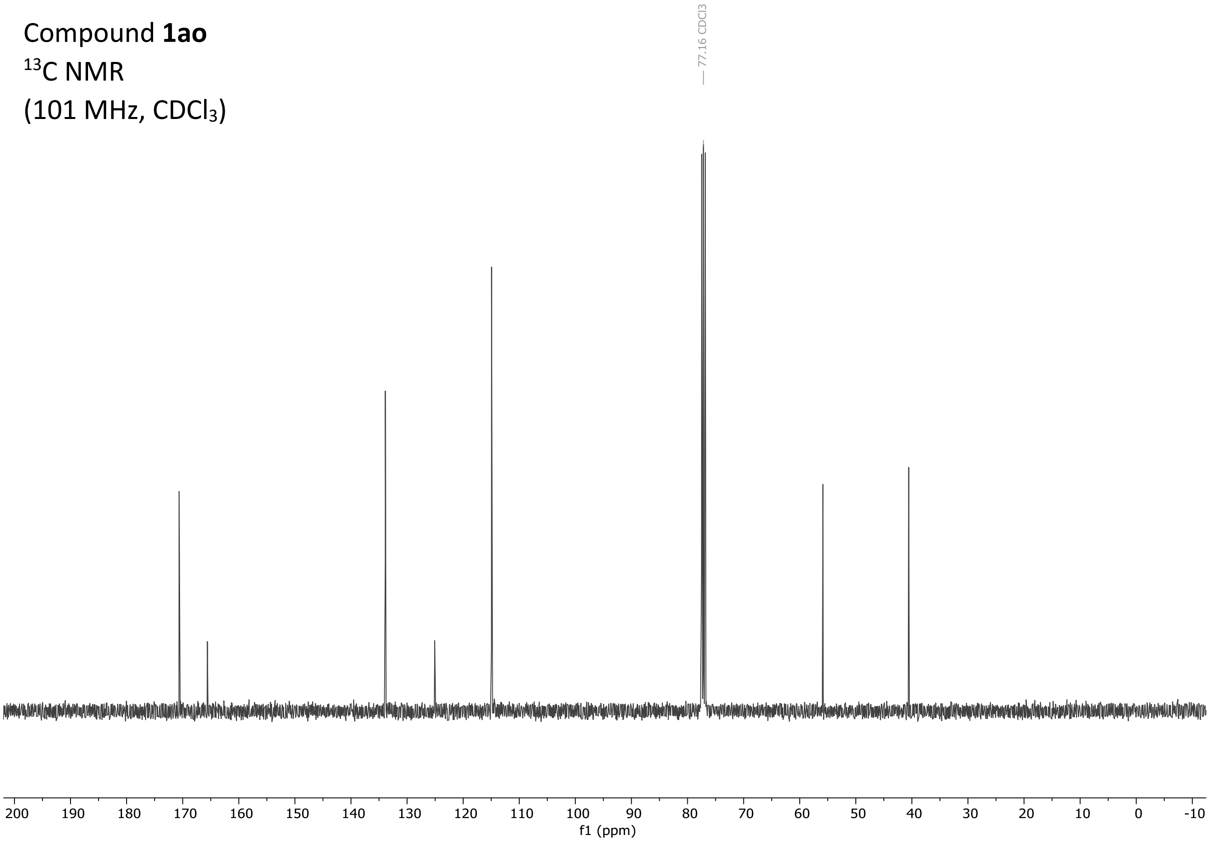

Compound **1ap**
^{1}H NMR
(400 MHz, $CDCl_3$)

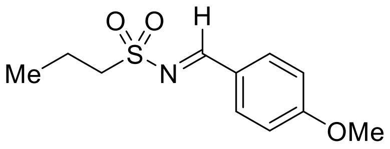

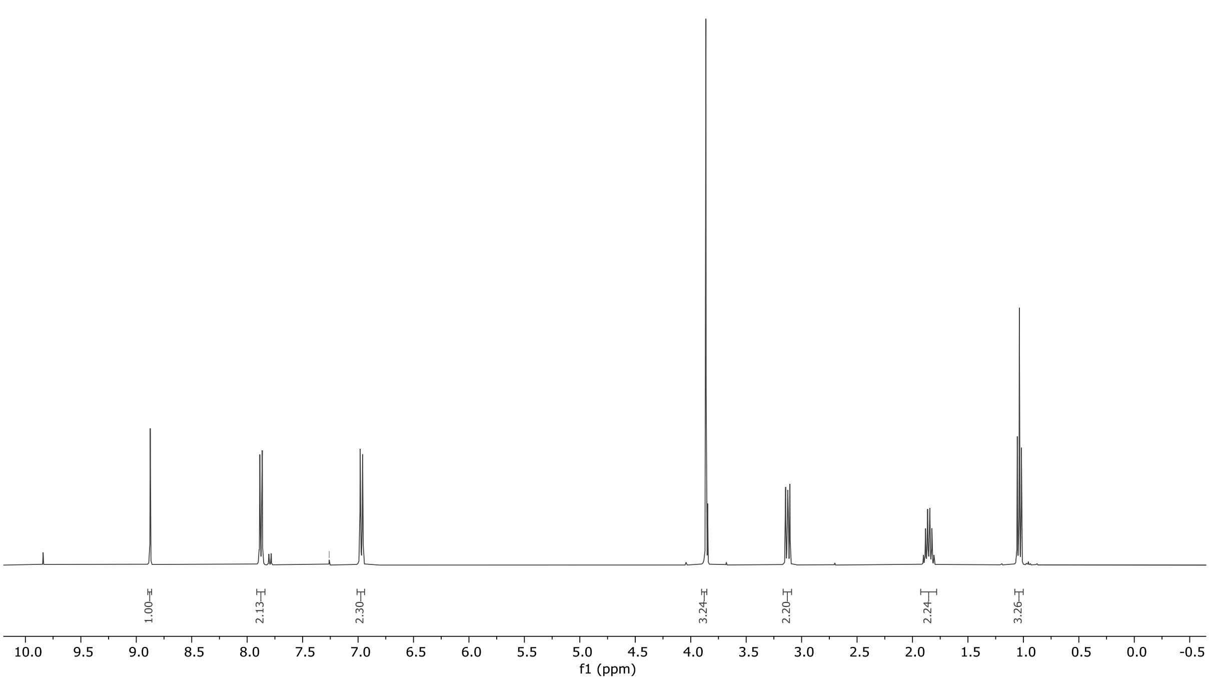

Compound **1ap**
^{13}C NMR
(101 MHz, $CDCl_3$)

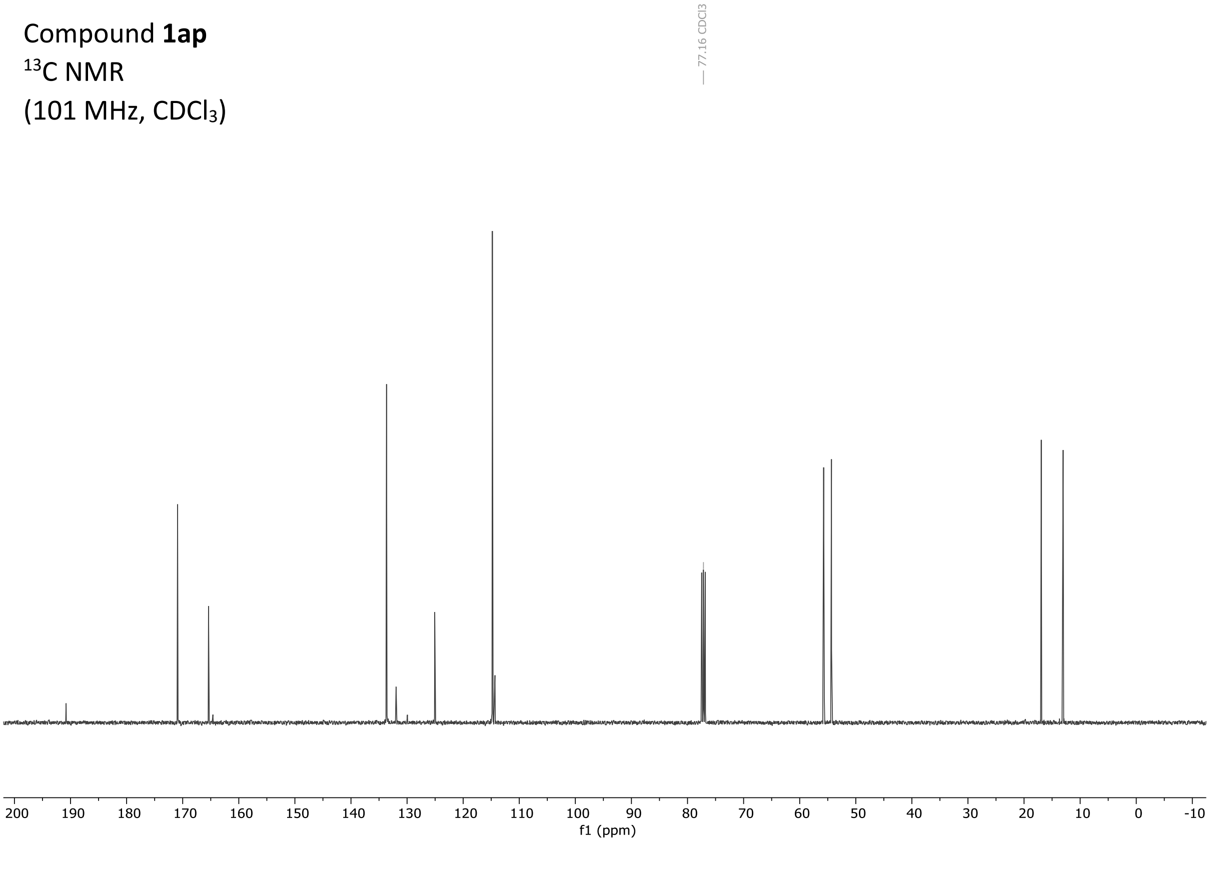

Compound **1aq**
^{1}H NMR
(400 MHz, $CDCl_3$)

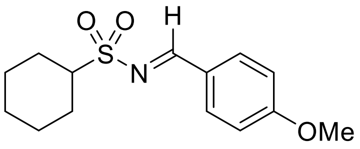

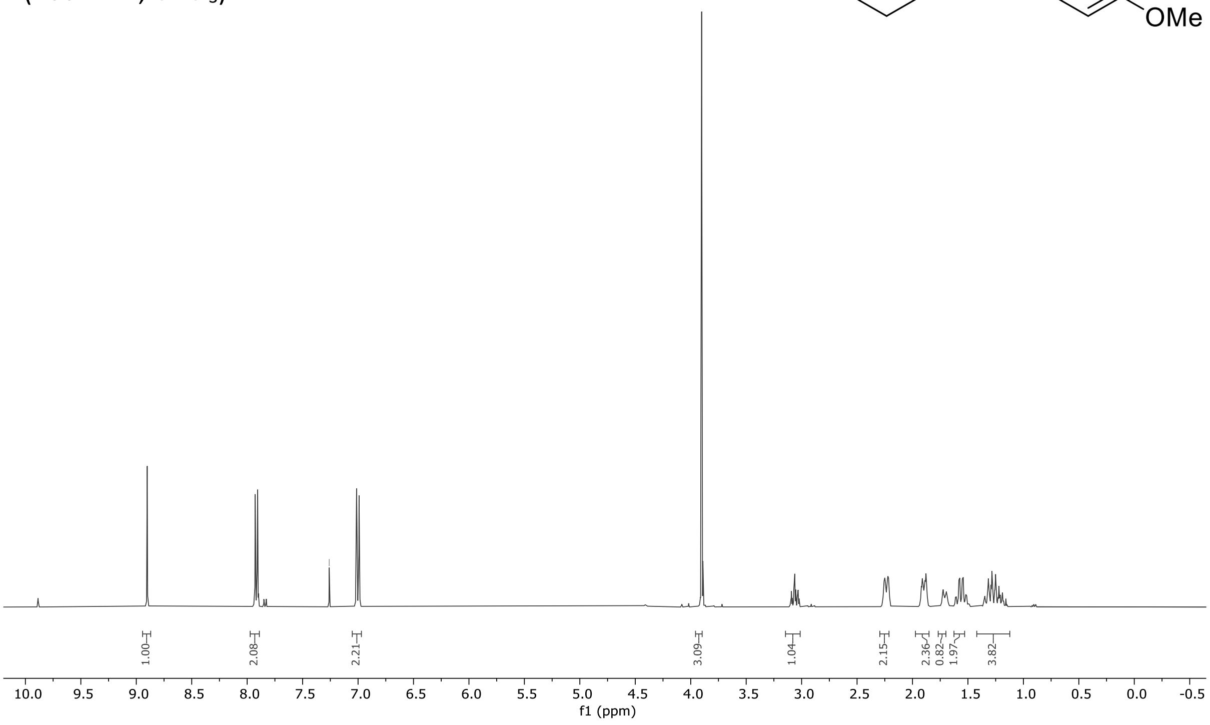

Compound **1aq**
^{13}C NMR
(101 MHz, $CDCl_3$)

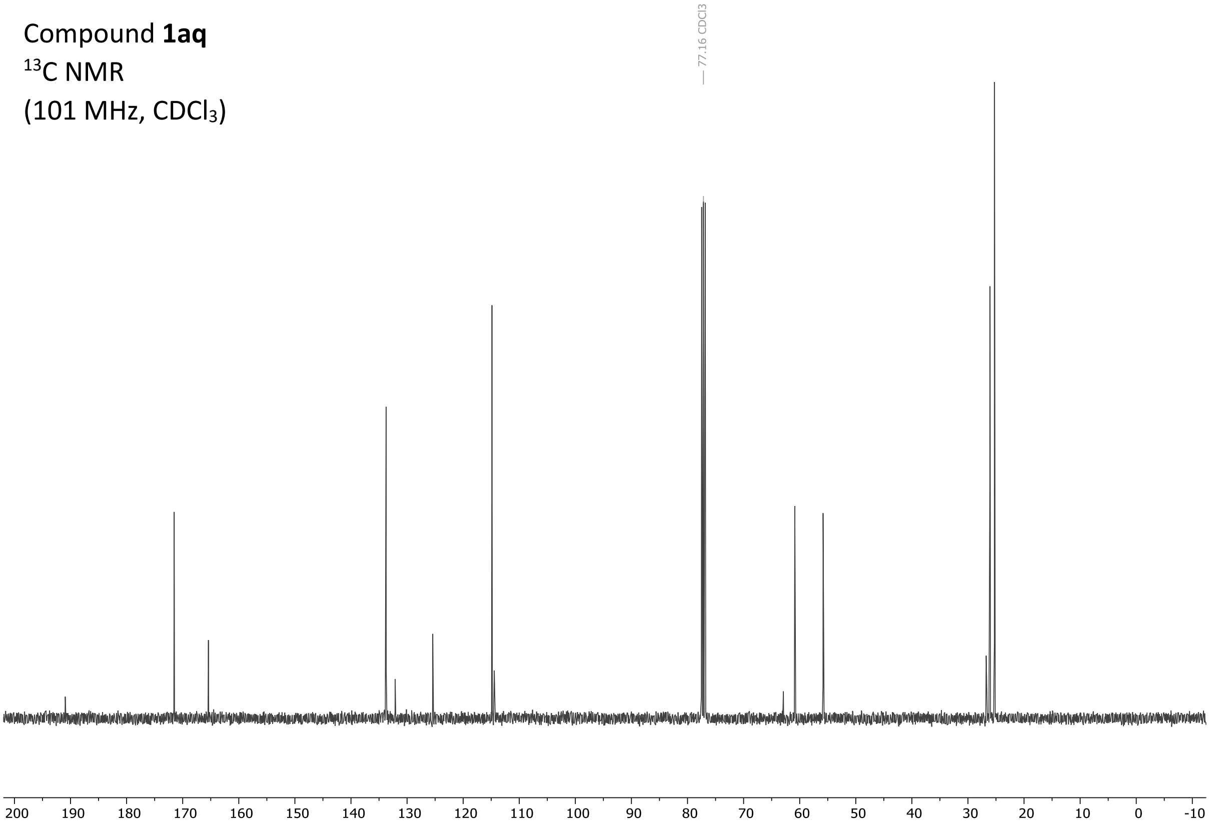

Compound **1ar**
^{1}H NMR
(400 MHz, $CDCl_3$)

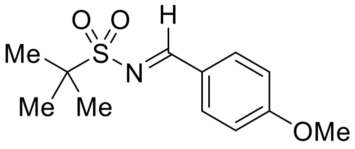

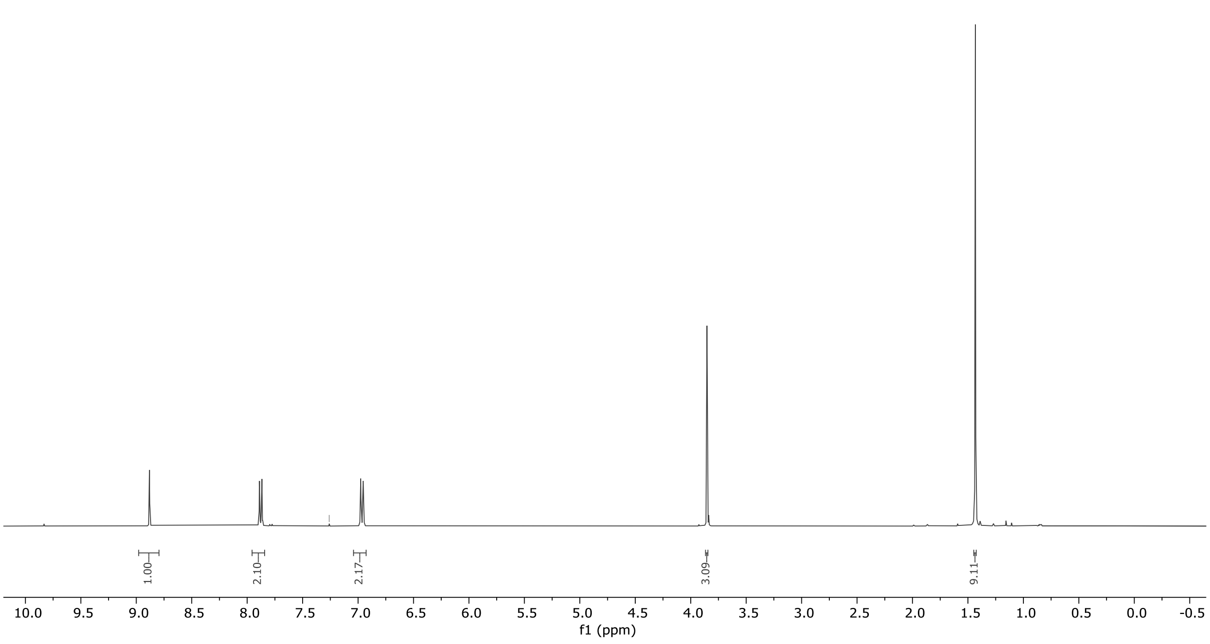

Compound **1ar**
^{13}C NMR
(101 MHz, $CDCl_3$)

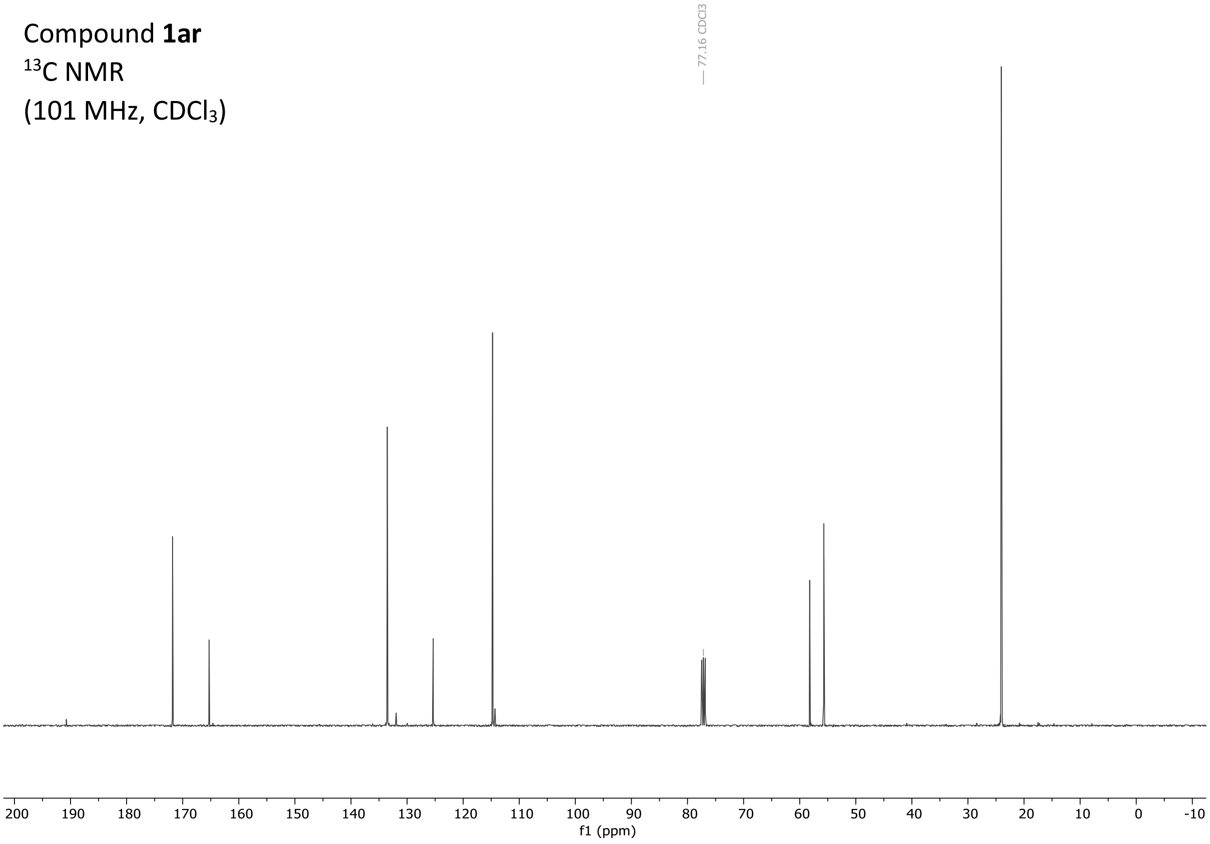

Compound **1as**
^{1}H NMR
(400 MHz, $CDCl_3$)

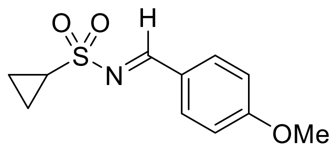

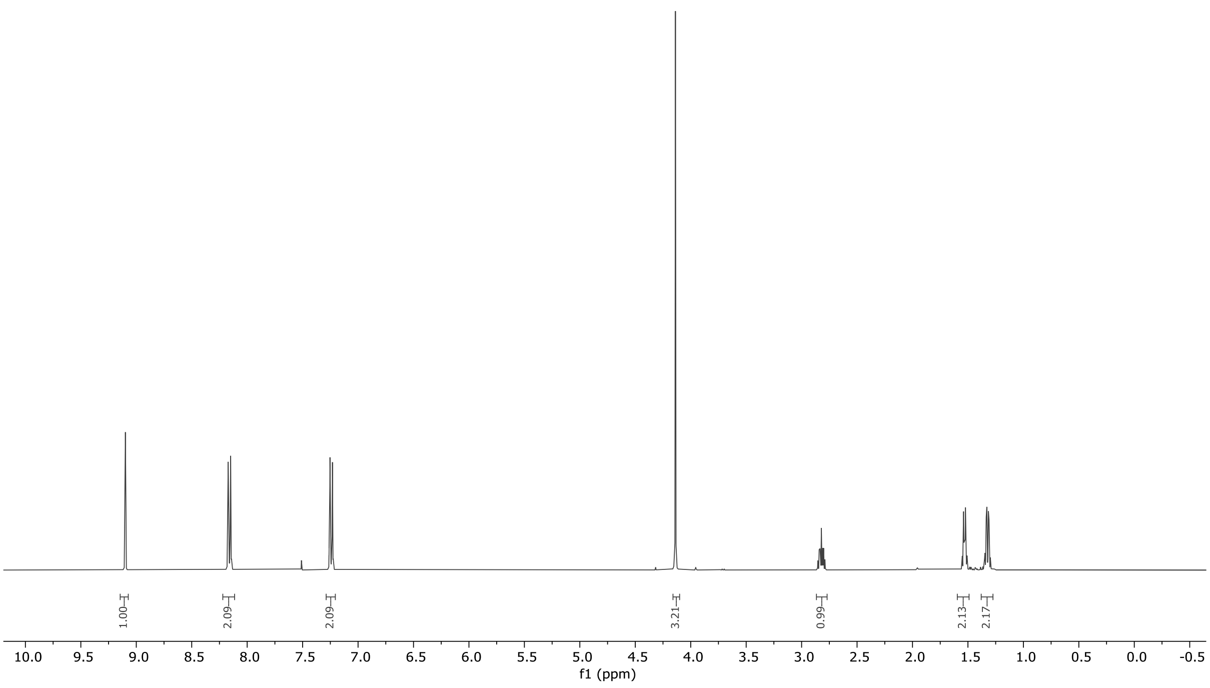

Compound **1as**
^{13}C NMR
(101 MHz, $CDCl_3$)

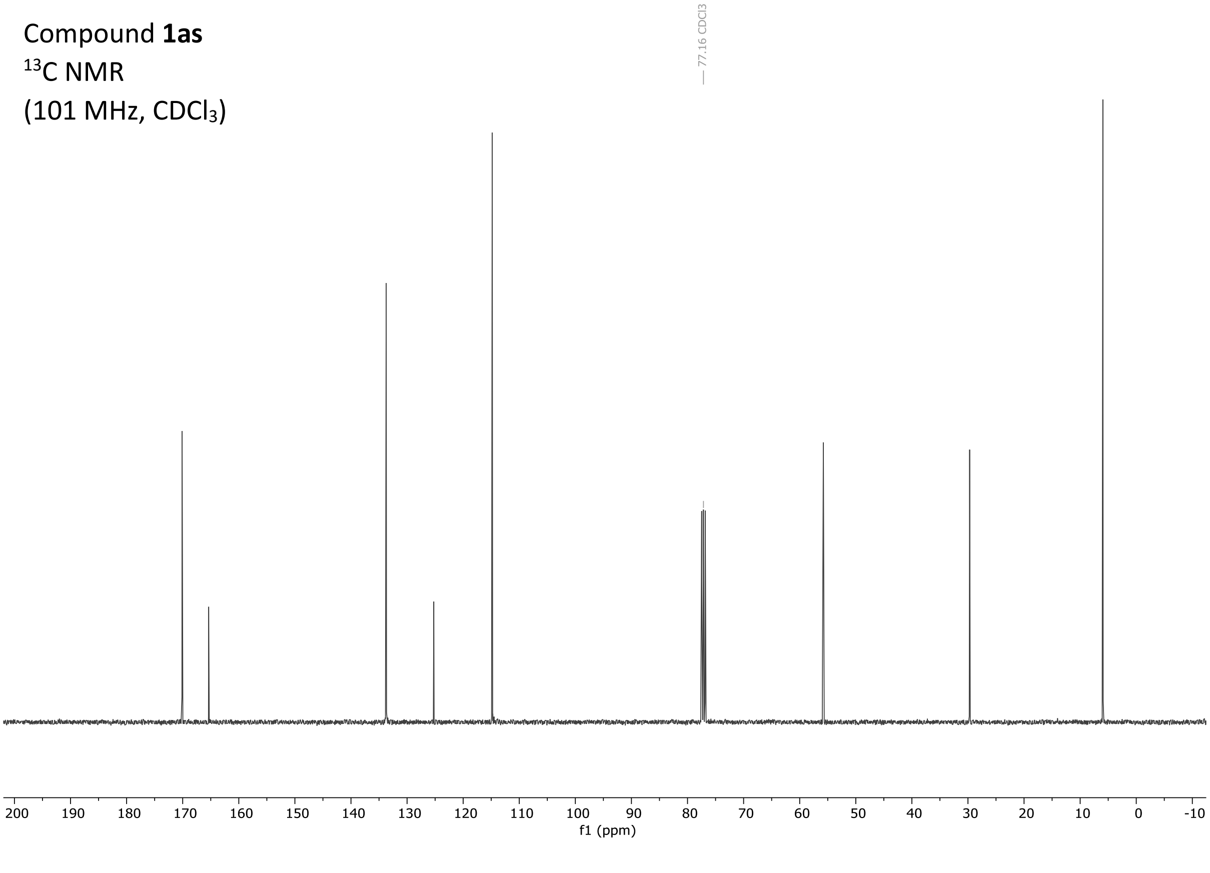

Compound **1at**
^{1}H NMR
(400 MHz, $CDCl_3$)

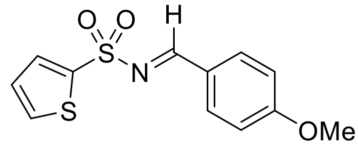

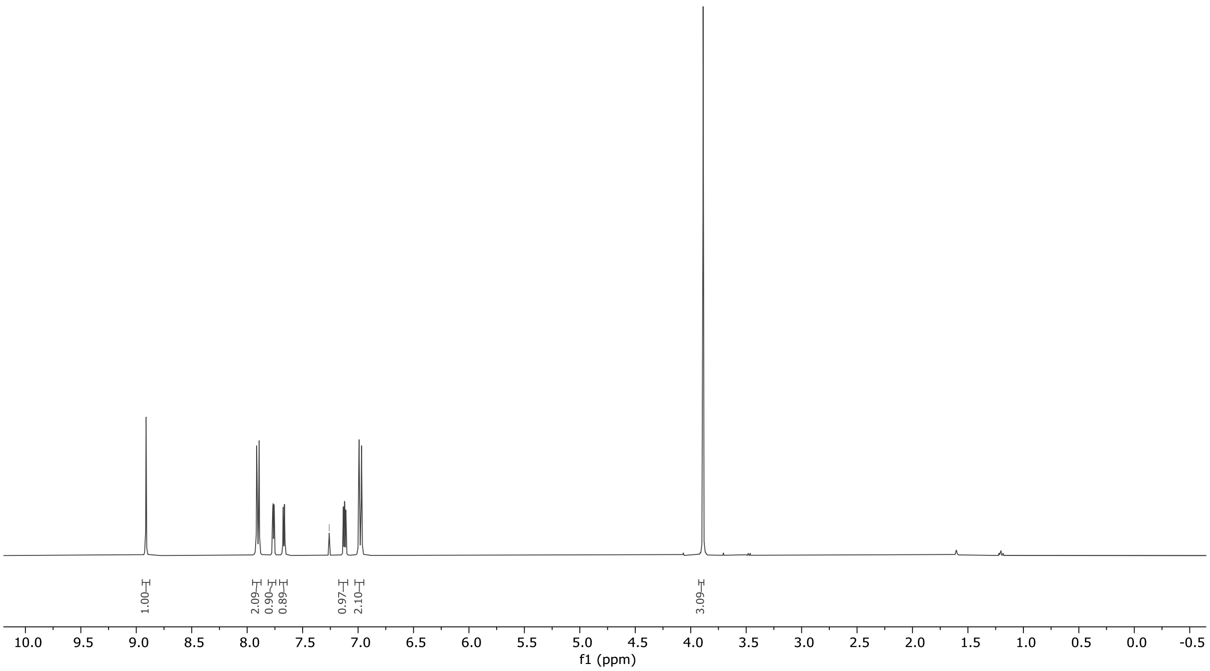

Compound **1at**
^{13}C NMR
(101 MHz, $CDCl_3$)

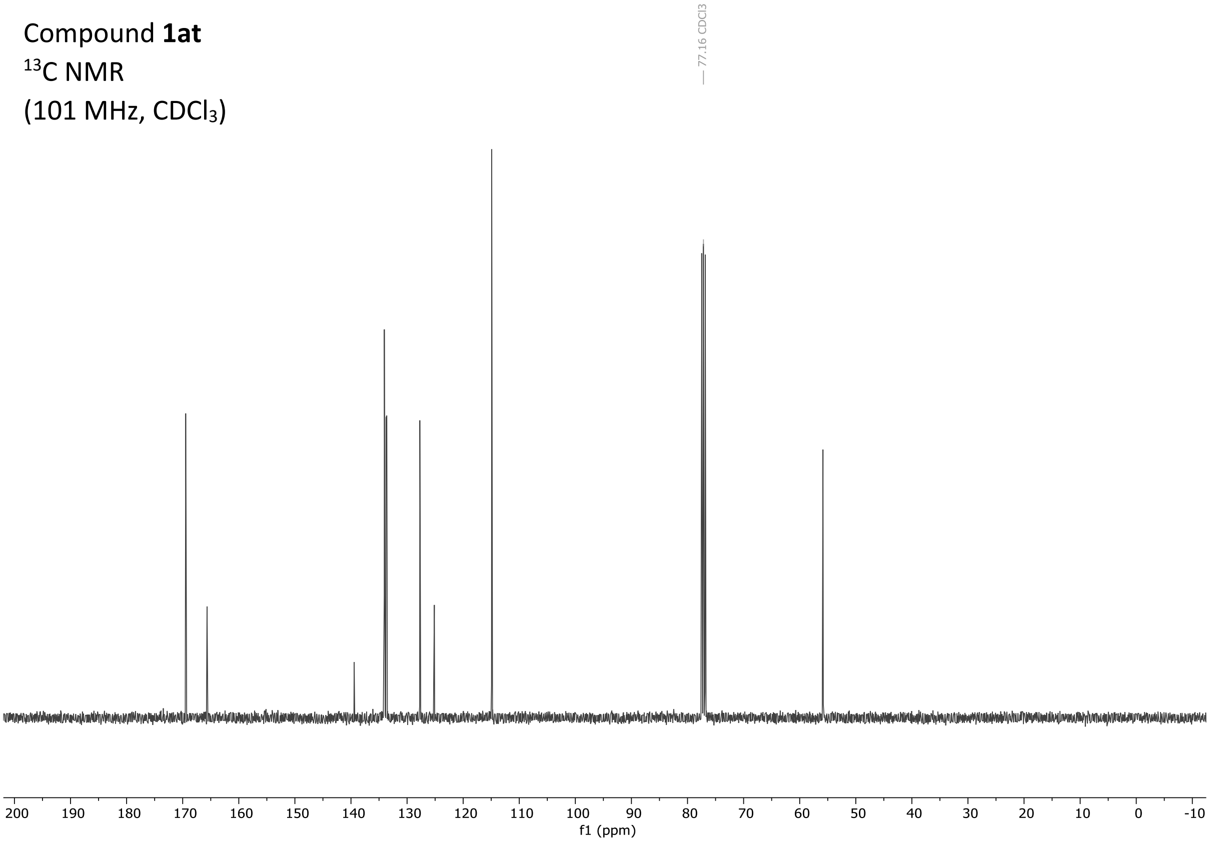

Compound **1au**
^{1}H NMR
(400 MHz, $CDCl_3$)

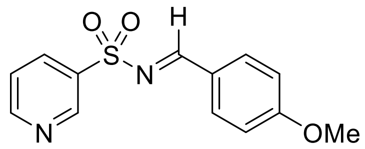

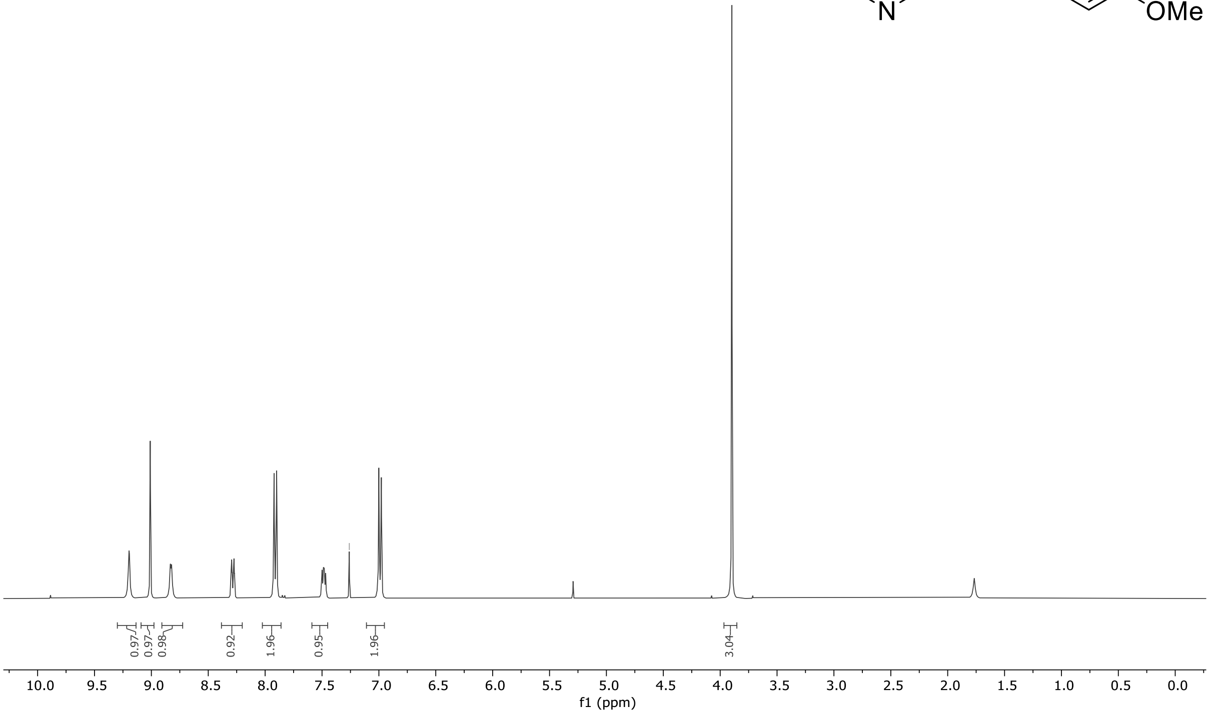

Compound **1au**
^{13}C NMR
(101 MHz, $CDCl_3$)

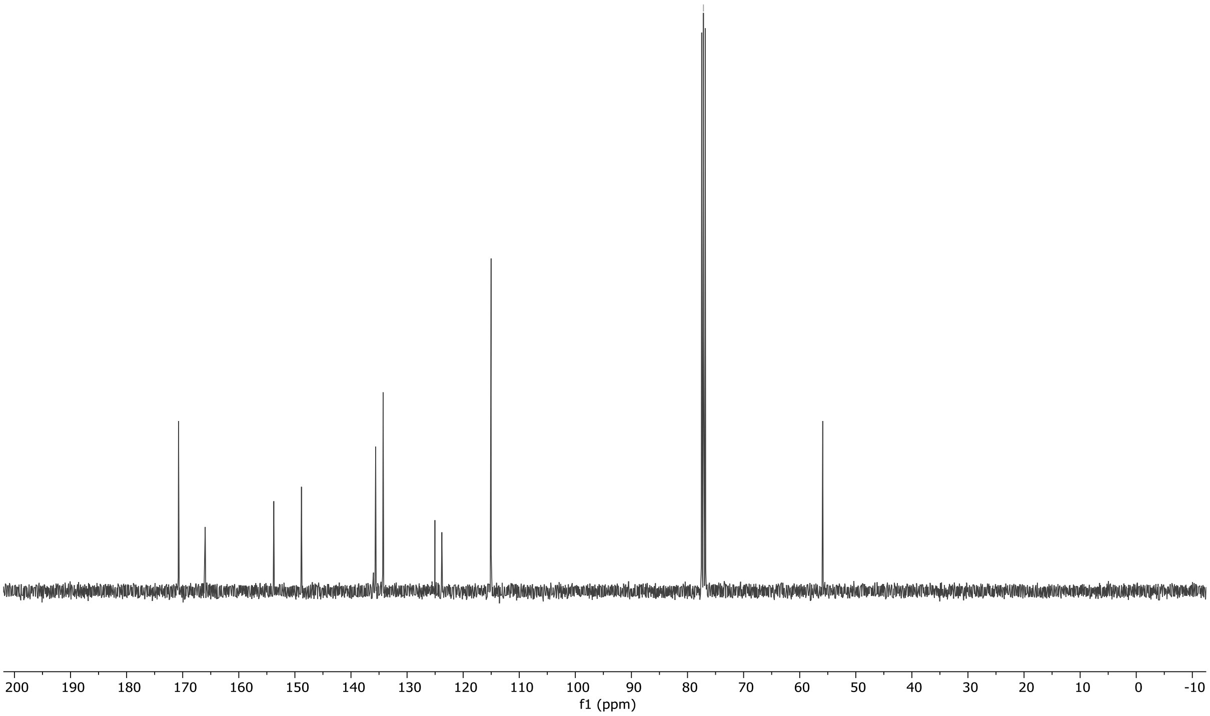

Compound **1av**
^{1}H NMR
(400 MHz, $CDCl_3$)

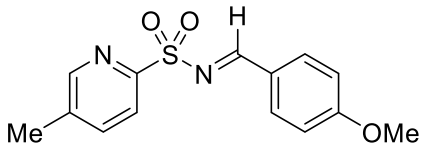

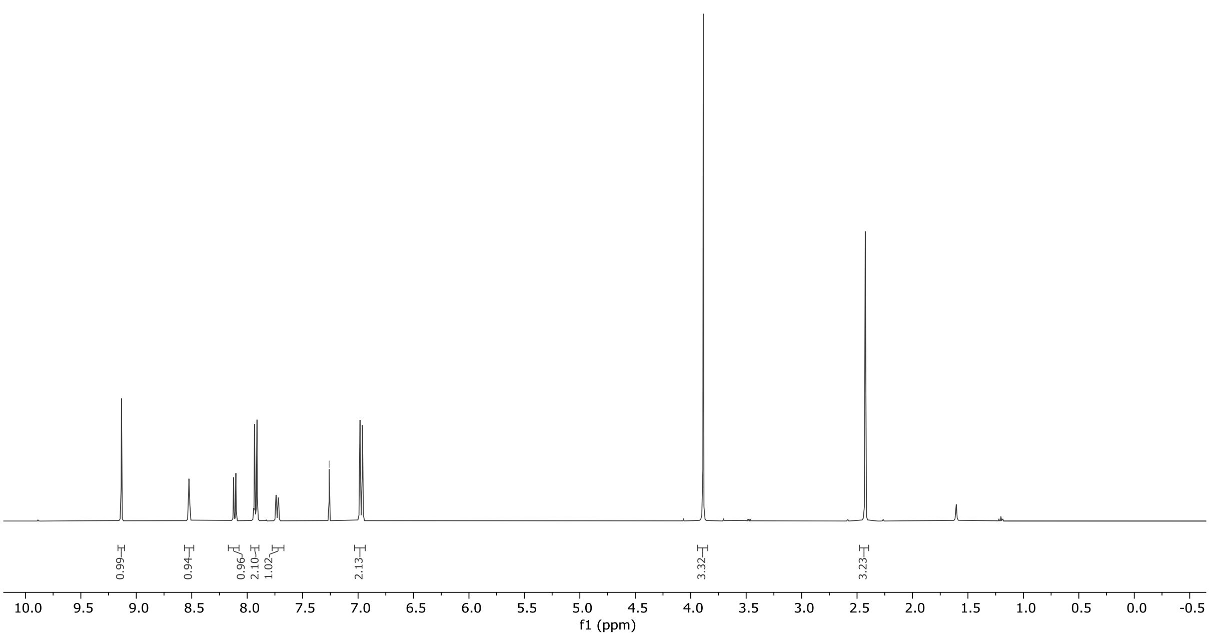

Compound **1av**
^{13}C NMR
(101 MHz, $CDCl_3$)

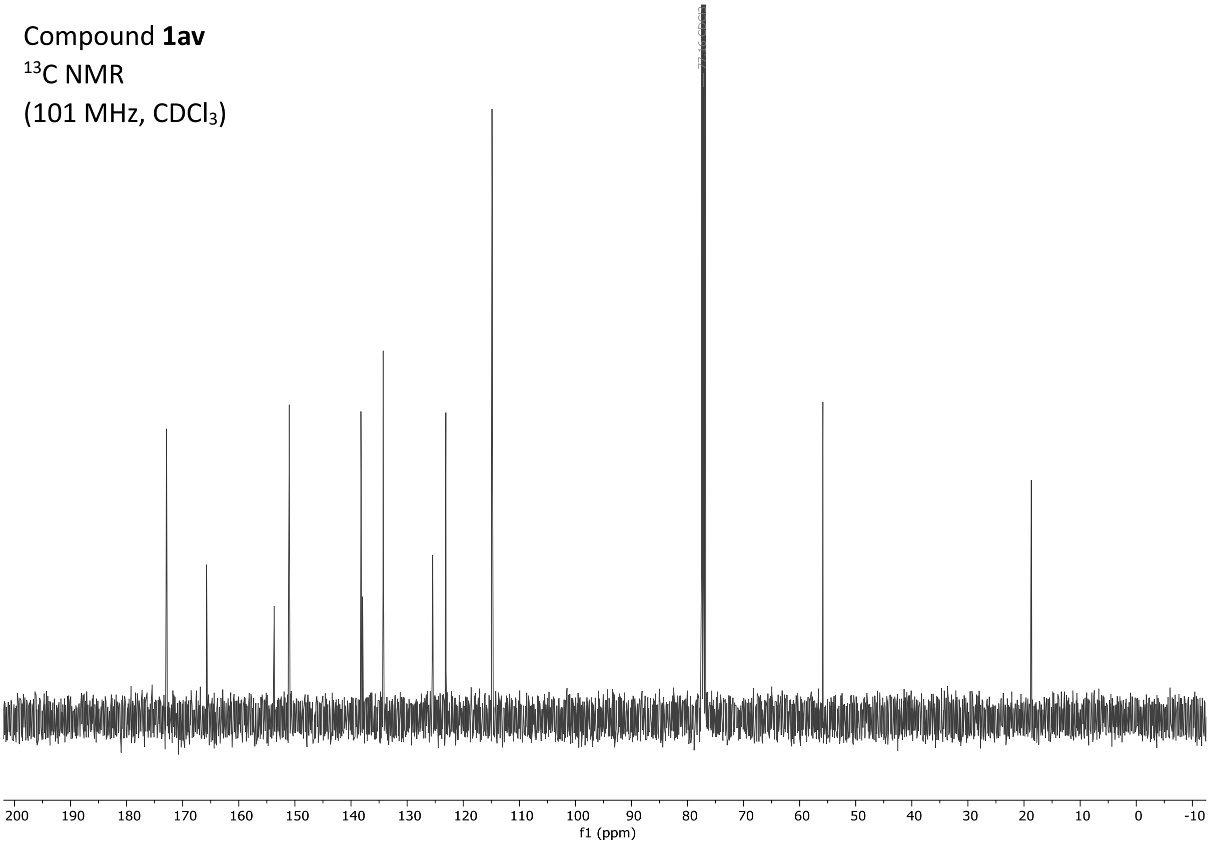

Compound **1aw**
^{1}H NMR
(400 MHz, $CDCl_3$)

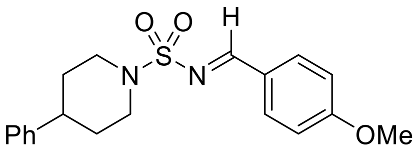

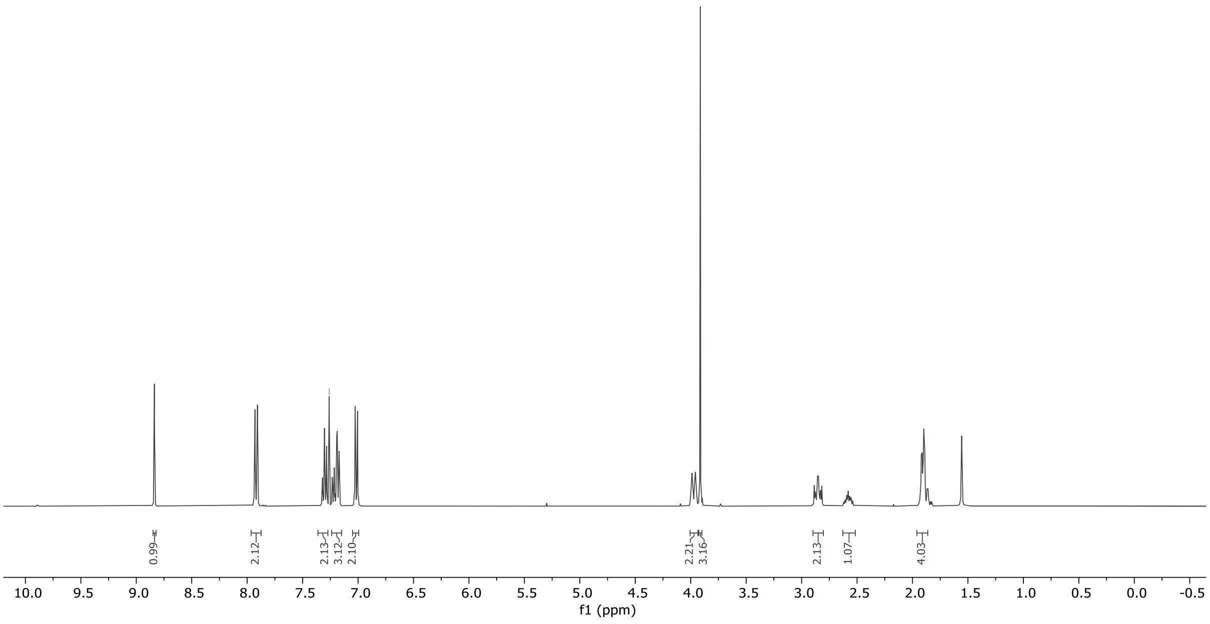

Compound **1aw**
^{13}C NMR
(101 MHz, $CDCl_3$)

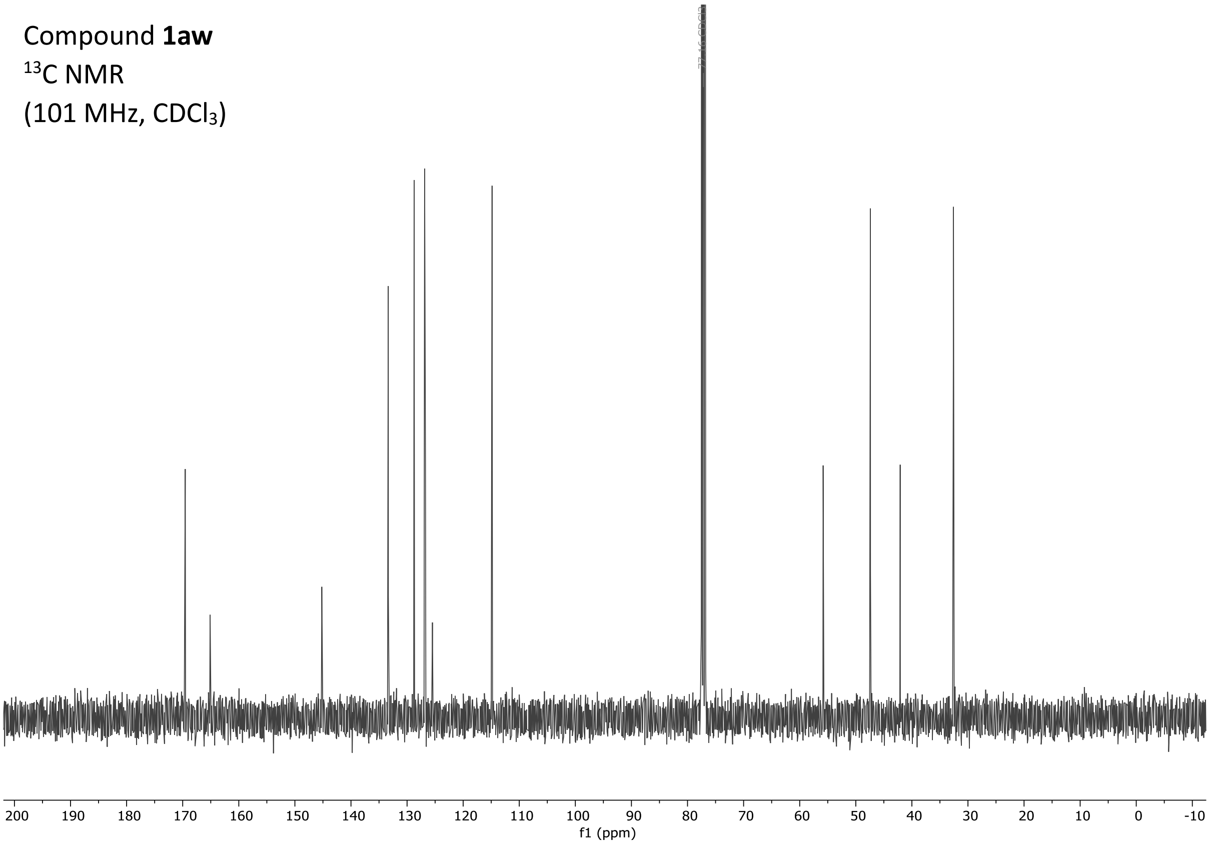

Compound **1ax**
^{1}H NMR
(400 MHz, $CDCl_3$)

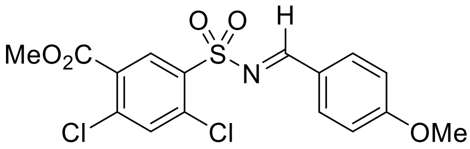

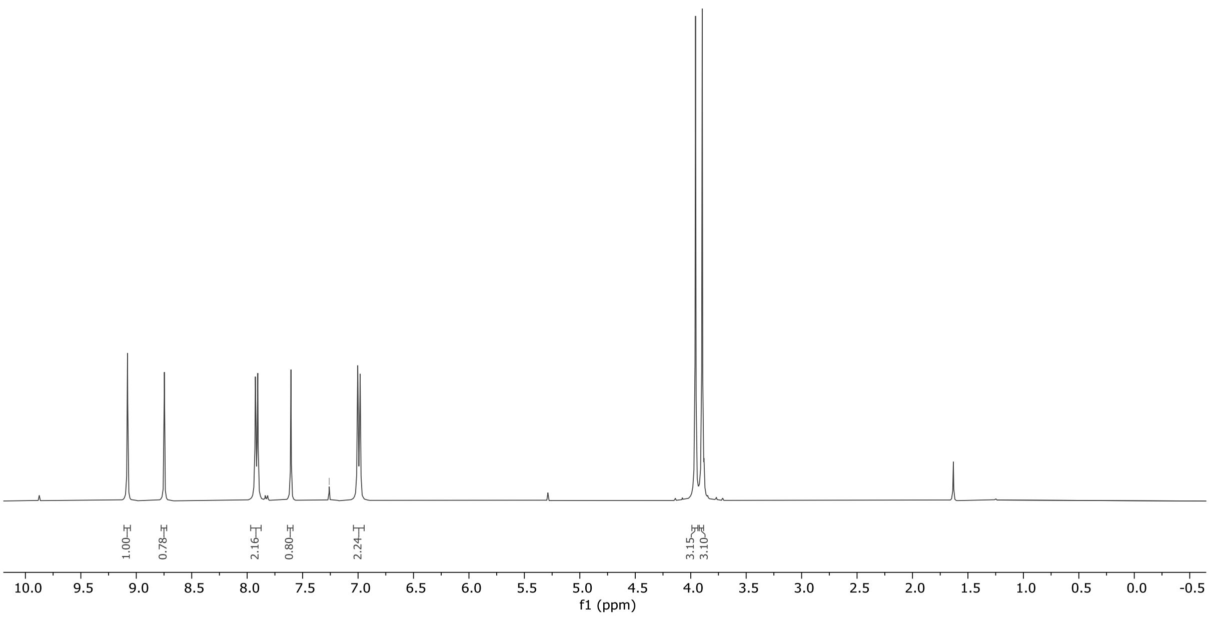

Compound **1ax**
^{1}H NMR
(101 MHz, $CDCl_3$)

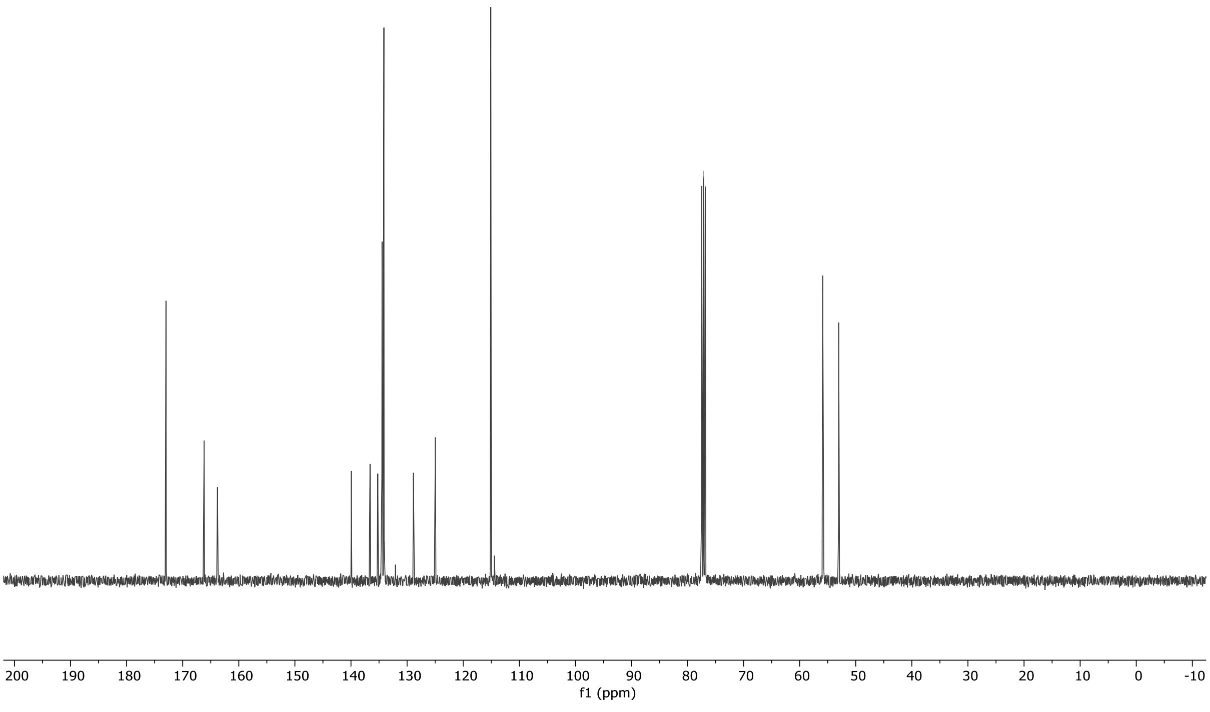

Compound **1ay**
^{1}H NMR
(400 MHz, DMSO-d_6)

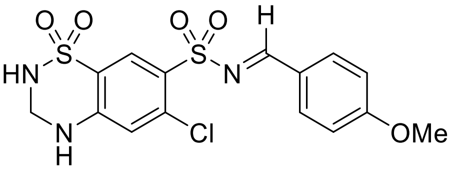

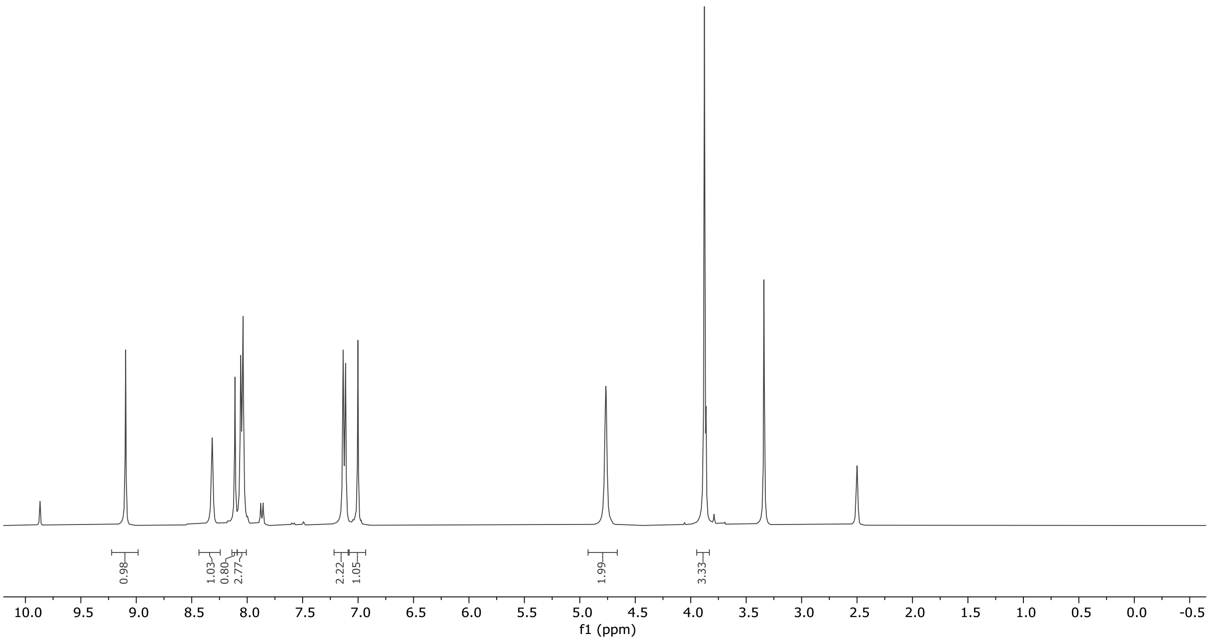

Compound **1ay**
^{13}C NMR
(101 MHz, DMSO-d_6)

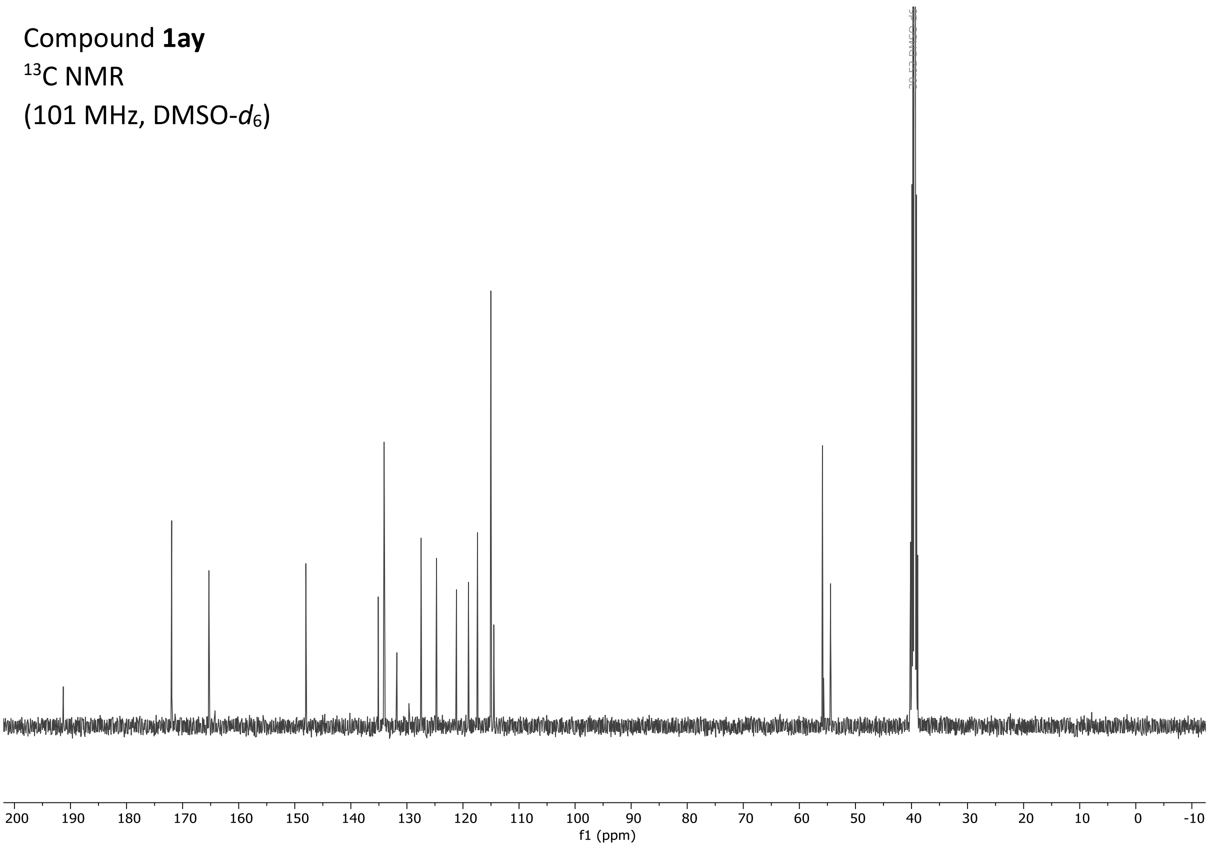

Compound **1az**
^{1}H NMR
(400 MHz, DMSO-d_6)

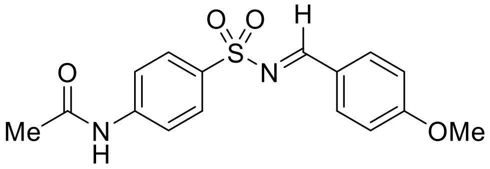

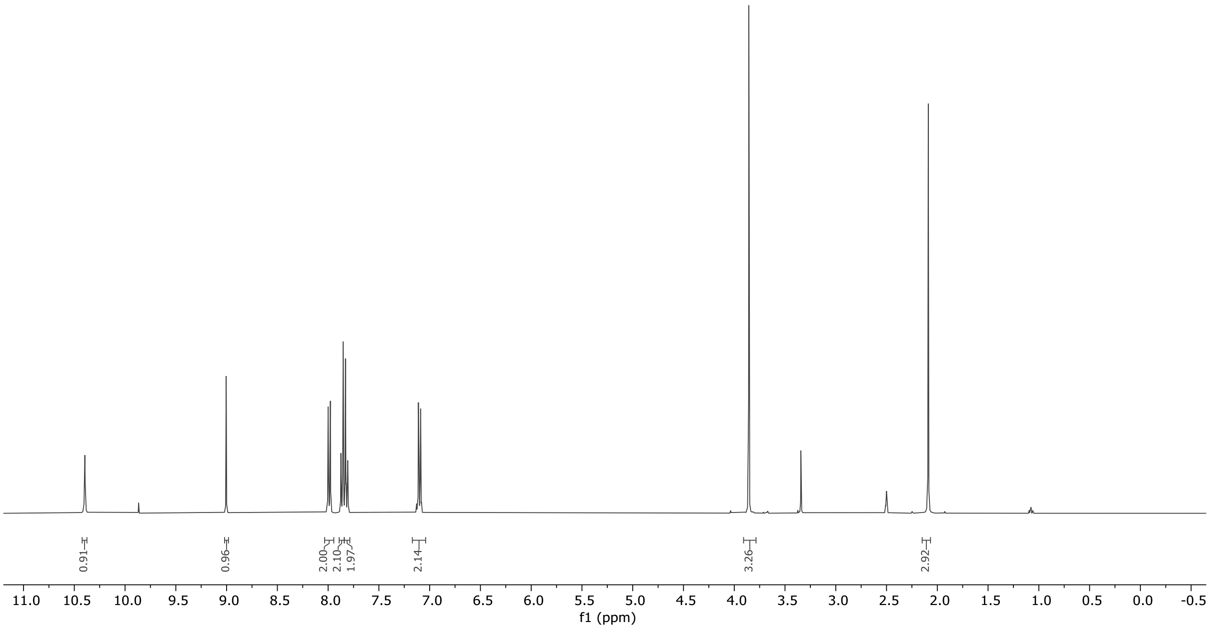

Compound **1az**
^{13}C NMR
(101 MHz, DMSO-d_6)

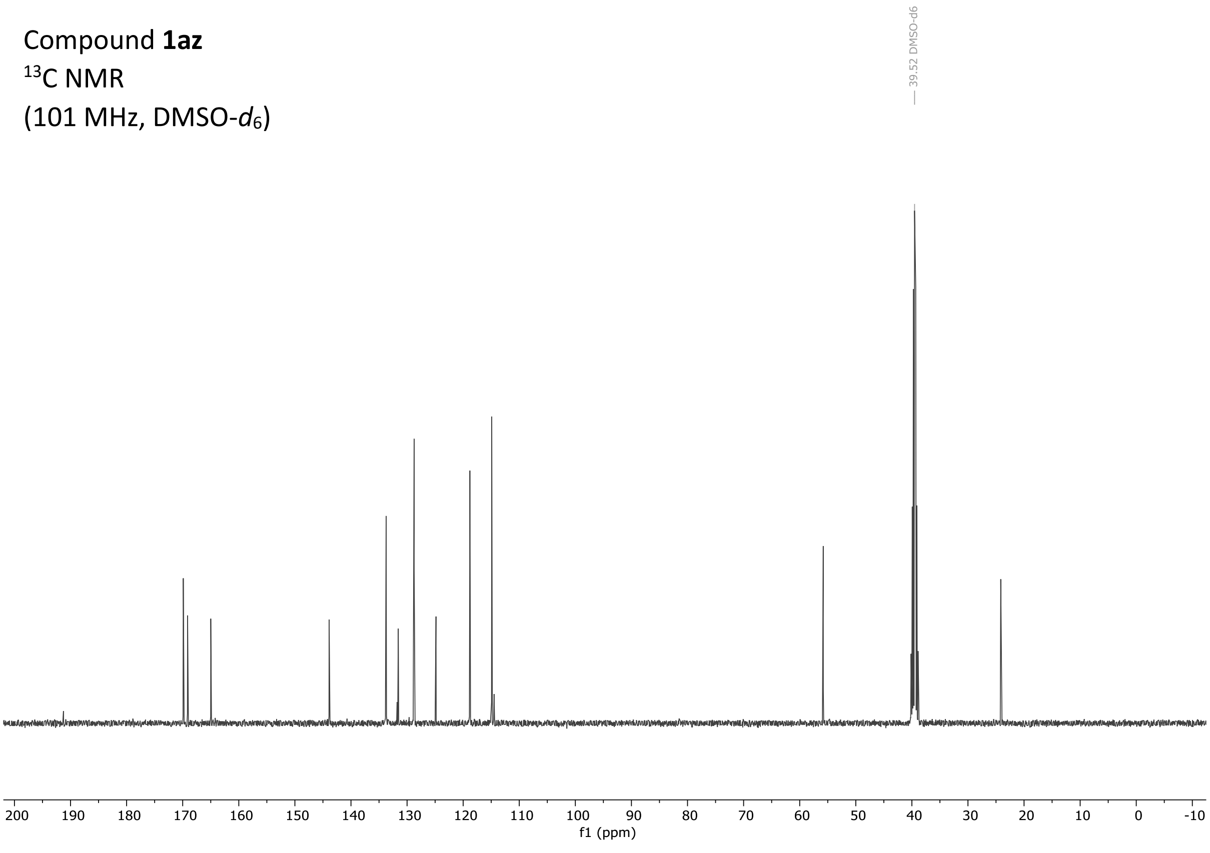

Compound **3a**
^{1}H NMR
(400 MHz, $CDCl_3$)

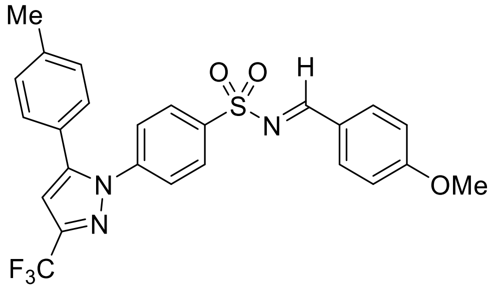

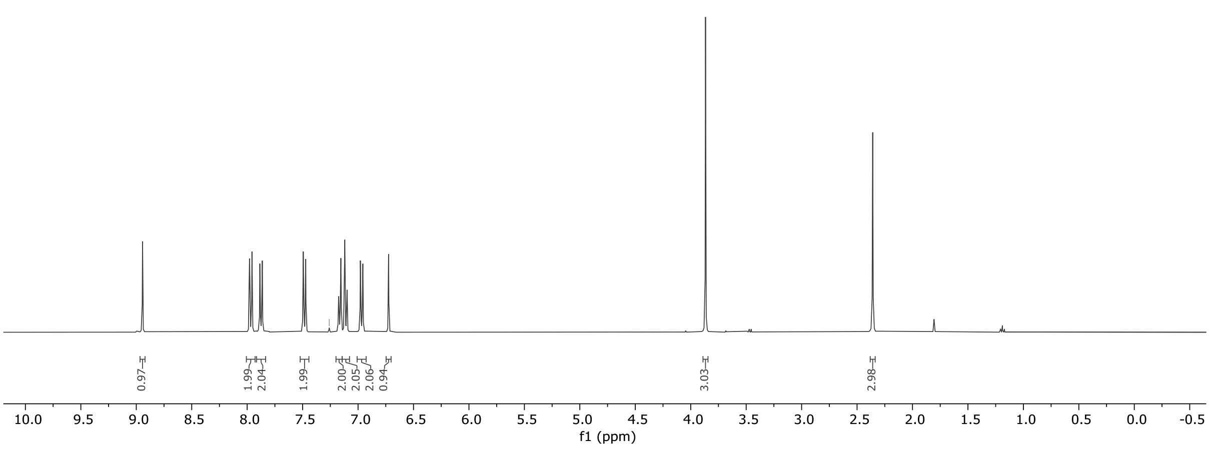

Compound **3a**
^{13}C NMR
(101 MHz, $CDCl_3$)

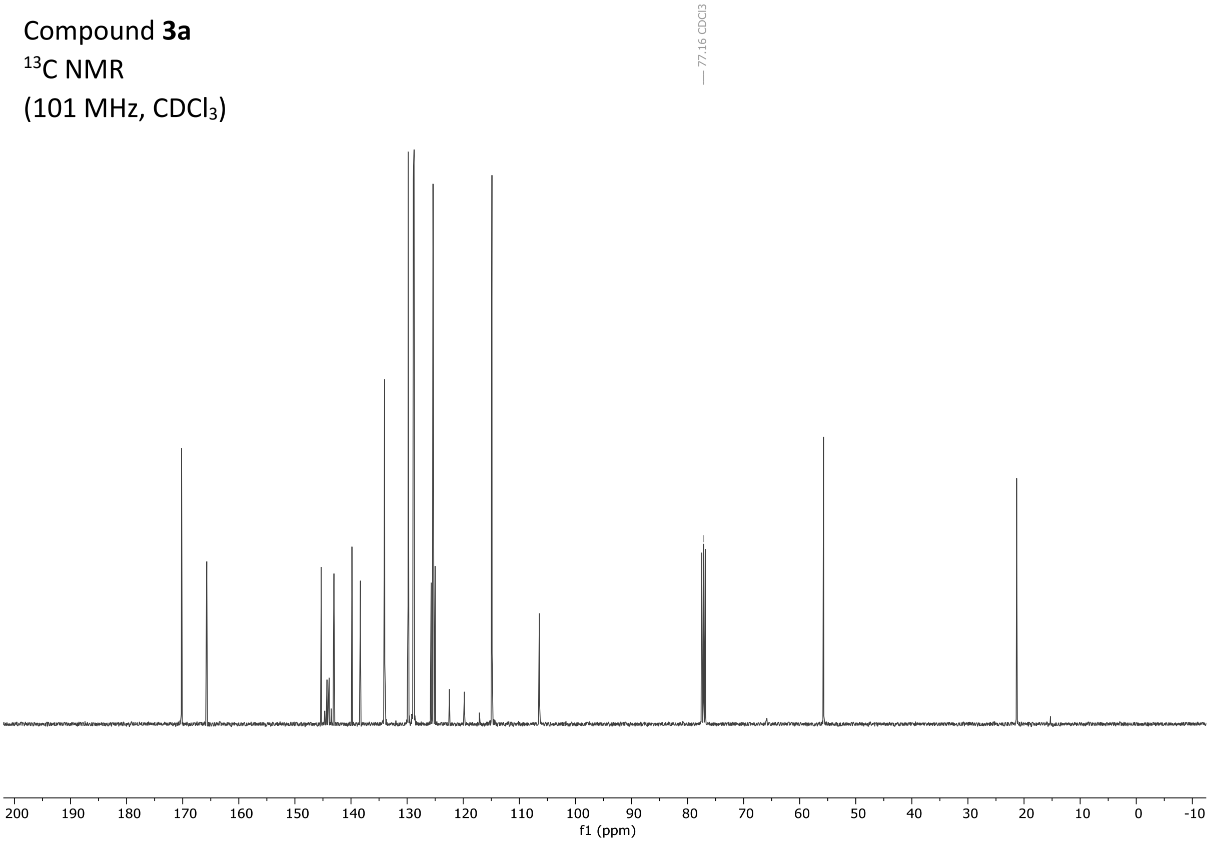

Compound **3a**
^{19}F NMR
(377 MHz, $CDCl_3$)

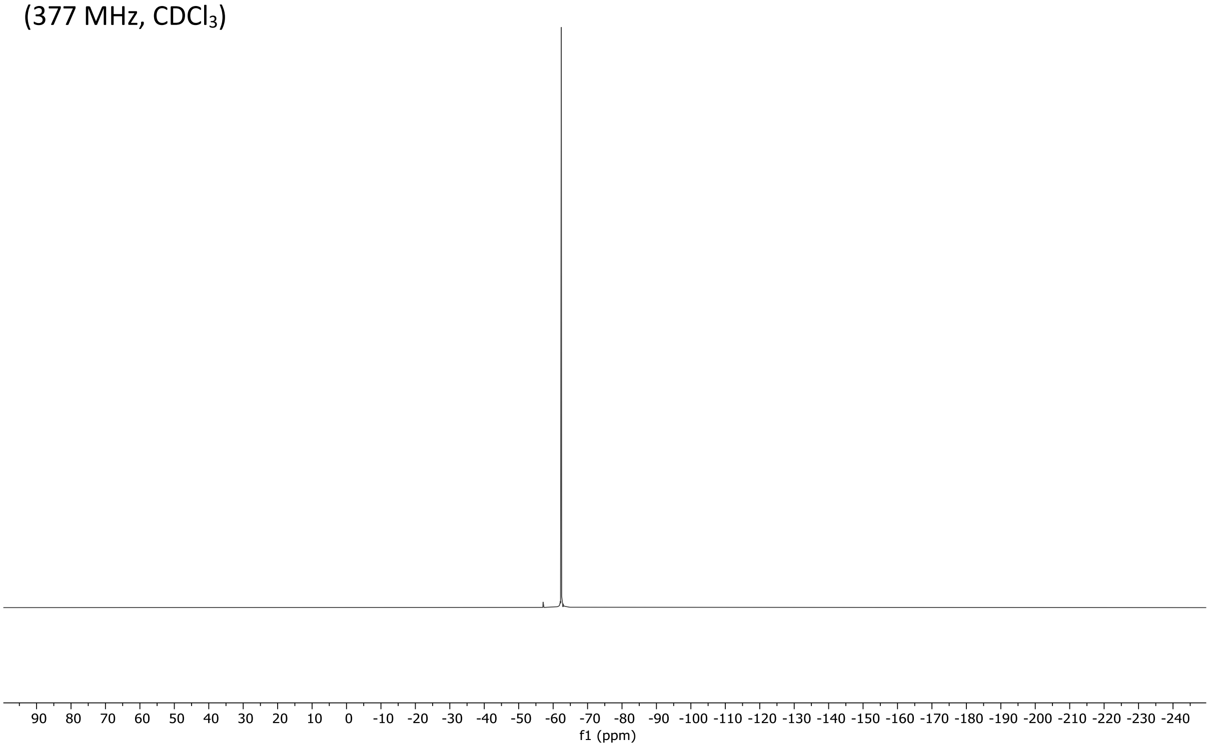

Compound **3b**
^{1}H NMR
(400 MHz, $CDCl_3$)

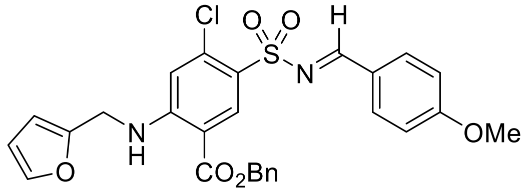

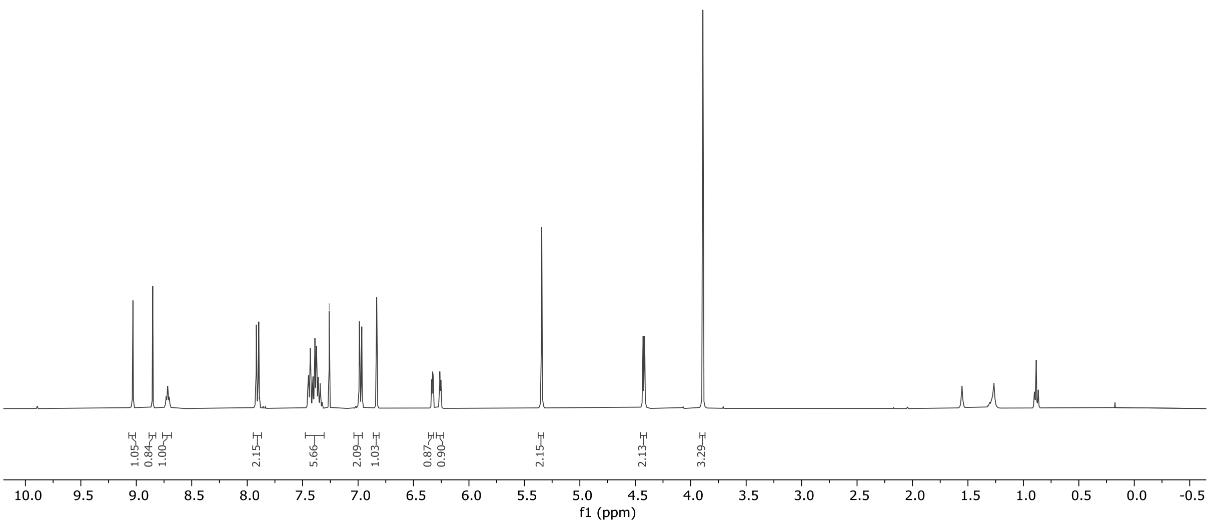

Compound **3b**
^{13}C NMR
(101 MHz, $CDCl_3$)

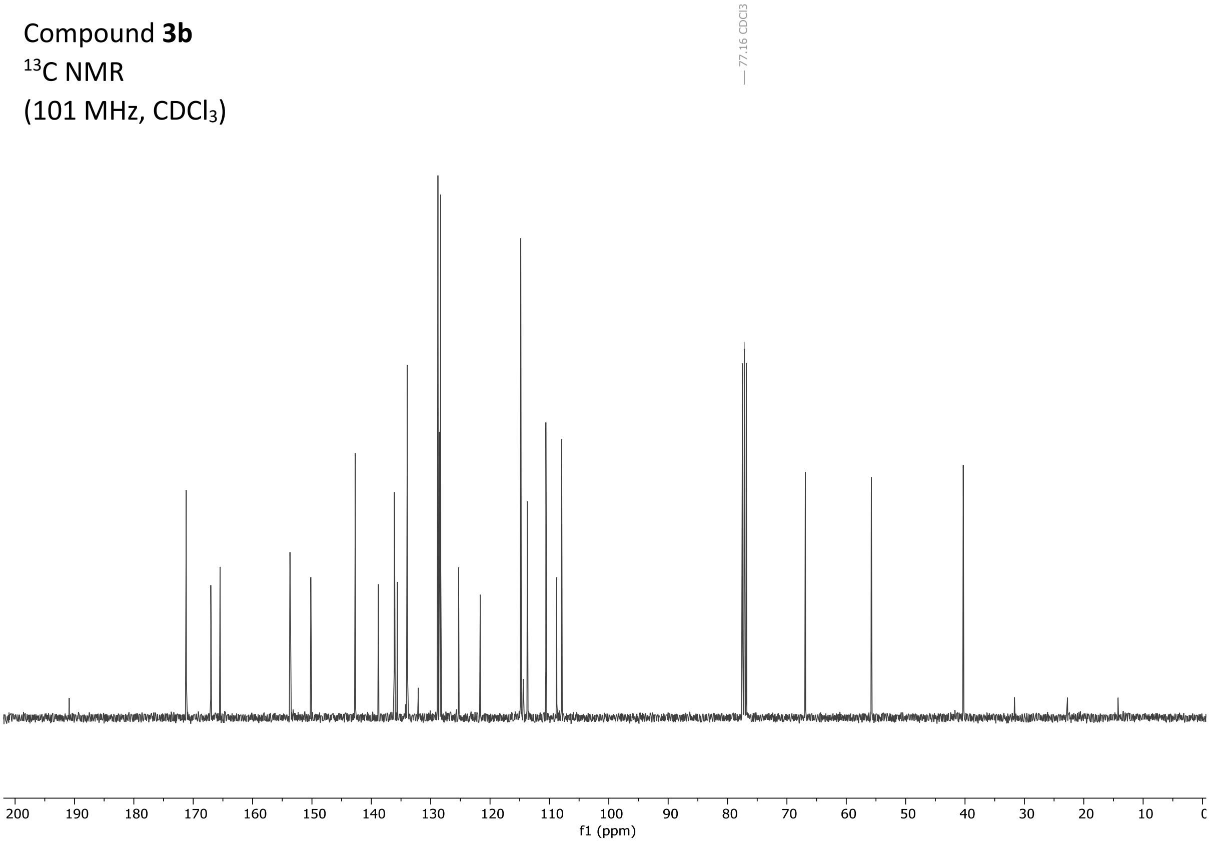

Compound **2a**
^{1}H NMR
(400 MHz, $CDCl_3$)

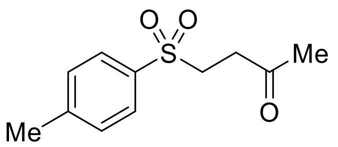

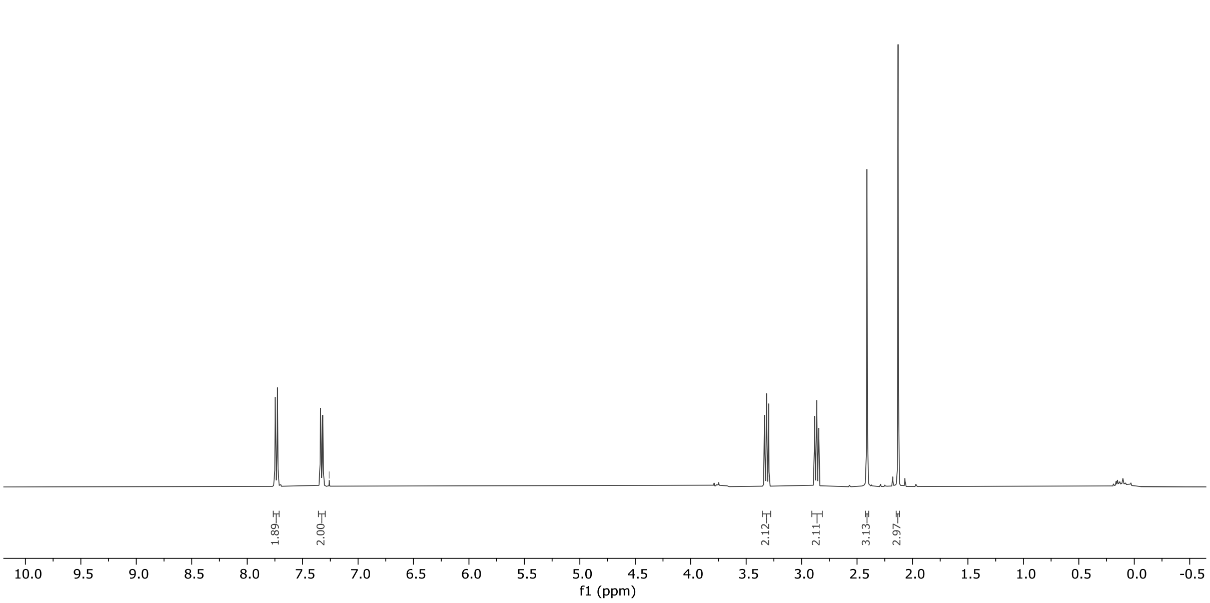

Compound **2a**
^{13}C NMR
(101 MHz, $CDCl_3$)

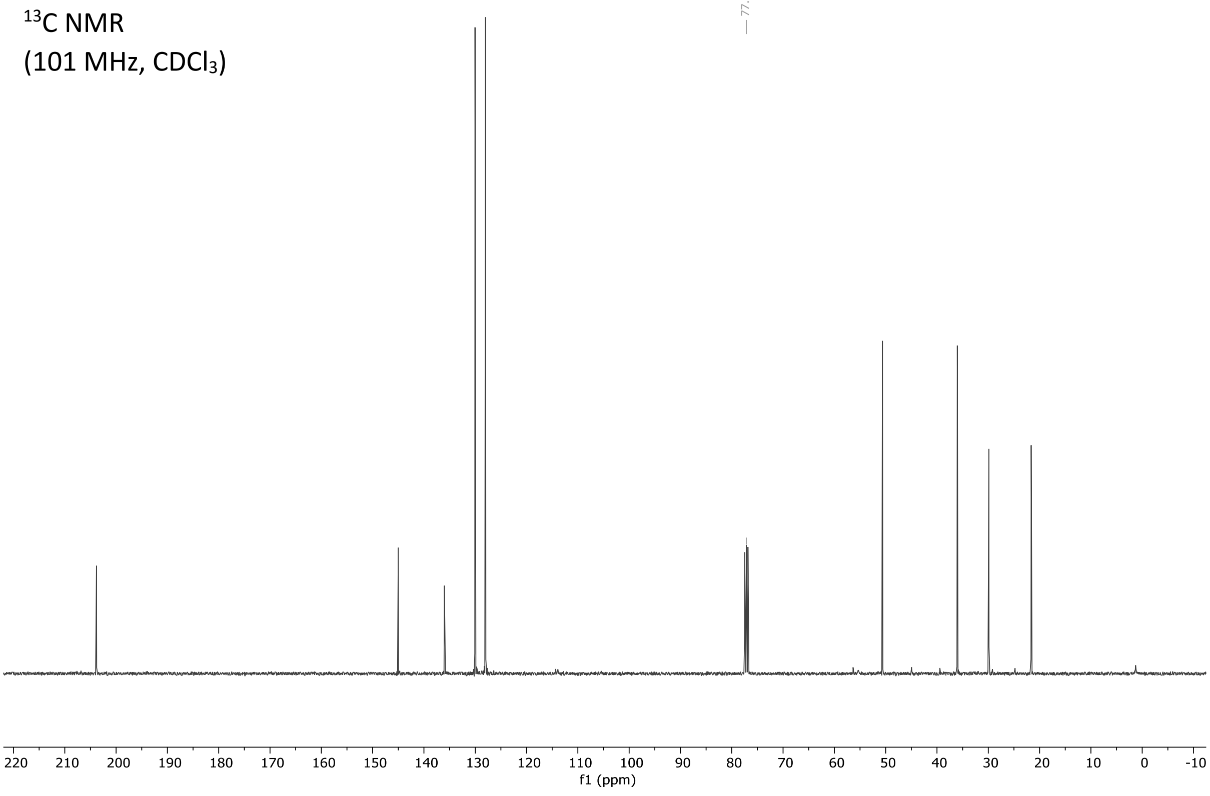

Compound **2b**
^{1}H NMR
(400 MHz, $CDCl_3$)

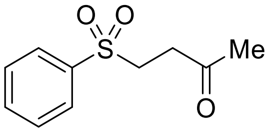

7.26 CDCl3

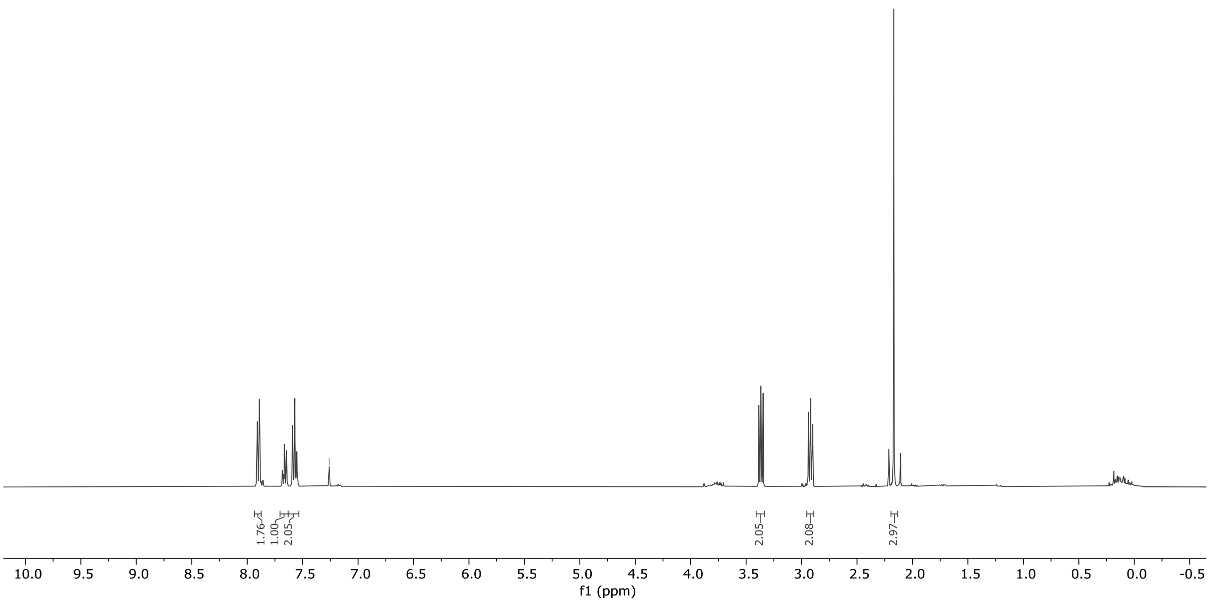

Compound **2b**
^{13}C NMR
(101 MHz, $CDCl_3$)

77.16 CDCl3

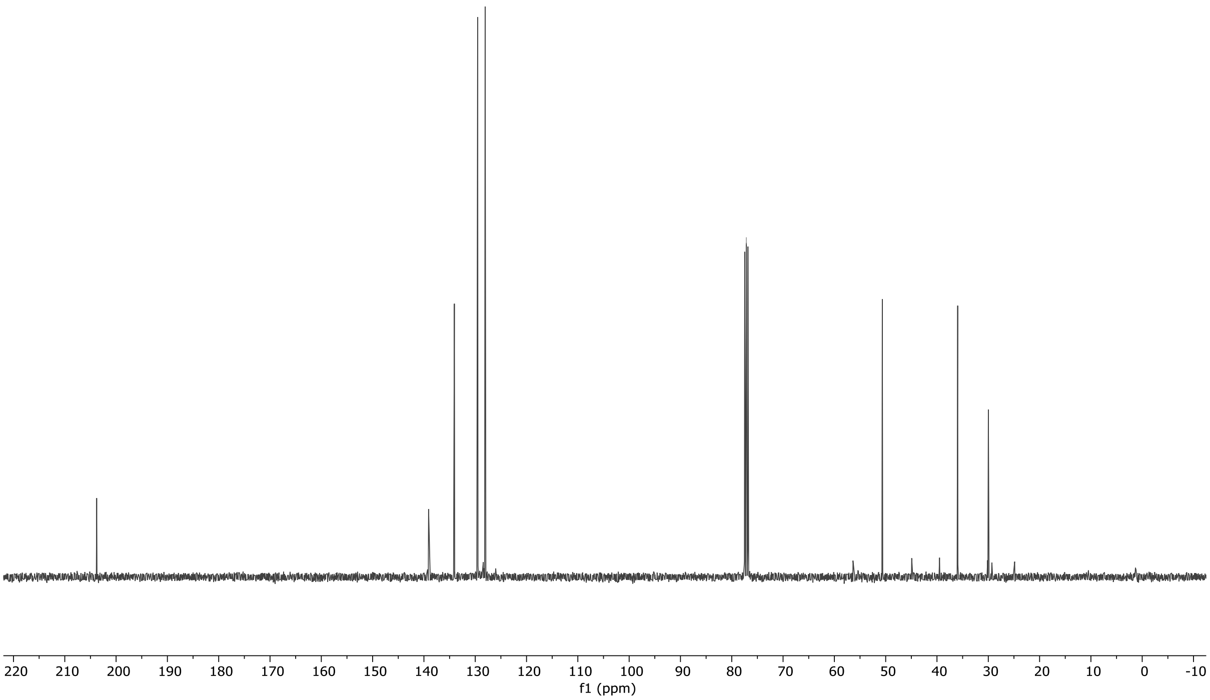

Compound **2c**
^{1}H NMR
(400 MHz, $CDCl_3$)

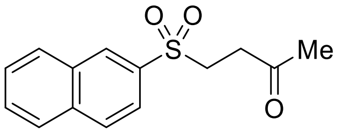

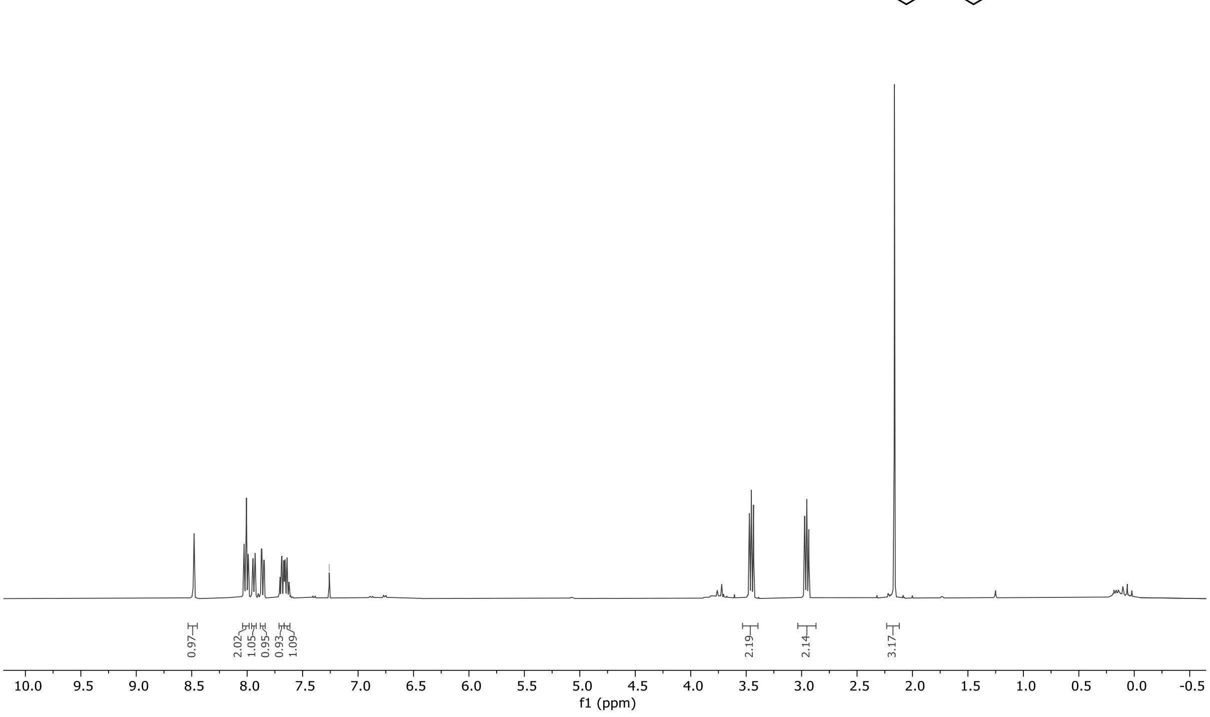

Compound **2c**
^{13}C NMR
(101 MHz, $CDCl_3$)

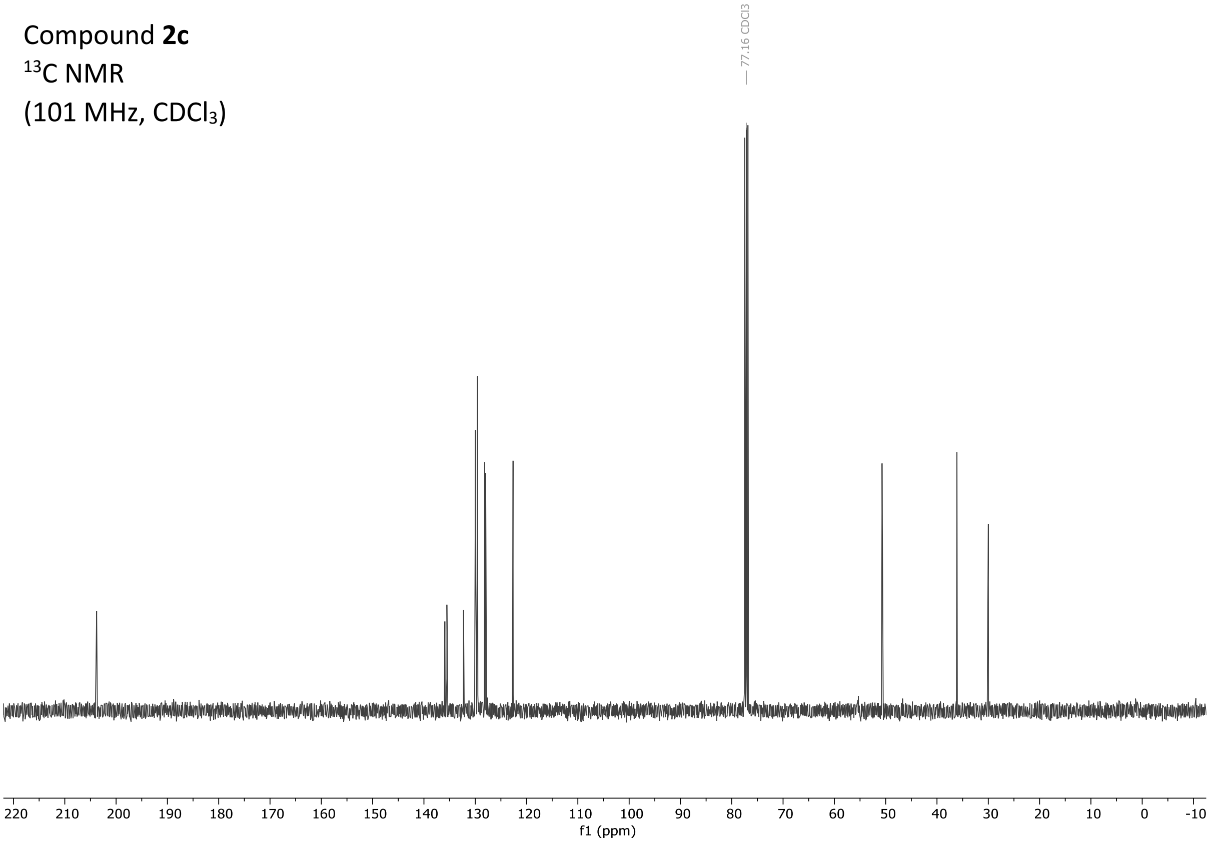

Compound **2d**
^{1}H NMR
(400 MHz, $CDCl_3$)

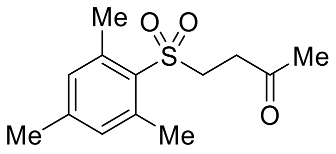

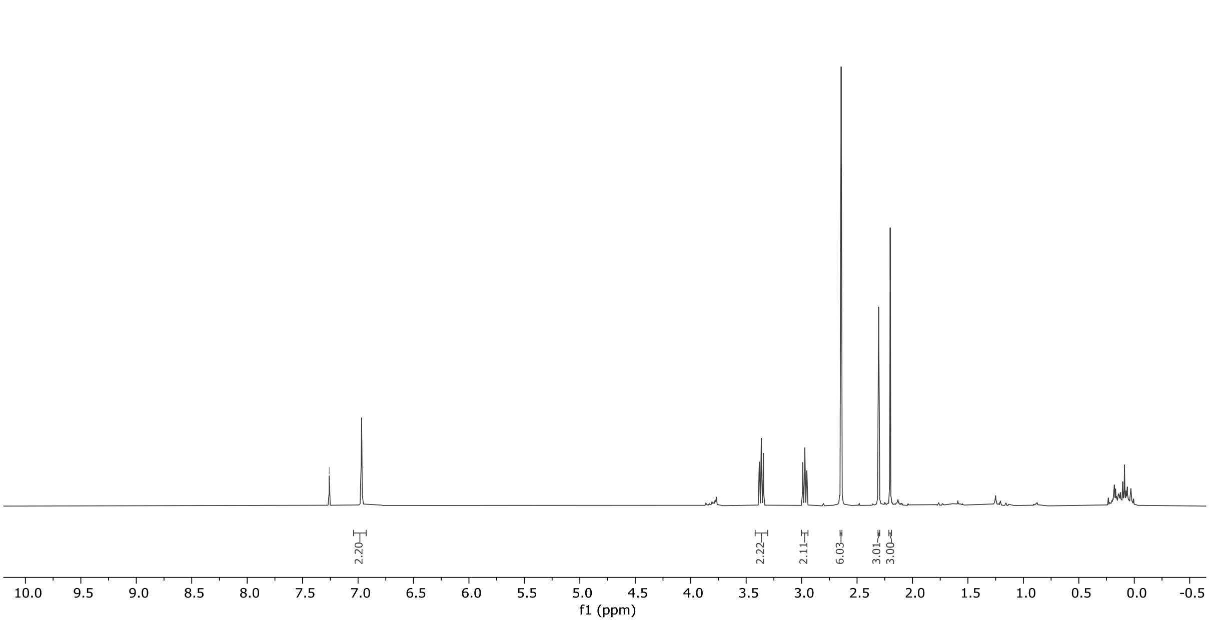

Compound **2d**
^{13}C NMR
(101 MHz, $CDCl_3$)

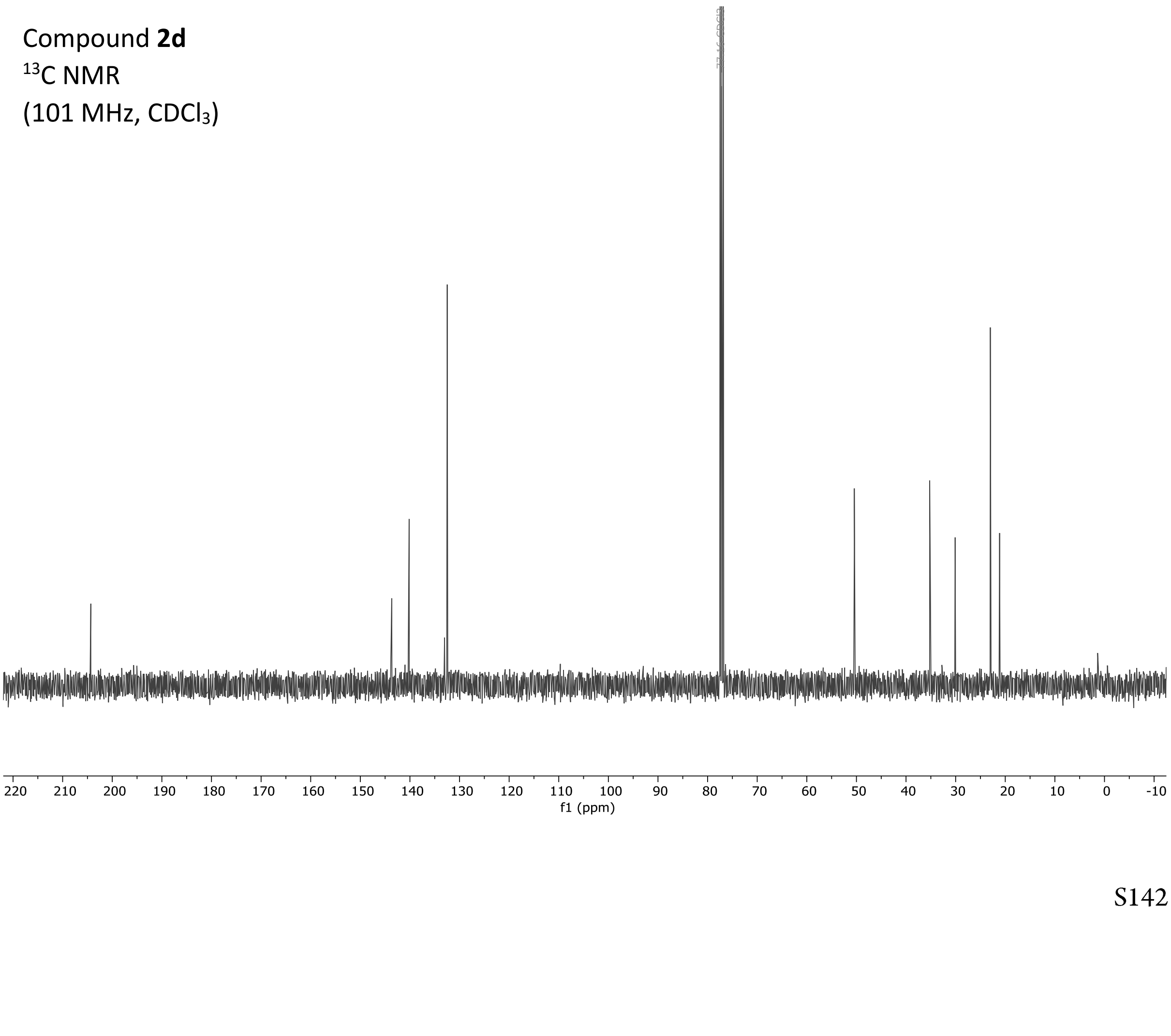

Compound **2e**
^{1}H NMR
(400 MHz, $CDCl_3$)

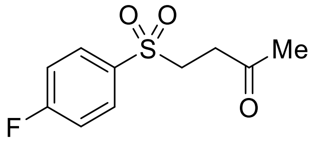

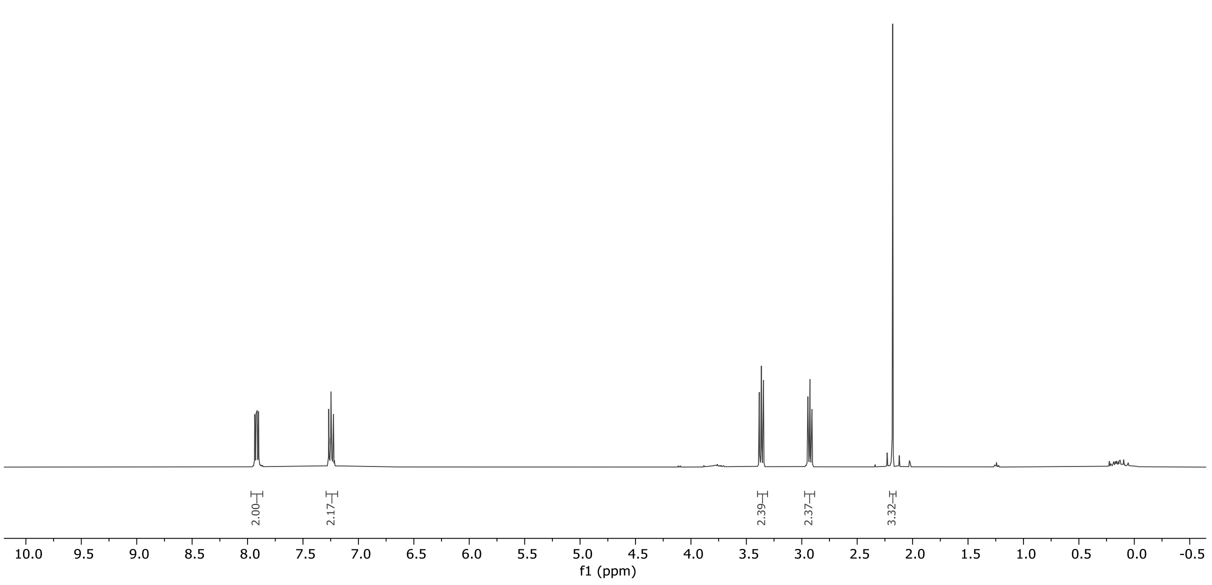

Compound **2e**
^{13}C NMR
(101 MHz, $CDCl_3$)

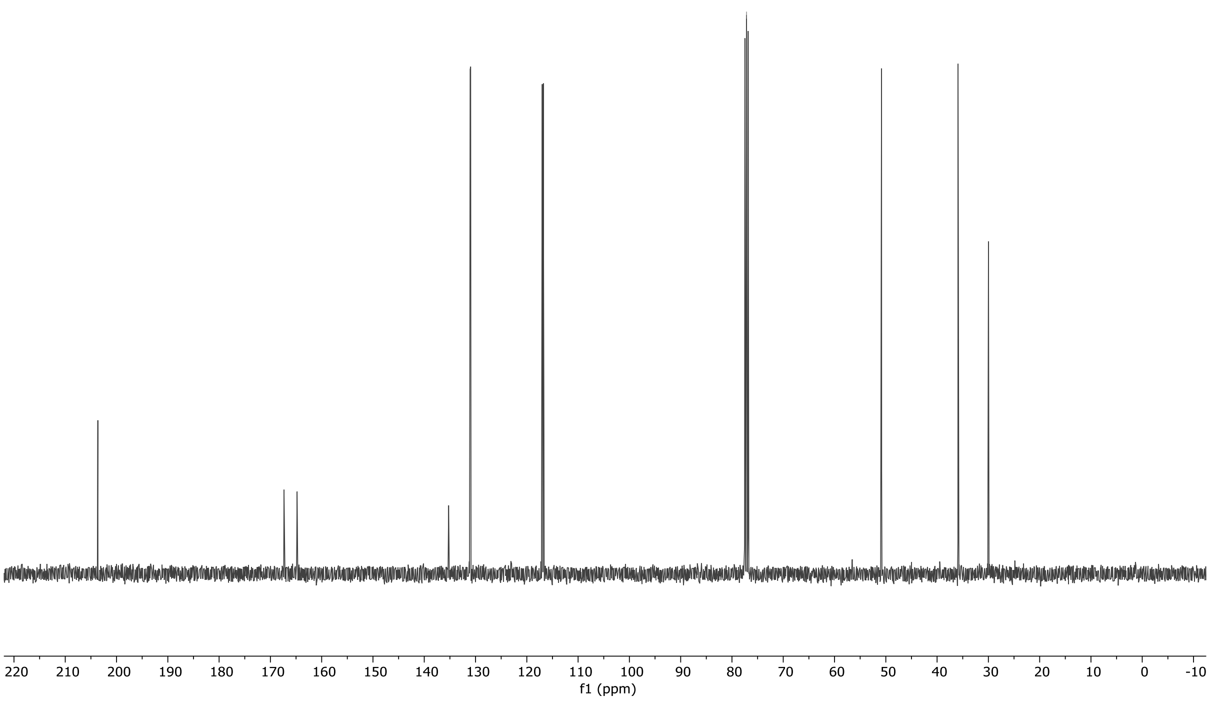

Compound **2e**
^{19}F NMR
(377 MHz, $CDCl_3$)

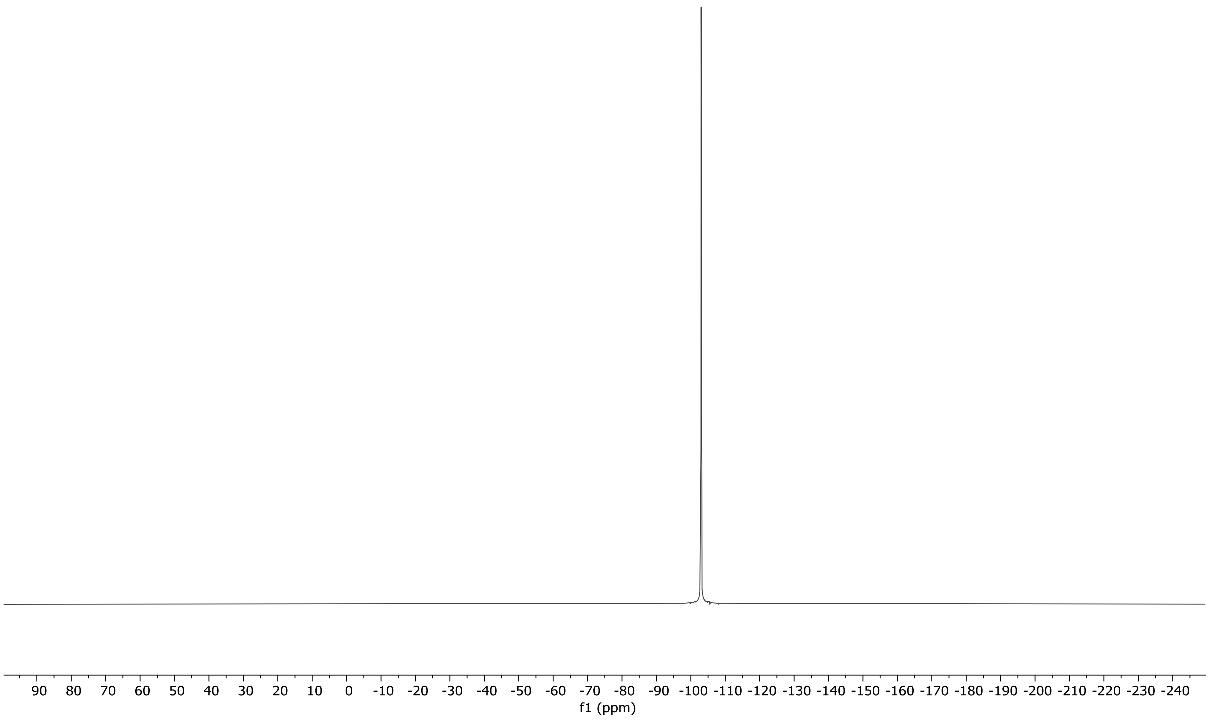

Compound **2f**
^{1}H NMR
(400 MHz, $CDCl_3$)

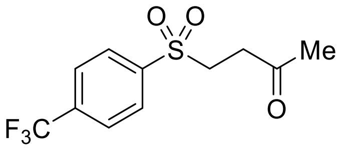

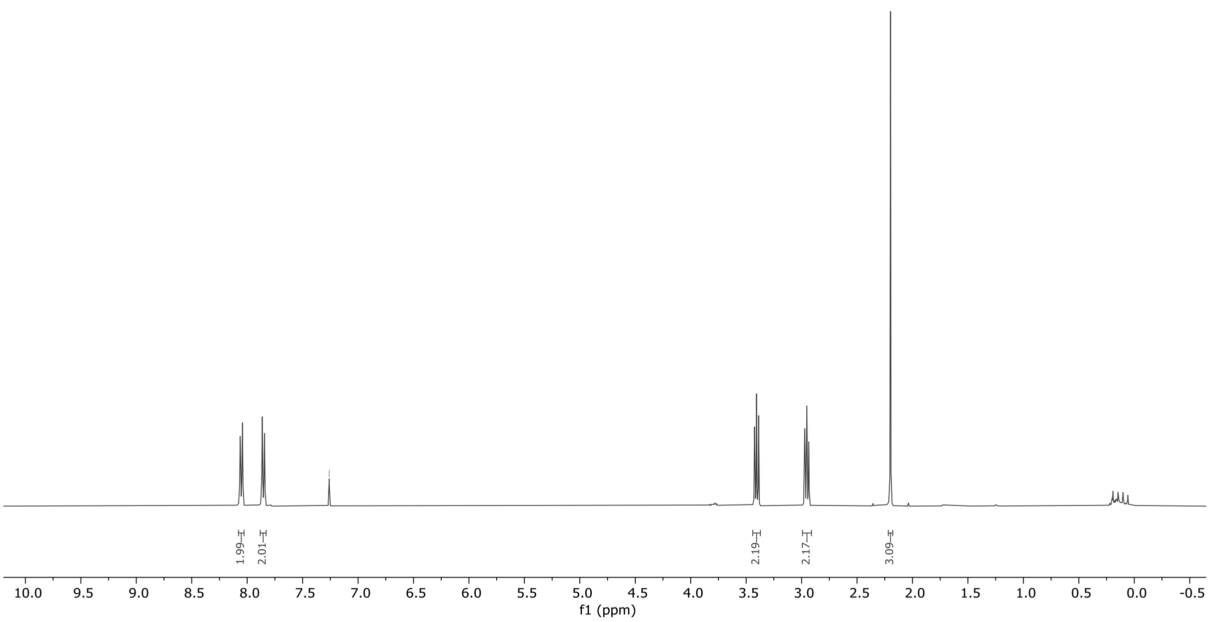

Compound **2f**
^{13}C NMR
(101 MHz, $CDCl_3$)

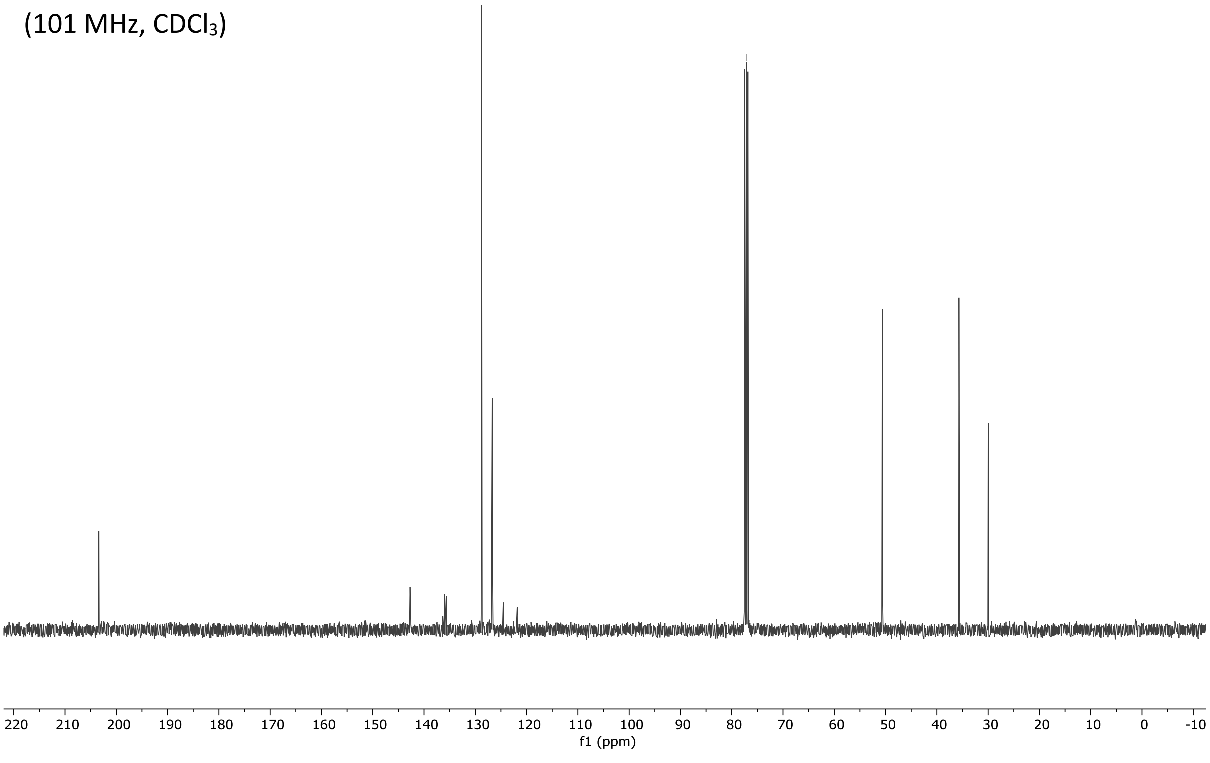

Compound **2f**
^{19}F NMR
(377 MHz, $CDCl_3$)

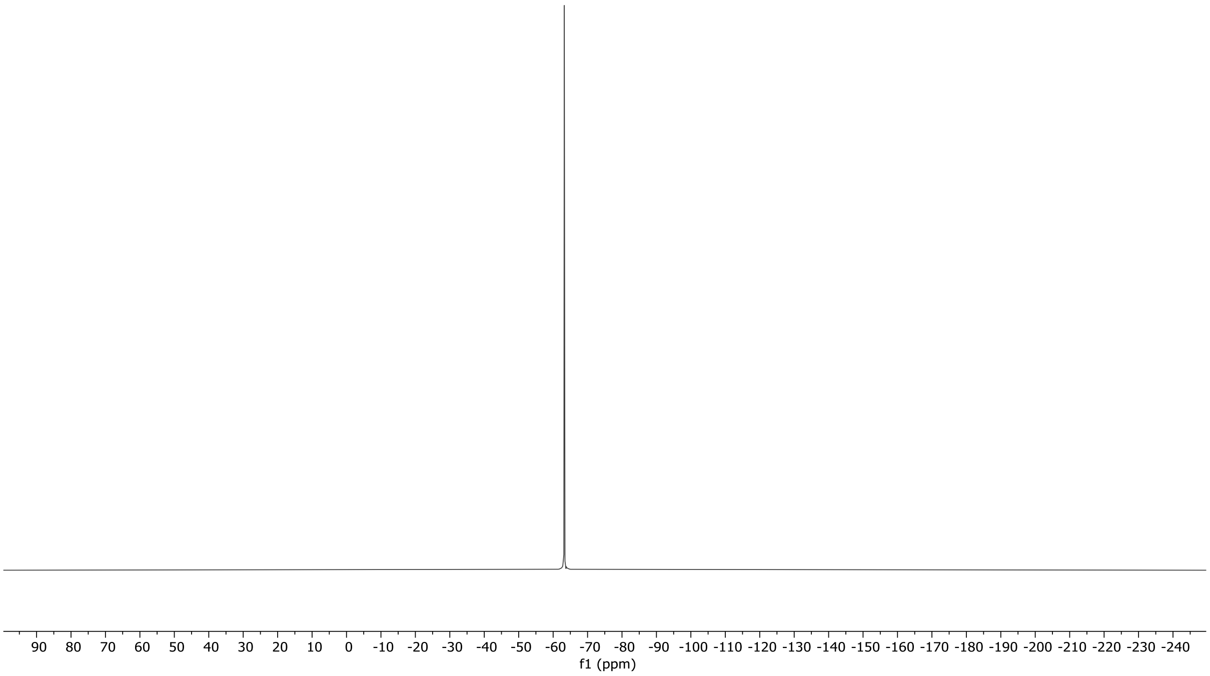

Compound **2g**
^{1}H NMR
(400 MHz, $CDCl_3$)

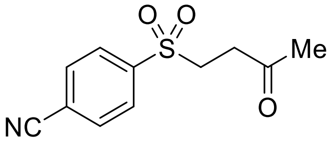

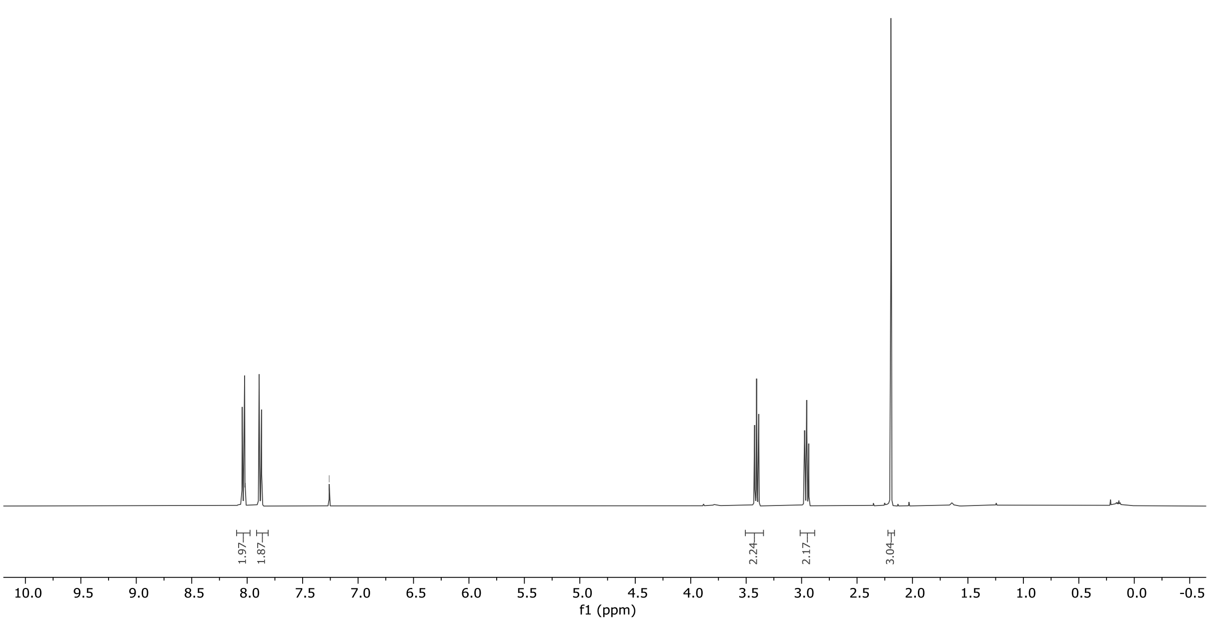

Compound **2g**
^{13}C NMR
(101 MHz, $CDCl_3$)

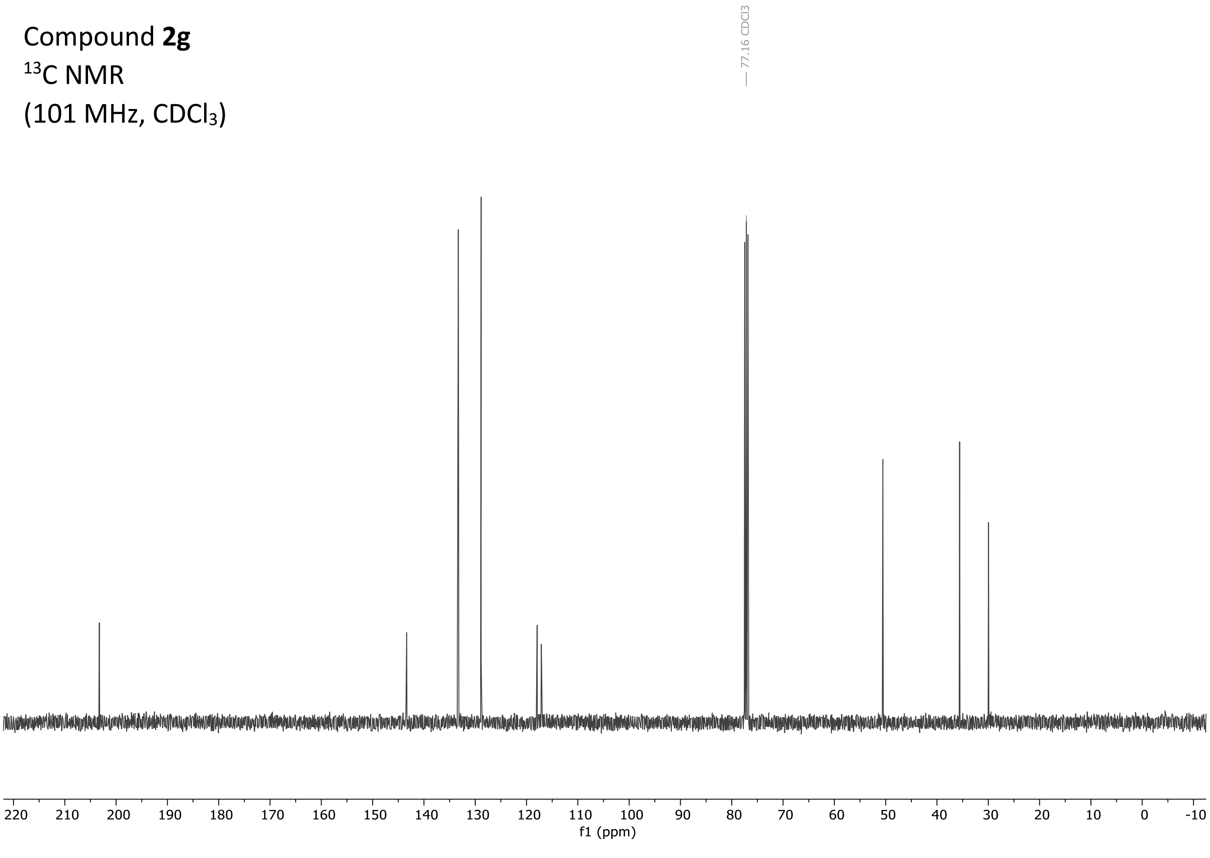

Compound **2h**
^{1}H NMR
(400 MHz, $CDCl_3$)

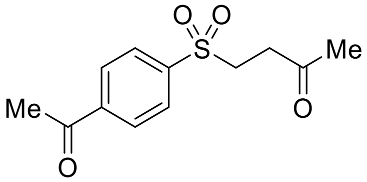

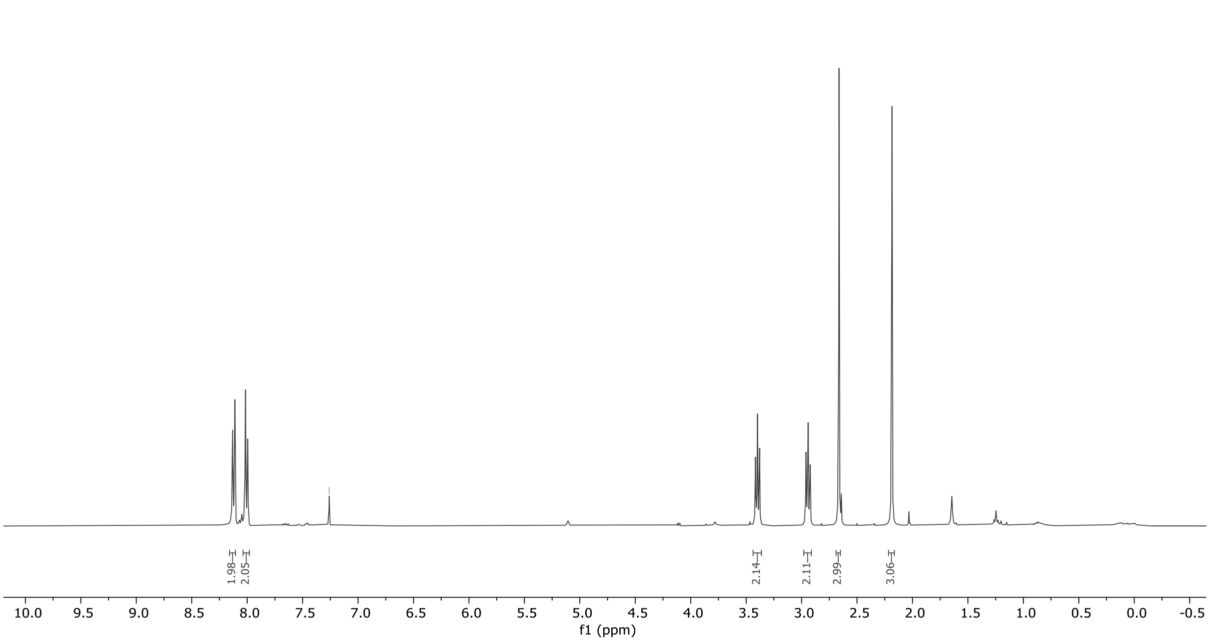

Compound **2h**
^{13}C NMR
(101 MHz, $CDCl_3$)

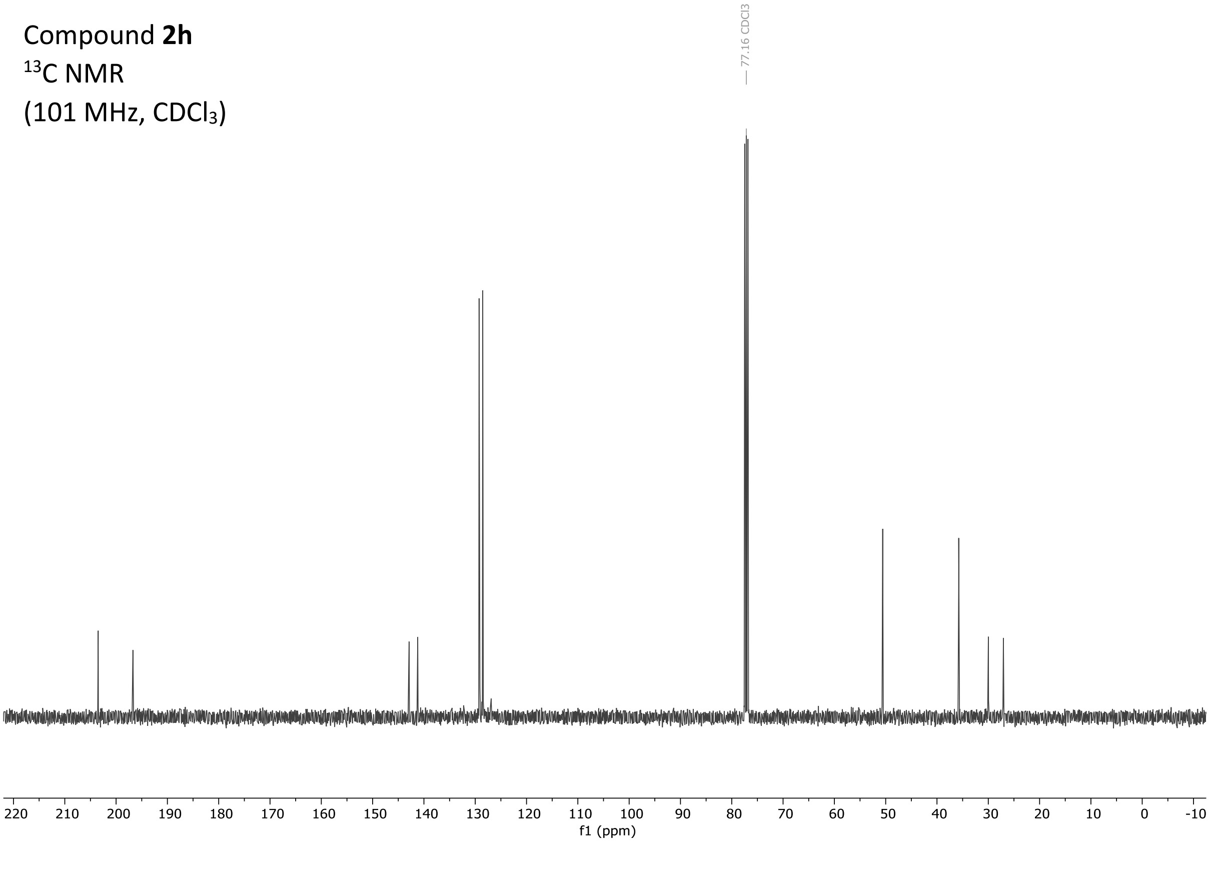

Compound **2i**
^{1}H NMR
(400 MHz, $CDCl_3$)

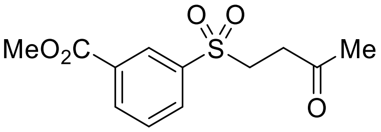

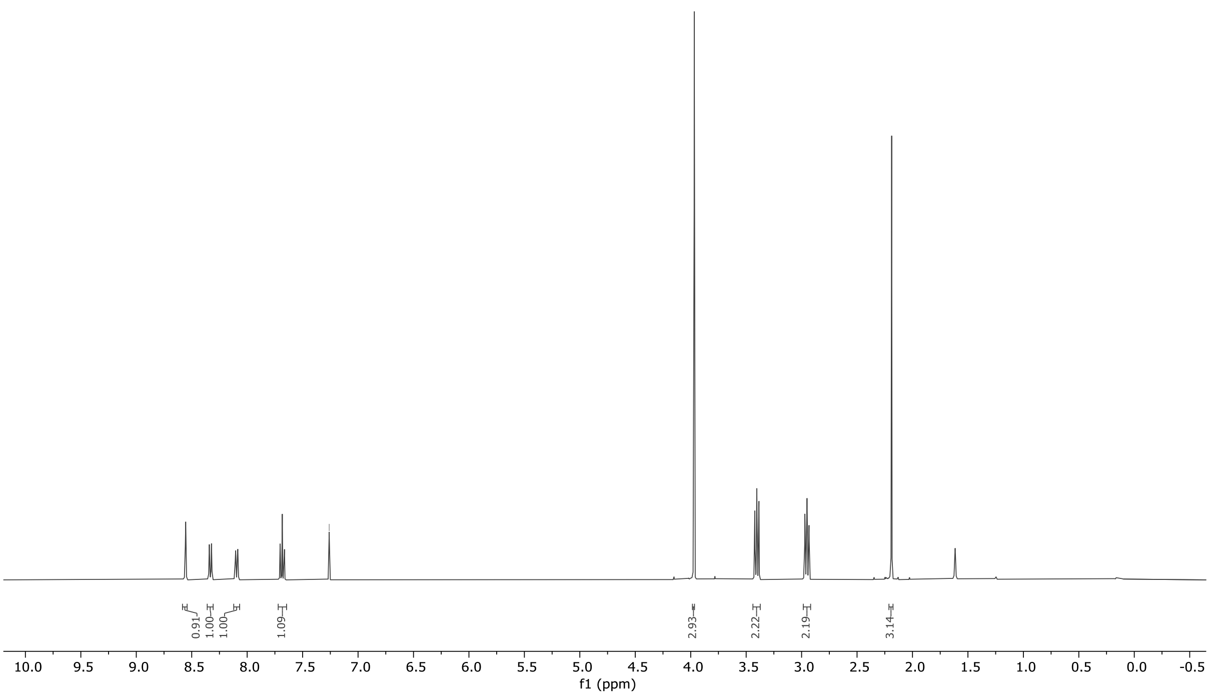

Compound **2i**
^{13}C NMR
(101 MHz, $CDCl_3$)

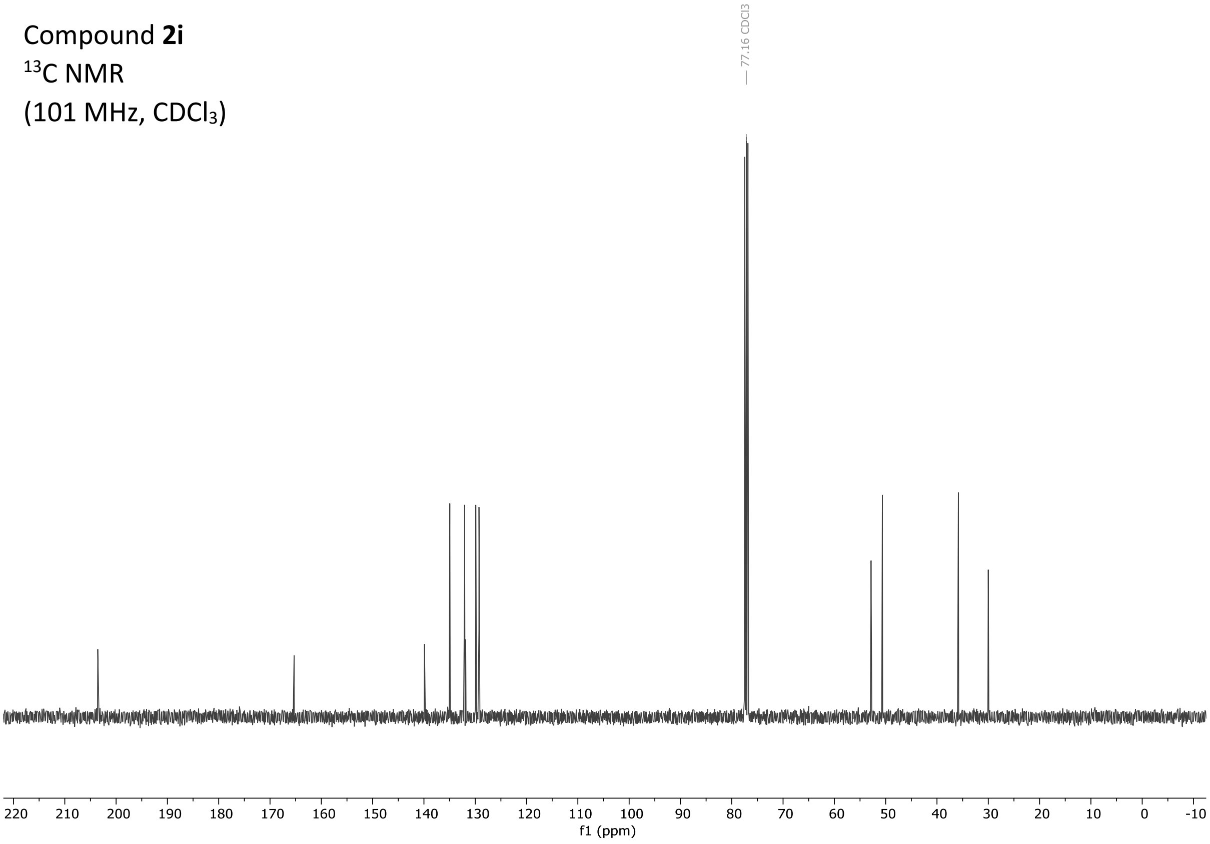

Compound **2j**
^{1}H NMR
(400 MHz, $CDCl_3$)

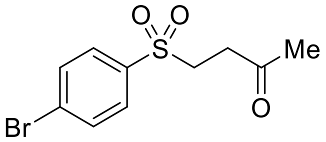

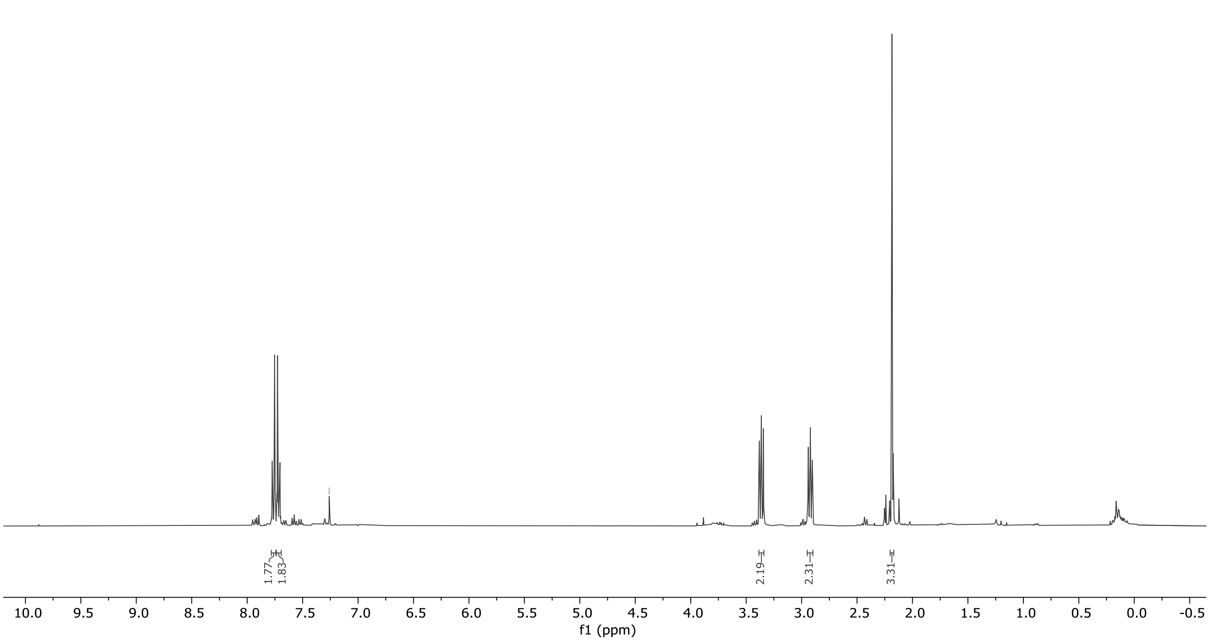

Compound **2j**
^{13}C NMR
(101 MHz, $CDCl_3$)

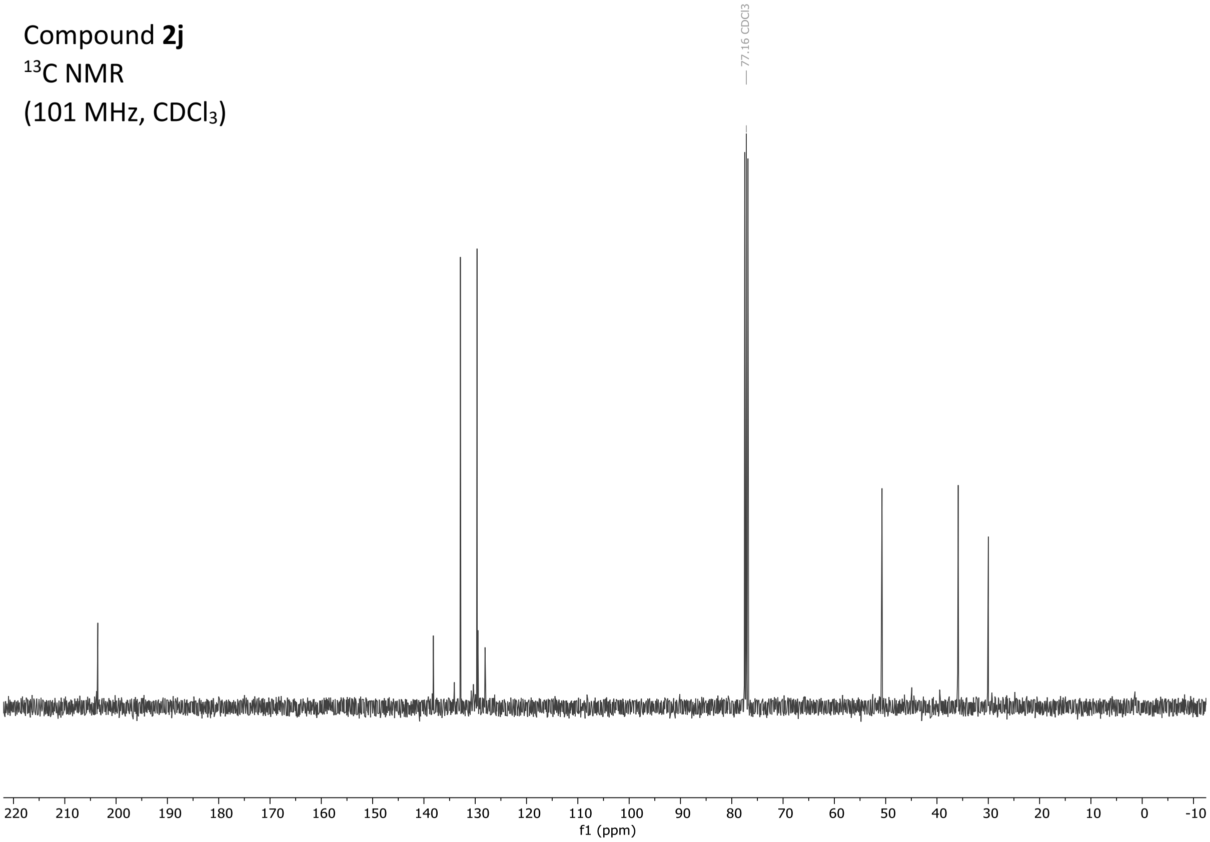

Compound **2k**
^{1}H NMR
(400 MHz, $CDCl_3$)

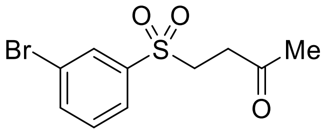

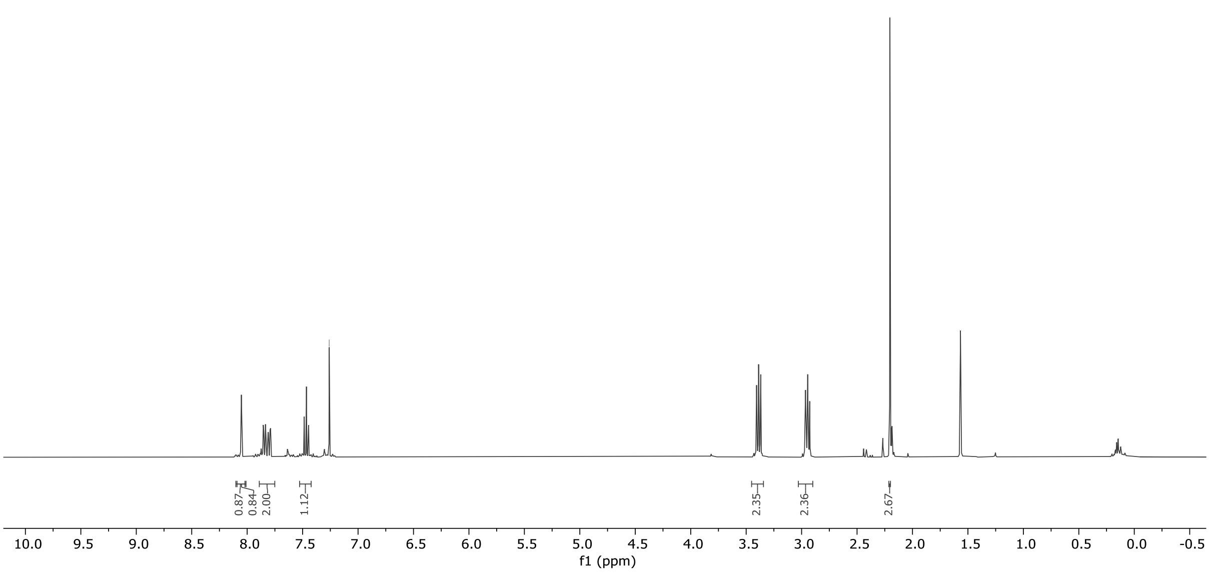

Compound **2k**
^{13}C NMR
(101 MHz, $CDCl_3$)

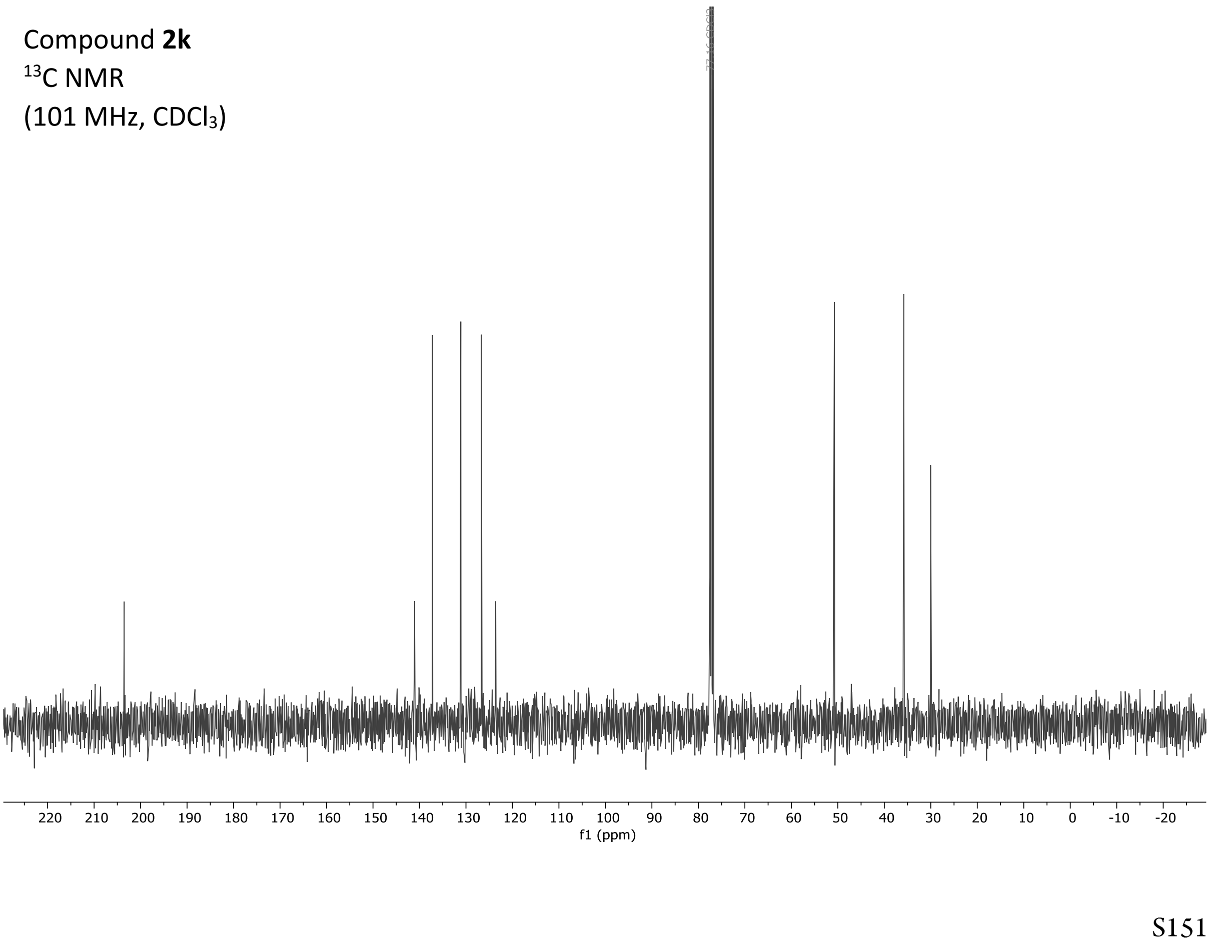

Compound **2l**
^{1}H NMR
(400 MHz, $CDCl_3$)

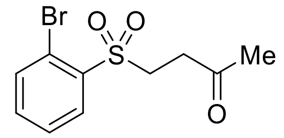

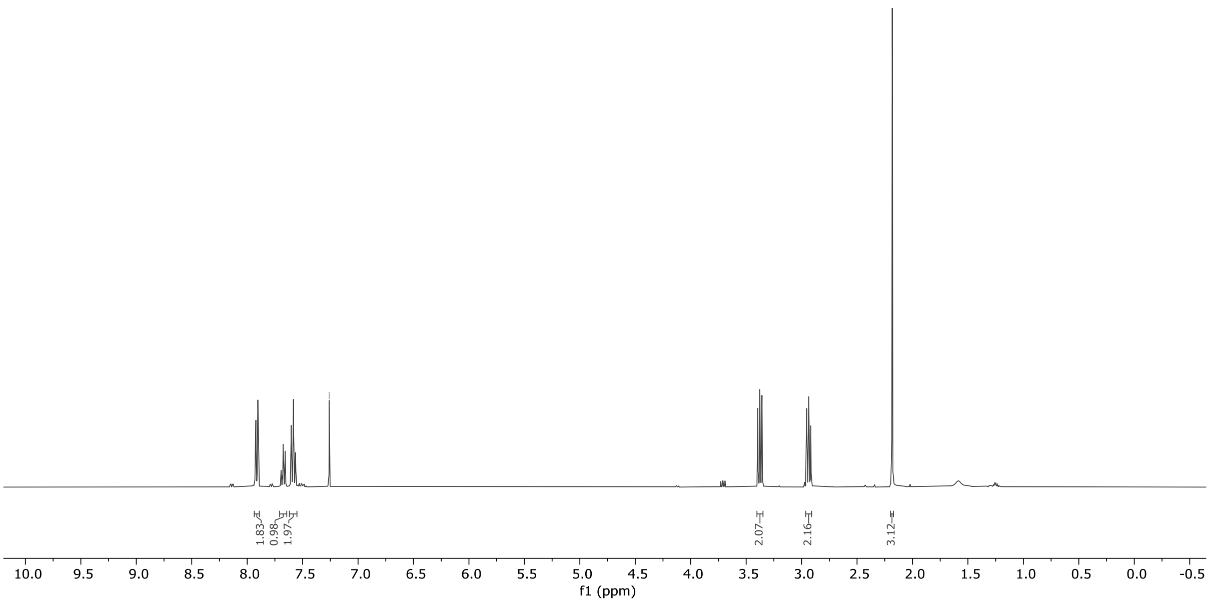

Compound **2l**
^{13}C NMR
(101 MHz, $CDCl_3$)

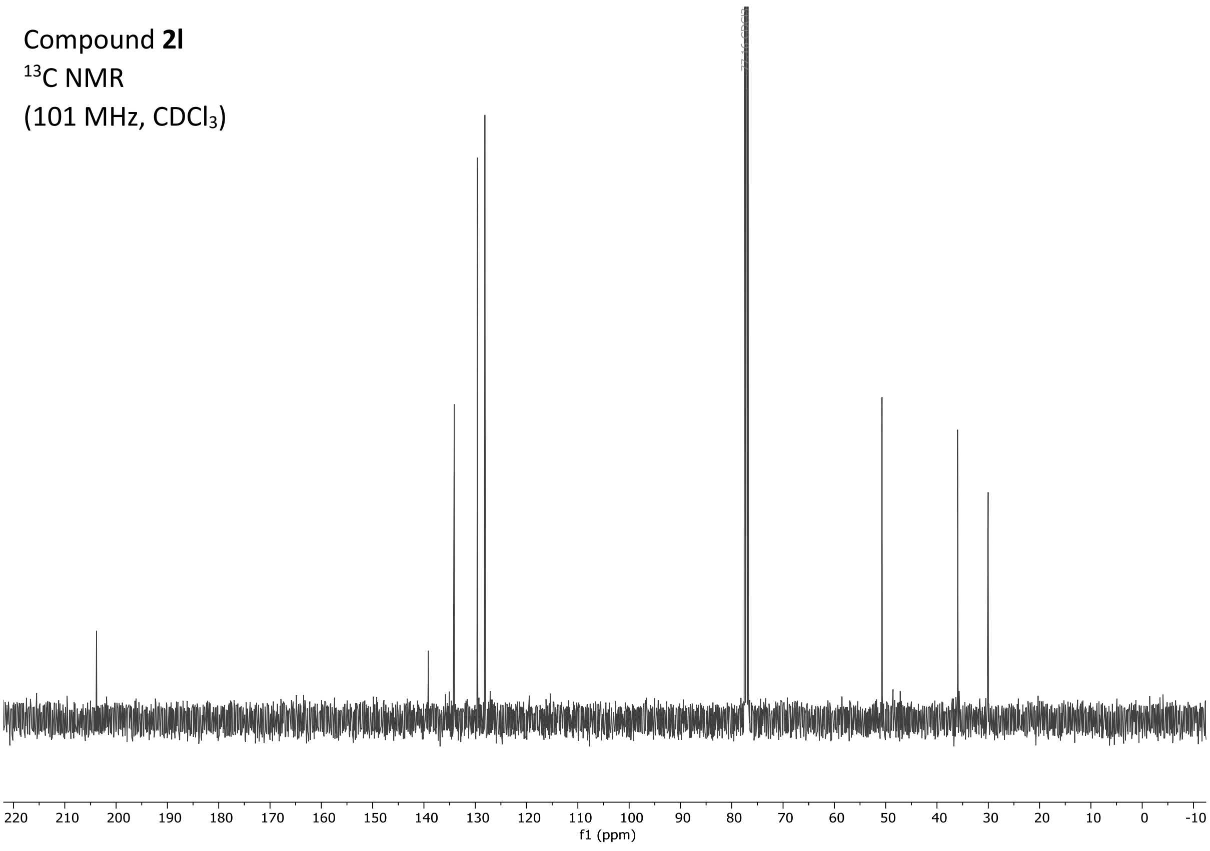

Compound **2m**
^{1}H NMR
(400 MHz, $CDCl_3$)

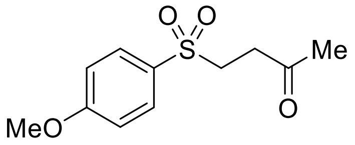

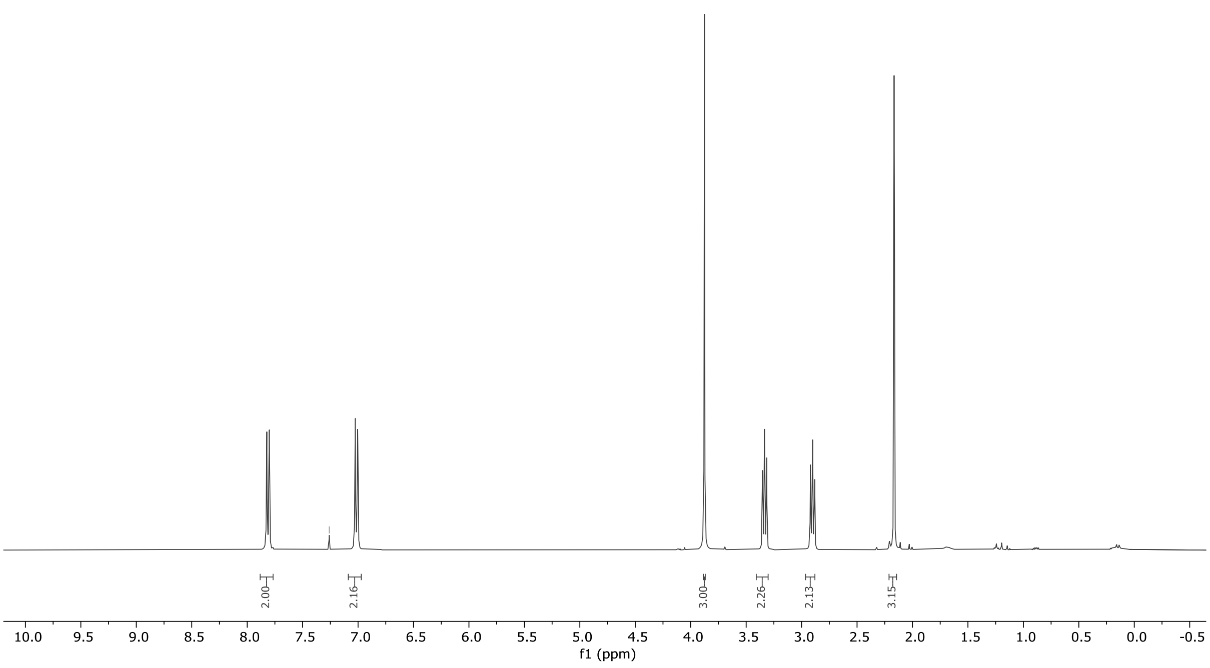

Compound **2m**
^{13}C NMR
(101 MHz, $CDCl_3$)

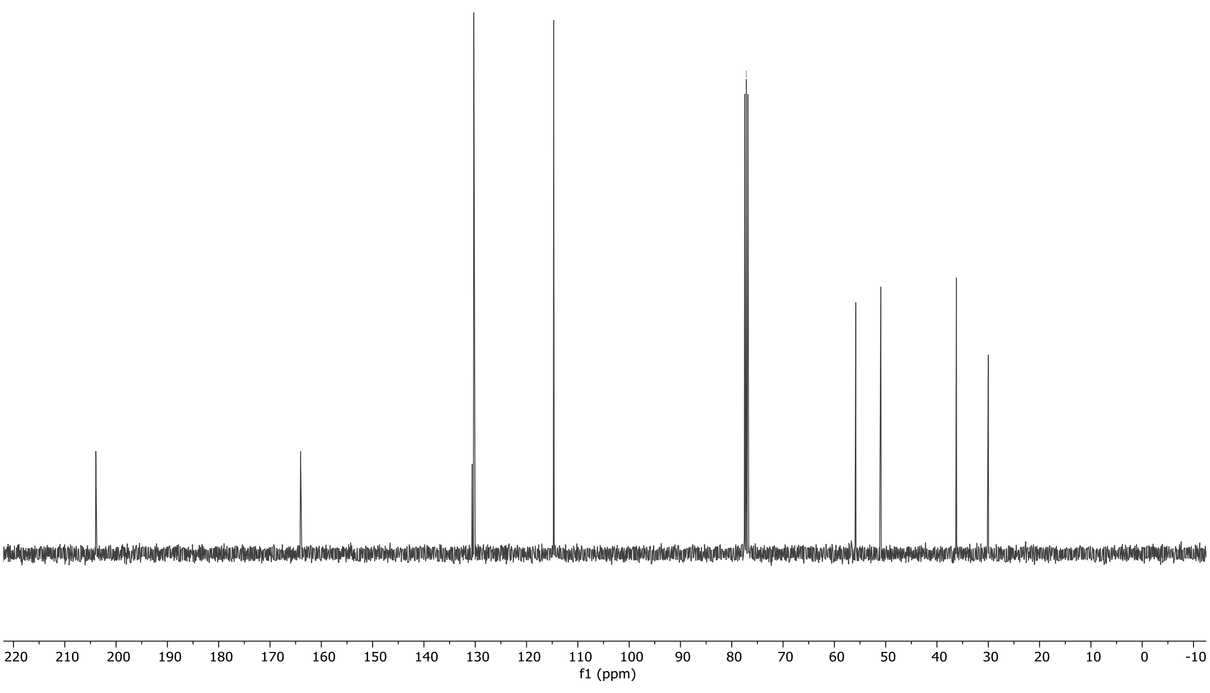

Compound **2n**
^{1}H NMR
(400 MHz, $CDCl_3$)

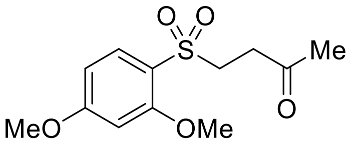

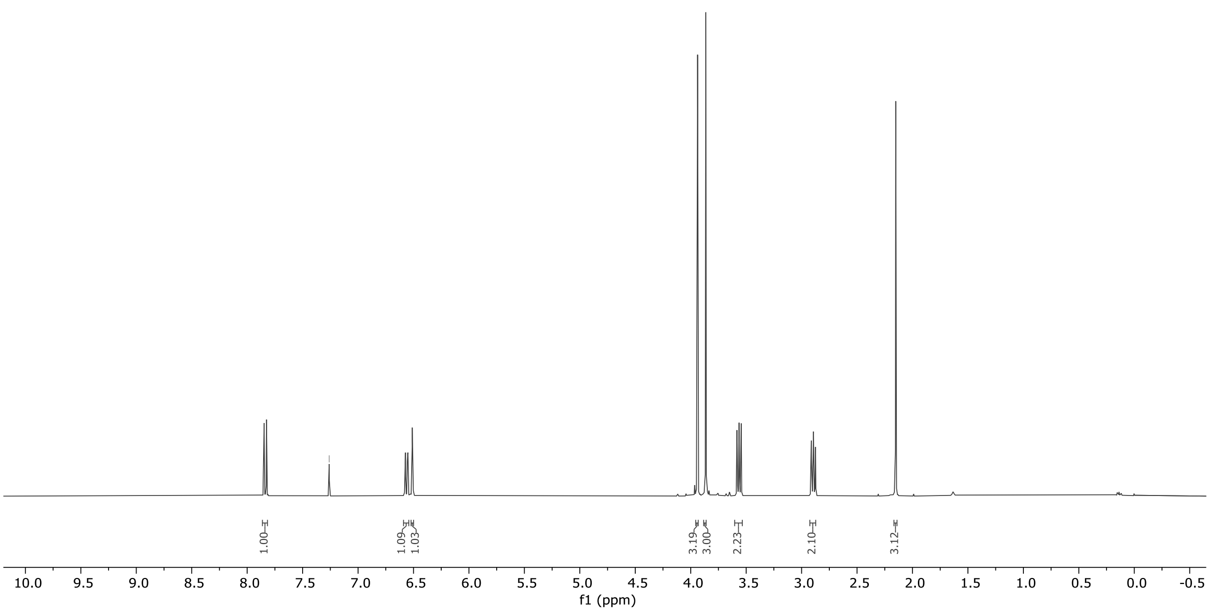

Compound **2n**
^{13}C NMR
(101 MHz, $CDCl_3$)

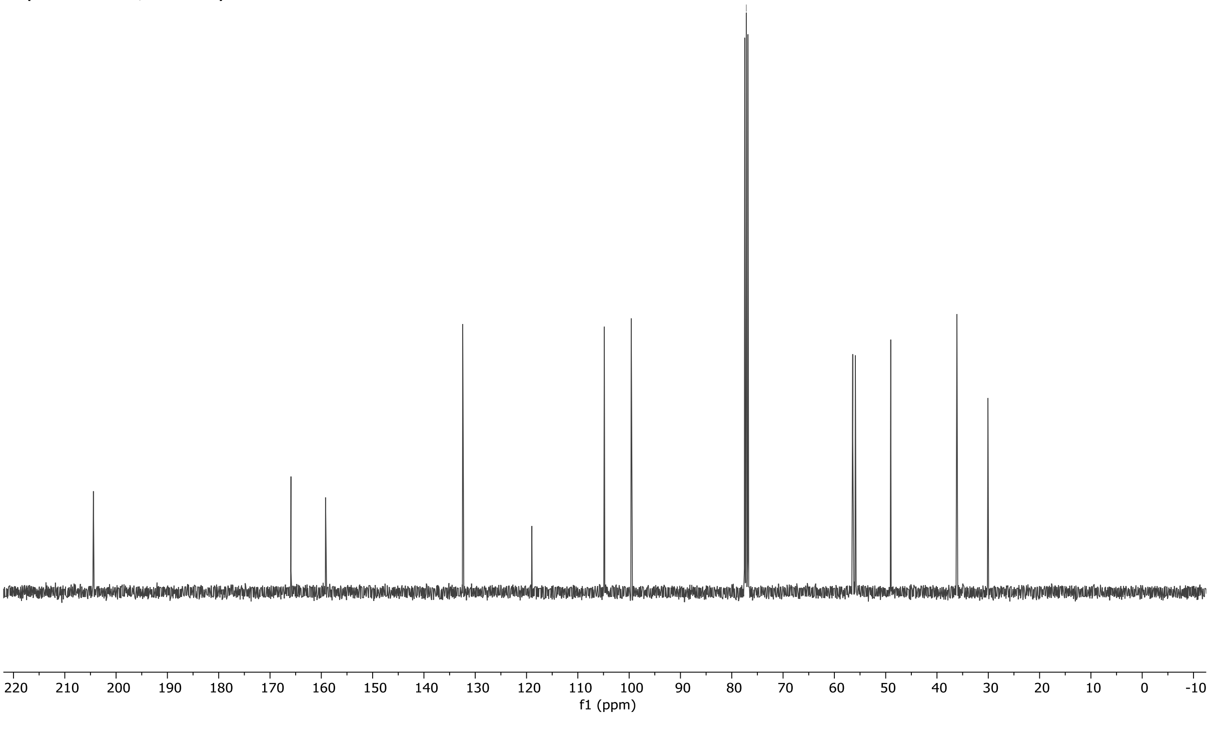

Compound **2o**
^{1}H NMR
(400 MHz, $CDCl_3$)

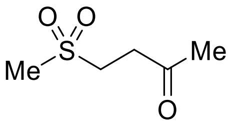

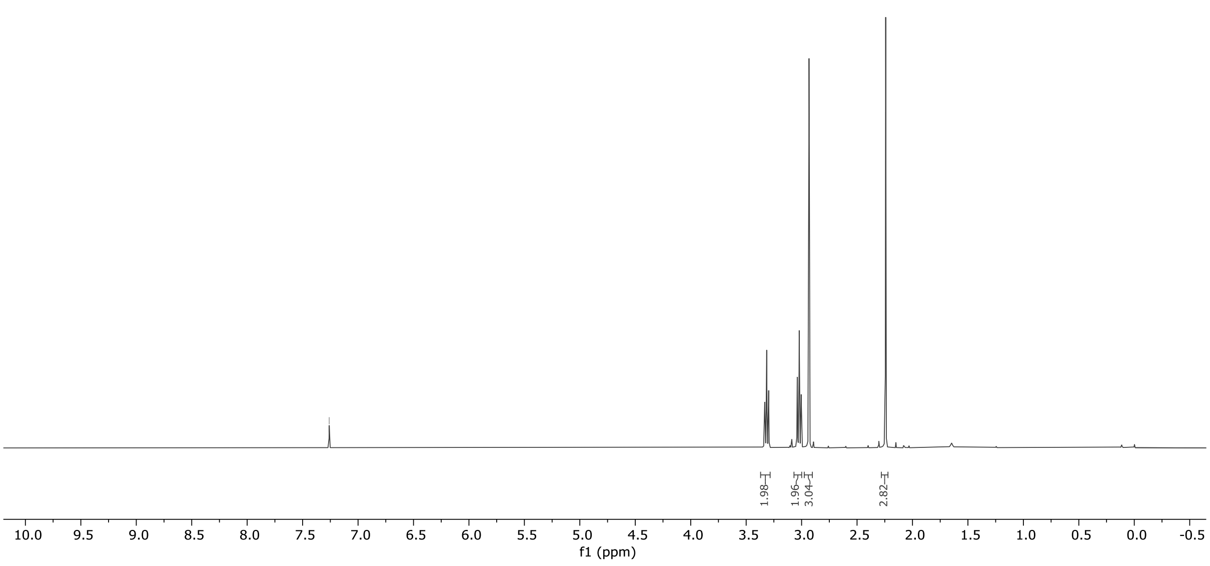

Compound **2o**
^{13}C NMR
(101 MHz, $CDCl_3$)

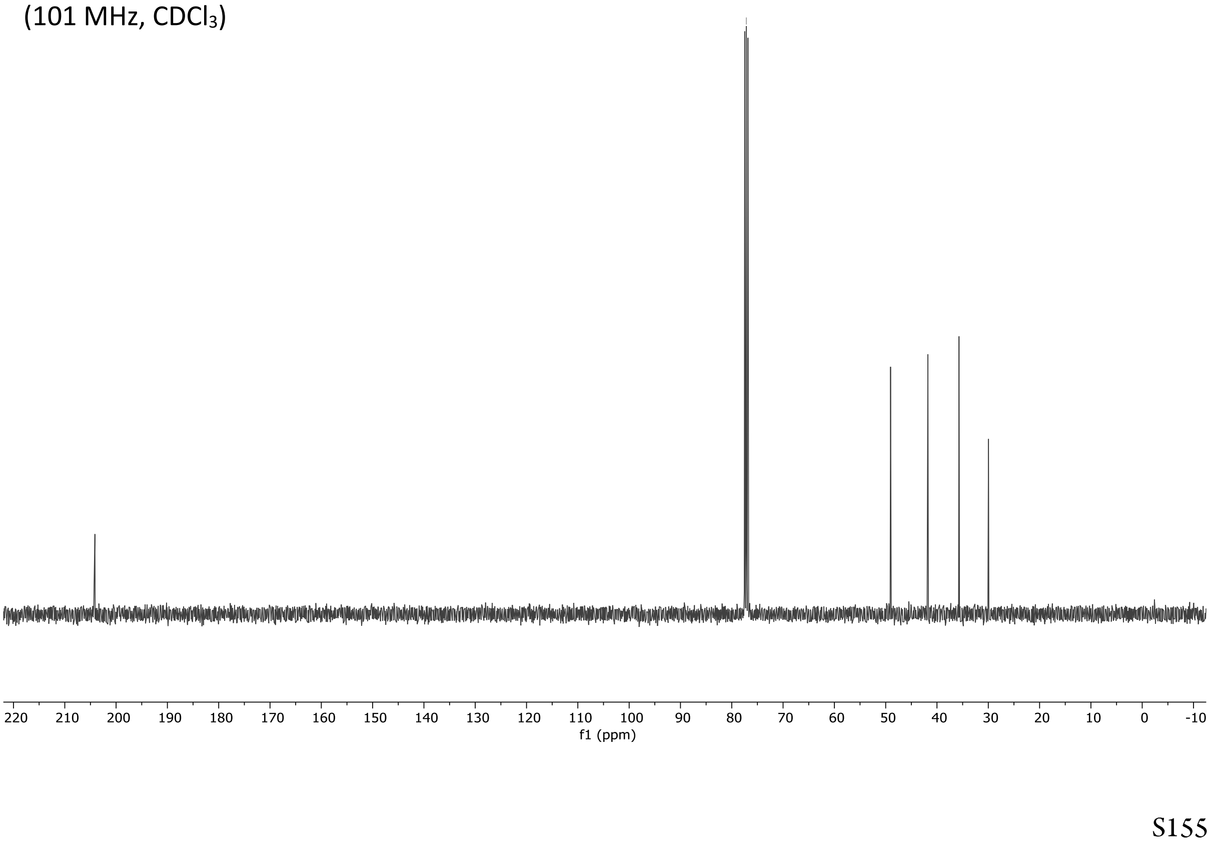

Compound **2p**
^{1}H NMR
(400 MHz, $CDCl_3$)

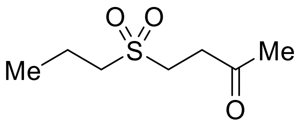

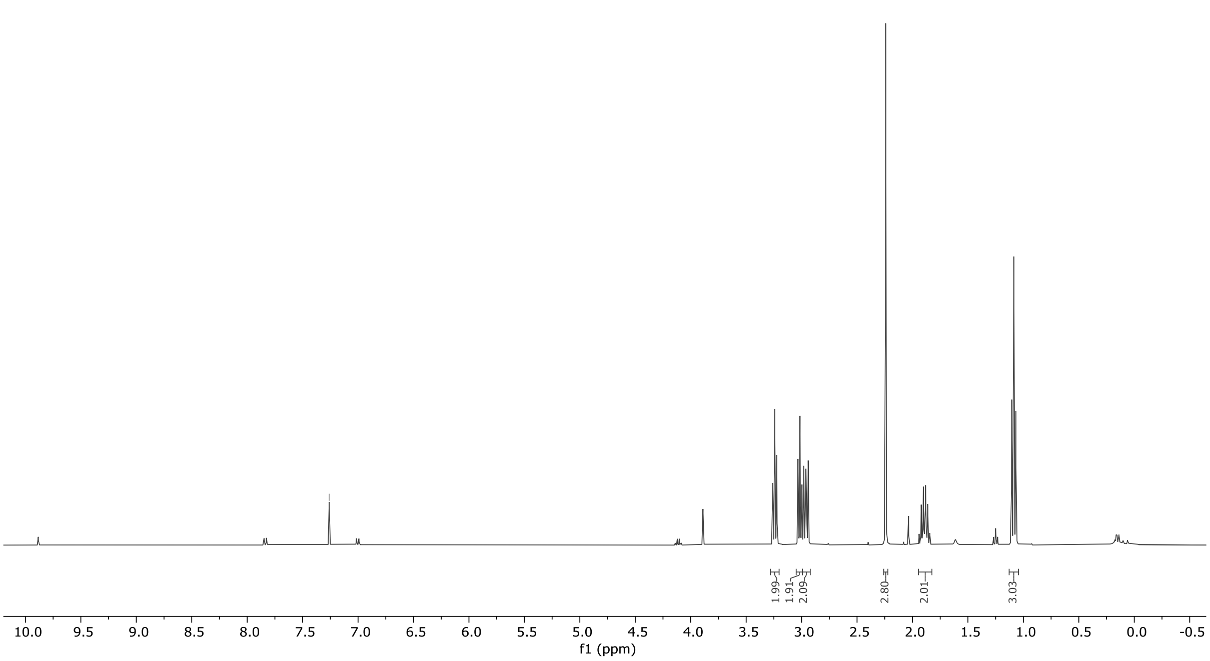

Compound **2p**
^{13}C NMR
(101 MHz, $CDCl_3$)

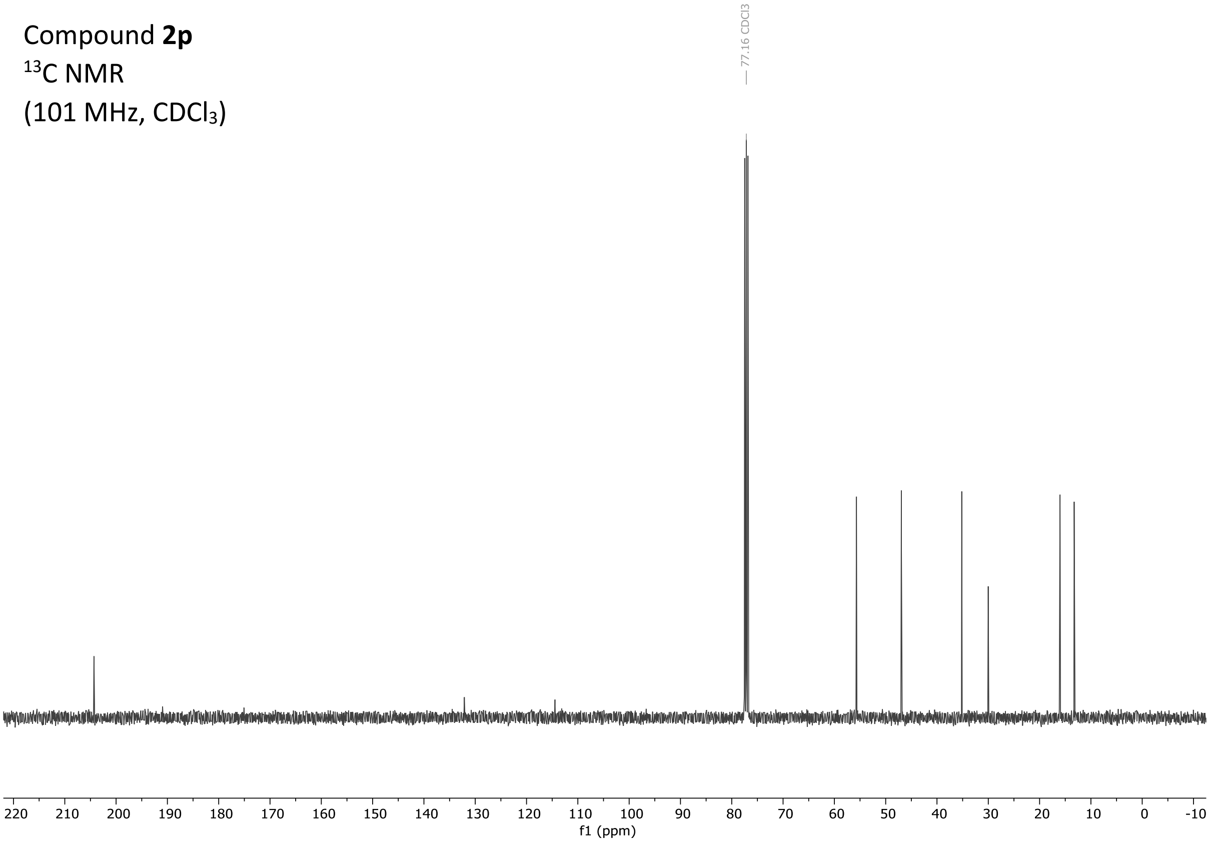

Compound **2q**
^{1}H NMR
(400 MHz, $CDCl_3$)

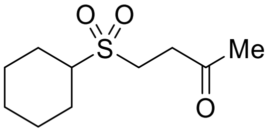

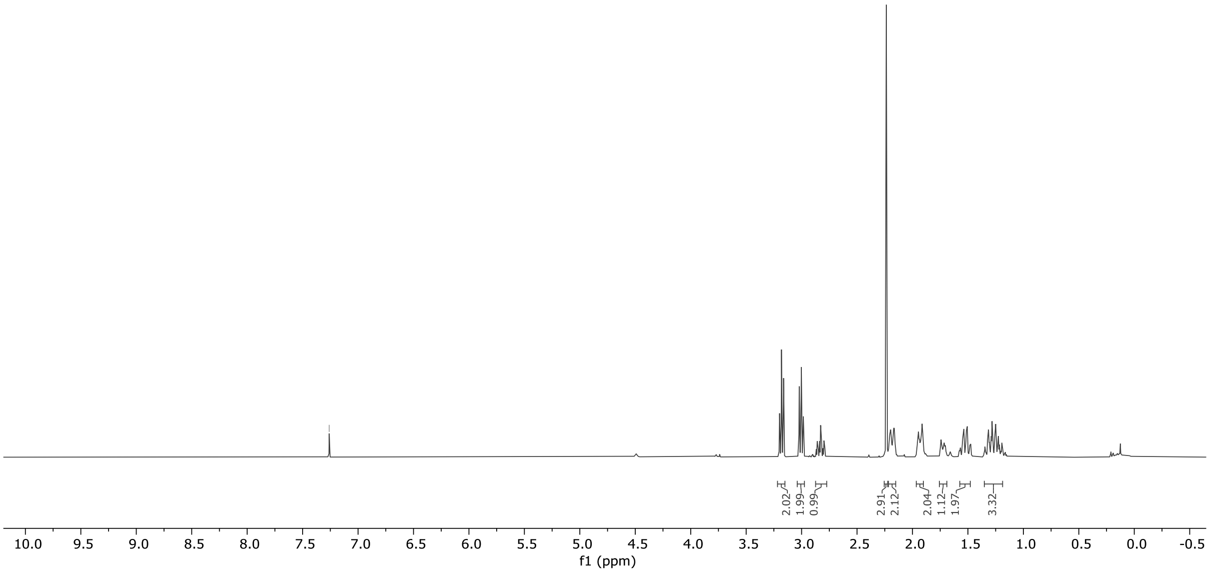

Compound **2q**
^{13}C NMR
(101 MHz, $CDCl_3$)

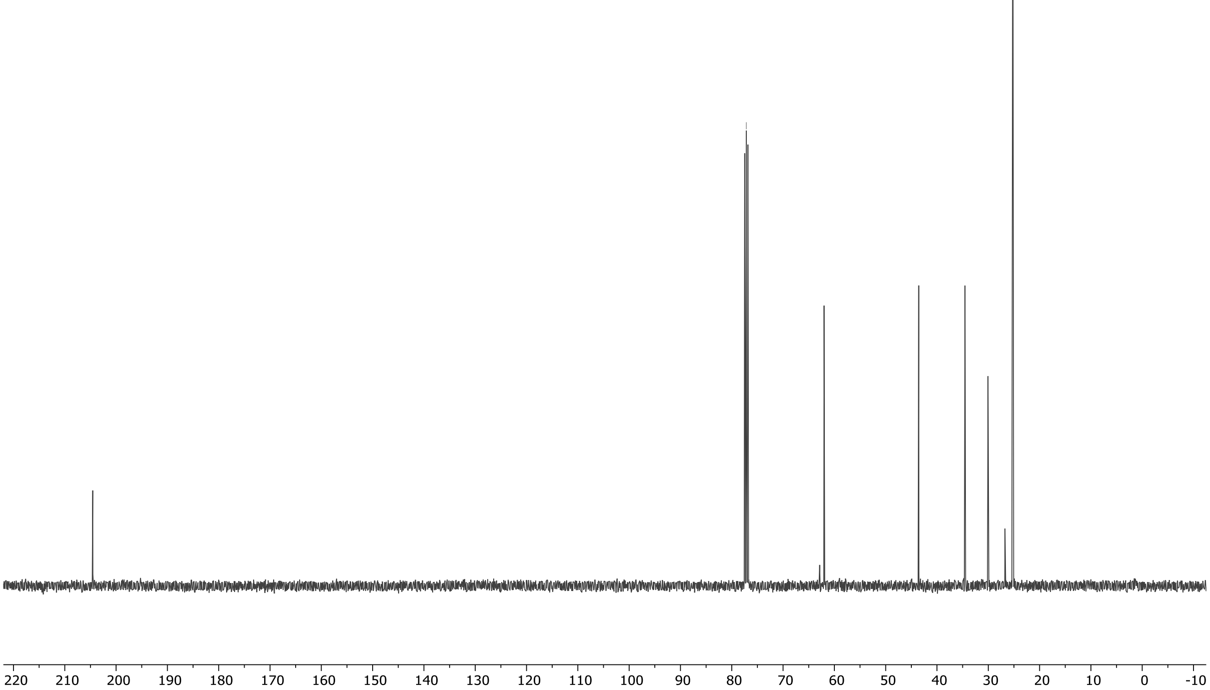

Compound **2r**
^{1}H NMR
(400 MHz, $CDCl_3$)

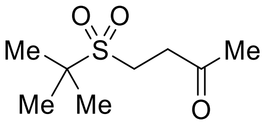

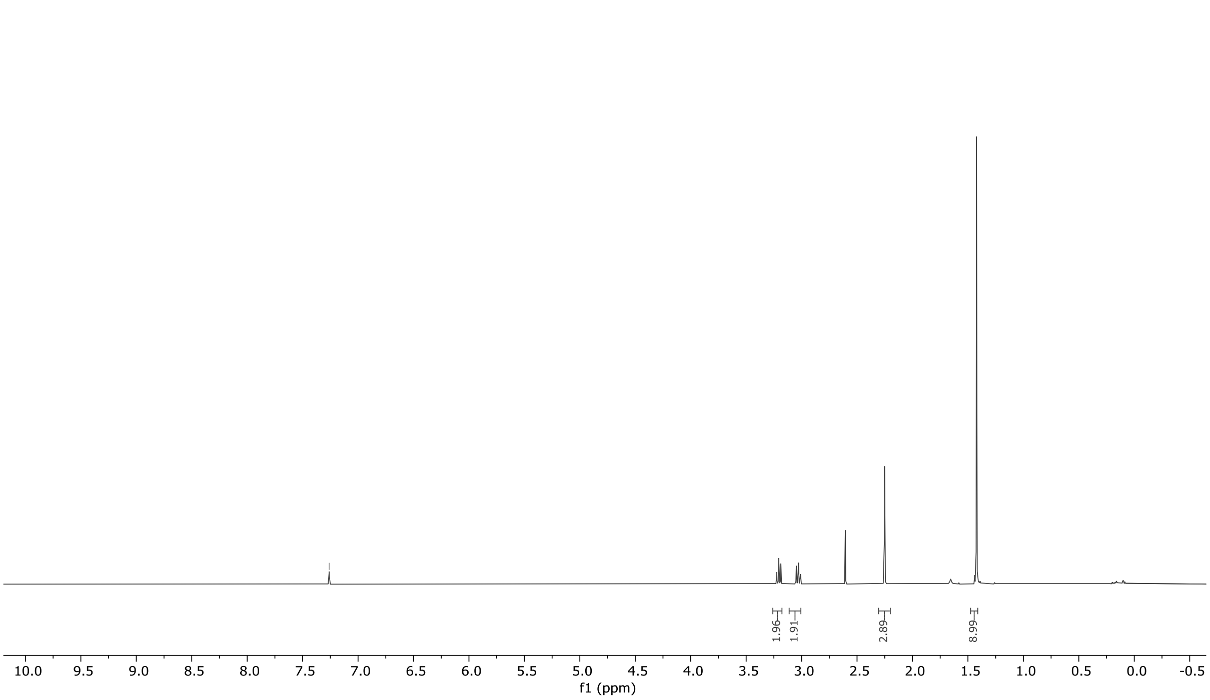

Compound **2r**
^{13}C NMR
(101 MHz, $CDCl_3$)

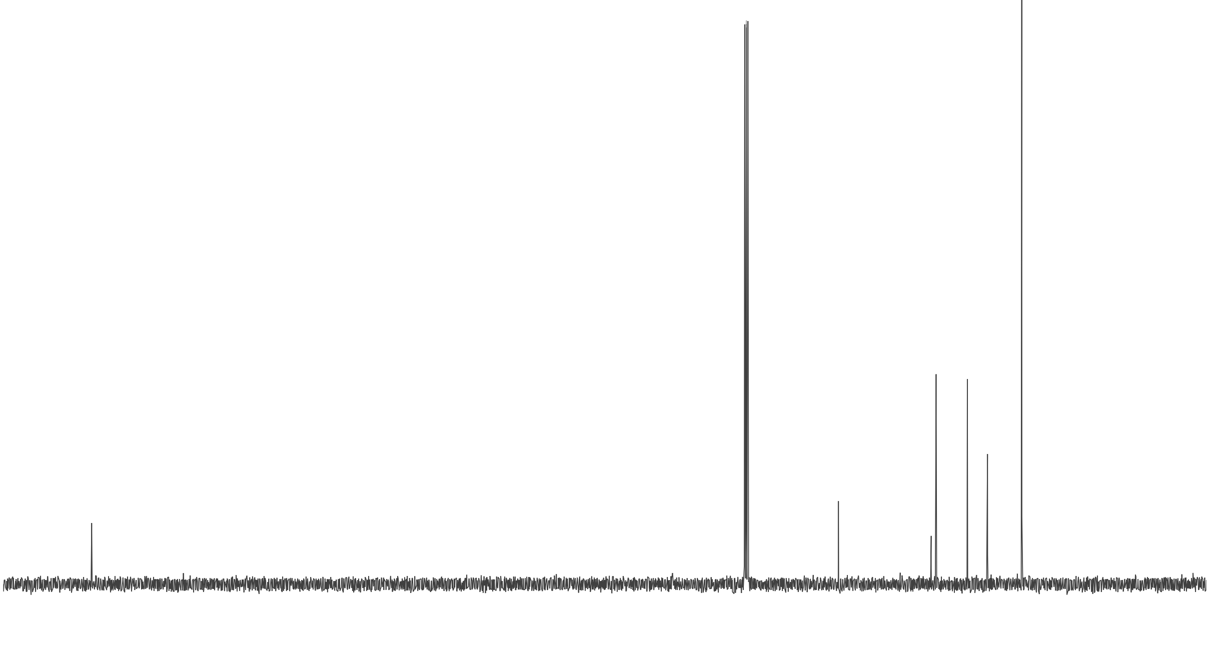

Compound **2s**
^{1}H NMR
(400 MHz, $CDCl_3$)

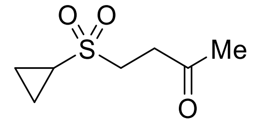

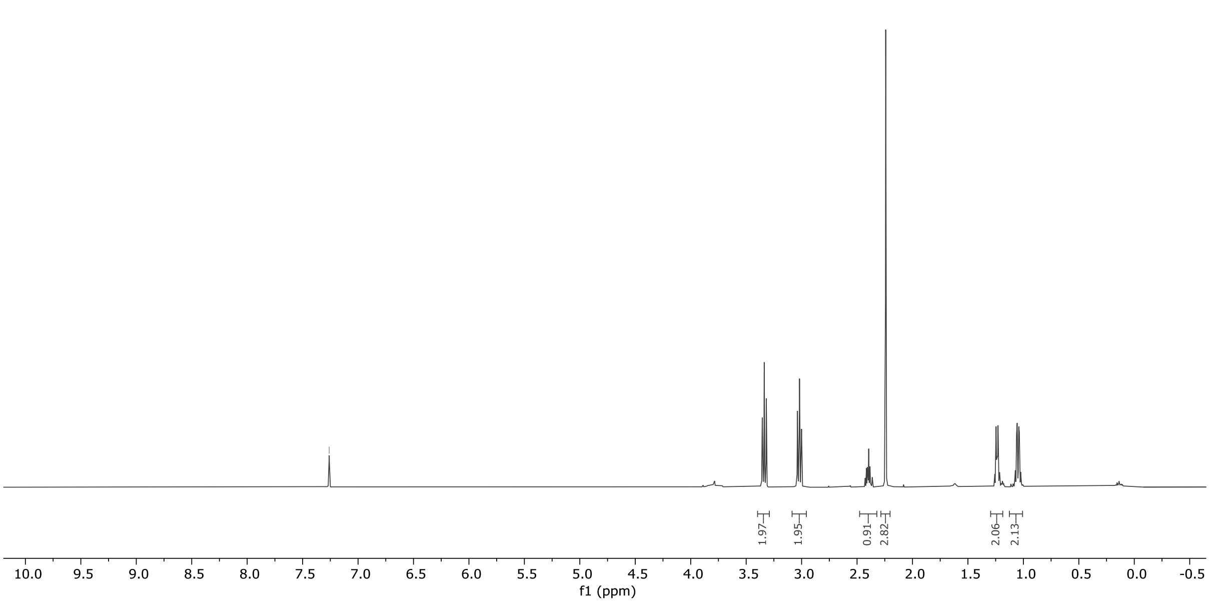

Compound **2s**
^{13}C NMR
(101 MHz, $CDCl_3$)

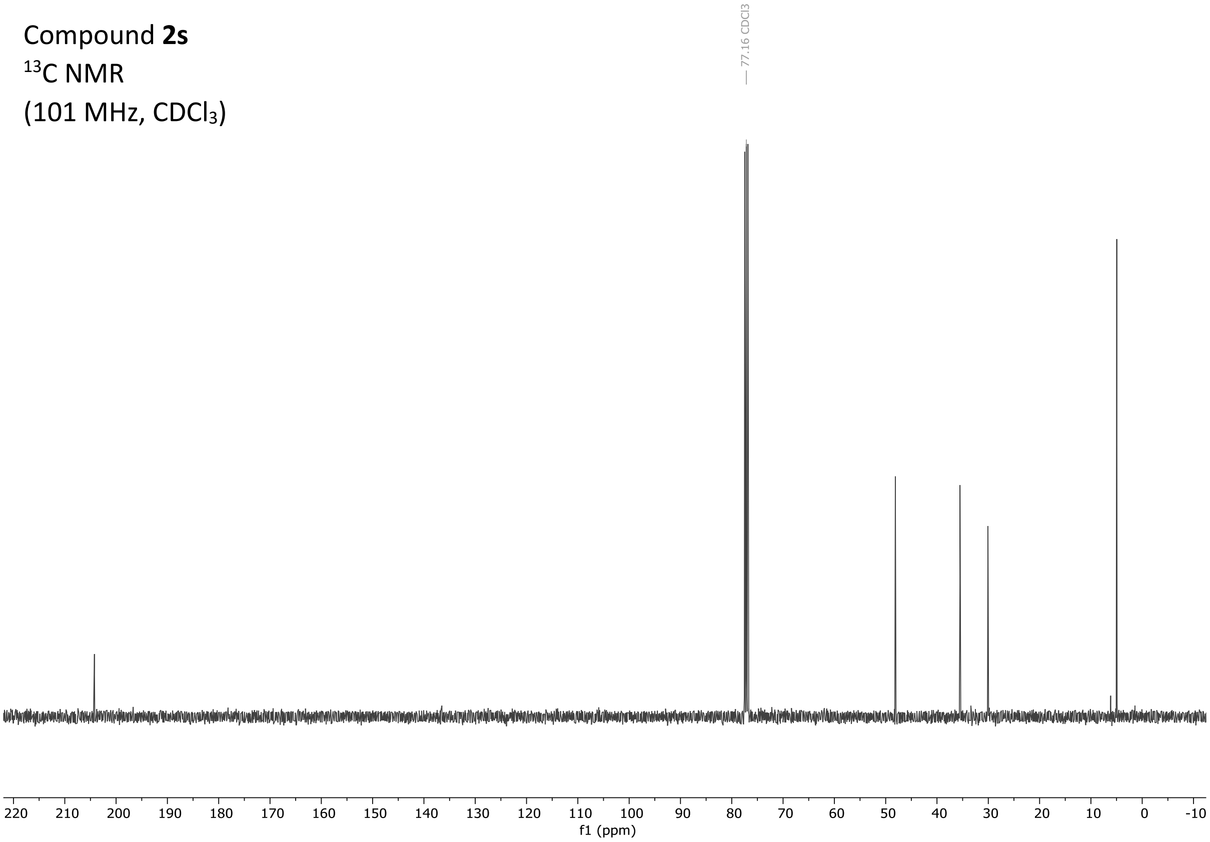

Compound **2t**
^{1}H NMR
(400 MHz, $CDCl_3$)

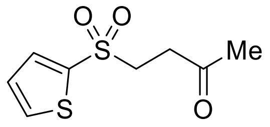

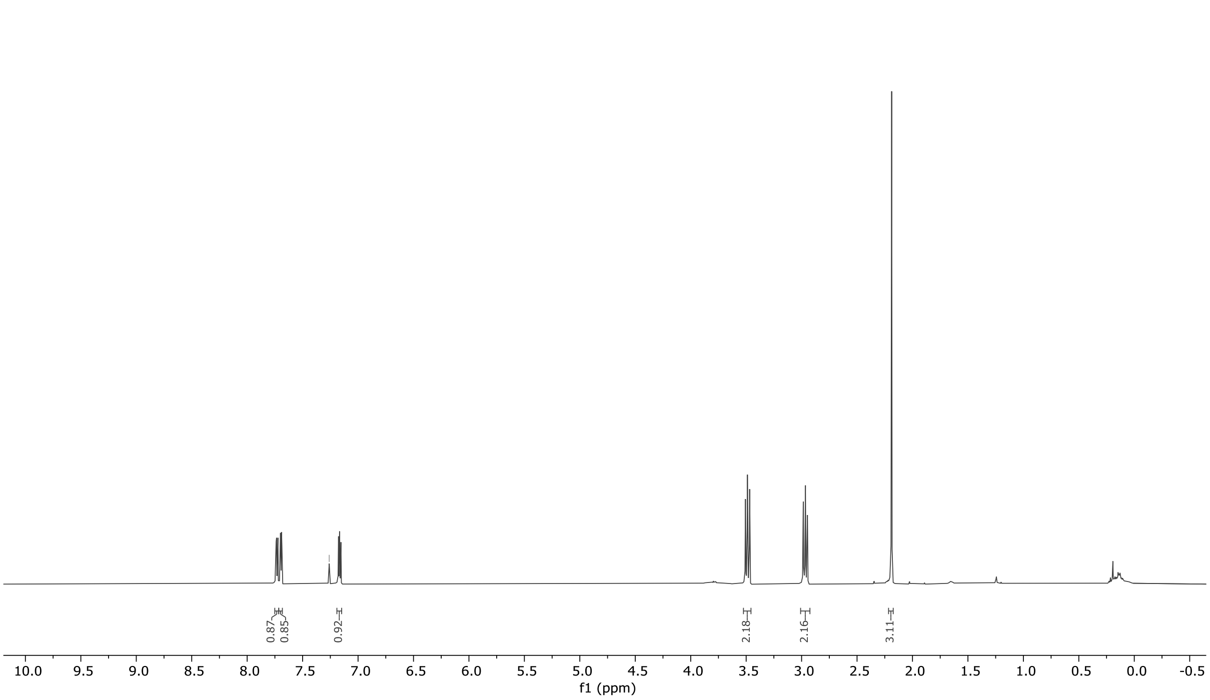

Compound **2t**
^{13}C NMR
(101 MHz, $CDCl_3$)

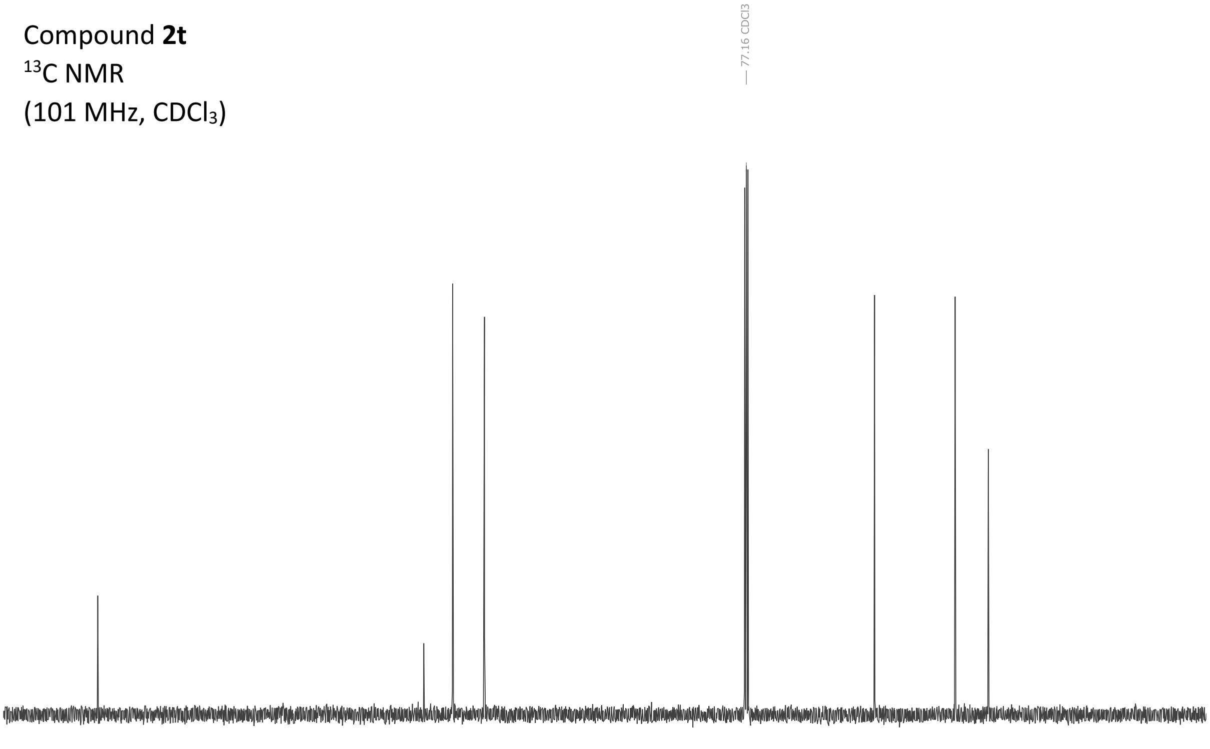

Compound **2u**
^{1}H NMR
(400 MHz, $CDCl_3$)

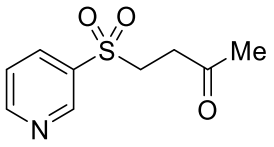

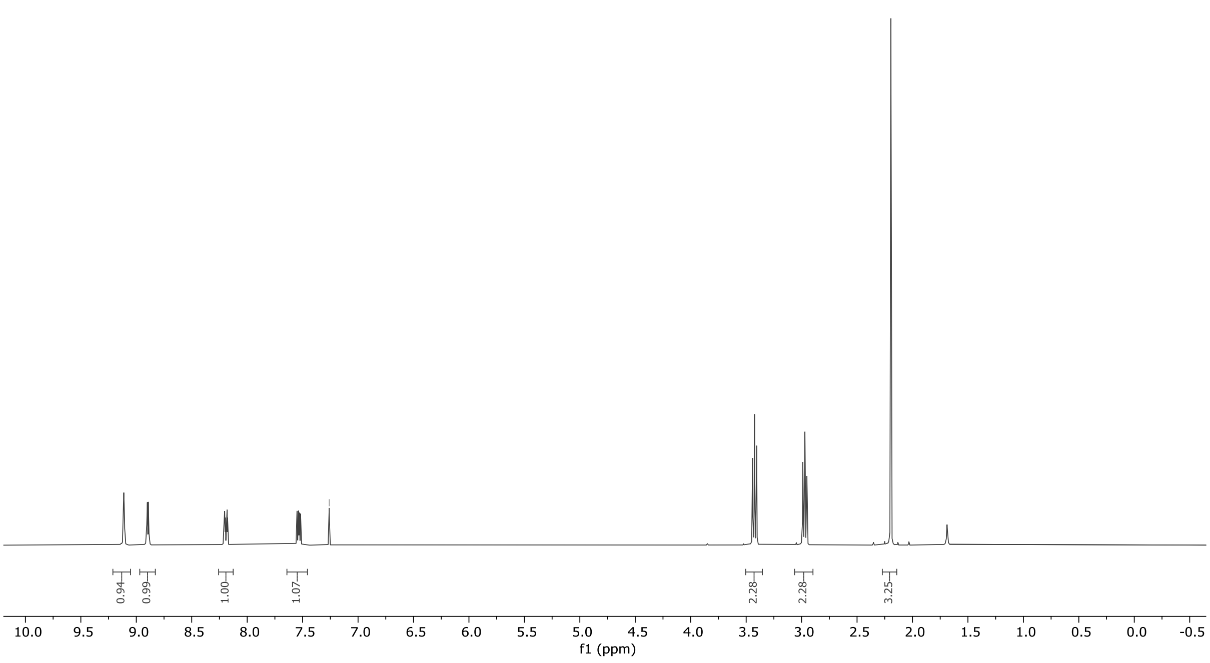

Compound **2u**
^{13}C NMR
(101 MHz, $CDCl_3$)

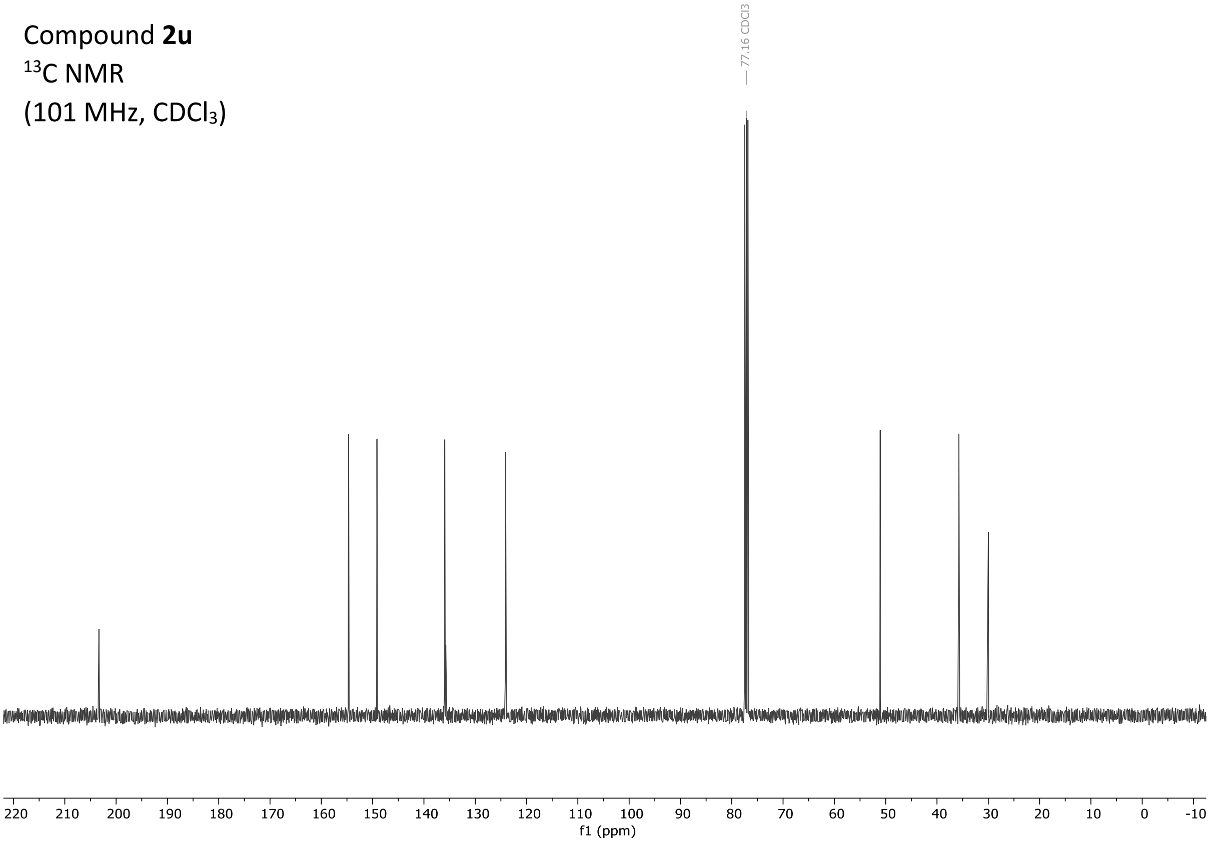

Compound **2v**
^{1}H NMR
(400 MHz, $CDCl_3$)

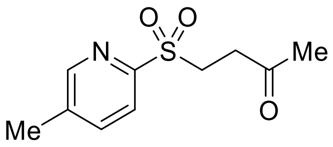

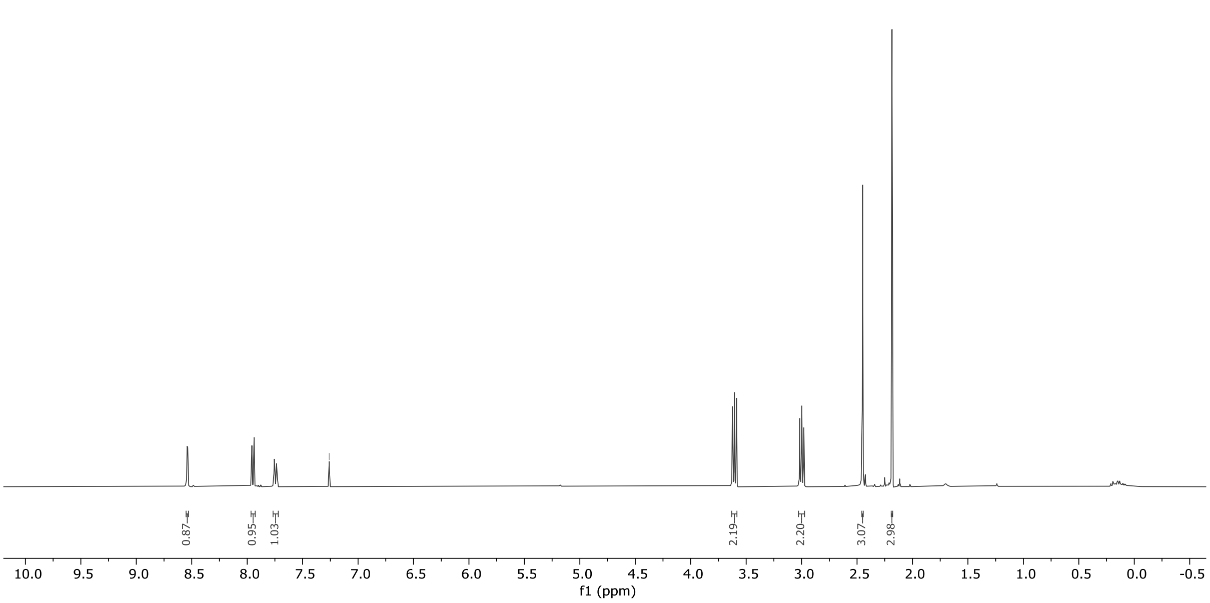

Compound **2v**
^{13}C NMR
(101 MHz, $CDCl_3$)

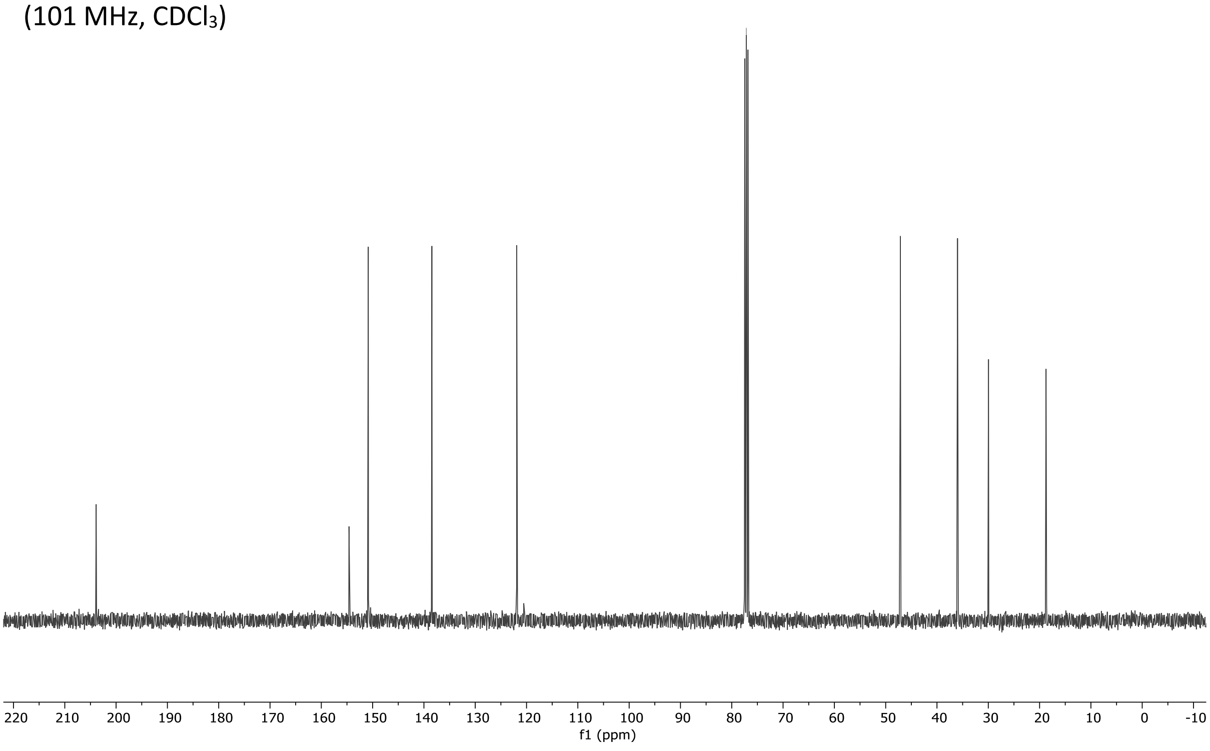

Compound **2w**
^{1}H NMR
(400 MHz, $CDCl_3$)

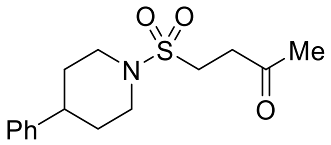

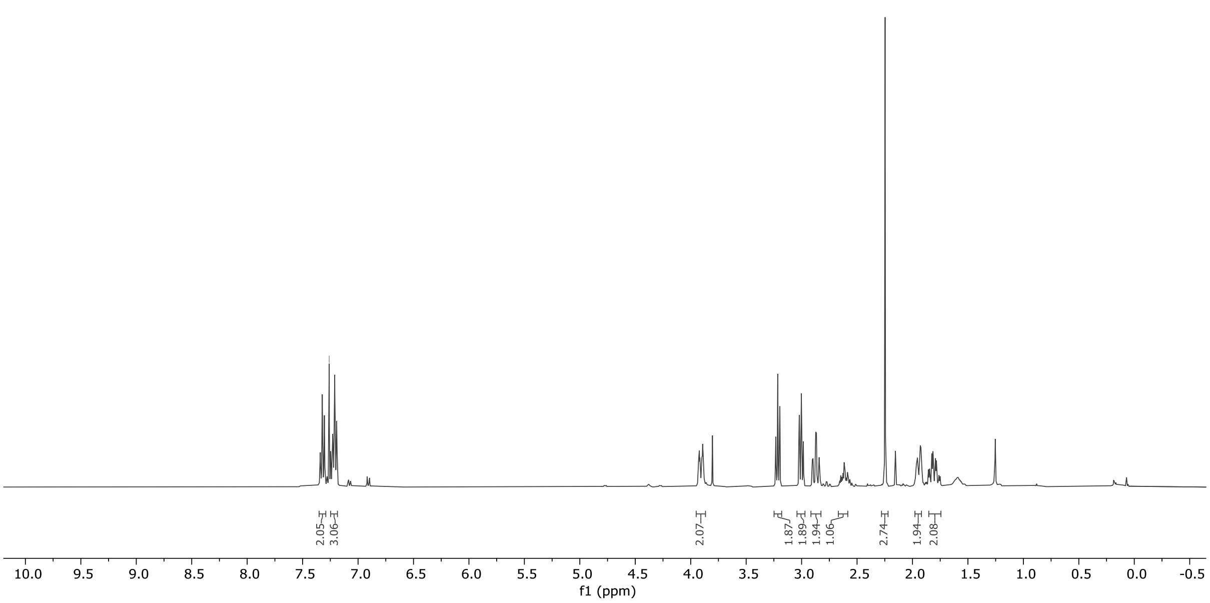

Compound **2w**
^{13}C NMR
(101 MHz, $CDCl_3$)

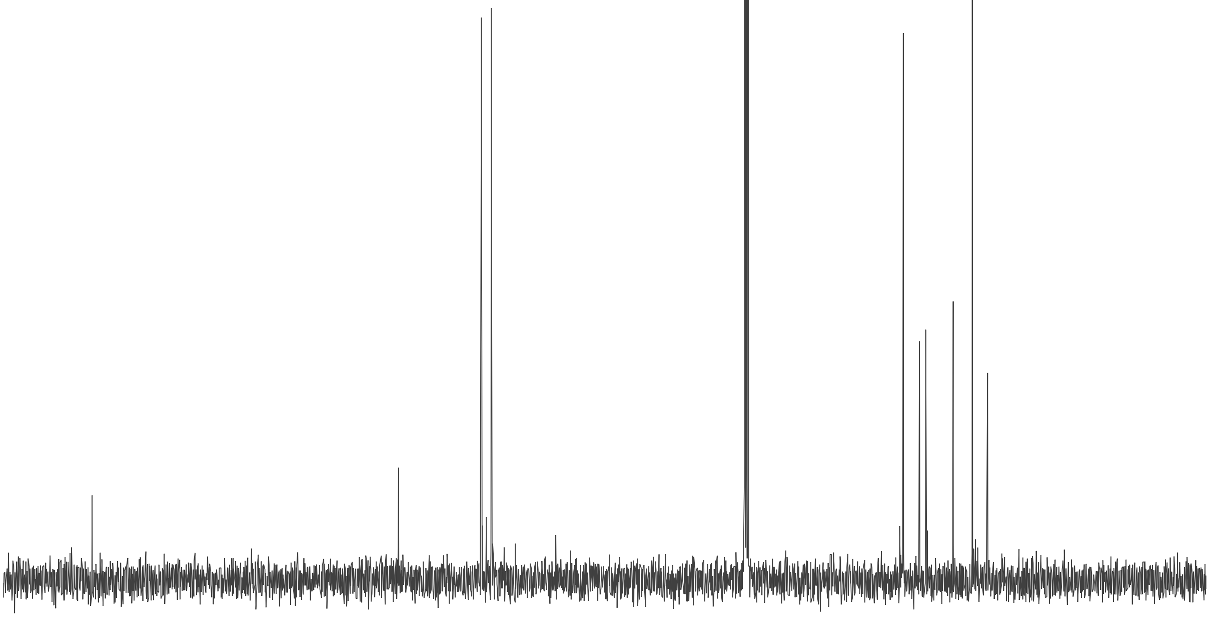

Compound **2x**
^{1}H NMR
(400 MHz, $CDCl_3$)

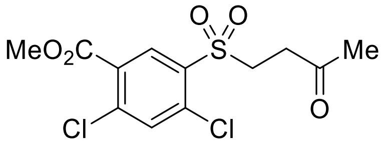

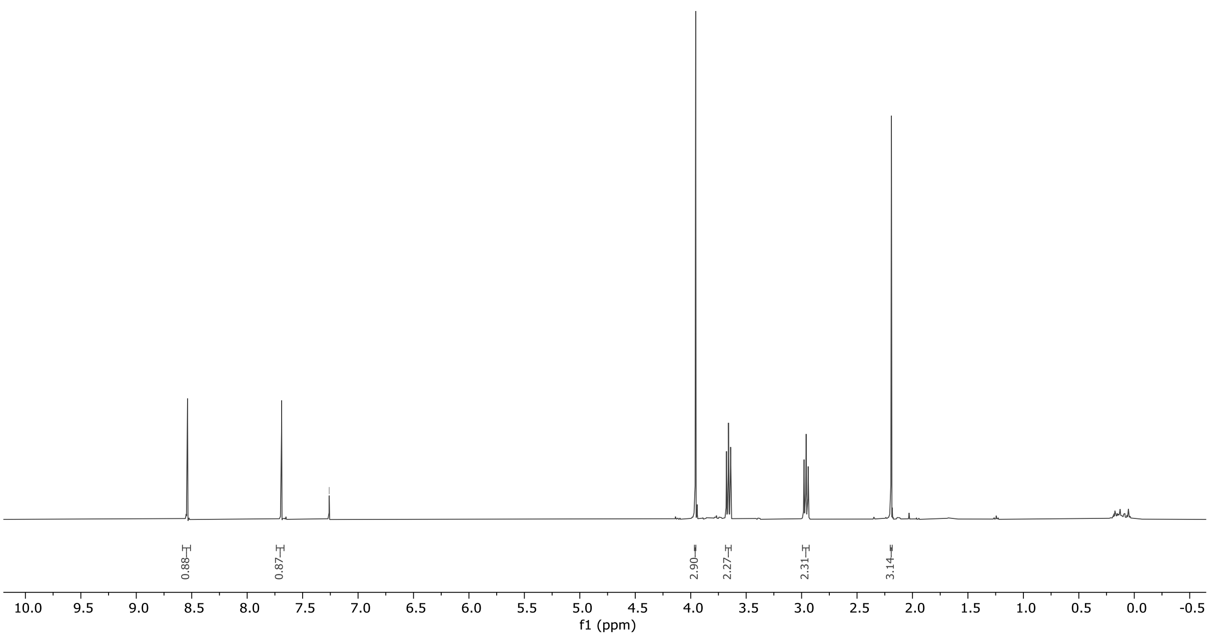

Compound **2x**
^{13}C NMR
(101 MHz, $CDCl_3$)

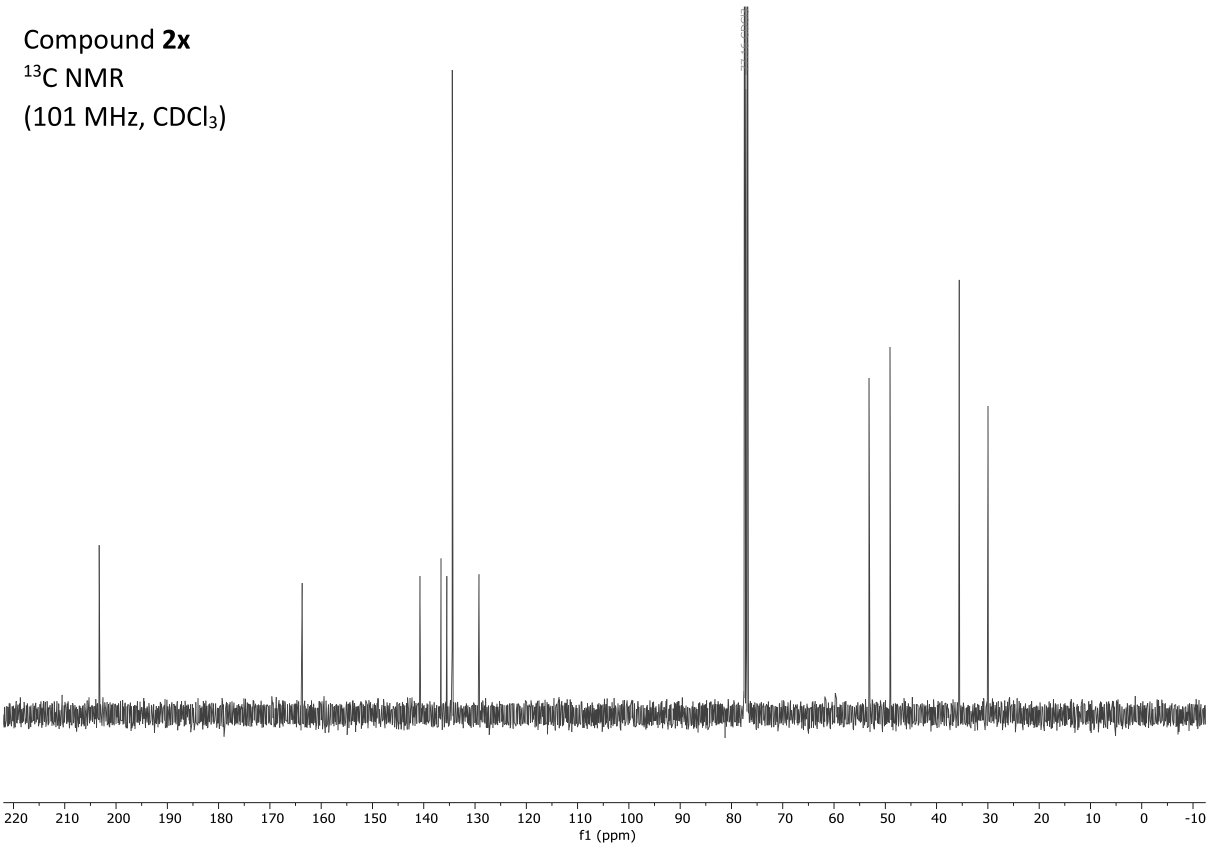

Compound **2y**
^{1}H NMR
(400 MHz, MeOD-d_4)

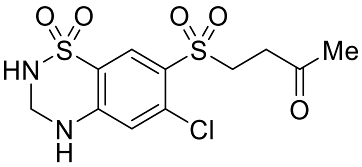

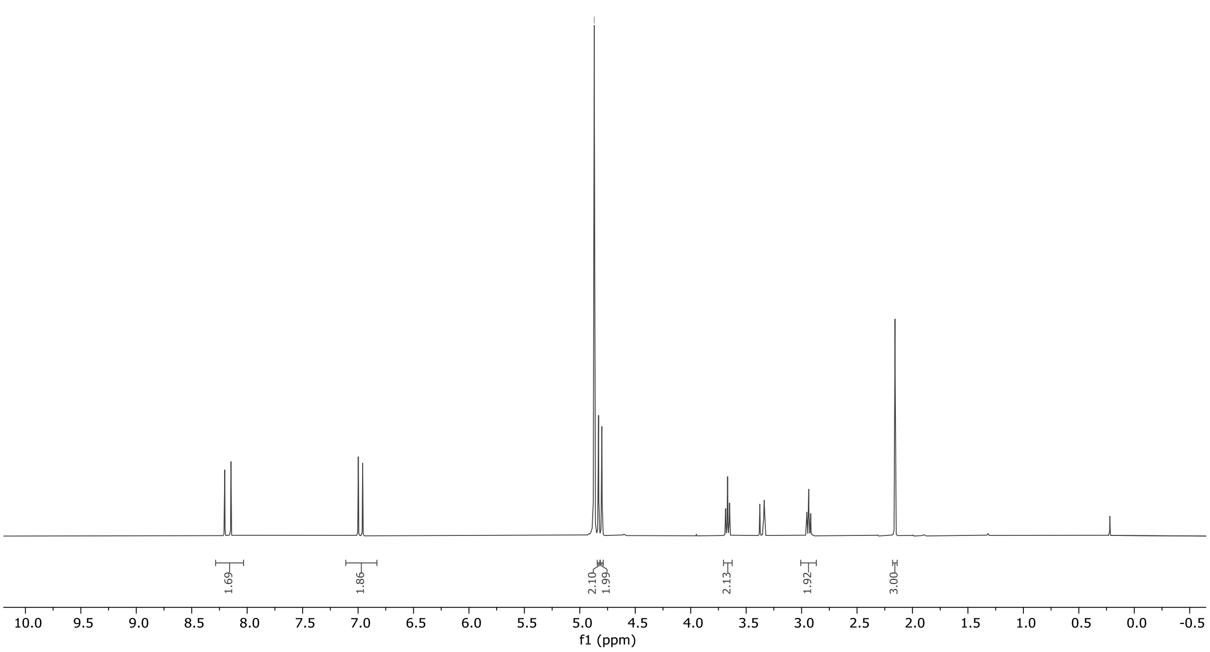

Compound **2y**
^{13}C NMR
(101 MHz, MeOD-d_4)

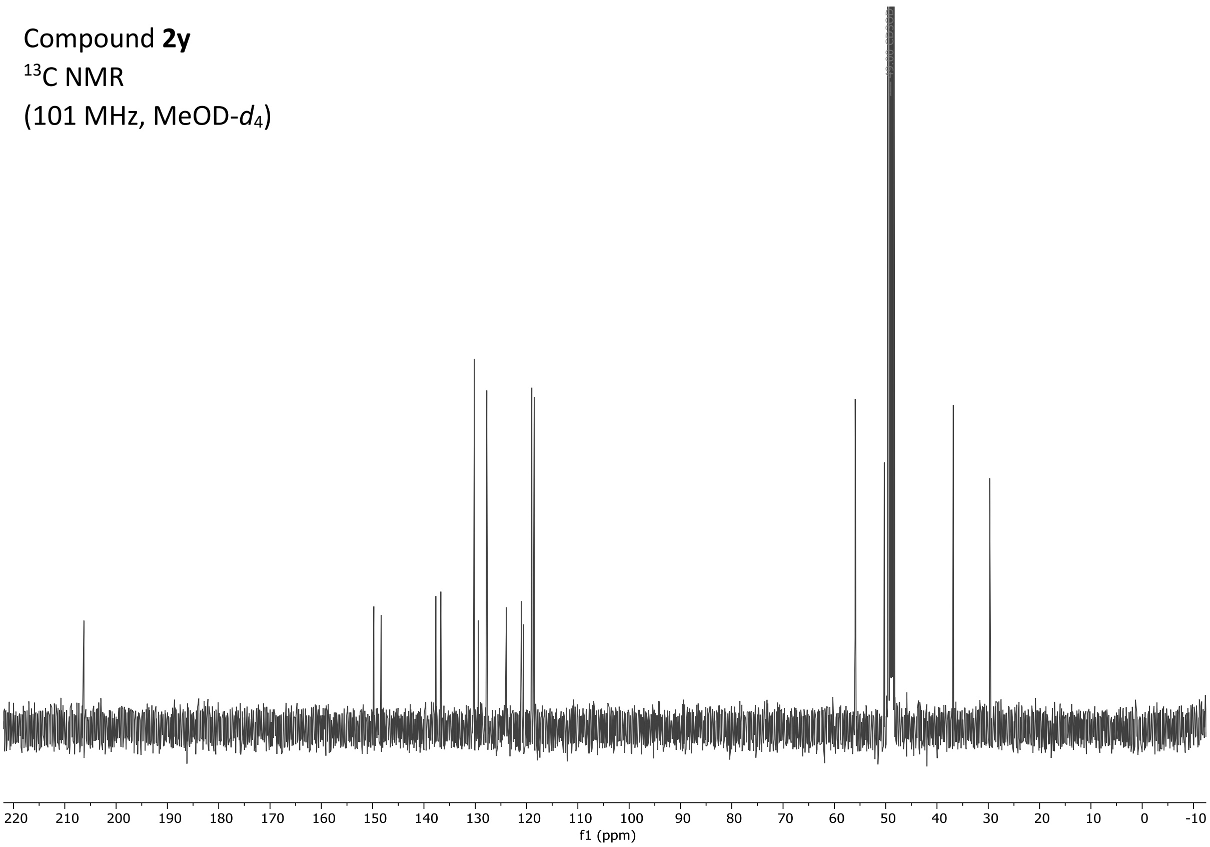

Compound **2z**
^{1}H NMR
(400 MHz, $CDCl_3$)

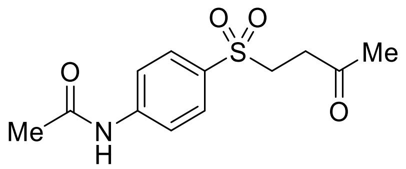

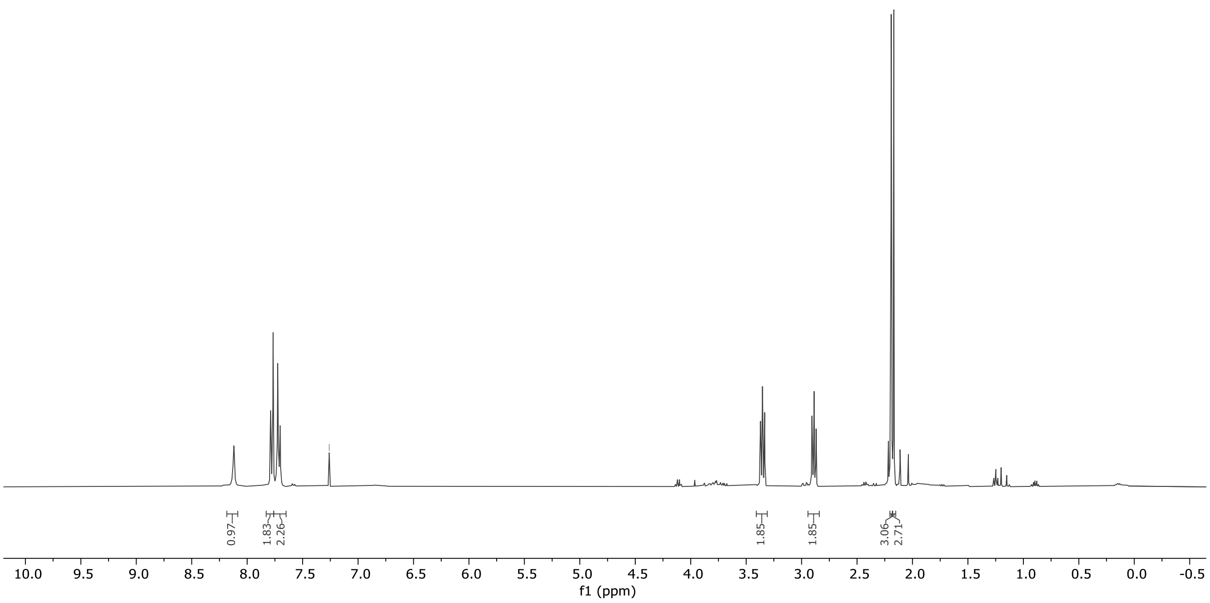

Compound **2z**
^{13}C NMR
(101 MHz, $CDCl_3$)

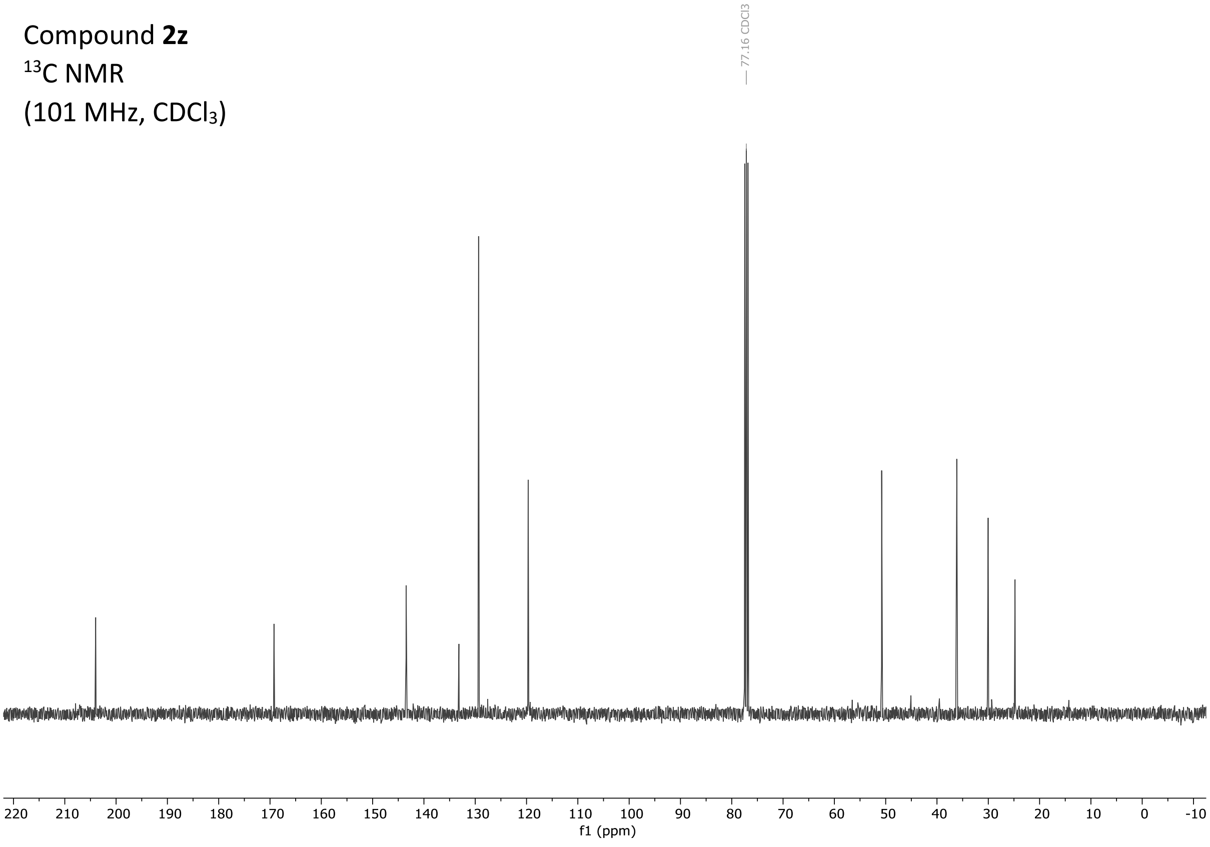

Compound **2aa**
^{1}H NMR
(400 MHz, $CDCl_3$)

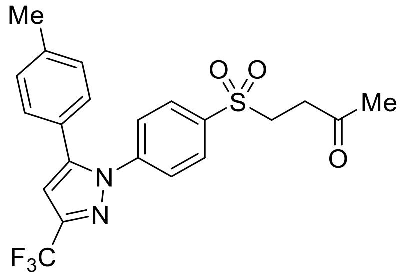

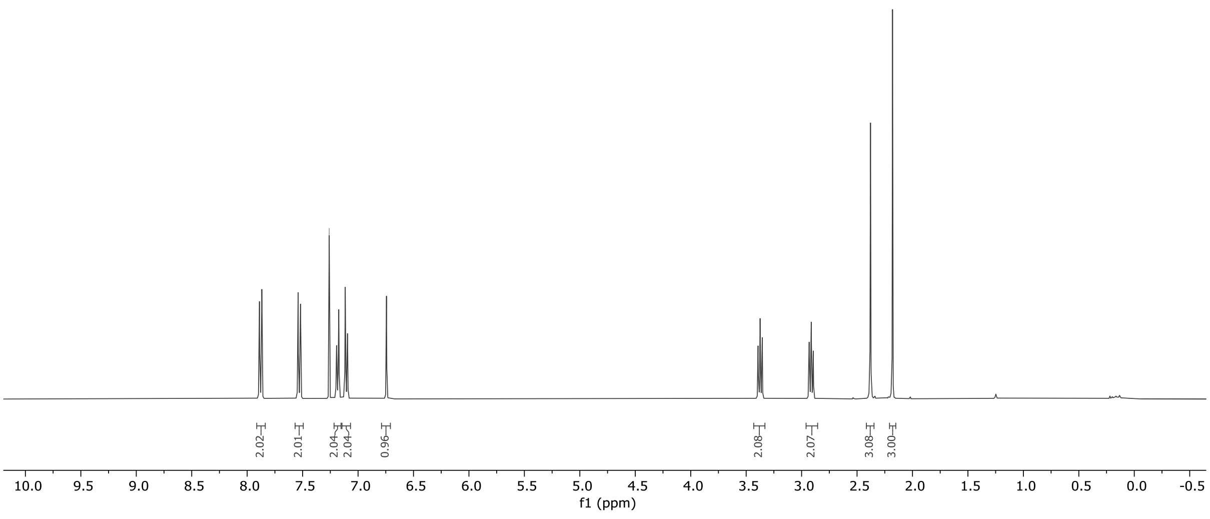

Compound **2aa**
^{13}C NMR
(101 MHz, $CDCl_3$)

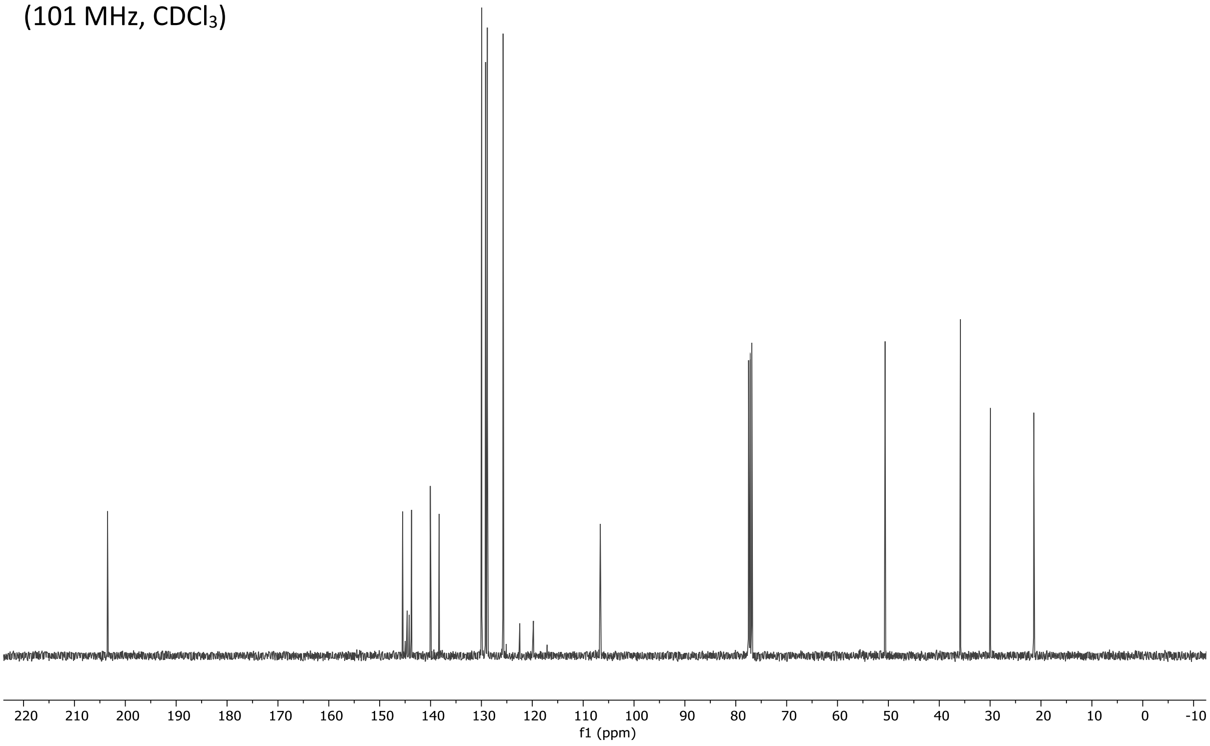

Compound **2aa**

^{19}F NMR

(377 MHz, $CDCl_3$)

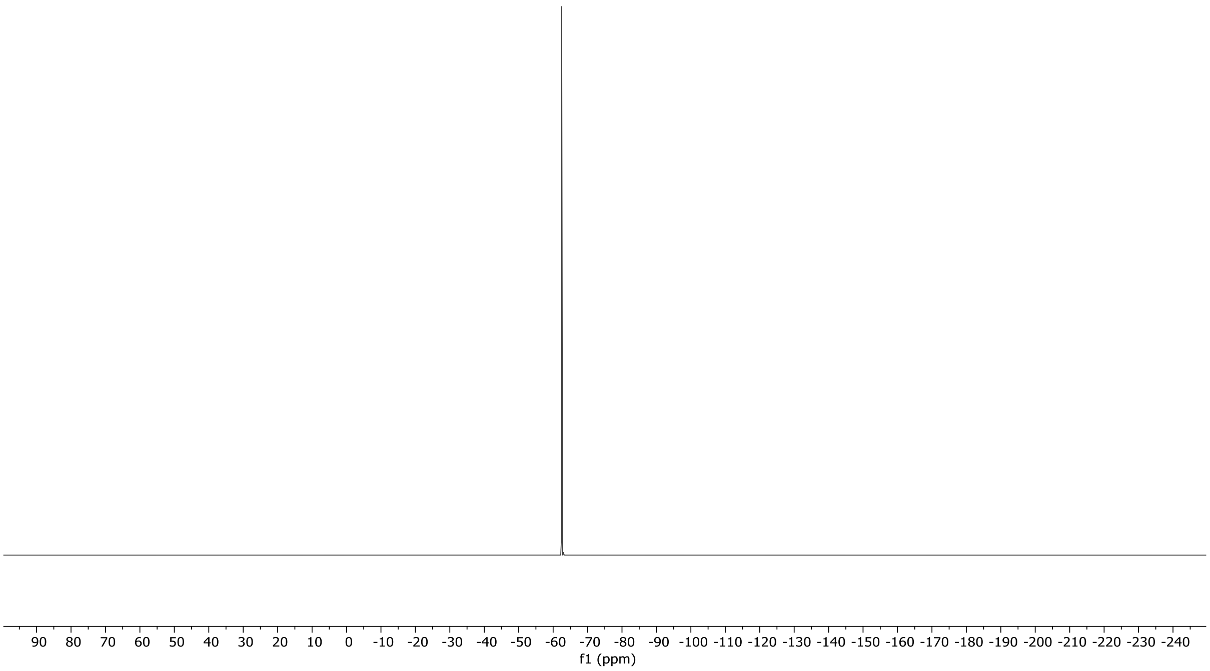

Compound **2ab**
^{1}H NMR
(400 MHz, $CDCl_3$)

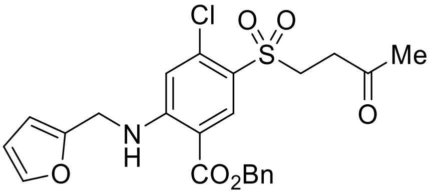

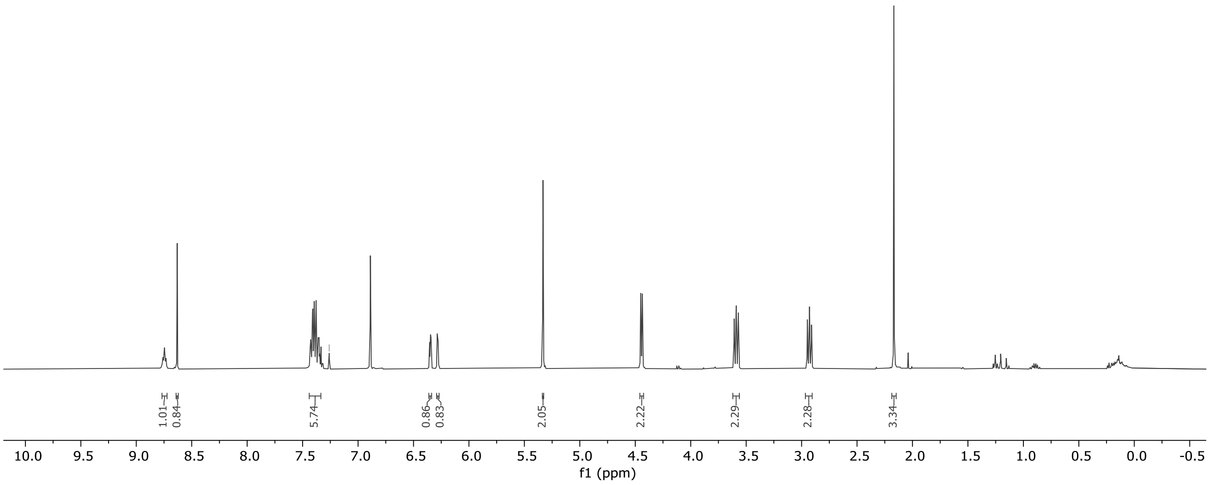

Compound **2ab**
^{13}C NMR
(101 MHz, $CDCl_3$)

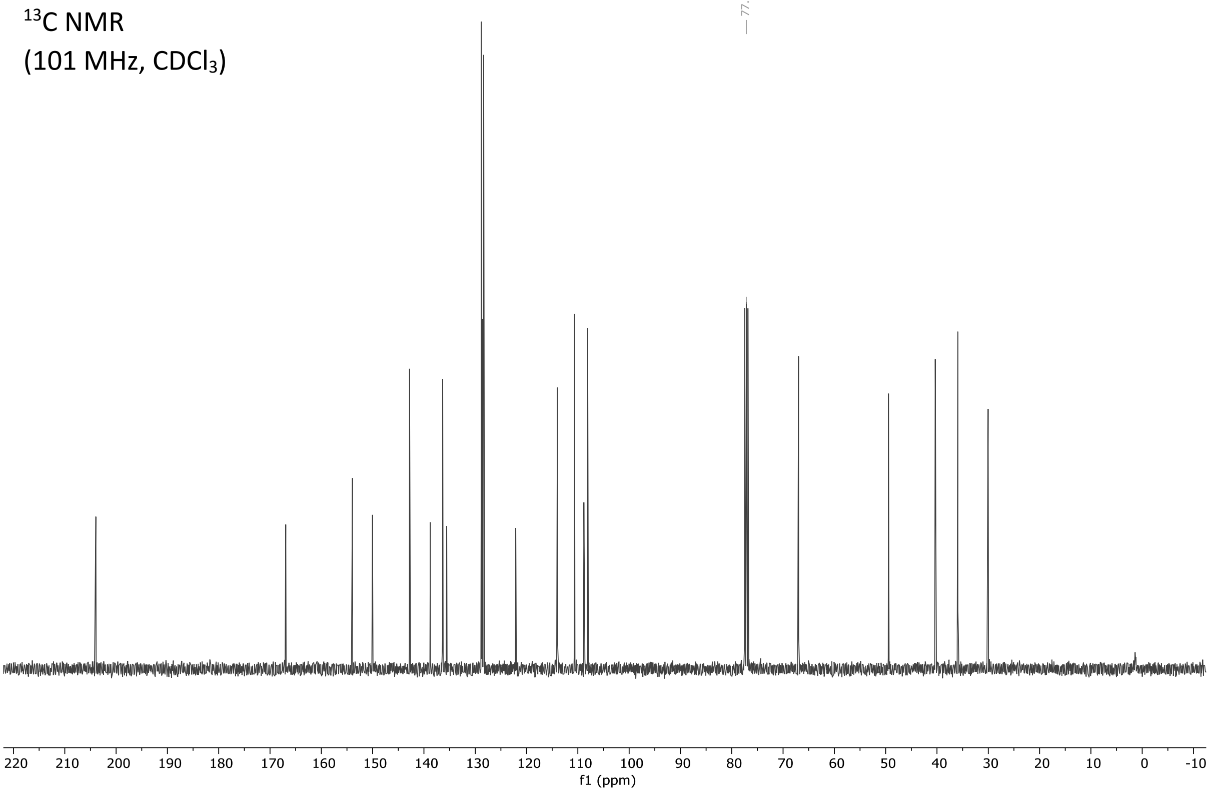

Compound **2ac**
^{1}H NMR
(400 MHz, $CDCl_3$)

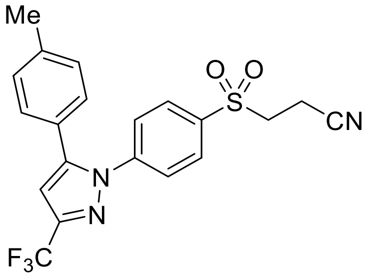

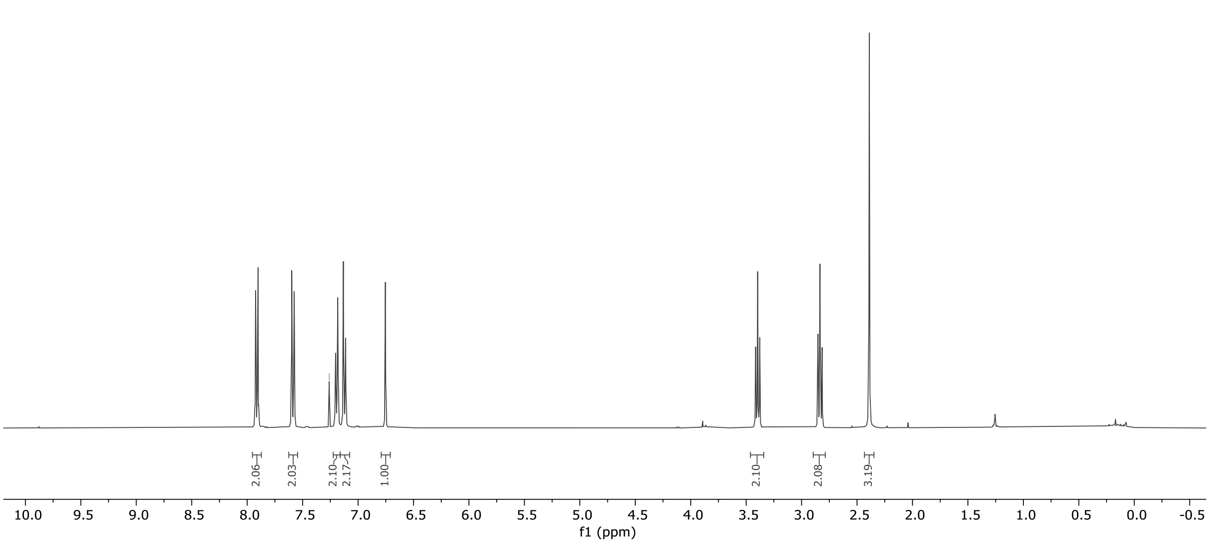

Compound **2ac**
^{13}C NMR
(101 MHz, $CDCl_3$)

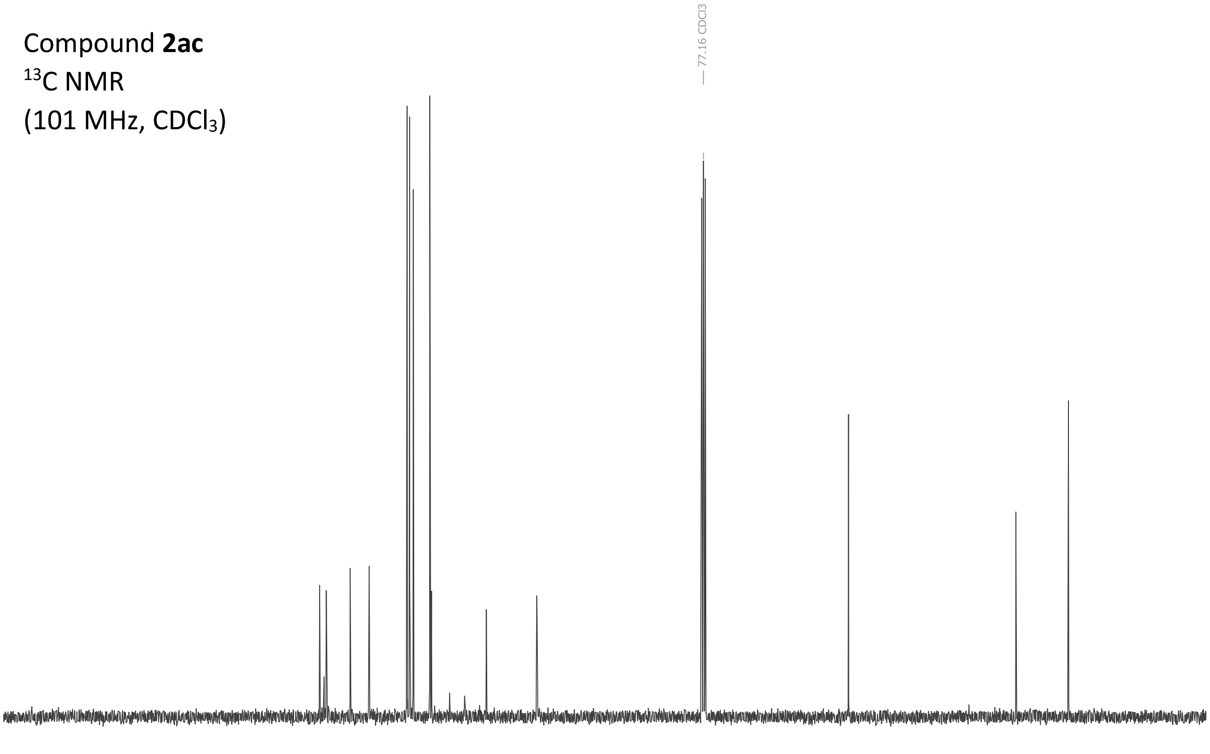

Compound **2ac**
^{19}F NMR
(377 MHz, $CDCl_3$)

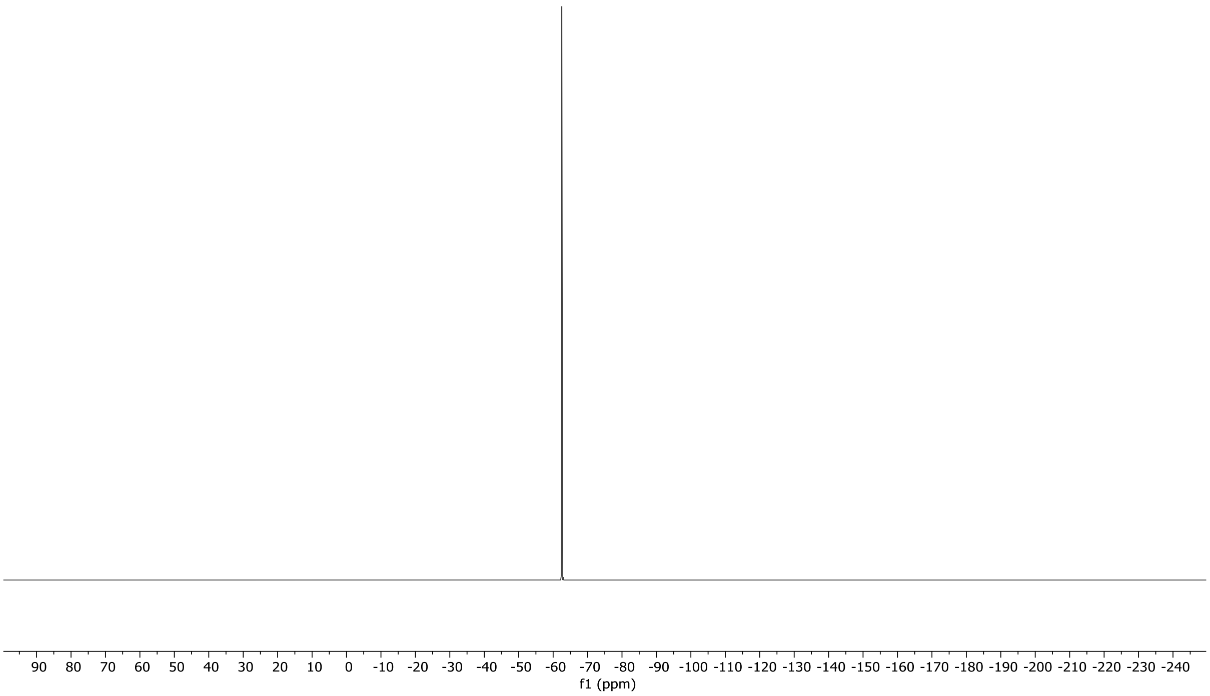

Compound **2ad**
^{1}H NMR
(400 MHz, $CDCl_3$)

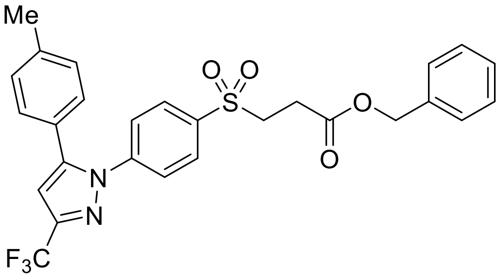

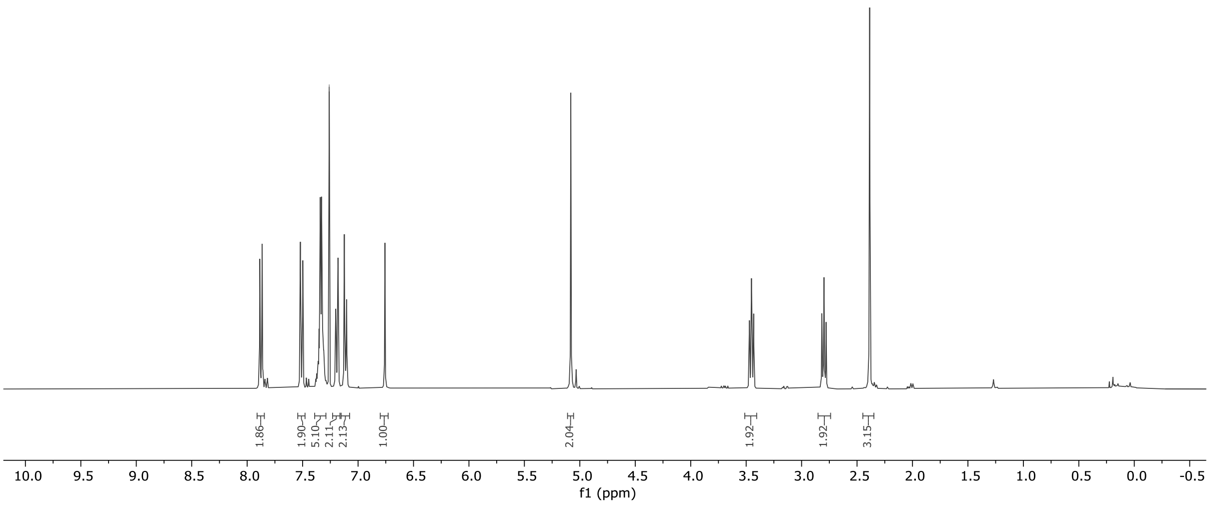

Compound **2ad**
^{13}C NMR
(101 MHz, $CDCl_3$)

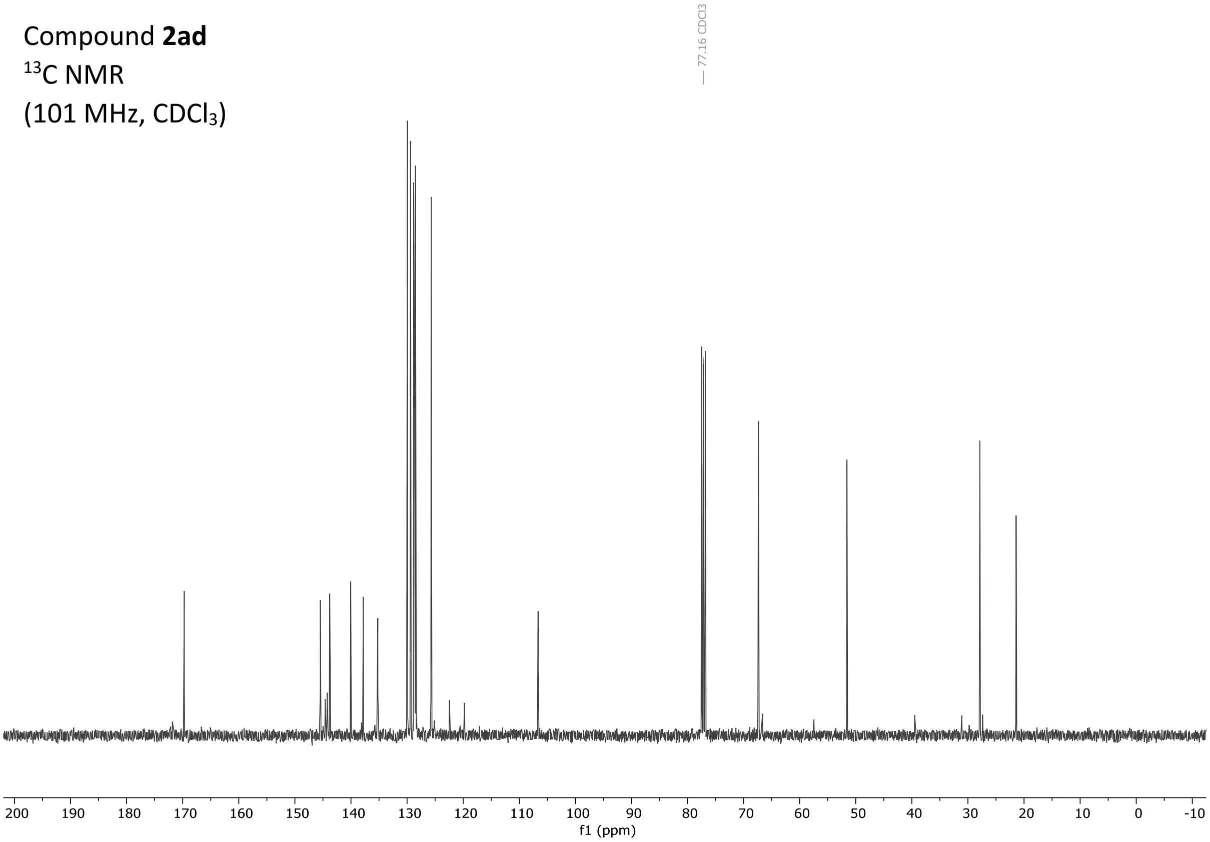

Compound **2ad**

^{19}F NMR

(377 MHz, $CDCl_3$)

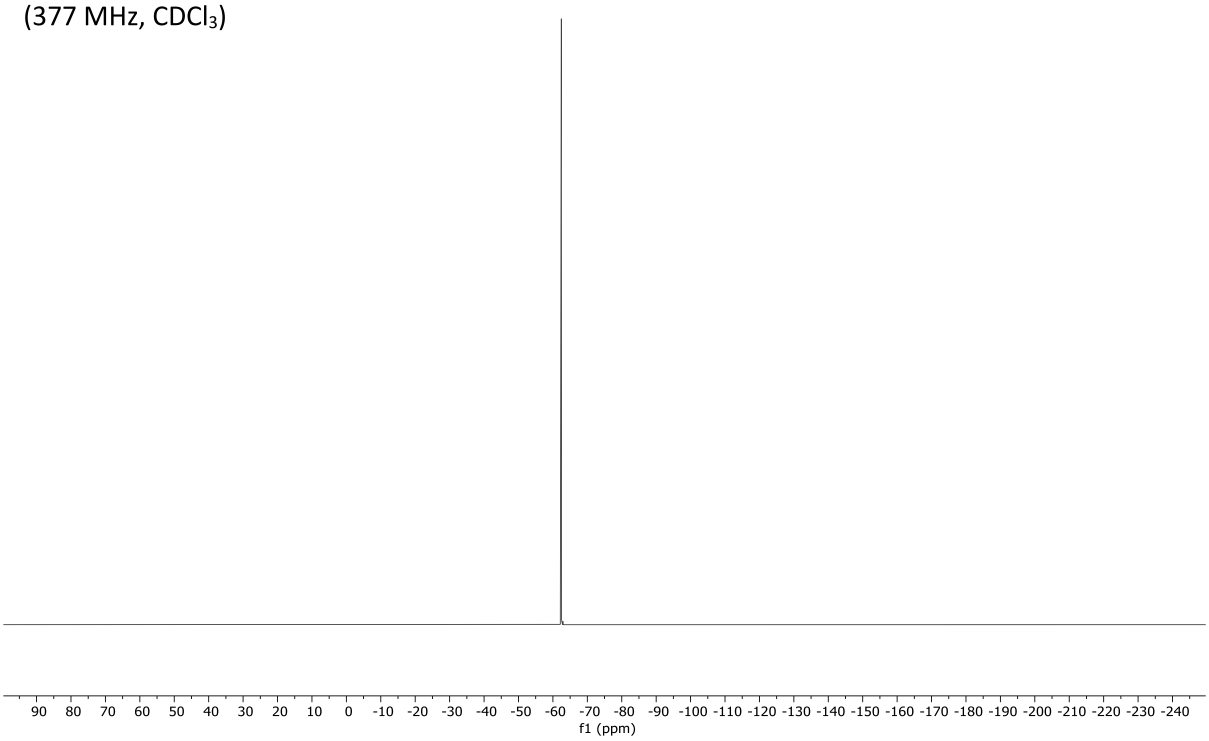

Compound **2ae**
^{1}H NMR
(400 MHz, $CDCl_3$)

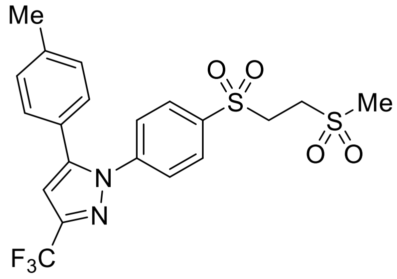

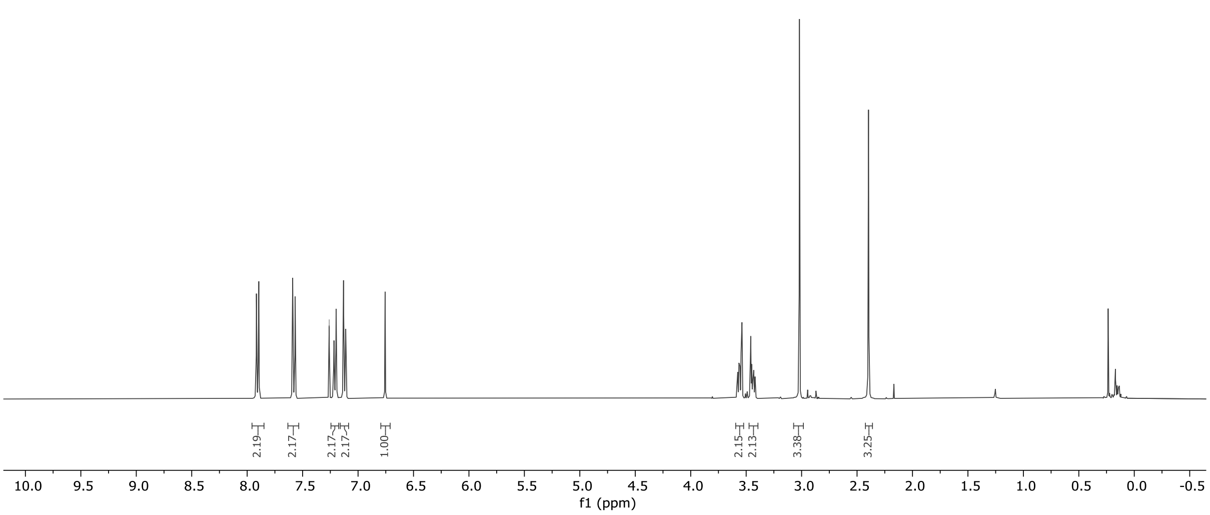

Compound **2ae**
^{13}C NMR
(126 MHz, $CDCl_3$)

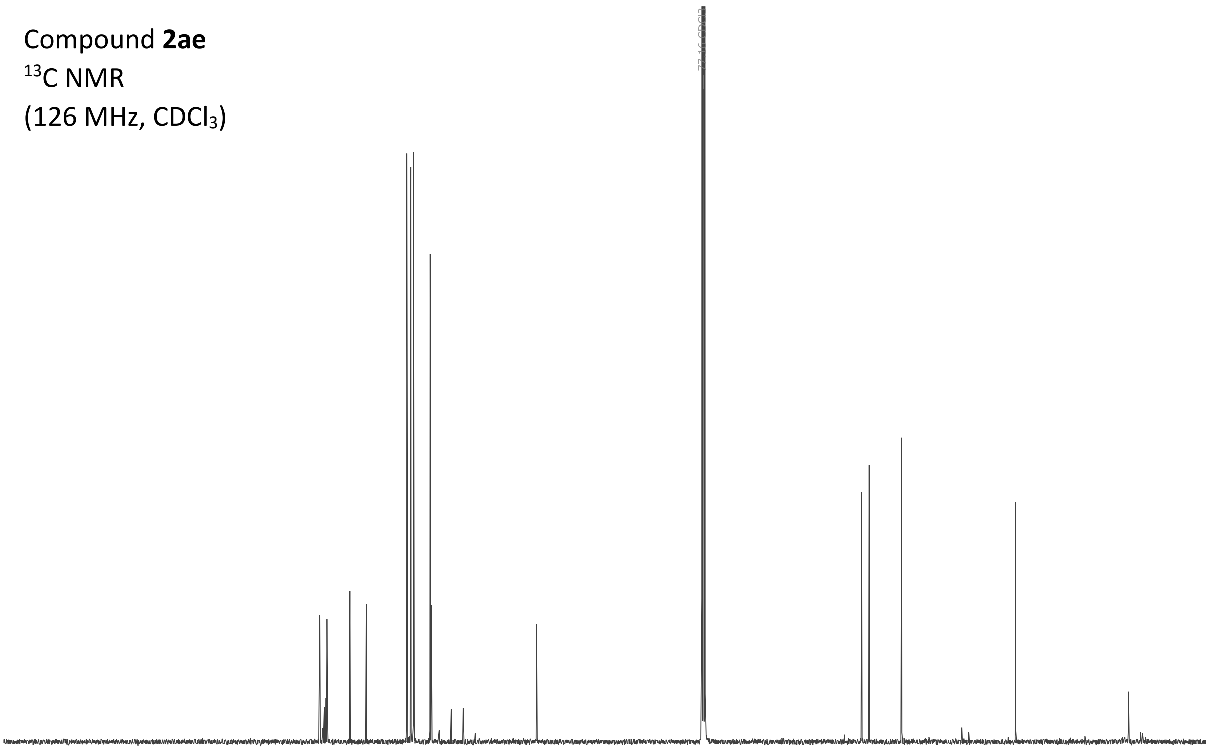

Compound **2ae**
^{19}F NMR
(377 MHz, $CDCl_3$)

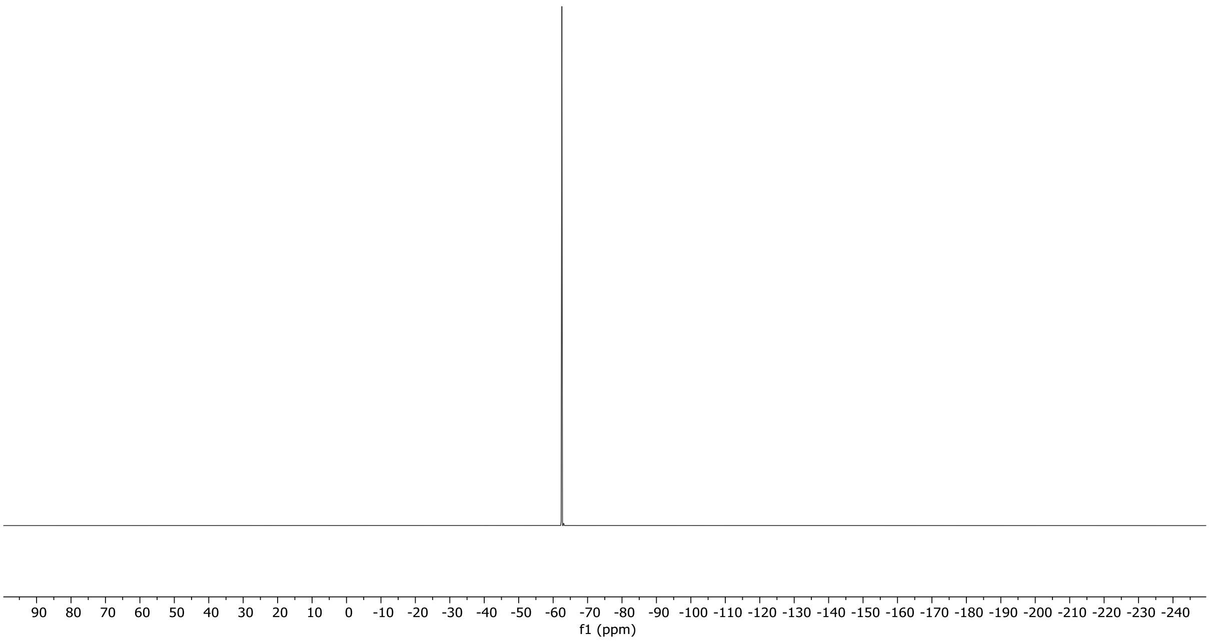

Compound **2af**
^{1}H NMR
(400 MHz, $CDCl_3$)

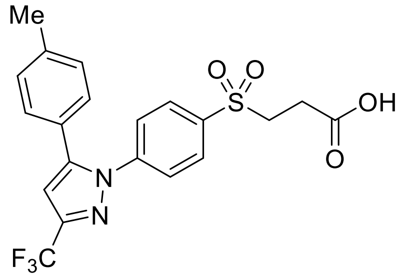

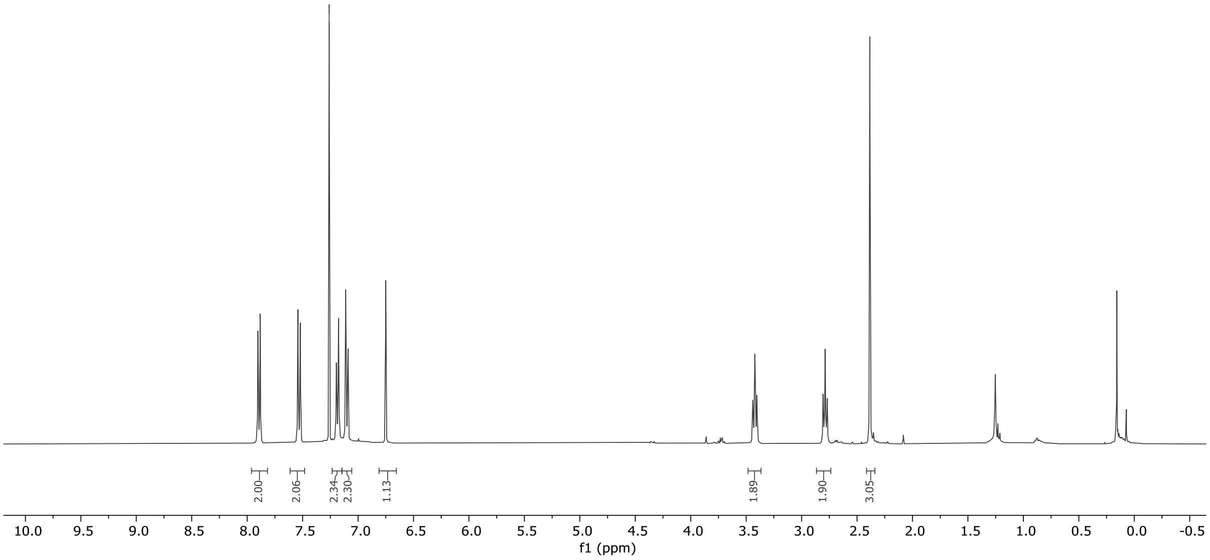

Compound **2af**
^{13}C NMR
(151 MHz, $CDCl_3$)

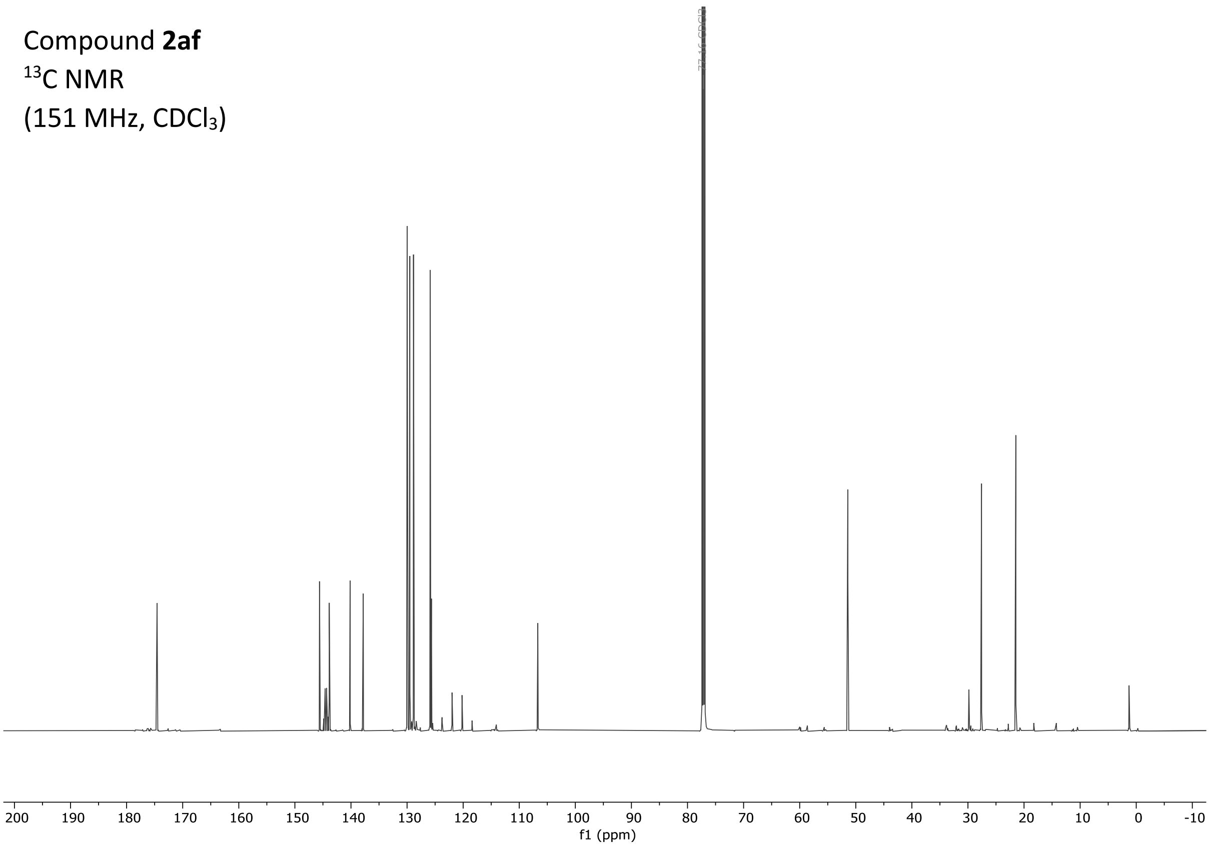

Compound **2af**
^{19}F NMR
(377 MHz, $CDCl_3$)

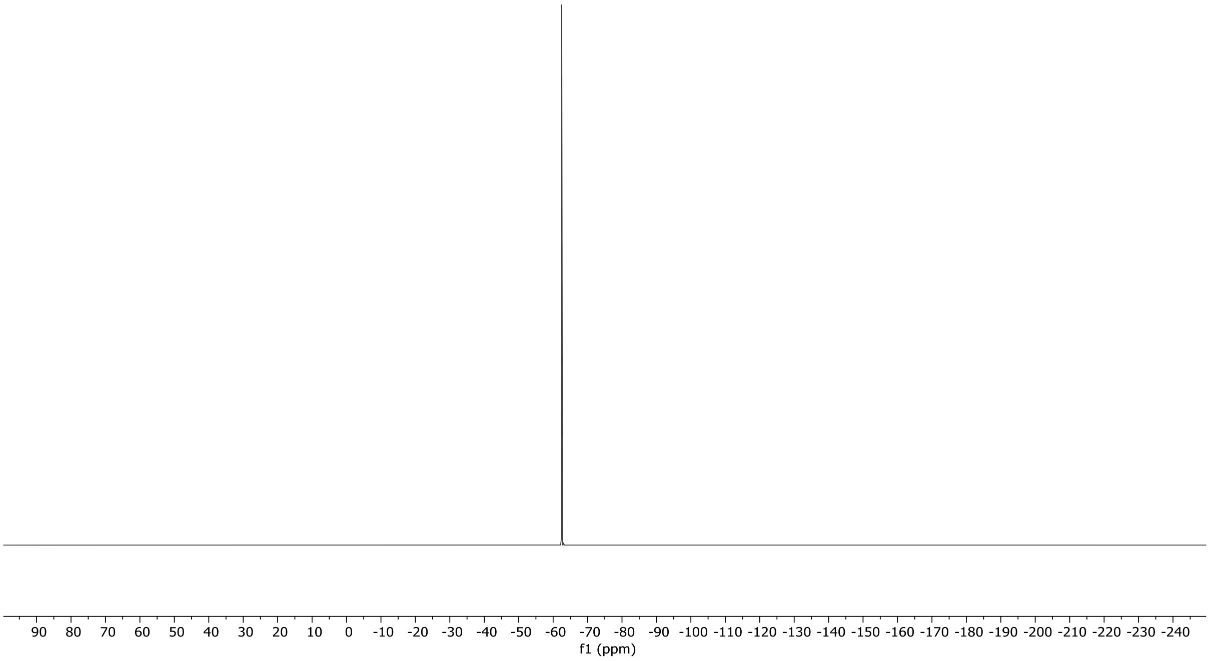

Compound **2ag**
^{1}H NMR
(600 MHz, $CDCl_3$)

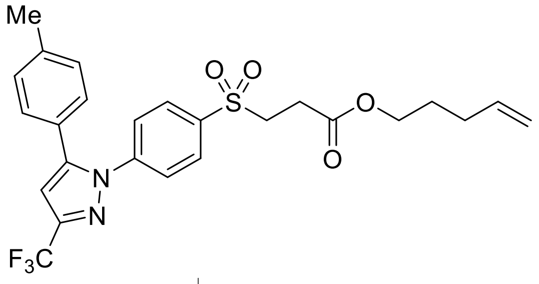

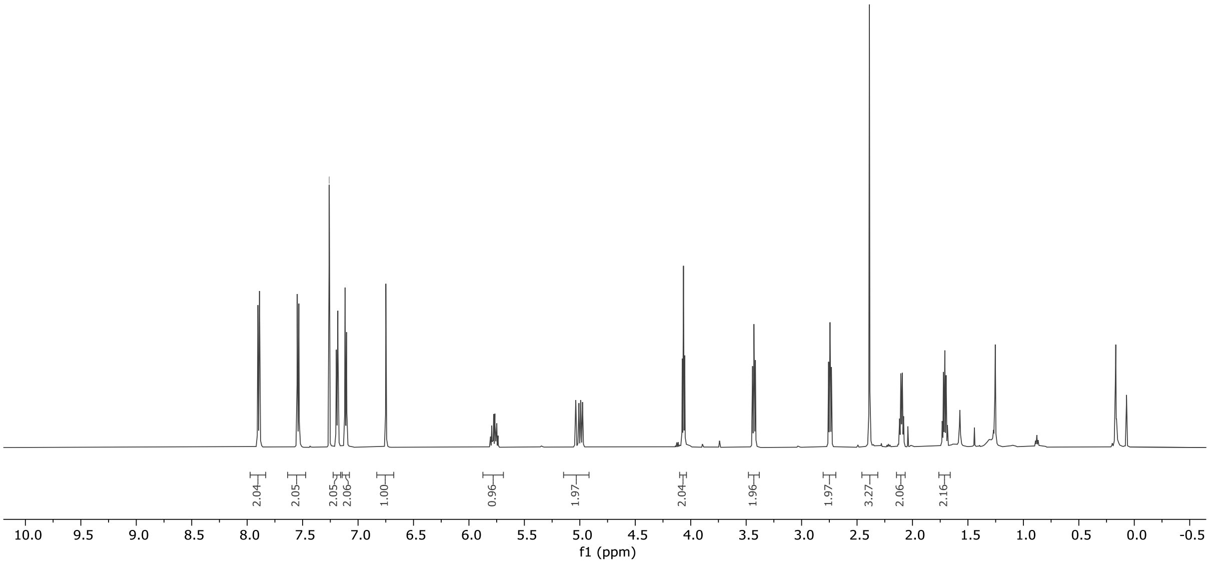

Compound **2ag**
^{13}C NMR
(151 MHz, $CDCl_3$)

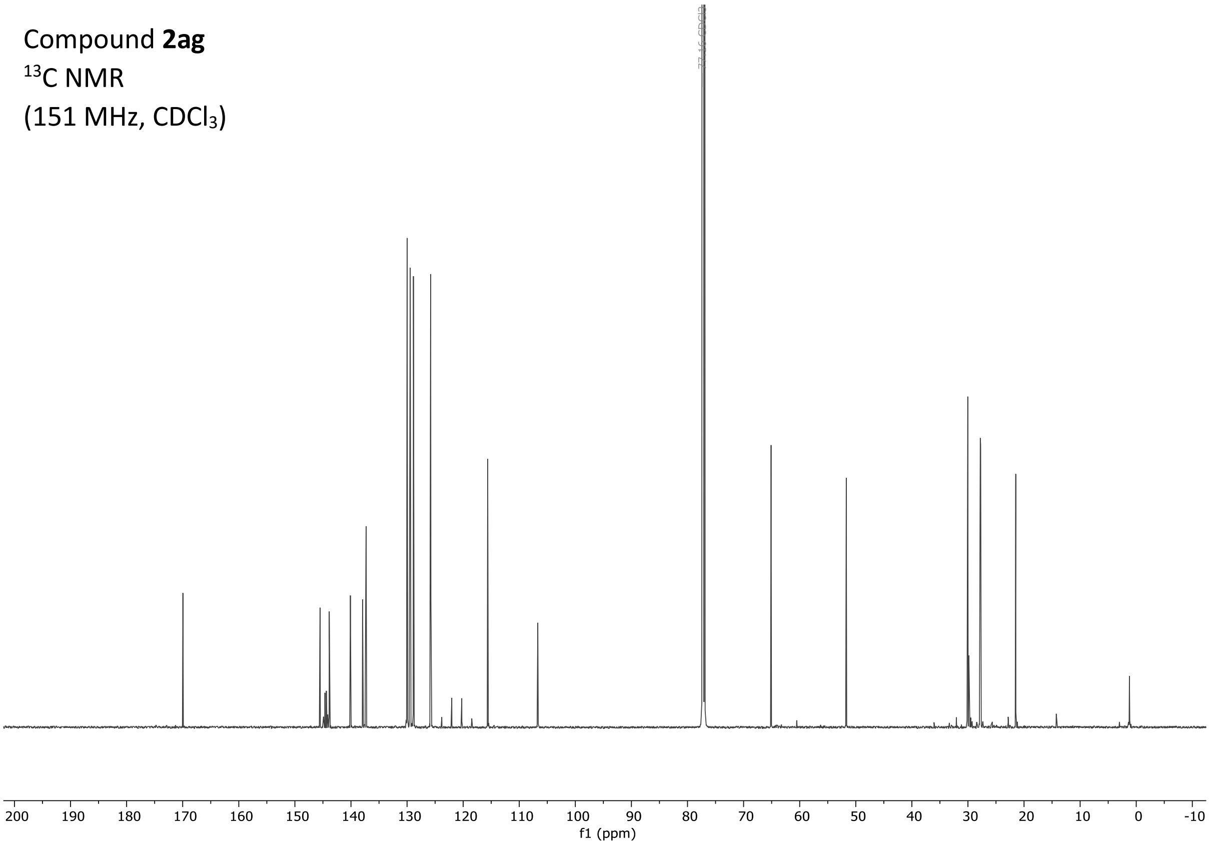

Compound **2ag**
^{19}F NMR
(377 MHz, $CDCl_3$)

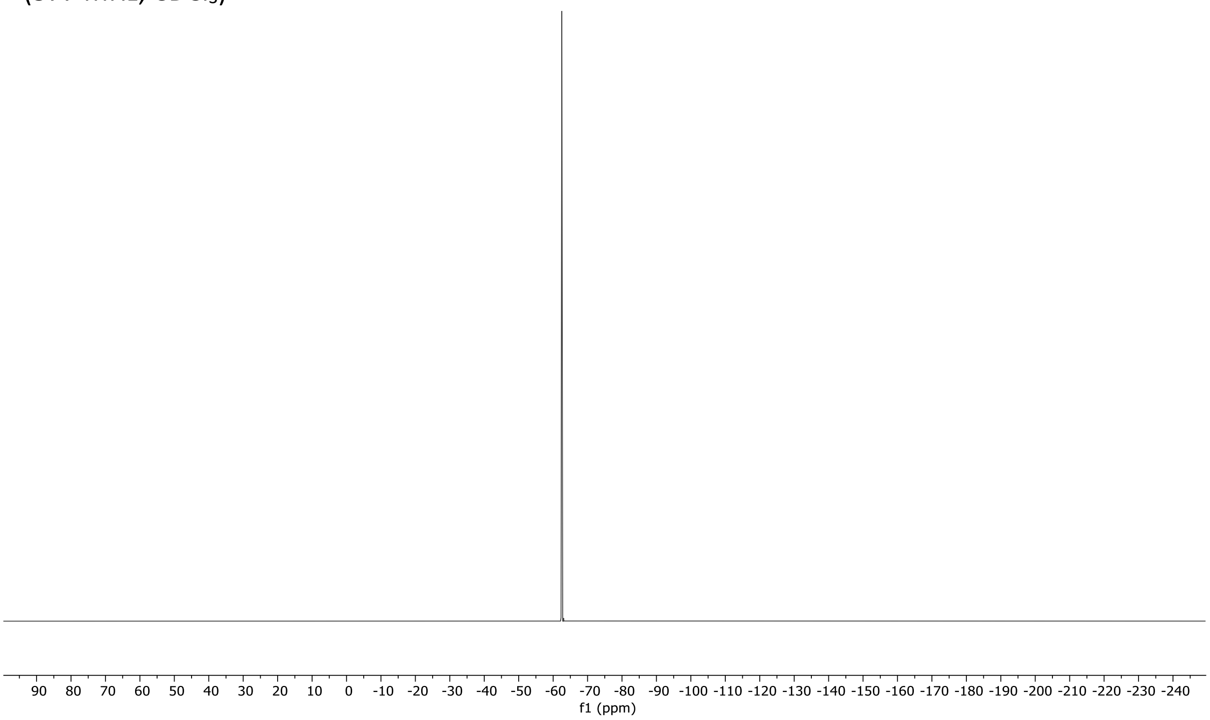

Compound **2ah**
^{1}H NMR
(600 MHz, $CDCl_3$)

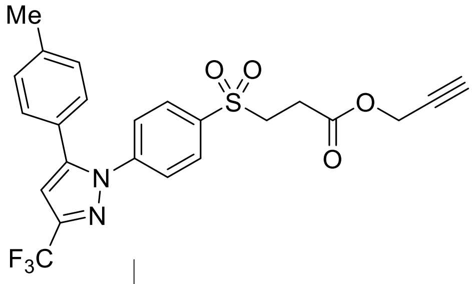

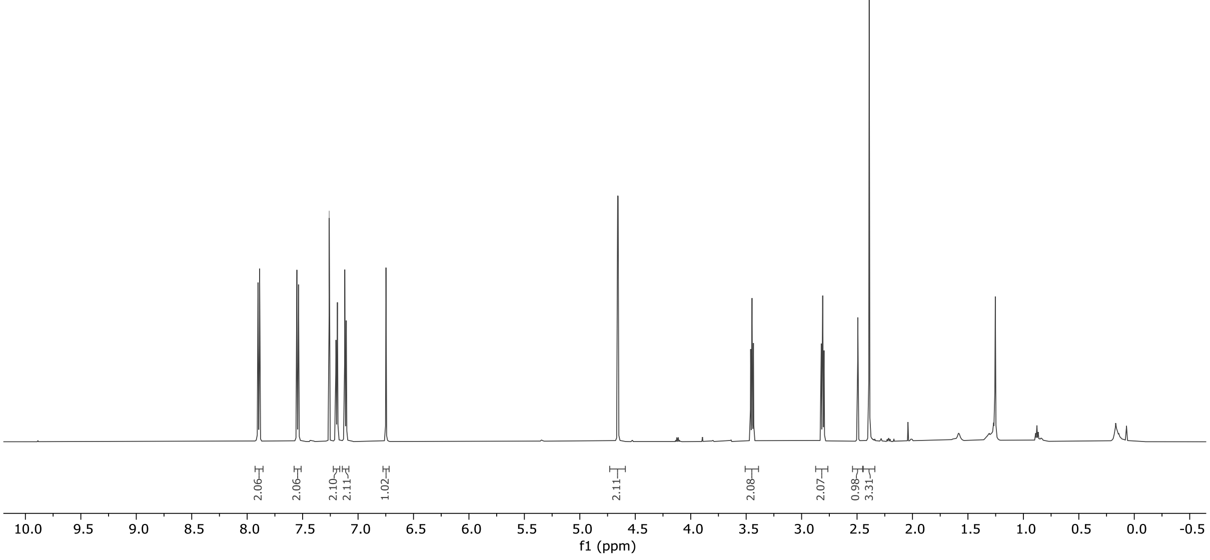

Compound **2ah**
^{13}C NMR
(151 MHz, $CDCl_3$)

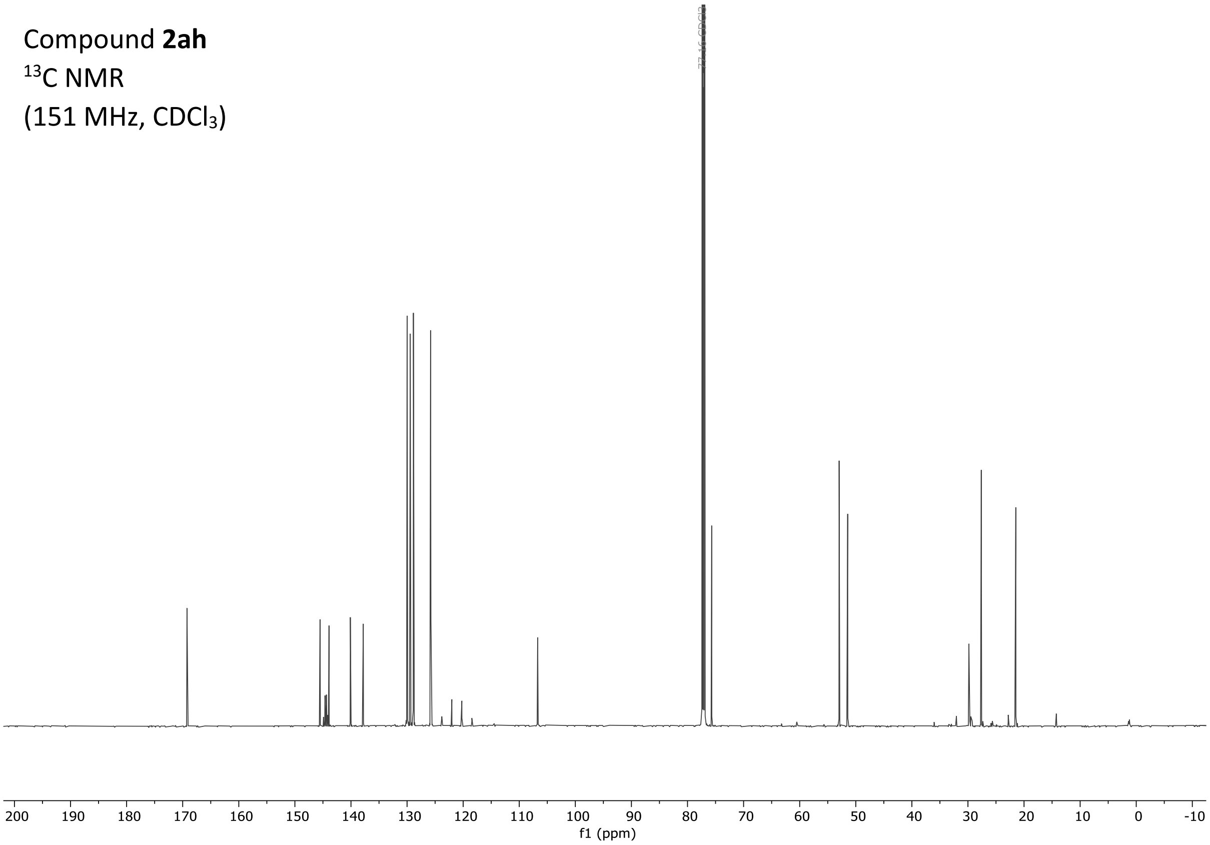

Compound **2ah**
^{19}F NMR
(377 MHz, $CDCl_3$)

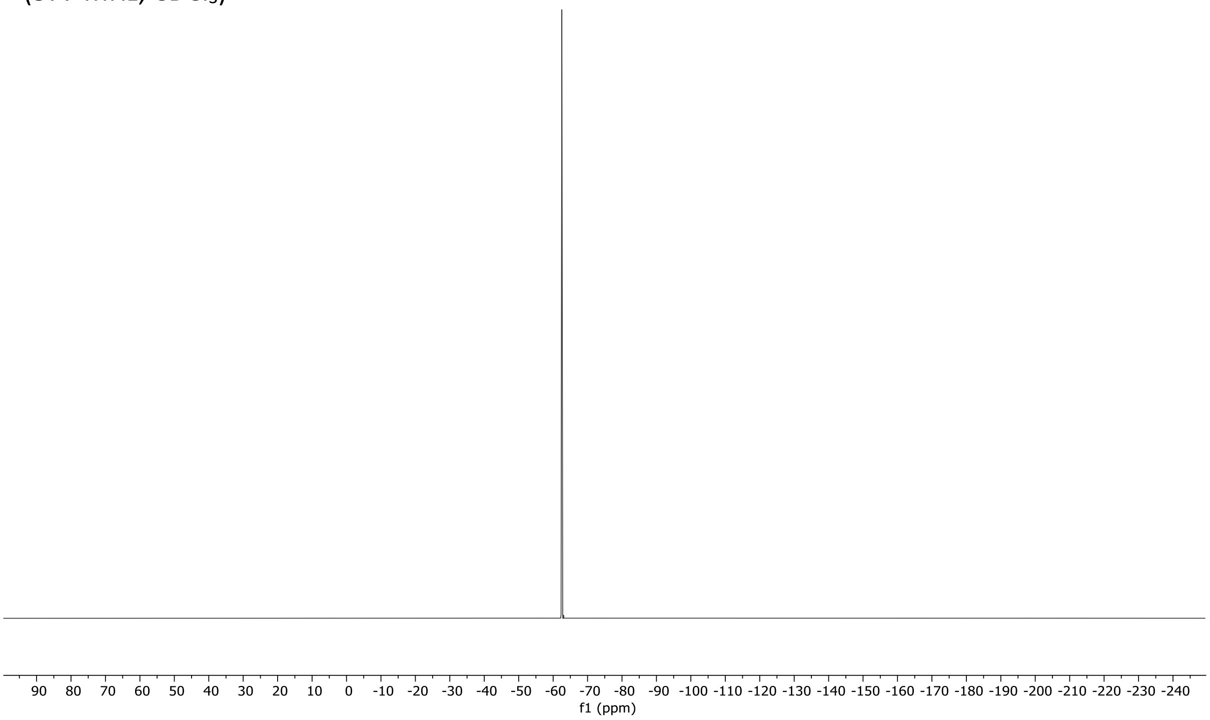

Compound **2ai**
^{1}H NMR
(400 MHz, $CDCl_3$)

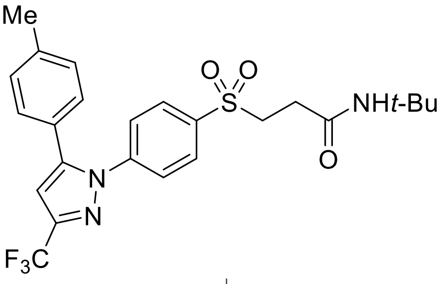

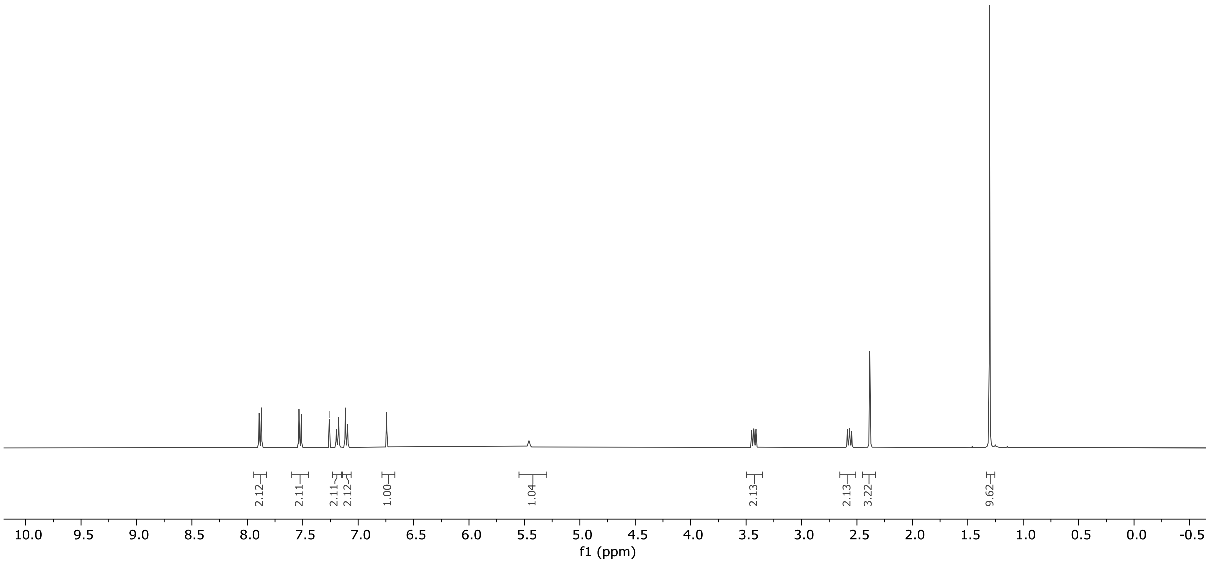

Compound **2ai**
^{13}C NMR
(151 MHz, $CDCl_3$)

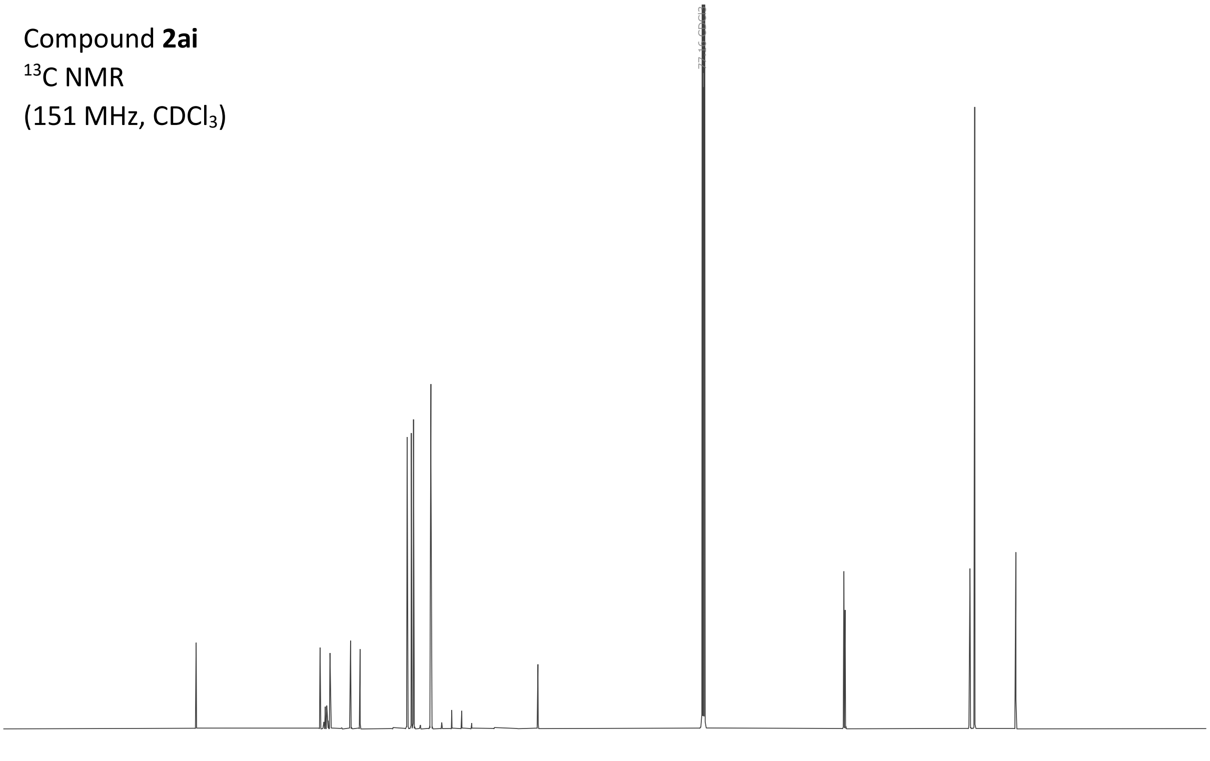

Compound **2ai**
^{19}F NMR
(377 MHz, $CDCl_3$)

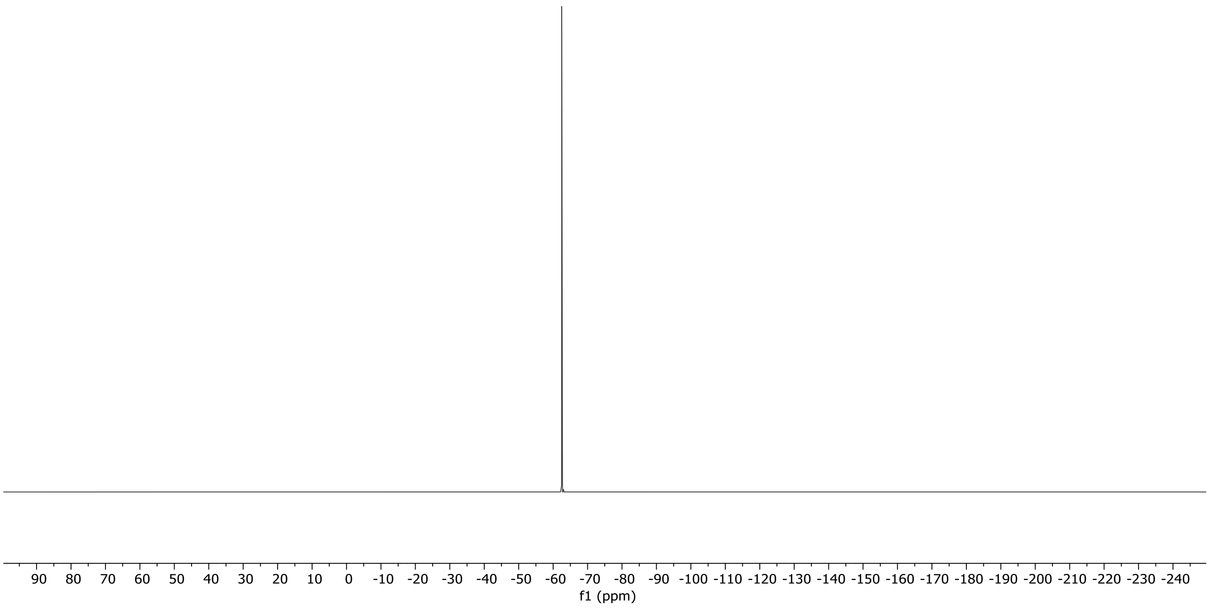

Compound **2aj**
^{1}H NMR
(400 MHz, $CDCl_3$)

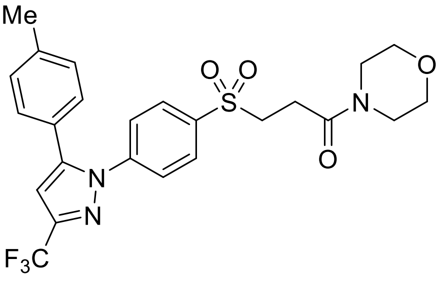

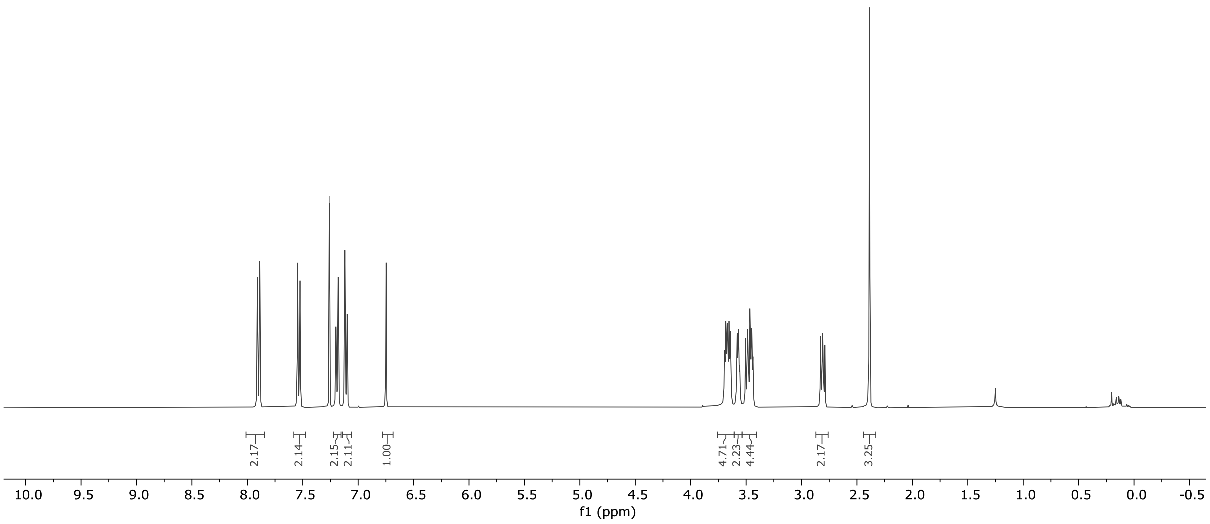

Compound **2aj**
^{13}C NMR
(101 MHz, $CDCl_3$)

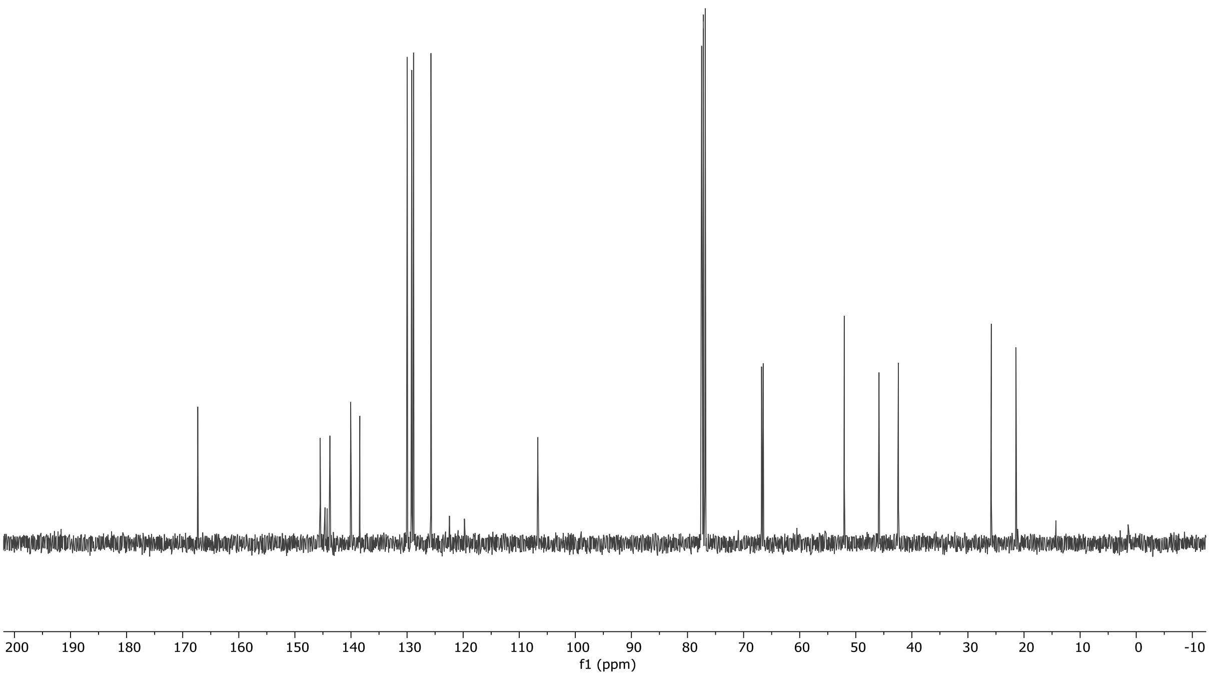

Compound **2aj**
^{19}F NMR
(377 MHz, $CDCl_3$)

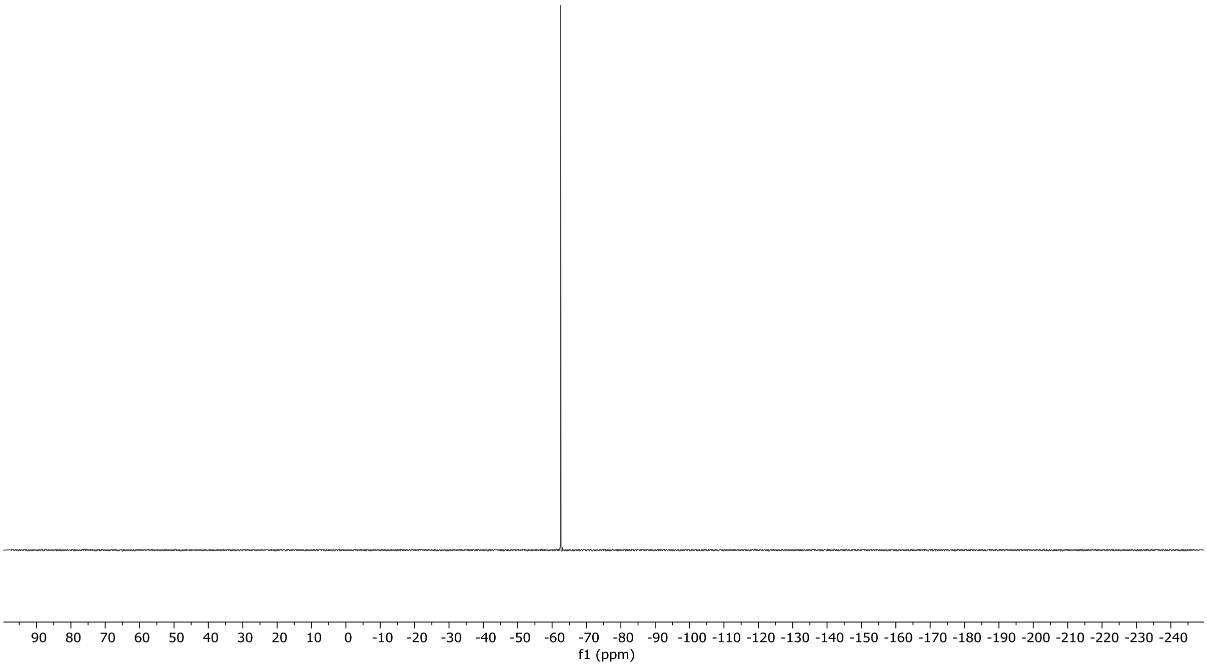

Compound **2ak**
^{1}H NMR
(400 MHz, $CDCl_3$)

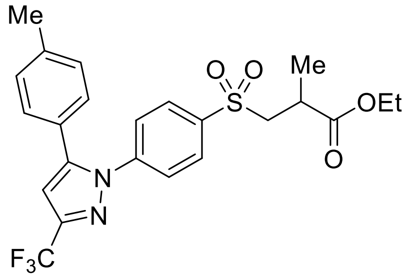

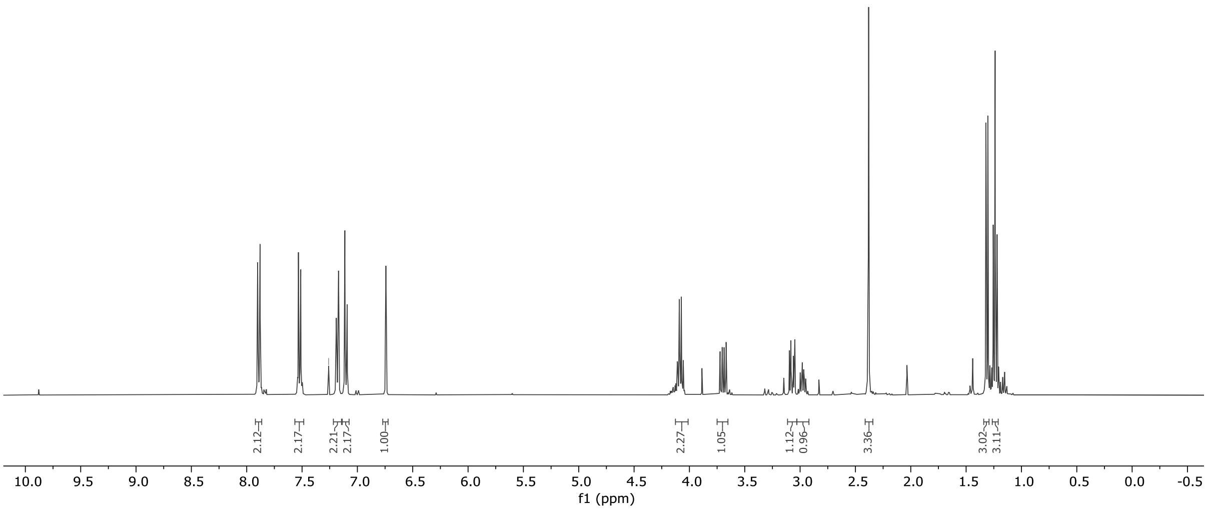

Compound **2ak**
^{13}C NMR
(101 MHz, $CDCl_3$)

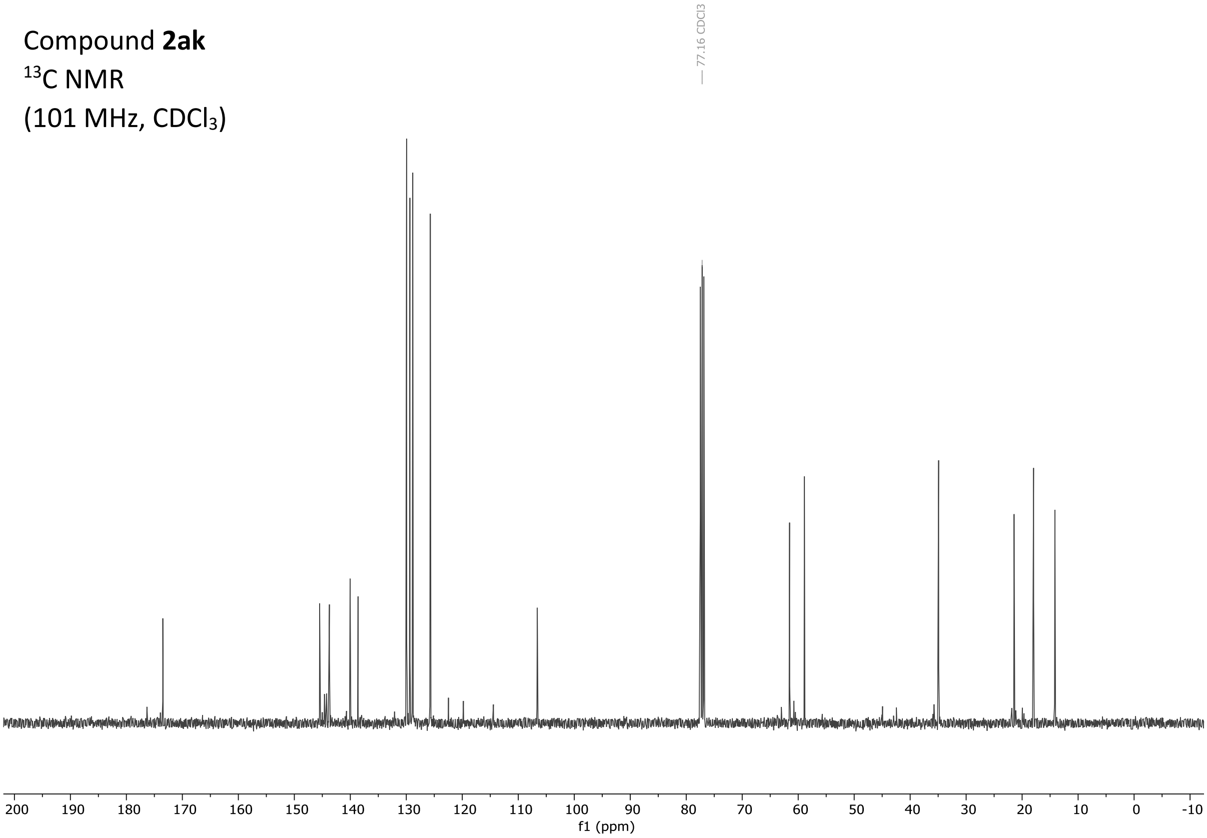

Compound **2ak**
^{19}F NMR
(377 MHz, $CDCl_3$)

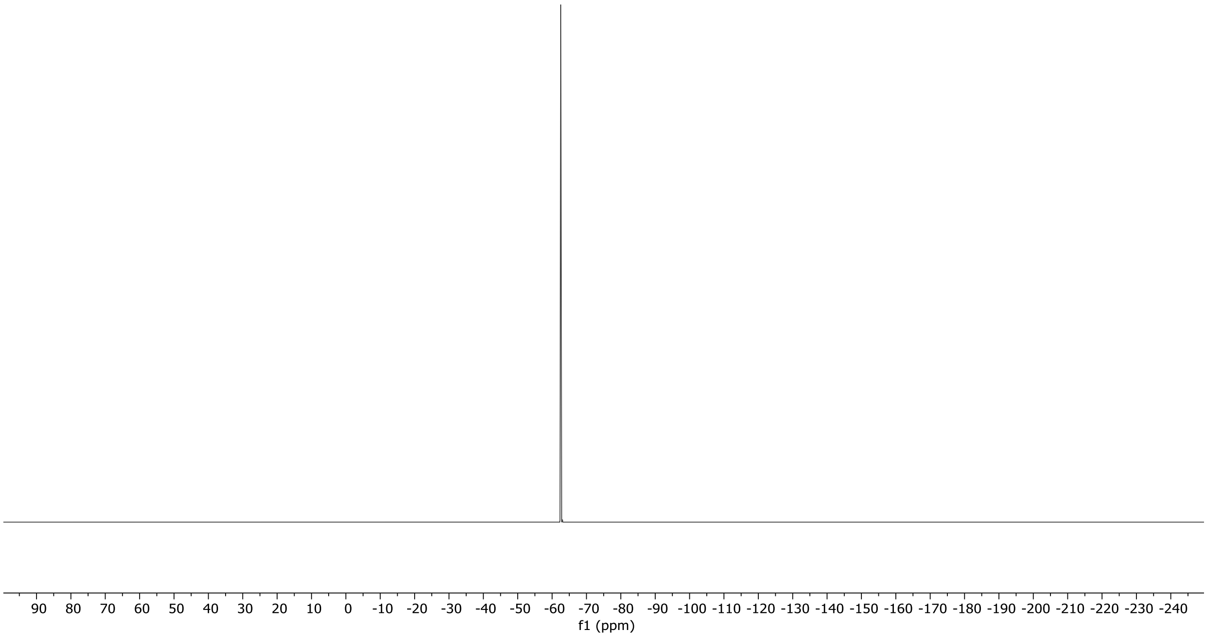

Compound **2al**
^{1}H NMR
(400 MHz, $CDCl_3$)

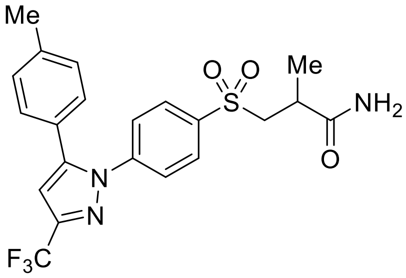

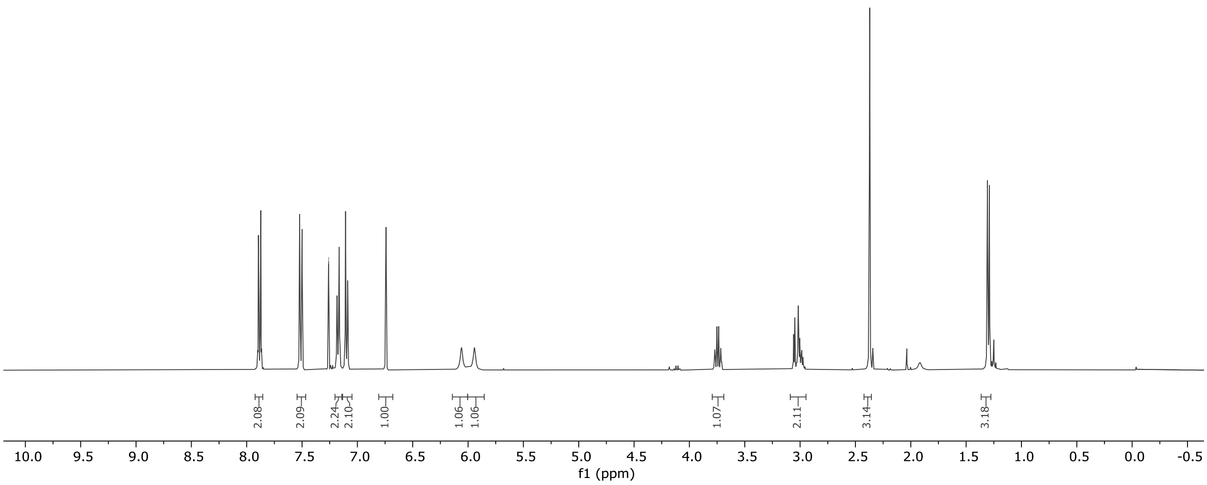

Compound **2al**
^{13}C NMR
(101 MHz, $CDCl_3$)

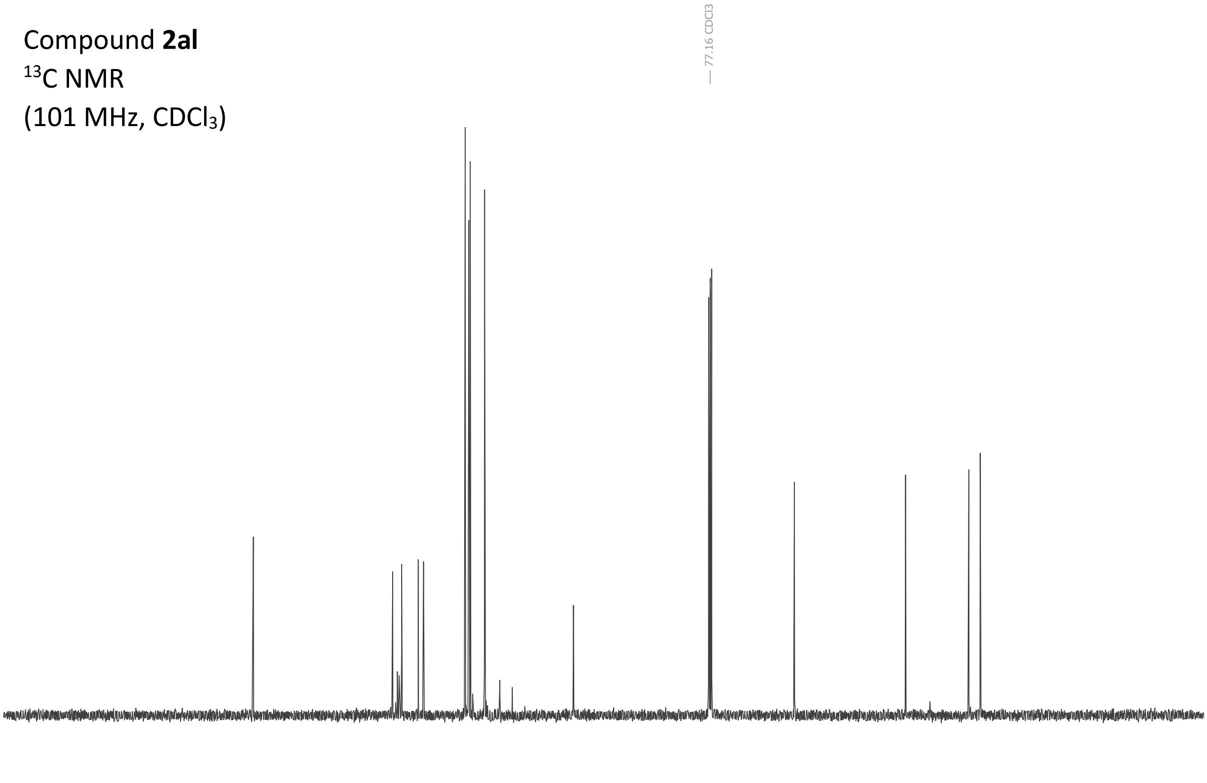

Compound **2al**
^{19}F NMR
(377 MHz, $CDCl_3$)

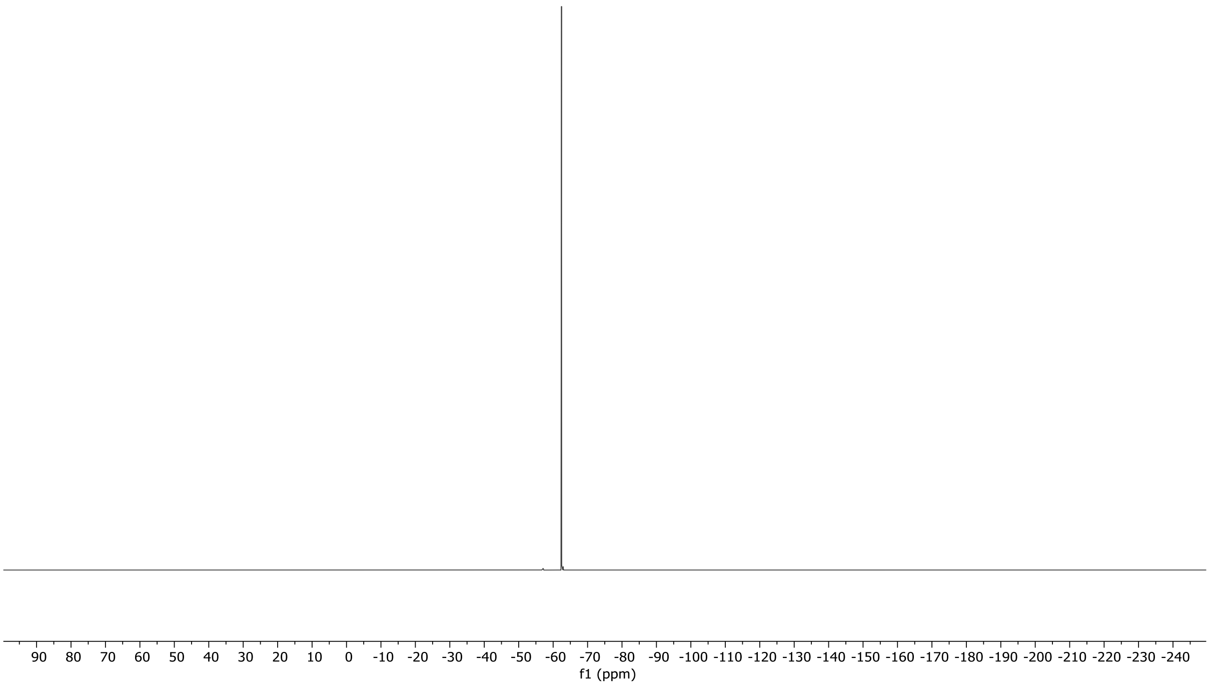

Compound **2am**
^{1}H NMR
(400 MHz, $CDCl_3$)

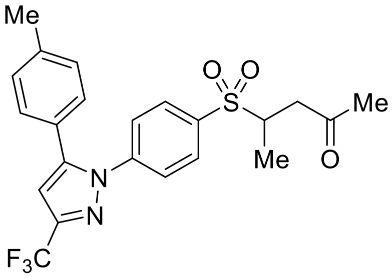

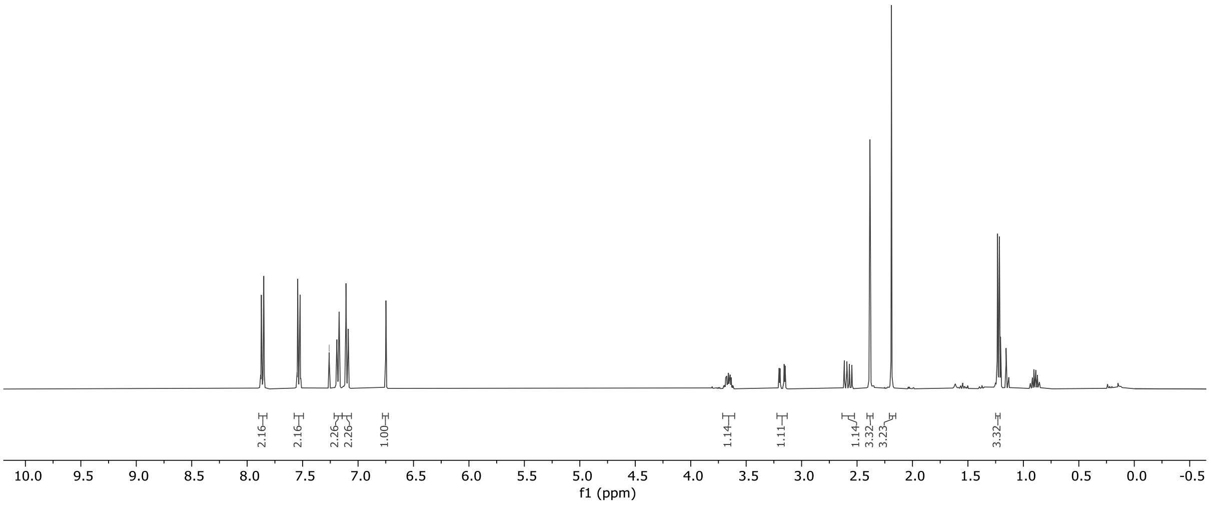

Compound **2am**
^{13}C NMR
(101 MHz, $CDCl_3$)

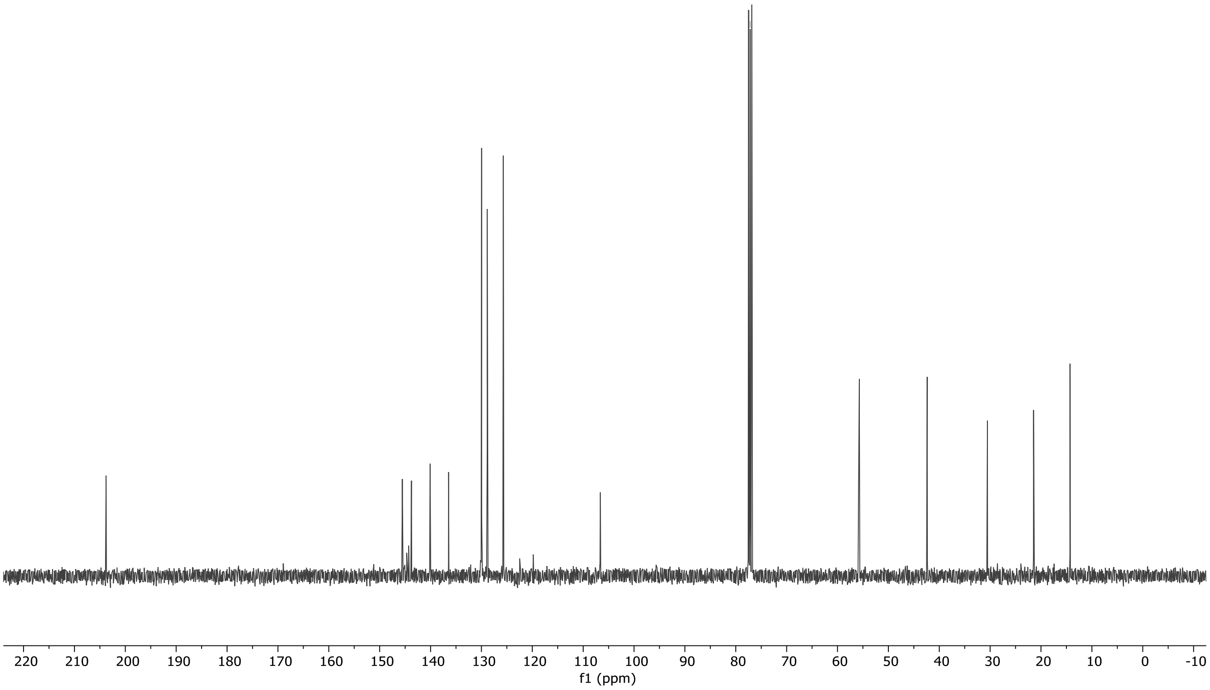

Compound **2am**
^{19}F NMR
(377 MHz, $CDCl_3$)

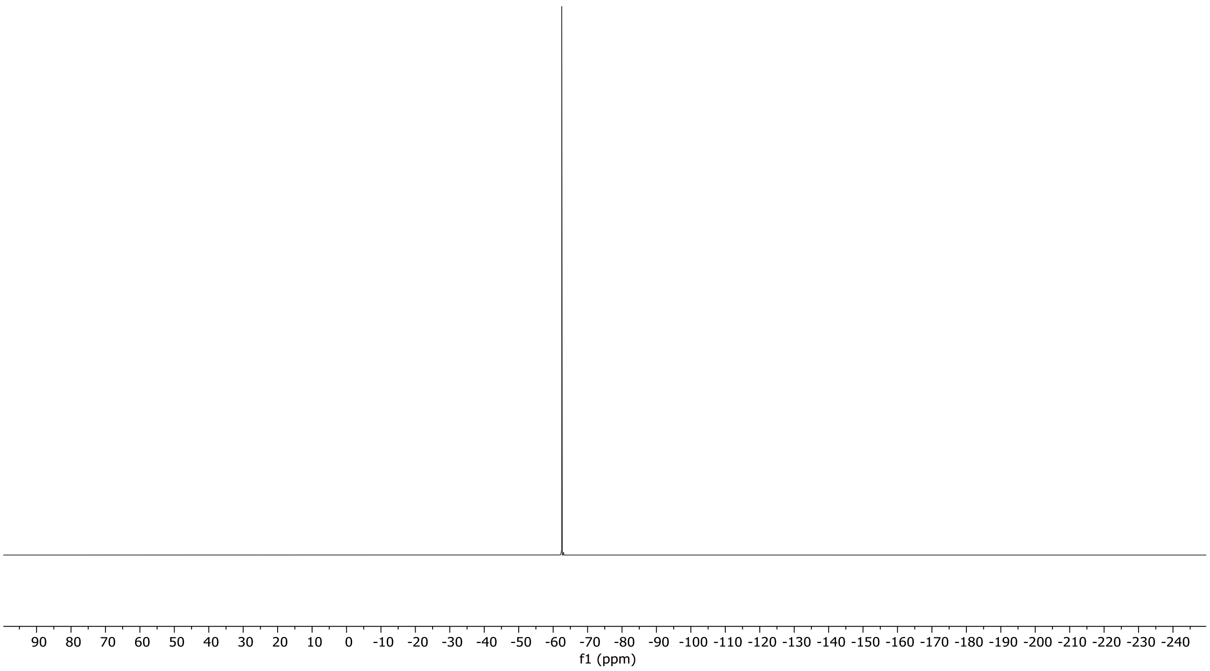

Compound **2an**
^{1}H NMR
(400 MHz, $CDCl_3$)

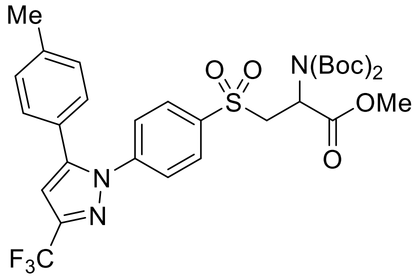

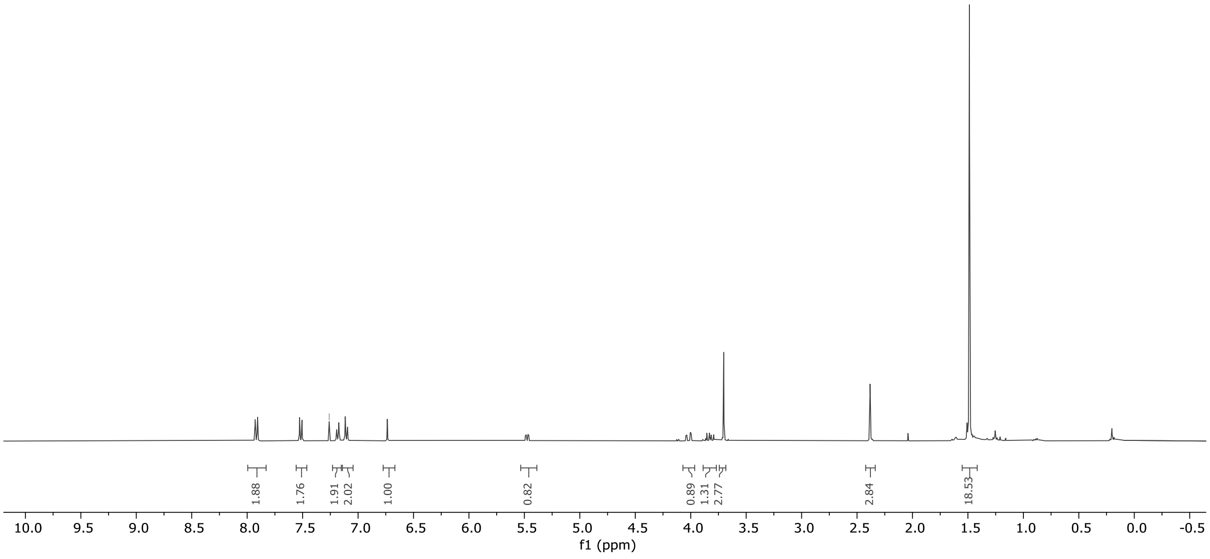

Compound **2an**
^{13}C NMR
(151 MHz, $CDCl_3$)

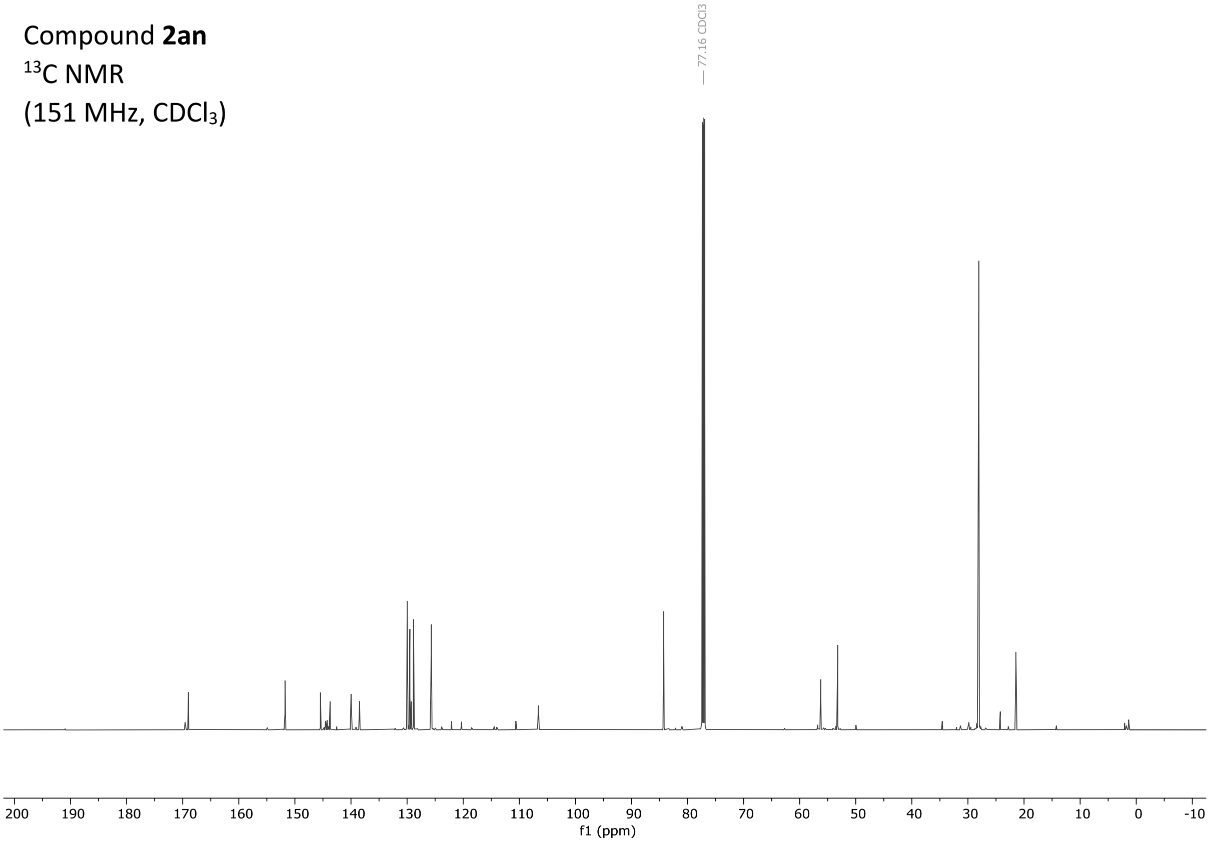

Compound **2an**
^{19}F NMR
(377 MHz, $CDCl_3$)

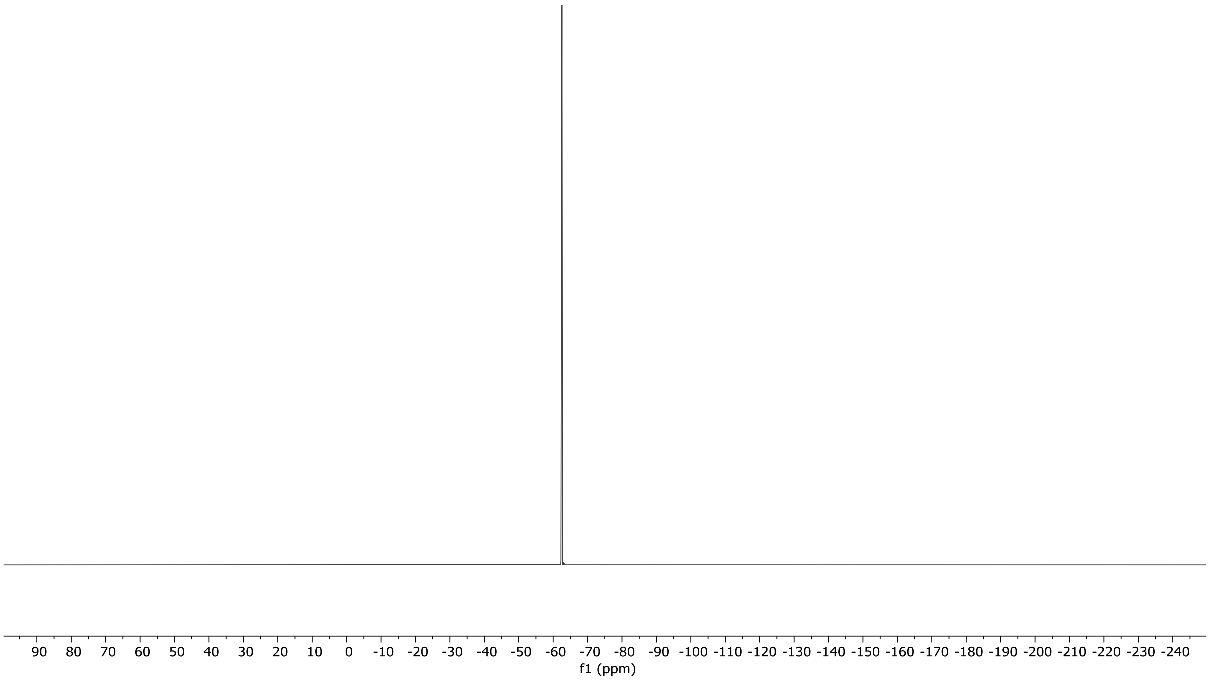

Compound **2ao**
^{1}H NMR
(600 MHz, $CDCl_3$)

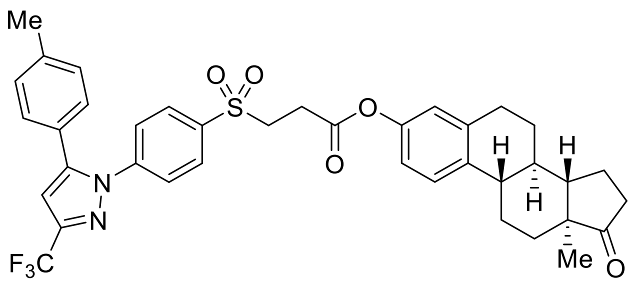

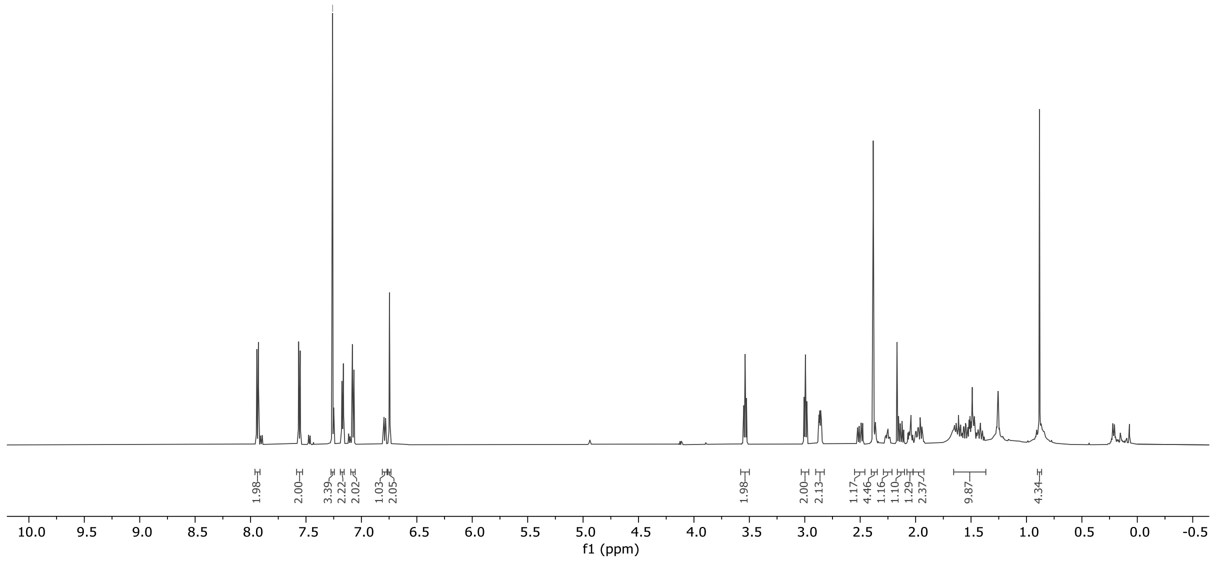

Compound **2ao**
^{13}C NMR
(151 MHz, $CDCl_3$)

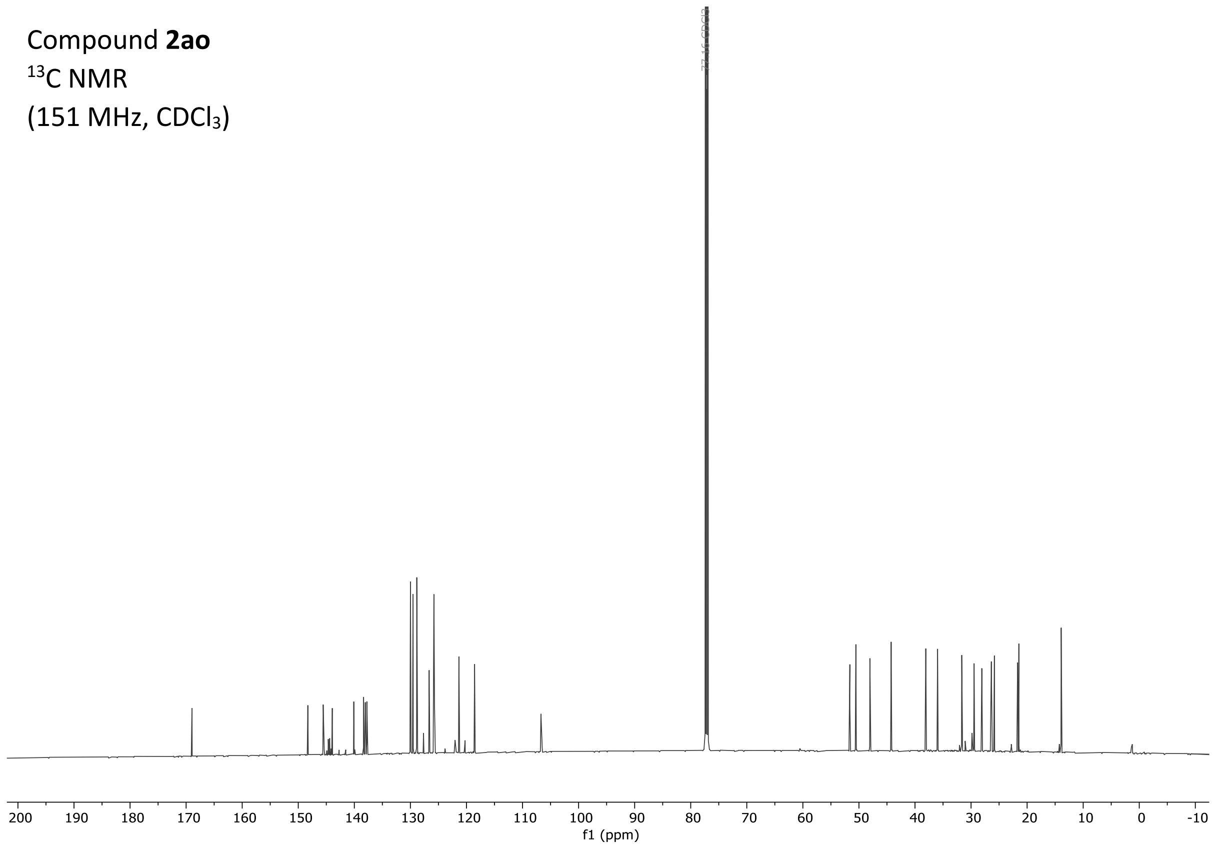

Compound **2ao**
^{19}F NMR
(565 MHz, $CDCl_3$)

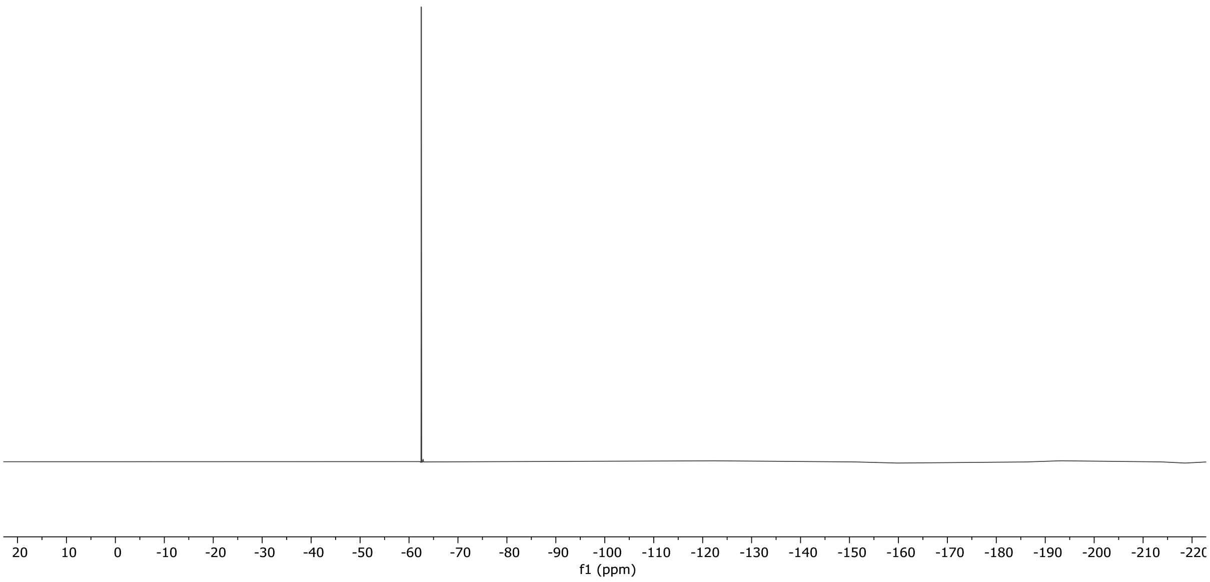

Compound **2ap**
^{1}H NMR
(600 MHz, $CDCl_3$)

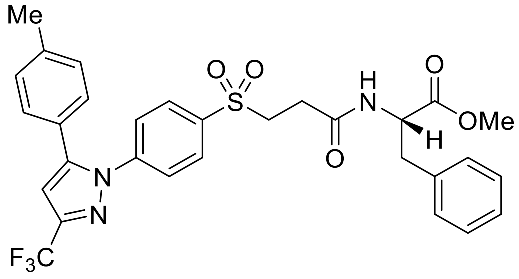

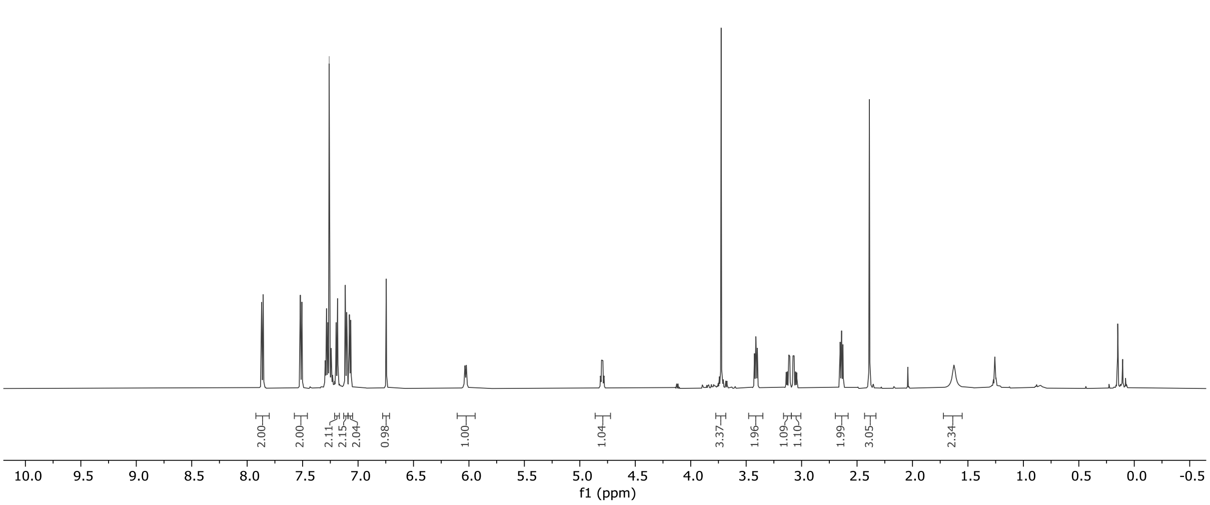

Compound **2ap**
^{13}C NMR
(151 MHz, $CDCl_3$)

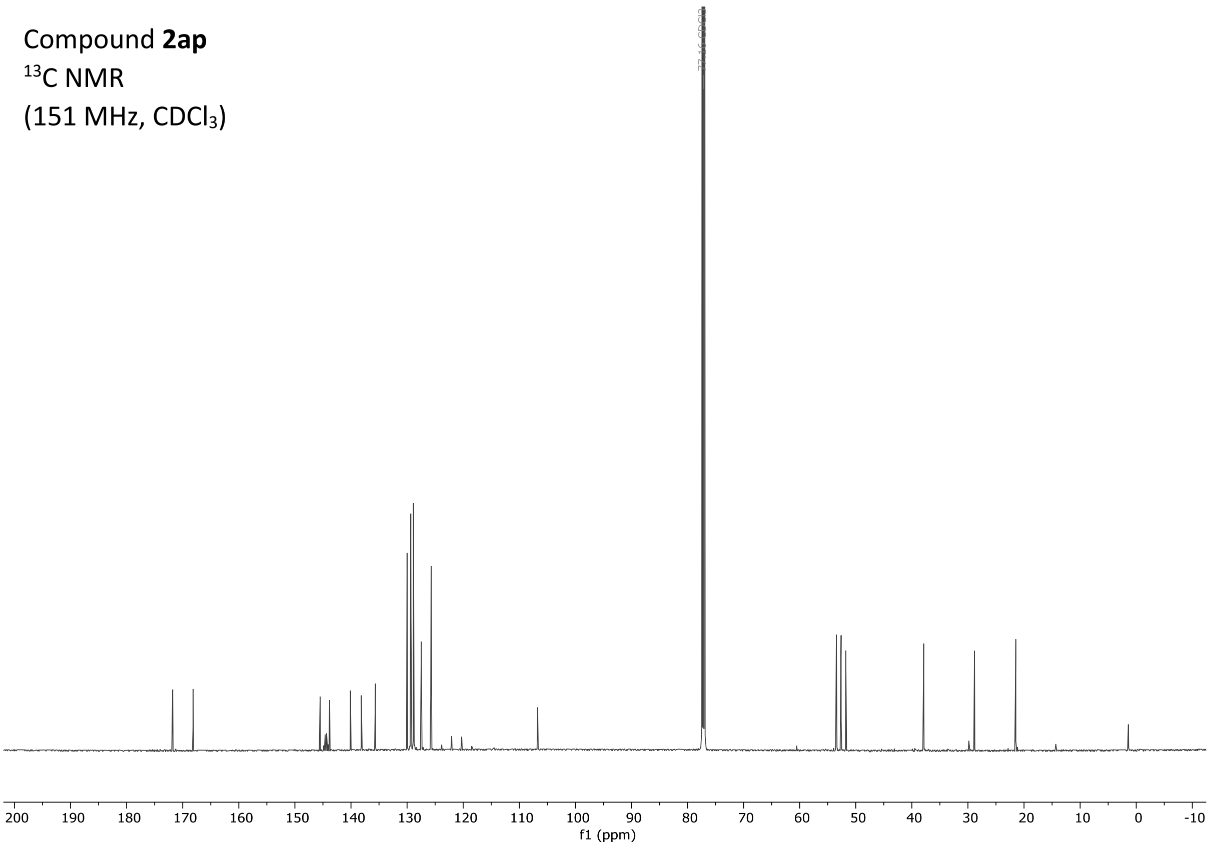

Compound **2ap**
^{19}F NMR
(565 MHz, $CDCl_3$)

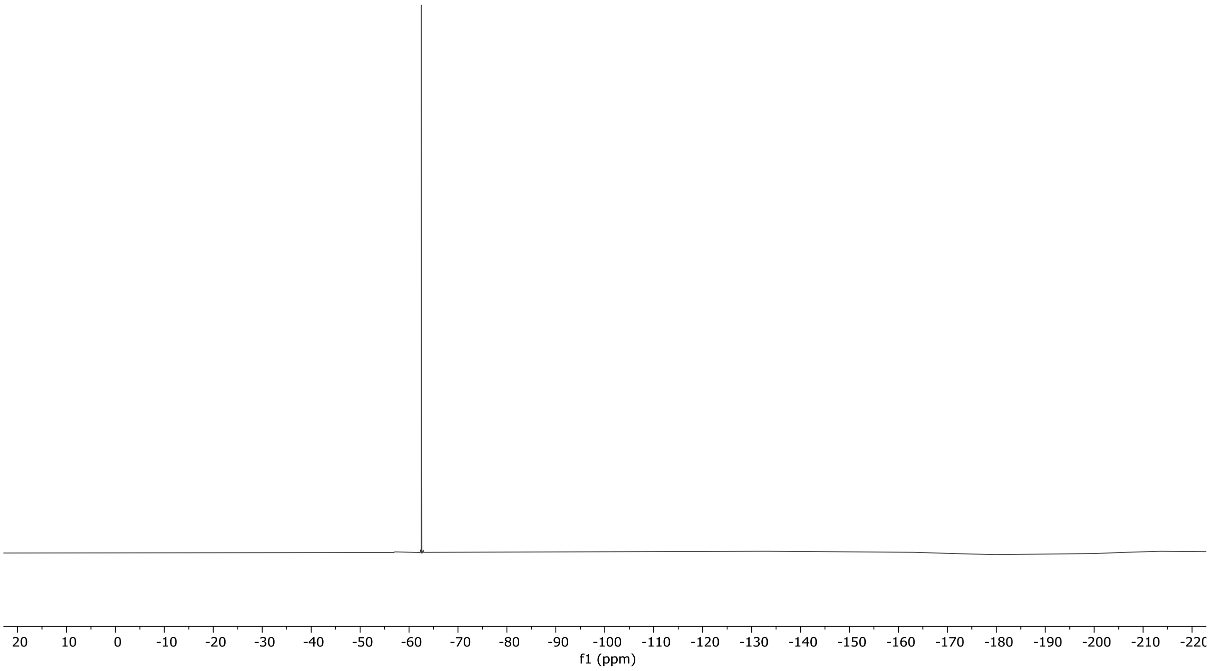

Compound **2aq**
^{1}H NMR
(600 MHz, $CDCl_3$)

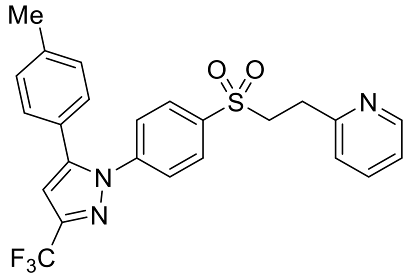

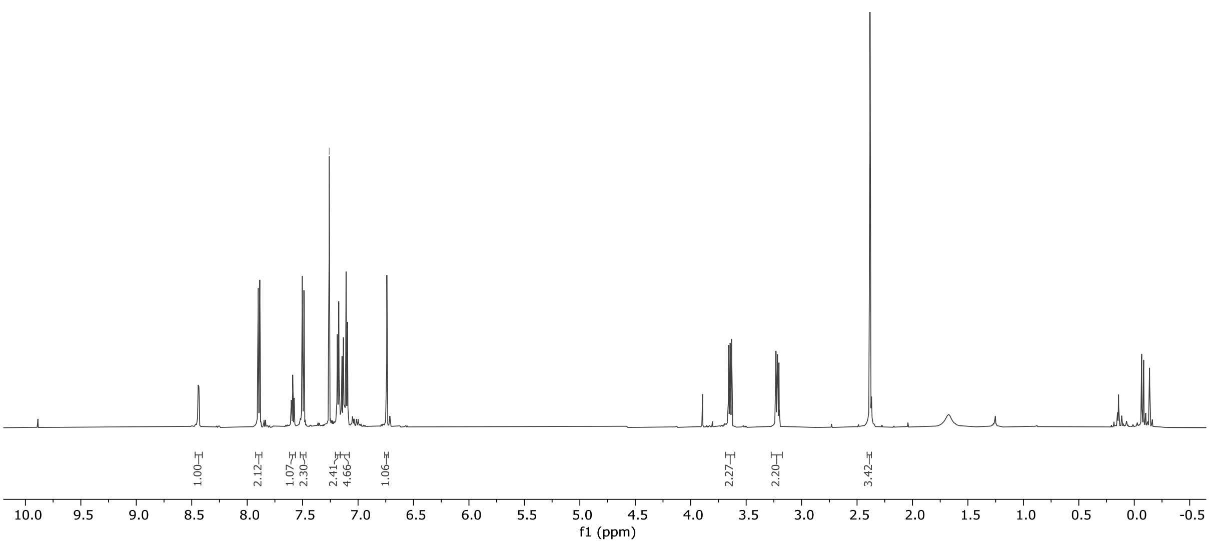

Compound **2aq**
^{13}C NMR
(151 MHz, $CDCl_3$)

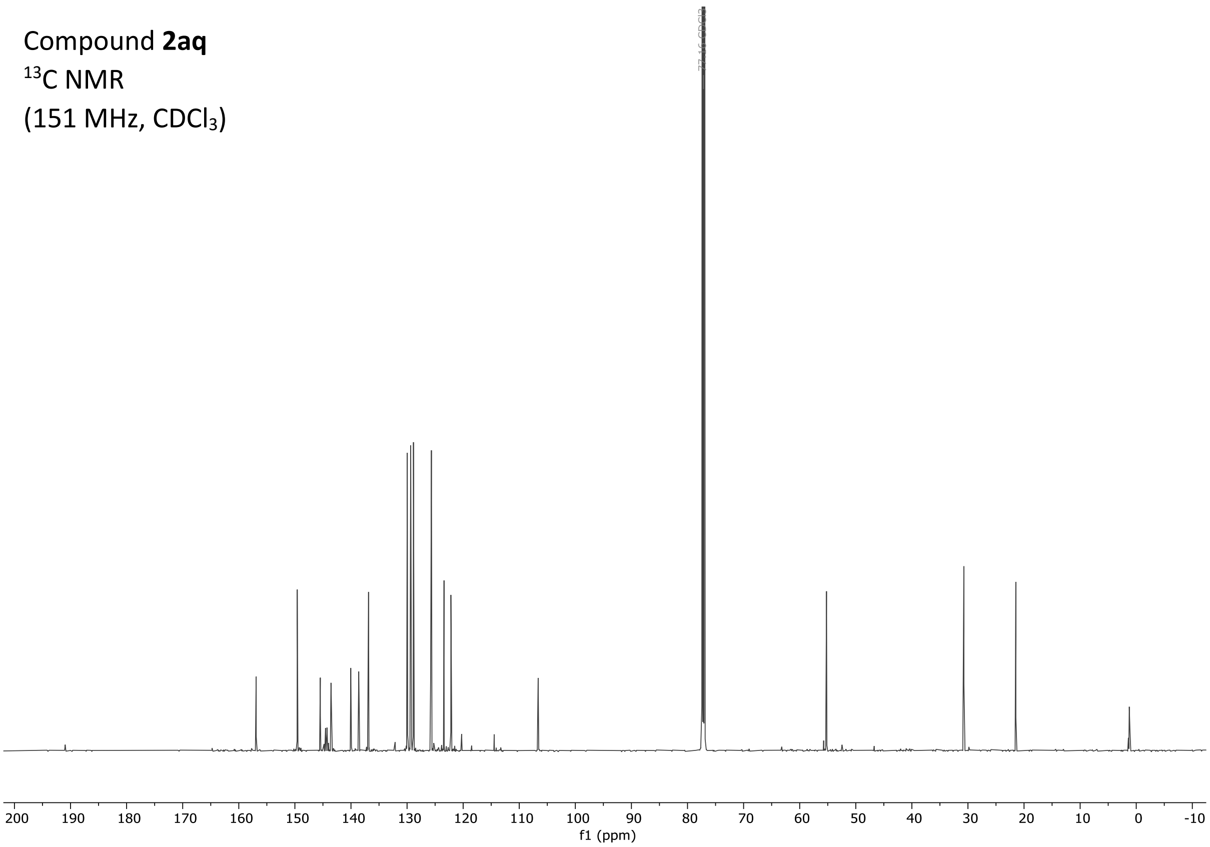

Compound **2aq**
^{19}F NMR
(565 MHz, $CDCl_3$)

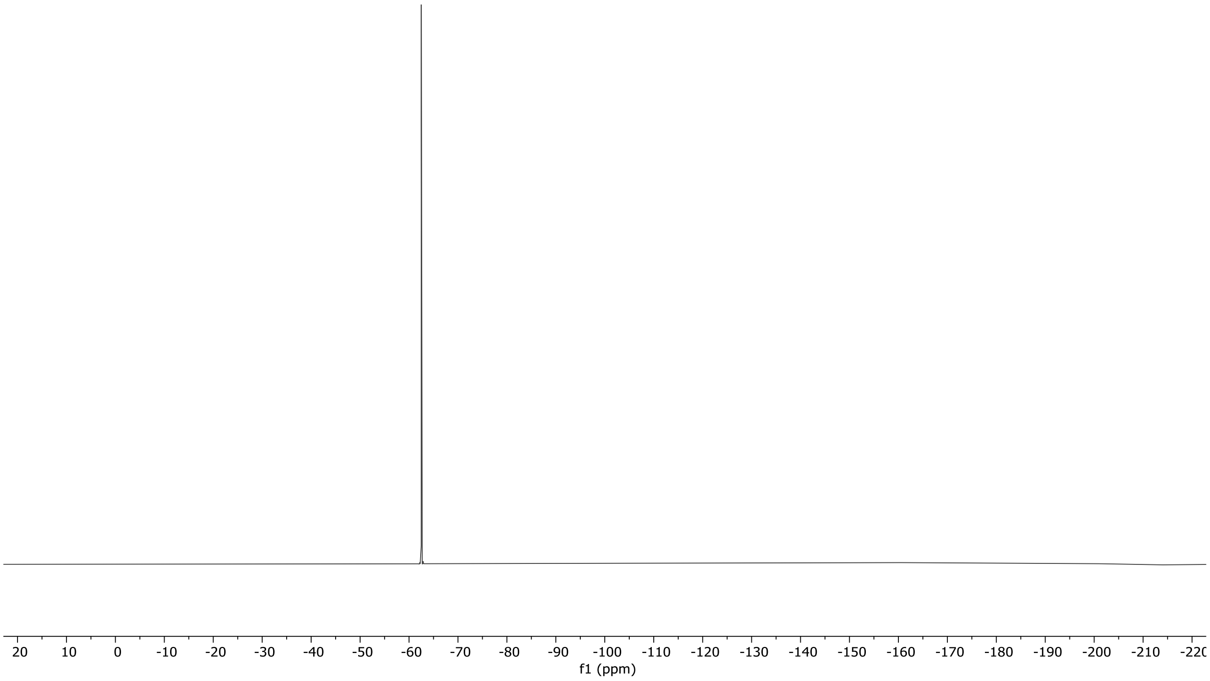

Compound **2ar**
^{1}H NMR
(400 MHz, $CDCl_3$)

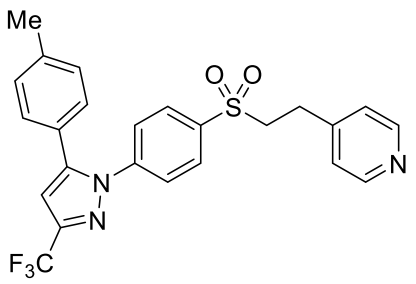

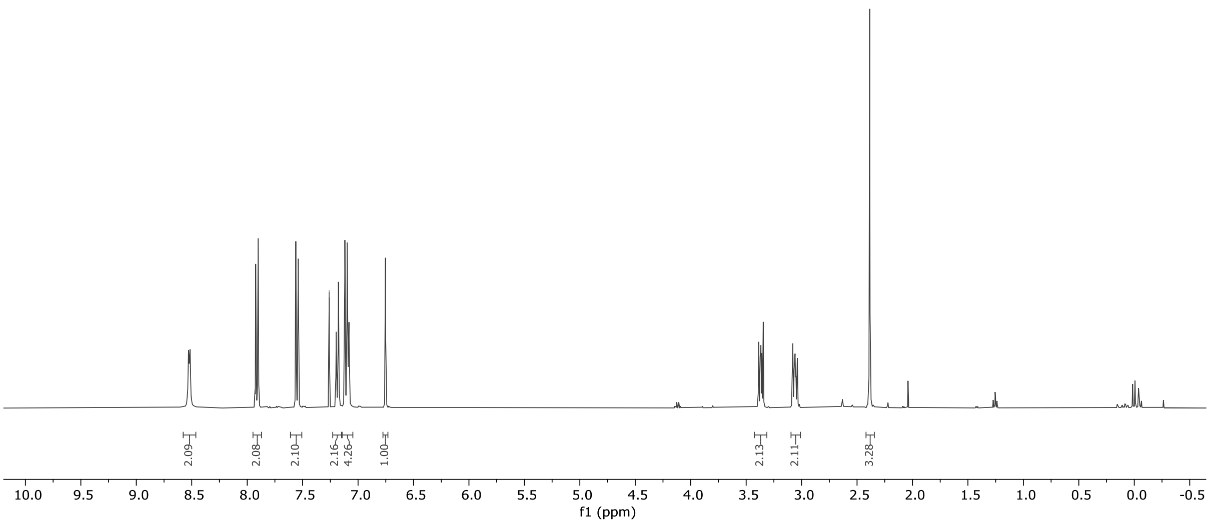

Compound **2ar**
^{13}C NMR
(126 MHz, $CDCl_3$)

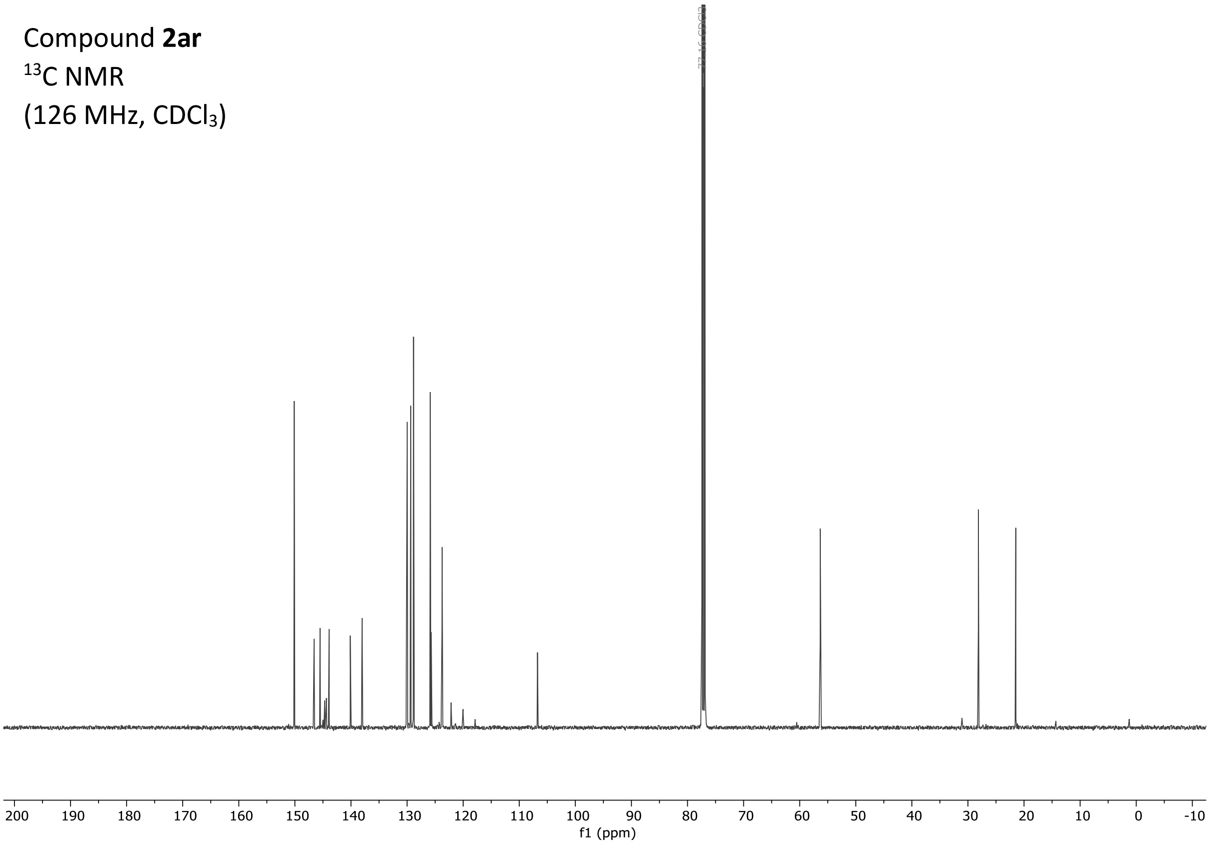

Compound **2ar**
^{19}F NMR
(377 MHz, $CDCl_3$)

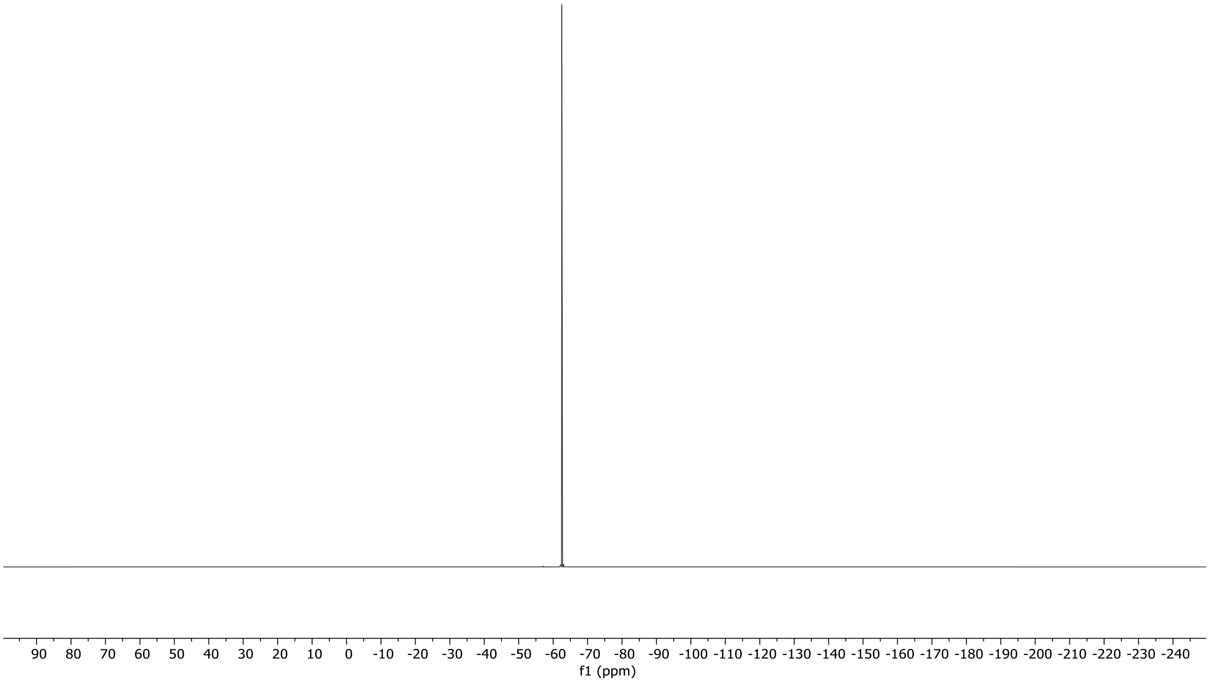

Compound **2as**
^{1}H NMR
(600 MHz, $CDCl_3$)

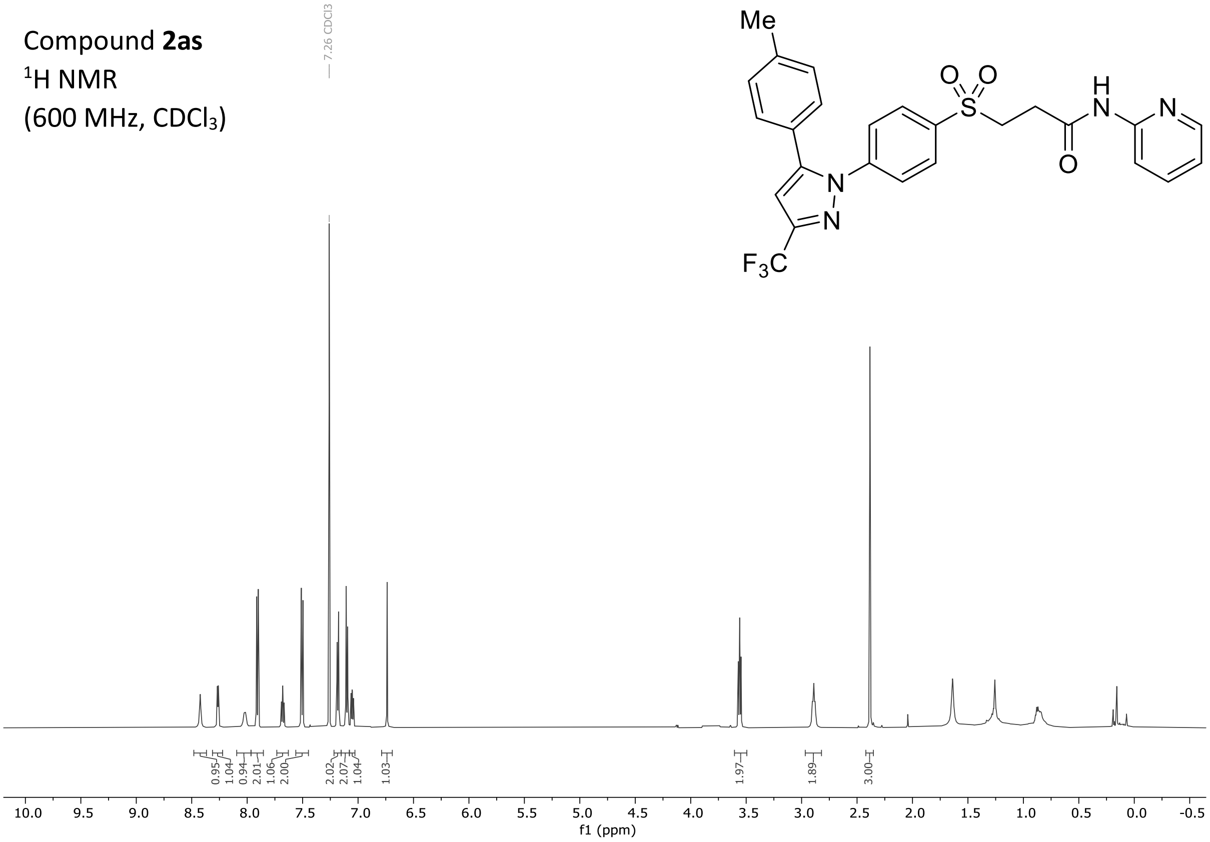

Compound **2as**
^{13}C NMR
(151 MHz, $CDCl_3$)

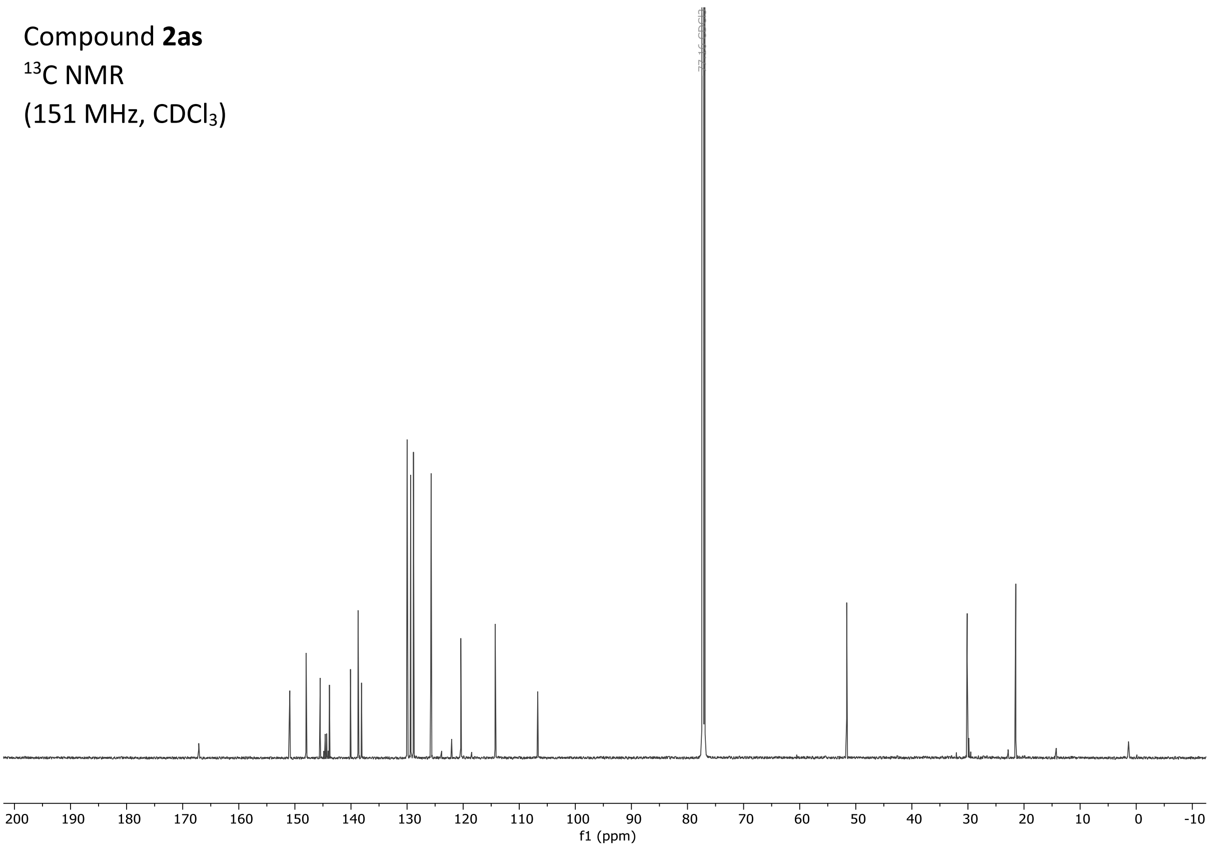

Compound **2as**
^{19}F NMR
(565 MHz, $CDCl_3$)

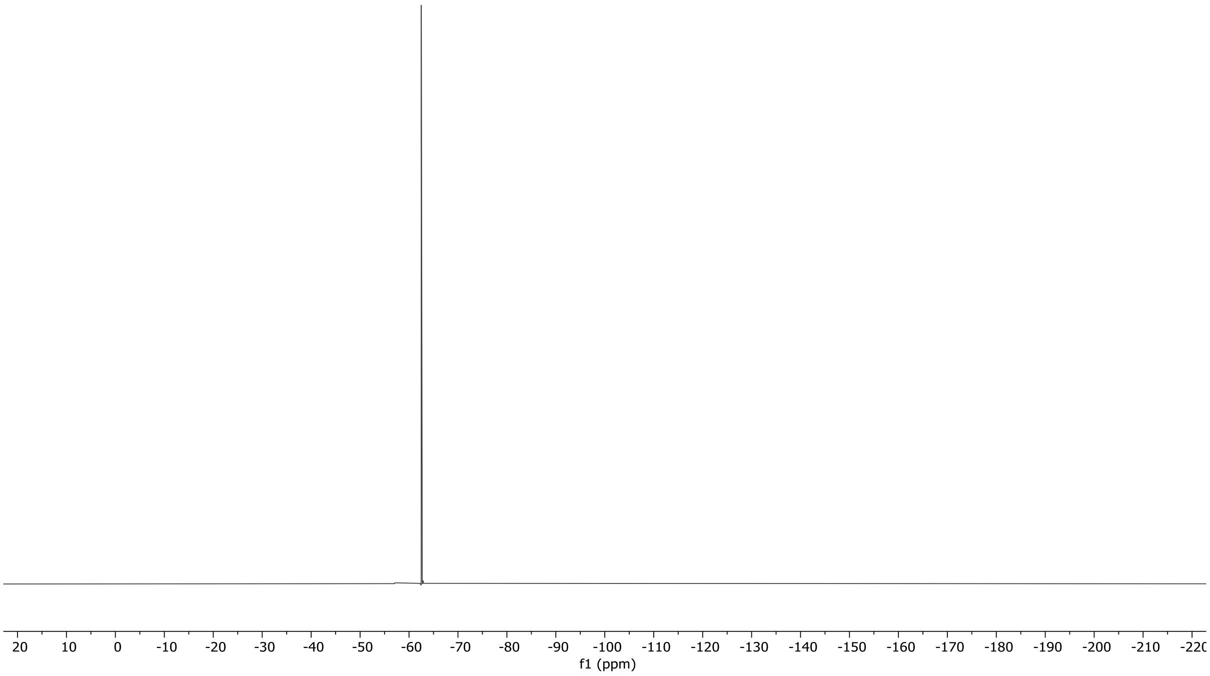

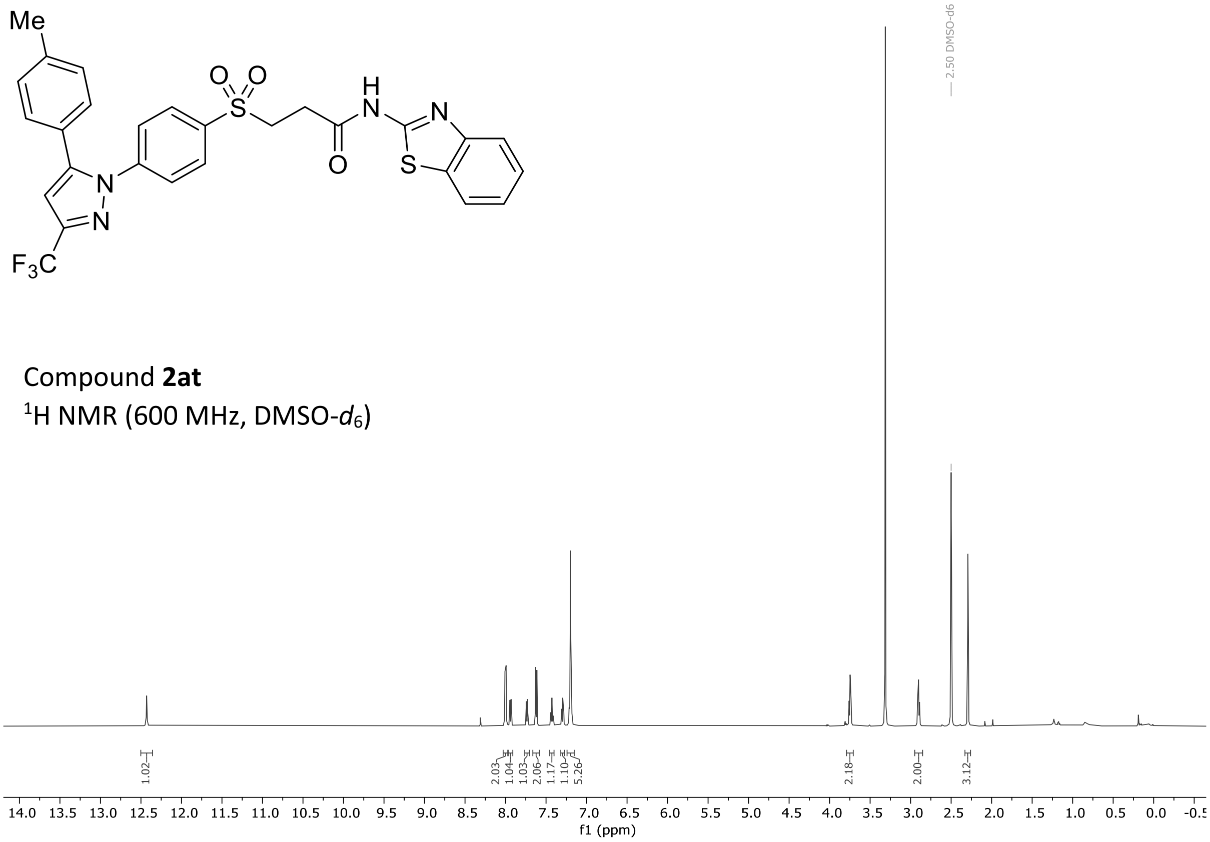

Compound **2at**

^{1}H NMR (600 MHz, DMSO-d_6)

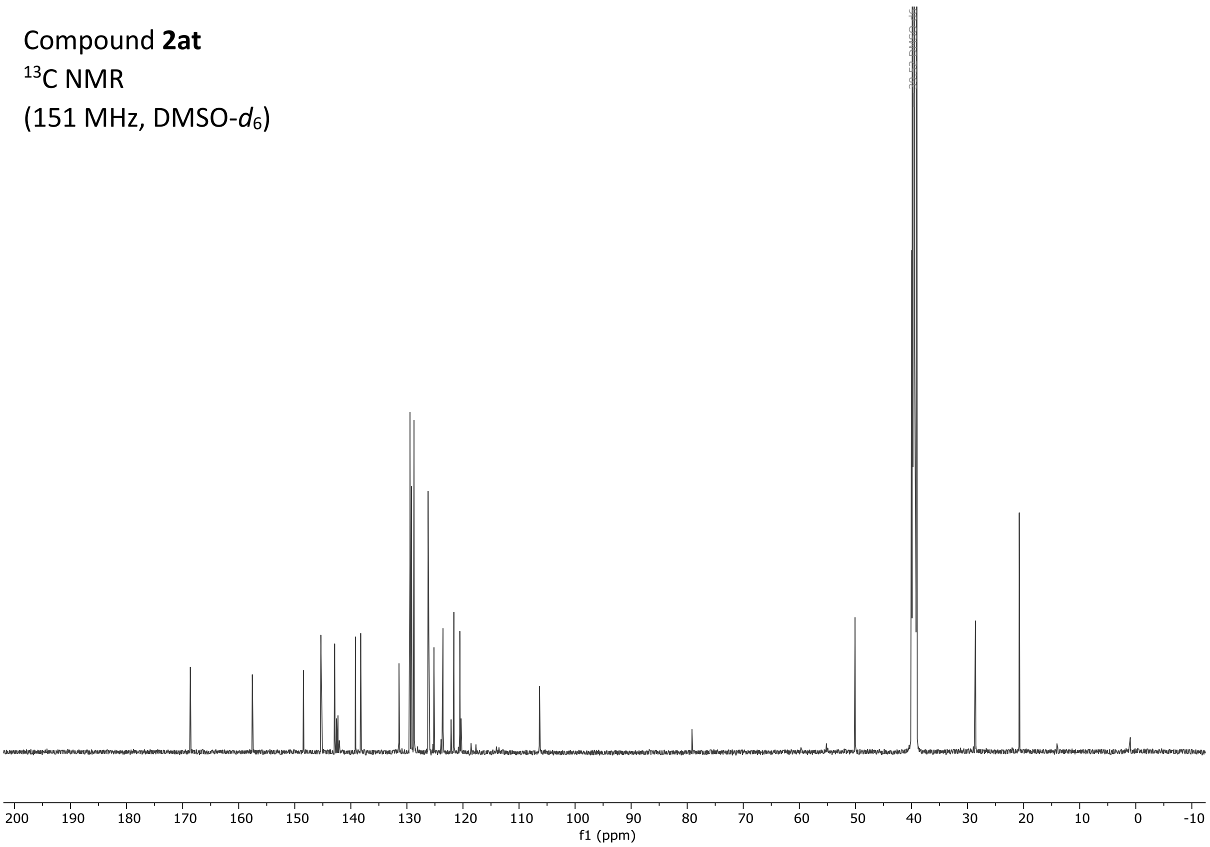

Compound **2at**

^{13}C NMR

(151 MHz, DMSO-d_6)

Compound **2at**
^{19}F NMR
(565 MHz, DMSO-d_6)

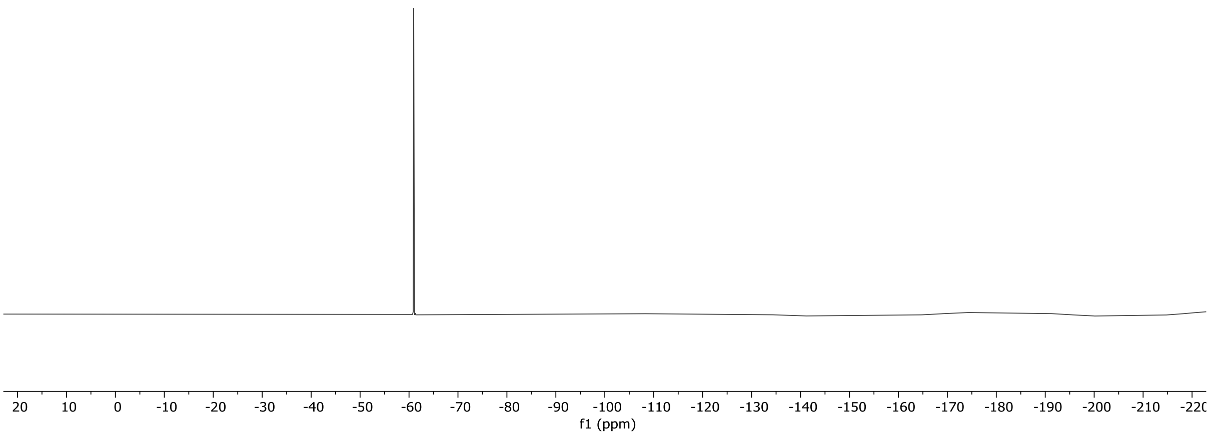

Compound **2au**
^{1}H NMR
(600 MHz, $CDCl_3$)

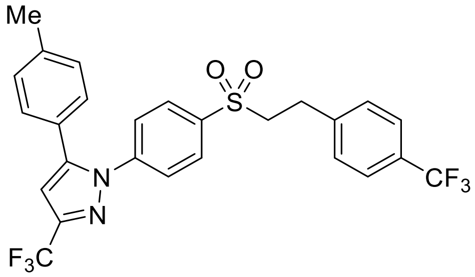

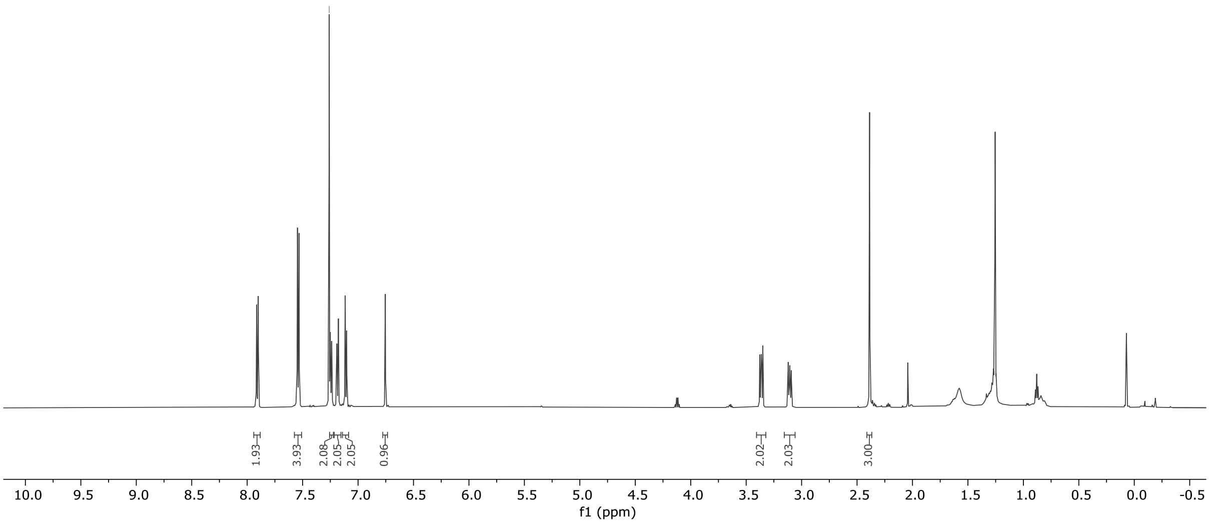

Compound **2au**
^{13}C NMR
(151 MHz, $CDCl_3$)

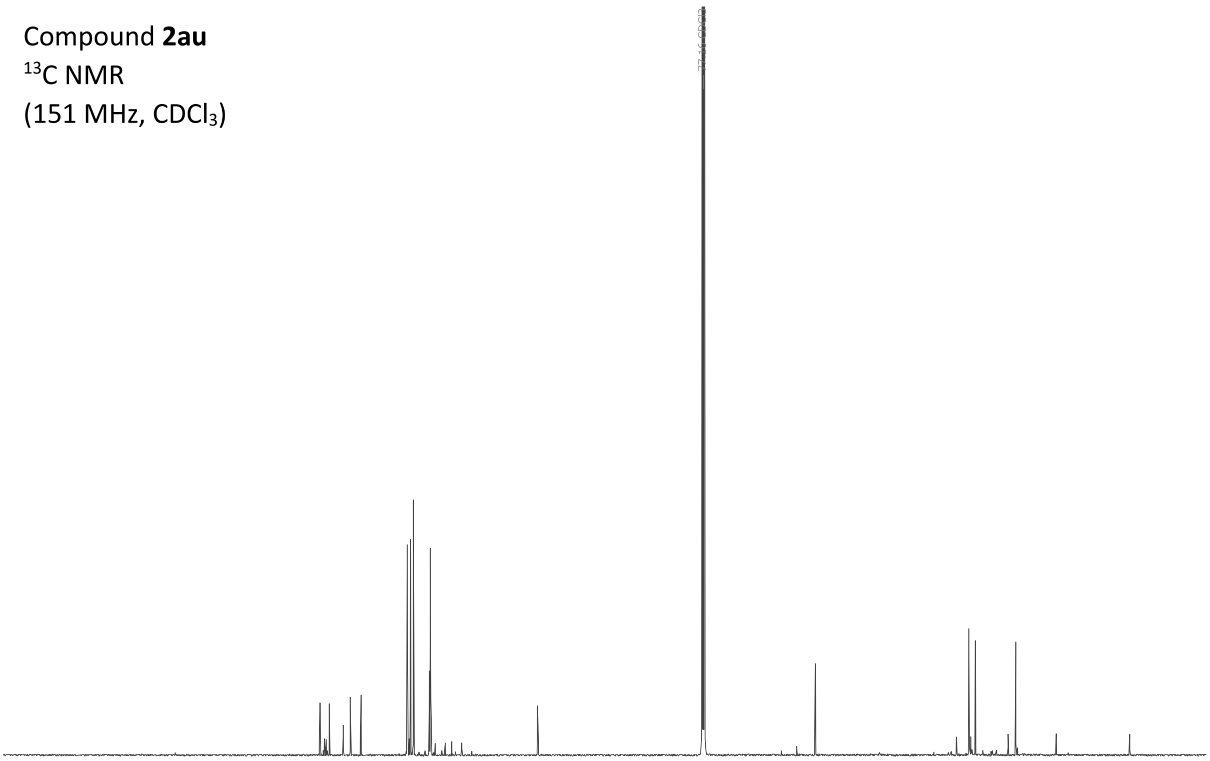

Compound **2au**
^{19}F NMR
(565 MHz, $CDCl_3$)

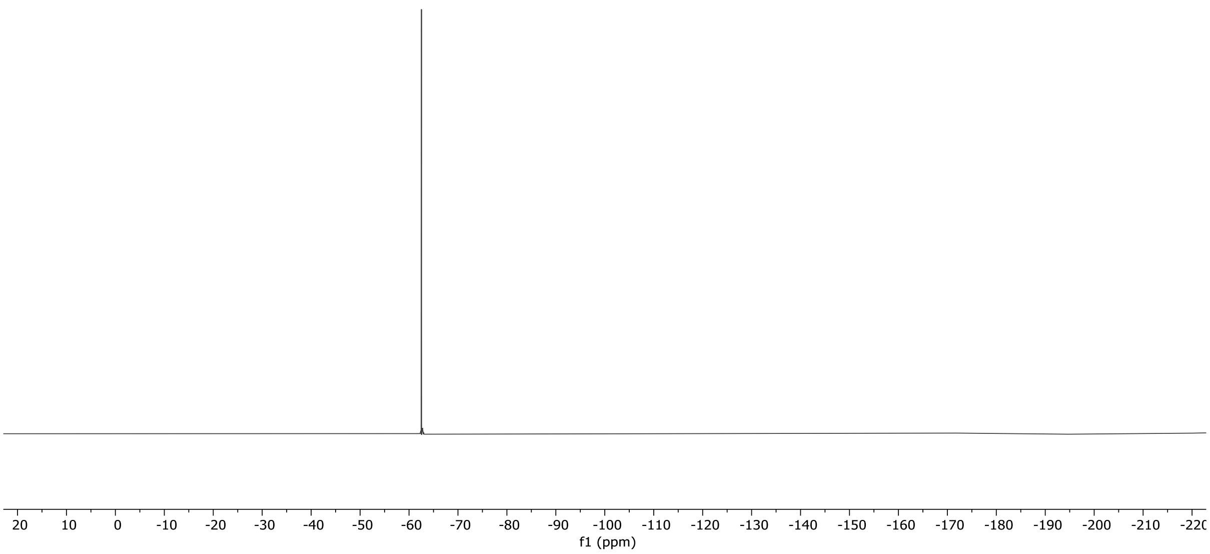

Compound **4a**
^{1}H NMR
(600 MHz, $CDCl_3$)

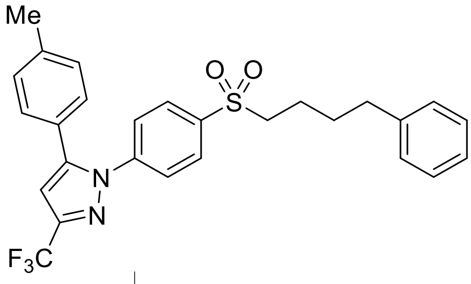

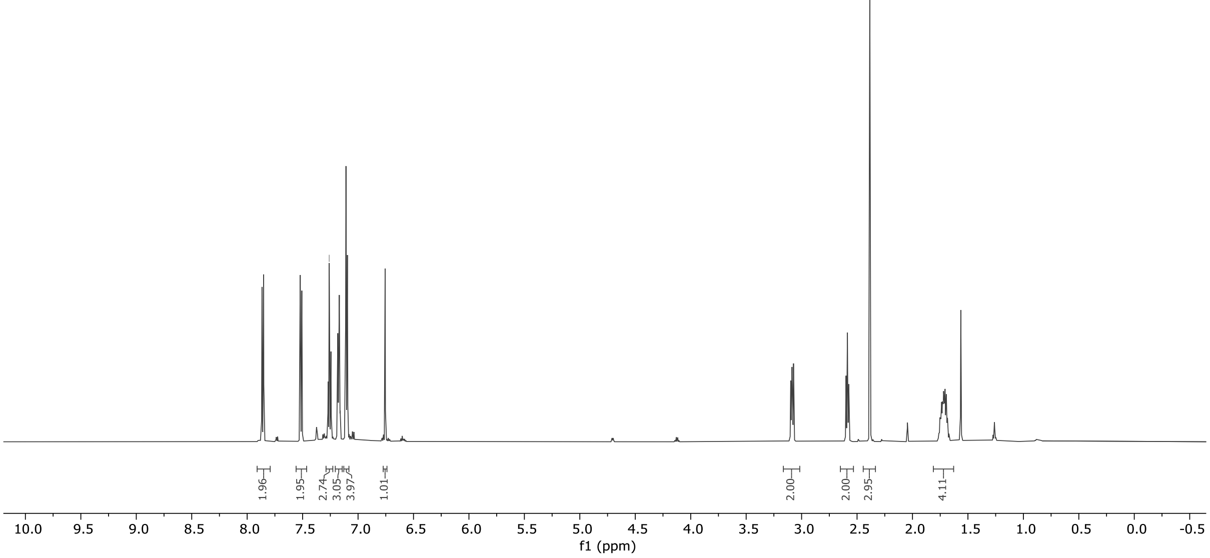

Compound **4a**
^{13}C NMR
(151 MHz, $CDCl_3$)

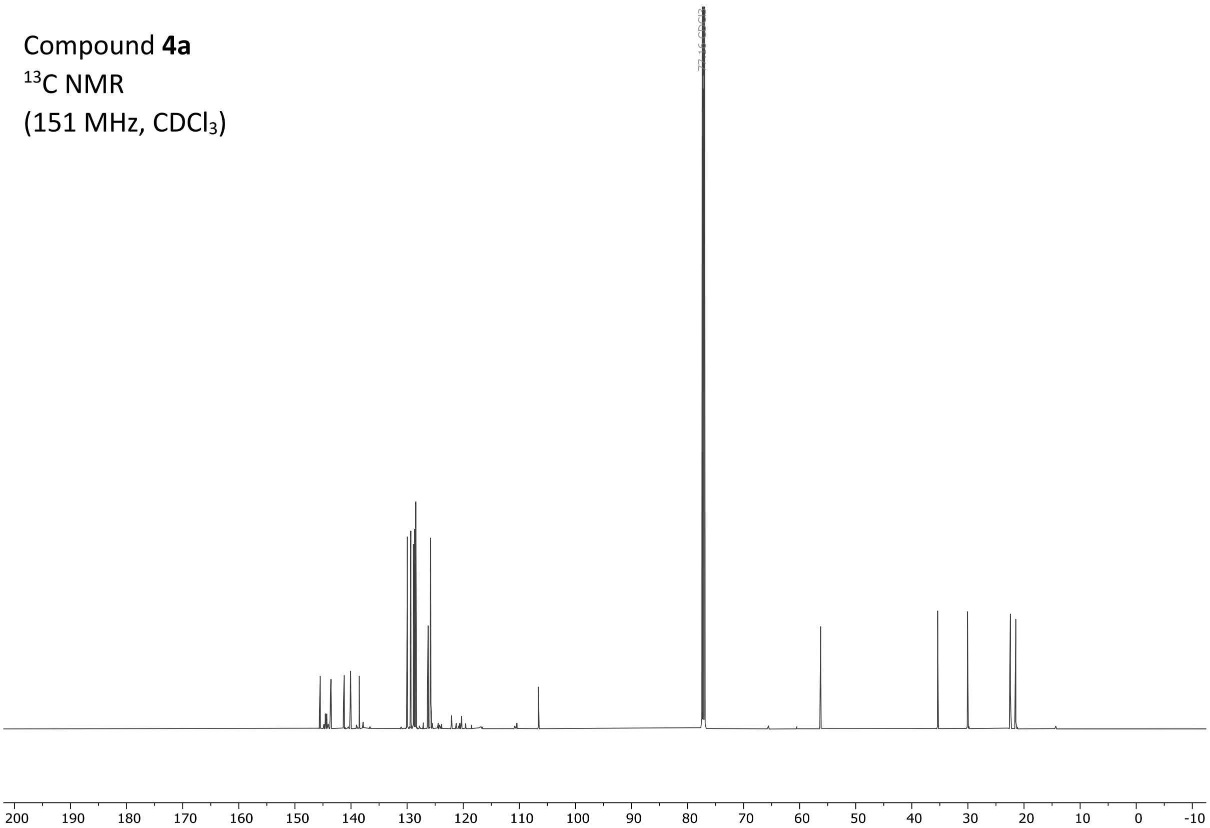

Compound **4a**
^{19}F NMR
(377 MHz, $CDCl_3$)

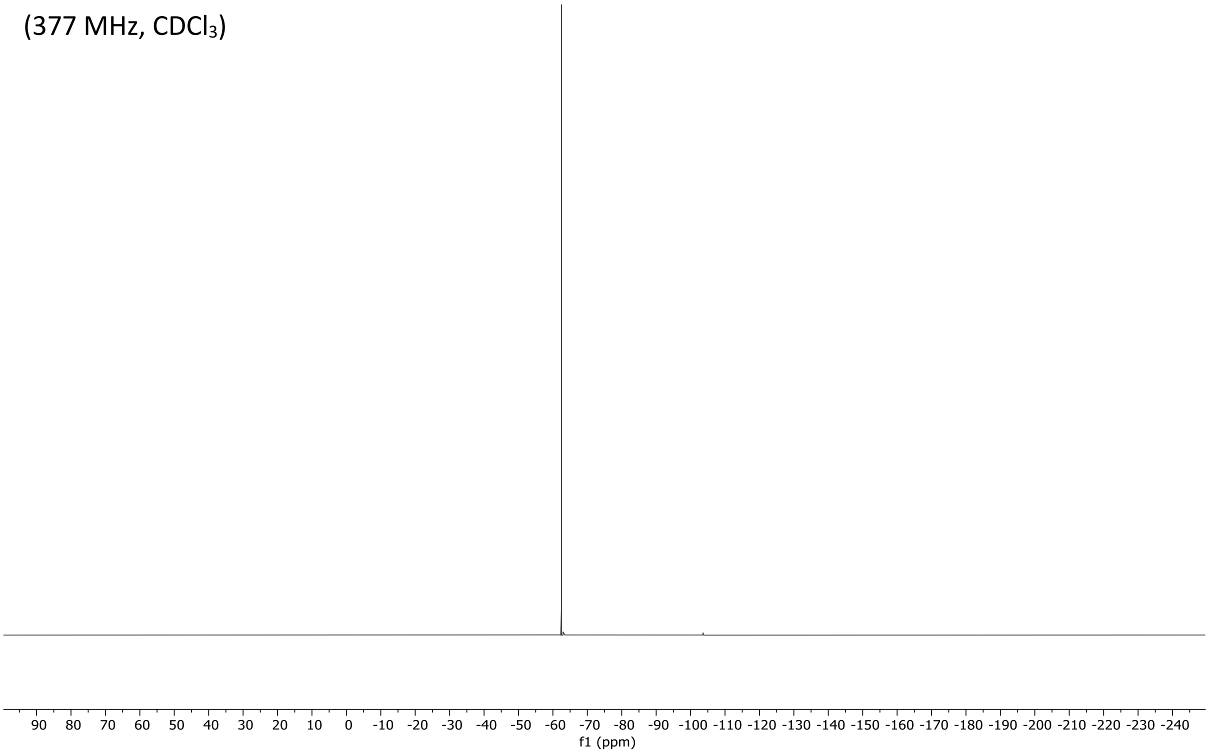

Compound **4b**
^{1}H NMR
(600 MHz, $CDCl_3$)

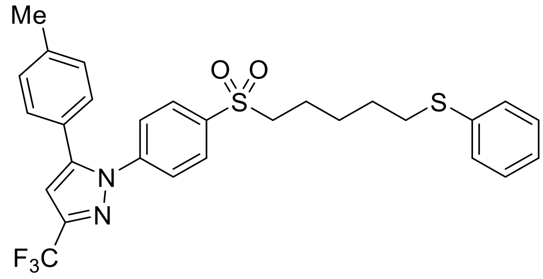

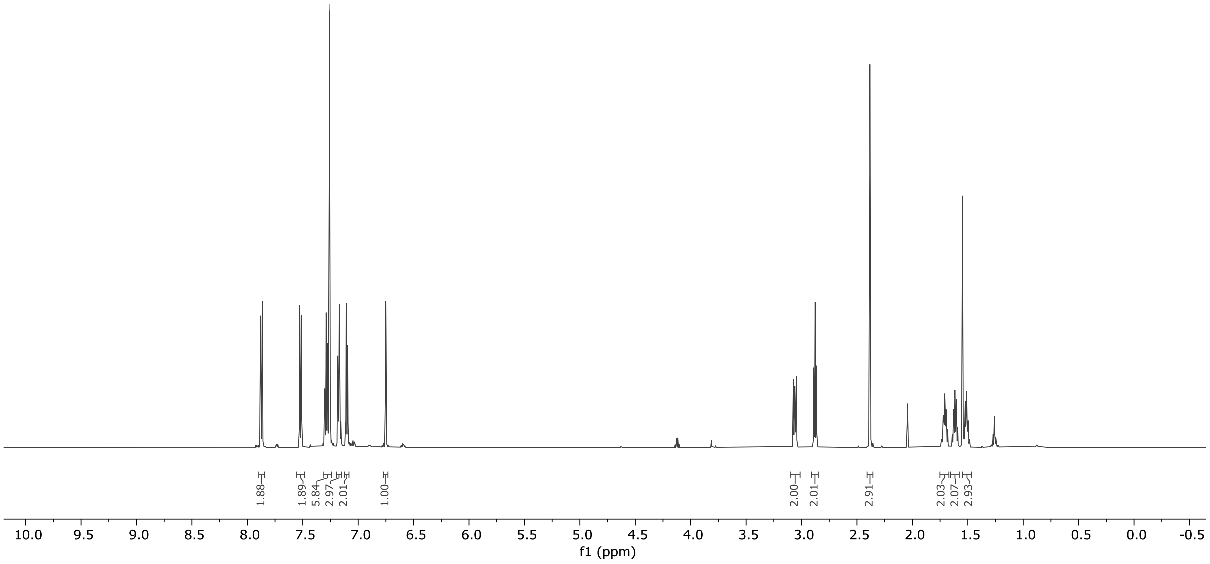

Compound **4b**
^{13}C NMR
(151 MHz, $CDCl_3$)

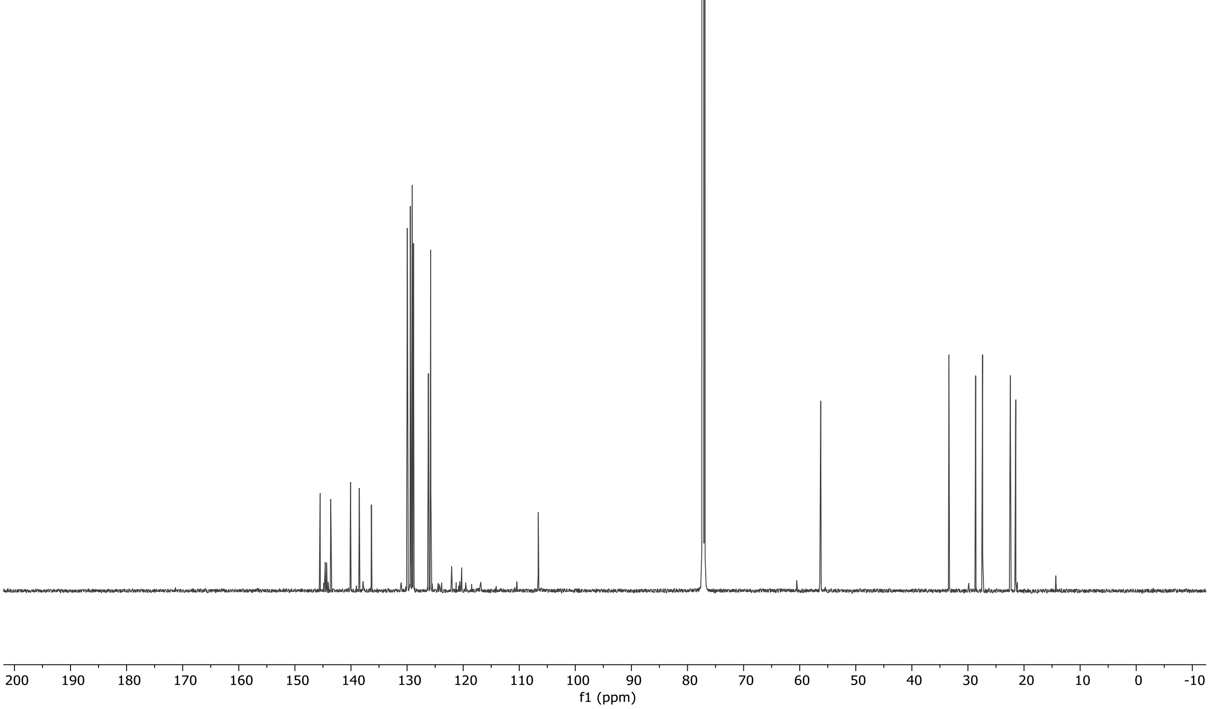

Compound **4b**
^{19}F NMR
(377 MHz, $CDCl_3$)

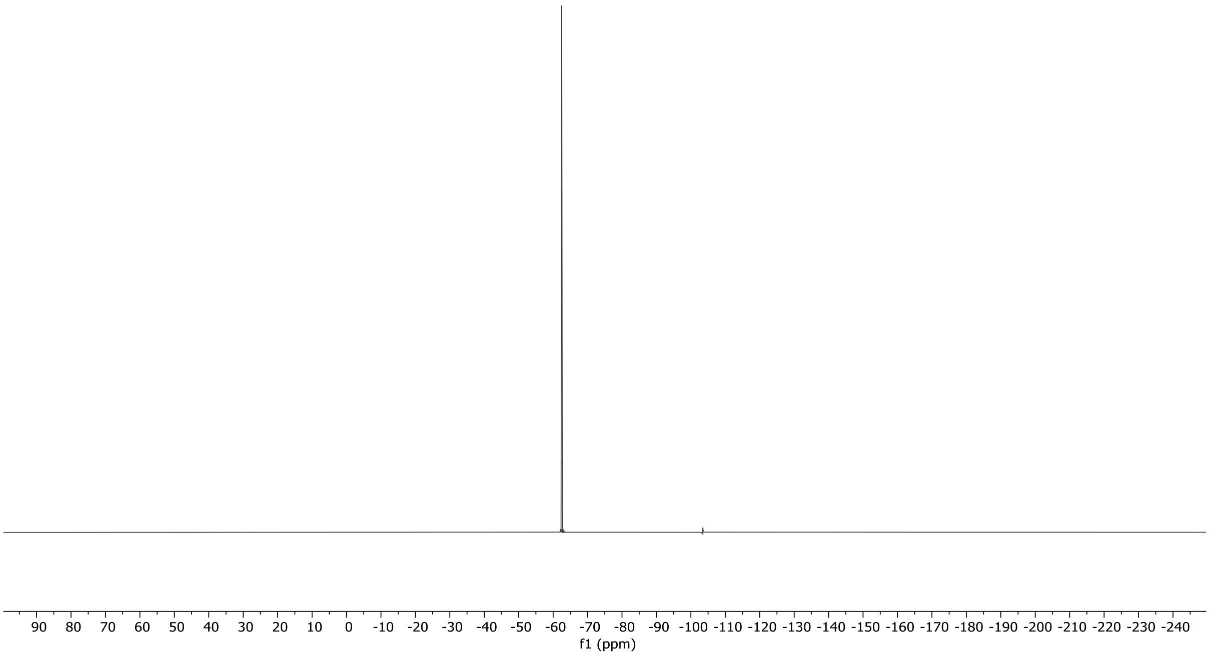

Compound **4c**
^{1}H NMR
(600 MHz, $CDCl_3$)

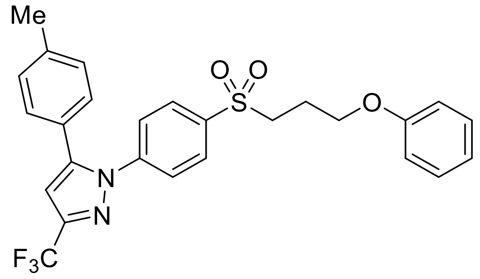

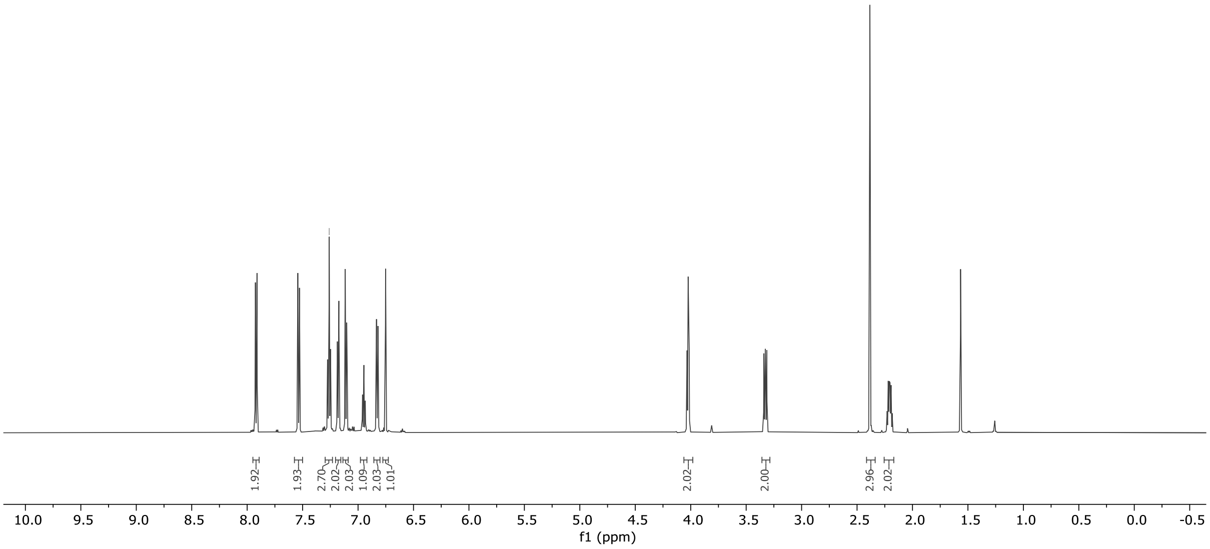

Compound **4c**
^{13}C NMR
(151 MHz, $CDCl_3$)

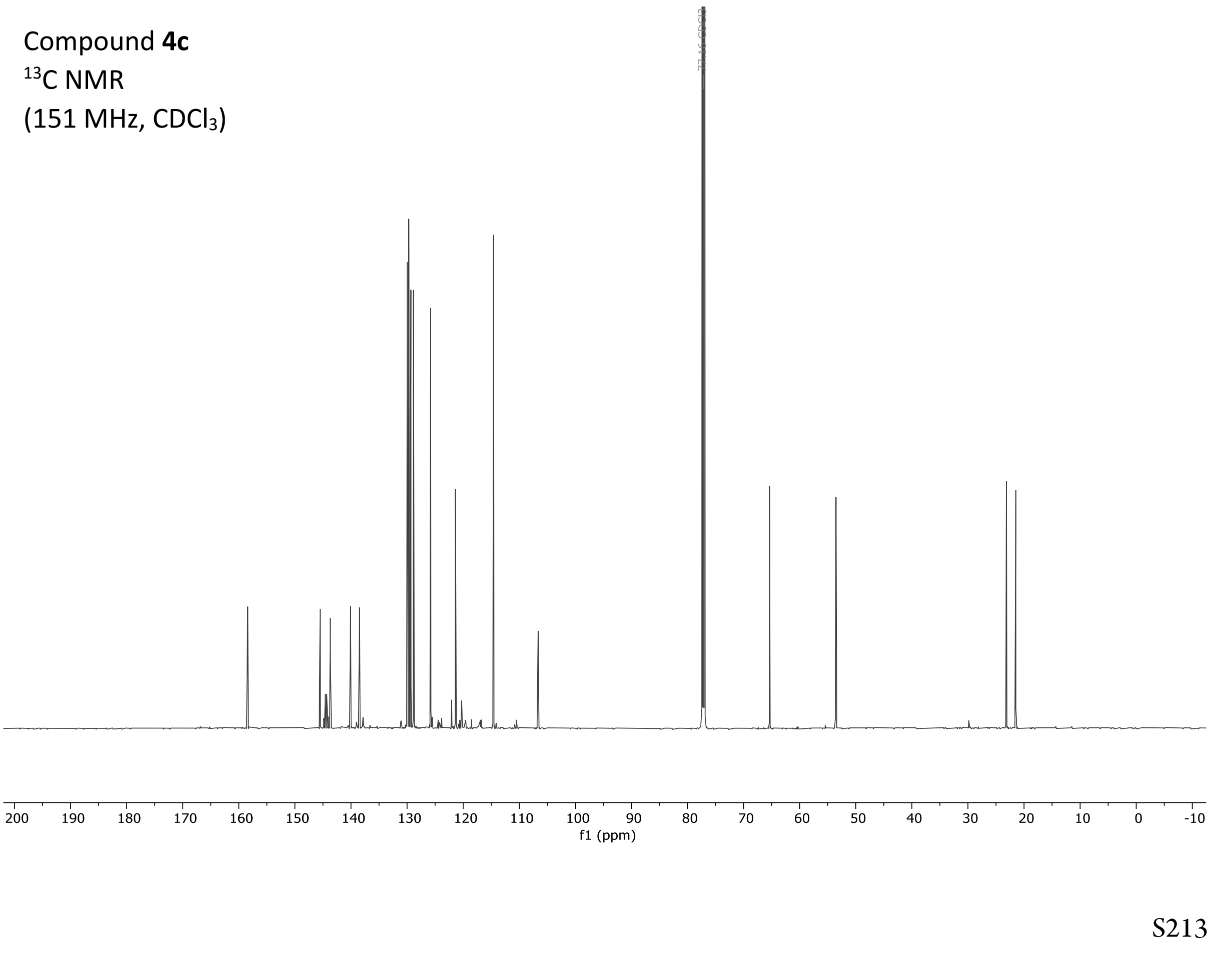

Compound **4c**
^{19}F NMR
(377 MHz, $CDCl_3$)

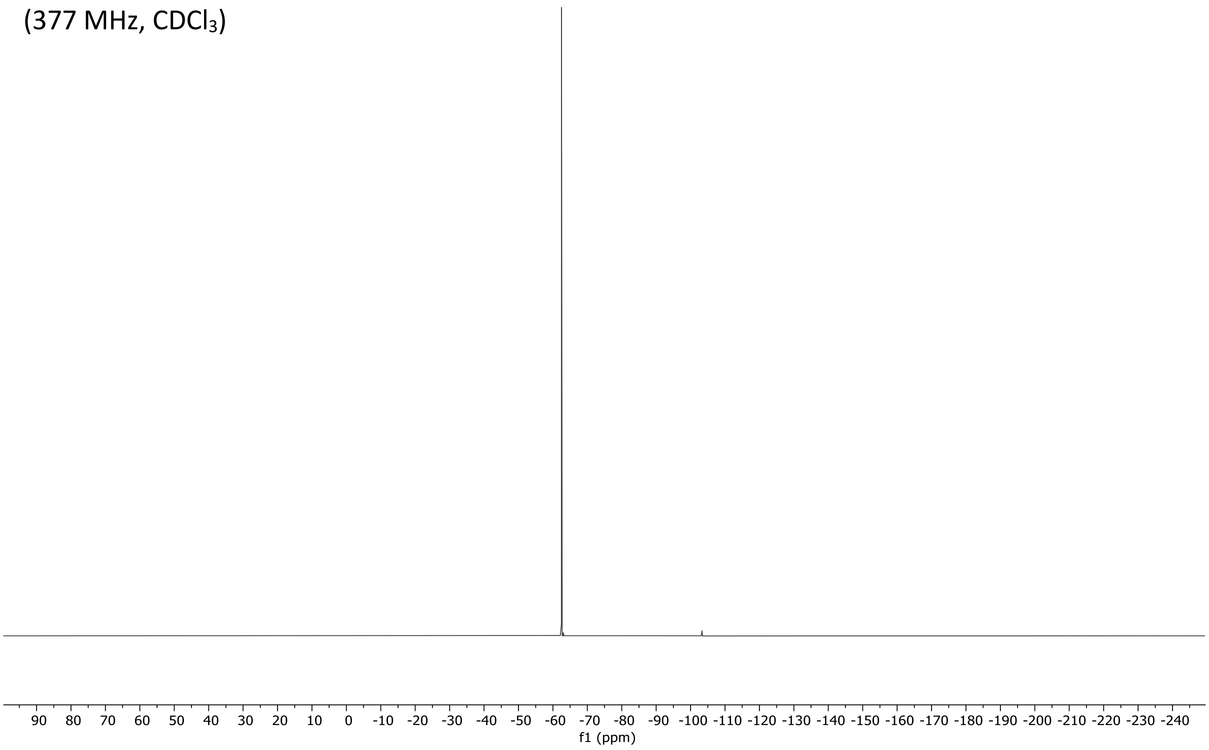

Compound **4d**
^{1}H NMR
(600 MHz, $CDCl_3$)

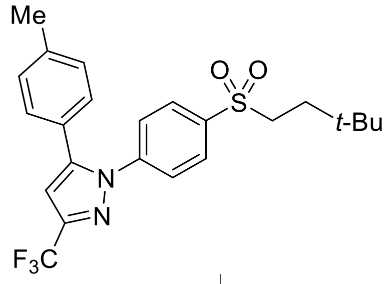

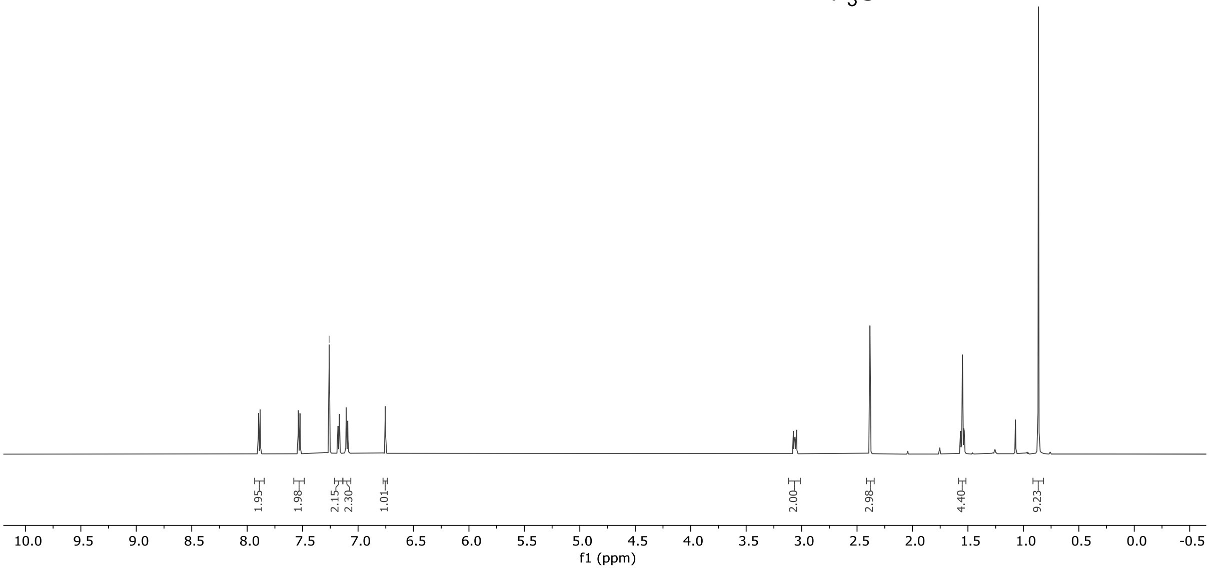

Compound **4d**
^{13}C NMR
(151 MHz, $CDCl_3$)

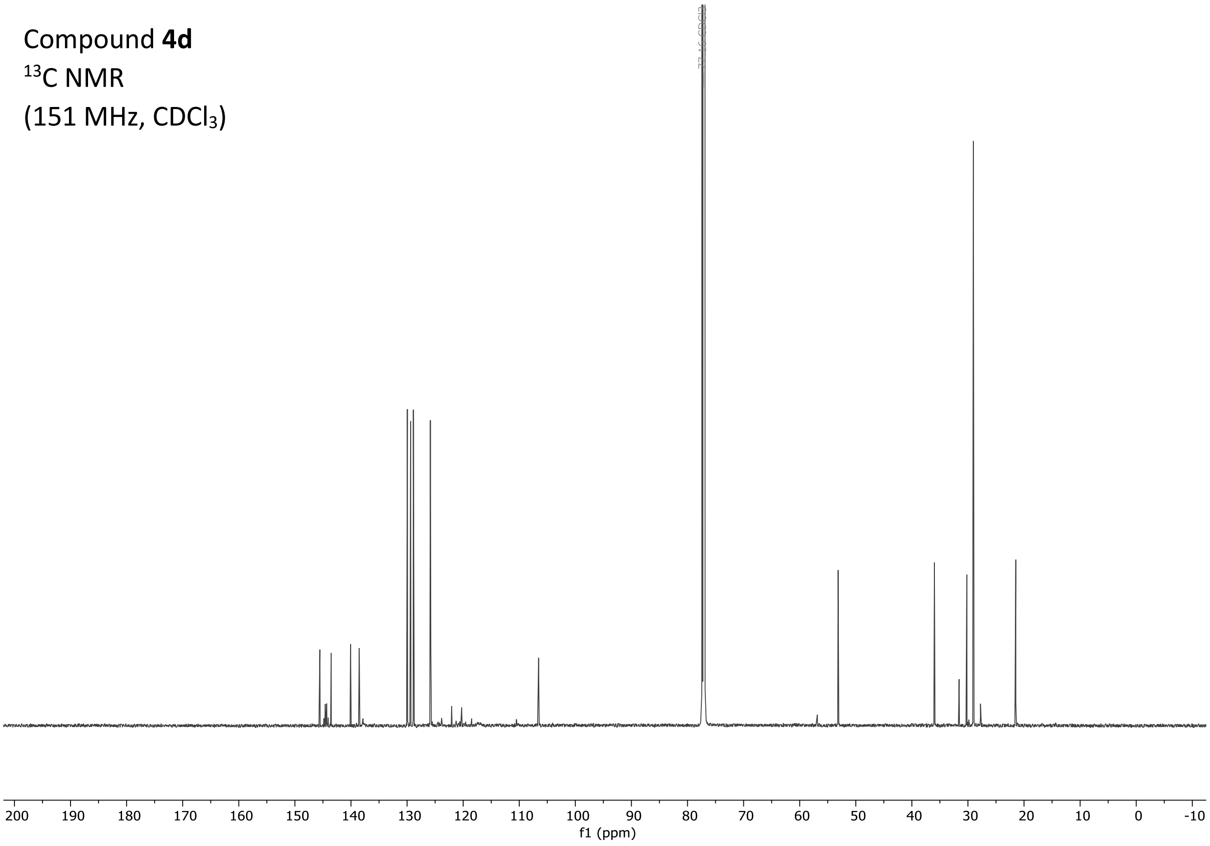

Compound **4d**
^{19}F NMR
(377 MHz, $CDCl_3$)

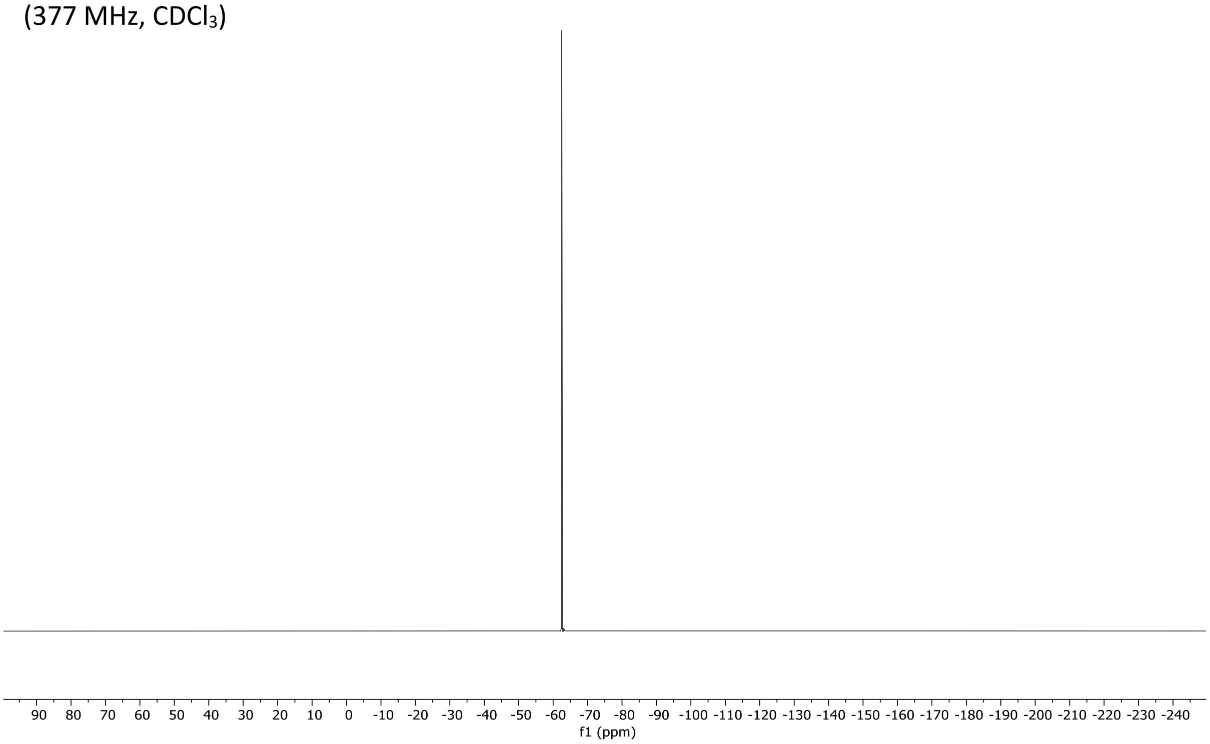

Compound **4e**
^{1}H NMR
(600 MHz, $CDCl_3$)

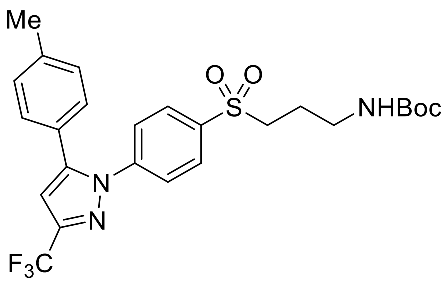

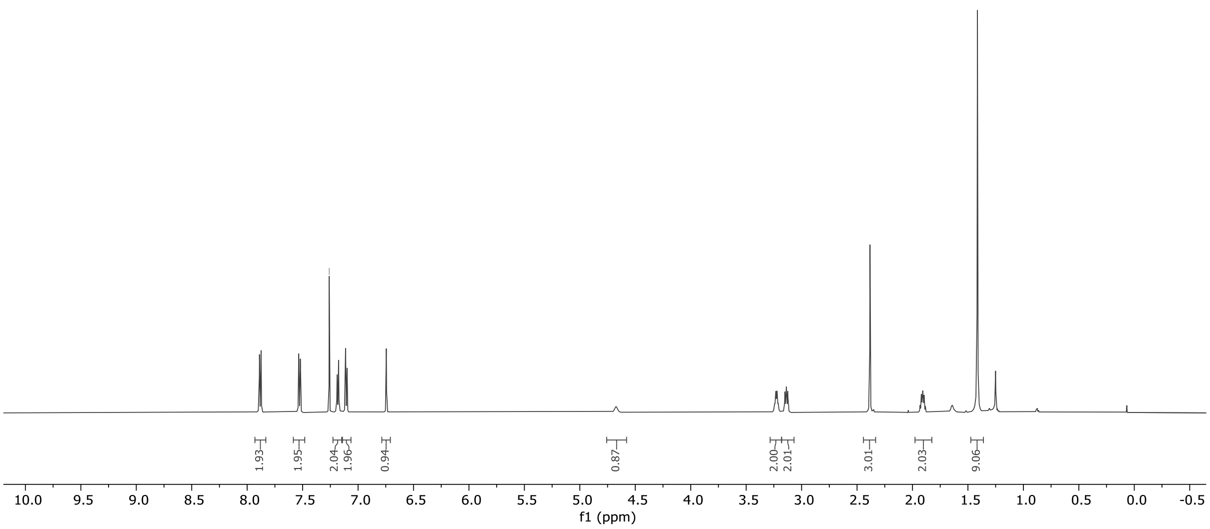

Compound **4e**
^{13}C NMR
(151 MHz, $CDCl_3$)

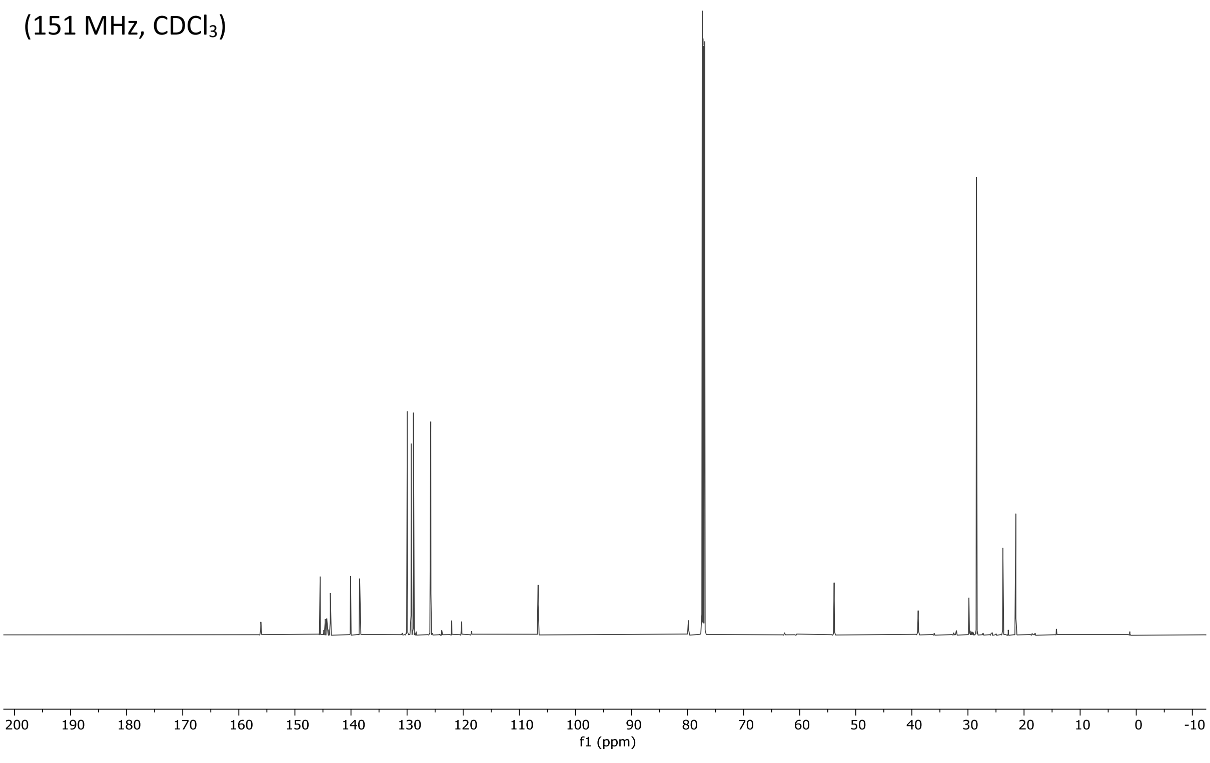

Compound **4e**
^{19}F NMR
(377 MHz, $CDCl_3$)

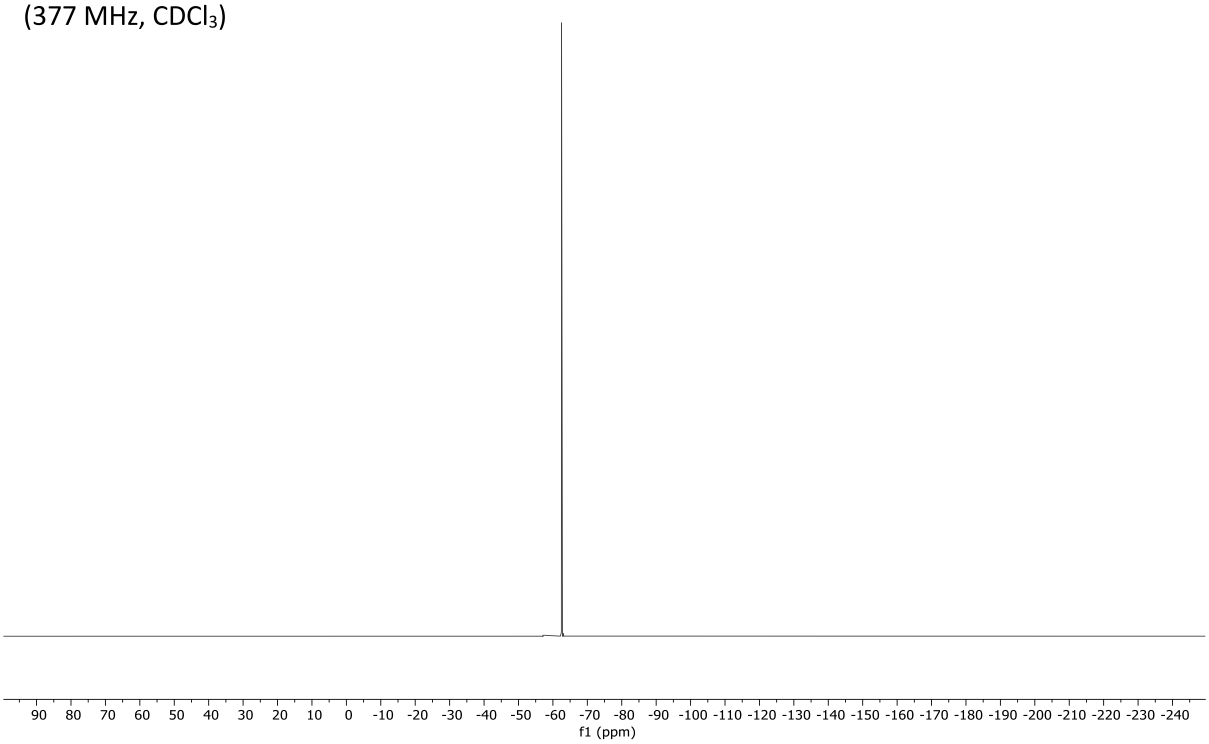

Compound **4f**
^{1}H NMR
(600 MHz, $CDCl_3$)

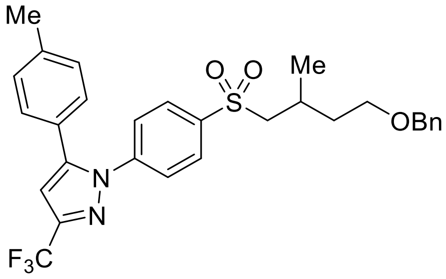

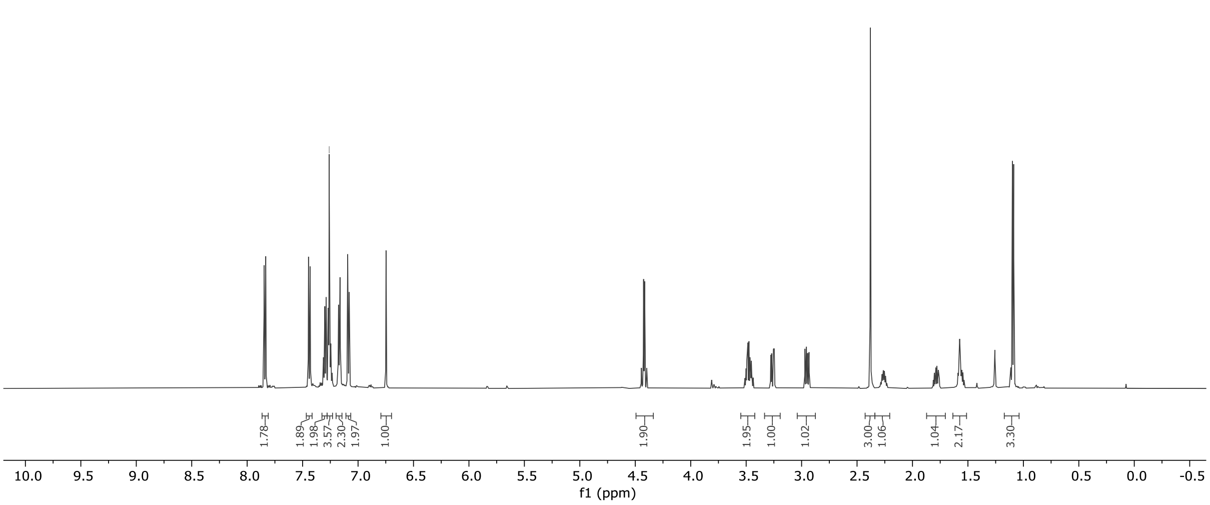

Compound **4f**
^{13}C NMR
(151 MHz, $CDCl_3$)

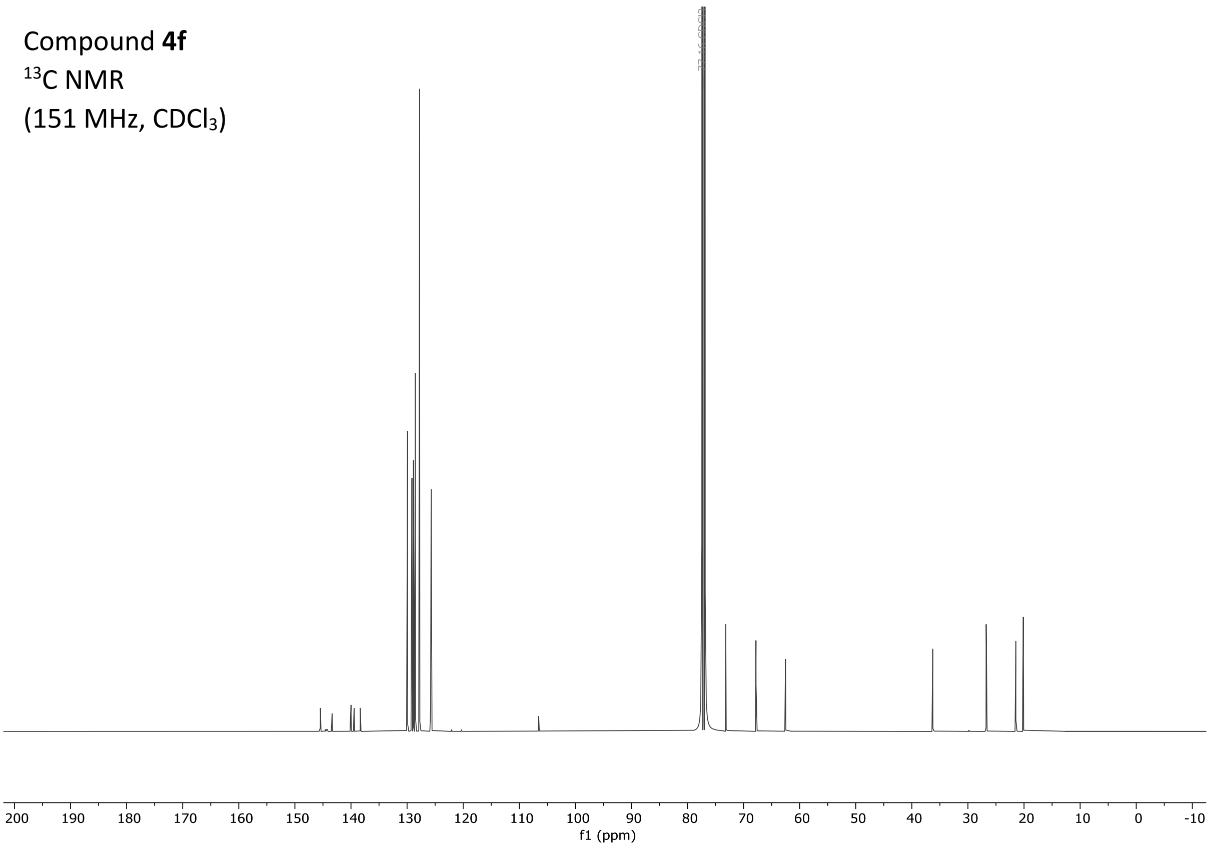

Compound **4f**
^{19}F NMR
(377 MHz, $CDCl_3$)

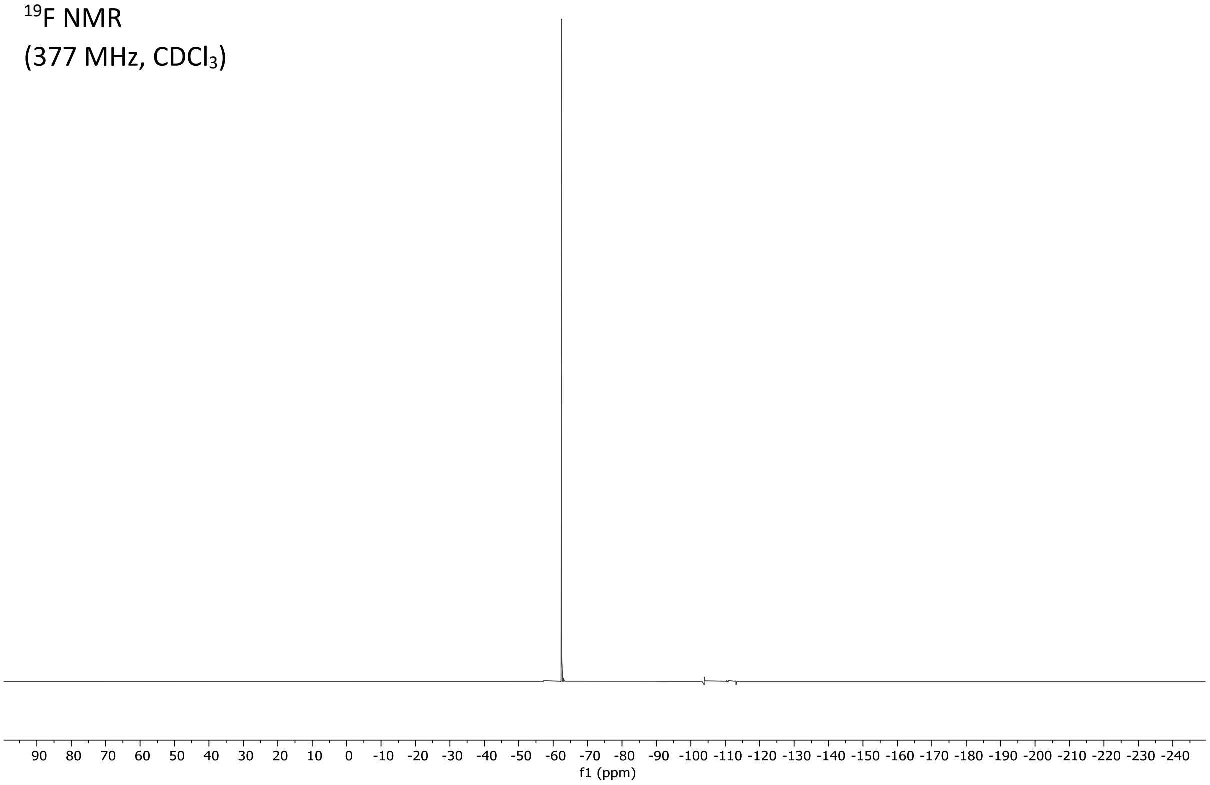

Compound **4g**
^{1}H NMR
(600 MHz, $CDCl_3$)

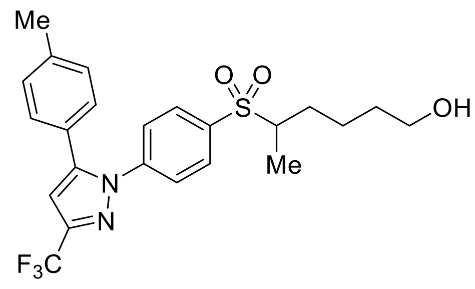

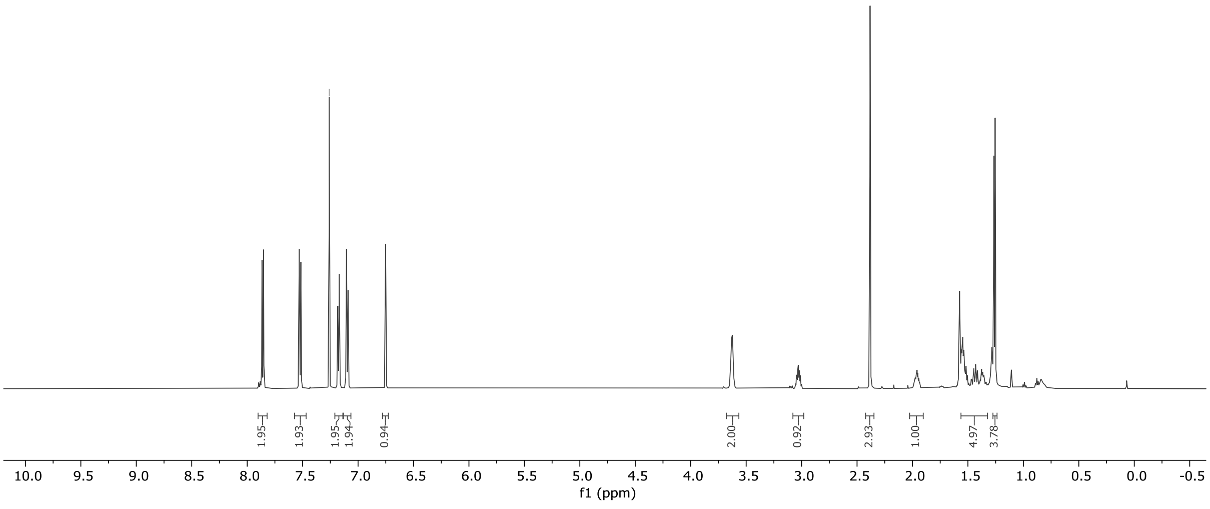

Compound **4g**
^{13}C NMR
(151 MHz, $CDCl_3$)

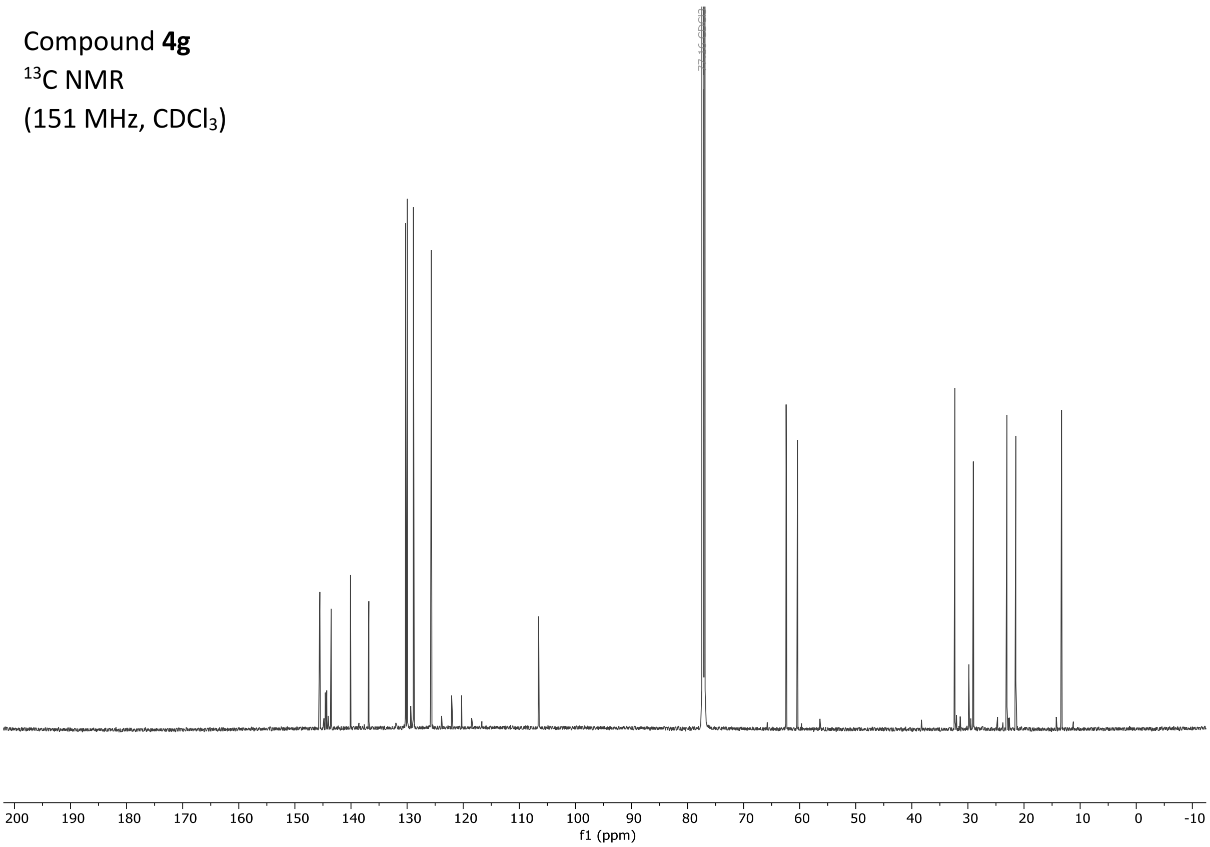

Compound **4g**
^{19}F NMR
(377 MHz, $CDCl_3$)

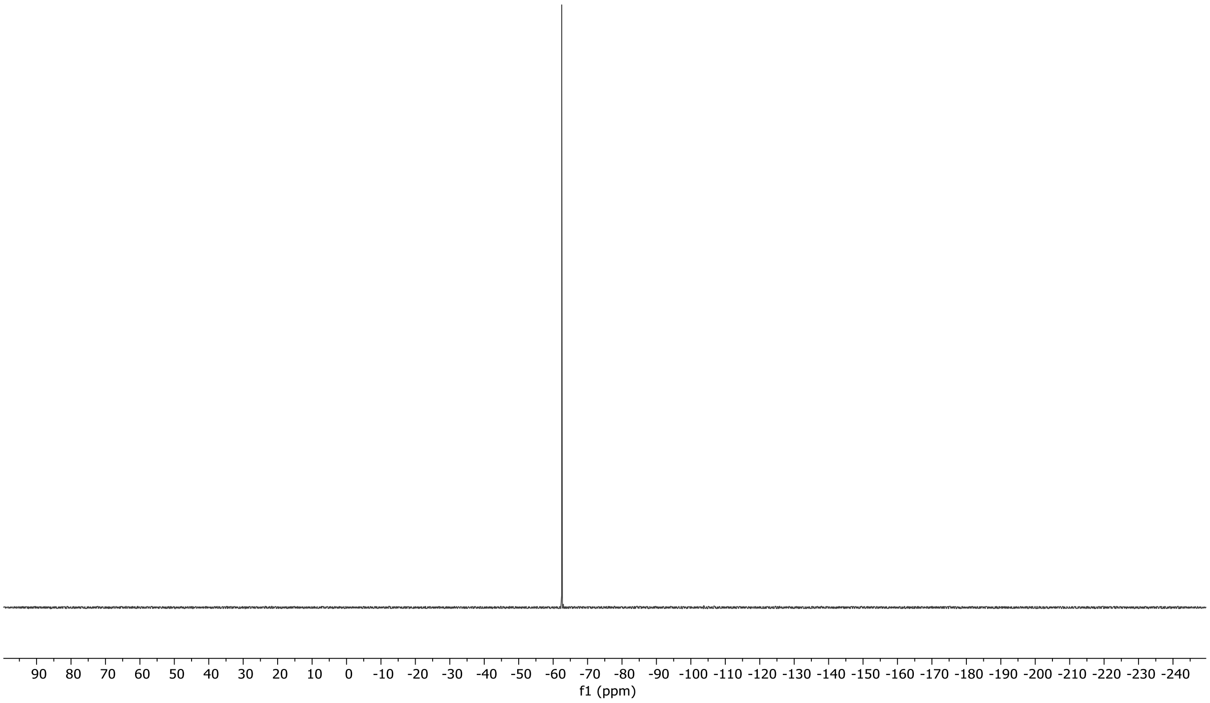

Compound **4g'**
^{1}H NMR
(600 MHz, $CDCl_3$)

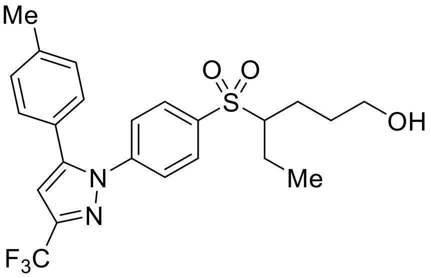

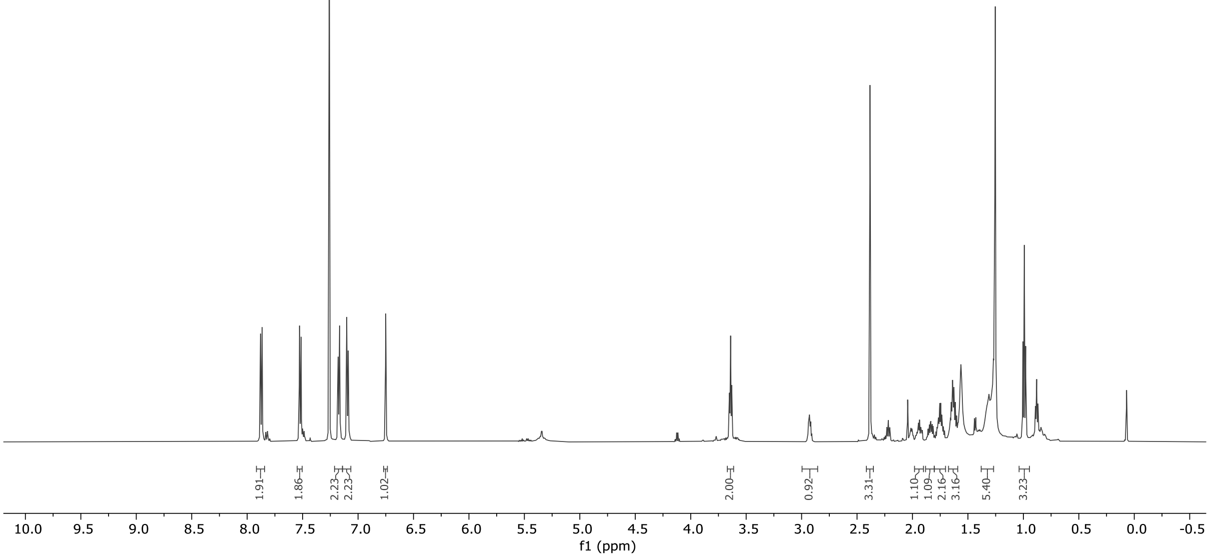

Compound **4g'**
^{13}C NMR
(151 MHz, $CDCl_3$)

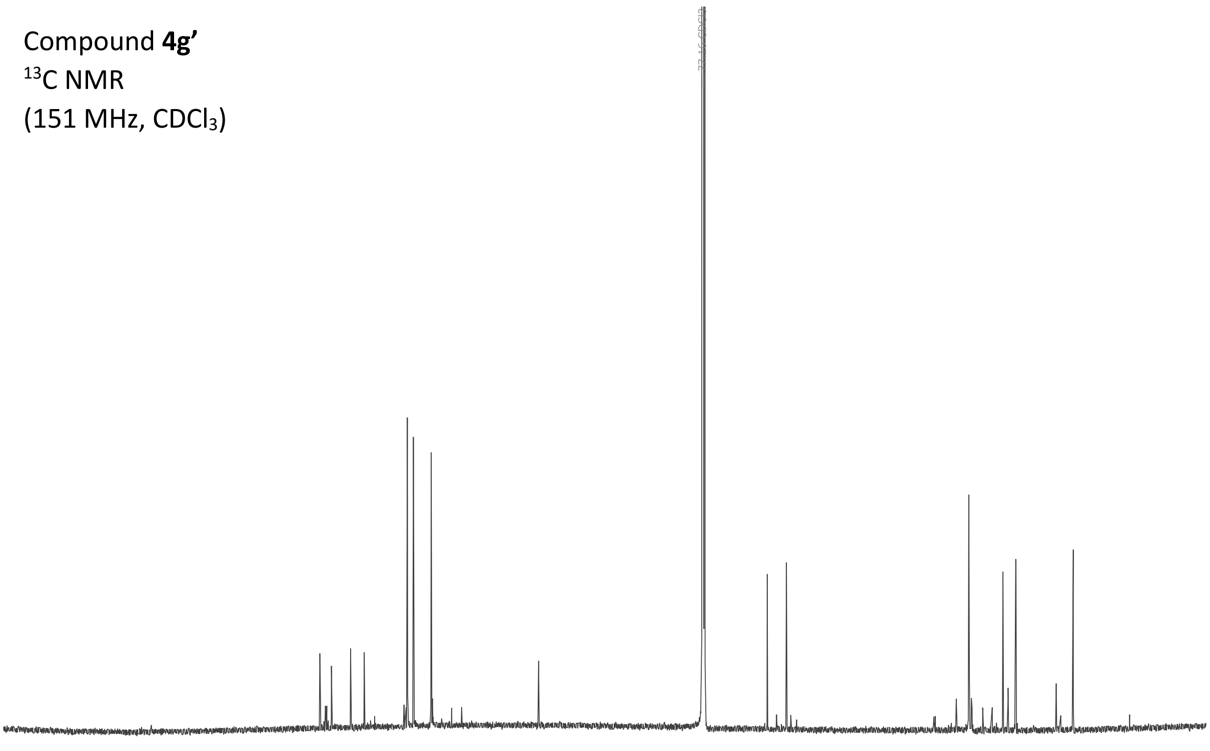

Compound **4g’**

^{19}F NMR

(377 MHz, $CDCl_3$)

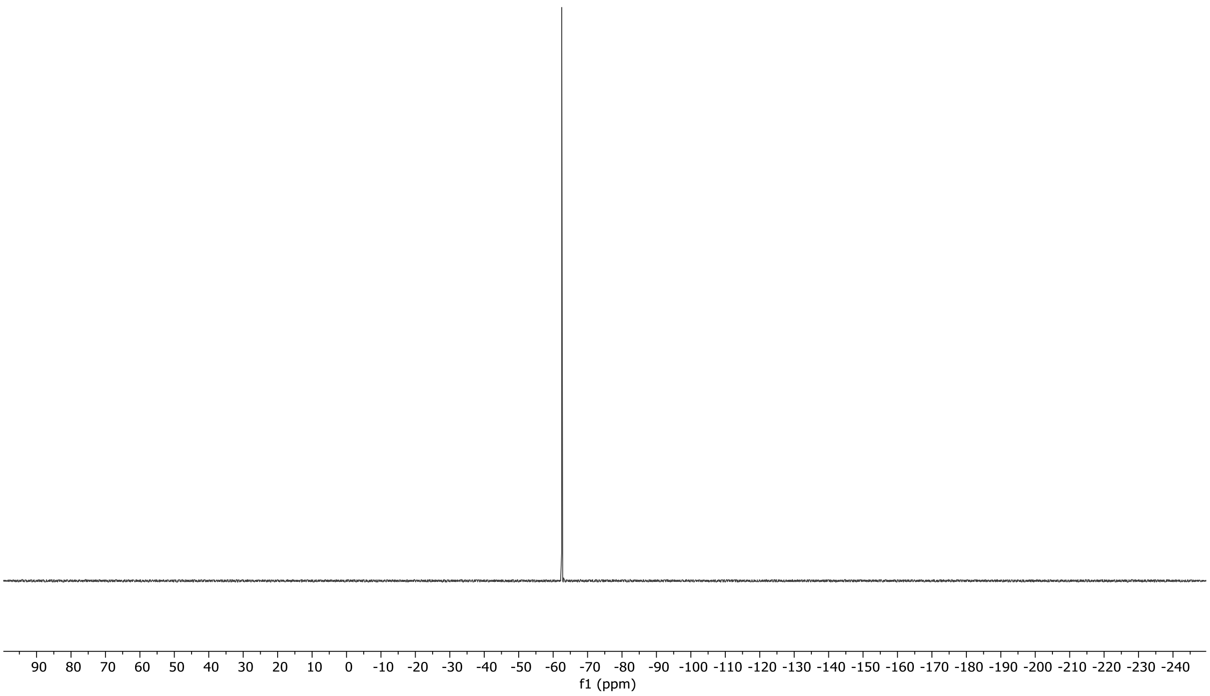

Compound **4h**
^{1}H NMR
(600 MHz, $CDCl_3$)

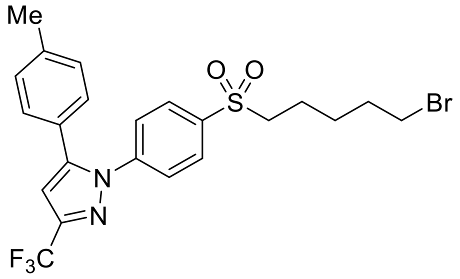

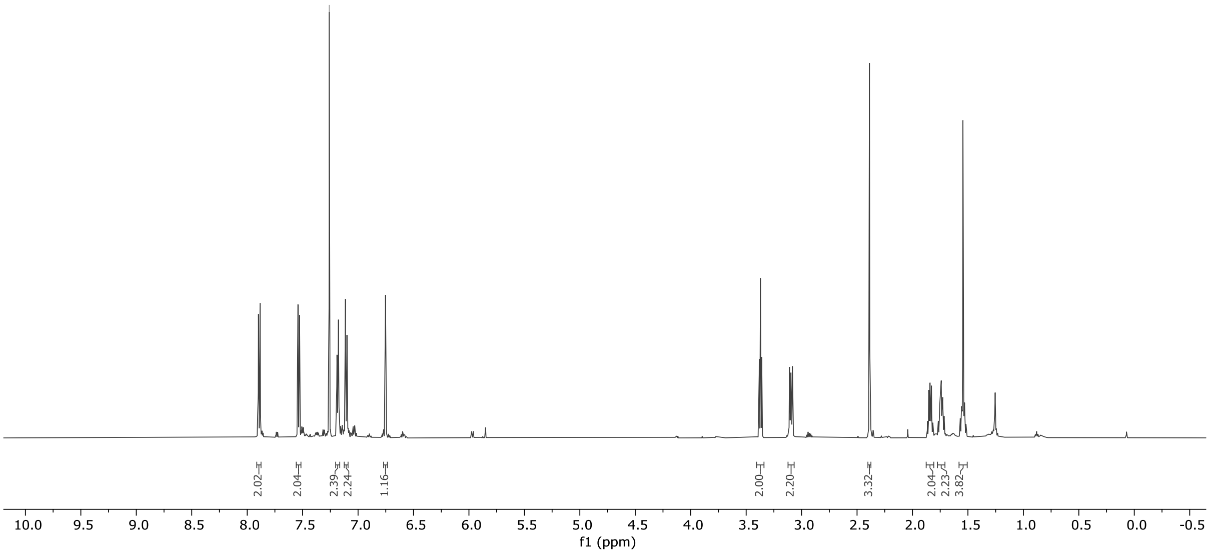

Compound **4h**
^{13}C NMR
(151 MHz, $CDCl_3$)

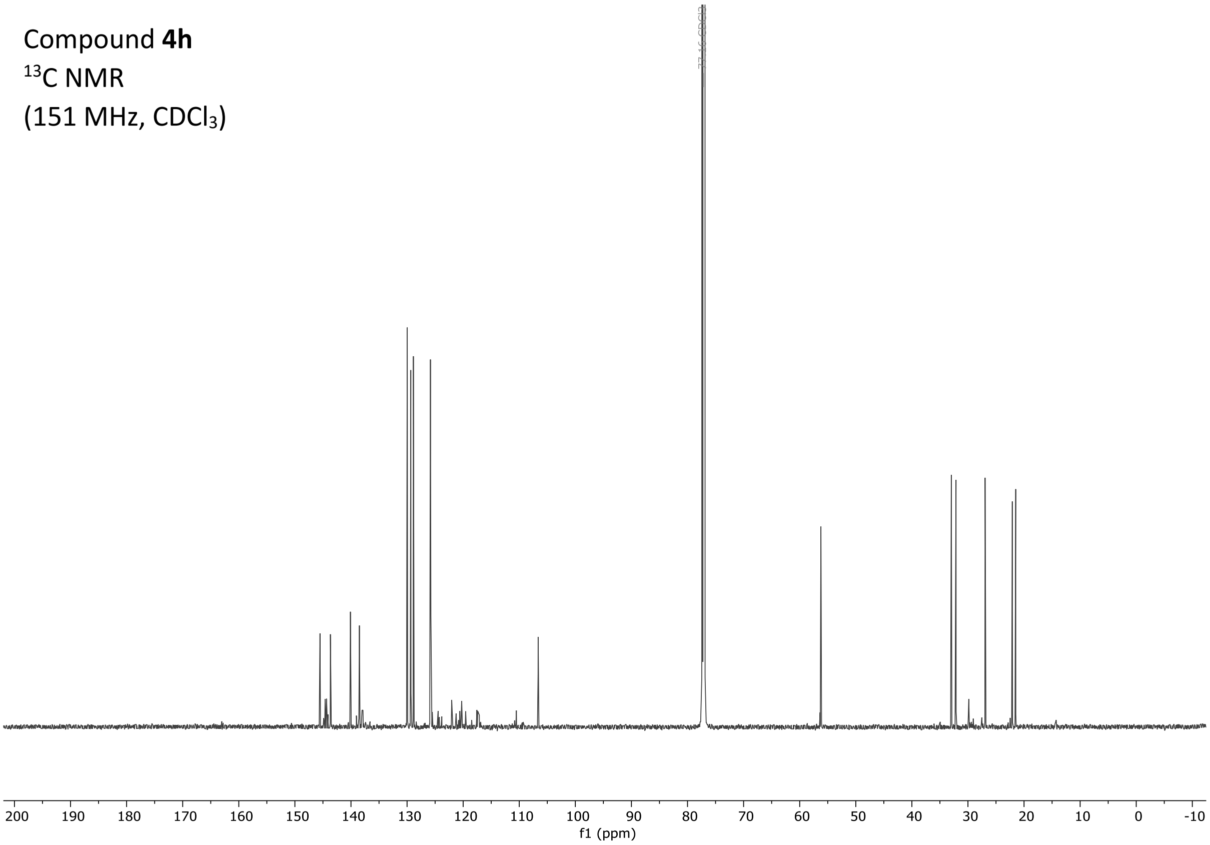

Compound **4h**
^{19}F NMR
(377 MHz, $CDCl_3$)

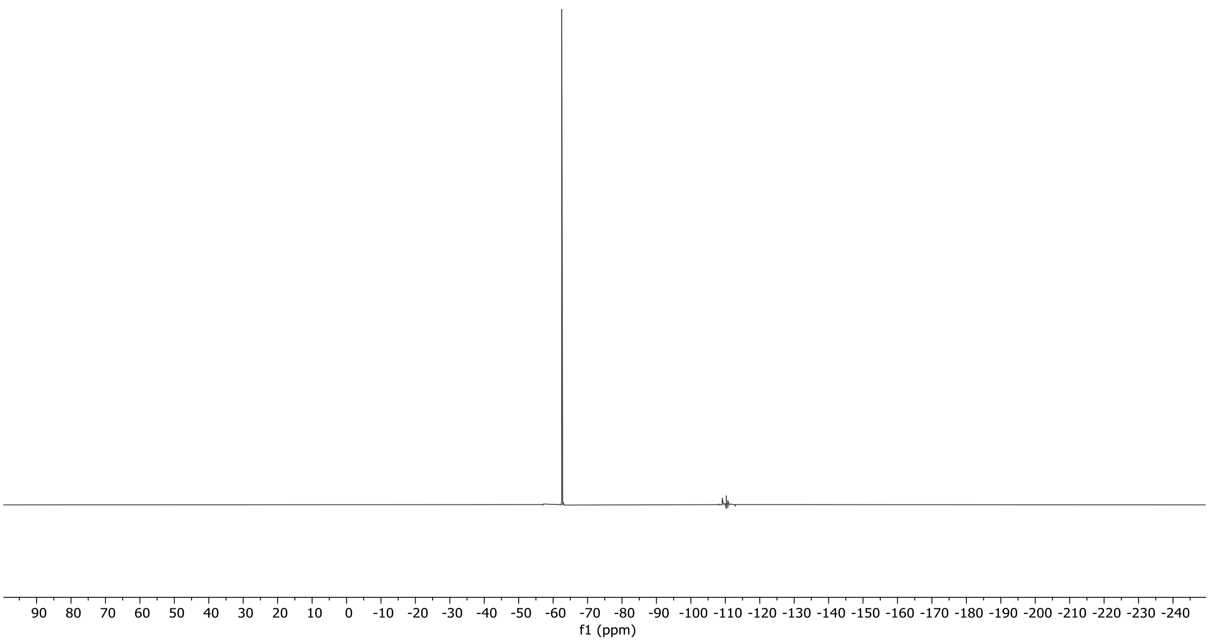

Compound **6**
^{1}H NMR
(400 MHz, $CDCl_3$)

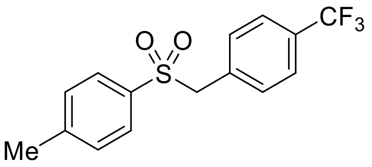

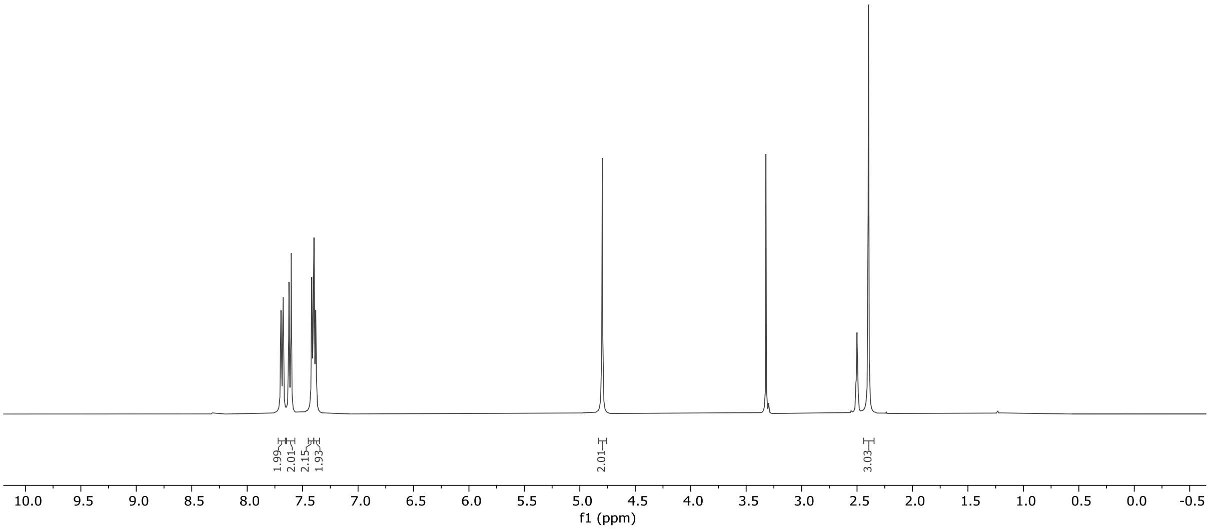

Compound **6**
^{13}C NMR
(101 MHz, $CDCl_3$)

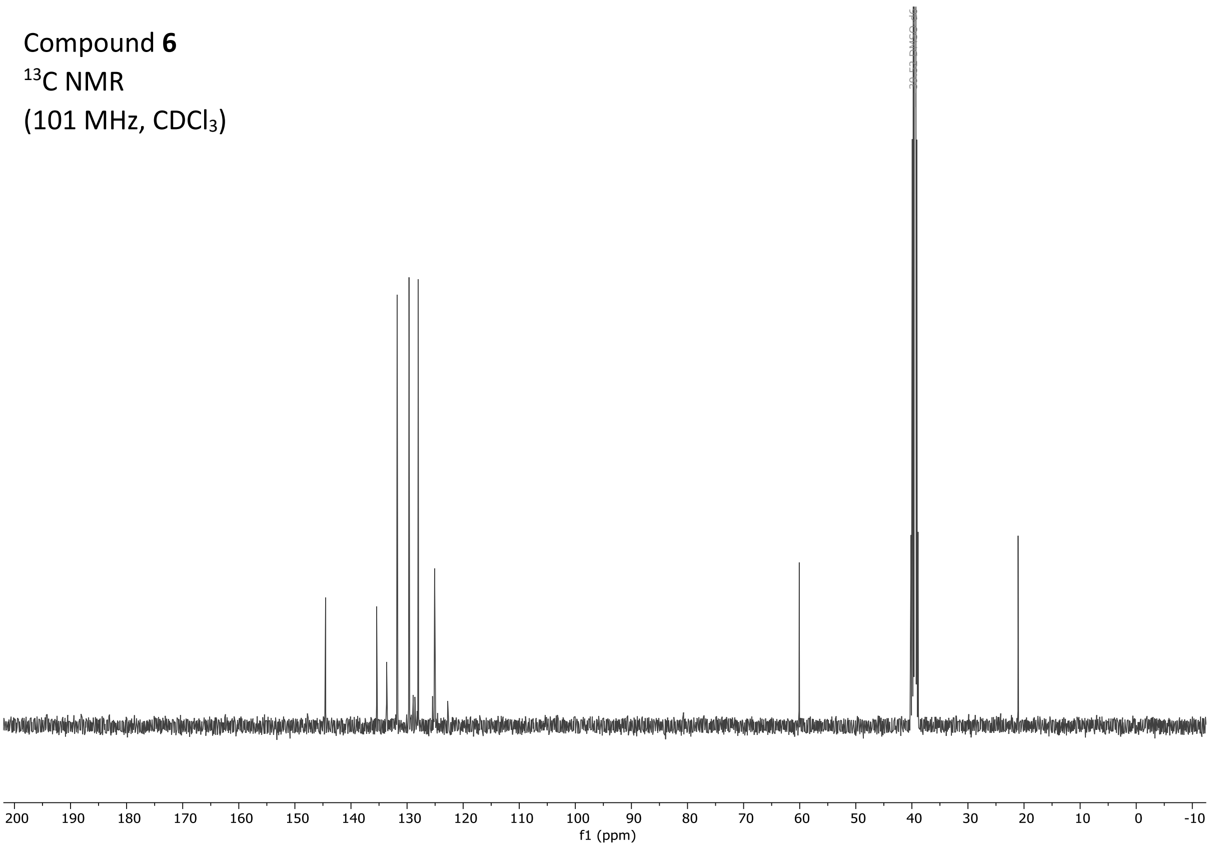

Compound **6**
^{19}F NMR
(377 MHz, $CDCl_3$)

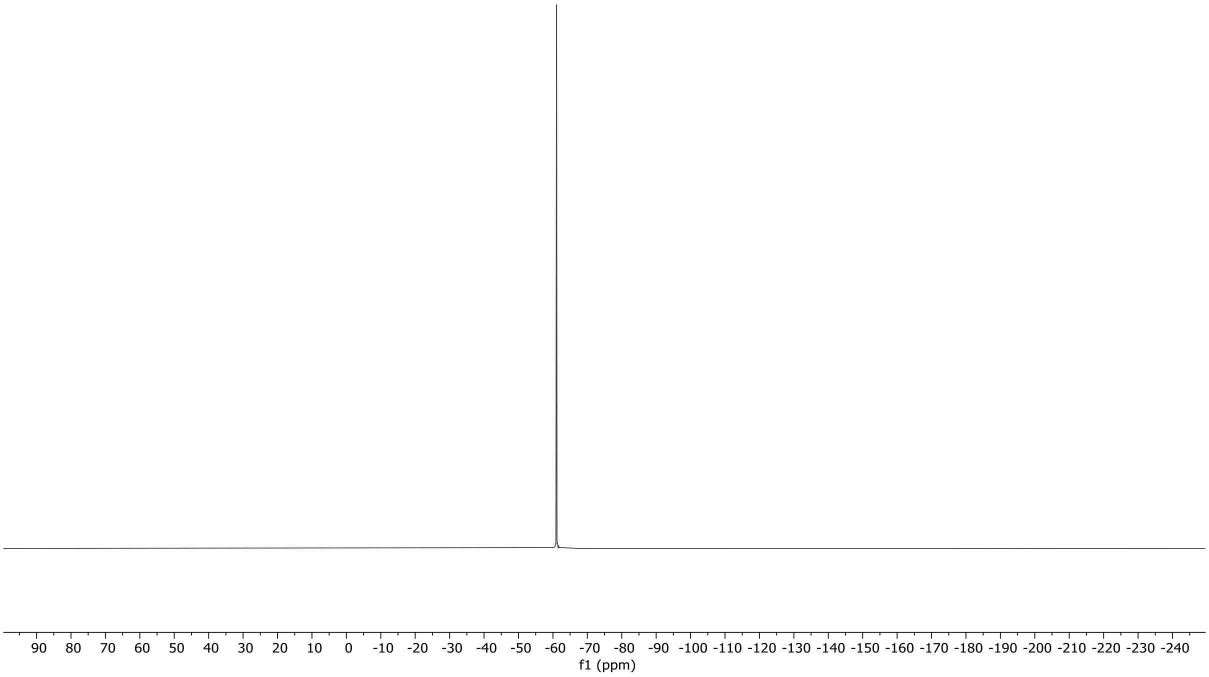

Compound **7**
^{1}H NMR
(400 MHz, $CDCl_3$)

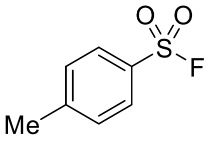

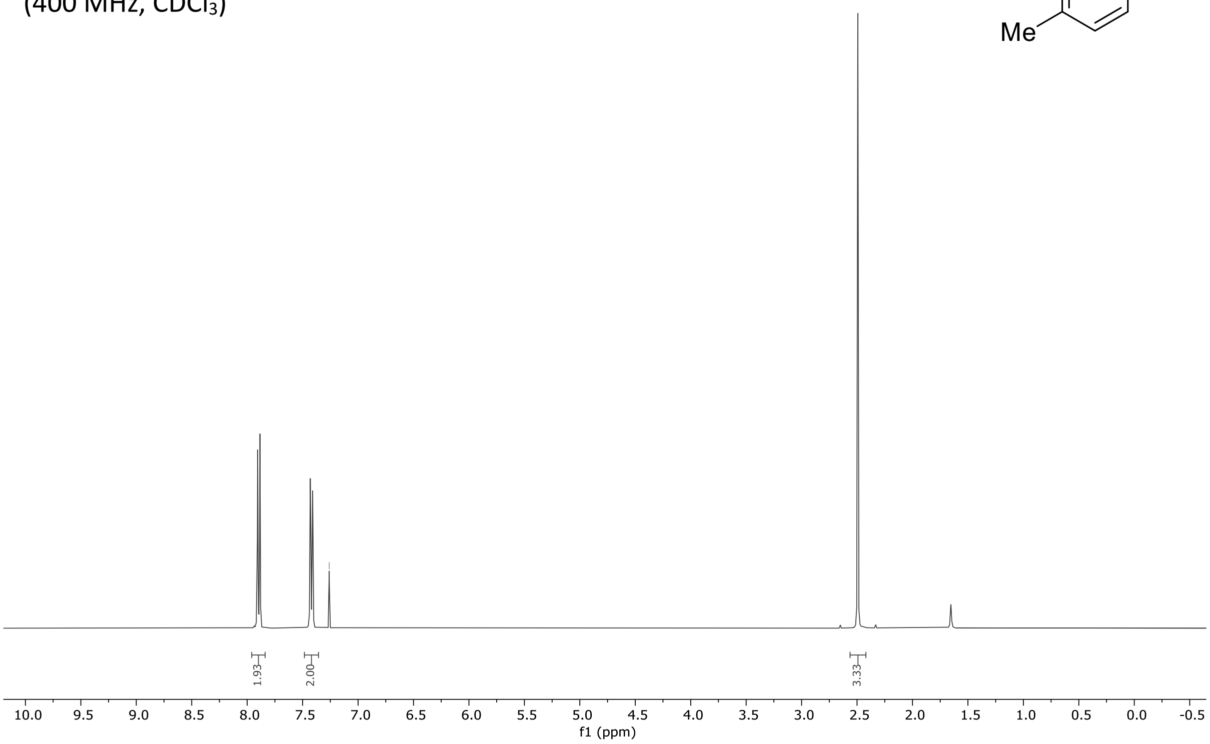

Compound **7**
^{13}C NMR
(101 MHz, $CDCl_3$)

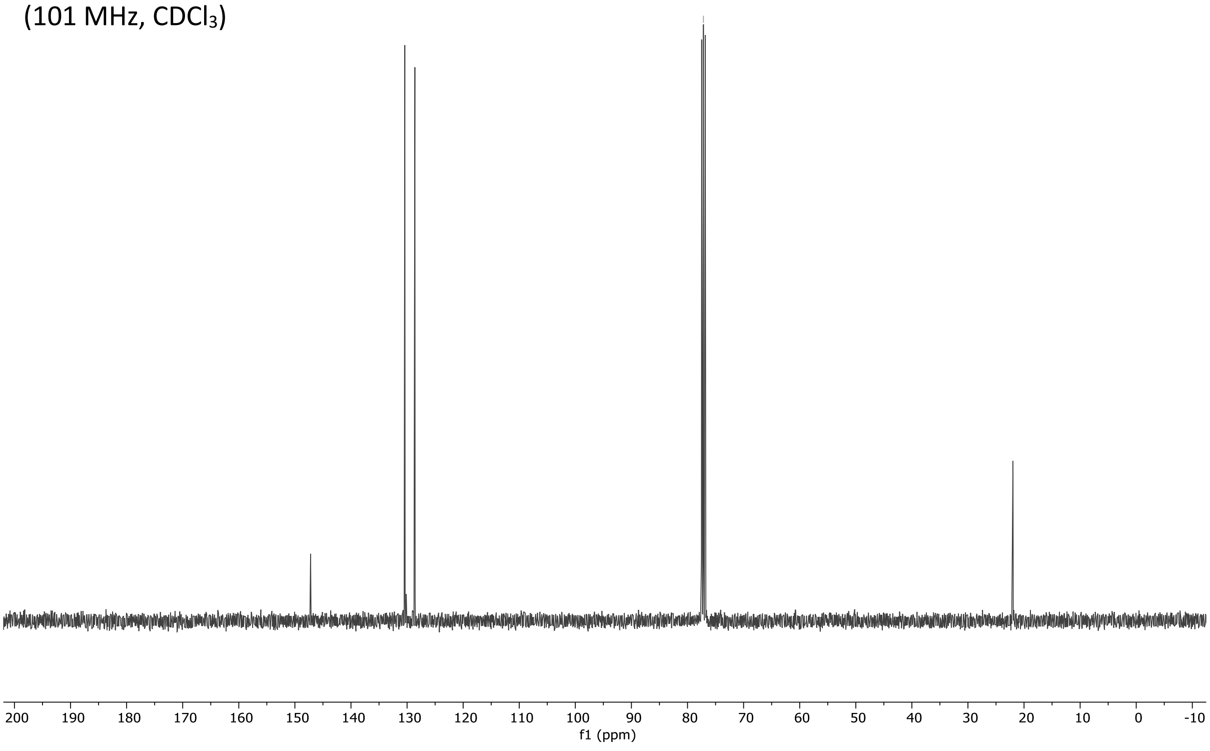

Compound **7**
^{19}F NMR
(377 MHz, $CDCl_3$)

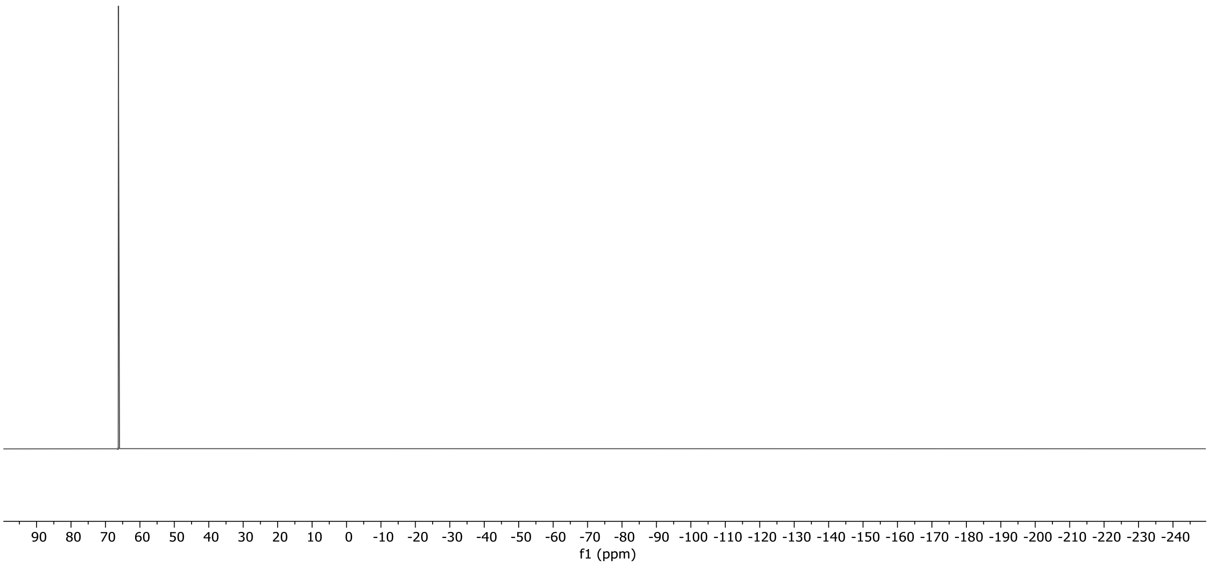

Compound **5-Na**
^{1}H NMR
(400 MHz, MeOD-d_4)

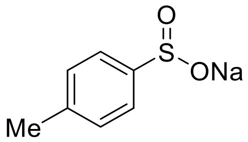

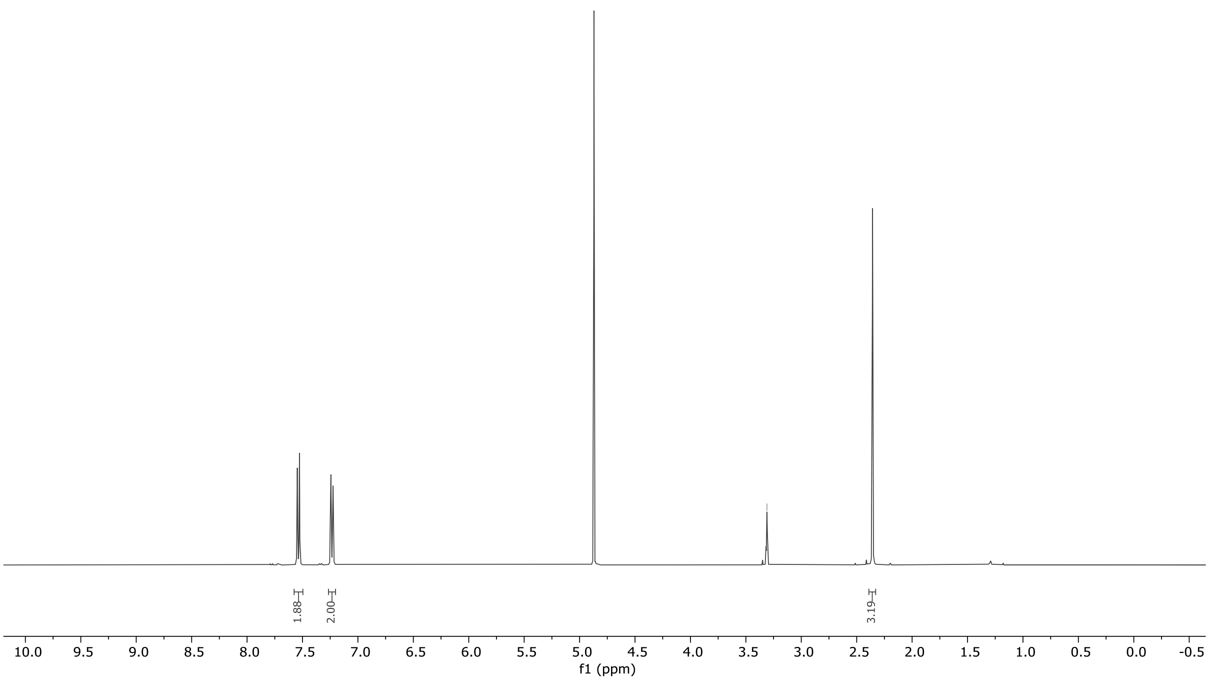

Compound **5-Na**
^{13}C NMR
(101 MHz, DMSO-d_4)

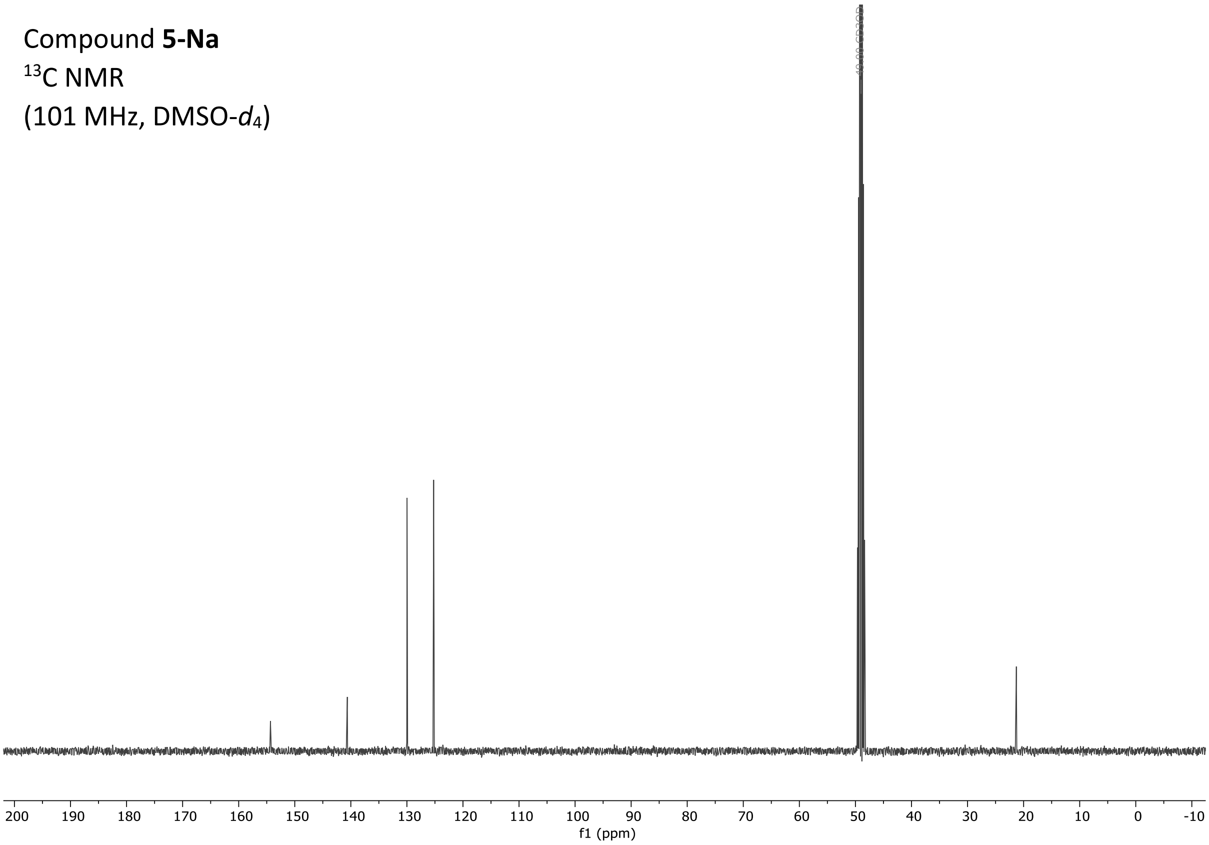

8. Computational Coordinates

1a-S$_0$

C	2.749542	-0.373587	-0.019923
C	3.284700	0.199535	-1.170322
C	3.113085	0.074686	1.242716
C	4.189226	1.243333	-1.043294
H	3.000128	-0.180005	-2.145831
C	4.023441	1.121268	1.350661
H	2.697919	-0.400615	2.124939
C	4.571890	1.720032	0.215763
H	4.612284	1.694295	-1.937218
H	4.315687	1.473168	2.336136
C	5.566787	2.844936	0.333072
H	6.551827	2.537129	-0.035179
H	5.255074	3.712279	-0.258256
H	5.684322	3.168360	1.370730
S	1.586145	-1.706107	-0.178923
O	1.481795	-2.381294	1.117893
O	1.906801	-2.468836	-1.378178
N	0.158954	-0.853739	-0.528378
C	-0.819975	-1.055525	0.272459
H	-0.702826	-1.715288	1.139774
C	-2.122040	-0.429337	0.088290
C	-3.134978	-0.680192	1.013145
C	-2.391623	0.420820	-0.998051
C	-4.394660	-0.105071	0.879863
H	-2.939803	-1.338932	1.855478
C	-3.633934	0.998066	-1.142339
H	-1.606013	0.612359	-1.721271
C	-4.644960	0.738599	-0.203380
H	-5.161067	-0.319564	1.614102
H	-3.860271	1.655689	-1.974891
O	-5.828110	1.351173	-0.434865
C	-6.900067	1.132225	0.465264
H	-6.646717	1.479406	1.473405
H	-7.735526	1.715734	0.079472
H	-7.178507	0.072799	0.497677

1a-T$_1$

C	-2.776247	-0.368805	0.060183
C	-3.253109	0.313746	1.175882
C	-3.273755	-0.105538	-1.209063
C	-4.235846	1.277894	1.006291
H	-2.862684	0.078924	2.160315
C	-4.260625	0.863388	-1.360094
H	-2.898466	-0.662023	-2.061084
C	-4.753998	1.567729	-0.261127
H	-4.613850	1.812917	1.873746
H	-4.656133	1.070812	-2.350605
C	-5.834594	2.604780	-0.423023
H	-6.775361	2.263281	0.023564
H	-5.561635	3.542834	0.071374
H	-6.025941	2.821533	-1.477413
S	-1.513870	-1.600391	0.266655
O	-1.503847	-2.448183	-0.928615
O	-1.672141	-2.213561	1.584940
N	-0.140534	-0.644113	0.335940
C	0.977159	-1.194061	-0.223719
H	0.919952	-2.141030	-0.757135
C	2.173388	-0.517027	-0.125001
C	3.402394	-1.060489	-0.710166
C	2.283721	0.769414	0.565734
C	4.588809	-0.404951	-0.603426
H	3.345317	-2.009951	-1.232586
C	3.477116	1.403175	0.658464
H	1.384086	1.188017	0.998316
C	4.658040	0.840869	0.084368
H	5.479326	-0.836780	-1.045531
H	3.572100	2.355987	1.169778
O	5.781346	1.564931	0.243777
C	7.006765	1.086007	-0.291221
H	6.946551	0.988616	-1.380217
H	7.754426	1.834729	-0.032619
H	7.279792	0.124891	0.156732

1a-T_1'

C	2.738635	-0.195212	0.279478
C	3.720067	-1.004887	-0.282193
C	2.961913	1.157471	0.508520
C	4.941776	-0.440384	-0.623205
H	3.529224	-2.061107	-0.438509
C	4.191080	1.703639	0.161303
H	2.187588	1.766750	0.961910
C	5.196354	0.917154	-0.407046
H	5.713712	-1.066645	-1.061999
H	4.373320	2.759932	0.338583
C	6.537332	1.510280	-0.750036
H	7.241709	1.380772	0.080117
H	6.974459	1.027568	-1.628833
H	6.460559	2.582186	-0.952172
S	1.168032	-0.899266	0.697560
O	0.518466	-0.051838	1.692496
O	1.334332	-2.333153	0.911247
N	0.290955	-0.705868	-0.775635
C	-0.966200	-1.290018	-0.797125
H	-0.972206	-2.321273	-1.147249
C	-2.178009	-0.602209	-0.544723
C	-3.410580	-1.269355	-0.723786
C	-2.223326	0.752531	-0.122276
C	-4.621931	-0.633551	-0.498546
H	-3.408863	-2.307619	-1.043888
C	-3.425689	1.384967	0.098081
H	-1.296641	1.290503	0.043416
C	-4.637462	0.702042	-0.086069
H	-5.543131	-1.184480	-0.645790
H	-3.461832	2.419458	0.423732
O	-5.765365	1.419301	0.161164
C	-7.018474	0.783546	0.005416
H	-7.117330	-0.071891	0.684266
H	-7.768098	1.533775	0.257345
H	-7.170045	0.450842	-1.028463

1a-S_0(PhMe)

C	2.748152	-0.374226	-0.019395
C	3.278253	0.200869	-1.171969
C	3.109880	0.078810	1.242761
C	4.178067	1.249008	-1.047325
H	2.994805	-0.176341	-2.148605
C	4.015594	1.129641	1.347720
H	2.697158	-0.392024	2.128262
C	4.560462	1.729234	0.210963
H	4.596837	1.700731	-1.942587
H	4.306089	1.484605	2.332376
C	5.548062	2.860197	0.325119
H	6.525866	2.566785	-0.072312
H	5.214600	3.733539	-0.244998
H	5.686391	3.166605	1.365116
S	1.587712	-1.707386	-0.173438
O	1.489854	-2.390184	1.122339
O	1.916057	-2.484141	-1.366516
N	0.160256	-0.874798	-0.534037
C	-0.815966	-1.046867	0.281244
H	-0.695308	-1.679790	1.166876
C	-2.115255	-0.424440	0.090082
C	-3.121180	-0.653745	1.030373
C	-2.394425	0.399916	-1.014611
C	-4.381739	-0.083563	0.893538
H	-2.919095	-1.291448	1.886903
C	-3.638310	0.972250	-1.162277
H	-1.617573	0.577513	-1.750637
C	-4.642048	0.733798	-0.208525
H	-5.141904	-0.280915	1.638706
H	-3.870156	1.609412	-2.009138
O	-5.826693	1.337681	-0.444032
C	-6.895832	1.134181	0.469415
H	-6.636695	1.505968	1.466384
H	-7.735100	1.705150	0.074359
H	-7.167151	0.074731	0.525542

1a-T$_1$(PhMe)

C	2.732020	-0.369744	-0.043944
C	3.223227	0.275763	-1.176966
C	3.126801	0.019094	1.230143
C	4.116972	1.324711	-1.020782
H	2.913793	-0.049798	-2.164263
C	4.025628	1.072237	1.367388
H	2.743456	-0.504065	2.099310
C	4.532032	1.739300	0.250706
H	4.505458	1.829444	-1.901399
H	4.341066	1.375858	2.361619
C	5.514873	2.870616	0.399104
H	6.493998	2.593400	-0.007163
H	5.178374	3.760125	-0.143470
H	5.653033	3.144853	1.448176
S	1.574886	-1.703091	-0.234559
O	1.544111	-2.463144	1.022455
O	1.885579	-2.412013	-1.478170
N	0.151803	-0.872118	-0.497583
C	-0.875395	-1.161313	0.335145
H	-0.749984	-1.884942	1.138557
C	-2.091631	-0.516271	0.159957
C	-3.228469	-0.799730	1.039859
C	-2.301529	0.466133	-0.903454
C	-4.425980	-0.183025	0.869043
H	-3.093348	-1.523423	1.836945
C	-3.503715	1.069954	-1.055160
H	-1.470854	0.687263	-1.561152
C	-4.597838	0.768222	-0.182986
H	-5.248493	-0.416302	1.534980
H	-3.673567	1.798384	-1.841847
O	-5.736730	1.430006	-0.434227
C	-6.887215	1.197667	0.375326
H	-6.688861	1.469052	1.416309
H	-7.665530	1.841812	-0.030195
H	-7.201699	0.152146	0.308887

1a-T$_1$’(PhMe)

C	2.711994	-0.234041	0.270617
C	3.704275	-0.920069	-0.425485
C	2.884228	1.088339	0.661028
C	4.883699	-0.259129	-0.734536
H	3.556083	-1.955848	-0.710982
C	4.074720	1.732402	0.342827
H	2.104584	1.600967	1.213667
C	5.087613	1.073348	-0.356677
H	5.663705	-0.787473	-1.275788
H	4.218005	2.764838	0.647927
C	6.382218	1.767245	-0.686207
H	7.208094	1.346791	-0.101590
H	6.637228	1.645430	-1.743646
H	6.328944	2.837025	-0.469208
S	1.193996	-1.057716	0.650323
O	0.512248	-0.338173	1.723107
O	1.436375	-2.495829	0.754840
N	0.288417	-0.834494	-0.802262
C	-0.986111	-1.381187	-0.792853
H	-1.032527	-2.420580	-1.115254
C	-2.167173	-0.640842	-0.543843
C	-3.427067	-1.270584	-0.662805
C	-2.153927	0.732946	-0.185064
C	-4.608766	-0.581856	-0.436674
H	-3.470197	-2.321295	-0.935236
C	-3.327304	1.418232	0.036510
H	-1.205798	1.248881	-0.078543
C	-4.566902	0.771053	-0.084229
H	-5.552233	-1.104908	-0.536079
H	-3.316498	2.468064	0.311383
O	-5.662167	1.537305	0.158395
C	-6.943486	0.938412	0.061799
H	-7.051805	0.119059	0.781241
H	-7.659503	1.725296	0.297480
H	-7.130636	0.566617	-0.951884

1a-S_0(MeCN)

C	2.742583	-0.373230	-0.007157
C	3.329239	0.126196	-1.167797
C	3.026791	0.175738	1.238375
C	4.211147	1.193202	-1.069369
H	3.103018	-0.317670	-2.131179
C	3.913945	1.243559	1.316384
H	2.566140	-0.227871	2.133347
C	4.518380	1.766517	0.170251
H	4.672204	1.586838	-1.970793
H	4.141100	1.674877	2.286836
C	5.495681	2.907587	0.261216
H	6.522039	2.548205	0.125127
H	5.306804	3.653116	-0.516952
H	5.441554	3.402803	1.233917
S	1.596995	-1.720207	-0.125789
O	1.505989	-2.377383	1.185988
O	1.948890	-2.539975	-1.288992
N	0.161774	-0.938191	-0.528541
C	-0.801731	-1.031290	0.319666
H	-0.665206	-1.581638	1.255787
C	-2.100432	-0.426337	0.099172
C	-3.086156	-0.567901	1.080067
C	-2.403730	0.297663	-1.068979
C	-4.348015	-0.008876	0.921400
H	-2.865377	-1.125234	1.986095
C	-3.650073	0.857435	-1.239041
H	-1.645896	0.411110	-1.836794
C	-4.632436	0.707835	-0.244088
H	-5.090709	-0.135798	1.698574
H	-3.898152	1.417948	-2.134205
O	-5.819986	1.291240	-0.504352
C	-6.868218	1.181396	0.455168
H	-6.577606	1.642498	1.404016
H	-7.714156	1.719473	0.030271
H	-7.141085	0.133957	0.615603

1a-T_1(MeCN)

C	-2.698740	-0.366910	0.015597
C	-3.261888	0.151622	1.180817
C	-2.941489	0.221921	-1.220823
C	-4.076950	1.271054	1.096017
H	-3.069723	-0.321748	2.137548
C	-3.761570	1.343706	-1.286137
H	-2.503104	-0.195552	-2.120692
C	-4.339895	1.884383	-0.135349
H	-4.521191	1.675668	2.001249
H	-3.956856	1.802978	-2.250786
C	-5.236416	3.091458	-0.208213
H	-6.264105	2.830184	0.067250
H	-4.903963	3.870899	0.484891
H	-5.254795	3.514649	-1.215581
S	-1.624947	-1.777824	0.116746
O	-1.613370	-2.436690	-1.200819
O	-2.033515	-2.581857	1.276985
N	-0.161668	-1.105747	0.500063
C	0.775495	-1.080719	-0.473689
H	0.578301	-1.520534	-1.449146
C	2.016375	-0.491806	-0.214147
C	3.051839	-0.456693	-1.241652
C	2.334577	0.100568	1.078594
C	4.267099	0.099598	-1.003019
H	2.831273	-0.891047	-2.210985
C	3.552239	0.653985	1.301176
H	1.577142	0.085087	1.851521
C	4.552123	0.670961	0.277827
H	5.017037	0.108565	-1.784886
H	3.802700	1.097073	2.259851
O	5.712258	1.238713	0.613631
C	6.783775	1.303514	-0.334106
H	6.486591	1.891371	-1.205846
H	7.601093	1.798695	0.186026
H	7.087981	0.297965	-0.634524

1a-T_1'(MeCN)

C	2.683024	-0.263567	0.259369
C	3.713505	-0.859358	-0.465005
C	2.772837	1.051685	0.702700
C	4.850146	-0.115178	-0.744824
H	3.629771	-1.887925	-0.798306
C	3.920938	1.779085	0.411699
H	1.963295	1.497873	1.269608
C	4.972681	1.210819	-0.311768
H	5.658920	-0.572249	-1.307594
H	3.999537	2.805865	0.755945
C	6.223228	1.994584	-0.604627
H	7.044993	1.662314	0.039819
H	6.544924	1.852507	-1.640569
H	6.074091	3.063242	-0.432176
S	1.219196	-1.188976	0.598186
O	0.508547	-0.583848	1.724610
O	1.532556	-2.619847	0.623658
N	0.288611	-0.952230	-0.838408
C	-0.999695	-1.463937	-0.801302
H	-1.083697	-2.505370	-1.109986
C	-2.151404	-0.679479	-0.547092
C	-3.434113	-1.270149	-0.625981
C	-2.084629	0.701608	-0.222320
C	-4.587180	-0.537599	-0.390809
H	-3.518328	-2.323987	-0.875075
C	-3.230603	1.430510	0.008436
H	-1.119093	1.192476	-0.156174
C	-4.493325	0.821500	-0.070142
H	-5.549085	-1.030782	-0.459723
H	-3.176697	2.486446	0.254012
O	-5.557652	1.627433	0.177418
C	-6.864041	1.069361	0.110946
H	-6.986827	0.268308	0.847167
H	-7.548172	1.884899	0.342258
H	-7.076853	0.686404	-0.892499
